# Fluoride as Ligand: Chemistry of Some New Terminal and Bridged Systems

**DOI:** 10.1002/open.201200046

**Published:** 2013-01-29

**Authors:** Torben Birk

**Affiliations:** [a] University of Copenhagen, Department of ChemistryUniversitetsparken 5, 2100 Copenhagen (Denmark) E-mail: chemistry@torbenbirk.dk

**Keywords:** chromium, fluoride bridges, inorganic polymers, manganese, metal-organic networks

**Awarding Institution:** University of Copenhagen, PhD School of Science (Denmark)

**Date Awarded:** June 22, 2012

**Supervisors:** Prof. Dr. Jesper Bendix, Department of Chemistry, University of Copenhagen

A comprehensive and unifying theme in this thesis has been the synthesis and characterization of new coordination polymers and metal-organic networks. The common structural organizing element for these systems is the fluoride ion, which, according to the Pearson classification, is a “hard” or “class a” ligand. Accordingly, it is expected that fluoride would be best suited as a bridging ligand in systems with correspondingly “hard” metal ions. The regions of the periodic table of primary relevance would thus be the early transition metals, but also main-group elements from group I and II (as well as Al^III^/Ga^III^, Si^IV^/Ge^IV^/Sn^II^;Sn^IV^ and P^V^/As^V^/Sb^V^) could be envisaged as centers in extended fluoride-bridged structures.

The studied systems are all based on the 3d–F unit where either Cr^III^ or Mn^III^ coordinates a minimum of one fluorido ligand. The 3d–F unit has been studied in proper mononuclear systems but also in polynuclear or polymeric systems connected through unsupported bridging by fluorido ligand(s). This bridging is of both homo- and, for chromium also, heterometallic character. For the latter type of systems, completely novel classes of compounds containing either alkali metals (3d–F–*n*s) or lanthanides (3d–F–4f) were synthesized and characterized structurally and magnetically.

A new synthetic route to systems with unsupported, bridging fluorido ligands is established by using kinetically robust Cr^III^ fluorido complexes such as *trans*-[Cr(py)_4_F_2_]^+^, *cis*-[Cr(phen)_2_F_2_]^+^, *cis*-[Cr(bpy)_2_F_2_]^+^ and *cis*-[Cr(phen)_2_(H_2_O)(F)]_2_^+^ (py=pyridine, phen=1,10-phenantroline, bpy=2,2′-bipyridine) as precursors. These metal-containing building blocks (or ligands) can be viewed as synthons for the fluoride-containing part of the final complex. The fluorido ligands in these robust synthons are fixed with respect to configuration, and due to the robustness of the synthons undesirable ligand substitution is avoided. This is particularly important as it prevents or retards metathesis reactions catalyzed by reaction partners, including precipitation reactions involving relatively insoluble simple fluorides. The stereochemical control of the disposition of the fluorido ligands in the starting materials provides the opportunity for controlling the structures of the resulting polynuclear or polymeric products. This fairly rare situation of stereochemical control is contingent on the preferential linear bridging by fluoride, which is a recurrent motif in the systems studied here and in agreement with established chemistry of fluoride as a bridging ligand. The applicability and generality of the method is exemplified by reaction with hard metal ions from different parts of the periodic table. A graphical overview of the investigated systems based on the synthetic route to systems with unsupported, bridging fluorido ligands is given in Scheme 1.

**Scheme 1:**
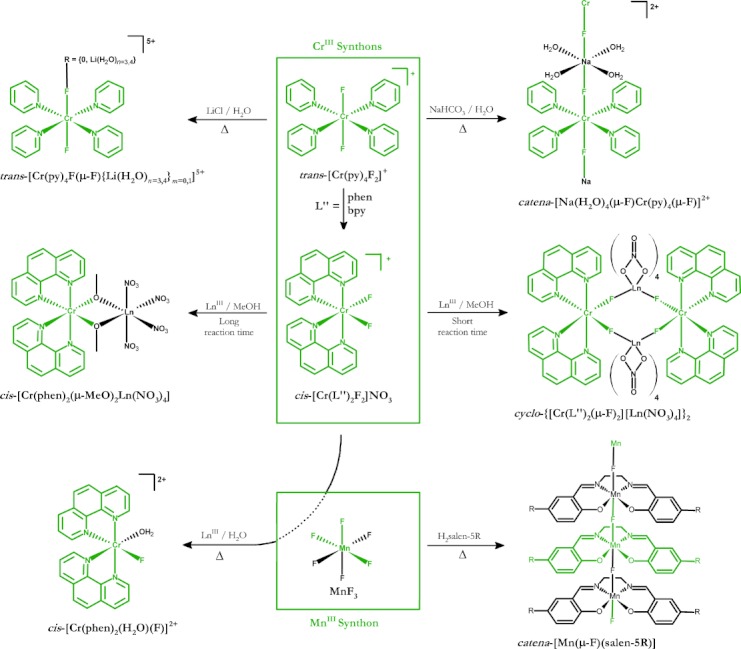
Overview of Cr^III^ and Mn^III^ fluorido complexes synthesized by the synthon approach.

The following sections discuss the overall results of the synthetic route to systems with unsupported, bridging fluorido ligands (3d–F–*n*s, 3d–F–3d and 3d–F–4f).

**3d–F–*n*s systems**: Alkali metal ions largely interact with the robust Cr^III^–fluoride synthon through second sphere coordination in solution. This interaction may, by suitable choice of a first coordination sphere synthon, allow for isolation of s-block complexes with a genuine coordinative dative bond from bridging fluoride ligands. Some structurally unprecedented systems containing the s-block ions Na^I^, Li^I^ and Cr^III^ bridged by the hard fluorido ligand have been isolated and characterized. The product formed for the two different s-block ions are quite different: For sodium, an infinite coordination polymer (1 D), *trans*-*catena*-poly[Na(H_2_O)_4_(μ-F)Cr(py)_4_(μ-F)](HCO_3_)_2_ results, while, for lithium, a discrete complex (0 D) of three different cations with variable coordination number, *trans*-{[Cr(py)_4_F_2_][Cr(py)_4_F(μ-F)Li(H_2_O)_3_][Cr(py)_4_F(μ-F)Li(H_2_O)_4_]}Cl_5_⋅6 H_2_O, was obtained. Despite the simple composition of the compounds synthesized, it is noteworthy that no immediate counterparts are found in the literature.

The difference in coordination geometry for the two different compounds elucidates the significance of size of the alkali metal ion in addition to the importance of the different tendency for hydrogen bond formation by the counter ions, as confirmed by the structural characterization. The structures of both compounds are dictated by the *trans* configuration of the starting material and are in agreement with the preference for approximately linear fluorido bridging (164.2–180.0 °) stated above.

In relation to the synthesis, a distinct perturbation of the Cr^III^-synthon spectra by the alkali metal ions in aqueous solution is found, demonstrating interaction through the coordinated fluorido ligands, which can be thought of as either bridge formation or second sphere coordination. Interestingly, the spectral shifts are invariably in the hypsochromic direction, which might be considered counterintuitive. This phenomenon has been rationalized based on density functional theory (DFT) calculations as a breakdown of the transferability of ligand field parameters between the unperturbed system and those with Lewis acids coordinating to the chromium-bound fluorido ligands.

**3d–F–3d systems**: High-spin Mn^III^ has dominated the work in molecule-based magnetism for more than a decade. Fluoride bridging is investigated for homometallic systems by combination with the well-investigated high-spin Mn^III^ center. The chain compounds *catena*-[Mn(μ-F)(salen-5R)] (R=H, F, Cl and salen-5H=2,2′-[ethane-1,2-diylbis(azanylylidenemethanylylidene)]diphenolato) were synthesized and structurally and magnetically characterized. *catena*-[Mn(μ-F)(salen-5H)] is structurally characterized and shows fluorido bridging with varying angle (150.4–180.0 °). For these systems an alternative synthetic strategy is employed, using MnF_3_ straightforwardly as starting material (Scheme 1).

Despite the very rich chemistry of the [Mn(salen)]^+^ fragment, *catena*-[Mn(μ-F)(salen-5H)] is the first and only structurally characterized example of a mono-atomic-bridged salen-ligated complex, not only for Mn^III^ but for all elements. This confers authority to the determination and the fundamental understanding of the fluorido ligands properties with respect to the magnetic interaction between two paramagnetic metal centers as compared to polyatomic ligands with or without supporting ligands. The magnetic properties of these systems demonstrate that the fluoride bridges, despite any preconceived notions, facilitate moderately strong magnetic interactions, when acting as linear bridges. The magnetic exchange coupling interaction across the fluorido bridge can be modeled by use of different theoretical methods with varying ranges of applicability, namely the Fisher model (*J*=38 cm^–1^ for *g*=2), exact diagonalization of finite-size rings (*J*=34-35 cm^–1^ for *g*=2) or the high-temperature expansion method (*J*≍32 cm^–1^).

*catena*-[Mn(μ-F)(salen-5H)] is also interesting, as the infinite chain structure is broken up into monomeric [Mn(F)(salen-5H)] in solution. Addition of fluoride in excess gives rise to in situ formation of the anion *trans*-[Mn(F)_2_(salen-5H)]^−^. Both [Mn(F)(salen-5H)] and *trans*-[Mn(F)_2_(salen-5H)]^−^ in solution show well-defined axial coordination of the fluorido ligands as determined by parallel-mode electron paramagnetic resonance (EPR) spectroscopy. On the basis of characterization of these two fluorido complexes, it was possible to resolve the superhyperfine interaction in the hexafluoridomanganate(III) ion, [MnF_6_]^3−^. This represents the first detailed determination of the superhyperfine interaction in a Mn^III^ complex.

**3d–F–4f systems**: The lanthanoids (Ln) from the f-block of the periodic table are classified as distinctly “hard” metal ions just like the cations of the s-block elements. This is reflected by a general preference for oxygen donor ligands, but also in their formation of sparingly soluble trifluorides, LnF_3_. Also in this context, using kinetically robust Cr^III^ synthons as a source for the bridging fluorido ligand in polynuclear systems was found not only to render a viable but also a very rewarding route. Isostructural series of tetranuclear compounds of the general formula *cyclo*-[(μ-F)Cr(L“)_2_Ln(μ-F)(NO_3_)_4_]_2_ (L”=phen, bpy and Ln=Ce, Pr, Nd, Sm, Eu, Gd, Tb, Dy) were synthesized by this approach (Figure 1). Common for these series of complexes is the square arrangement of two pairs of Cr^III^ and Ln^III^ linked by almost linear fluorido bridges (168.7 ° in *cyclo*-[(μ-F)Cr(phen)_2_Nd(μ-F)(NO_3_)_4_]_2_). These complexes are the first examples of unsupported fluorido bridges between lanthanoids and a transition metal (3d). The importance of this is that the unsupported bridge makes it possible to establish general conclusions concerning the role of the fluoride ligand, not only in determining the cluster structure but also in mediating magnetic interaction between paramagnetic 3d and 4f metal centers. The antiferromagnetic exchange interactions between Cr⋅⋅⋅Gd and Gd⋅⋅⋅Gd, respectively, could be analyzed and quantified for the *cyclo*-[(μ-F)Cr(phen)_2_Gd(μ-F)(NO_3_)_4_]_2_ complex and for related systems subsequently developed and studied by the group.

**Figure 1:**
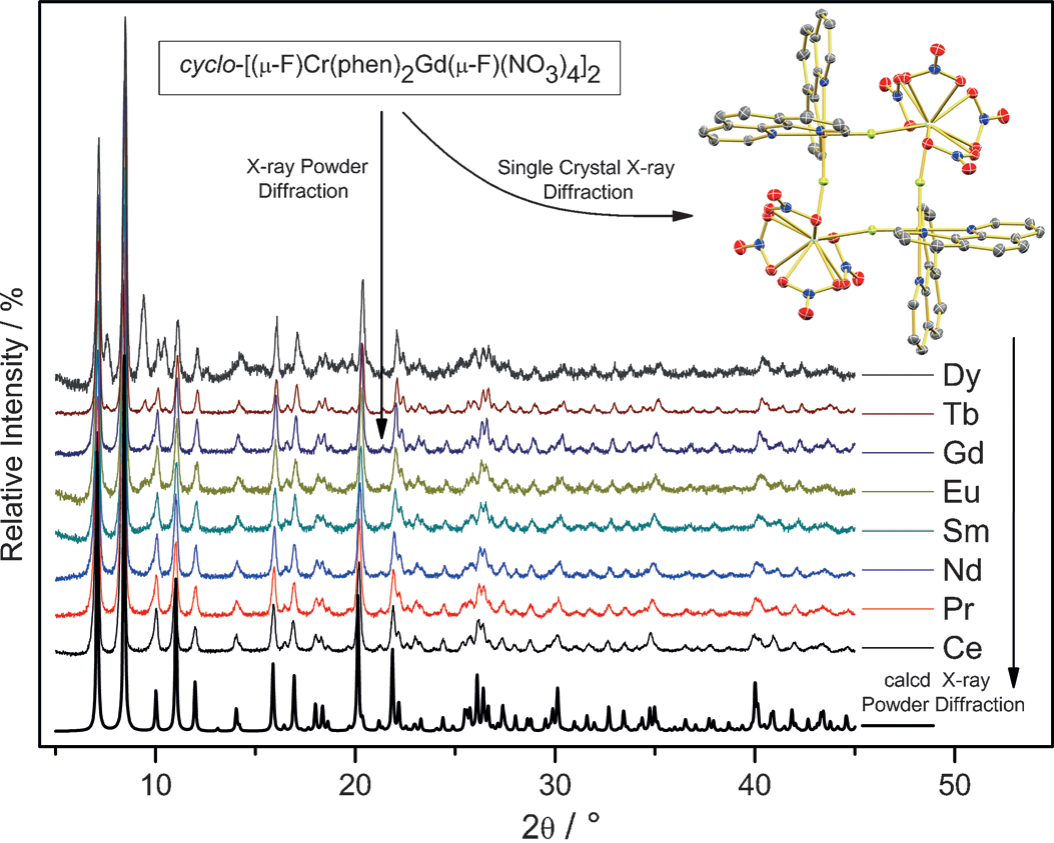
Molecular structure and powder X-ray diffractogram of *cyclo*-[(μ-F)(phen)_2_Cr(μ-F)Gd(NO_3_)_4_]_2_ together with powder X-ray diffractograms of the isomorphous series of *cyclo*-[(μ-F)(phen)_2_Cr(μ-F)Ln(NO_3_)_4_] for Ln=Ce–Dy.

The Cr^III^ synthons used are kinetically robust, but the reaction leading to insoluble lanthanoid fluorides may under appropriate reaction conditions lead to complete dissociation of the fluoride ligand(s). Such reactions were also observed and investigated. It was found that the bond cleavage proceeds in a controlled manner with formation of characterizable products. Accordingly, modification of the reaction conditions for the synthesis of tetranuclear *cyclo*-[(μ-F)Cr(L“)_2_Ln(μ-F)(NO_3_)_4_]_2_ systems towards long reaction times results in replacement of the fluorido ligands with methoxido ligands (MeO^−^) and concomitant formation of dinuclear methoxido-bridged systems of the general type [(phen)_2_Cr(μ-MeO)_2_Ln(NO_3_)_4_] (Ln=Nd, Tb, Dy). Structure determination confirms the methoxido ligand to facilitate the *η*^2^-coordination mode of the chromium-containing metallo ligand through formation of bent bridges in contrast to fluorido ligand-derived structures.

**Aquation of** ***cis*****-difluorido Cr^III^** **complexes**: Reacting lanthanoide nitrate, Ln(NO_3_)_3_**⋅***n* H_2_O with *cis*-[Cr(L“)_2_F_2_]NO_3_**⋅***n* H_2_O (L”=phen, bpy) in methanol at elevated temperature results in formation of the above-mentioned cyclic cluster species such as *cyclo*-[(NO_3_)_4_Ln(μ-F)Cr(L“)_2_(μ-F)]. Fairly small changes to the reaction conditions such as replacing the methanol solvent by a water/acetonitrile solvent mixture and replacing the counter ion with a noncoordinating ion, ClO_4_^−^, led to a completely different outcome of the reaction. Under these conditions the reaction exclusively proceeds as an aquation of the *cis*-[Cr(L”)_2_F_2_]^+^ cation, selectively forming *cis*-[Cr(L“)_2_(H_2_O)(F)]_2_^+^ which in its own right can be considered a synthon for monofluorido-bridged and/or mixed hydroxido-fluorido-bridged systems. The aquated system together with its precursor has been subject for independent studies regarding synthesis, reactivity and structural characterization.

## Supplementary material

As a service to our authors and readers, this journal provides supporting information supplied by the authors. Such materials are peer reviewed and may be re-organized for online delivery, but are not copy-edited or typeset. Technical support issues arising from supporting information (other than missing files) should be addressed to the authors.
