# Supplementary material for: Fluoride as Ligand: Chemistry of Some New Terminal and Bridged Systems
Source: ChemistryOpen. 2013 Jan 29;2(1):13–6. doi: 10.1002/open.201200046 (PMC3594586; doi:10.1002/open.201200046)
Supplement: Supplementary file 1 [file open0002-0013-SD1.pdf]

Open Access

ChemistryOPEN
Thesis Treasury

Supporting Information

© 2013 The Authors. Published by Wiley-VCH Verlag GmbH & Co. KGaA, Weinheim

Fluoride as Ligand: Chemistry of Some New Terminal and Bridged Systems

Torben Birk*[a]

open_201200046_sm_miscellaneous_information.pdf

The full thesis passed by an examination board of the awarding institution can be found below

WILEY-VCH ChemPubSoc Europe

FACULTY OF SCIENCE
UNIVERSITY OF COPENHAGEN

TORBEN BIRK

FLUORIDE AS LIGAND

Chemistry of some new terminal and bridged systems

PhD Thesis 2012

FLUORIDE AS LIGAND

CHEMISTRY OF SOME NEW TERMINAL AND BRIDGED SYSTEMS

TORBEN BIRK

COPENHAGEN 2012

DEPARTMENT OF CHEMISTRY
FACULTY OF SCIENCE
UNIVERSITY OF COPENHAGEN

FLUORIDE AS LIGAND. CHEMISTRY OF SOME NEW TERMINAL AND BRIDGED SYSTEMS

Torben Birk, Cand.scient.

ISBN: 978-87-7611-511-1

Print: SL Grafik, Frederiksberg, Denmark

© 2012 Torben Birk

Department of Chemistry
University of Copenhagen
Universitetsparken 5
DK-2100 Copenhagen Ø

Birk@kiku.dk

This thesis has been submitted to the Faculty of Science, University of Copenhagen in fulfilment of the requirements for the PhD degree in Chemistry.

Academic supervisor: Professor Jesper Bendix. Department of Chemistry, University of Copenhagen.

Submitted: March, 30 2012

Til dem jeg holder af

PREFACE

Fulgit super universum orbem
Lux amoris

This thesis entitled "*Fluoride as Ligand. Chemistry of some new Terminal and Bridged Systems*" has been submitted to the Faculty of Science, University of Copenhagen as a part of the requirement to achieve the PhD degree in Chemistry. The work presented was carried out at the Department of Chemistry at the University of Copenhagen from October 1, 2005 to December 1, 2008 (official time of study) and subsequently from December 1, 2008 to March 30, 2012 under the supervision of Professor Jesper Bendix and Dr. Høgni Weihe (during official time of study). I thank Dr. Høgni Weihe for his supervision and interest in the project. The road that was trodden to reach this final point has been littered with obstacles, and I would like to thank my supervisor Professor Jesper Bendix for his generous help, patience and inspiration to explore new areas of chemistry.

During my time as a PhD fellow I have worked at the Vienna Technical University with Dr. techn. Peter Weinberger in the group of Professor Wolfgang Linert at "Institut für Angewandte Synthesechemie". Both are thanked for giving me the opportunity for this stay abroad and work with new research areas.

The work performed during the time as a PhD student, and the results presented in this thesis would not have been possible without the help and cooperation of a number of people.
Among these should be mentioned the technical staff at the Department of Chemistry, University of Copenhagen especially Brigitta Kegel and Solveig Kallesøe Hansen for their tireless help with various problems and analysis of numerous samples.
Data from single crystal X-ray diffraction have been obtained with assistance from Flemming Hansen, Johan Vibenholt, Magnus Magnussen and Dr. Thomas Weyhermüller (Max-Planck-Institut für Bioanorganische Chemie, Mülheim). Dr. Susanne Mossin has helped with both magnetisation messurment at the facility in Erlangen as well as thermogravimetric measurements at the Technical University of Denmark. Magnetisation messurments have aditionally been performed in Copenhagen in collaboration with Kasper Steen Pedersen and Christian Aagaard Thuesen. All the above persons are thanked for their help.

The work has been presented at a number of conferences and meetings: "37th International Conference of Coordination Chemistry" August, 13-18 2006 Cape Town, South Africa. "22. Workshop on Novel Materials and Superconductors" February, 10-17 2007 Planneralm, Austria. "Gordon Research Conferences of Inorganic Chemistry" July, 13-18 2008 Salve Regina University, Newport.

I am grateful to The Faculty of Science, University of Copenhagen for granting me a 3 year PhD scholarship and The Danish Ministry of Science, Technology and Innovation for granting me a Elite Research Scholarship.

Copenhagen, March, 30 2012

Torben Birk

ABSTRACT

The thesis, "*Fluoride as Ligand. Chemistry of some new Terminal and Bridged Systems*" consists of two parts. First part (Part I: Thesis), the proper thesis, consists of an introduction to the research area together with a description of a selection of results obtained during the project. Second part (Part II: Publications) is a complete collection of the scientific papers (Paper 1–10) at present time published or submitted to internationally recognized journals. These papers constitute the formal yield of the project work.

A comprehensive and unifying theme of the project, has been the synthesis and characterization of new coordination polymers and metal-organic networks, especially with focus on their magnetic properties. For all these systems, the common structural organizing element is the fluoride ion, which according to the Pearson classification is a "hard" or "class a" ligand. The properties of fluoride in general and in particular as ligand in coordination complexes is treated in Chapter 2. The fluoride ligand enters into unsupported bridging between a number of hard metals from both the main groups (*s*-block), transition metals periods (*d*-block) and lanthanoids (*f*-block). Chapter 3 summarizes some general aspects of magnetochemistry of relavance to the systems studied in the thesis work and the associated papers.

A novel synthetic route to systems with unsuported, bridging fluorido ligands is established taking as employing kinetically robust Cr(III) fluorido complexes such as *trans*-$[Cr(py)_4F_2]^+$, *cis*-$[Cr(phen)_2F_2]^+$, *cis*-$[Cr(bpy)_2F_2]^+$ and *cis*-$[Cr(phen)_2(H_2O)(F)]^{2+}$ as precursors. These metal containing building blocks (or ligands) can be viewed as synthons for the fluoride containing part of the final complex. The fluorido ligands in these robust synthons are fixed with respect to configuration, and due to the robustness of the synthons undesirable ligand substitution is

avoided. This is particularly important, as it prevents or retards metathesis reactions catalyzed by reaction partners, including precipitation reactions involving relatively insoluble simple fluorides. The stereochemical control over the disposition of the fluorido ligands in the starting materials provides opportunity for controlling the structures of the resulting polynuclear or polymeric products. This fairly rare situation of stereochemical control is contingent on the preferential linear bridging by fluoride, which is a recurrent motif in the systems studied here and in agreement with established chemistry of fluoride as a bridging ligand. The applicability and generality of the method is excemplified by reaction with hard metal ions from different parts of The Periodic Table.

From the *s*-block Na(I) and Li(I) are represented in the 1D system *trans-catena*-poly$[Na(H_2O)_4(\mu\text{-}F)Cr(py)_4(\mu\text{-}F)](HCO_3)_2$ and the 0D system *trans*-$[Cr(py)_4F(\mu\text{-}F)\{Li(H_2O)_{n=3,4}\}_{m=0,1}]Cl_5{\cdot}6H_2O$, respectively (See Chapter 5 and Paper 4). The two different compounds exhibit differences in coordination geometry, in that the Na(I) ions in the former enter into infinite chain formation while the Li(I) ions in opposite occur as discrete entities with variable coordination number. This difference elucidate the significance of size of the alkali metal ion in addition to the importance of the different tendency for hydrogen bond formation by the counter ions , as confirmed by the structual charaterization. The structures of both compounds are dictated by the *trans* configuration of the starting material and are in agreement with the preference for approximatly linear fluorido bridging (164.2 ° til 180.0 °) stated above. Spectropic studies demonstrate that the fluoride bridging occurs also in solution and is thus not a property of the solid state. Thus, by UV/vis spectroscopy a distinct perturbation of the Cr(III) synthon spectra by hard metal ions is found, demonstrating interaction via the coordinated fluorido ligands, which can be thought of as bridge formation or second sphere coordination as one prefers. Interestingly, the spectral shifts are invariably in the hypsochromic direction, which might be considered counterintuitive. An explanation of this phenomenon based on DFT calculations, which is different from the retionalization hitherto invoked in the literature is forwarded in Paper 4.

The lanthanoids (in the following abbreviated Ln) from the *f*-block of The Periodic Table are classified as distinctly "hard" metal ions just as the cations of the *s*-block elements. This is reflected by a general preference for oxygen donor ligands, but also in their formation of sparingly soluble trifluorides, LnF_3. Also in this context, the use of kinetically robust Cr(III) synthons as source for the bridging fluorido ligand in polynuclear systems was found not only a viable, but indeed a very rewarding route. Thus, isostructural series of tetra nuclear compounds of the general formula *cyclo*-$[(\mu\text{-}F)Cr(L'')_2Ln(\mu\text{-}F)(NO_3)_4]_2$ (L''=phen, bpy and Ln=Ce, Pr, Nd, Sm, Eu, Gd, Tb, Dy) were synthesized by this approach (See Chapter 7 and Paper 6, 7). Common for these series of complexes are the square arrangement of to pair Cr(III) and Ln(III) linked by approximatly linear fluorido bridges (168.7 ° in *cyclo*-$[(\mu\text{-}F)Cr(phen)_2Nd(\mu$-

F)$(NO_3)_4]_2$). These complexes are the first examples of unsuported fluorido bridges between lanthanoids and a transition metal (3*d*). The importance of this aspect lies in that the unsuported bridge makes it possible to establish general conclusions concerning the role of the fluoride ligand not only in determining the cluster structure, but also in mediating magnetic interaction between paramagnetic 3*d* and 4*f* metal centers. The aniferromagnetic exchange interactions between Cr–Gd and Gd–Gd respectively, could be analyzed and quantified for the *cyclo*-[(μ-F)Cr$(phen)_2$Gd(μ-F)$(NO_3)_4]_2$ complex and for related systems subsequently developed and studied by the group. (See Paper 7)

Although the Cr(III) synthons used are kinetically robust, the reaction leading to insoluble lanthanoid fluorides, which is probably thermodynamically favored in general, can at appropriate reaction conditions leads to complete dissociation of the fluoride ligand(s). Such reactions were also observed and investigated. It was found that the bond cleavage proceeds in a controlled manner with formation of characterizable products. Accordingly, modification of the reactions conditions for the synthesis of the tetra nuclear *cyclo*-[(μ-F)Cr(L")$_2$Ln(μ-F)$(NO_3)_4]_2$ systems towards long reaction times result in replacement of the fluorido ligands with methoxido ligands (MeO^-) and concomitant formation of dinuclear methoxido bridged systems of the general type [(phen)$_2$Cr(μ-MeO)$_2$Ln$(NO_3)_4$] (Ln=Nd, Tb, Dy) (See Chapter 8 and Paper 8). Structure determination confirm the methoxido ligand to facilitate the η^2-coordination mode of the chromium containing metallo-ligand througn the formation of bent bridges in contrast to the fluorido ligand derived structures.
By reaction of the Cr(III) synthons with lanthanoid ions at elevated temperatures aquation one of the two fluorido ligands in *cis*-$[Cr(L")_2F_2]^+$ occurs selectively with formation of *cis*-$[Cr(L")_2(H_2O)(F)]^{2+}$, which is in its own right can be considered a synthon for mono fluorido bridged and/or mixed hydroxido-fluorido bridged systems. The aquated system together with its precursor have been subjects for independent studies with respect to synthesis, reactivity and structural charaterization (See Chapter 6 and Paper 2, 3).

High-spin manganese(III) has dominated the work in molecule based magnetism for more than a decade. In this thesis, fluoride bridging is also investigated for homo-metallic systems by combination with the well investigated H.S. Mn(III) centre. The chain compounds *catena*-[Mn(μ-F)(salen-5R)] (R=H, F, Cl, “Br”) have thus been synthesized and structurally and magnetically characterized. *catena*-[Mn(μ-F)(salen-5H)] is structural charaterized and shows fluorido bridged with variating angle (150.4 ° to 180.0 °). Although metal complexes of the salen ligand are extensively studied, the 1D chain structure of these compounds with monoatomic bridging ligands is completely unprecedented. For these systems an alternative strategy is employed using straightforwardly MnF_3 as starting material (See Chapter 4 and Paper 1, 5). EPR investigations, in conjunction with studies on the $[MnF_6]^{3-}$ ion, have shown that the chain dissolves by symmetric clevage into monomeric mono fluorido complexes. Additionally, a simple dinuclear fluorido complex $[(Me_3tacn)MnF_2(\mu\text{-}F)MnF_2(Me_3tacn)]^+$ was

synthesized by the same approach and the magnetic exchange in this and the fluorido-bridged chain was quantified and found to be quite sizeable and of approximately the same magnitude (32.7 and 38 cm^{-1}, respectively).

RESUME PÅ DANSK

Denne afhandling ”*Fluoride as Ligand: Chemistry of some new Terminal and Bridged Systems*” består af to dele. Første del (Part I: Thesis), der er den egentlige afhandling, udgøres af en introduktion af forskningsområdet samt en beskrivelse af et udvalg af de resultater der er opnået under projektarbejdet. Anden del (Part II: Publications) er en fuldstændig samling af de videnskabelige artikler (Paper 1–10), der på nuværende tidspunkt er enten offentliggjorte eller indsendt til anerkendte videnskabelige tidsskrifter. Disse artikler udgør det formelle udbytte af projektarbejdet.

Et overordnet og samlende tema for projektarbejdet har været syntese og karakterisering af nye koordinationspolymerer og metal-organiske netværk, specielt med henblik på deres magnetiske egenskaber. For alle de beskrevne systemer er det fælles strukturelt organiserende element fluoridionen, der ifølge Pearsons klassifikation er en ”hård” eller ”klasse a” ligand.
Egenskaberne af fluorid generelt, og specielt som ligand i koodinationskomplekser, er omtalt i kapitel 2. Fluoridliganden indgår i ikke-understøttet brodannelse mellem en række hårde metaller fra både hovedgrupperne (*s*-blokken), overgangsmetalperioderne (*d*-blokken) og lanthanoidrne (*f*-blokken). Kapitel 3 opsummerer forskellige generelle aspekter magnetokemien af relevans for de systemer der undersøges i denne afhandling, samt de tilknyttede artikler.

En ny syntesemetode til systemer med ikke-understøttede brodannende fluorido ligander er etableret ved at benytte kinetisk robuste Cr(III) fluoridokomplekser såsom *trans*-$[Cr(py)_4F_2]^+$, *cis*-$[Cr(phen)_2F_2]^+$, *cis*-$[Cr(bpy)_2F_2]^+$ og *cis*-$[Cr(phen)_2(H_2O)(F)]^{2+}$ som udgangsmaterialer. Disse metalindholdende byggesten (eller ligander) kan opfattes som synthoner for den fluoridindholdende del af det endelige kompleks. Fluoridoliganderne er i disse robuste

synthoner fikseret med hensyn til konfiguration, og som følge af robustheden af synthonerne undgås uønsket ligandsubstitution. Dette er specielt vigtigt idet metathesereaktioner, katalyseret af andre reaktanter forsinkes eller undgås, bl.a. fældningsreaktioner indvolverende relativt uopløselige simple fluorider.
Stereokemisk kontrol af fluoridoligandens placering i udgangsmaterialet giver mulighed for at kontrollere strukturen af det endelige polynukleare eller polymere produkt. Denne forholdsvis sjældne situation mht. stereokemisk kontrol er betinget af fluorids foretrukne lineære brodannelse, hvilket er tilbagevendende motiv for de undersøgte systemer og i overensstemmelse med fluorids etablerede kemi som brodannende ligand.
Anvendelsesmulighederne og metodens almenhed er eksemplifiseret ved reaktion med hårde metalioner fra forskellige dele af det periodiske system.

Fra *s*-blokken er Na(I) and Li(I) repræsenteret henholdsvist ved 1D systemet *trans*-*catena*-poly[$Na(H_2O)_4$(μ-F)$Cr(py)_4$(μ-F)]$(HCO_3)_2$ og 0D systemet *trans*-[$Cr(py)_4$F(μ-F){$Li(H_2O)_{n=3,4m=0,1}$]$Cl_5 \cdot 6H_2O$, (se Kapitel 5 og Artikel 4). De to forbindelser udviser forskellige koordinationsgeometri, idet Na(I) ionen i førstnævnte kompleks indgår i en uendelig kædedannelse, mens Li(I) ionen modsat forekommer som diskrete entiteter med varierende koordinationstal. Denne forskel tydeliggør betydningen af dels alkalimetalionens størrelse, dels modionernes forskellige tendens til hydrogenbindingsdannelse hvilket bekræftes ved strukturel karakterisering. Strukturerne af begge forbindelser er domineret af udgangsmaterialets *trans* konfiguration og i overensstemmelse med tidligere anførte præference for tilnæmelsesvist lineære fluoridobroer (164,2 ° til 180,0 °). Spektroskopiske undersøgelser viser at fluorids brodannelse også forekommer i opløsning og således ikke er en fastfaseegenskab. Ved UV/vis spektroskopi vises en distinkt perturbation af Cr(III)synthonet ved tilsætning af hårde metalioner hvilket viser interaktion gennem den koordinerede fluoridoligand, hvilket kan opfattes som enten en brodannelse eller en 2. sfære koordination. Bemærkelsesværdigt går det spektrale skifte altid i hypsochrom retning, hvilket kan opfattes som ulogisk. En forklaring af dette fænomen baseret på DFT beregninger er givet i Artikel 4 og adskiller sig fra den rationalisering der kendes i litteraturen.

Lanthanoidrne (i det følgende forkortet Ln) fra *f*-blokken af det periodiske system klassificeres som udpræget "hårde" metalioner ligesom kationerne af *s*-blok grundstofferne. Dette afspejles i en generel preference for oxygendonorligander, men også i deres dannelse af svagt opløselige trifluorider, LnF_3. Også i denne forbindelse blev brugen af kinetisk robuste Cr(III) synthoner, som kilde til den brodannende fluoridoligand i polynukleare systemer, fundet at være en ikke alene brugbar, men også meget udbytterig metode. Således blev en isostrukturel serie af tetranukleare forbindelser af generel form *cyclo*-[(μ-F)Cr(L")$_2$Ln(μ-F)$(NO_3)_4$]$_2$ (L"=phen, bpy og Ln=Ce, Pr, Nd, Sm, Eu, Gd, Tb, Dy) syntetiseret ved brug af denne strategi (se Kapitel 7 og Artikel 6, 7). Fælles for denne serie af komplekser er det kvadratiske arrangement af de to par

Cr(III) og Ln(III) forbundet af tilnærmelsesvist lineære fluoridobroer (168,7 ° i *cyclo*-[(μ-F)Cr(phen)$_2$Nd(μ-F)(NO$_3$)$_4$]$_2$). Disse komplekser er det første eksempel på ikke understøttet fluoridobrodannelse mellem lanthanoidr og et overgangsmetal (3*d*). Betydningen heri består i at den ikke understøttede bro gør det muligt at drage generelle konklusioner vedrørende fluoridligandens rolle ikke kun i bestemmelse af klyngestrukturen, men også i formidlingen af magnetisk interaktion mellem paramagnetiske 3*d* og 4*f* metal centre. Den antiferromagnetiske exchange interaktion mellem henholdsvis Cr–Gd og Gd–Gd kunne analyseres og kvantificeres for *cyclo*-[(μ-F)Cr(phen)$_2$Gd(μ-F)(NO$_3$)$_4$]$_2$ komplekset og tilsvarende systemer efterfølgende udviklet og undersøgt af forskningsgruppen (Se Artikel 7).

Selvom den benyttede Cr(III)synthon er kinetisk robust kan reaktioner, der fører til dannelse af de sandsynligvis termodynamisk farvoriserede uopløselige lanthanoidfluorider, ved passende reaktionsbetingelser medføre fuldstændig dissociation af en til to af fluoridliganderne. Sådanne reaktioner blev også iagttaget og undersøgt. Det blev fundet, at bindingsbrydningen forløb på kontrolleret vis under dannelse af karakteriserbare produkter. I overensstemmelse hermed fandtes, at modifikation af reaktionsbetingelserne mod længere reaktionstid for syntesen af det tetranukleare *cyclo*-[(μ-F)Cr(L")$_2$Ln(μ-F)(NO$_3$)$_4$]$_2$ system resulterede i udskiftning af fluoridoliganderne med methoxidoligander (MeO$^-$) under samtidig dannelse af det dinukleare methoxid-brosystem af den generelle type [(phen)$_2$Cr(μ-MeO)$_2$Ln(NO$_3$)$_4$] (Ln=Nd, Tb, Dy) (se Kapitel 8 og Artikel 8). Strukturbestemmelse bekræfter at methoxidoliganden faciliterer η^2-koordination af den chromindholdende metalloligand gennem dannelse af en vinklet bro. Dette står i modsætning til afledte strukturer af fluoridoliganden.
Ved reaktion af Cr(III) synthonerne med lanthanoidioner i varme indtræder selektiv aquation af en af de to fluoridoligander i *cis*-[Cr(L")$_2$F$_2$]$^{2+}$ under dannelse af *cis*-[Cr(L")$_2$(H$_2$O)(F)]$^{2+}$, der selvstændigt kan betragtes som synthon for monofluoridobroer og/eller blandede hydroxido-fluoridobrosystemer. Det aquatiserede system sammen med sin precursor har været genstand for selvstændige undersøgelser med hensyn til syntese, reaktivitet og strukturel karakterisering (se Kapitel 6 og Artikel 2, 3).

Højspin Mn(III) har domineret arbejdet indefor molekylbaseret magnetisme i mere end 10 år. I denne afhandling undersøges fluorid brodannelse i tilfælde af homometalliske systemer kombineret med velkendte højspin Mn(III) centre. Kædeforbindelsen *catena*-[Mn(μ-F)(salen-5R)] (R=H, F, Cl, ”Br”) er således syntetiseret samt strukturelt og magnetisk karakteriseret. *catena*-[Mn(μ-F)(salen-5H)] er strukturelt karakteriseret og viser fluoridobroer med varierende bindingsvinkel (150,4 ° til 180,0 °). Selvom metalkomplekser af salen liganden er undersøgt i vid udstrækning, er 1D kædestrukturen i disse forbindelser med monoatomiske broligander hidtil ukendt. Hvad angår disse systemer, anvendes en alternativ syntesestrategi med MnF$_3$ som udgangsmateriale (se Kapitel 4 og Artikel 1, 5). EPR undersøgelser i sammenhæng med undersøgelser af [MnF$_6$]$^{3-}$ ionen har vist, at kæden ved opløsning kløves symmetrisk i monomere monofluoridokomplekser. Derudover er et simpelt dinukleart fluoridokompleks

$[(Me_3tacn)MnF_2(\mu\text{-}F)MnF_2(Me_3tacn)]^+$ blevet syntetiseret ved brug af samme metode. Den magnetiske exchange i denne og fluorido-bro kædeforbindelsen blev kvantificeret og fundet at være relativt store og af omtrent samme størrelse (32,7 og 38 cm^{-1}).

ABOUT THE AUTHOR

Torben Hilde Birk was born 1978 in Denmark as only child of optometrist Benny Birk (*1946) and shop assistant Nina Hilde Birk (*1946). Most of his childhood was spent in the town Birkerød in northern Zealand, where he early became interested in natural sciences, neo-classical music and heraldry[1].

He graduated high school (Gymnasium) at Marie Kruse in 1998 and he got matriculated the same year at the University of Copenhagen with studies in Chemistry. The bachelor degree in Chemistry (B.Sc.) was achieved in 2002 within the field of theoretical chemistry with Professor Kurt V. Mikkelsen as supervisor. He then continued his chemistry studies in the field of Inorganic Chemistry with Professor Jesper Bendix as supervisor. The interest in coordination chemistry was awakened and he completed his studies with a dissertation for the Candidatus Scientiarum degree (Cand.scient.) in Chemistry about Terminal Nitrido Complexes of Chromium(V).

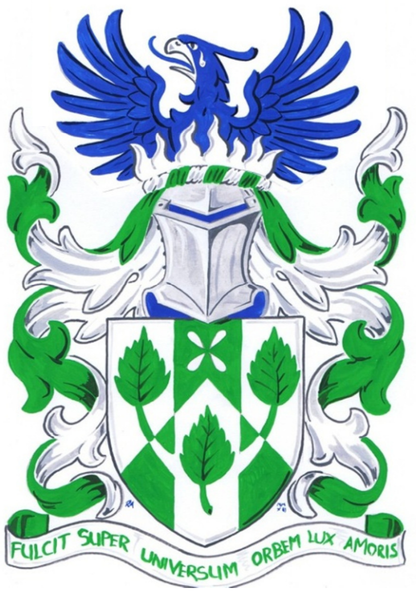

[1] The author's coat of arms shown to the right is still in preparation. This coat of arms is made in collaboration with Royal Herald Painter Ronny Andersen.

CONTENTS

PREFACE i

ABSTRACT iii

RESUME PÅ DANSK vii

ABOUT THE AUTHOR xi

CONTENTS xiii

LIST OF FIGURES xxi

LIST OF TABLES xxv

PART I: THESIS 1

1 INTRODUCTION 3

AIMS OF WORK ... 3

Synthetic strategy for new terminal and bridged fluorido complexes ... 4

STRUCTURE AND CONTENT OF THESIS ... 6

2 FLUORIDE AS LIGAND IN COORDINATION COMPLEXES 9

INTRODUCTION ... 9

CHEMICAL PROPERTIES AND REACTIVITY ... 10

ELECTRONEGATIVITY AND HARDNESS ... 12

CLASSES OF FLUORINE CONTAINING COMPOUNDS ... 15

Stabilization of fluoride containing lattices ... 16
Fluoride as terminal ligand ... 17
Fluoride as bridging ligand ... 19

ELECTRONIC PROPERTIES OF FLUORIDE AS LIGAND ... 23

3 MAGNETISM AND THERMODYNAMICS 27

INTRODUCTION ... 27

MODELS OF MAGNETIC BEHAVIOR ... 28

Magnetization and magnetic susceptibility ... 29
Spin-Hamiltonians ... 30
Application of the Spin-Hamiltonian formalism ... 32
Analysis of data ... 35
Analysis of data for infinite systems ... 35

EXCHANGE MECHANISMS AND SOME SELECTED DATA ... 36

Magnetic exchange in fluorido complexes ... 37

4 Mn(III) FLUORIDO COMPLEXES – CHAINS AND DISCRETE SYSTEMS 39

INTRODUCTION ... 39

THE CHEMISTRY OF Mn(III) 41
Ligand spheres encompassing fluorine ligator atoms 41
Ligand spheres encompassing oxygen ligator atoms 43
Ligand spheres encompassing nitrogen ligator atoms 45
Ligand spheres encompassing mixed oxygen and nitrogen ligator atoms 45
Schiff bases and related complexes 45
Applications Schiff-base complexes of Mn(III) 46
Catalytic behaviour 47
Biological systems and medicine 48
JAHN-TELLER EFFECTS IN Mn(III) COMPLEXES 49
Structural manifestation of the Jahn-Teller effect 50
Magnetic manifestation of the Jahn-Teller effect 52
SYNTHETIC STRATEGY FOR 3*d*-(μF)-3*d* BRIDGED SYSTEMS 56
General methods for the synthesis of [Mn(X)(salen)] and [Mn(X)(SB)] 56
1. Method: MnY_3 as precursor 57
2. Method: [Mn(Y)(salen)] as precursor 58
3. Method: Ligand substitution 59
Other methods 59
Conclusion 60
Development of a new method for synthesis of *catena*-[Mn(μ-F)(salen-5R)] .. 60
Development of a method for synthesis of *trans*-$[Mn(F)_2(salen\text{-}5H)]^-$ 65
STRUCTURAL DESCRIPTION OF *catena*-[Mn(μ-F)(salen-5R)] 67
Introduction 67
Discrete [Mn(X)(SB)] and [Mn(X)(S)(SB)] 67
1D Infinite coordination polymers *catena*-[Mn(μ-X)(SB)] 70
Discrete dimers $[Mn_2(SB)_2(S)_2(\mu\text{-}X)]$ 70
"Out of plane" dimers $[Mn_2(SB)_2(S)_2]$ 70
Structure of *catena*-[Mn(μ-F)(salen-5R)] for R=H, F, Cl 71
MAGNETIC BEHAVIOR AND EPR-SPECTROSCOPY OF 1D Mn(III) 76
EPR-spectroscopy on solutions of *catena*-[Mn(μ-F)(salen-5H)] 76
Qualitative description 76
Quantitative description by Spin-Hamiltonian formalism 79
Magnetic behaviour of *catena*-[Mn(μ-F)(salen-5H)] 80
EXPERIMENTAL DETAILS 86
General comments 86

Synthesis of H_2salen-5R for R=H, F, Cl, Br 87
R = H 87
R = F 87
R = Cl 88
R = Br 88
Synthesis of *catena*-[Mn(μ-F)(salen-5H)] 89
Synthesis of *catena*-[Mn(μ-F)(salen-5F)] 90
Synthesis of *catena*-[Mn(μ-F)(salen-5Cl)] 91
Synthesis of *catena*-[Mn(μ-F)(salen-5Br)] 92
Crystals for X-ray diffraction of *catena*-[Mn(μ-F)(salen-5R)] for R=H, F, Cl. 93
catena-[Mn(μ-F)(salen-5H)] 93
catena-[Mn(μ-F)(salen-5F)] 93
catena-[Mn(μ-F)(salen-5Cl)] 93

5 ALKALI METAL CATION COMPLEXATION BY Cr(III) FLUORIDO COMPLEXES 95

INTRODUCTION 95

The synthon approach 96

SECOND SPHERE COORDINATION IN Cr(III) FLUORIDO COMPLEXES 97

Introduction 97

FLUORIDO BRIDGES BETWEEN *d*- AND *s*-BLOCK METALS 98

The 3*d*-(μ-F)-n*s* bonding motif 98

SYNTHETIC STRATEGY FOR 3*d*-(μF)-n*s* BRIDGED SYSTEMS 100

Synthons with two fluorido ligands, *cis*/*trans*-[Cr(L')$_2$F$_2$] 100
Synthons with three fluorido ligands, *mer*/*fac*-[Cr(L‴)F$_3$] 103

STRUCTURAL DESCRIPTION 104

Single crystal structure of *trans*-*catena*-poly[Na(H_2O)$_4$(μ-F)Cr(py)$_4$(μ-F)](HCO$_3$)$_2$ 104
Structure of the formula unit 104
Packing of unit cell 107
Hydrogen bonding 108
Structural description of *trans*-[Cr(py)$_4$F(μ-F){Li(H_2O)$_{n=3,4}$}$_{m=0,1}$]Cl$_5$·6H_2O .. 110
Molecular structure 110
Hydrogen bonding 112

EXPERIMENTAL DETAILS 114

General comments 114
Synthesis of *trans-catena*-poly[$Na(H_2O)_4(\mu\text{-}F)Cr(py)_4(\mu\text{-}F)](HCO_3)_2$ 115
Crystals for single crystal diffraction 115
Reactivity and use of
trans-catena-poly[$Na(H_2O)_4(\mu\text{-}F)Cr(py)_4(\mu\text{-}F)](HCO_3)_2$ 116
Degradation of
trans-catena-poly[$Na(H_2O)_4(\mu\text{-}F)Cr(py)_4(\mu\text{-}F)](HCO_3)_2$ into constituent 116
Synthesis of *trans*-[$Cr(py)_4F_2$]X from *trans*-[$Cr(py)_4F_2$]HCO_3 117
Synthesis of *trans*-[$Cr(py)_4F(\mu\text{-}F)\{Li(H_2O)_{n=3,4}\}_{m=0,1}$]$Cl_5\cdot 6H_2O$ 118
Crystals for single crystal diffraction 118

6 **FLUORIDE LABILITY: AQUATION OF *cis*-DI-FLUORIDO Cr(III) COMPLEXES** 119

INTRODUCTION 119

DIFLUORIDO COMPLEXES, [$Cr(L'')_2F_2$]$^+$ 120

AQUATION REACTIONS IN [$Cr(L'')_2F_2$]$^+$ 122

Reaction kinetic in aquation reaction 122
Synthesis of mixed aqua-fluorido complexes 123
Anation - Reactivity of [$Cr(L'')_2(H_2O)(F)$]$^{2+}$ 124

AQUATION OF DIFLUORIDO COMPLEXES BY USE OF LANTHANOIDS 126

STRUCTURAL DESCRIPTION OF *cis*-DI-FLUORIDO AQUATION COMPLEXES 128

Structural characterized complexes in literature 128
Single crystal structural description of two *cis*-di-Fluorido aquation 130
Crystal packing 132
Trans influenced bond lengths in *cis*-[$Cr(L'')_2F_2$]ClO_4 134
Hydrogen bonding and binuclear units 134
Configuration of [$Cr(L'')_2F_2$]$^+$ and [$Cr(L'')_2(H_2O)(F)$]$^{2+}$ 139
X-ray powder analysis of *cis*-[$Cr(L'')_2(H_2O)(F)$]$(ClO_4)_2$ 139

ATTEMPTS AT SYNTHESIS OF DI-μ-FLUORIDO BRIDGED COMPLEXES 141

Oxygen bridged Cr(III) complexes 141
Conformation and possibility of fluorido bridged complexes 143
Thermolysis of *cis*-[$Cr(L'')_2(H_2O)(F)$]$(ClO_4)_2\cdot nH_2O$ 144

EXPERIMENTAL DETAILS 146

General comments ... 146
In situ generation of $Ln(ClO_4)_3{\cdot}nH_2O$ ... 147
Synthesis of *cis*-$[Cr(L'')_2(F)(H_2O)](ClO_4)_2{\cdot}nH_2O$ for L''=phen, bpy ... 148
cis-$[Cr(phen)_2(F)(H_2O)](ClO_4)_2{\cdot}nH_2O$ 148
cis-$[Cr(bpy)_2(F)(H_2O)](ClO_4)_2$ 149
Comment on the water content of the compounds 149
Crystals for X-ray diffraction of aquation *cis*-di-fluorido Cr(III) complexes . 150
cis-$[Cr(phen)_2F_2]ClO_4{\cdot}H_2O$ 150
cis-$[Cr(bpy)(F)(H_2O)](ClO_4)_2{\cdot}2H_2O$ 150
Synthesis $[(L'')_2Cr(\mu\text{-}F)_2Cr(L'')_2](ClO_4)_4$ for L''=phen, bpy ... 151
$[(phen)_2Cr(\mu\text{-}F)_2Cr(phen)_2](ClO_4)_4$ 151
$[(bpy)_2Cr(\mu\text{-}F)_2Cr(bpy)_2](ClO_4)_4$ 151

7 METHOXIDO-BRIDGED Ln(III)-Cr(III) SYSTEMS OBTAINED BY FLUORIDE ABSTRACTION 153

INTRODUCTION ... 153
STRUCTURAL DISCUSSION ... 154
MAGNETIC PROPERTIES ... 156
PERSPECTIVES ... 157
EXPERIMENTAL DETAILS ... 158
General comments ... 158
Synthesis of *cis*-$[Cr(phen)_4F_2]NO_3{\cdot}nH_2O$ ... 159
Synthesis of *cis*-$[Cr(bpy)_4F_2]NO_3$ ... 159
Synthesis of $[(phen)_2Cr(\mu\text{-}MeO)_2Ln(NO_3)_4]$ for Ln=Nd, Sm, Gd ... 160

8 FLUORIDE BRIDGING AS A STRUCTURE-DIRECTING MOTIF IN 3*d*-4*f* COMPLEXES 161

INTRODUCTION ... 161
STRUCTURAL DISCUSSION ... 162
EXPERIMENTAL DETAILS ... 165
General comments ... 165
General synthesis of *cyclo*-$[(\mu\text{-}F)(phen)_2Cr(\mu\text{-}F)Ln(NO_3)_4]$ ... 166
Genearl synthesis of *cyclo*-$[(NO_3)_4Ln(\mu\text{-}F)Cr(bpy)_2(\mu\text{-}F)]_2$ ... 168

9 CONCLUSION AND OUTLOOK 169

CONCLUSION 169

FINAL COMMENT AND OUTLOOK 170

10 APPENDIX 1: ABBREVIATIONS & UNITS 173

GENERAL COMMENTS 173

Abbreviations of ligands 173
Physical quantities and their units 174

TABLES OF ABBREVIATIONS 175

SYSTEMATIC NAMING OF COMPLEXES DESCRIBED IN THESIS 183

11 APPENDIX 2: INSTRUMENTARIUM 185

GENERAL COMMENTS 185

Elementary analysis 186
Carbon, Hydrogen and Nitrogen 186
Other elements 186
Electron Paramagnetic Resonance spectroscopy (EPR) 186
Infrared spectroscopy (IR) 186
Ultraviolet / visual spectroscopy (UV/vis) 187
Mass spectrometric methods 187
Magnetic susceptibility 187
Preparation of sample 188
Pascal's constants 188
Comment 189
Thermogravimetric analysis (TGA) 189

12 APPENDIX 3: X-RAY DIFFRACTION 191

GENERAL COMMENTS 191

SINGLE CRYSTAL DIFFRACTION 192

Special details for the refinement and presentation 193
Geometry 193
Refinement 193
Comparison of crystallographic data 194
Molecular graphics and presentation 194

TABELLARIUM: SINGLE CRYSTAL X-RAY DIFFRACTION DATA 195

X-ray data No. 1: *catena*-[Mn(μ-F)(salen-5H)] 195

X-ray data No. 2: *catena*-[Mn(μ-F)(salen-5F)] ... 196
X-ray data No. 3: *catena*-[Mn(μ-F)(salen-5Cl)] ... 197
X-ray data No. 4: *trans*-*catena*-poly[$Na(H_2O)_4$(μ-F)$Cr(py)_4$(μ-F)]$(HCO_3)_2$ ... 198
X-ray data No. 5: *trans*-[$Cr(py)_4F$(μ-F){$Li(H_2O)_{n=3,4}$}$_{m=0,1}$]$Cl_5 \cdot 6H_2O$ ... 199
X-ray data No. 6: *cis*-[$Cr(phen)_2F_2$]$ClO_4 \cdot H_2O$ ... 200
X-ray data No. 7: *cis*-[$Cr(bpy)_2(H_2O)(F)$]$(ClO_4)_2 \cdot 2H_2O$ ... 201
X-ray data No. 8: *cyclo*-[$(NO_3)_4$Nd(μ-F)$Cr(phen)_2$(μ-F)]$_2$ ... 202
X-ray data No. 9: [$(NO_3)_4$Nd(μ-MeO)$_2$$Cr(phen)_2$] ... 203

POWDER X-RAY DIFFRACTION ... 204

BIBLIOGRAPHY **205**

PART II: PUBLICATIONS **251**

GENERAL COMMENTS ... 253

List of publication related to thesis ... 253
List of other publications ... 254

PAPER 1 ... 255

PAPER 2 ... 261

PAPER 3 ... 269

PAPER 4 ... 277

PAPER 5 ... 289

PAPER 6 ... 301

PAPER 7 ... 309

PAPER 8 ... 321

PAPER 9 ... 343

PAPER 10 ... 351

LIST OF FIGURES

Figure 1 Types of terminal and bridged fluorido complexes studied in project.......................... 4

Figure 2 The halogens first electron affinity, first ionization energy and bond dissociation energy of halogen containing diatomic molecule 11

Figure 3 Number of structures containing a M–F bond 18

Figure 4 Highly bridging fluorido ligands in discrete transition metal polynuclear systems..... 20

Figure 5 Solid state structures of MoX_5 (X=Cl, F) 21

Figure 6 Geometries of selected bridge types between transition metal and Ln(III) centres..... 22

Figure 7 Bond formation by fluoride to metal σ- and π-donation.. 23

Figure 8 Molecular orbital splitting diagram for d^2-system 24

Figure 9 Mechanism for superexchange interaction 36

Figure 10 Variation of overlap with respect to bond angle 37

Figure 11 Crystal structure of Mn_{12}-analogue.. 44

Figure 12 Imine synthesis by nucleophilic addition of a primary amine to a carbonyl.............. 45

Figure 13 Enantioselective epoxidation of alkenes catalysed by $[Mn^{III}(X)(salen)]$ 47

Figure 14 Tetragonal Jahn-Teller distortion of $[MnF_6]^{3-}$ octahedra.. 50

Figure 15 Energy level diagram for Mn(III) in weak Octahedral and Tetragonal ligand field.. 53

Figure 16 Zeeman splitting of Mn(III) 54

Figure 17 Synthesis strategies of [Mn(X)(salen)] 56

Figure 18 Electrospray mass spectrometry (ESP) of MnF_3 in MeOH 64

Figure 19 Summary of different types of Schiff base complexes and their interrelation 67

Figure 20 Molecular structure of *catena*-[Mn(μ-F)(salen-5H)] 72

Figure 21 Molecular structure of *catena*-[Mn(μ-F)(salen-5F)] 72

Figure 22 Powder diffraction pattern *catena*-[Mn(μ-F)(salen-5H)] 73

Figure 23 Unit cell for *catena*-[Mn(μ-F)(salen-5H)] 74

Figure 24 Low temperature EPR spectrum of [Mn(F)(salen-5H)] and $[Mn(F)_2(salen\text{-}5H)]^-$ in nmf 77

Figure 25 EPR splitting diagram for [Mn(F)(salen)] and *trans*-$[Mn(F)_2(salen)]^-$ 78

Figure 26 Temperature dependences of molar Magnetic Susceptibility of *catena*-[Mn(F)(salen-5H)] 80

Figure 27 Model for description of magnetic behaviour of *catena*-[Mn(μ-F)(salen-5H)] 81

Figure 28 Modelling of the magnetic properties of *catena*-[Mn(μ-F)(salen-5H)] 82

Figure 29 Molecular structure of $[MnF_2(Me_3tacn)(\mu\text{-}F)MnF_2(Me_3tacn)]PF_6$ 84

Figure 30 χT product and modelling thereof for $[MnF_2(Me_3tacn)(\mu\text{-}F)MnF_2(Me_3tacn)]PF_6$ 85

Figure 31 Representation of 1. and 2. coordination sphere around *trans*-$[Cr(py)_4F_2]^+$ 101

Figure 32 Molecular structure of *trans*-*catena*-poly$[Na(H_2O)_4(\mu\text{-}F)Cr(py)_4(\mu\text{-}F)](HCO_3)_2$ 105

Figure 33 Unit cell of *trans*-*catena*-poly$[Na(H_2O)_4(\mu\text{-}F)Cr(py)_4(\mu\text{-}F)](HCO_3)_2$ 108

Figure 34 Hydrogen bonding in *trans*-*catena*-poly$[Na(H_2O)_4(\mu\text{-}F)Cr(py)_4(\mu\text{-}F)](HCO_3)_2$ 109

Figure 35 Molecular structure of *trans*-$[Cr(py)_4F(\mu\text{-}F)\{Li(H_2O)_{n=3,4}\}_{m=0,1}]Cl_5{\cdot}6H_2O$ 110

Figure 36 Hydrogen bonding and packing in *trans*-$[Cr(py)_4F(\mu\text{-}F)\{Li(H_2O)_{n=3,4}\}_{m=0,1}]Cl_5{\cdot}6H_2O$ 1112

Figure 37 Hydrogen bonding in dimeric units of *trans*-$[Cr(py)_4F(\mu\text{-}F)\{Li(H_2O)_{n=3,4}\}_{m=0,1}]Cl_5{\cdot}6H_2O$ 113

Figure 38 Molecular structure of *cis*-$[Cr(phen)_2F_2]ClO_4{\cdot}H_2O$ 130

Figure 39 Molecular structure of *cis*-$[Cr(bpy)_2(H_2O)(F)](ClO_4)_2{\cdot}2H_2O$ 131

Figure 40 Crystal packing of *cis*-$[Cr(phen)_2F_2]ClO_4{\cdot}H_2O$ 133

Figure 41 Crystal packing of *cis*-$[Cr(bpy)_2(H_2O)(F)](ClO_4)_2{\cdot}2H_2O$ 133

Figure 42 Hydrogen bonding in *cis*-$[Cr(phen)_2F_2]ClO_4{\cdot}H_2O$ 135

Figure 43 Hydrogen bonding in *cis*-$[Cr(bpy)_2(H_2O)(F)](ClO_4)_2{\cdot}2H_2O$ 137

Figure 44 X-ray powder pattern of *cis*-$[Cr(L'')_2(H_2O)(F)](ClO_4)_2$ for L''=phen, bpy 140

Figure 45 X-ray powder diffraction of thermolysis products 144

Figure 46 Molecular structure of the dinuclear complex in $[(phen)_2Cr(\mu\text{-}MeO)_2Nd(NO_3)_4]{\cdot}2MeOH$. 154

Figure 47 Unit cell contents of $[(phen)_2Cr(\mu_2\text{-}MeO)_2Nd(NO_3)_4]{\cdot}2MeOH$ 155

Figure 48 Magnetization of Cr and Dy in $[(phen)_2Cr(\mu\text{-}MeO)_2Dy(NO_3)_4]$ 157

Figure 49 Titration of *cis*-$[Cr(phen)_2F_2]^+$ with Gd(III) 162

Figure 50 Molecular structure of *cyclo*-$[(\mu\text{-}F)(phen)_2Cr(\mu\text{-}F)Gd(NO_3)_4]$ 163

Figure 51 Powder X-ray diffractograms of the isomorphous series of *cyclo*-$[(\mu\text{-}F)(phen)_2Cr(\mu\text{-}F)Ln(NO_3)_4]$ for Ln=Ce to Dy 164

Figure 52 Phoenix, The Aberdeen Bestiary ca. 1200 170

Figure 53 Part 1: Preparation of the sample for SQUID measurement. 188

Figure 54 Part 2: Preparation of the sample for SQUID measurement. 188

LIST OF TABLES

Table 1 AOM parameters and ligand field factorization for Cr(III) complexes 23

Table 2 Bonding parameters for Fluoridomanganate(III) complexes 50

Table 3 Geometric parameters for complexes of type [Mn(X)(SB)].. 68

Table 4 Selected geometric parameters for coordination polyhedra in
catena-[Mn(μ-F)(salen-5H)]... 75

Table 5 Selected geometric parameters for coordination polyhedra in
catena-[Mn(μ-F)(salen-5F)] 75

Table 6 Selected geometric parameters for coordination polyhedra in
catena-[Mn(μ-F)(salen-Cl)].. 75

Table 7 Spin-Hamiltonian parameters for [Mn(F)(salen-5H)] and *trans*-$[Mn(F)_2(salen\text{-}5H)]^-$.. 79

Table 8 Bonner-Fisher fitting parameters for Mn(III) chain systems.. 83

Table 9 $[Na(15\text{-crown-}5]^+$ and $[K(18\text{-crown-}6]^+$complexes of fluorido complexes. 99

Table 10 Selected geometric parameters for
trans-catena-poly$[Na(H_2O)_4(\mu\text{-}F)Cr(py)_4(\mu\text{-}F)](HCO_3)_2$ 106

Table 11 Hydrogen bond geometry for
trans-catena-poly$[Na(H_2O)_4(\mu\text{-}F)Cr(py)_4(\mu\text{-}F)](HCO_3)_2$ 109

Table 12 Selected geometric parameters for
trans-$[Cr(py)_4F(\mu\text{-}F)\{Li(H_2O)_{n=3,4}\}_{m=0,1}]Cl_5{\cdot}6H_2O$ 111

Table 13 Hydrogen bond geometry for *trans*-[Cr(py)$_4$F(μ-F){Li(H$_2$O)$_{n=3,4}$}$_{m=0,1}$]Cl$_5$·6H$_2$O 113

Table 14 Synthesis of mixed fluoride complexes by anation of [Cr(en)$_2$(H$_2$O)(F)]$^{2+}$ 125

Table 15 Selection of structurally characterized *cis*-[M(bpy)$_2$X$_2$]Y$_n$ complexes 128

Table 16 Structural characterized fluorido complexes of Cr(III) 129

Table 17 Selected geometric parameters for *cis*-[Cr(phen)$_2$F$_2$]ClO$_4$·H$_2$O 132

Table 18 Selected geometric parameters for *cis*-[Cr(bpy)$_2$(H$_2$O)(F)](ClO$_4$)$_2$·2H$_2$O................ 132

Table 19 Classification of hydrogen bonds according to properties Ref. [388] 135

Table 20 Strong hydrogen-bond geometry for *cis*-[Cr(phen)$_2$F$_2$]ClO$_4$·H$_2$O 136

Table 21 Weak hydrogen-bond geometry for *cis*-[Cr(phen)$_2$F$_2$]ClO$_4$·H$_2$O 136

Table 22 Selected bond lengths for [(phen)$_2$Cr(μ-MeO)$_2$Ln(NO$_3$)$_4$]·2MeOH.......................... 154

Table 23 Selected bond lengths and angles for *cyclo*-[(μ-F)(phen)$_2$Cr(μ-F)Ln(NO$_3$)$_4$] 163

Table 24 Conversion between units in different unit system.. 175

Table 25 Acronyms. Including experimental and calculation techniques 175

Table 26 General symbols used in reaction equations and for chemical entities 176

Table 27 Abbreviations for functional groups.. 176

Table 28 Abbreviations for solvents.. 176

Table 29 Abbreviations for ligands and other entities.. 177

Table 30 Symbols used in the thesis.. 181

Table 31 Complexes characterized by single crystal X-ray diffraction................................... 191

Table 32 Collection and refinement for single crystal diffraction for complex 1-9 193

Table 33 Colour codes used in graphical representation of the crystal structures.................... 194

Table 34 X-ray data No. 1: *catena*-[Mn(μ-F)(salen-5H)] 195

Table 35 X-ray data No. 2: *catena*-[Mn(μ-F)(salen-5F)] 196

Table 36 X-ray data No. 3: *catena*- [Mn(μ-F)(salen-5Cl)]... 197

Table 37 X-ray data No. 4: *trans*-*catena*-poly[Na(H$_2$O)$_4$(μ-F)Cr(py)$_4$(μ-F)](HCO$_3$)$_2$ 198

Table 38 X-ray data No. 5: *trans*-[Cr(py)$_4$F(μ-F){Li(H$_2$O)$_{n=3,4}$}$_{m=0,1}$]Cl$_5$·6H$_2$O 199

Table 39 X-ray data No. 6: *cis*-[Cr(phen)$_2$F$_2$]ClO$_4$·H$_2$O.. 200

Table 40 X-ray data No. 7: *cis*-[Cr(bpy)$_2$(F)(H_2O)](ClO_4)$_2$·2H_2O 201

Table 41 X-ray data No. 8: *cyclo*-[(NO_3)$_4$Nd(μ-F)Cr(phen)$_2$(μ-F)]$_2$ 202

Table 42 X-ray data No. 9: [(NO_3)$_4$Nd(μ-MeO)$_2$Cr(phen)$_2$] 203

Table 43 Collection parameters for Powder X-ray diffraction 204

PART I: THESIS

CHAPTER 1

INTRODUCTION

AIMS OF WORK

The aim of this work, of which parts are presented in this thesis, was from the outset focused on coordination polymers and metal-organic frameworks and their properties. [1-3] A major goal was the development of new types of extended coordination compounds with potential interesting magnetic properties rather than doing variations over known structure types. Fundamentally, coordination polymers as well as coordination cluster compounds are defined structurally, by the properties of the metal ions and the bridging ligands entering the structure.

Since fluorido complexes has played an important role in the contributions from the Inorganic Laboratory at University of Copenhagen to the development of ligand field theory, [4, 5] and general synthetic methods, [6, 7] it was decided to address the possible use of fluoride as a bridging ligand in the synthesis of clusters and coordination polymers. Apart from being an area where one could build on previously developed expertise concerning monomeric complexes, this direction of research had the attractive feature of being largely unexplored. Although Timco and co-workers, [8-10] have synthesized some very attactive wheel-shaped structures, which also include fluoride bridging, no systematic studies of fluoride bridging as a structural design motif have been conducted.

Due to the hardness of fluoride as a ligand in the Pearson classification, [11-13] it would be expected that fluoride would be best suited as a bridging ligand in systems with correspondingly "hard" metal ions. Accordingly, the regions of the periodic table of primary relevance would be the early transition metals, but also main-group elements from group I and II as well as Al(III), Ga(III), Si(IV)/Ge(IV)/Sn(II);Sn(IV) and P(V)/As(V)/Sb(V) could be envisaged as centers in extended fluoride-bridged structures. From the point of view of magnetism, it would be

especially interesting to pursue fluoride bridges between the hard lanthanoid ions and transition metals. Less than a handfull of such systems have been described to date and none of these involve paramagnetic transition metal centers. [14, 15] Thus one of the goals, which were pursued and achived in the work described here was the synthesis of the first 3*d*-F-4*f* systems with both paramagnetic transition metal and lanthanoid ions. After our synthesis and dissemination at meetings, the Winpenny group published two wheel-structures, which feature fluoride bridges between lanthanoids and Cr(III). However, in these systems, the fluoride bridges are supported by carboxylate bridges and neither the structural role nor magnetic exchange properties of the bridging fluoride can be determined. [16]

In order to understand and analyze the properties of complicated fluoride bridged systems, it is a prerequisite, that their mono-nuclear building blocks are well understood and characterized. Therefore, this thesis also encompasses work on electronic and spectral characterization of simpler systems including mono-nuclear 3*d* systems, chains of 3*d*-centers and bridged systems, wherein one of the metal centers is a spectroscopically innocent closed shell ion. The latter situation leads to a simplified description of the perturbing effects on a terminal fluorido ligand by its engagement in bridging.

The various systems, which have been studied can be viewed as a series of systems with gradually increasing complexity (*cf.* Figure 1). All are based on the 3*d*-F unit where either Cr(III) or Mn(III) coordinates minimum one fluorido ligand. The 3*d*-F unit has then been studied in proper mononuclear systems, but also in polynuclear or polymeric systems connected through bridging by fluorido ligand(s). This bridging is both of homo- and, for chromium also, hetero-metallic character. For the latter type of systems, completely novel classes of compounds containing either alkali metals or lanthanides have been synthesized and structurally and magnetically characterized.

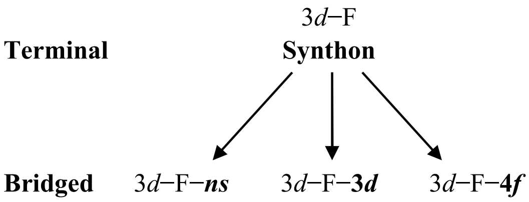

Figure 1 Types of terminal and bridged fluorido complexes studied in project

Synthetic strategy for new terminal and bridged fluorido complexes

As mentioned above and discussed in more detail in Chapter 2, fluoride is classified as a hard ligand, which favours interaction with hard metal ions. A synthesis strategy for extended structures based on direct molecular self-assembly reaction between the fluoride ligand and desired hard metal ion centres may in general be considered as problematic. On qualitative basis, the thermodynamically favoured product of direct reaction is likely to be a binary fluoride or hydroxide/oxide due to the basicity of fluoride.

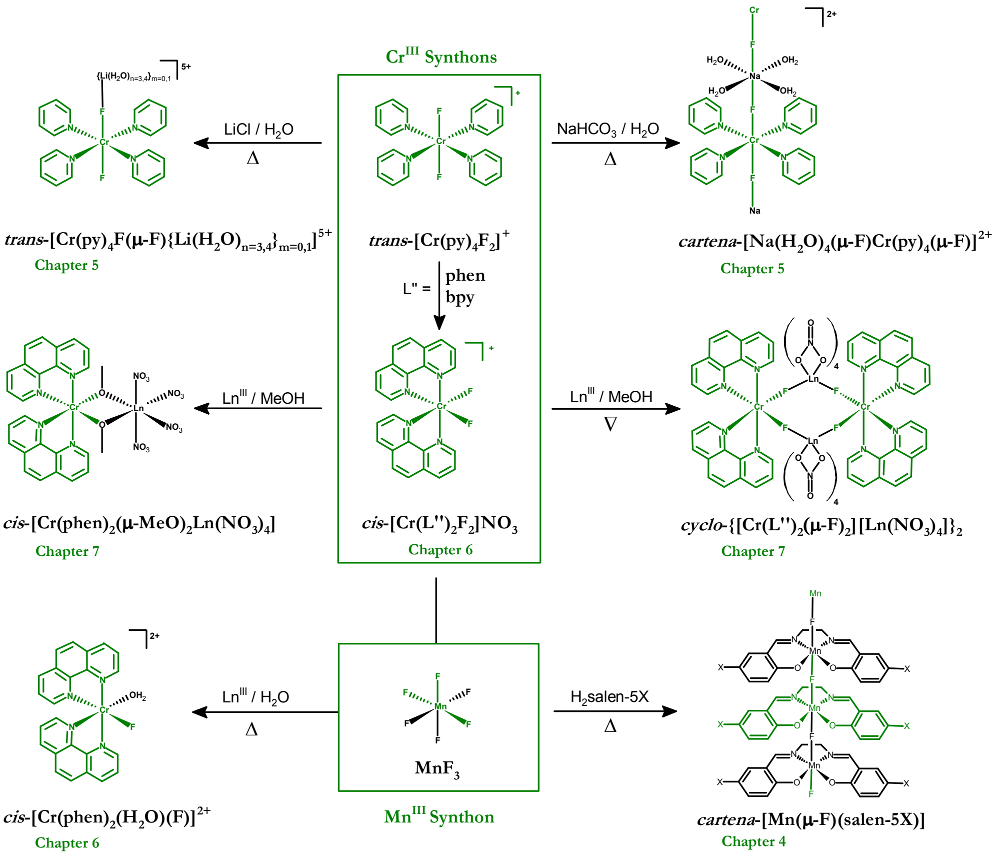

Scheme 1 Overview of Cr(III) and Mn(III) fluorido complexes synthesized by the synthon approach

The synthon parts of the complexes are depicted in green. Under each type of complexes are given in which chapter of the thesis, a detailed description can be found. The phen-synthon, *cis*-$[Cr(phen)_2F_2]^+$ is used as an example for illustration, but everywhere it can be substituted with bpy.

This fact must especially be taken into consideration if lanthanoid ions are involved, due to the very low solubility of LnF_3. As a solution to this problem a synthon based approach is used. The term is defined by Corey [17] as:

> *"...structural units within a molecule which are related to possible synthetic operations..."*.

The concept is commonly used in organic chemistry where it is intimately associated with the disconnection approach used for design of new synthesis strategies.
The entities that meet these requirements are considered as synthons for the (bridging) fluorido ligand. The synthons incorporated are all based on simple fluorido complexes of transition metals such as Mn(III) and Cr(III) focusing specifically on the latter, which is kinetically robust.
A graphical overview of the system investigated during the project is given in Figure 1 and Scheme 1. In these figures colour labelling is used to illustrate the synthon way of thinking.

STRUCTURE AND CONTENT OF THESIS

The thesis consists of two parts. “Part I Thesis” being a description of the selected results achieved during the project, which pertains to fluoride complexes and coordination polymers, while “Part II Publications” consists of a complete collection of the scientific papers that have been published during the thesis work.

The accompanying papers and the thesis do not form a one to one correspondence. It is thus unavoidable that some material in this thesis is not found in the articles and vice versa.

“**Part I: Thesis**” consists of the following seven chapters:

- **Chapter 1: Introduction**
  This, present, chapter gives a short overview presentation of the project. The aim of the project, its objectives as well as the synthon approach as synthetic strategy is introduced. The contents of this chapter will be the benchmark for the entire thesis. The overview given in Scheme 1 collects in a single figure all the threads of the dissertation. The reader is encouraged to return to this figure if needed.

- **Chapter 2: Fluoride as ligand in coordination complexes**
  This introductory chapter provides a general review of the chemistry of fluorine and its compounds. Special emphasis is given on fluoride as a terminal and bridging ligand in coordination complexes. The necessary concepts for understanding the distinct and often unique properties of fluorine containing systems are presented. This includes topics such as the HSAB-principle, bonding and electronic structure.

- **Chapter 3: Magnetism and thermodynamics**
  This introductory chapter provides a summary of the prerequisites for understanding the model framework used to describe the magnetic properties of the systems presented in the following chapters and associated papers. Concepts of magnetic susceptibility,

exchange couplings, Spin-Hamiltonians and models for magnetic chains are briefly introduced and discussed.

- **Chapter 4: Mn(III) fluorido complexes – chains and discrete systems**
  This chapter provides an introduction to the general chemistry of Mn(III) and the coordination chemistry of $[Mn^{III}(salen)]^+$ as well as the main applications and research areas, these systems takes part in (*e.g.* catalysis and magnetism). Synthesis and structural characterization of *catena*-[Mn(μ-F)(salen-5H)] and the derivatives *catena*-[Mn(μ-F)(salen-5R)] for R=F, Cl, "Br" are presented. *In situ* formation of the dianion *trans*-$[Mn(F)_2(salen)]^-$ is shown when fluoride in excess is added to *catena*-[Mn(μ-F)(salen-5H)]. On basis of characterization of these two fluorido complexe, it is possible to resolve the superhyperfine interction in the hexafluoridomagnanate(III) ion, $[MnF_6]^{3-}$. Associated with this chapter are Papers 1 and 5.

- **Chapter 5: Alkali metal cation complexation by Cr(III) fluorido complexes**
  This chapter investigates alkali metal ions interaction with robust Cr(III) fluoride synthons, in solution showing that this largely proceeds through second sphere coordination. This interaction may, by suitable choice of first coordination sphere synthon *e.g. trans*-$[Cr(py)_4F_2]^+$, allow for isolation of *s*-block complexes as exemplified by the infinite coordination polymer *trans*-*catena*-poly$[Na(H_2O)_4(\mu\text{-}F)Cr(py)_4(\mu\text{-}F)](HCO_3)_2$ and the discrete *trans*-$[Cr(py)_4F(\mu\text{-}F)\{Li(H_2O)_{n=3,4m=0,1}]Cl_5 \cdot 6H_2O$. Associated with this chapter is Paper 4.

- **Chapter 6: Fluoride lability: Aquation of *cis*-di-fluorido Cr(III) complexes**
  This chapter describes synthesis and structural characterization of the aquation products *cis*-$[Cr(L'')_2(H_2O)(F)]^{2+}$ for L''=phen, bpy produced (partial) solvolysis of the paternal difluorido complex by a new mild method using Ln(III) in MeCN. In addition, this chapter provides an introduction to difluorido complexes and their reactivity in aquations. Associated with this chapter are Papers 2 and 3.

- **Chapter 7: Methoxido-bridged Ln(III)-Cr(III) systems obtained by fluoride abstraction**
  In this chapter reaction of the same Cr(III) fluorido complexes studied in chapter 6, *trans*-$[Cr(py)_4F_2]^+$ with Ln(III) ions in methanolic solution is re-examined by use of non-harsh reaction conditions (*e.g.* ambient temperature). This reaction yielded heterobimetallic 1:1 complexes of the general type $[(phen)_2Cr(\mu\text{-}MeO)_2Ln(NO_3)_4]$ for Ln=Nd, Sm, Gd, Tb, Dy. The complexes are characterized by single crystal diffraction and the magnetic properties are examined. Associated with this chapter is Paper 8.

- **Chapter 8: Fluoride bridging as a structure-directing motif in 3*d*-4*f* complexes**
  This chapter describes synthesis and characterization of the isostructural series of tetranuclear complexes with the general formula *cyclo*-[(μ-F)Cr(L")$_2$Ln(μ-F)(NO$_3$)$_4$]$_2$ for L"=phen, bpy and Ln=Ce, Pr, Nd, Sm, Eu, Gd, Tb, Dy. The aniferromagnetic exchange interactions between Cr–Gd and Gd–Gd respectively, could be analyzed and quantified for the *cyclo*-[(μ-F)Cr(phen)$_2$Gd(μ-F)(NO$_3$)$_4$]$_2$ complex and for related systems of the by the group. Associated with this chapter are Papers 6 and 7.

- **Chapter 9: Conclusion and Outlook**
  This chapter summarizes and concludes on the synthetic results and progress achieved. Perspectives to the scientific results obtained to were the thesis end and what opportunities there are for using the results in further research are given.

- **Appendix:**
  This part of the thesis includes a collection of three appendices. These encompass a lists of abbreviations frequently used in the thesis, an Instrumentarium in which the apparatus and methods used in the characterization and analysis of the various compounds are described. Not included in this Instrumentarium, are the X-ray crystallographic techniques (single crystal- and powder diffraction) used in the characterization. These are described in a separate appendix, which also contain tables of the crystallographic data for all the compounds characterized by single crystal diffraction.
  Minor formalities in the main text, which are repeated several times and are without much importance *e.g.* general comment in connection with crystal structures, have been transferred to the appendix section.

"**Part II: Publications**" consists of ten papers all included in their full length, including supporting material to the extent considered necessary. Papers 1 to 8 are the subject of "Part I: Thesis" whereas the Papers 9 and 10 are not treated further. These last two papers can be read independently of the thesis and of the other articles and both are of such a closed subject area that they should not need further elaboration to be comprehensible to the reader of the thesis.

CHAPTER 2

FLUORIDE AS LIGAND IN COORDINATION COMPLEXES

INTRODUCTION

Fluorine is unique!

Fluorine stands out from the rest of the elements of The Periodic Table, both as the element and in the compounds, it forms no matter what branch of chemistry they are categorized to belong to. In comparison with the rest of the elements fluorine and fluorides exhibit very pronounced differences in reactivities, physical properties and structures, but not always in a rationalizable manner. This makes fluorine compounds highly appreciated but also feared for their sometimes unpredictable properties.
This chapter provides a brief introduction to the chemistry as well as the physical properties of fluorine and its compounds. All this is presented with fluoride as a ligand in coordination complexes in mind. One of the main purposes of this chapter is, through a simple conceptual framework, to provide an explanation of the properties that make fluorine and its compounds unique, and how the sometimes odd behaviour of such systems should be appreciated and turned into advantage rather than scorned.

Among all elements Fluorine is in many ways unique. Both with respect to chemical reativity and physical properties fluorine outdistance the related halogens as well as the rest of the elements. While interest in fluorine chemistry is growing, it is still much less investigated in coordination chemistry than the other halogens. This may be due to the differences in reactivities that make fluorine unique compared to other elements. A gift and misfortune in that conventional methods known and used for other systems does not necessarily work.

Fluoroorganic compounds is widely studied as polytetrafluroethylene (Teflon) and chlorofluorocarbons (Freon) and has had several uses as a result of their high thermodynamic stability and other physical properties.
Studies of transition metal fluoride complexes in terms of structural and magnetic properties are still a discipline with many white areas on the map. This should be contrasted with the overall development in areas such as materials science, catalysis and supramolecular chemistry where rational design of desired topologies is essential. The complex systems considered within these areas are often assemblies based on smaller units arranged in respect to each other by use of different bridging ligands - a task which the fluorido ligand might be expected to contribute with new properties.
Basic research in fluorine and its compounds are published in journals as *"Journal of Fluorine Chemistry"*. Actual review articles or monographs on fluorine chemical properties and reactivity with particular focus on coordination chemical systems are few in number *e.g.* "*Comprehensive Inorganic Chemistry*". [18] More generally the chemistry of fluorine and other halogens is treated in advanced textbooks. [19, 20] Of the few monographs is the classic and frequently quoted, but somewhat outdated "*The chemistry of fluorine and its compounds"* by Emeléus worth mentioning. [21]

CHEMICAL PROPERTIES AND REACTIVITY

Substantial parts of the physical and chemical properties of fluorine and fluorine-containing compounds can qualitatively be rationalized on basis of a very few concepts based on the atomic properties of the element. Although the simplicity of this description, and that it can be extracted from advanced textbooks in inorganic chemistry it is nevertheless a strong model that greatly gives an intuitive picture of the properties element and in particular compounds.

Fluorine, with ground state electron configuration $[He]2s^22p^5$ is the last 1. row element before the noble gasses and the first element in the 17. group. The group of halogens, exhibit a high degree of homology with respect to both physical and chemical properties. Nevertheless fluorine departs from this homology in several respects. This can be illustrated by depicting the variation in electron affinity, $E_{ea,n}$ and ionization energies, $E_{i,n}$ for the individual elements and the bond strength in relation to other elements, D(A–X) as shown in Figure 2. The first group of properties is atomic in nature and refers to the atom as well as the formation of ions, while the latter is a molecule-based property. Among the halogens, both the electron affinity and the first ionization energy follow an approximate linear variation down through the group with fluorine constituting an exception. That very distinct deviation initiated the discussion of whether such simple sets of observations could explain fluorines overall "anomalous" behaviour. [22]

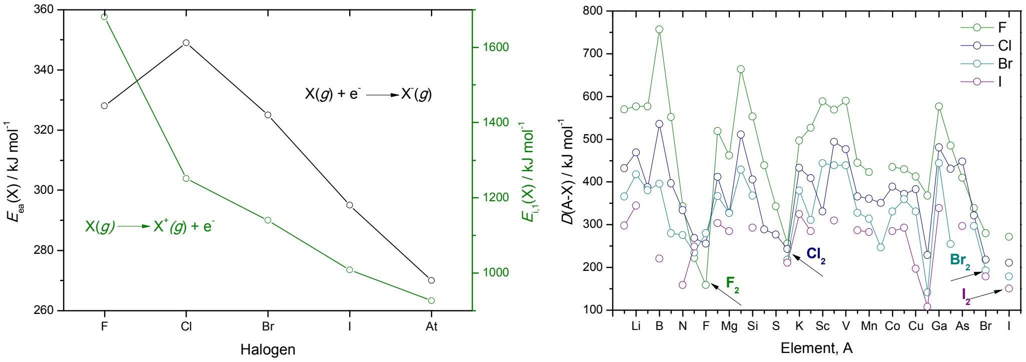

Figure 2 The halogens first electron affinity, first ionization energy and bond dissociation energy of halogen containing diatomic molecule

The homo atomic X_2 molecules are specially marked with arrows. All species are in the gas phase at temperature 298 K. The plots are drawn on the basis of values given in [23].

Based on variations shown in Figure 2 these differences from the rest of the halogens regarding the chemical properties and reactivity of fluorine and its compounds can be summarized by following statements: [18, 21]

- Low homolytic bond dissociation energy of the F_2 molecule.
- High bond strength of bonds formed between fluorine and other elements.

Besides these statements following must be added to complete the description of the properties of fluorine:

- Relative small size of the F atom and F^- ion.
- Low polarizability

Generally, instead of these two underlying properties, a description of the F atom and F^- ion will usually be based on a classification of fluorine in terms of the high electronegativity and high chemical hardness. The above mentioned high bond strength of bonds formed between fluorine and other elements translates directly by the definition into an extreme value for the Pauling electronegativity of fluorine, while the high chemical hardness in the Pearson sense is closely interconnected to the size and polarizability.

Fluorine continues the increasing reactivity seen in the 1. row and peaks as the most reactive element. Fluorine reacts exothermically with all elements when the right conditions are given - except the first three noble gasses (This general statement, however, were modified by

Khriachtechv *et al.* in 2000, with the synthesis of the argon fluoride, HArF by photolysis of hydrogen fluoride in a solid argon matrix. [24]).
In accordance with the magnitude of the electron affinity and ionization energy fluorine always occurs in reduced form with other elements as the fluoride ion F^{-} or as the negatively polarized part in electron sharing covalent compounds. In both cases the fluorine atom obtain the energetic farvorable electron configuration $[He]2s^2 2p^6$.
This reactivity is due to two factors, on one side the low bond dissociation energy of F_2 with a facile formation of the free fluorine atoms, which is important for obtaining low reaction barriers. On the other hand, the strength of the bond formed to the other element, which gives rise to a thermodynamic driving force for the reaction. Variation of bonding strength of the diatomic A–X molecule, were A is another or identical general element shown in Figure 2, reveals that the A–F bond regardless of whether the other element is a non-metal, metalloid or metals almost invariably surpass A–X bonds with respect to bond dissociation energy. The conclusion must however be taken with some reservations as these values represent dissociation energies for di-atomic molecules. However, the trend is clear. The significance of the interplay between the various properties such as electron affinity and binding energy, can be viewed in light of the considerable stability of the general A–F bond which on the basis of fluoride "anomalous" low electron affinity relative to the rest of the halogen group, would not be expected.

The causes of the low dissociation energy of F_2 has been subject of some consideration in the literature as to whether this is a property primarily to be referred to fluorine as atom or fluorine in the (a) molecule. [18, 22, 25-27] The most frequent description refers to the latter, saying that electronic repulsion between the outer shells electrons in the molecule is a dominant reason for the low dissociation energy. [26] On the other hand Politzer emphasizes that the high charge density of fluorine (small size and high effective nuclear charge) leads to unfarvorabel inter-electronic repulsion between valence electrons and the added electron by bond formation both ionic and covalent. [22]

ELECTRONEGATIVITY AND HARDNESS

As previously described, the reactivity of fluorine depends on, among other things, the strength of the bond fluorine form to other elements. Nevertheless, apparent from Figure 2 the strength of these bonds greatly depends on what other element is involved. Thus can be seen within the metals in the first transition metal period that the A–F bond dissociation energy of Sc, Ti and V are significantly higher than corresponding of Ni, Cu and Zn. This variation in bond strengths is recovered in the fluorine reactivity of these elements *e.g.* the use of copper-nickel alloys such as Monel metal for containing F_2.

Two principal issues for the understanding the chemical properties of fluoride is its electronegativity and polarizability. The former property is an important concept in many areas of chemistry and was introduced by Pauling, and loosely described as:

> *"...the qualitative property that the chemist calls electronegativity, the power of an atom in a molecule to attract electrons to itself."* [28]

The originally electronegativity concept introduced by Pauling was formed on a loosely semi-empirical basis, based on the bond dissociation energies. Several alternative definitions have been proposed over the years which include those by Mulliken, [29] Allred and Rochow, and Sanderson. Common to these are that they generally gives scales of electronegativity being approximately proportional, despite having different theoretical fundaments. [30]
Based on density functional theory is it possible to establish an absolute quantitative definition of the electronegativity as well as the related property hardness. A thorough review is given in, [31-33]. The electronegativity, χ_A and hardness, η_A (alternatively the softness: $\sigma_A = \eta_A^{-1}$) of an entity, A is defined as the first and second derivative respectively of the total electronic energy, E_A with respect to the total number of electrons, N at constant nuclear potential. [34]

$$\chi_A \equiv -\left(\frac{\partial E_A}{\partial N}\right)_Z \approx \frac{1}{2}\left(E_{i,A}(N) + E_{ea,A}(N)\right) \tag{2.1}$$

$$\eta_A \equiv \frac{1}{2}\left(\frac{\partial^2 E_A}{\partial N^2}\right)_Z \approx \frac{1}{2}\left(E_{i,A}(N) - E_{ea,A}(N)\right) \tag{2.2}$$

In these definitions is assumed that the total electronic energy can be expressed as a continus function of the number of electrons, which makes the definition directly implementable in DFT, which can conveniently be formulated without constraints on the number of electrons to be integral.
The derivatives can be calculated through numerical differentiation using a finite central difference approximation. This gives a expression of the inherent electronegativity identical to the definition by Mulliken. [29] The advantage of the definition (2.1) is that it can be used independently of whether it is applied to an atomic or a molecular entity. The derivative (2.2) defines the chemical hardness as the derivative of the electronegativity of the entity again with respect to the total number of electrons. Electronegativity being a measure of the ability to retain/attract electrons, the hardness corresponds to entities' ability to resist change in charge, which in broad strokes are related to the polarizability of the entity. By comparison of the approximate expressions for electronegativity (2.1) and chemical hardness (2.2) with the values for electron affinities and ionization energies for the halogens (given in Figure 2) it is evident that fluorine tops the rest of the halogens with respect to both properties.

By applying Koopman's theorem the electron affinity and ionization energy can be associated with orbital energies of the lowest unoccopied (LUMO) and highest occupied (HOMO) molecular orbital, respectively. [35] This means that the two concepts of electronegativity and chemical hardness are related to entities which can be calculated by the electronic structure model of choice. [33]

From the definition (2.2) it follows that a hard species is associated with a large difference between the the electron affinity and ionization energy, which again corresponds to a large energy difference between HOMO and LUMO. In a simplified description, this difference in energy is related to the polarizability of the entity meaning that hard entities have low polarizability.

The concept of polarizability is essential to understand the properties of reacting species. Based on this Ahrland *et al.* [36] and Pearson [11-13] early on established qualitative descriptions, which in many respects are in accordance with the quantitative description of a species chemical hardness based on (2.2). In both descriptions, ions are divided into two classes referred to as class (a) and (b) by Ahrland or hard acids/bases and soft acids/bases by Pearson. In the following there will only be referred to the designations given by Pearson.

In general hard acids/bases are characterized by a small radius and low polarizability while the soft acids/bases in principle can be understood as non-hard which means large and highly polarizable. These simple rules make high oxidation states and *d*-orbital configurations inaccessible for π-bonding likely to lead to hard acidic species. Examples of hard acids are, thus, H^+, alkali metals (*e.g.* Li(I), Na(I)), alkali earth metals (*e.g.* Be(II), Mg(II)), lanthanoids (Ln(III)), actinoides (Th(IV), U(IV)), and among the *d*-block elements, in particular early high-valent d^0 systems; Ti(IV), Zr(IV), Hf(IV), V(V), Nb(V), Ta(V), but also to a lesser extent *e.g.* Mn(II), Cr(III), and Fe(III). Correspondingly, examples of hard bases are the smallest and least polarizable ligands F^-, H_2O, OH^- and O^{2-}.

These properties of Lewis acids and bases have led to the following celebrated rule, often referred to as the HSAB-principle which in many respects, serves as a guide for rationalizing and predicting reactivities and thermodynamic stabilities:

> *Interaction between species having similar hardness (or softness) are energetic favoured.*

A qualitative derivation of the HSAB principle has been given in [37, 38].

The validity of this principle can be justified by looking at the possible bond formation between species with similar or different hardness. Combination of elements (groups) with similar hardnesses results in bond formation with pronounced electrostatic (hard acid–hard base) or covalent (soft acid–soft base) character, while combination of entities with dissimilar hardness gives a energetically less favorable, intermediate bond character.

It is important to note that the HSAB-principle can only be used as rough guideline for rationalization of chemical behavior and reactivity. Despite the fact that many exceptions exists, as discussed *e.g.* by Jørgensen, [39] the HSAB principle is very useful within the area of coordination chemistry and rationalizes the main features of the aqueous chemistry of metal ions. Concerning reactivity, ligand substitution reactions can rationalized by use of the HSAB principle, giving rise to the following rule:

> *"Ligand substitution reactions in which a hard ligand is to be displaced from a soft acid will be assisted (catalysed) by addition of a hard acid coordinated to a softer base. The assisting acid cation must be present in a labile complex"* [13, 40]

As a concrete example, the conversion of Co(III)–Cl into Co(III)–F by Hg(II)-assisted ligand metathesis in liq. HF can be mentioned. In general, however, the metal-ion-assisted ligand substitution suffers from the problem, that it is difficult to gauge, in advance, the reactivity in the specific chemical system and the nature of the reaction products. The balance between the hardness of the outgoing ligand and the metal center to which it is bound before reaction on the one hand and the hardness of the metal ion or metal ion complex assisting the ligand substitution on the other hand is delicate, and can be balanced out by a number of conditions including temperature, hydrolysis and the solubility of the products. A classic example of such a balancing out of driving forces and kinetices is in the quantitative analysis of halogen containing complexes of robust metal centers, where treatment with Ag(I) in the cold leads to precipitation of ionically bound halogenide alone, while a similar hot treatment leads to precipitation of both ionically and complex bound halogenide.

CLASSES OF FLUORINE CONTAINING COMPOUNDS

As previously mentioned fluorine will always appears in reduced form in its compounds. The bond type vary froms: ionic (lattice), intermediate forms and covalent (dative). An important element in a qualitative description of the general bonding of fluorine in its compounds is the smal atomic and ionic radius. This characteristic practicing its influence in all the general types of bonding. In the ionic and intermediate polar bond situation giving rise to significant lattice contribution and in the the covalent situation allows an efficient bond formation due to close proximity of fluorine and its bonding partner. [41]
Which of these bond types are involved depends largely on what other element fluorine combines with. The product of direct reaction (fluorination) between fluorine and other elements will frequently yields product containing the other element in its highest accessible oxidation state as seen for the molecular species MF_6 (M=Mo or W) and MF_7 (Re). These products are covalent in nature with low melting and boiling points and generally volatile. On the other hand,

binary fluorides obtained at more gentle reaction conditions with oxidation state +3 or lower of central atom are ionic or primarily ionic in nature.
In the following sections a brief qualitative introduction will be given to the bonding of fluorine in its compounds.

Stabilization of fluoride containing lattices
Fluorine containing compounds with moderate oxidation state (≤ +3) of the central atom are ionic or partially ionic in nature. The synthesis of these compounds can be either by reduction of high valent fluorides or more commonly from aqueous media by direct reaction between metal ion and fluoride often driven by a quantitatively precipitation of the product. Variation on this theme includes neutralization of a metal oxide, hydroxide or carbonate with aqueous hydrofluoric acid.
For the hard metal ions from the *s*-block, formation of ionic compounds is the rule. Such ionic crystal lattices are characterized by regular three-dimensional lattice consisting of ions with alternating electric charge and in the case of fluorides, relatively high coordination numbers of the metal centers. The fluorides of 1. group LiF (Griceite), NaF (Villiaumite), KF (Carobbiite) all crystallize in NaCl-lattice. The heavy fluorides of 2. group CaF_2 (Fluorite), SrF_2 (Strontiofluorite) and BaF_2 (Frankdicksonite) all crystallize with the same fluorite lattice consisting of a cubic 8 coordination of fluoride around the 2. groupe metal ion and a tetrahedral 4 coordination the opposite way. The smaller Mg(II) ion makes MgF_2 (Sellaite) deviate from this by adopting a rutile structure (6:3 coordination).

It is not possible to present a unified explanatory model for fluoride stabilization of the higher oxidation states of the elements, simply because the higher/highest oxidation states cover a range in which both ionic and covalent interactions are important. As already mentioned above, for the very highest oxidation states (+6 and +7), fluorides become covalent, and discretely molecular compounds with their associated characteristics. In the case of ionic systems the stabilization of high oxidation states (in practice meaning +2, +3 and possibly, +4) can rationalized fairly straight-forwardly by thermodynamic considerations involving a Born-Haber cycle. [20, 22, 41]
As a consequence of the thermodynamic functions are state functions, a number of equal Born-Haber cycles can be established. Perhaps the simplest referes to the enthalpy of formation, $\Delta_f H(MF_n)$ of $MF_n(s)$ from the elements $M(s)$ and $F_2(g)$. This can be expressed by a sum of contributions from the metal, the halogen and the product respectively. Contribution refering to the metal are the enthalpy of sublimation, $\Delta_{sub}H(M)$ and the total ionization energy. Similarly, the halogen contributions are the bond dissociation energy, $D(X_2)$ and the electron affinity, $E_{ea,1}(X)$ and from the product is included the lattice energy of MX_n, $U(MX_n)$. For metals, the total ionisation energy is in general significantly higher than the enthalpy of sublimation. Also the difference between the bond dissociation energy and the electron affinity is approximately

constant. Applying this to the Born-Haber cycle it is found that the enthalpy of formation for MX_n roughly given as the difference between the total ionization energy required for the formation of the M^{n+} and the lattice energy gained by formation of the ionic lattice. Of these two, it is generally the lattice energy, that gives rise to the largest contribution. Until now, the considerations has been relatively independent of the bond type between fluorine and the other element. In the following it is used, that the lattice energy for an ionic lattice depends in a reciprocal manner on the internuclear distance between the ions of the lattice according to the Born-Landé equation. The small size of F^{-} therefore results in a significantly higher lattice energy compared to the rest of halogens and concommitant more efficient stabilization of higher metal oxidation states.
An example the diminshed tendency towards stabilization of high oxidation state down the group of halogens is given by the fluorides and iodides of osmium. These fall into two distinct groups of high-valent fluorides ($Os^{VII}F_7$, debated), $Os^{VI}F_6$, $Os^{V}F_5$, $Os^{IV}F_4$ and low-valent iodides $Os^{III}I_3$, $Os^{II}I_2$ and $Os^{I}I$. The thermodynamic explanation based on the assumption of ionic structure breaks partly down when looking at systems where covalent contributions are sufficiently large. This is seen for example in systems such as OsI_2 and HgI_2 where also π-bonding occurs.
Fluoride stabilization of high oxidation state is general and independent of the other element and its position in the periodic table. Frequently the highest oxidation state is achieved in binary fluorides, but in some cases, further stabilization through complex formation is necessary. As an example, directly fluorination of Ni(0) results in $Ni^{II}F_2$ as the only product. However, both Ni(III) and Ni(IV) can be reached in the complex anions, $[NiF_6]^{3-}$ and $[NiF_6]^{2-}$ by fluorination of a mixture of $NiCl_2$ and KCl at high pressure and temperature. The improved stability of the higher oxidation states of *e.g.* nickel in discrete complexes demonstrates, not unexpectedly, that terminally coordinated fluoride it a better donor than shared (bridging) fluoride.

Fluoride as terminal ligand

Numerous terminal fluorido complexes are known and this chemistry has been reviewed several times. It is not suited for detailed review here, but a few general remarks concerning the chemistry of fluorido complexes are in place.

Fluoride, as a strongly π-donating ligand ([5] and section below) exhibits a preference for the ealier *d*-block metals. As an example $[TiF_6]^{2-}$ is easily formed, while analogous fluorido complexes of the later transition metals are much rarer, and frequently difficult to prepare. This can be considered a chemical manifestation of the HSAB principle discussed above. The preference af fluoride for binding to the early transition metals can be quantified by the number of known structures of fluoride complexes of the individual transition elements. This has been done in Figure 3, which illustrates that point, but with copper standing out as an exception. This is not due to any peculiarity of copper, but rather to the fact that copper complexes are so

favored by coordination chemists that the copper containing structures outnumber those of the other elements by a large margin. Thus, for copper a total of more than 40.000 structures have been reported while less than 7000 and 8000 have been reported for Ti and Cr, respectively.

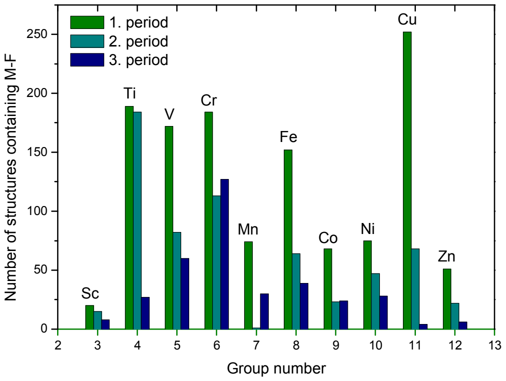

Figure 3 Number of structures containing a M–F bond

In general metal fluoride bonds are short (Cr(III)–F: 1.85-1.90 Å; Cr(III)–OH; 1.85-1.95 Å; Cr(III)–Cl: 2.30-2.35Å), which reflects the small size of the fluorido ligand as well as its propensity for π-bonding (see next section).

In a simple picture based on Lewis structures, the π-bonding ability of fluoride would be depicted by the resonance forms.

$$M - F \longleftrightarrow \overset{-}{M} = \overset{+}{F} \longleftrightarrow \overset{2-}{M} \equiv \overset{2+}{F} \qquad (2.3)$$

These are analogous to those normally invoked for explaining the π-donating properties of the isoelectronic nitrido and oxido ligands. Clearly, the multiply bonded structures are disfavored by the formal positive charges of fluorine, but the small size of fluoride and the ensuing good overlap with metal orbitals offsets this. The experimental evidence is clear, in that fluoride surpasses the other halide ions as π-donor, although it does not quite match hydroxide in this respect. [5, 42]

The importance of π-bonding in determining the short bond-lengths should, however, not be overestimated, as can be seen from the fact that Cr–F is larger (1.72 Å) in $M^{I}_2[CrF_6]$ than Ni–F (1.70 Å) in low-spin, d^6, $K_2[NiF_6]$. [43] This is the order expected from the decreasing ionic radius along the first transition row, which is not completely compensated by the d^6 configuration for the nickel compound, which obstructs π-donation from fluoride. A similar difference of ca. 0.02 Å is observed in Cr–F and Co–F bond lengths between the ions *trans*-$[M(py)_4F_2]^+$ (M=Cr, Co) with the Co–F bond length being the shortest. Again this shows that the π-bonding in the earlier transition metal complexes is not sufficient to offset the general size difference relative to the later systems caused by difference in effective nuclear charge.

A final general comment, to the structural chemistry of the terminal fluoride complexes, which naturally bridges to the next section, concerns their interaction with counter ions. It is very frequent that anionic terminal fluoride complexes are isolated as salts of counter ions, which are good at engaging in hydrogen bonding. Examples include ammonium, guanidinium, and

protonated polyamines. This illustrates that the ability of fluoride to act as a hydrogen bond acceptor has not been quenched by coordination to the metal centre. Another illustration of this point is the increasing number of bifluoride (M–FHF) complexes [44-51] being investigated in the recent years. Thus, coordinated fluoride retains some of the Brøndsted basicity of the free fluoride ion, but most importantly, it is also a Lewis base with the capability of interacting with other metal centers.

Fluoride as bridging ligand

Fluoride, like the rest of halogens has the ability to simultaneously bind to two (or more) different centers thereby allowing the formation of μ_2-, μ_3-, μ_4-, μ_5-, and μ_6-fluorido bridges in molecular systems. The highly bridging fluorido ligands, although well know from ionic lattices (NaF: μ_6-F, [52] CaF_2: μ_4-F, [53] and LnF_3: μ_3-/μ_4-F, [54]) are much more rare than highly bridging isoelectronic oxide or hydroxide. To illustrate this point: the number of structurally characterized μ_3-oxido systems is ~2500, for hydroxide ~1400 μ_3-bridged systems have been structurally characterized, while a mere 69 structures are reported with μ_3-fluoride bridges.[2]

As the bridging fluorido ligands are significantly poorer π-donors than terminal ones, the requirement for hard, early transitions metal centers becomes more relaxed for fluorido bridged systems than for terminal fluorido complexes. Thus, 4 of the 5 established systems with μ_5-, and μ_6-fluorido bridges features the soft, d^{10} configuration, Ag(I) as the metal center *e.g. catena*-[(μ_6-F)(μ_4-CN)(μ_4-$CF_3COO)_4(H_2O)_2Ag^I_6$] were F is centered in a octahedral arrangement of six Ag(I) ions. [55] Examples of discrete molecular clusters with highly bridging fluorido ligands are shown in Figure 4. The most common bridging motifs are, as for all other simple bridging ligands μ_2- and di(μ_2-). It might be expected that the small size of the fluoride ion as compared with the rest of the halides would result in the mono-μ_2-fluorido bridge motif (M–F–M′) being the most common. However, it turns out that μ_2-fluorido and di(μ_2-fluorido) bridged systems are comparable in number (ca. 110-120 characterized for each type).

[2] Structure searching in this and subsequent sections are made in "The Cambridge Structural Database" (CSD version 5.32, updated Nov. 2010). It should be noted that this database, as a criterion for inclusion of structures requires the presence of carbon in the compounds. True inorganic compounds without carbon are not included in the given considerations.

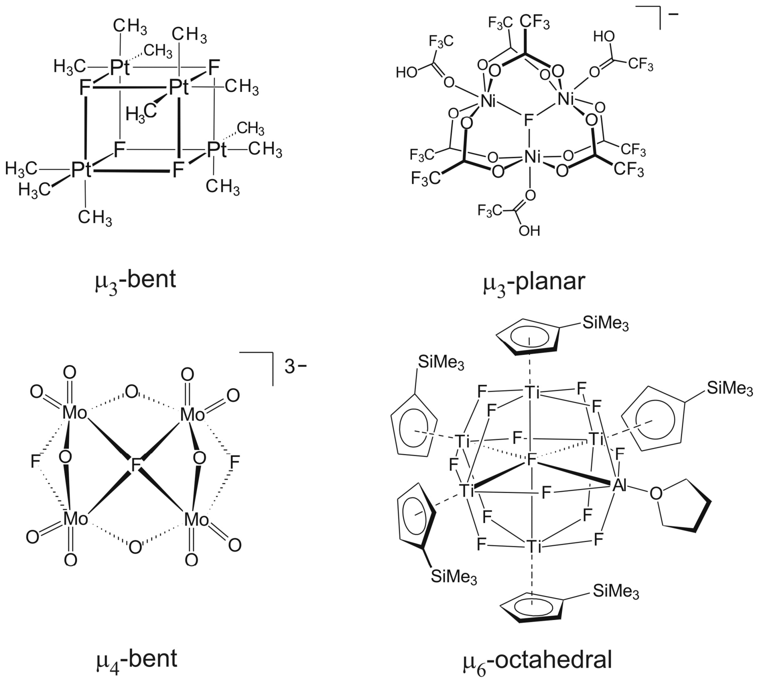

Figure 4 Highly bridging fluorido ligands in discrete transition metal polynuclear systems

Ref: μ_3-bent: [56], μ_3-planar: [57], μ_4-bent: [58], μ_6-octahedral: [59].

With increasing number of fluoride bridges, the two centers linked by the bridges are forced closer together, which leads to unfavorable repulsion between the metal centers as well as between the bridging ligands. Furthermore, increasing the number of bridges forces the bridging geometry towards more bent structures which is less common for donor atoms from the second row of the Periodic Table as compared to their heavier congeners. [19, 43, 51] This phenomenon is classically illustrated by the structures of the penta-halides of molybdenum (which are mirrored by the structures of penta-halides of several other transition metals). The structures of $MoCl_5$ and MoF_5 are shown in Figure 5, with characteristically bent chloride bridges and linear fluoride bridges.

All di(μ_2-fluorido) bridged systems are in general homo metallic and mainly derived from the early transition metals from Gruop 4. (particularly zirconium) as well as from vanadium. With exception of iron, palladium and in particular copper, the rest of the transition metals exhibit only few examples. Also the very large number of cubane clusters with μ_3-sulfide bridges as

compared to the much less frequent cubane structures with oxide-corner ligands indicates that the tendency towards forming bent bridges increases down any given group due to less *s-p* hybridization on the ligating atom. Despite the ensuing strained geometries, a few examples of the tri(μ_2-fluorido) bridges (M–(μ-F)$_3$–M′) in face sharing octahedral structures are known. Most prominently the $[M_2F_9]^{3-}$ ions (M=V, Cr, Fe), but also heteroleptic structures have been reported, *e.g.* $[\{Mo(H)_2(PMePh_2)_3\}_2(\mu\text{-}F)_3]^+$ and $[\{W(CO)_2(PMe_2Ph)_2\}_2(\mu\text{-}F)_3]^+$ with average M–F bond distances of 2.16(1) Å and 2.12(1) Å and bridges angles ∠M–F–M of 97.7(2) ° and 98.3(6) °. [60, 61] The bridging angles in the tri(μ_2-fluorido) bridged structures are fairly acute at ca. 90 ° and thus compressed very significantly from the commonly linear coordination. This, however, do not give rise to especially short M–M distances (3.256(2) Å and 3.216(3) Å). The $[M_2F_9]^{3-}$ ions (M=V, Cr, Fe) are particularly interesting in the present context since the magnetic properties these systems have been investigated relatively thoroughly (*vide infra*). [62, 63]

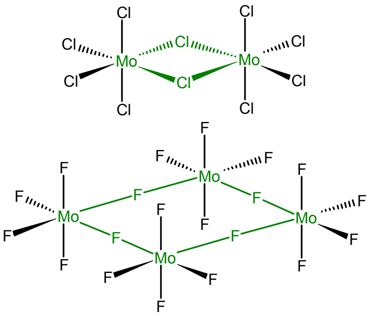

Figure 5 Solid state structures of MoX_5 (X=Cl, F)

These structures emphasize the different preference for bridging geometries for the two halides.

There are many examples where bridging ligands occur together with other supporting bridges. In such cases it is difficult or impossible to gauge if the resulting structural – or for that matter magnetic – properties can be referred primarily to one of the bridges. In order to evaluate the inherent properties of fluoride as a bridging ligand it is, therefore, most instructive to limit the discussion to cases where fluoride acts as an unsupported bridging ligand. From the *Cambridge Structural Database* all systems with unsupported, μ_2-bridging F^-, O^{2-}, OH^-, and Cl^- ligands have been extracted. Unsupported is here taken to denote situations where the two metal centers and the bridge are not members of rings with 10 or less atoms. For all these structures the distributions of the bridging angle and the average bond lengths in the bridges have been plotted in Figure 6. From these data a number of points can be made: Most importantly, fluoride resembles oxide very closely in the distribution of the bridging angles.

For both of these ligands there are a propensity for linear bridging demonstrated by the distribution of the angles peaking pronouncedly at 180 °. In this respect fluoride is quite different from both hydroxide and chloride where the angle distributions peak around 120 ° (average values are 130.9 ° and 129.2 ° for hydroxide and chloride bridges respectively). This preference for linear bridging by fluoride is important as it suggests that the use of fluoride bridges may serve as a structure directing motif allowing some control over the resulting topology of poly nuclear systems. Such control is not usual, but definitely desirable if any claims towards “cluster design” is to be made.

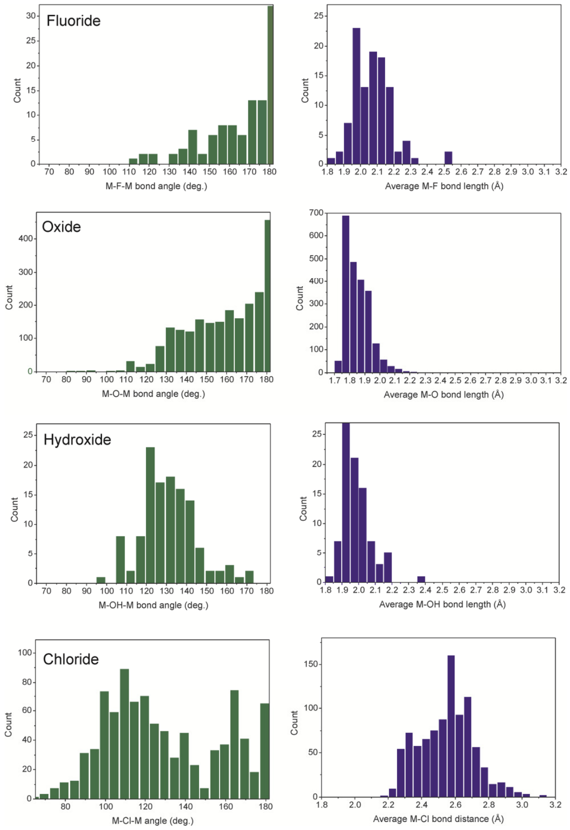

Figure 6 Geometries of selected bridge types between transition metal and Ln(III) centres

Only unsupported bridges are included. These were selected from the CSD database as systems with the pertinent bridging ligand, but without other supporting bridges yielding rings consisting of 10 or less atoms. Data are from CSD version 5.32, updated Nov. 2010.

The distributions of bond lengths shows the fluorido bridges to be significantly shorter than the chloride bridges (2.074 Å vs. 2.550 Å). In the context of magnetic properties it seems relevant to note that the more bent chloride bridges do not compensate for the longer metal-chloride bond lengths. On average metal-metal distances are longer in the chloride bridged systems at 4.60 Å than in fluoride bridged 4.12 Å. However, as it will be discussed below, metal-metal or metal-bridging ligand distances are of minor importance compared to bridging angles in determining the strength of magnetic interaction across the (fluorido) bridge. Finally, it should be noted that both hydroxide and, not surprisingly, oxide exhibit shorter bond lengths as bridging ligands. Slightly surprising is, though, the quite small difference between these two ligands in terms of bridging bond lengths.

ELECTRONIC PROPERTIES OF FLUORIDE AS LIGAND

As ligand, fluoride has the possibility of both σ- and π-donation towards a central metal ion. In Figure 7, such interactions are depicted. Both the σ- and π-interaction occurs by donation from a filled *p*-orbital (or *sp*-hybrid). In principle, all ligands possess the competing capability for back-donation into empty, ligand-based orbitals. However, in practice, empty ligand-based orbitals are far too high in energy for back-bonding to be of any importance in halide complexes in general and for fluoride complexes in particular.

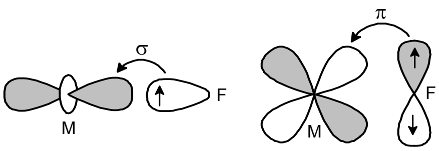

Figure 7 Bond formation by fluoride to metal σ- and π-donation

Fluoride is found to a much stronger π-donor compared to the other halogenide ions. This is due to a much better π-overlap between metal *d*-orbitals and the compact fluoride 2*p*-orbital as compared to the situation for the heavier halogenides were the *p*-orbitals are much more diffuse.

In classical, non-additive, ligand field theory, symmetry-based parametrizations of the ligand fields do not in general allow a separation of σ- and π-bonding effects. Since, the observable cubic ligand field splitting depends on a difference between σ- and π-bonding parameters ($\Delta = 3e_\sigma - 4e_\pi$) differences between ligands become leveled out if the variation between different ligands is in the same direction for σ- and π-parameters. This is the situation found for the halogenide ions as ligands as demonstrated by the data summarized in the Table 1. [64]

	f(ligand)	e_σ/cm^{-1}	e_π/cm^{-1}
F^-	0.9	7800	2000
Cl^-	0.8	5600	900
Br^-	0.76	5400	1000
I^-	0.63	4400	500
OH^-	0.94	8700	2100
OH_2	1.00	7500	1400
NH_3	1.25	7200	0

Table 1 AOM parameters and ligand field factorization for Cr(III) complexes

Column 2 contains the ligand factor in Jørgensen's factorization of the total cubic ligand field ($\Delta = f_{metal} \cdot g_{metal}$). [64], [65]

Column 3 and 4 lists approximate absolute AOM parameter values for Cr(III)

A number of observations are possible from these data: It is seen that the parallel variation of σ- and π-parameters leads to a much smaller relative difference in the cubic ligand field splitting among the halides than the relative difference in σ- and, particularly π-parameters, when these

are considered alone. It should also be noted that both fluoride and the isoelectronic hydroxide are better σ- as well as π-donors towards Cr(III) than the "strong field" ligands water and ammonia. Their low position in the spectrochemical series reflects their pronounced π-donor properties. This spectroscopic classification of fluoride and hydroxide, of course agrees well with their chemical ability to stabilize high oxidation states when functioning as ligands.

One disadvantage of using AOM parameter values extracted from d^3 systems such as Cr(III) is that the σ- and π-parameters have to be determined simultaneously and in conjunction with interelectronic repulsion parameters since the observable transitions depend parametrically on all three types of parameters. This may clutter values for the numerically smaller π-parameters with uncertainties from imperfections in the modeling of the σ-dependent part of level energies, because the σ- and π-parameters are statistically correlated in the modelling. The ideal situation for extracting ligand parameters would be to parametrize spectra of one-electron, two-atomic systems (M–L). These systems are, however, chemically irrelevant and only gas-phase spectroscopic information can be obtained. A chemically realizable compromise, which allows determination π-parameters practically uncorrelated to other ligand-field parameters are the so-called strongly tetragonally compressed d^2 systems. [5]
In these systems an axial field from a strong donor (*e.g.* O^{2-}) causes a splitting of the t_{2g} orbitals of octahedral origin, which is sufficiently large to cause pairing of the two electrons in a non-bonding d_{xy} orbital. The total *d*-orbital splitting pattern is depicted in Figure 8. The orbital splitting corresponds to that originally derived for the vanadyl ion (VO^{2+}) by Jørgensen and by Ballhausen and Gray. For fluoride complexes an anlogous orbital splitting diagram is expected, however, with much smaller splittings of the levels of octahedral parentage.

For d^2-systems the orbital splitting diagram of Figure 8 leads to diamagnetic complexes if the axial donor are strong enough. A large number of such systems are known. [66] These systems have the virtue, that the lowest-energy electronic transitions are between the filled d_{xy}-orbital and the π^* set of orbitals, $\{d_{yz}, d_{zx}\}$. If the axial ligand *trans* to the strong donor is varied, but the remaining ligand sphere kept constant, the variation in the energy of the first (spin allowed) electronic excitation directly measures the variation in the π-bonding parameter of the "*trans*-ligand".

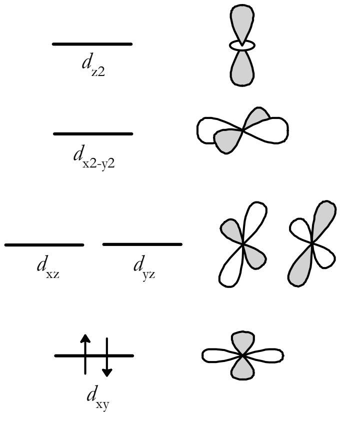

Figure 8 Molecular orbital splitting diagram for d^2-system

In the beginning of this chapter, the electronegativity of fluoride was discussed at some length. There is, though, one property of fluoride complexes related to its electronegativity, which we have not touched upon yet, namely the so-

called spectroscopic innocence of fluoride as a ligand.

It was early on recognized that complexes of the different halides differed markedly not only in the position of the ligand field transitions as discussed above, but also in the positions of the ligand to metal charge transfer (LMCT) transitions. Thus, while LMCT transitions occur to very high energies for fluoride complexes they occur at progressively lower energies for the heavier halogens. Use as example the position of the onset of CT bands in the halides of Ti(IV): $TiCl_4$: 35.600 cm^{-1}, $TiBr_4$: 29.500 cm^{-1} and TiI_4: 19.600 cm^{-1}. [67, 68]

Consequently, ligand field transitions in systems with *d* electrons are frequently, totally or partially obscured by much more intense charge transfer transition in complexes of bromide and iodide. One might say that fluoride is spectroscopically transparent or spectroscopically innocent (not to be confused with ligand redox innocence – but also not unrelated to this property) as compared to the other halides. This observation was quantified by Jørgensen, who introduced the concept of optical electronegativity. Put in quantitative terms the difference between the halides described above can be summarized in the approximate differences in their positions of the first charge transfer transition, ν(CT) according to: Cl: 28.000 cm^{-1}, Br: 34.000 cm^{-1}, I: 44.000 cm^{-1}. These differences are approximately proportional to the conventionally defined electronegativities of the halides. This led Jørgensen to assume that the charge transfer transition energies can be related to the electronegativity of the donor and acceptor species involved in the transition. By this assumption, it was found possible to arrive at a consistent set of "orbital" electronegativities, which parameterized most of the known data for LMCT transitions.

For our purpose this insight has two possibly important consequences. First, the spectroscopic innocence of fluoride as a ligand allows for the observation of ligand field transitions, which we employ in investigation of the second sphere interaction between coordinated fluoride and respectively solvent and metal ions in solution. Secondly, the high energies of charge transfer transitions in fluoride complexes could be feared to be a disadvantage in using fluoride as a bridging ligand in magnetic systems. The reason for this being that in the Anderson theory of magnetic exchange, important contributions to the exchange coupling between metal centers stem from interactions between the ground state and charge transfer states wherein the bridging ligand has acted as donor to one of the involved metal centers. These states are obviously much higher in energy for fluoride than for the other halides – or for hydroxide or oxide for that matter – and as a consequence the contrubitions to the exchange coupling from this mechanism will be smaller for fluoride. Fortunately, pre-empt the conclusion of the thesis and reassure the reader, that this does not render fluoride unsuited as mediator of magnetic exchange.

CHAPTER 3

MAGNETISM AND THERMODYNAMICS

INTRODUCTION

During the last decades, research in molecular magnetism has experienced a steeply increasing attention. Fuelled by the development of single molecule magnets, all aspects of molecular magnetism have benefited and grown. One of the main goals in the study of molecule-based magnetic systems has been the development of magnetic materials with specific and predictable properties *e.g.* magnetic ordering temperatures. [69-81]
An underlying and in some ways unifying theme for research in molecule based magnetism is the search for magneto-structural correlations relating the molecular structure and magnetic properties of polynuclear metal complexes. Important factors in such magneto-structural correlations include, but are not limited to: electronic configuration(s) of the metal centre(s), chemical nature of the bridging ligand(s), and geometry of the bridging arrangement. Naturally, these factors are not mutually independent, but they constitute probably the most natural independent parameters for the chemist trying to understand the properties of a related class of systems. In principle the goal of developing magneto-structural correlations is achievable even without control over the structures of the obtained systems. However, in order to seize the "holy grail" and achieve materials with designed magnetic properties, the simultaneous establishment of magneto-structural correlation and control over molecular structure is required. As will be demonstrated in the remaining of this thesis, employing fluoride as a bridging ligand has some positive benefits in terms of its ability to direct the structure of polynuclear systems, but, of importance to the idea behind this approach is also the ability of fluoride to mediate magnetic interactions between the metal centres it is bridging. This property, is studied and quantified primarily by measurements of magnetic susceptibilities of (large) polynuclear metal complexes

of interest. Frequently, these systems are of such complexity and the susceptibility data of such limited information content, that it is difficult to extract all of the desired parameters from such measurements. This calls for as simple (or rather simply bridged) systems as possible to obtain the maximal information on the local interactions.
In order to facilitate appreciation of the magnetism data obtained for the systems presented in this thesis, a very brief introduction to the most relevant concepts of magnetism will be given in this chapter. This will encompass:

- General behaviour of paramagnetic systems
- The concept of magnetic susceptibility and its connection to quantum mechanics
- The Spin-Hamiltonian formalism illustrated by an example
- The parameterization of magnetic exchange in the Spin-Hamiltonian formalism
- A brief mention of the Anderson theory for magnetic exchange
- Comments on some selected literature data

Generally, only the points of most relevance to the present work will be discussed. The numbers of textbooks and reviews offering more complete discussions and derivations are legio.

MODELS OF MAGNETIC BEHAVIOR

Classically, the overall variation of the magnetic susceptibility with respect to temperature for a paramagnetic compound has often been described by use of the Curie-Weiss law:

$$\chi(T) = \frac{C}{T-\theta} \tag{3.1}$$

The value and sign of the Weiss temperature, θ tells about the overall nature of the interaction between the system's magnetic centres. $\theta = 0$ is seen for a pure paramagnetic system with absence of magnetic interaction while $\theta \neq 0$ indicates interaction that depending on the sign can be divided an into ferromagnetic ($\theta > 0$) or antiferromagnetic interaction ($\theta < 0$). Despite the simplified description of the magnetic interaction given by the Curie-Weiss law, it is however a frequently used tool to describe the overall magnetic behaviour before more detailed modelling is used. Especially in older literature, an evaluation based on this approach will often be the only parametrization given of the magnetic data. The strength of the descriptions is, of course, the few parameters involved, but it is simultaneously its weakness, since little can be learned from the parameters except for the sign and magnitude of averaged interactions. Also the modelling offers no parameters transferability and hence little playground for the synthetic chemist.

Magnetization and magnetic susceptibility

Frequently, the magnetic susceptibility is introduced simply as the response of the energy to a change in "magnetic field strength" at the limit of low "magnetic field strengths". As an alternative, the concept can be approached from a more thermodynamic angle: Consider a closed system placed in a magnetic induction, $\overline{B}$ the differential magnetic work done on the system is given as:

$$\delta w = \vec{M} \cdot d\vec{B} \tag{3.2}$$

At constant temperature (as well as other external variables if present) the magnetization component, M_α is obtained as the derivative of the Helmholtz free energy, F with respect to the magnetic induction componemt, B_α.

$$dF = -SdT - M_\alpha dB_\alpha \Rightarrow M_\alpha = -\left(\frac{\partial F}{\partial B_\alpha}\right)_T \tag{3.3}$$

This classical thermodynamic expression is related to the statistical thermodynamics and thereby quantum mechanics through the partition function, $Z(T,B) \equiv Z$ defined as the sum of Boltzmann factors of every state in the system and a function of both temperature, T and magnetic induction. N_A and k_B are the Avogadro and Boltzmann constants respectively.

$$F = -N_\mathrm{A} k_\mathrm{B} T \ln Z \tag{3.4}$$

Were the partition function, Z is defined by the infinite sum over all states, $E_n(B)$:

$$Z \equiv \sum_{n=0}^{\infty} \exp\left[\frac{-E_n(B)}{k_\mathrm{B}T}\right] \tag{3.5}$$

Not only can the Helmholtz energy be determined from knowledge of the partition function but all thermodynamic state functions and derived quantities are accessible from this. The expression of Helmholtz free energy establishes a connection between the classical and the statistical thermodynamic for a system with magnetic work caused by a magnetic induction.

The magnetization, M_α is obtained by inserting the expression for Helmholtz free energy (3.4) and the partition function (3.5) into equation for the magnetization (3.3).

$$M_\alpha = N_\mathrm{A} k_\mathrm{B} T \left(\frac{\partial \ln Z}{\partial B_\alpha}\right)_T = \frac{N_\mathrm{A} k_\mathrm{B} T}{Z}\left(\frac{\partial Z}{\partial B_\alpha}\right)_T = N_\mathrm{A} \frac{\sum_{n=0}^{\infty}\left\{-\frac{\partial E_n}{\partial B_\alpha}\exp\left[\frac{-E_n}{k_\mathrm{B}T}\right]\right\}}{\sum_{n=0}^{\infty}\exp\left[\frac{-E_n}{k_\mathrm{B}T}\right]} \tag{3.6}$$

The differential magnetic susceptibility, $\overline{\overline{\chi}}$ is a second rank tensor with components, $\chi_{\alpha,\beta}$. These components are defined as the first derivative of the magnetization with respect the magnetic induction.

$$\chi_{\alpha,\beta} \equiv \mu_0 \frac{\partial M_\alpha}{\partial B_\beta} \tag{3.7}$$

Insertion of the magnetization, M_α from (3.6) gives a expression for the relation between the magnetic susceptibility and the partition function.

$$\chi_{\alpha,\beta} \equiv \mu_0 \frac{\partial M_\alpha}{\partial B_\beta} = N_\mathrm{A}\mu_0 k_\mathrm{B}T \left(\frac{\partial^2 \ln Z}{\partial B_\alpha \partial B_\beta} \right)_T = N_\mathrm{A}\mu_0 \frac{\partial}{\partial B_\beta} \left(\frac{1}{Z} \sum_{n=0}^{\infty} \left\{ -\frac{\partial E_n}{\partial B_\alpha} \exp\left[-\frac{E_n}{k_\mathrm{B}T} \right] \right\} \right)_T \tag{3.8}$$

The last expression can be made to the following by performing the differentiation with respect to the magnetic induction.

$$\begin{aligned} \chi_{\alpha,\beta} = & \frac{N_\mathrm{A}\mu_0}{k_\mathrm{B}T} \frac{1}{Z^2} \sum_{n=0}^{\infty} \left\{ -\frac{\partial E_n}{\partial B_\alpha} \exp\left[-\frac{E_n}{k_\mathrm{B}T} \right] \right\} \sum_{n=0}^{\infty} \left\{ \frac{\partial E_n}{\partial B_\beta} \exp\left[-\frac{E_n}{k_\mathrm{B}T} \right] \right\} \\ & + \frac{N_\mathrm{A}\mu_0}{Z} \sum_{n=0}^{\infty} \left\{ \left(-\frac{\partial^2 E_n}{\partial B_\alpha \partial B_\beta} + \frac{1}{k_\mathrm{B}T} \frac{\partial E_n}{\partial B_\alpha} \frac{\partial E_n}{\partial B_\beta} \right) \exp\left[-\frac{E_n}{k_\mathrm{B}T} \right] \right\} \end{aligned} \tag{3.9}$$

Equation (3.6) and (3.8) are the fundament for describing the magnetic behavior of matter. The remaining task is to determine the energy, $E_n(B)$ for all states of the system. This is *not* a trivial task and is usually approached numerically, requiring a model for the energies of the relevant eigenstates of the system. In theory the sums in the equations above run to infinity, but the Boltzmann weighting makes only the lowest energy states practically important in determining the magnetic properties. Therefore, model Hamiltonians, which describe only the energetically lowest eigenstates of the systems are useful and represent significant practical simplifications. The most widely used way of generating such effective Hamiltonians is by use of the spin-Hamiltonian formalism.

Spin-Hamiltonians

The effective (spin) Hamiltonian for a polynuclear system consisting of the N paramagnetic metal centres, each with a total spin angular momentum $\overline{S}_\mathrm{A}$ exposed to an external magnetic induction is given as a sum of the exchange interaction contribution and a Zeeman part.

The effective Spin-Hamiltonian consists of contributions arising from the individual centres, $\hat{H}_\mathrm{A}$ and contributions from the interaction between pair of centres, $\hat{H}_\mathrm{AB}$.

$$\begin{aligned}\hat{H} &= \hat{H}_{\mathrm{A}}^{\mathrm{Total}} + \hat{H}_{\mathrm{AB}}^{\mathrm{Total}} = \sum_{\mathrm{A}}^{N} \hat{H}_{\mathrm{A}} + \sum_{\mathrm{A}}^{N} \sum_{\mathrm{A<B}}^{N} \hat{H}_{\mathrm{AB}} \\ &= \sum_{\mathrm{A}}^{N} \left(\hat{H}_{\mathrm{A}}^{\mathrm{Z}} + \hat{H}_{\mathrm{A}}^{\mathrm{ZFS}} \right) + \sum_{\mathrm{A}}^{N} \sum_{\mathrm{A<B}}^{N} \hat{H}_{\mathrm{AB}}^{\mathrm{Exchange}} \\ &= \mu_{\mathrm{B}} \sum_{\mathrm{A}}^{N} \vec{S}_{\mathrm{A}}^{\mathrm{T}} \cdot \overline{\overline{g}}_{\mathrm{A}} \cdot \vec{B} + \sum_{\mathrm{A}}^{N} \vec{S}_{\mathrm{A}}^{\mathrm{T}} \cdot \overline{\overline{D}}_{\mathrm{A}} \cdot \vec{S}_{\mathrm{A}} + \sum_{\mathrm{A}}^{N} \sum_{\mathrm{A<B}}^{N} \vec{S}_{\mathrm{A}}^{\mathrm{T}} \cdot \overline{\overline{J}}_{\mathrm{AB}} \cdot \vec{S}_{\mathrm{B}}\end{aligned} \tag{3.10}$$

The individual parts of the Hamiltonian (3.10) is given as

$$\hat{H}_{\mathrm{A}}^{\mathrm{Z}} \equiv \mu_{\mathrm{B}} \vec{S}_{\mathrm{A}}^{\mathrm{T}} \cdot \overline{\overline{g}}_{\mathrm{A}} \cdot \vec{B} \qquad \hat{H}_{\mathrm{A}}^{\mathrm{ZFS}} \equiv \vec{S}_{\mathrm{A}}^{\mathrm{T}} \cdot \overline{\overline{D}}_{\mathrm{A}} \cdot \vec{S}_{\mathrm{A}} \qquad \hat{H}_{\mathrm{AB}}^{\mathrm{Exchange}} \equiv \vec{S}_{\mathrm{A}}^{\mathrm{T}} \cdot \overline{\overline{J}}_{\mathrm{AB}} \cdot \vec{S}_{\mathrm{B}} \tag{3.11}$$

- were $\hat{H}_{\mathrm{A}}{}^{\mathrm{Z}}$ is the spin Zeeman Hamiltonian which accounts for the orientation dependent interaction between spin and magnetic induction through the second rank tensor $\overline{\overline{g}}_{\mathrm{A}}$. $\hat{H}_{\mathrm{A}}{}^{\mathrm{ZFS}}$ is the zero-field splitting term, which account for anisotropy on the individual centres. A wide variety of contributions can be included in the $\hat{H}_{\mathrm{A}}$ term such as orbital Zeeman, spin-orbit coupling, hyperfine interaction between electron and nucleus.

$\hat{H}_{\mathrm{AB}}{}^{\mathrm{Exchange}}$ is the magnetic exchange Hamiltonian and is of particular importance for understanding magnetic exchange properties of systems with more paramagnetic metal centers. The spin-spin interaction tensor, $\overline{\overline{J}}_{\mathrm{AB}}$ is a second rank tensor, which in its most general form consists of nine components. A description that fully account for the nine general tensor components is often not necessary. Contributions from some components will be more significant than others. Likewise, will the presence of symmetry give rise to a reduction in the number uniqe tensor components, sometimes with sufficiently high symmetries, forcing it to be diagonal. It is appropriate to make rearrangement of the general tensor in order to clarify and symmetry adapt the individual contributions.

The effective Spin-Hamiltonian, $\hat{H}_{\mathrm{AB}}$ can be expressed as sum of three individual contributions.

$$\hat{H}_{\mathrm{AB}}^{\mathrm{Iso}} \equiv J_{\mathrm{AB}} \left(\vec{S}_{\mathrm{A}} \cdot \vec{S}_{\mathrm{B}} \right) \qquad \hat{H}_{\mathrm{AB}}^{\mathrm{Antisym}} \equiv \vec{d}_{\mathrm{AB}} \cdot \left(\vec{S}_{\mathrm{A}} \times \vec{S}_{\mathrm{B}} \right) \qquad \hat{H}_{\mathrm{AB}}^{\mathrm{Aniso}} \equiv \vec{S}_{\mathrm{A}} \cdot \overline{\overline{D}}_{\mathrm{AB}} \cdot \vec{S}_{\mathrm{B}} \tag{3.12}$$

Where the first, Heisenberg-Dirac-van Vleck operator, $\hat{H}_{\mathrm{AB}}{}^{\mathrm{Iso}}$ account for the isotropic exchange interaction, the second Dzyaloshinskii-Moriya operator, $\hat{H}_{\mathrm{AB}}{}^{\mathrm{Antisym}}$ for the antisymmetric exchange and the third operator, $\hat{H}_{\mathrm{AB}}{}^{\mathrm{Aniso}}$ account for asymmetric exchange. J_{AB} in Heisenberg-Dirac-van Vleck operator is the exchange coupling constant. Depending on the sign of the constant the exchange interaction is said to either be ferromagnetic ($J_{\mathrm{AB}} < 0$) or antiferromagnetic ($J_{\mathrm{AB}} > 0$).

The general expression obtained by insertion of (3.12) into (3.11) is in many situations not nessesary. Often the isotropic contribution is the only one of significance in the description:

$$\hat{H}_{AB}^{Total} = \sum_{A}^{N}\sum_{A<B}^{N}\hat{H}_{AB} \approx \sum_{A}^{N}\sum_{A<B}^{N}\hat{H}_{AB}^{Iso} = \sum_{A}^{N}\sum_{A<B}^{N} J_{AB}\left(\vec{S}_A \cdot \vec{S}_B\right) \tag{3.13}$$

More generally, the interaction can be considered to have an anisotropy dominated by the metal-metal direction (choosen as the z-direction), and the interaction part of the Hamiltonian can then be forrmulated as:

$$\hat{H} = -J\sum_{i=1}^{N-1}\left[\alpha\hat{S}_{i,z}\hat{S}_{i+1,z} + \beta\left(\hat{S}_{i,x}\hat{S}_{i+1,x} + \hat{S}_{i,y}\hat{S}_{i+1,y}\right)\right]$$

Depending on the two coefficients α and β the Hamiltonian for the magnetic exchange reduce o the classical limiting cases of the *Heisenberg model* ($\alpha = 1$, $\beta = 1$), *Ising model* ($\alpha = 1$, $\beta = 0$) and the *XY mod*el ($\alpha = 0$, $\beta = 1$).

Application of the Spin-Hamiltonian formalism

In the following, we consider a dinuclear system consisting of the magnetic centers, A and B exposed to a magnetic induction, $\overline{B}$ with only isotropic exchange interaction present. The effective Spin-Hamiltonian of such a system, is according to (3.10) and (3.13) given by:

$$\hat{H} = \hat{H}_A^Z + \hat{H}_B^Z + \hat{H}_{AB}^{Iso} = \mu_B\left(\vec{S}_A \cdot \overline{\overline{g}}_A + \vec{S}_B \cdot \overline{\overline{g}}_B\right)\cdot\vec{B} + J_{AB}\left(\vec{S}_A \cdot \vec{S}_B\right) \tag{3.14}$$

Assume further that the individual magnetic centers possess identical isotropic spin, then $\overline{\overline{g}}_A = \overline{\overline{g}}_B = g$. The total spin, $\overline{S}$ is obeys the following:

$$\vec{S} = \vec{S}_A + \vec{S}_B \qquad \vec{S} = \left|\vec{S}_A - \vec{S}_B\right|,\ldots,\vec{S}_A + \vec{S}_B \tag{3.15}$$

By introduction of (3.15) in (3.14) the Spin-Hamitonian can be rewritten as:

$$\hat{H} = \tfrac{1}{2}J_{AB}\left(\vec{S}^2 - \vec{S}_A^{\,2} - \vec{S}_B^{\,2}\right) + \mu_B g B \cdot \vec{S} \tag{3.16}$$

and the energy levels are:

$$\begin{aligned} E_{S,M_S}(B) &= \langle S,M_S \,|\, \hat{H} \,|\, S',M_S'\rangle \\ &= \tfrac{1}{2}J_{AB}\left(S(S+1) - S_A(S_A+1) - S_B(S_B+1)\right) + \mu_B g B M_S \end{aligned} \tag{3.17}$$

The meaning of the J_{AB} parameter appears from (3.17) as a measure of the energy difference between the $2S + 1$ fold degenerate S-states. In the absence of a magnetic induction ($B = 0$) the energy difference between any two consecutive states S and $S + 1$ is given as:

$$\Delta E_{S,S+1} \equiv E_{S+1}(B=0) - E_S(B=0) = J_{AB}\left(S+1\right) \tag{3.18}$$

Equation (3.17) gives an expression for the energy of all states of the system, which provides the necessary information to determine the partition function, Z by insertion in (3.5).

$$Z = \sum_{S=|S_A-S_B|}^{S_A+S_B} \sum_{M_S=-S}^{+S} \exp\left[\frac{-E_{S,M_S}(B)}{k_B T}\right] = \sum_{S,M_S} \exp\left[\frac{JS(S+1)}{2k_B T} + \frac{\mu_B g B M_S}{k_B T}\right] \tag{3.19}$$

This expression can be used directly in combination with the appropriate derivatives of the energy with respect to the magnetic induction to determine the magnetic properties of the system. This is achieved by insertion in the expressions for the magnetization (3.6) or the differential magnetic susceptibility (3.8). The exact expression obtained by this procedure is even for this simple system quite complicated. Commonly certain approximations are introduced by assuming that the exponential part of (3.19) depending on the magnetic induction can be expanded as a Taylor series and truncated after the linear part.

$$\exp\left[\frac{-E_{S,M_S}}{k_B T}\right] = \exp\left[\frac{-JS(S+1)}{2k_B T} - \frac{\mu_B g B M_S}{k_B T}\right] \approx \exp\left[\frac{-JS(S+1)}{2k_B T}\right]\left(1 - \frac{\mu_B g B M_S}{k_B T}\right) \tag{3.20}$$

An approximate expression for the partition function is obtained by insertion of (3.20) into (3.19).

$$\begin{aligned} Z &\approx \sum_{S,M_S}\left\{\exp\left[\frac{-JS(S+1)}{2k_B T}\right]\left(1 - \frac{\mu_B g B M_S}{k_B T}\right)\right\} \\ &= \sum_{S,M_S} \exp\left[\frac{-JS(S+1)}{2k_B T}\right] - \mu_B g B \beta \sum_{S,M_S} M_S \exp\left[\frac{-JS(S+1)}{2k_B T}\right] = \sum_{S,M_S} \exp\left[\frac{-JS(S+1)}{2k_B T}\right] \end{aligned} \tag{3.21}$$

At the last equality sign it is used that:

$$\begin{aligned} &\sum_{S,M_S}\left\{M_S{}^n \exp\left[\frac{-JS(S+1)}{2k_B T}\right]\right\} = \sum_{S}\left\{\exp\left[\frac{-JS(S+1)}{2k_B T}\right]\right\}\sum_{M_S=-S}^{+S} M_S{}^n \\ &\sum_{M_S=-S}^{+S} M_S{}^n = \begin{cases} 2S+1 & \text{for } n=0 \\ 0 & \text{for } n=1 \\ \frac{1}{3}S(S+1)(2S+1) & \text{for } n=2 \end{cases} \end{aligned} \tag{3.22}$$

The magnetization is obtained by insertion of (3.20) and (3.21) into (3.6) together with the first derivative of the energy with respect to the magnetic induction given directly by (3.17).

$$M = \frac{N_A \mu_B{}^2 g^2 B}{k_B T} \frac{\sum_{S,M_S} \left\{ M_S{}^2 \exp\left[\frac{-JS(S+1)}{2k_B T}\right]\right\}}{\sum_{S,M_S} \left\{ \exp\left[\frac{-JS(S+1)}{2k_B T}\right]\right\}} = \frac{N_A \mu_B{}^2 g^2 B}{3k_B T} \frac{\sum_{S} \left\{ S(S+1)(2S+1) \exp\left[\frac{-JS(S+1)}{2k_B T}\right]\right\}}{\sum_{S} \left\{ (2S+1) \exp\left[\frac{-JS(S+1)}{2k_B T}\right]\right\}} \tag{3.23}$$

At the last equality sign the sum over M_S has been evaluated using (3.22).
The differential magnetic susceptibility is given as the first derivative of the magnetization according to (3.8).

$$\chi = \mu_0 \frac{\partial M}{\partial B} = \frac{N_A \mu_0 \mu_B{}^2 g^2}{3k_B T} \frac{\sum_{S} \left\{ S(S+1)(2S+1) \exp\left[\frac{-JS(S+1)}{2k_B T}\right]\right\}}{\sum_{S} \left\{ (2S+1) \exp\left[\frac{-JS(S+1)}{2k_B T}\right]\right\}} \tag{3.24}$$

This result is equivalent to that obtained by use of the van Vleck equation for the same model system. Actually, the derivation of the van Vleck equation follows directly from the same pattern as just used combined with the general assumption that the energy of the states can be expressed by a Taylor series expansion with respect to the magnetic induction and the only that linear part with respect to the magnetic induction is important. The above algebra can be made more concrete by considering a dinuclear, high-spin Mn(III) complex, which would have S_A=S_B=S=2:

$$\chi_{S_A,S_B=2} = \frac{N_A \mu_0 \mu_B{}^2 g^2}{k_B T} \frac{2\exp(x) + 10\exp(3x) + 28\exp(6x) + 60\exp(10x)}{1 + 3\exp(x) + 5\exp(3x) + 7\exp(6x) + 9\exp(10x)} \quad \text{for } x \equiv \frac{-J}{k_B T} \tag{3.25}$$

Analysis of data

The magnetization data obtained for clusters containing infinit chains are often in the literature analyzed by the use of different models as the frequently used exact semi-classical Fisher-model or high-temperature expansion model. $[Cu_2(dpa)_2(\mu\text{-}F)_2(\mu_4\text{-}SiF_6)]\cdot 2MeOH$ is analyzied on the basis of the Bleaney-Bowers expression for dinuclear Cu(II) compounds.

$$\chi(T)=\frac{N_A\mu_0\mu_B{}^2g^2S(S+1)}{3k_BT}\cdot\frac{1+\coth u-u^{-1}}{1-\coth u+u^{-1}}\quad \text{were}\quad u=\frac{2JS(S+1)}{k_BT} \tag{3.26}$$

While restriction to S=½ yields the so-called Bleaney-Bowers expression:

$$\chi(T)=\frac{N_A\mu_0\mu_B{}^2g^2}{3k_BT}\left\{1+\frac{1}{3}\exp\left[\frac{-2J}{k_BT}\right]\right\} \tag{3.27}$$

which has often been used for the analysis of magnetic data for dinuclear Cu(II) systems. A fluoride-bridged example of this is the study of $[Cu_2(dpa)_2(\mu\text{-}F)_2(\mu_4\text{-}SiF_6)]\cdot 2MeOH$. [82]

Analysis of data for infinite systems

Obviously, all of the above considerations lose their practical applicability for systems containing an infinite (or just very large) number of interacting spins. Therefore, magnetization data obtained for systems containing *e.g.* infinite chains are often analysed in the literature by the use of different models. A very frequently employed description is the exact semi-classical Fisher-model. Alternatives encompass high-temperature expansion approaches (*cf.* Chapter 4).

EXCHANGE MECHANISMS AND SOME SELECTED DATA

While the Spin-Hamiltonian formalism provides a convenient parameterization of magnetic data in general and magnetic exchange in particular, it does not provide any direct physical picture of the mechanism of the exchange interactions. The exchange interaction between the localized magnetic moments on the cluster ions can in principle take place in a number of different ways - either as a:

- **Direct exchange interaction** where the coupling takes place through a direct overlap between the magnetic orbitals. This is a strong but short-ranged interaction.

- **Superexchange interaction** where the coupling proceeds via a diamagnetic bridge which connects the magnetic orbitals. This is a strong long-ranged interaction.

Of these two coupling mechanisms, it is predominantly the latter indirect exchange which is observed, even in cases where direct overlap between the magnetic orbitals is a possibility *e.g.* $3d$ transition metal ions as opposed to the $4f$ lanthanoid ions.
A simplified picture of the super exchange mechanisms can be seen in Figure 9 where a bridging ligand with possibility for both σ- and π-interaction has been depicted.
Clearly it must be required that the symmetry of the magnetic orbitals of the paramagnetic metal centers and of the diamagnetic bridge allows for an adequate overlap. This overlap can normally be of σ- or π-nature depending on whether it is the e_{2g} or t_{2g} orbitals of the metal centre that overlap with a suitable p-orbital (or hybrid orbital) on the bridge, as shown in the Figure 9. The interaction via an σ-type overlap is normally stronger than via a π-type overlap. As opposed to direct spin-polarization of intervening bonds, the super-exchange mechanism can give rise to either parallel (ferromagnetic) or anti parallel (anti ferromagnetic) arrangement of the interacting spins. Due to the importance of the orbital overlap, the strength of interactions mediated by the super exchange mechanism will typically depend on the bond angle of the bridge, as seen in Figure 10.

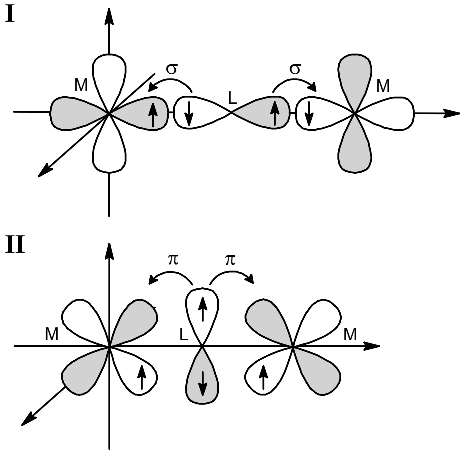

Figure 9 Mechanism for superexchange interaction

One particular physical model of magnetic exchange deserves a special note. In the so-called Anderson theory for magnetic exchange the exchange coupling between interacting spins is analysed in terms of the interaction of the ground state with energetically high-lying excited states (on top of the zero'th order term stemming from direct electron-electron interaction). [83, 84] Important contributions stem from interaction with metal-to-metal charge transfer states (kinetic exchange) and metal-to-ligand as well as ligand-to-metal charge transfer states. With fluoride as the bridging ligand, both of the latter mechanisms are as energetically unfavorable. If these mechanisms were dominant, it would be expected, that fluoride would be an extermely poor mediator of magnetic exchange. However, as we shall see in the following, Nature is more kind than so.

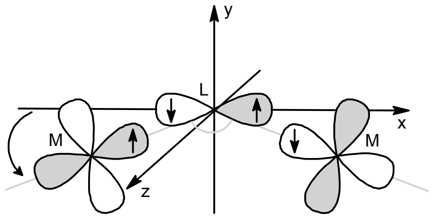

Figure 10 Variation of overlap with respect to bond angle

Magnetic exchange in fluorido complexes

As stated in the previous Chapter on the structural aspects fluoride as a bridging ligand, the overall picture can be significantly affected by a complex structural arrangement where several types of fluoride bridges (μ_2-, μ_3-, μ_4-, μ_5-, and μ_6-) alone or in combination with other supporting bridges as carboxylates. Identification and understanding of a magneto-structural correlations specifically assigned to a particular type of fluoride bridge is thus made difficult and requires in principle that the structure is well determined and simple. Based on the same criteria as stated earlier, the systems where fluoride acts as an unsupported bridging ligand has been selected focusing on mono, di and tri(μ_2-fluorido) containing systems. By removing the compounds with diamagnetic metal centres *e.g.* Zr(IV), V(IV) and W(VI), the number of remaining systems with the possibility of magnetic exchange interaction is limited in number. The number of systems and the limited degree, to which they are magnetically characterized, however, means that it is still difficult to draw really general conclusions about correlations between structural features and magnetic properties.

Comparing the exchange coupling interactions of a number of pentafluoridomanganate(III), $[MnF_5]^{2-}$, salts which is a 1D chain, shows a clear correlation with respect to the bridging angle $\beta_{Mn\text{-}F\text{-}Mn}$ and shows no or little dependence of Mn–Mn/Mn–F distances, indicating the importance of the overlap between the p_z orbital of the fluorido ligand and $d_z{}^2$ orbital on Mn(III). [85] In case of μ_2-halogenido complexes it is generally observed that the magnetic exchange decreases when the bond angle of the bridge, β is compressed from the linear coordination. This relationship can be exemplified by the series of Mn(III) mono(μ_2-fluorido) complexes, $A_2[MnF_5]\cdot nH_2O$ (A= *s*-block cation) were a compression of bond angle going from the Cs-complex (β = 180 °) to the Li-complex (β = 121.5 °) results in a decrease in magnetic exchange

corresponding to a change in J from –16.5 cm^{-1} to –6.5 cm^{-1}. Variation of the exchange interaction with respect to bond angle and the μ-fluorido bridge seems to be quite general; however, it seems difficult to generalize the quantitative significance of this variation from one transition metal to another. Even relatively close structurally related complexes, with little variation in bridge bond angle, β may show relatively large variation in the exchange interaction, J. This situation is observed in Cu(II) complexes $[Cu_2(dpa)_2(\mu\text{-}F)_2(\mu_4\text{-}SiF_6)]\cdot 2MeOH$ (dpa= 2,2′-dipyridylamine) (J = –74 cm^{-1}, β = 99.85(8) °), [82] and $[Cu_2(mppzH)_4(\mu\text{-}F)_2](BF_4)_2$ (mppzH= 3-methyl-5-phenylpyrazole) (J = –118 cm^{-1}, β = 98.9 °), [86]. It is also worth noting that even closely related ligand systems can give rise to large variation in structural and magnetic properties. This becomes evident when comparing abovementioned Cu(II) systems with the substituted 5-phenylpyrazole complex $[Cu(R,R'\text{-}phpz)_2(\mu\text{-}F)_2]$ (R,R'-phpz= 3R,4R'-5-phenylpyrazol and (R,R') = (H,H); (Me,Me); (Me, Et)) which in first case exhibit ferromagnetic exchange coupling (J = –2.8 cm^{-1}) and in the latter cases at best weak antiferromagnetic coupling (J = +0.2 and +0.6). [87] The intra nuclear distance between the paramagnetic metal centres in a dimer cluster decreases with the number of fluoride bridges connecting them. In spite of even very short intranuclear distances in di- and tri(μ_2-fluorido) complexes exchange interaction is in general seems not to be direct. This is exemplified by the mono(μ_2-fluorido) Ni(II) complex $[Ni_2L_4(\mu_2\text{-}F)_2](BF_4)_3$ (L= 2,5,8-trithia[9],(2,9)-1,10-phenanthrolineophane) and the Cu(II) complex $[Cu_2(mpz)_2(dmpz)_4(\mu_2\text{-}F)_2](BF_4)_2$ (mpz= 3-methylpyrazole and dmpz= 3,5-dimethylpyrazole) with M–M' distance of 3.887(1) and 2.9962(9) Å respectively. For the Ni(II) complex a significant antiferromagnetic exchange was reported, while the dinuclear copper system showed no detectable exchange down to 2 K despite a Cu–Cu' distance of 2.9962(9) Å. [88]

Quite a number of tri-μ_2 fluorido complexes have been prepared and structurally characterized, but most of these feature diamagnetic metal centres or have not been magnetically characterized, and magneto-structural therefore cannot be established.
Since the magnetic exchange between the paired ions, assuming a super-exchange mechanism passes through the bridge many of the exchange properties depends on the chemically nature and structural properties of the bridge. The potential influence of the bridge nature on the exchange can be seen by comparing the J = –40 to –67 cm^{-1} found in the μ_2-fluorido complex $[Ni_2L_4(\mu_2\text{-}F)](BF_4)_3$ (L= 2,5,8-trithia[9],(2,9)-1,10-phenanthrolineophane) with J = –74 cm^{-1} for the μ_2-chlorido $[Ni_2L'_2(\mu_2\text{-}Cl)](PF_6)_3$ (L'= 2,6-bis(1′,3′-diamino-2′-methylpro-2′-yl)pyridine). This change in the magnetic exchange must be seen in light of an increase in bond angle, β from 161.31(12) to 165.5(3) ° On this note, we can conclude the chapter, by reiterating that fluoride despite being unpolarizable and its complexes having charge transfer states of very high energies, act as a decent mediator of magnetic exchange.

CHAPTER 4

Mn(III) FLUORIDO COMPLEXES – CHAINS AND DISCRETE SYSTEMS

INTRODUCTION

Manganese in oxidation state +3 has been subject of considerable research. Two discoveries have in several ways acted as promoters for work within the field of Mn(III) chemistry:

- Single molecular magnetism.
- Catalytic behaviour in organic chemistry.

It is not possible to attribute these attractive properties of Mn(III) complexes exclusively to the metal center, but they arise as a combined result of the manganese center and the surrounding ligand framework. Among the coordination chemistry of manganese, $[Mn^{III}(salen)]^+$ stands out by having played important roles in both catalysis and magnetism. This system constitute one of the most ubiquitous building blocks used in molecule-based magnetic systems. From this building block, homo- as well as hetero-metallic systems with both terminal and bridging ligands have been synthesized and investigated. In spite of this attention received by the $[Mn^{III}(salen)]^+$ building block, simple fluorine-containing systems have in practice not been described in literature. In the present project synthesis and structural characterization of *catena*-[Mn(μ-F)(salen-5H)] and the derivatives *catena*-[Mn(μ-F)(salen-5R)] for R=F, Cl, “Br” was achieved. In the first three of these systems, unsupported fluorido ligands bridge a homo-metallic manganese chain. The magnetic properties of these systems demostrate that the fluoride bridges, despite any preconcieved notions, facilitate moderately strong magnetic interactions

when acting as linear bridges.[3] The magnetic exchange coupling across the fluorido bridge can be modelled by use of a different theoretical methods with varying ranges of applicability, namely the Fisher model, exact diagonalizations of finite-size rings or the high-temperature expansion method.

Despite the very rich chemistry of the $[Mn(salen)]^{+}$ fragment, *catena*-[Mn(μ-F)(salen-H)] is the first and only structurally characterized example of a mono-atomic bridge salen-ligated complex, not only for Mn(III) but for all elements. This make the determination and the fundamental understanding of the fluorido ligands properties with respect to the magnetic interaction between two paramagnetic metal centers more authoritative compared to poly atomic ligands, with or without suporting ligands. *catena*-[Mn(μ-F)(salen-H)] is also interesting since the infinite chain structure is broken up into monomeric [Mn(F)(salen-H)] in solution. Addition of fluoride in excess give rice to *in situ* formation of the anion *trans*-$[Mn(F)_2(salen\text{-}H)]^{-}$. Both [Mn(F)(salen-H)] and *trans*-$[Mn(F)_2(salen\text{-}H)]^{-}$ in solution shows well defined axial coordination of the fluorido ligands as determined by parallel mode EPR spectroscopy. On basis of characterization of these two fluorido complexe, it was possible to resolve the superhyperfine interction in the hexafluoridomagnanate(III) ion, $[MnF_6]^{3-}$. This represent the first detailled determination of the superhyperfine interction in a Mn(III) complex.

This chapter gives a brief introduction to general Mn(III) chemistry, and the magnetic properties of this class of system in particular with focus on Jahn-Teller effects and EPR studies. The development of the preparative method for *catena*-[Mn(μ-F)(salen-R)] for R=H, F, Cl, “Br” is reviewed. Work on fluorido containing complexes of Mn(III) has formed basis of two papers. One with the synthesis, structural and magnetic charaterization of *catena*-[Mn(μ-F)(salen-5H)] (appended to the thesis as Paper 5) and another with the determination of the superhyperfine interaction in $[MnF_6]^{3-}$ (appended to the thesis as Paper 1).

[3] Single crystal X-ray diffractions data of [Mn(F)(salen-5Br)] and the possible solution gives no clear answer to whether the complex crystallize in as a infinite chain structure as in the other cases (R=H, F, Cl) or if this complex in particular crystallizes as discrete units. If the bromo-substituted complex is given in a context that can be read as the complex with certainty is present in the crystalline phase with a chain structure the symbol “Br” is used to indicate the here given reservations - this apply to the rest of the discussion of this complex type.

THE CHEMISTRY OF Mn(III)

The chemistry of manganese in general and Mn(III) specifically has been reviewed several times in literature. [89, 90] A serious and critical, but somewhat outdated, review of Mn(III) is found in *"Gmelin Handbuch der Anorganischen Chemie"*. [91, 92]

Mn(III) has a d^4 electron configuration and most commonly forms high-spin octahedral complexes. The Mn(III) ion is generally a strong oxidant and disproportionate in aqueous solution to Mn(II) and Mn(IV) – the latter generally in form of the sparingly soluble MnO_2. In spite of the redox activity, Mn(III) exhibit a extensive coordination chemistry due to a significant lowering of the reduction potential in presence of a suitable ligand sphere and consequent stabilization of this oxidation state. The incorporation of Mn(III) in the coordination entity is in genereal based on ligand substitutions or redox reactions such as oxidation of Mn(II), reduction of Mn(VII) or the frequently used method by comproportionation between Mn(II) and Mn(VII). Not all ligator atoms brings about the necessary decrease in reduction potential to enable a stable Mn(III) containing unit. Coordination complexes of Mn(III) must in general be stabilized by ligands containing hard ligators such as, oxygen alone or in combination with nitrogen. In the presence of a stabilizing ligand such as cyanide CN^- or some polydentate ligands, Mn(III) can be obtained directly from a suitable Mn(II) species by oxidation *e.g.* with air. The cyanide complex, $[Mn(CN)_6]^{3-}$ can alternatively be obtained by ligand substitution in *e.g.* $MnPO_4{\cdot}H_2O$ [93] together with a few other examples like $[Mn(CN)_5(OH)]^{3-}$ this ion belong to the few known low-spin Mn(III) (d^4 electron configuration, $t_{2g}{}^4e_g{}^0$). Most systems are high-spin; $t_{2g}{}^3e_g{}^1$.

In the following sections Mn(III) systems with ligand spheres dominated by the following ligator atoms: Fluorine, Oxygen, Nitrogen and Oxygen/Nitrogen in combination (with focus on Schiff-base complexes) are discussed. The description is connected with certain parts of the following section "Synthetic strategy for $3d$–(μF)–$3d$ bridged systems" and those two sections complement each other to form a whole.

Ligand spheres encompassing fluorine ligator atoms

A number of simple fluorido complexes of Mn(III) are known. All these take the general stoichiometric form $[Mn^{III}F_n]^{3-n}$ were n=1–6. [91] All of these fluoridomanganate(III) ions are found in varying amount in aqueous solution after a composition between Mn(II) and MnO_4^- according to following general reaction equation:

$$4\,Mn^{2+} + MnO_4^- + 8\,H^+ + 5n\,F^- \xrightleftharpoons{H_2O} 5\,[MnF_n]^{3-n} + 4\,H_2O \qquad (4.1)$$

The stability of the individual species depends on the fluoride ion concentration and are highest for the fluoridomanganate(III) ions, $[Mn^{III}F_n]^{3-n}$ with n=4–6. Again reflecting the stability constants, several of the species are hydrolyzed to varying degree by water. Well defined

complexes with or without association of crystal water have been described in case of *n*=3–6 and are known specifically in salts of alkali and ammonium cations. As a crude rule, it is found that $AMn^{III}F_4$ precipitates with alkali metal ions with small ion radius such as Li and Na, while the compounds of stoichiometry $A_3Mn^{III}F_6$ precipitates with ions of large radius as Rb and Cs. The synthetic method for the different fluoridomanganate(III) systems varies. Only in case of $[MnF_5]^{2-}$ is it possible to carry out the synthesis in aqueous solution. Two approaches have been reported: either dissolution of a suitable Mn(III) compound, commonly the oxides MnOOH or Mn_2O_3 but also hydroxide $Mn(OH)_3$ in hydrofluoric acid or alternatively comproportionation between Mn(II) and MnO_4^- or MnO_2 in hydrofluoric acid. Both the $[MnF_4]^-$ and $[MnF_6]^{3-}$ are prepared by reactions at high temperature. In case of $[MnF_4]^-$ generally by reduction of $[Mn^{IV}F_5]^-$ with hydrogen and in case of $[MnF_6]^{3-}$ frequently by direct reaction between alkali metal fluoride and MnF_3. However, one exception exists. Despite that $[MnF_6]^{3-}$ is hydrolyzed completely in water, it can be prepared by comproportionation between Mn(II) and Mn(VII) in a mixture of HF and NH_4F, and then be isolated as sparingly soluble salts of $[M(NH_3)_6]^{3+}$ for M=Cr, Co, Rh. [94]
All Mn(III) centers in the above mentioned fluoridomanganates(III) complexes are linked by a single fluorido bridge, Mn–(μ-F)–Mn. Systems with double fluorido bridges, $Mn–(\mu\text{-}F)_2–Mn$ are also known including the piperazine (pipz) complexes $pipzH_2[Mn_2F_8]$, [95] $(pipzH_2)_3[Mn_4F_{18}(H_2O)]\cdot H_2O$ and $(pipzH_2)_4[Mn_2F_9]_2[MnF_4(H_2O)_2][MnF_4(HF)_2]$ [96]. The $[Mn_2F_8]^{2-}$ of the fist complexes consist edge-sharing MnF_6-octahedra, $[(\mu\text{-}F)F_2Mn(\mu\text{-}F)_2MnF_2(\mu\text{-}F)]^{2-}$ assembled in a infinite layer structure. A structural motif also seen, slightly modified in the remaining complexes.

The fluorido complexes of Mn(III) described so far have all being simple in the sense that the Mn(III) centre in all cases exclusively has been coordinated by fluorido ligands. Simple complexes with mixed ligand sphere consisting of fluorido ligands as well as oxygen and/or nitrogen donor ligands are also known, but their number is however considerably smaller than that of the homoleptic fluoridomanganate(III) complexes. In the mixed ligator systems, a predominance of oxygen donor ligands over nitrogen donor ligands is observed. This, probably, originates from the difference in hardness between the oxygen and nitrogen ligator atoms and a pronounced hardness of the Mn (III) centre. According to the HSAB principle (Chapter 2) the bond formation, which involve interaction of species with matching hardness is favoured. An illustration of the difference in hardness between oxygen and nitrogen in this type of systems are the $[Mn(H_2O)_6]^{3+}$ and $[Mn(NH_3)_6]^{3+}$ complexes. Of these, the former is well known and characterized, while the latter is unknown. The hexaaquamanganese(III) ion is found *e.g.* in the alum $[A(H_2O)_6][Mn(H_2O)_6](SO_4)_2$ for A=Rb, Cs synthesized by Christensen, in 1900. [97, 98]
Another problem in synthesis of Mn(III) complexes with nitrogen donor ligands often in form of amines or heteroaromatic ligands as pyridine, 1,10-phenantroline, or 2,2'-bipyridine is the basicity of the nitrogen ligator atoms. In the presence of protic solvents, protolysis leading to

increasing pH. This complicates matters due to the instability of Mn(III) is in aqueous solution towards disproportionation which is favored at high pH by the formation of the very insoluble MnO_2. Successful synthesis of nitrogen donor complexes thus requires a concentration window, which favors the desired product by taking into account the ligand and solvent acid/base properties as well as undesired disproportionation of Mn(III) and the possibility of undesired complex formation with ligators with a higher hardness than the nitrogen donor ligand *e.g.* solvents.

Disproportionation of Mn(III) in aqueous solution in the presence of phen and bpy to dioxobridged $[Mn_2O_2]^{3+}$ has been described. [99] Nevertheless, proper complexes of these classical ligands are known in case of the trifluorido system $[Mn(L'')F_3(H_2O)]$ for L″=phen, bpy. [99-101] Both complexes are synthesized in aqueous HF. If bpy is replaced by 4,4′-bipyridine (4,4′-bpy) the mixed fluorido/aqua complex 4,4′-bpy$H_2[MnF_4(H_2O)_2]_2\cdot 2H_2O$ can be isolated. The reasons for the different outcome is not discussed in the literature. However, aside from crystal packing effects, the dependence on the nature of the bipyridine ligand is in line with the discussion above and the difference in basicity constants[4] These basicity constants [102] show a substantial difference in Brønsted base strength between the two ligands, with 4,4′-bpy being the most readily protonated of the two in good agreement with the observed products. Nevertheless, complexation with 4,4′-bpy is possible as witnessed by the fact that [Mn(4,4′-bpy)F_3] with a polymeric layer structure has been obtained by diffusion in a less protic organic solvent. [103] Parallel reactions leading to isolation of salts of *trans*-$[MnF_4(H_2O)_2]^-$ was seen in case of the ligand systems 1,2-bis(4-pyridyl)ethane and *trans*-1,2-bis(4-pyridyl)ethylene. [104] In addition to the bidentate heteroaromatic ligands, complexes based on tridentate ligands as 2,2′:6,2″-terpyridine (terpy) and *N,N′*-bis(2-pyridylmethyl)-ethylamine (bpea) of general form $[Mn(L''')F_3]$ are known. [105] Both complexes were synthesized by reaction of MnF_3 and the respective ligand in MeOH at room temperature. The analogous complexes of the quite basic azide ligand are also known, but obtained by ligand substitution from $Mn(OAc)_3\cdot 2H_2O$.

A number of fluorido complexes with oxygen donor atoms from other ligands than water are known. These ligands include *e.g.* oxalate ($C_2O_4^{2-}$) and phosphates (HPO_4^{2-} and $H_2PO_4^-$) as seen in the complexes $K_3[Mn(C_2O_4)_2F_2]\cdot 3H_2O$, pyH$[Mn(C_2O_4)F_2(H_2O)_2]$, $K[Mn(HPO_4)_2F_2]\cdot 3H_2O$ and [Mn(2,2′-bpy)$F_{3-n}(H_2PO_4)_n$] for n=1,2. [101, 106, 107]

Ligand spheres encompassing oxygen ligator atoms

With regard to coordination chemistry, the most important and abundant group of Mn(III) complexes is the one where the compounds are stabilized by a ligand field of oxygen ligator atoms. This is seen in cases of Mn_2O_3, MnOOH and the simple solid-state manganate(III) ions

[4] Acid dissociation constants for 2,2′-bipyridine: pK_{a1}= 4.42, pK_{a2}= 1.5 and 4,4′-bipyridine: pK_{a1}= 4.77, pK_{a2}= 2.69.

were the ligator atom enters as oxide or hydroxide as in $[MnO_2]^-$ and $[Mn(OH)_n]^{3-n}$ (for n=5-6) respectively. [108, 109] Oxygen ligator atoms can originate from inorganic oxo anions such as SO_4^{2-}, [97, 98, 111] and PO_4^{3-}, [93, 112] but more predominantly organic carboxylates constitute an important class in manganese(III) chemistry. Especially noteworthy is the acetate $Mn(OAc)_3{\cdot}nH_2O$ (n=0, 2) but also the oxalate $[Mn(ox)_3]^{3-}$, [98, 113] and malonate, $CH_2(COO)_2^{2-}$ [114]. The hydrated acetate, $Mn(OAc)_3{\cdot}2H_2O$ is obtained by the method of Christensen, which involve a comproportionation between $Mn(OAc)_2{\cdot}4H_2O$ and MnO_4^- in glacial acetic acid. [97, 98] The alternative solvent free $Mn(OAc)_3$ can obtained by reaction of $Mn(NO_3)_2{\cdot}6H_2O$ with hot acetic anhydride. This method is effective, but personal experience dictates that great care should be taken due to the highly exothermic reaction. [111] Structurally, $Mn(OAc)_3{\cdot}2H_2O$ differs from the apparent stoichiometry by containing an oxo core with three Mn(III) ions. Thus the aqueous acetate is described more correctly as $[Mn_3O(\mu_2\text{-}OAc)_6]OAc$. [19] This structural motif with a, μ_3-O bridged metal triangle is found in other carboxylate species of trivalent elements *e.g.* V, Cr, Fe and Ru. [19] Other derived Mn-species with of mixed valence containing the μ_3-O motif are known *e.g.* $[Mn_3O(\mu_3\text{-}OAc)_6(py)_3]$ with an average manganese oxidation state of ~2⅔. [115]

For Mn(III) as for most other metal centers carboxylate groups tend to form polynuclear species. The comproportionation reaction of Mn(II) and Mn(VII) can be tuned give rise to a variety of different polynuclear species with manganese in oxidation states +3 or +4 either alone or mixed. The product composition depends on the ratio between the reactant species. The classic example of this synthetic method leading to mixed Mn(III)/Mn(IV) complexes is the famous dodecanuclear complex with stoichiometric formula, $[Mn_{12}(OAc)_{16}(H_2O)_4O_{12}]{\cdot}2AcOH{\cdot}4H_2O$ (often abbreviated as Mn_{12}) prepared and structurally characterized by Lis in 1980. [116] The complex consists of a central Mn_4^{IV} cubane unit surrounded a ring of alternating Mn(III) and oxide ions as depicted in Figure 11, the outher rim being blocked by carboxylate ligands. This structure gives the high total spin of S = 10). [117, 118] Analogous compounds with other carboxylate ligands can be prepared either by ligand substitution of acetate with *e.g.* propionate, benzoate, crotonate, [117] or directly *e.g.* trifluoroacetate. These systems exhibit single-molecule magnet properties. [110]

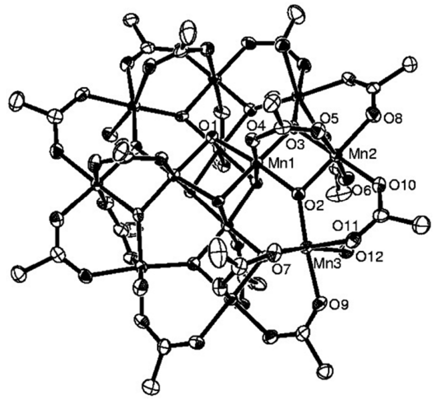

Figure 11 Crystal structure of Mn_{12}-analogue

$[Mn_{12}(CF_3COO)_{16}(H_2O)_4O_{12}]{\cdot}2CF_3COOH{\cdot}4H_2O$
Figure adopted form reference [110]

Another large group of related compounds of Mn(III) are based on β-diketones as an example the simple tris-complex of pentan-2,4-dione, [Mn(acac)$_3$] synthesized by comproportionation. [119] A wide range of compounds with substituted β-diketones *e.g.* 1-phenyl-1,3-butanedione and hexafluoroacetylacetone are known. [120-122] Some of these systems were among the first to be characterized by HF-EPR spectroscopy. [123] Derivatives of [Mn(acac)$_3$] can be prepared by ligand substitution in acid aqueous solution *e.g.* [Mn(acac)$_2$(H$_2$O)$_2$]$^+$. [124]

Ligand spheres encompassing nitrogen ligator atoms

Mn(III) complexes stabilized by nitrogen atom donor atoms are much less common than those with oxygen donors. Very few examples are known with only nitrogen donors in the coordination sphere. These encompass the [Mn(L‴)(N$_3$)$_3$] systems for L‴=terpy, bpea (mentioned above) [Mn(terpy)$_2$]$^{3+}$, [125] and a few cage complexes with saturated amine donors. [126, 127] The class of Mn(III) complexes with nitrogen donors are mainly exemplified by 2,2′-bipyridine and 1,10-phenanthroline complexes of general type, [Mn(L")(H$_2$O)X$_3$] for L"=phen, bpy and X=F, Cl. [128] A wide range of complex (adducts) of general type [Mn(L)Cl$_3$] for L=3NH$_3$, py, bpy, phen, can be prepared by reaction of the quite unstable MnCl$_3$ and the appropriate nitrogen donor ligand. [129] Similar reaction with ligands containing other group 15 donor atoms than nitrogen such as EPh$_3$ for E=P, As leads to redox reactions and formation of the Mn(II) complexes [Mn(OEPh$_3$)Cl$_2$]. [130]

Ligand spheres encompassing mixed oxygen and nitrogen ligator atoms

Systems with a mixed ligand sphere encompassing both oxygen and nitrogen ligator atoms are plentiful. These systems are primarily represented by the ubiquitous Schiff-base complexes, but other, very stable examples of such systems exist such as the quinoline-8-ol complex, [Mn(quin)$_3$]. [131]

Schiff bases and related complexes

A frequently used group of ligands with mixed nitrogen and oxygen ligator atoms are the one based on a imino (R$_1$R$_2$C=N–R$_3$) derivated backbone. These ligands are easily synthesied by nucleophilic addition of a primary amine to a carbonyl group as shown in Figure 12. The carbonyl compound can be added as either a aldehyde or ketone and with no immediate restrictions on the presence of other functional groups. Reduction of the imine (=N–) with *e.g.* NaBH$_4$ gives access to the corresponding amine (–NH–). Imines are not in general stable with respect to hydrolysis. Presence of a aryl group in conjugation with the imine bond results in relatively stable and often

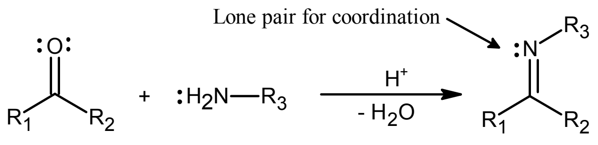

Figure 12 Imine synthesis by nucleophilic addition of a primary amine to a carbonyl

colorful compounds, normally refered to as “Schiff bases”. This name honors Schiff who was the first to describe imin condensation products of salicylaldehyde and different amines in 1869. [132]

The imine nitrogen lone pair is capable to function as a donor group towards metals, see Figure 12. This in combination with the ease with which Schiff bases can be formed and the variety of different systems which may be obtained has led to an immense popularity of Schiff bases as ligands in quite diverse applications. One of the most used Schiff-base ligands in coordination chemistry and in special in relation to the chemistry of Mn(III), is the tetradentate and divalent imino ligand, *N,N'*-ethylenebis(salicylideneimine) (abbreviated H_2salen or as is often will be used in the following H_2salen-5H) obtained by condensation of salicylaldehyde (2-hydroxybenzaldehyde) with ethane-1,2-diamine. Due to the relative disposition of the imine nitrogens and the hydroxyl groups leading to six-membered rings upon metal coordination chalation is highly favored for this ligand and related systems. H_2salen is first described by Mason in his work from 1887 on condensation products of ethane-1,2-diamine and a number of different aromatic aldehydes (*e.g.* salicylic aldehyde and benzaldehyde). [133] Complexes of the H_2salen ligand, as known today were synthesized and characterized by Pfeiffer *et al.* starting in 1933. [134, 135] The ability of the salen ligand to stabilize dicationic metal centers (also for higher oxidation states) was early recognized in reactions of M(II) for M=Co, Ni, Cu, Zn, Cd, as well as VO^{2+}, UO_2^{2+} with the ligand. [136, 137] The more redox-active metal centers as Mn, Fe and in some cases Co gave, in contrast to more inactive redox centers, not formation of $[M^{II}$(salen)], but in oxidized systems such as $[Fe^{III}$(salen)$]_2$O and $[Mn^{III}$(OH)(salen)]. The divalent complexes, $[Mn^{II}$(SB)] for SB=salen, salpn, salphen and $[Fe^{II}$(salen)] were later prepared in connection with investigation of their magnetic properties and interaction with oxygen, as moisture sensitive but air stable orange-red crystals. [138-140] Rational synthesis of simple Fe(III) Schiff-base complexes is exemplified 1938 by Thielert *et al.* with $[Fe^{III}$(Cl)(salen)] and $[Fe^{III}$(Cl)(saloph)]. [141] In the following the focus will be placed on the Schiff-base complexes of Mn(III). To date the most authoritative review within this area is from 2007 by Miyasaka *et al.* [142] In this paper a quite encompassing introduction to the area is given with special emphasis on polymers of variable types and their structural and magnetic. Reviews dealing with complexes of other transition metals are also available. [137]

Applications Schiff-base complexes of Mn(III)

In the following a brief description is given of some of the discoveries, which have contributed to the central position of Mn(III)-salen systems in contemporary manganese chemistry. The discoveries to be touched upon will encompass catalytic properties in organic and biological chemistry and the magnetic properties and applicability as building blocks for single molecule magnets.

Catalytic behaviour

The application of Schiff base complexes of Mn(III), [Mn^{III}(X)(SB)] in organic chemistry is mainly exemplified by the work of Jacobsen *et al.* who studied the catalytic behaviour of these systems in enataenantioselective epoxidation of alkenes as illustrated in Figure 13.

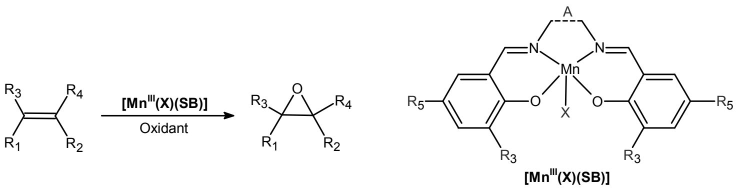

Figure 13 Enantioselective epoxidation of alkenes catalysed by [Mn^{III}(X)(salen)]

The markings, R_3, R_5 and A on the structure formula of [Mn(X)(SB)] shows the positions where substitution are usually made.

Frequently, hypochlorite, OCl^- has been employed as oxidant or, if anhydrous media were required, *m*-chlorperbenzoic acid or *N*-methylmorpholine *N*-oxide. [143-145]

The selection of mono-, di- and tri-substituted alkenes being catalytic epoxidated in high enantiomeric excess of the Mn(III) system is broad and can be both cyclic and acyclic. [144, 146] The ligand and the catalyst as a group can be modified by substitution on both the aromatic ring system and the diamine backbone. This allows changes in ligand conformation *e.g.* by use of sterically demanding substituents such as the ^{t}Bu group, but also the electronic properties can be by optimized by introduction of various electron accepting or donating substituents. Overall, this allows a tailor-made ligand and specific selectivity for a given reaction type or even substrate. A possible mechanism for epoxidation postulates the formation of an oxo species, [Mn(O)(salen)] which reacts with the alkene by a “side-on approach”. [144] This simple model explains to some extent the difference in reaction rate of epoxidation of *trans* versus *cis* isomeric alkenes, due to the expected non-favourable steric interaction between the ligand plane spanned by the [Mn(salen)] moiety and the substituents in *trans* position. Nevertheless, several works has shown that tri-substituted alkens as 1-phenylcyclohexene can be catalytic epoxidated in high enantiomeric excess using the normal protocol. [146]

Salen complexes similar to those described for Mn(III), but with other (transition) metals in oxidation state +3 as central ion has been studied for catalytic activity. A number of metal ions shows to be catalytically active. Frequently, this is utilized for stereoselective catalysis employing a salen-derived ligand based on the chiral 1.2-diaminocyclohexane as backbone, [M^{III}(X)(salcy-3,5^{t}Bu)]. The Cr(III) complex of this ligand exhibits catalytic activity regarding enantioselective allylic C–H oxidation, [147] as well as enantioselective alkylation of acylic α,α-disubtituted tributyltin enolates, [148] while the analogous Co(III) complex is capable of catalyzing intramolecular opening of oxetanes, [149]. Finally are enantioselective conjugated

addition of cyanide to α,β-unsaturated carbonyl compounds catalyzed with a second-order kinetic by the Al(III) complex. [150] All these systems are subjects to investigations of the mechanistic aspects of the catalytic reactions and optimization studies with regard to catalytic efficiency. The catalytic performance is probably an interplay of several factors including the nature of the axial ligand on the metal centre. This kind of influence is seen in the enantiomeric excess in case of alkylation of non-cyclic tributyltin enolates with Cr(III) highly depending on the axial ligand on the metal increasing from Cl to I, whereas the opposite trend is observed for cyclic enolates. [148]
Schiff-base complexes containing of transition metals in oxidation step $\neq$ 3 with potential catalytic activity includes among others Zr(IV), [151] and Cu(II), [152]. The latter system, which can catalyze allylic oxidation of cyclohexene to 2-cyclohexen-1-ol and 2-cyclohexen-one with oxygen as oxidant, contains a peculiar charged ligand based on a acetylacetone-ethylendiimine skeleton with pyridinium as substituent in 3-position.

Biological systems and medicine
The chemistry of manganese in biological systems been reviewed numerous times. [153] Manganese is widely found in natural occurring enzymes, especially in systems that require redox activity. Among the most important are the oxygen evolving complex photo system II and the group of enzymes scavenging reactive oxygen species (ROS). The important role of manganese in Photosynthesis will not be subject to further considerations but references to a series of articles in *Coordination Chemistry Reviews* are given. [154-163]

The group of reactive oxygen species is formed *in vitro* by oxygen contaning radicals, hydrogen peroxide, H_2O_2 superoxide, O_2^- and other species produced as by-products of the metabolism. Numerous enzymatic systems are found in nature to protect the cell against oxidative and eventually fatal damage from O_2^- (*e.g.* Cu/Zn, Mn, Fe and Ni superoxide dismutase) and H_2O_2 (*e.g.* Catalase, Chloroperoxidase, glutathione peroxidase, [164] and Cytochrome c peroxidase). The degradation sequence of the reactive oxygen species is a simple reduction first from O_2^- to H_2O_2 catalyzed by superoxide dismutase and then a disproportionation of H_2O_2 catalyzed by catalase. [165]
Several of the described systems can be found in other versions with different redox-active metal centre in the catalytic active site. If the organism for some reason experiences a low level of scavengers against the reactive oxygen species, a state of oxidative stress occurs. Several compounds has been attempted and studied to remedy against the potentially fatal state of oxidative stress *e.g.* the glutathione peroxidase mimic 2-phenyl-1,2-benzisoselenazol-3(2*H*)-one. The well-known catalytic and oxidative properties of [Mn^{III}(X)(salen)] exhibit some similarities with the naturally occurring oxidative protective systems. As previously mentioned, these systems are well defined, stable and yet with a relatively high valent metal centre. This has given rise to a considerable research in the intersection area of chemistry and medicine about

use of the Mn(III)-salen systems as scavengers of reactive oxygen species and eventually superoxide dismutase/catalase mimetics. Experiment show that Mn(III)-salen complexes in general exhibits superoxide dismutase activity but to a varying degree. [166] Most promising seem the simple parent molecules [Mn(Cl)(salen)] and [Mn(Cl)(salen-3MeO)] both having superoxide dismutase activity while catalase and cytoprotective activities is somewhat higher in the [Mn(Cl)(salen-3MeO)]. [167, 168] Interestingly, it has been shown that the superoxide dismutase activity in all these systems is independent of the substitution pattern of the salen-ligand whereas catalase activity is substitution pattern dependent. [164]

The activities of the Mn(III)-salen systems regarding apoptosis and anti-tumour activities has been investigated in severals works. [169] The superoxids dismutase/catalase activity among other things originating from the redox active $[Mn^{III}(salen)]^{+}$ can meridiate specific cleavage and can be strangely damaging towards free DNA especially in combination with an oxidant (hydrogen perxoide, iodosyl benzene and others). [170] Treatment in combination with glutathione and α-Lipoic acid has shown to reduce some of the harmful effects on DNA. [171]

JAHN-TELLER EFFECTS IN Mn(III) COMPLEXES

A central concept in understanding the often unique structural, spectroscopic and magnetic properties exhibited by Mn(III) in formally octahedral complexes is the Jahn-Teller effect. In the following, a description of the Jahn-Teller effect will be provided with respect to its:

- Structural manifestations
- Magnetic manifestations

This division is somewhat artificial due to the fact both manifestations often occurs at the same time and in combination. However, with respect to the structural manifestation of the Jahn-Teller effect it is possible to make a more phenomenological description whereas the magnetic manifestations requires a more rigid electronic structure model apparatus for a proper description. Though, a certain overlap between the two descriptions must be expected.
The qualitative introduction to the Jahn-Teller effect will be used in later sections for description and analysis of properties of the new Mn(III)-Schiff-base complexes, *catena*-[Mn(μ-F)(salen-5R)].The magnetic manifestations will be of importance in the ensuing discussion of the magnetic coupling in the *catena*-[Mn(μ-F)(salen-5R)] systems and in the discussion of the superhyperfine couplings observed in monomeric fluorido complexes: [Mn(F)(salen-5R)], *trans*-$[MnF_2(salen\text{-}5H)]^{-}$ and MnF_6^{3-}.

Structural manifestation of the Jahn-Teller effect

On a purely phenomenological basis a manifestation of the Jahn-Teller effect can often be identified directly in the structures of Mn(III) complexes. As an example, consider the series of fluoridomanganate(III) ions of type $[MnF_n]^{3-n}$ for n=3-6. Structure determination by single crystal X-ray crystallography of the fluoridomanganate(III) ions shows that all of the complexes consists of 3D structure of corner and edge sharing $[MnF_6]^{3-}$ "octahedra" arranged in rows or networks. This ion adopts in general a pseudo octahedral coordination geometry of the six fluorido ligands surrounding the central Mn(III). Bond lengths of the axial and equatorial ligands in a selection of fluoridomanganate(III) complexes are set out in Table 2. Based on these data is clearly seen that the six Mn–F bond lengths of the distorted octahedra fall into two distinct sets. One set of values around 1.8 Å and another set of larger bond lengths around 2.0 Å. Compare with Figure 14. A classic example of Jahn-Teller effects in Mn(III) complexes is provided by solid MnF_3. [172-175]

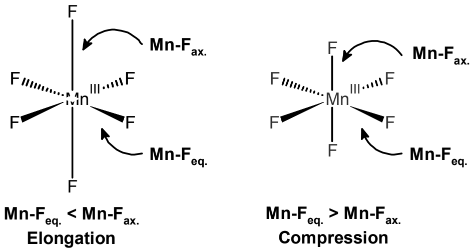

Figure 14 Tetragonal Jahn-Teller distortion of $[MnF_6]^{3-}$ octahedra

The here described bonding situation between the axial and equatorial bond lengths should be seen in relation to what is observed in systems as in the corresponding Mn(II) fluorides such as MnF_2, which crystallizes with the Rutile-structure.

		Mn–F$_{eq,1}$ (Å)	Mn–F$_{eq,2}$ (Å)	Mn–F$_{ax}$ (Å)	∠Mn–F–Mn (°)	Ref.
n=3	MnF_3	1.79	1.91	2.09		[178]
n=4	$K[MnF_4]\cdot H_2O$	1.79, 1.87	1.88	2.09, 1.98	135.1	[179]
	$Rb[MnF_4]\cdot H_2O$	1.84, 1.83	1.85	2.12, 1.96	137.7	[179]
n=5	$Li_2[MnF_5]$	1.84	1.85	2.12	121.5	[180]
	$Na_2[MnF_5]$	1.83, 1.84	1.87, 1.85	2.10, 2.11	132.5	[181]
	$(NH_4)_2[MnF_5]$	1,84	1.84	2.09	143.4	[182]
	$K_2[MnF_5]\cdot H_2O$	1.82	1.84	2.07	163.3	[183]
	$Rb_2[MnF_5]\cdot H_2O$	1.84	1.86	2.09	175.4	[184]
n=6	$NaK_2[MnF_6]$	1.86	—	2.06	—	[94]
	$Na_3[MnF_6]$	1.86	1.90	2.02	—	[185]

Table 2 Bonding parameters for Fluoridomanganate(III) complexes

In the case of n=4,5 the data given are from [186]. More complexes as well as additional data for each individual are also given in [186]. References given in the table allocate to the original literature.

The geometry of the coordination polyhedra is a regular octahedra with identical Mn(II)–F bond lengths, (~2.12 Å). [176, 177]

An overview of the different geometry relevant for systems subject to Jahn-Teller distortion and their relation to each other is given in Figure 15. The electronic configuration of a high spin Mn(III) ion in a cubic ligand field is $t_{2e}^3e_g^1$. Only one of the *d*-orbitals, d_{x2-y2}, d_{z2} in the degenerate e_σ set is occupied. On a purely electrostatic basis, the electrons in the occupied orbital and the ligands that are directed against the orbital are repelled. This gives the occupied orbital a higher energy than the non-occupied, and gives rise to an elongation of the bond. Based on this extremely simple analysis, it is expected that the elongation of the axial ligands are associated with elimination of orbital degeneration if the single electron of the e_g set resides in the d_{z2} orbital.
The bonding situation described for Mn(III) is also found in the isolelectronic Cr(II) as well as in the $3d^9$ system of Cu(II), which is related to the d^4 systems by having and additional, spherically-symmetric half-filled *d*-shell of electrons. Complexes of Cu(II) which traditionally have been, and still are, very rich in number represent the classic examples of Jahn-Teller systems - and constitute a varied research field. [187-196] The Jahn-Teller effect is often significant which may for example be seen in the hexaaqua ion, $[Cu(H_2O)_6]^{2+}$ which in the Tutton salt, $M^I_2[Cu(H_2O)_6](SO_4)_2$ for M= NH_4, Cs holds two axial bonds of 2.28 Å (NH_4) and 2.31 Å (Cs) compared with four equatorial bonds of 1.97 Å/2.00 Å (NH_4) and 1.97 Å/2.00 Å (Cs). [197, 198] The counter ions seems to have some effect on elongation by comparing the above Tutton salts with *e.g.* $(NH_4)_2[Cu^{II}(H_2O)_6][Cu^ISO_3]_4$ with axial and equatorial bond lengths of 2.33 Å and 1.99 Å respectively. [199]
This considerable Jahn-Teller effect in Cu(II) systems has direct influence on the reactivity of in respect of *e.g.* ligand substitution of the aqua ligands in $[Cu(H_2O)_6]^{2+}$ with NH_3. Only the equatorial aqua ligands is substituted in $NH_3(aq)$ as reported by Bjerrum, in his celebrated dissertation from 1940 (translation, 1957), [200] and as seen in the synthesis of $[Cu(NH_3)_4]SO_4{\cdot}H_2O$. [111] On the other hand, the Jahn-Teller elongated axial requires forcing conditions with $NH_3(l)$ to be occupied by ammonia ligands. [201-203] Similar reactivity is seen for ligand substitution with bidentate ligand were the ideal bite angle of the ligand and Jahn-Teller distortion may be in conflict. Elongation in Cu(II) complexes can be so pronounced that the two axial ligands are completely detached from the central ion giving a square plane square geometry *e.g.* in CuO, $[CuCl_4]^{2-}$. [204]

The overwhelming majority of structurally characterized Mn(III) complexes are described as being subject to Jahn-Teller distortion. This is exemplified by the Mn(III)-Schiff-base complexes, which without exception show Jahn-Teller distortion as seen from Table 3.
However it should be noted that systems, which theoretically are subject to Jahn-Teller distortions, but in practice exhibit close to regular symmetry due to other opposing physical processes are known. This is *e.g.* seen in the alum $[Cs(H_2O)_6][Mn(H_2O)_6]SO_4$. [205] Another

classicexample is provided by [Mn(acac)$_3$] (coordination polyhedron with a deviation of only 0.03Å from the regular octahedra). [206] However later reports in the literature points out possible errors in the structure solution and finds Jahn-Teller distortion of normal magnitudes. [207] It must also be remembered that of distortions from regular symmetry may not necessarily be manifestations of Jahn-Teller effects, but may originate in other physical phenomena *e.g.* crystal packing.
The discussion of Jahn-Teller effects is generally divided in static and dynamic effects. The examples given on the structural manifestations of the Jahn-Teller effect has all been static, which means that the complex independent of external conditions is permanently present in one configuration of the tetragonal, D_{4h} symmetry. On the other hand, dynamic Jahn-Teller effect observed if the complex undergoes a dynamic equilibrium between a elongated/compressed tetragonality along different axes. Depending on the oscillation rate between the directions of elongation, an actual distorted complex, (D_{4h} symmetry) may appear non-distorted, (O_h symmetry).

Magnetic manifestation of the Jahn-Teller effect
The reason for the effect known as Jahn-Teller distortions is to be sought in the ionic electronic structure. As mentioned above, the electron configuration of Mn(III) is high-spin $3d^4$ (S = 2) with a ^5D free ion ground term. Depending on the symmetry of the ligand field the free ion ground term are split in various ways. In case of a weak octahedral, O_h ligand-field this free ion ground term split into a ^5E_g ground-term and a excited $^5T_{2g}$ term. By lowering the symmetry of the ligand-field from O_h to tetragonal, D_{4h} symmetry the two double and triple degenerated terms is lifted into $^5A_{1g} \oplus {}^5B_{1g}$ and $^5B_{1g} \oplus {}^5E_g$. [208] This symmetry lowering in the form of an axial elongation with $^5B_{1g}$ as ground state or compression with the reversal of the order of $^5B_{1g}$ and $^5A_{1g}$ is observed in connection with the Jahn-Teller distortion, *cf.* the structures of fluoridomanganate(III) ions in Table 2. The electronic structure consequences of the Jahn-Teller effect(s) are summarized in Figure 15.

The decrease in symmetry upon change from octahedral to tetragonal symmetry by axial bond elongation or compression results in a breaking of the orbital degeneracies which form the basis of a theorem by Jahn and Teller proven by use of complicated group theoretical considerations in 1937. Alternative and relative more simple proof for the theorem has however, been given susequently. [209] The Jahn-Teller theorem can be expressed as:

> *A nonlinear molecule cannot be stable in a degenerate electronic ground state and the molecule must become distorted in such a way that the degeneracy is broken.* [210, 211]

Two key questions are not answered by the Jahn-Teller theorem in the form given, namely, why and how the effect occurs.

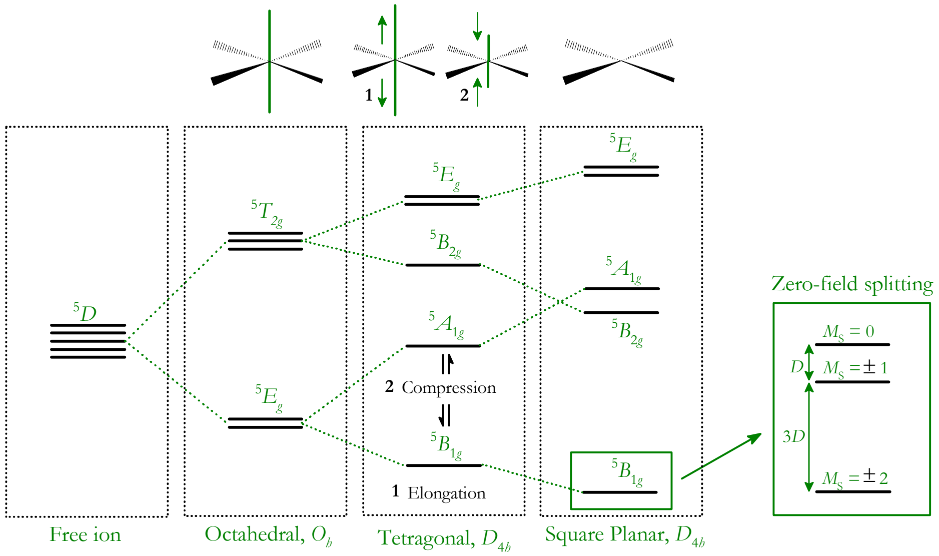

Figure 15 Energy level diagram for Mn(III) in weak Octahedral and Tetragonal ligand field.

Molecular distortions according to the Jahn-Teller theorem are in principal allowed for all electronic configurations except d^3, high spin d^5, low spin d^6 and d^8 all of which possess a non-degenerated ground states ($^4A_{2g}$, $^6A_{1g}$, $^1A_{1g}$ and $^3A_{2g}$). Jahn-Teller behaviour is seen in system with E_g ground-term and ^5E_g term undergoes a very large splitting to leave an orbital singlet ground state. The principal Jahn-Teller ions are: d^4: Cr(II), Mn(III) (high-spin, $t_{2e}^3e_g^1$), d^7: Co(II), low-spin, Ni(III) ($t_{2e}^6e_g^1$), low-spin and d^9: Cu(II) ($t_{2e}^6e_g^3$). Of these, only the first three ions are relevant since Ni(III) is in practice only known in very few complexes as the fluorido complex, $[NiF_6]^{3-}$ (stabilized according to the HSAB principle). [212]

Even without a applied magnetic induction, low-symmetry ligand field effects in conjunction with spin-orbit coupling will give rise to Zero-field splitting in form of different energies of the various M_S states. Due to the Zero-field splitting and the resulting lifted degeneracy of the spin, the ground state of either the tetragonal elongated $^5B_{1g}$ or compressed system $^5A_{1g}$ d^4-system consists therefore of three states. The energy difference between the various states can be expressed by an integer coefficient to the D parameter, which may be of both positive or negative. The energy difference between the states of highest and lowest M_S is generally:

$$\left|E\left(M_{S,Max.}\right)-E\left(M_{S,Min.}\right)\right|=|D|S^2 \quad (4.2)$$

The typical range of the Zero-field splitting is approx. $|D| \leq 2\ cm^{-1}$.

The ground state and the degeneration of the excited states depends in general on whether the system holds an even or odd number of unpaired electrons. If the total spin is integer, S = 1,2,3,… then the system holds an even number of unpaired electrons and the ground state is non-degenerate with M_S = 0 (for positive values of D) and all excited states are doubly degenerate ($M_S = \pm S$, $\pm(S{-}1)$,…, ±1). On the other side if the total spin is half-integers, S = 1/2, 3/2, 5/2,… then the system holds an odd number of unpaired electrons which results in the degenerate M_S = ±1/2 Kramer's doublet as ground state (again for positive values of D). The excited states will similarly be double degenerate (M_S = ±3/2, ±5/2,…).

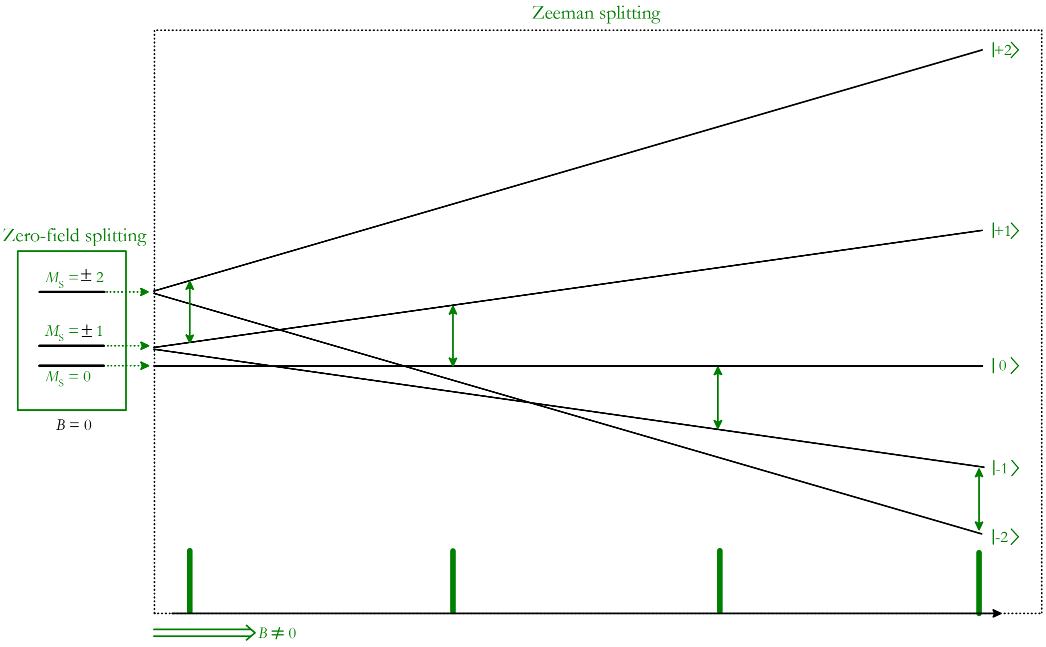

Figure 16 Zeeman splitting of Mn(III)

The zero-field splitting depends on various factors such as Jahn-Teller effects and spin-orbit coupling. An important characteristic of Mn(III)-salen systems are the coincidence between preferred orientation of the magnetic spin (easy axis) and their Jahn-Teller axis.

Phenomenologically the removal of degeneracies of spin-states is usually described in the socalled Spin-Hamiltonian formalism (Chapter 3). For systems with S > 1/2 there are terms in the Spin-Hamiltonian that will be non-zero when the symmetry of the molecule is lower than octahedral. These terms are associated with axial (D) and rhombic (E) Spin-Hamiltonian

parameters *cf.* (4.3). The magnitude of the zero-field splitting depends on various factors such as ligand field deviations from an octahedral field (Jahn-Teller effects) and spin-orbit coupling.

$$\hat{H}_{\mathrm{ZFS}} = D\left[\hat{S}_z^{\,2} - \tfrac{1}{3}\hat{S}\left(\hat{S}+1\right)\right] + E\left(\hat{S}_x^{\,2} - \hat{S}_y^{\,2}\right) \tag{4.3}$$

Most single-molecule magnets (SMM) including the celebrated Mn_{12} are based on Mn(III) due to the fairly large spin and due to the sizable zero field splittings of this centre brought about by the Jahn-Teller effects. An important characteristic of Mn(III)-salen systems is the coincidence between preferred orientation of the magnetic spin (easy axis) and their Jahn-Teller axis. This property facilitates the rational design of polynuclear systems with large magnetic couplings.

SYNTHETIC STRATEGY FOR 3*d*-(μF)-3*d* BRIDGED SYSTEMS

General methods for the synthesis of [Mn(X)(salen)] and [Mn(X)(SB)]

The synthetic strategy described in the literature applicable to the synthesis of species of general type [Mn^{III}(X)(salen)] is in principle based on an appropriate Mn(II) species, $Mn^{II}Y_2$ which through a two-step sequence consisting of, respectively, an oxidation of Mn(II) to Mn(III) and a ligand substitution of Y with X gives the desired product. As shown in Figure 17 the synthesis of [Mn^{III}(X)(salen)] can be performed in several ways, either as a complete reaction or divided into a number of separate steps. In the description of the methods for synthesizing [Mn^{III}(X)(salen)] it is convenient to consider the sequence of reactions with two different mono-anionic ligands. On one side the ligand X desired in the product species, and on the other the ligand, Y beneficial under the reaction conditions until a final ligand substitution with X can be made.

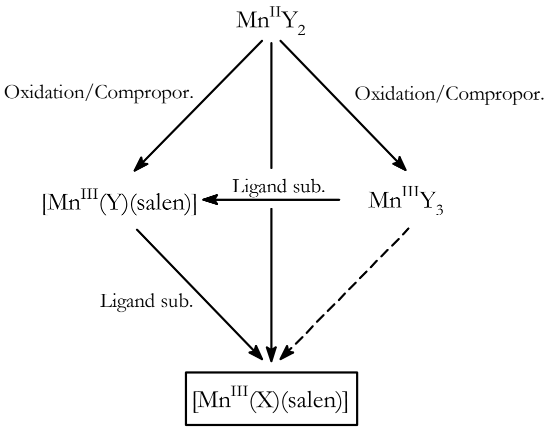

Figure 17 Synthesis strategies of [Mn(X)(salen)]

Despite the fact that the sacrificial ligand is described as an abstraction,the overwhelming majority of the literature makes use of acetate (OAc^-) for this purpose. The different reaction pathways used in literature will be reviewed in the following. An overall picture of the reaction steps and their interrelationship is given in Figure 17. Based on the methods which appear in the literature for incorporation of Mn(III) in the salen based system, it is advantageous to divide the reactions according to the oxidant used and classify them as either, comproportionation or oxidation by air. In both classes of the reaction, a suitable Mn(II) precursor is target of oxidation. Depending on the final product compatibility with the reaction mixture during the oxidation, the generated Mn(III) can either be used *in situ* or isolated as [Mn^{III}(Y)(salen)] or $Mn^{III}Y_3$. The following sections will address some aspects of these reactions based on a number of examples from the literature and drawing upon on the general features of Mn(III) chemistry discussed above.

1. Method: MnY_3 as precursor

The $Mn^{III}Y_3$ species can in principle be obtained by either oxidation of a $Mn^{II}Y_2$ species or reduction of a suitable high valent manganese species, often Mn(VII). These redox-reaction can be difficult to control with respect to to yield the desired oxidation state of the product and therefore, the synthesis of the simple $Mn^{III}Y_3$ compounds is most advantageously made by a comproportionation between Mn(II) and a higher oxidation state. In general terms this reaction can be expressed as following reaction equation.

$$(n-3)\ MnY_2\ +\ Mn^{n+} + n\ HY \longrightarrow (n-2)\ MnY_3 + n\ H^+ \qquad (4.4)$$

Due to the oxidizing properties of Mn(III), the range of the appropriate $Mn^{III}Y_3$ species as precursors for synthesis of Mn(III)-salen complexes is limited by the requirement of suitable coordination sphere of stabilizing ligands often oxygen ligators *e.g.* Schiff bases or β-diketones. The number of Mn(III) precursors with simple ligand/counter ions is in practice limited to the two acetates $Mn(OAc)_3{\cdot}2H_2O$ and $Mn(OAc)_3$, the fluoride MnF_3 and the acetylacetonates as $Mn(acac)_3$ and $[Mn(X)(acac)_2]$. As mentioned above are other simple Mn(III) species as the hygroscopic sulphate $Mn_2(SO_4)_3$, caesium alum $CsMn(SO_4)_2{\cdot}12H_2O$ and chlorido complex $K_2[MnCl_5]$, [213] well known, but none of these has so far found usage for synthesis of Mn(III)-salen complexes. The general comproportionation (4.4) finds its most frequent use in connection with synthesis of the aqueous acetate, $Mn(OAc)_3{\cdot}2H_2O$ by the method of Christensen, as well as for preparation of the acetylacetonato complexes, $[Mn(X)(acac)_2]$ ($X=N_3$, NCS) starting from $Mn^{II}(OAc)_2{\cdot}4H_2O$ (or $MnSO_4{\cdot}4H_2O$) and $KMnO_4$ with a large excess of acetylacetone. [214] Among the first work on the synthesis of complexes of the general type, [Mn(X)(salen)] is the one by Prabhakaran and Patel from 1969. [215] This work establishes the now classic method where a precusor $Mn^{III}Y_3$ is reacted in presens of H_2salen (or another related Schiff-base ligand) and the incoming ligand, X.

$$MnY_3 + H_2salen + X^- \longrightarrow [Mn(X)(salen)] + 2\ HY + Y^- \qquad (4.5)$$

The acetate, $Mn(OAc)_3$ is predominantly used as a precursor source of Mn(III) like the incoming ligand generally entering the system as either Li(I) or Na(I) salt. A wide range of compounds, $[Mn(X)(SB)]{\cdot}nH_2O$ has been synthesized by this approach. Examples of the variations of these products includes: Aqua-complexes $[Mn(H_2O)(SB)]ClO_4$ were SB=H_2salen-5F, H_2salen-MeO, H_2salophen. [216] Halogenido-complexes for X=Cl, Br, I of the Schiff-bases H_2salen, [215] and H_2salpn, [217]. Azido complexes which, depending on the Schiff-base ligand can be both monomeric and polymeric in the form of an infinite 1D-chain structures. The two bonding situations are seen respectively in $[Mn(N_3)(SB)]$ were SB=H_2salen-3,5^{t}Bu, H_2salphen-3,5^{t}Bu, [218] and *catena*-$[Mn(\mu_{1,3}\text{-}N_3)(SB)]$ were SB=H_2salen,5F, H_2salen-5MeO, H_2salophen, [216]. Similar reactions can be used for synthesis of the acetylacetonato complexes $[Mn(X)(acac)_2]$ for $X=N_3$, NCS. [214]

2. Method: [Mn(Y)(salen)] as precursor

Oxidation of Mn(II) to Mn(III) by presence of the H_2salen ligand or other Schiff-base ligand can be achieved by arial oxidation in hot methanol as shown in (4.6).

$$4\ MnY_2 + 4\ H_2salen + O_2 \xrightarrow[\Delta]{MeOH} 4\ [Mn(Y)(salen)] + 4\ HY + 2\ H_2O \qquad (4.6)$$

Other oxidizing agents such as NO constitute a less common as alternative to oxygen. [139] Although reaction (4.6) is given on general form, it is in the vast majority of cases only applied for synthesis of the acetato or chlorido complexes of H_2salen, *e.g.* [Mn(X)(salen)], X=OAc, [144, 217, 219], X=Cl, Cl(H_2O), [220, 221] sometimes directly combined with reaction (4.7). Nevertheless, it has been demonstrated that the reaction can be used for synthesis of a variety of other complexes – covering variations of the axial ligand as well as of the Schiff-base ligand. Examples of simple complexes includes the [Mn(Cl)(H_2O)(SB)] and [Mn(H_2O)(SB)]ClO_4 were SB= H_2salpn-5Br, H_2salmen-5Br and others. [222] The perchlorate complex exist besides in the monomeric form also as a dimer [Mn(H_2O)(salen)]$_2$(ClO_4)$_2$. [223]

The reaction (4.6) occurs under realitively mild conditions and it is therefore sometimes possible to convert the initially formed [Mn^{III}(Y)(salen)] by ligand substitution with X to the final product, [Mn^{III}(X)(salen)] without previous isolation.

$$4\ MnY_2 + 4\ H_2salen + 4\ X^- + O_2 \xrightarrow[\Delta]{MeOH} 4\ [Mn(X)(salen)] + 4\ HY + 4\ Y^- + 2\ H_2O \qquad (4.7)$$

The incoming axial ligand, X is in general added to the reaction mixture in the form of an alkali metal salt. This direct reaction method was made popular by the work of Jacobsen *et al.* in connection with the work on $[Mn(salen)]^+$-catalyzed asymmetric olefin epoxidation. [143, 164, 224] The method is preferred especially for the synthesis of complexes with simple axial ligands such as acetate, [217], halogenide, [143, 219] or [Mn(X)(H_2O)$_n$(SB)] were X=F, Br, I, NCS (n=0), N_3 (n=0) and SB=H_2salen-5Br, H_2salmen-5Br. In all casesl starting with Mn(ClO_4)$_2$·6H_2O as Mn(II) precusor. [222]

Also polymeric complexes has been synthesized by this method. These polymers are often 1D infinite chains of mononuclear building blocks or dimeric entities. A chain of mononuclear units is found in case of the nitrato complex, *catena*-[Mn(NO_3)(salen)]. [223] Both types of systems are found for the cyanamide complexes *catena*-[Mn($\mu_{1,3}$-NCNH)(salen-5R)] were R=Cl, Br and [Mn_2($\mu_{1,3}$-NCNH)(salen-5R)$_2$]ClO_4·MeOH were R=H, F, Cl, OMe. [225] Similar behavior is seen using NCS^- as ligand, which yield both *catena*-[Mn($\mu_{1,3}$-NCS)(salpn)] and [Mn(NCS)(salpn)]$_2$. [226]

3. Method: Ligand substitution

A number of conditions must be fulfilled for a successfully completed reaction according to (4.7) besides the mild reaction conditions. In order to achieve a pure product from the multi-component reaction mixture, it is necessary that the incoming ligand, X compared with other potential ligating species in the mixture results in either thermodynamic stability or a kinetically favorable route for product isolation. In the latter context, a relatively low solubility of the product [Mn(X)(salen)] is particular important. If the competition between the incomming ligand, X and other species in the reaction mixture is of such a character that formation of the desired product [Mn(X)(salen)] is prevented, then the intermediate product, $[Mn^{III}(Y)(salen)]$, must be obtained according to (4.6) or (4.5) and isolated before ligand substitution is conducted.

$$[Mn(Y)(salen)] + X^{-} \longrightarrow [Mn(X)(salen)] + Y^{-} \qquad (4.8)$$

Several examples of this procedure have been described in the literature. The bromido complex [Mn(Br)(salen)] complex is "intermediate" in stability, which means that it can be synthesized and isolated in pure form by the normal procedure similar to (4.6) and then used in ligand substitution of the bromido ligand by (4.8). One example of a complex obtained by this method is the nitro complex, $[Mn(NO_2)(salen)]$. [227] Another widely used precursor complex used for this type of reaction is the aqua complex, $[Mn(H_2O)(SB)]ClO_4$ which can be made with af wide range of Schiff-bases *e.g.* H_2salen-5R for R=H [228], F, OMe [216], Cl, Br [229] as well as H_2salpn [230] and H_2salphen [216]. The water ligand can be replaced by a number of other ligands and aquo complexes have been used for the synthesis of the structural very different azido complexes *catena*-$[Mn(\mu_{1,3}\text{-}N_3)$(salen-5Br)] and $[Mn(N_3)(salen\text{-}5Cl)]_2$, [229] but also the mixed $S = 1$ and $S = 2$ cyanido complex, *catena*-[Mn(CN)(salen)]. [228]

Ligand substitution of the acetato ligand in the classic [Mn(OAc)(SB)] according to (4.8) is used for incorporation of among other X=F, Cl, Br, I, NCS, CN in case of H_2salen, [217] and H_2salen-4(^{sec}Bu), [231]. The synthesis of the polymeric azido, *catena*-$[Mn(\mu_{1,3}\text{-}N_3)$(salen)] and dicyanoargentato species, *catena*-$[Mn(Ag(CN)_2)$(salen)]. [232] Ligand substitutions where the desired product is not directly farvored can be performed *e.g.* by precipitating the outgoing ligand as a sparingly soluble salt before addition of the incoming ligand. This principle can be illustrated by synthesis of the analogous acetylacetonato complexes $[Mn(X)(acac)_2]$ for X=Cl, Br produced by treatment of $[Mn(SCN)(acac)_2]$ with Ag(I). [214]

Other methods

A kind of ligand substitution, not directly illustrated in Figure 17 is the one where the but the salen ligand replaces a different polydentate ligand. That reaction type is rare and only mentioned a very limited number of times in the literature. This is due to the scarcity useful, stable systems of composition $[Mn^{III}(X)(L)_2]$ with substitutable bidentate ligands. However, the reaction type has found usage in in synthesis of the complexes [Mn(X)(salen)] for X=N_3, SCN by reaction of the precursors $[Mn(X)(acac)_2]$ with H_2salen in dmf/EtOH. [227]

Conclusion

Despite the fact that the synthesis of [Mn(X)(salen)] complexes in many ways can be rationalized and made by one of the methods described, it should be noted that the synthesis in some cases might be problematic in sense of reproducibility and product purity. Intricate factors such as solvent purity and reaction conditions can affect the quality and yield of the product to a significant extent.

Development of a new method for synthesis of *catena*-[Mn(μ-F)(salen-5R)]

Although synthesis, characterization and properties investigation of [Mn(X)(salen)] complexes with a wide range of both Schiff base ligands and axial ligands, X has been the subject of a large number of studies fluorido-containing complexes have received minimal attention.

The only previous mantion of a mixed salen-fluorido complex of Mn(III) in the litterature is [Mn(F)(salen)]·H_2O reported by Ashmawy *et al.* in 1985. Its purported synthesis followed the previously mentioned general method of ligand substitution between stoichiometric amounts of [Mn(OAc)(salen)] and NaF in EtOH. [217] Additionally, fluorido complexes of the substituted Schiff-bases, H_2salen-5Br, H_2salmen-Br, [222] and H_2salen-4(^{sec}Bu), [231] have also been claimed in the form of the aquated complexes [Mn(F)(H_2O)(SB)], but without structural support for the claims,

As mentioned above, the work by Ashmawy *et al.* represents the only report on the fluorido complex [Mn(F)(salen)]·H_2O. However, following the directions provided in that report, it has not been possible to reproduce the synthesis of [Mn(F)(salen)]·H_2O. The product obtained was not homogeneous and probably contained a fluorido product contaminated with significant amounts of the starting material or decomposition products. As a result of a almost identical solubility of the reactant, [Mn(OAc)(salen)] and product, [Mn(F)(salen)] purification by recrystallization is problematic. Frequently, halogenide is added during the synthesis of [Mn(X)(SB)] as lithium or sodium salts. However, the solubility of alkali metal fluorides in ethanol at room temperature has been determined for both LiF and NaF to be low, at < 0.01mol/100 mL EtOH (27°C) which may complicate the suggested synthetic protocol. [233] By analysis of the mother liquor from the published synthetic protocol evaporated to dryness, it was found to contain nearly pure [Mn(OAc)(salen)]. It must be concluded that the necessary thermodynamic or/and kinetic conditions for quantitative ligand substitution are not satisfied in the reported protocol. These problems represent a clear example of the fact that "general" synthetic methods are not always transferable to new ligand systems.

One goal of this work has been to develop a synthetic method for preparing [Mn(F)(salen-5H)]. As a starting point in pursuit of a reliable and reproducible method, the known methods for synthesis by ligand substitution of the analogous halogenido complexes were exhaustively tested and rejected. It was deemed that, in the case of the fluorido ligand it seems to be of crucial importance that the metal and ligand are bound when the complex is assembled. It, thus,

seems natural to think of the synthetic problem in the conceptual framework of the synthon approach. This, in combination with the previously described use of MnY_3 in the synthesis of $[Mn^{III}(Y)(salen)]$ complexes, brought focus on the simple binary fluoride MnF_3. Despite its moisture sensitivity MnF_3, can be handled under normal laboratory conditions and is in many ways a well-known compound, especially in the organic laboratory as a fluorinating agent. As discussed previously MnF_3 has successfully been used as starting material for synthesis of different simple fluorido complexes. [103, 105, 106] Designation of MnF_3 as "synthon" has previously been used in the literature in the meaning of "precursor", but not emphasizing the requirement for Mn–F bonding and therefore completely parallel to the meaning intended in this thesis. [234]
It was found, that the reaction between MnF_3 and H_2salen in MeOH in the presence of base, Et_3N and a small amount of water gave rise to the desired product, *catena*-[Mn(μ-F)(salen)] with high purity and yield. In addition it could be shown that the reaction with small modifications could be generalized to the synthesis of the analogous halogen substituted complexes *catena*-[Mn(μ-F)(salen-5R)] for R=F, Cl, "Br".

The synthetic method developed can be summarized in the following reaction equations:

- **Synthesis 1: R = H, F**

$$MnF_3 + H_2\text{salen-5R} + 2\ Et_3N \xrightarrow[\Delta]{\text{MeOH, }(H_2O)} \textit{cartena}\text{-}[Mn(\mu\text{-}F)(\text{salen-5R})] + 2\ [Et_3HN]F \qquad (4.9)$$

- **Synthesis 2: R = Cl, "Br"**

$$MnF_3 + H_2\text{salen-5R} \xrightarrow[\Delta]{\text{MeOH}} \textit{cartena}\text{-}[Mn(\mu\text{-}F)(\text{salen-5R})] \qquad (4.10)$$

The synthesis of *catena*-[Mn(F)(salen-5R)] for R=H, F (4.9) have been fully optimized. The optimerization process has focussed firstly on purity and secondly on yield. It has involved parameters such as reaction time, temperature, addition of base and water.
At room temperature (4.9) proceeds completely with a reaction time of approx. 2 days. With this extended reaction time a certain degree of degradation of the ligand and the product can be directly detected through the very characteristic odor of salicylaldehyde of the reaction mixture - a firm qualitative indicator (Products of the other potential secondary reactions are complexes of the bifluorido ligand (HF_2^-). While heating under reflux, the reaction time for complete conversion is approx. 30 minutes. Degradation, although to a lesser extent is also identified in this case. The imine bonds of the H_2salen ligand are sesitive towards acid catalyzed hydrolysis, which takes place even in the presence of the weak acid HF. This can be verified easily by the liberation of salicylaldehyde upon adding solid H_2salen ligand to HF in aqueous solution. However, formation of HF during the reaction is not expected to be a particular problem since

the hydrated fluoride, $MnF_3 \cdot 2H_2O$, is known to be stable with respect to hydrolysis in aqueous HF solution. Despite the common susceptibility towards hydrolysis of Mn(III)-salen complexes in the presence of acid, there are well-documented exceptions, which complicate predictions regarding stability of new species.

An important example of such an exception is the synthesis of Mn(IV) chlorido complex [Mn(Cl)$_2$(salen)]. This emerald green complex is synthesized by oxidation in concentrated hydrochloric acid of the corresponding Mn(III) species, [Mn^{III}(Cl)(salen)]. [235, 236] The reaction proceeds without noticable destruction of the salen ligand, just as the product is stable in strongly acidic solution. With respect to *catena*-[Mn(μ-F)(salen)] partial hydrolysis is observered in the presence of moisture accompanied by formation of HF. Complete hydrolysis occurs by treatment with dilute mineral acid such as 2 M sulfuric acid. In order to overcome the problems of degradation of the ligand and the product by hydrolysis, base in the form of triethylamine, Et_3N, was added to the reaction mixture. The addition of base has the further advantage, that the reaction can be carried out in standard laboratory glassware without etching of it.

Another crucial point in the development of the synthesis was the addition of water to the reaction mixture. The addition of water may appear illogical, since MnF_3 can hydrolyze by contact with moisture under non-acidic conditions with disproportionation to yield Mn(II) and MnO_2.

$$2\ MnF_3 + 2\ H_2O \longrightarrow Mn^{2+} + MnO_2 + 4\ H^+ + 6\ F^- \qquad (4.11)$$

Nevertheless, the purity of the product, *catena*-[Mn(μ-F)(salen)] is considerably improved by this modification.[5] The original idea behind the addition of water to the reaction mixture originates from the formulation of the product as a dihydrate in the previously mentioned work of Ashmawy *et al.* This suggests either co-ordination of a water ligand to the sixth coordination site on Mn(III) or the presence of water of crystallization in the structure. Addition of water to the reaction mixture at the end of reaction was thus thought to facilitate isolation of the product as di-hydrate. At end of reaction it must be assumed that complete coordination of the salen ligand to Mn(III) has occurred and that the problem of hydrolysis of MnF_3 can be disregarded.

[5] Improvement of the product purity by the addition of water to the reaction mixture can directly be seen by comparing the hydrogen, carbon and nitrogen elemental analysis data for a test study for each of the two cases. The following data are obtained for such a trial study (with addition of base, Et_3N).

- Without water addition: Elemental analysis calculated (found) (%) for $H_{14}C_{16}N_2O_2F_1Mn_1$: H 4.15 (4.10), C 56.48 (52.02), N 8.23 (7.39).

- With water addition: Elemental analysis calculated (found) (%) for $H_{14}C_{16}N_2O_2F_1Mn_1$: H 4.15 (4.01), C 56.48 (56.10), N 8.23 (8.08).

However, as indicated by (4.9) the resulting product is actually a chain polymer without the presence of neither coordinated water ligands nor crystal water. An explanation of the influence of the added water on the purity of the product is not obvious, but open for speculation. One possibility is that the presence of water ensures that an excess of unreacted MnF_3 is hydrolyzed to MnF_2 and MnO_2 according to (4.11). Another, that the solubility properties of the solvent with respect to Mn(II) by-products from the reaction and/or the hydrolysis of MnF_3 is modified so that solely the desired product precipitated when ether is added to the reaction mixture. It should be noted that the synthesis of *catena*-[Mn(μ-F)(salen-5F)] can be carried out with products of satisfactory purity without addition of water to the reaction mixture.

Synthesis of [Mn(μ-F)(salen-5R)] for R=Cl, "Br" (4.10) is performed by applying the synthesis of *catena*-[Mn(μ-F)(salen-5H)] (4.9) directly and in its most simple form, without any consideration of the optimization procedures. Despite reproducible, succesful results of this approach (4.9) the synthesis can eventually be further optimized. It should be noted that the series of compounds *catena*-[Mn(μ-F)(salen-5R)] do not behave analogously in all situations, which must be taken into account into the synthesis. Especially in terms of solubility, which is relatively high in case of R=H, F but only moderate in case of R=Cl, Br. A detailed description of the syntheses of both the new fluorido complexes of Mn(III) and the ligands can be found in the section "Experimental details" at the end of this chapter.

The products obtained by (4.9) and (4.10) have been studied with among others by single crystal X-ray diffraction, susceptibility measurements and EPR spectroscopy. The results of structure determination and magnetism measurements are subject to a detailed treatment in the sections "Structural description of *catena*-[Mn(μ-F)(salen-5R)]" and "Magnetic behaviour and EPR-spectroscopy of 1D Mn(III)" of this chapter. Single crystal X-ray diffraction shows that all the products, in the crystalline phase forms a infinite chain polymer, *catena*-[Mn(μ-F)(salen-5R)] for R=H, F, Cl. Electron Paramagnetic Resonance spectroscopy (EPR) shows that the one-dimensional polymeric products obtained by (4.9) and (4.10) are completely dissociated into monomeric units, [Mn(F)(salen-5R)] in solution. Analysis of the products obtained by re-precipitation shows that the infinite chain structure is restored.

Use of MnF_3 as synthon raises some interesting questions concerning how coordination of the ligand to the synthon is initiated and how the formation of [Mn(F)(salen-5R)] proceeds during the progress of reaction. As a starting point, it is unlikely to assume that MnF_3 is fully dissociated in solution. Coordination is likely to proceed through a stepwise process. Literature concerning mechanisms of formation of Mn(III)-salen complexes is very limited if existing. A few studies regarding kinetic issues have been published, but none of these can be transferred to the situation described here. [237-241]
Another issue that might be interesting to elucidate the is why addition of a small amount of water to the reaction mixture during the synthesis of *catena*-[Mn(μ-F)(salen-5H)] and not *e.g.*

catena-[Mn(µ-F)(salen-5F)] has a significant impact on the purity of the and if information about the reaction pathway could contribute to unravel this.

Electrospray mass spectrometry, ESP (See Appendix 2 “Instrumentarium”) was recorded in order to try to determine the speciation of MnF_3 dissolved in MeOH and the stability of such a solution with time. The mass spectra were recorded on a solution prepared by suspension of solid MnF_3 (Strem 98%) in MeOH (Sigma-Aldrich, Anhydrous 99.8%) at room temperature. After 1-2 min. the suspension was filtered through a 0.45 µm filter (Millipore, Durapore Membrane Filters) and the first spectrum was recorded (See Figure 18, Spectrum I). The remaining solution was left at room temperature for 1½ hour before the second spectrum was recorded (See Figure 18, Spectrum II).
Upon qualitative visual inspection of the spectra in Figure 18 it is found that the contents profile of the two solutions was almost the same. After the initial degradation of some unknown part of MnF_3 during the dissolution process, the solution/suspension of MnF_3 in MeOH is relatively stable over time. A quantitative assignment of the two spectra is unfortunately almost impossible due to their complex nature, which results in a remarkable complexity of the mass spectra. If compared with the molar mass of MnF_3 (111.9 g·mol^{-1}) it must be assumed that a wide variety of species are present in the solutions including polynuclear complexes and assignment of the *M/z*=465 and 496 peaks to species of composition $[Mn_3O(CH_3O)_6(CH_3OH)_{3/4}]^+$ is tempting, but not in any way revealing. Because of these complications further studies on the mechanisms of the formation of [Mn(F)(salen-5R)] were abandoned.

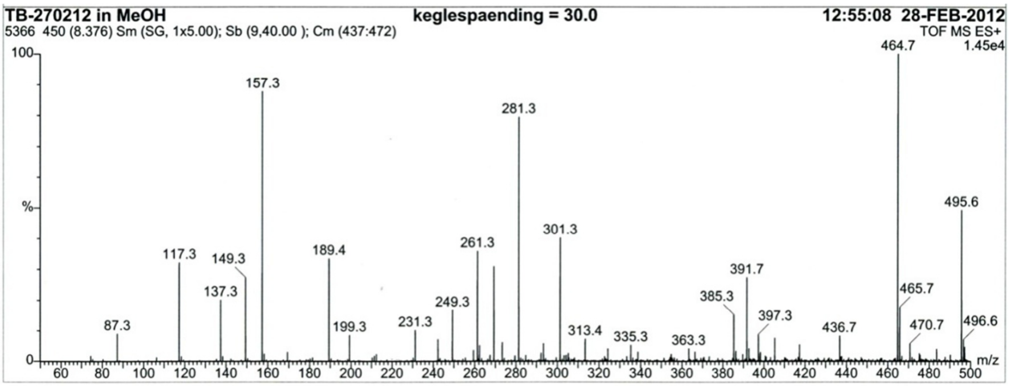

I: Spectrum of freshly prepared solution

Figure 18 Electrospray mass spectrometry (ESP) of MnF_3 in MeOH

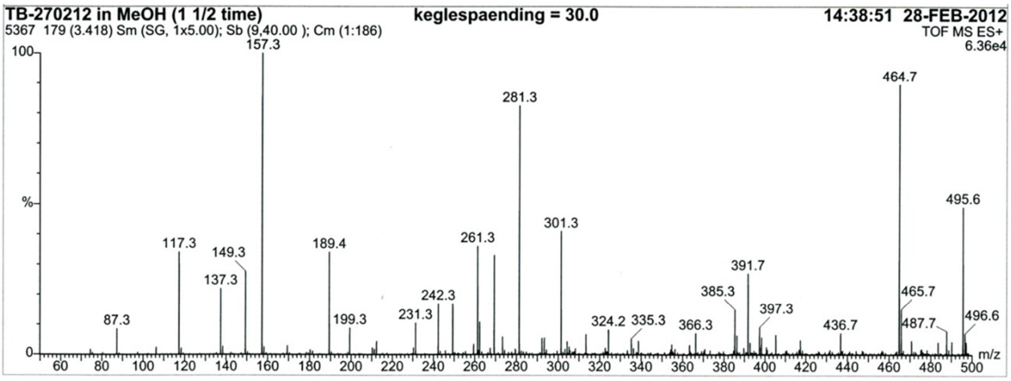

II: Spectrum of solution after 1½ h

Figure 18 (*continued*) Electrospray mass spectrometry (ESP) of MnF_3 in MeOH

Development of a method for synthesis of *trans*-[Mn(F)$_2$(salen-5H)]$^-$

As will be shown in the following section, addition of an excess of addition of solid tetraethylammonium fluoride, [Et$_4$N]F to a dilute solution of *catena*-[Mn(μ-F)(salen-5H)] in an appropriate solvent such as *N*-methylformamide results in quantitative *in situ* formation of the difluoridobis(salicylidene)ethylenediaminatomanganate(III) anion, *trans*-[Mn(F)$_2$(salen-5H)]$^-$.

$$\text{[Mn(F)(salen-5H)]} + [\text{Et}_4\text{N}]\text{F} \xrightarrow{\text{nmf}} \textit{trans}\text{-[Mn(F)}_2\text{(salen-5H)]}^- + [\text{Et}_4\text{N}]\text{X} \quad (4.12)$$

The bromide complex, [Mn(Br)(salen-5H)], which is known from literature, [227] to participate effectively in ligand substitution, can also be as reactant for the *in situ* formation of *trans*-[Mn(F)$_2$(salen-5H)]$^-$ as shown in (4.13). It is likely that a variety of different [Mn(X)(salen-5H)] complexes can be used in the synthesis as long as the axial ligand, X is relatively weakly bound *e.g.* for the previously described aqua complexes.

$$\begin{aligned}&\text{[Mn(Br)(salen-5H)]} + 2\,[\text{Et}_4\text{N}]\text{F} \xrightarrow{\text{nmf}} \\ &\qquad [\text{Et}_4\text{N}]^+ + \textit{trans}\text{-[Mn(F)}_2\text{(salen-5H)]}^- + [\text{Et}_4\text{N}]\text{Br}\end{aligned} \quad (4.13)$$

Both the [Mn(F)(salen-5H)] and difluorido anion, *trans*-[Mn(F)$_2$(salen-5H)]$^-$ are unambiguously identified by EPR spectroscopy. The aim of isolating the difluorido anion from these solutions has not yet been achieved. The feasibility of isolation of the ion from methanolic, ethanolic and aqueous solutions was tested with [Et$_4$N]F and simple alkali metal fluorides. In all cases, with the exception of potassium fluoride, no visual evidence of reaction in the form of precipitation was seen. However, for KF instantaneous precipitation of a greenish

mud-like substance was observed upon mixing solutions of the reactants in either water or ethanol. The resulting product was very soluble in both methanol and excess of water.
That the precipitate is a result of a simple salting-out process initiated by the fluoride ion addition is questionable due to the significantly different color of the substance compared with *catena*-[Mn(μ-F)(salen-5H)]. Important information about the composition of the precipitated material can eventually be obtained by low temperature EPR spectroscopy.
However, there is no firm justification to conclude on the given basis that the product contains the *trans*-$[Mn(F)_2(salen)]^-$ ion - only that the possibility exists. Nevertheless, a reaction equation for the possible reaction is given in (4.14).

$$[Mn(F)(salen\text{-}5H)] + KF \xrightarrow[EtOH/H_2O]{?} K[Mn(F)_2(salen\text{-}5H)] \qquad (4.14)$$

Other products may also be formed during the reaction, possibly in a mixture. It could, thus, be imagined, that complexes of the bifluorido ligand, HF_2^- were formed under the reaction conditions. This ion results from the reaction of HF and F^-, and a bifluorido complex may be formed *in situ* via reaction between the coordinated fluorido ligand on [Mn(F)(salen-5H)] the water present in the solvent and added fluoride. If the water takes part in the reaction, it may be the reason why only water and ethanol as solvent give rise to the reaction and not strictly anhydrous methanol.

STRUCTURAL DESCRIPTION OF *catena*-[Mn(μ-F)(salen-5R)]

Introduction

Transition metal complexes of H_2salen as well as other related Schiff-base ligands, SB form a variety of structural motifs. The rigid structure of the Schiff-base ligand leads to octahedral 6-coordination and square-pyramidal 5-coordination geometries as the most common coordination geometries. More complex structures may be derived from these two basic coordination geometries, see Figure 19, *e.g.*

- [Mn(X)(SB)], [Mn(X)(S)(SB)]
- *catena*-[Mn(μ-X)(SB)]
- [Mn_2(SB)$_2$(μ-X)], [Mn_2(SB)$_2$(S)$_2$].

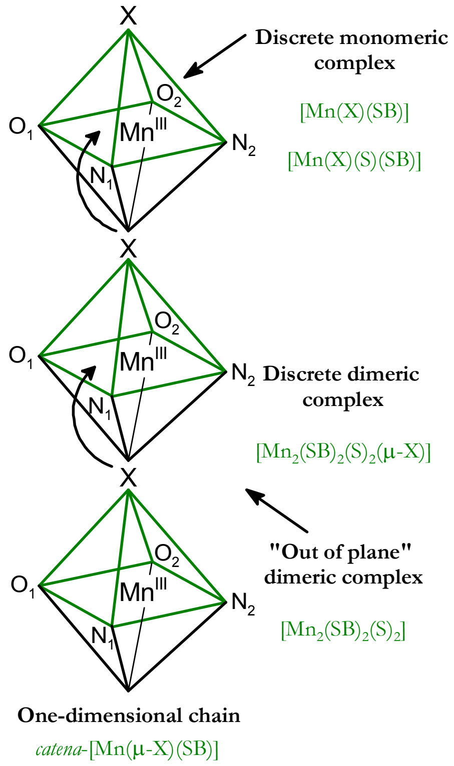

Figure 19 Summary of different types of Schiff base complexes and their interrelation

Discrete [Mn(X)(SB)] and [Mn(X)(S)(SB)]

In both octahedral 6-coordination and square-pyramidal 5-coordination the tetradentate Schiff-base ligand span the equatorial plane with the set of ligators {$O_1N_1N_2O_2$}, which combined with simple coordination of a mono anionic ligand, X in apex gives the overall stoichiometry [Mn(X)(SB)] as found in *e.g.* [Mn(Cl)(salen)]·MeCN, [220] and [Mn(NCS)(salen)], [239]. Coordination of solvent or in general other neutral ligands to the vacant coordination site on the Mn(III) ion by leads to 6-coordinated systems with the general formula *trans*-[Mn(X)(SB)(S)] as in *trans*-[Mn(Cl)(H_2O)(salen-5R)] for R=H [221], Br [222]. Although these complexes do not exhibit bridging through the axial chlorido ligand, the axial water ligand form hydrogen bonds to the oxygen ligator atoms from the salen-ligand in neighboring entities. These structures consists of discrete units and can be found in a number of complexes. An almost complete compilation of the discrete Mn(III)-Schiff-base systems which have been structurally characterized to date are given in Table 3. As explained in the previous section a wide range of Mn(III)-Schiff-base complexes with varying axial ligands are known. Table 3 shows, however, that only a very limited selection of these systems are structurally characterized in practice it is only the complexes with Cl^-, N_3^-, $RCOO^-$, H_2O and ROH as axial ligands.

X	[Mn(X_1)(X_2)(SB)]	Mn–O	Mn–N	Mn–X_1	Mn–X_2	Ref.
**Halogenido--compl.**	[Mn(Cl)(salen)]					[220]
	[Mn(Cl)(salen-5,7Me)]					[242]
	[Mn(Cl)(salcy-3,5^{t}Bu)]	1.860	1.980	2.360	—	[144]
	—	1.867	1.983	2.383	—	[243]
	[Mn(Cl)(saltmen)]					[244]
	[Mn(Cl)(saldphen-7Ph)]	1.865	1.974	2.391	—	[245]
	[Mn(Cl)(H_2O)(salen)]					[221]
	—	1.883	1.983	2.621	2.333	[246]
	—	1.876	1.977	2.584	2.266	[222]
	[Mn(Cl)(H_2O)(salen-5Cl)]	1.879	1.980	2.572	2.319	[247]
	[Mn(Cl)(H_2O)(salen-5Br)]	1.875	1.980	2.587	2.274	[248]
	[Mn(Cl)(H_2O)(salen-3EtO)]	1.876	1.984		2.328	[249]
	[Mn(Cl)(H_2O)(salen-5NO_2)]	1.908	2.091	2.306	2.246	[250]
	[Mn(Cl)(H_2O)(salmen-3EtO)]					[251]
	[Mn(Cl)(EtOH)(saldphen)]	1.873	1.989	2.573	2.307	[252]
	[Mn(Cl){salen-3(O=CH-),5Me}]	1.878	1.978	2.369	—	[253]
**Carboxylato-compl.**	[Mn(HCOO)(H_2O)(salen-3EtO)]	1.924	2.009	2.040	1.985	[254]
	[Mn(OAc)(H_2O)(salen-3MeO)]	1.888	1.987	2.130	2.334	[255]
	[Mn(OAc)(H_2O)(salcy-3EtO)]					[256]
	[Mn(OAc)(H_2O)(salen-5NO_2)]	1.886	1.974	1.890	2.347	[255]
	[Mn(PhCOO)(salen)]					[257]
	[Mn{4(OH)-Ph-COO}(salen3MeO)]	1.883	1.985	2.116	2.326	[258]
	[Mn{3(NO_2)-PhCOO}(H_2O)(salen)]	1.900	1.996	2.151	2.325	[259]
	[Mn(BzCOO)(H_2O)(salen-3MeO)]	1.884	1.983	2.135	2.353	[255]
	[Mn(BzCOO)(H_2O)(salpn-3MeO)]	1.913	2.038	2.139	2.316	[255]
**Azido-compl.**	[Mn(N_3)(salen-5Br)]	1.888	1.988	2.130	—	[260]
	[Mn(N_3)(salen-3,5^{t}Bu)]					[218]
	[Mn(N_3)(salcy-3,5^{t}Bu)]					[218]
	[Mn(N_3)(MeOH)(salcy)]	1.879	1.993	2.201	2.431	[261]
	[Mn(N_3)(MeOH)(salcy-3,5^{t}Bu)]	1.877	1.985	2.214	2.358	[262]

Table 3 Geometric parameters for complexes of type [Mn(X)(SB)]

Polymeric complexes, *catena*-[Mn(X)(salen-5R)] are indicated with "∞" as subscript.
Solvents of crystallization have been omitted for clarity. Bond lengths are given as averages.

X	[Mn(X_1)(X_2)(SB)]	Mn–O	Mn–N	Mn–X_1	Mn–X_2	Ref.
Aqua-compl.	[Mn(H_2O)$_2$(salen-3,5Cl)]ClO_4	1.880	1.968	2.272	2.220	[263]
	—					[250]
	[Mn(H_2O)$_2$(salen-5MeO)]ClO_4	1.8702	1.978	2.259	2.313	[264]
	[Mn(H_2O)$_2$(salcy-3,5Br)]ClO_4	1.889	1.978	2.278	2.269	[265]
	[Mn(H_2O)$_2$(salcy-3MeO)]Cl	1.885	1.987	2.214		[266]
	[Mn(H_2O)$_2$(salcy-3CHO,5Me)]Cl	1.881	1.986	2.247	—	[267]
	[Mn(H_2O)$_2$(salcy-3CHO,5Me)]PF_6	1.878	1.972	2.256	—	[268]
	[Mn(H_2O)$_2$(salmen-3CHO-5Me)]Cl	1.875	1.976	2.247	—	[269]
	[Mn(H_2O)$_2$(salmen-3MeO)]ClO_4	1.888	1.974	2.256	2.238	[270]
	[Mn(H_2O)$_2$(saldmen-3MeO)]ClO_4	1.851	1.962	2.360	2.237	[271]
	[Mn(H_2O)$_2$(saldphen-3^{t}Bu,5Br)]BF_4					[144]
	[Mn(H_2O)(MeOH)(salen-3,5Cl)]	1.886	1.988	2.222	2.266	[264]
	[Mn(H_2O)(MeOH)(salen-5NO_2)]ClO_4	1.904	1.988	2.263	2.233	[272]
	[Mn(H_2O)(MeOH)(salmen-3MeO]ClO_4					[273]
	[Mn(H_2O)(EtOH)(salcy)]ClO_4					[242]
	[Mn(MeOH)$_2$(saltmen-5Br)]ClO_4					[274]
	[Mn(EtOH)$_2$(saldphen-5Me)]ClO_4					[242]
	[Mn(^{i}PrOH)(salcy-3,5^{t}Bu)]SbF_6					[275]
Other compl.	[Mn(H_2O)(1,4-dioxane)(salcy3,5^{t}Bu)]ClO_4					[144]
	[Mn(4NO_2-Ph-S)(salen)]	1.879	1.979	2.491	—	[276]
	[Mn(NCO)(salen)]	1.882	1.981	2.118	—	[277]
	[Mn(NCS)(salen)]	1.981	1.878	2.160	—	[278]
	[Mn(ClO_4)(MeOH)(salen-3,5Br))]					[250]
	[Mn(OAc)(salen-5H)]$_\infty$	1.888	1.989	2.201		
	[Mn(μ-HCOO)(salen-5H)]$_\infty$					
	[Mn(μ-NO_2)(salen-5H)]$_\infty$					
	[Mn(NO_3)(salen-5H)]$_\infty$					
	[Mn(μ_2-NCNH)(salen-5Br)]					
	[Mn(*N*-4-pyridylglycinato)(salen)]	1.897	1.997	2.309	2.203	
	[Mn{(4-pyridylthio)acetate}(salen)]	1.894	1.980	2.437	2.132	
	[Mn{*N*-4-cyanophenylglycinato}(salen)]	1.904	1.978	2.268	2.273	
	[Mn(CN)(salen-5H)]$_\infty$					
	[Mn(N_3)(salen-5H)]$_\infty$	1.882	1.984	2.307		
	[Mn($\mu_{1,3}$-N_3)(salen-5F)]$_\infty$	1.879	1.981	2.307		[216]
	[Mn($\mu_{1,3}$-N_3)(salen-5Br)]$_\infty$					[229]

Table 3 (*continued*) Geometric parameters for complexes of type [Mn(X)(SB)]

1D Infinite coordination polymers *catena*-[Mn(μ-X)(SB)]

Presence of potential bridging ligands as the mono anionic halides (Cl^-) or pseudo halides (CN^-, SCN^-, N_3^-) opens up the possibility of polymerization and establishment of a 1D infinit chain coordination polymers by coordination of a axial monoanionic ligand, X to the sixth and free coordination position on a subsequent metal center. This bond formation results in a distorted octahedral coordination polyhedra. The establishment of a coordination polymer through a bridging ligand, X is schematically shown in Figure 19. One dimensional coordination polymers in form of *catena*-[Mn(μ-X)(SB)] are known in several cases among others in the important starting material *catena*-[Mn(μ-OAc)(salen)], the nitrato-complex *catena*-[Mn(μ-NO_3)(salen)], [279] the azido complexes *catena*-[Mn(N_3)(salen-5R)] for R=H [232], F [216], Br [229], OMe [216] and *catena*-[Mn(N_3)(salpn)], [230]. Cyanide occupies a unique position as ambidentate bridging ligand in the chain structures of *catena*-[Mn(CN)(salen)], [228] and *catena*-[Mn(salen)Ag$(CN)_2$], [232]. In the latter case the linear dicyanoargentate(I), $[Ag(CN)_2]^-$ forms linear chains by coordination of the nitrogen ligator of the cyanido ligand to the Mn(III)-salen entity. Besides being capable of forming chain structures, Mn(salen) building blocks have found frequent usage as blocking ligands in formation of discrete polynuclear complexes based on hexa- (and octa-) cyano complexes of the *d*-block metals. Such systems have been intensely investigated as single molecule magnets. [142]

The 1D coordination polymer structures are most frequently found for simple unsubstituted salen-ligands, as in the listed azido complexes. More steric demanding ligands, such as salen-3,5^{t}Bu and salcy-3,5^{t}Bu, [218] mostly result in structures consisting of discrete units, but such structures can also be found in simple unsubstituted systems as [Mn(N_3)(saloph)] [216].

Discrete dimers [$Mn_2(SB)_2(S)_2$(μ-X)]

Polymerization may stop before a real 1D infinite coordination polymer is reached. This is seen for the group of descrete dimers, [$Mn_2(SB)_2(S)_2$(μ-X)]. Which can be viewed as constructed from two $[Mn(SB)(S)]^+$ bridged by a common ligand, X. This motif is often seen with oxygen as ligator atom originating from the salen ligand or as oxo, hydroxide ligands, *e.g.* in [Mn(μ_2-OAc)(salen-3MeO)]·H_2O. Some examples of this type of dimerization of with other transition metals centers include: [Cr(salen-5Br)]$_2$O, [Bu_4N][Cr(salen)(CN)]$_2$CN·4H_2O and [Co(salen)$(py)_2$]BF_4. A general feature of all these systems is that the bridging ligand is poly-atomic, only a few exampels on mono-atomic briding in Mn(III)-SB complexes exisist *e.g.* the oxo bridged dinuclear [Mn_2(salen-5Br)$_2$(μ-O)]. [280] Importantly, no examples of chain structures involving salen-type ligands and mono-atomic bridges had been reported before the work presented here.

"Out of plane" dimers [$Mn_2(SB)_2(S)_2$]

"Out of plane" dimerization in form of the dimers through cyclization is also common and seen in [Cr(salen)(OH)]$_2$ and [Mn(H_2O)(salen)]$_2$(ClO_4)$_2$. [279]

Structure of *catena*-[Mn(μ-F)(salen-5R)] for R=H, F, Cl

The structure of a selection of compounds belonging to the new group *catena*-[Mn(μ-F)(salen-5R)] for R=H, F, Cl has been solved by a single crystal X-ray diffraction. Detailed information about the single crystal diffraction experiment, data collection and refinement data are given in the Appendix 3 "X-ray diffraction". The single crystal structure of the parent *catena*-[Mn(μ-F)(salen-5H)] has been published in Paper 5. In addition, the article includes a powder diffraction pattern of the compound

The structures of *catena*-[Mn(μ-F)(.salen-5R)] for R=H, F, Cl exhibit some common features - among which the most important is the one dimensional unsupported fluorido bridged homo-metallic chain of Mn(III). Mn(III) is the centralion of distorted octahedral coordination polyhedra formed by the equatorial tetradentate H_2salen-5R ligand and two axially positioned fluorido ligands. In accordance with the situation outlined in Figure 19, one of the axial fluorido ligands coordinates to the neighboring Mn(III) ion establishing the infinite 1D coordination polymer. A graphical representation of the structures of *catena*-[Mn(μ-F)(salen-5R)] for R=H, F is shown in Figure 20 and Figure 21, while the unit cell content for *catena*-[Mn(μ-F)(salen-5H)] is shown in Figure 23. A selection of geometric parameters (bond lengths and angles) for the coordination polyhedra in *catena*-[Mn(μ-F)(salen-5R)] are given in Table 4 (R=H), Table 5 (R=F) and Table 6 (R=Cl). These data were used to simulate a theoretical powder diffraction pattern. This, as well as the experimental pattern shown in Figure 22. The two patterns show a clear agreement, which can be taken as evidence of high purity of the bulk powder sample as well as evidence of the representability of the single crystal structure.

All three complexes crystalize in the triclinic crystal system and belong to the space group *P*1. The structures differ in the number crystallographically inequivalent Mn(III) centers in the unit cell. In case of *catena*-[Mn(μ-F)(salen-5R)] for R=H, Cl there are there inequivalent Mn(III) centers, while only two are found in in *catena*-[Mn(μ-F)(salen-5F)].The axial Mn–F bonds distances fall within the range of 2.0492(2) Å–2.0956(2) Å with average 2.0802(9) Å in case of *catena*-[Mn(μ-F)(salen-5H)] and similarly for *catena*-[Mn(μ-F)(salen-5R)] the averages are 2.0590(5) Å (R=F) and 2.0570(8) Å (R=Cl). The two Mn–F bonds associated with each manganese center are slightly different in length *e.g.* Mn_I–F_1 2.0492(2) Å versus Mn_I–F_2 2.0915(6) Å in *catena*-[Mn(μ-F)(salen-5H)]. As stated in the previous section, the di-fluorido anion, *trans*-$[Mn(F)_2(salen\text{-}5H)]^-$ exists and can be prepared quantitatively by addition of fluoride in excess of *catena*-[Mn(μ-F)(salen-5H)]. Systems which exhibits similar reactivity are known *e.g.* the cyanide complex *catena*-[Mn(CN)(salen-5H)]. The infinite 1D coordination polymer in that system is composed of *trans*-$[Mn(CN)_2(salen\text{-}5H)]^-$ and $[Mn(salen\text{-}5H)]^+$, with alternating low- and high-spin Mn(III) centers The coordination polymer in the case of *catena*-[Mn(μ-F)(salen-5H)] cannot be viewed to be composed in this manner in the present system and it must be concluded that the polymer formed by discrete square pyramidal [Mn(F)(salen-5H)] and not alternating chains of *trans*-$[Mn(F)_2(salen\text{-}5R)]^-$ and $[Mn(salen)]^+$ moieties. This picture is recovered in the other two systems under consideration.

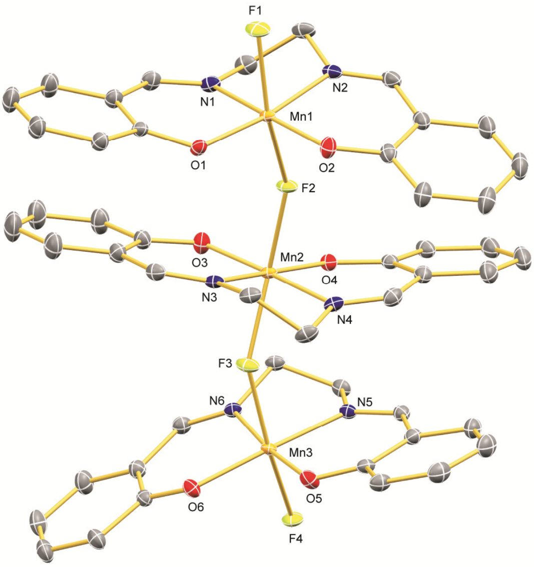

Figure 20 Molecular structure of *catena*-[Mn(μ-F)(salen-5H)]

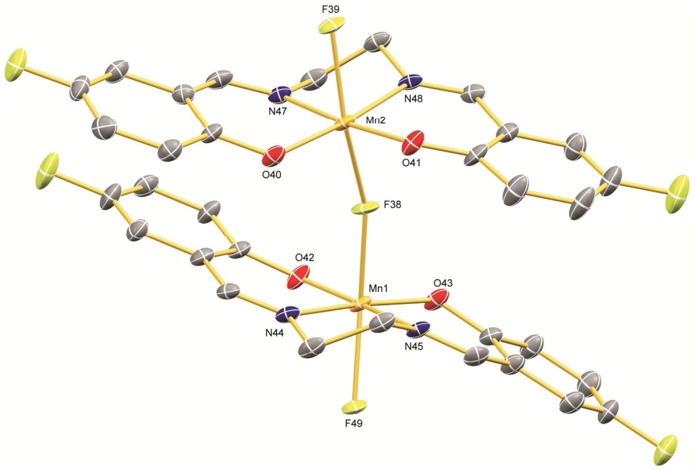

Figure 21 Molecular structure of *catena*-[Mn(μ-F)(salen-5F)]

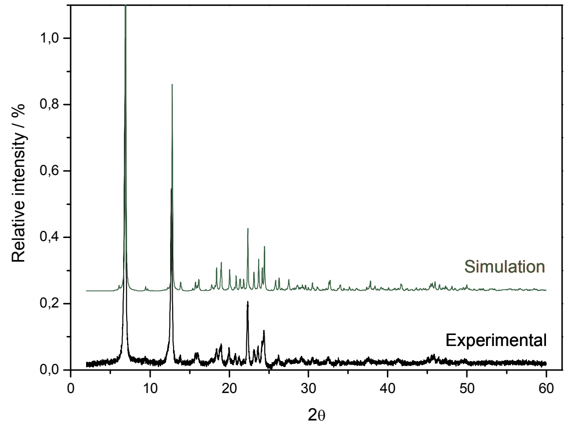

Figure 22 Powder diffraction pattern *catena*-[Mn(μ-F)(salen-5H)]

Comparing the average axial Mn–F bond for *catena*-[Mn(μ-F)(salen-5R)] with the Mn–F bond lengths found in molecular fluorido complexes, $[MnF_n]^{3-n}$ for n=3-6 given in Table 2 show direct correspondance with the axial Mn–F bond lengths (2.06–2.09 Å) in these systems. In spite of the inherent low symmetry around the manganese centers, it is, thus, safe to conclude that the manganese centers in *catena*-[Mn(μ-F)(salen-5R)] for R=H, F, Cl are subject to tetragonal elongation by Jahn-Teller distortion. The different number of inequivalent Mn(III) centers gives rise to three different bridging ∠Mn–F–Mn angles in *catena*-[Mn(μ-F)(salen-5R)] for R=H, Cl. For salen-5H, these are 150.43(4) ° (∠Mn_2–F_3–Mn_3), 151.72(3) ° (∠Mn_1–F_2–Mn_2) and 180.0 ° (∠Mn_1–F_1–Mn_1 and ∠Mn_3–F_4–Mn_3) while the corresponding angles in the case of salen-5Cl are 157.02(6) °, 161.22(7) ° and 180.0 °. In case of salen-5F the two different bridging ∠Mn–F–Mn angles fall, compared to the other two, in a narrow inverval 163.645(7) ° and 166.78(7) °. The bond angles reflect semilinear fluorido bridges, as expected for fluoride bridging (*cf.* Chapter 2). From the three systems being compared, it can be seen that unsubstituted *catena*-[Mn(μ-F)(salen-5H)] forms the most bent bridges while the substituted systems form more linear bridges. Rationalization of this relationship is not straight-forward. On one hand, steric effects from packing of the larger ligand system have some influence one the other hand the such influence could be expected to be related to substituent size. The most open angles would then be expected with the largest substituent. From the data given above, it is seen that this is not the case.

The equatorial coordination sphere {O_2N_2} spanned by the SB ligand around the central Mn(III) center is in terms of bond lengths and angles close to identical in all of *catena*-[Mn(μ-F)(salen-5R)] for R=H, F, Cl, with average Mn–O: 1.9007(8), 1.899(3), 1.9050(7) Å and Mn–N: 2.0017(8), 2.0105(3), 1.9996(16) Å for the three systems, respectively. It must be concluded that substitution pattern on the salen-ligand does not significantly affect the details of the conformation of the coordination polyhedra. Comparing the equatorial coordination geometry of *catena*-[Mn(μ-F)(salen-5R)] with other similar systems as the polymers as shown in Table 3 but also with discrete complexes such as [Mn(Cl)(salen)], [220] underlines the invariance of the Schiff-base coordination with respect to changes in the substitution pattern and the remaining coordination sphere. The "out of plane distance" for the Mn(III) ion relative to the plane consisting of the equatorial ligator atoms {O_2N_2} is for *catena*-[Mn(F)(salen-5H)] 0.002 Å

($O_1N_1N_2O_2$), 0.026 Å ($O_3N_3N_4O_4$) and 0.030 Å ($O_5N_5N_6O_6$) and with nearly identical values for both *catena*-[Mn(μ-F)(salen-5Cl)] (0.006 Å, 0.021 Å and 0.030 Å) and *catena*-[Mn(μ-F)(salen-5F)] (0.003 Å ($O_{42}N_{44}N_{45}O_{43}$) and 0.029 Å ($O_{40}N_{47}N_{48}O_{41}$)). All values, even with their large internal variation of are remarkably small compared with the out-of-plane distortions for discrete complexes, for instance [Mn(Cl)(salen-5H)] (0.19 Å), [220] but comparable to the values for other 1D coordination polymers, such as *catena*-[Mn($\mu_{1,3}$-N_3)(salen-5H)] (0.019 Å), [232] and *catena*-[Mn($\mu_{1,3}$-N_3)(salen-5F)] (0.01 Å), [216].
For all three fluoride bridged structures, the chains feature alternating orientations of the salen-ligand with respect to the chain axis corresponding to a roation of ligand at 180 ° from one Mn(III) center to the next. This behavior is different from what is observed in the more widely spaced 1D coordination polymeres such as *catena*-[Mn(N_3)(salen-5H)].

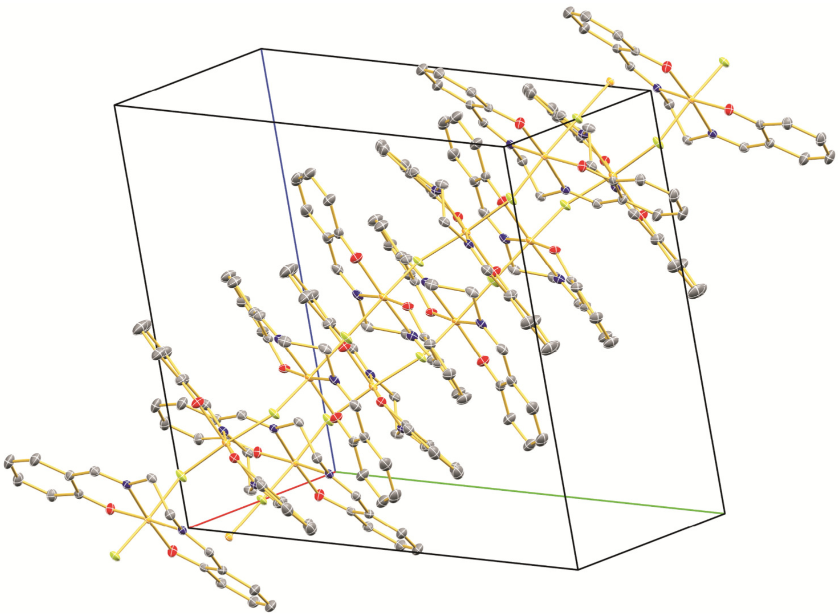

Figure 23 Unit cell for *catena*-[Mn(μ-F)(salen-5H)]

There has been some interest in the literature regarding a possible relationship between substitution pattern, and electronic nature of substituents on the Schiff-base ligand and effects on the bond length(s) between Mn(III) centre and the axial ligand(s) . A correlation between the nature of the equatorial ligand and the axial bond distance is interesting in terms of understanding *e.g.* the catalytic properties of such systems and allows a more rational way of choosing the ligand given the system specific properties.

Bond length (Å)				Bond angle (°)			
Mn_1–F_1	2.0492(2)	Mn_2–O_4	1.9119(7)	O_1–Mn_1–O_2	94.16(3)	N_2–Mn_1–F_1	85.64(3)
Mn_1–F_2	2.0915(6)	Mn_3–O_5	1.8924(8)	O_1–Mn_1–N_2	173.64(3)	N_1–Mn1–F_1	82.58(2)
Mn_2–F_2	2.0790(6)	Mn_3–O_6	1.9045(7)	O_2–Mn_1–N_2	90.82(3)	O_1–Mn_1–F_2	90.70(3
Mn_2–F_3	2.0833(7)	Mn_1–N_1	2.0059(8)	O_1–Mn_1–N_1	92.63(4)	O_2–Mn_1–F_2	102.84(3)
Mn_3–F_3	2.0829(6)	Mn_1–N_2	2.0059(8)	O_2–Mn_1–N_1	172.04(3)	N_2– Mn_1–F_2	84.34(3)
Mn_3–F_4	2.0956(2)	Mn_2–N_3	2.0008(8)	N_2–Mn_1–N_1	82.71(4)	N_1– Mn_1– F_2	81.21(3)
Mn_1–O_1	1.8986(8)	Mn_2–N_4	1.9949(9)	O_1–Mn_1–F_1	98.07(3)	F_1–Mn_1–F_2	161.885(19)
Mn_1–O_2	1.9044(7)	Mn_3–N_5	2.0028(8)	O_2–Mn_1–F_1	92.34(2)	Mn_2–F_3–Mn_3	150.43(4)
Mn_2–O_3	1.8925(8)	Mn_3–N_6	2.0000(8)				

Table 4 Selected geometric parameters for coordination polyhedra in *catena*-[Mn(μ-F)(salen-5H)]

Bond length (Å)				Bond angle (°)			
Mn_1–F_{38}	2.056(2)	Mn_2–O_{40}	1.903(3)	O_{43}–Mn_1–O_{42}	95.53(12)	N_{44}–Mn_1–F_{38}	84.20(12)
Mn_1–F_{49}	2.0705(8)	Mn_2–O_{41}	1.899(3)	O_{43}–Mn_1–N_{44}	172.61(13)	N_{45}–Mn_1–F_{38}	86.47(12)
Mn_2–F_{38}	2.055(2)	Mn_1–N_{44}	2.002(3)	O_{42}–Mn_1–N_{44}	91.70(13)	O_{43}–Mn_1–F_{49}	91.09(9)
Mn_2–F_{39}	2.0546(8)	Mn_1–N_{45}	2.016(3)	O_{43}–Mn_1–N_{45}	90.88(13)	O_{42}–Mn_1–F_{49}	98.68(10)
Mn_1–O_{42}	1.898(3)	Mn_2–N_{47}	1.997(3)	O_{43}–Mn_1–N_{45}	173.29(13)	N_{44}– Mn_1–F_{49}	86.28(10)
Mn_1–O_{43}	1.896(3)	Mn_2–N_{48}	2.007(3)	N_{44}–Mn_1–N_{45}	81.95(14)	N_{45}– Mn_1– F_{49}	83.12(10)
				O_{43}–Mn_1–F_{38}	97.22(12)	F_{38}–Mn_1–F_{49}	166.78(7)
				O_{42}–Mn_1–F_{38}	90.77(12)	Mn_2–F_{38}–Mn_1	160.70(14)

Table 5 Selected geometric parameters for coordination polyhedra in *catena*-[Mn(μ-F)(salen-5F)]

Bond length (Å)				Bond angle (°)			
Mn_1–F_{73}	2.0436(3)	Mn_2–O_{63}	1.9242(13)	O_{66}–Mn_1–O_{65}	94.34(6)	N_{59}–Mn_1–F_{73}	81.33(5)
Mn_1–F_6	2.0577(11)	Mn_2–O_{64}	1.8907(14)	O_{66}–Mn_1–N_{59}	173.56(6)	N_{60}–Mn_1–F_{73}	87.25(5)
Mn_2–F_{82}	2.0596(3)	Mn_1–N_{59}	1.9964(16)	O_{65}–Mn_1–N_{59}	91.46(6)	O_{66}–Mn_1–F_6	93.64(6)
Mn_2–F_5	2.0630(11)	Mn_1–N_{60}	1.9978(16)	O_{66}–Mn_1–N_{60}	91.66(6)	O_{65}–Mn_1–F_6	97.78(5)
Mn_1–O_{65}	1.8919(14)	Mn_2–N_{57}	2.0044(16)	O_{65}–Mn_1–N_{60}	173.92(6)	N_{59}– Mn_1–F_6	88.32(6)
Mn_1–O_{65}	1.9139(14)	Mn_2–N_{58}	1.9991(17)	N_{59}–Mn_1–N_{60}	82.50(7)	N_{60}– Mn_1– F_6	82.78(6)
				O_{66}–Mn_1–F_{73}	95.75(4)	F_{73}–Mn_1–F_6	166.48(4)
				O_{65}–Mn_1–F_{73}	91.18(4)	Mn_3–F_6–Mn_1	161.22(7)

Table 6 Selected geometric parameters for coordination polyhedra in *catena*-[Mn(μ-F)(salen-Cl)]

By comparing the average bond lengths (from Table 4, Table 5 and Table 6) for the axial Mn–F bond in *catena*-[Mn(μ-F)(salen-5R)] for R=H(2.0802(9) Å), F(2.0605(4) Å), Cl(2.0570(8) Å) it is seen that the Mn–F bond length diminishes statistically significantly through the series. The variation between the unsubstituted salen complex and the halogen functionalized systems follows the expected trend based on electronic effects on the donor properties of the SB ligand, but the ordering of the F and Cl substituted systems is not simply rationalized. In general terms it is assumable that electron donating substituents in 5 position *e.g.* OCH_3, on the aromatic ligand system give rise to an increased axial Mn–X bond distance while electron accepting substituents give the opposite effect due to less competition for donation.Structurally characterized examples from the literature, that allow for quantification of this effect by comparison with the *catena*-[Mn(F)(salen-5R)] complexes are few in number, and adequate data are only available for axial chlorido ligands. In the series [Mn(Cl)(salen-5R)] for R=Cl (2.572(1) Å), H (2.4650(16) Å) and NO_2 (2.306(4) Å) the variation is large, but again not simple to rationalize.

MAGNETIC BEHAVIOR AND EPR-SPECTROSCOPY OF 1D Mn(III)

EPR-spectroscopy on solutions of *catena*-[Mn(μ-F)(salen-5H)]

Parallel-mode electron paramagnetic resonance spectroscopy was performed on solutions of *catena*-[Mn(F)(salen-5H)] with and without added fluoride. The presence of the dianion $[Mn(F)_2(salen\text{-}5H)]^-$ in fluoride containing media could elegant be demonstrated by EPR-spectroscopy (Figure 24). That the two spectra I and II qualitatively gives rise to an interpretation of the reaction and the species present as indicated by equation (4.15) follows from a simple analysis of the number of transitions and their relative intensity for each spectrum.

$$\begin{aligned} &\textit{cartena}\text{-[Mn(}\mu\text{-F)(salen-5H)]}(s) \xrightarrow{\text{nmf}} \text{[Mn(F)(salen-5H)]}(sol) \\ &\text{[Mn(F)(salen-5H)]}(sol) + \text{F}^-(sol) \xrightarrow{\text{nmf}} \textit{trans}\text{-[Mn(F)}_2\text{(salen-5H)]}^-(sol) \end{aligned} \qquad (4.15)$$

Qualitative description

The spectrum of experiment I consists of a seven lines pattern with an approximately relative intensity of 1:2:2:2:2:2:1. Correspondingly, in the spectrum of experiment II eight lines are observed with an approximately relative intensity of 1:3:4:4:4:4:3:1. The natural isotope distribution of manganese is 100 atom % of ^{55}Mn. The number of transitions in the two spectra follows from the splitting of the fine structure. Interaction between the nuclear spin (angular moment) of the ^{55}Mn (I^{Mn}=5/2) nucleus with the magnetic moment of the unpaired electrons

originating from the Mn(III) ion itself as well as nuclear magnetic moments of the fluorido ligands (I^F=1/2) coordinating to the Mn(III) ion. The two types of interactions are respectively termed hyperfine and superhyperfine interaction. The numbers of levels in each structure are given by the nuclear magnetic spin quantum number, M_I as $2I + 1$. The allowed transitions between these levels are given by the selection rule:

$$\Delta M_I = 0 \quad \text{were} \quad M_I = \{I - j\}_{j=1}^{2I} \tag{4.16}$$

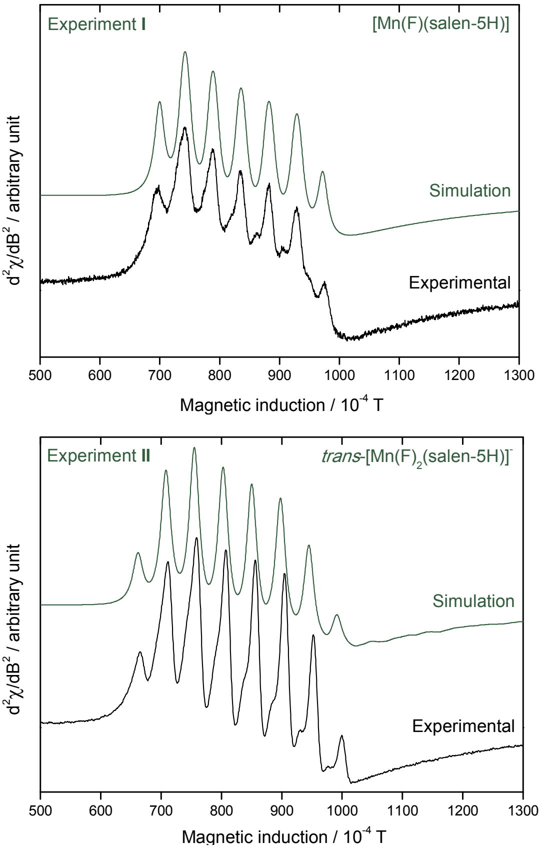

I Operational parameters
Frozen NMR glass
T/K 5.65
ν/Hz $9.416425 \cdot 10^9$

II Operational parameters
Frozen NMR glass
T/K 6.08
ν/Hz $9.410778 \cdot 10^9$

Figure 24 Low temperature EPR spectrum of [Mn(F)(salen-5H)] and $[Mn(F)_2(salen\text{-}5H)]^-$ in nmf

The spectrum, II of $[Mn(F)_2(salen\text{-}5H)]^-$ is obtained by directly addition of ~1 spatula solid $[Et_4N]F$ (or $[Me_4N]F$) to the EPR quartz tube containing the nmf-solution of *catena*-[Mn(μ-F)(salen-5H)] used in I.

Based on (4.16) follows that the fine structure split into a hyperfine structure consisting of six levels ($2I^{Mn} + 1$), with six allowed transitions between them. Each level in the hyperfine structure is split into two levels ($2I^{F} + 1$) by each fluorido ligands present. One fluorido ligand gives a superhyperfine structure consisting of twelve levels in total whereas two (non-equivalent) fluorido ligands give rise to twenty-four levels. As seen from Figure 25 large a number of these levels are degenerate and in the two systems, the number of non-degenerate levels and consequently the number of transitions are reduced to seven and eight respectively. This number of transitions is what is observed in experiment I and II, and it must therefore be concluded that the two complexes features one and two fluorido ligands, respectively. Furthermore, the predicted intensity of each transition agrees with the experiment. The given analysis of the splitting due to the superhyperfine interaction has only taken into account the fluorido ligands. However the coordination polyhedral around the central Mn (III) ion consist in addition to the fluorine ligators of two nitrogen and two oxygen ligators originating from the salen ligand. Only the nitrogen ligator could be potentially relevant to the analysis since the only oxygen isotope with non-zero nuclear spin is ^{17}O, $I(^{17}O)=5/2$ with a very low natural abundance of 0.038(1) atom%. On the other side nitrogen occurs as ^{14}N, $I(^{14}N)=1$and ^{15}N, $I(^{15}N)=1/2$with natural abundances of 99.632(7) and 0.368(7) atom%, respectively. The vanishing superhyperfine coupling to nitrogen as compared to that of fluorine reflects the electronic structure of the complexes. As emphasized in connection with the structures of the manganese fluoride chains above, the manganese centres are electronically elongated along the chain direc-

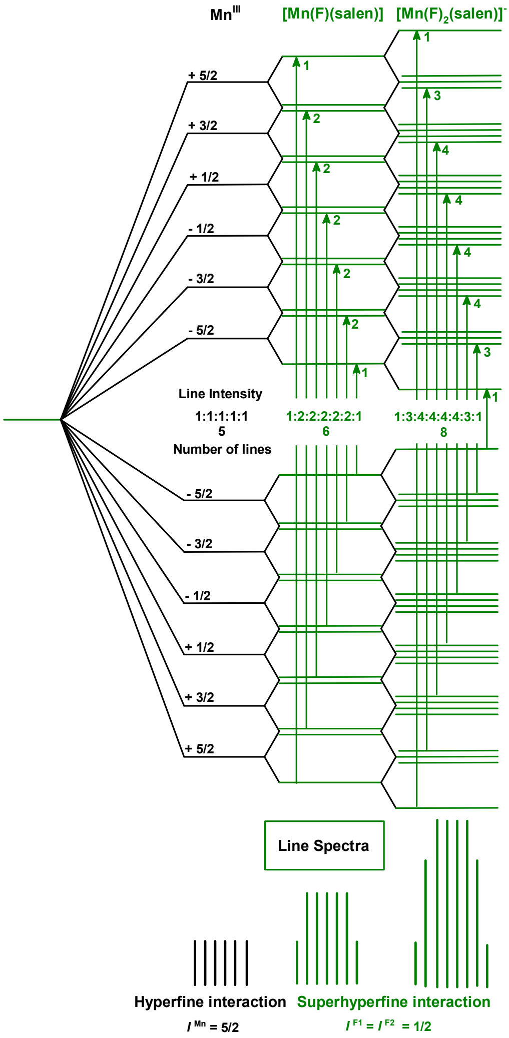

Figure 25 EPR splitting diagram for $[Mn(F)(salen)]$ and *trans*-$[Mn(F)_2(salen)]^-$

tion. This could be verified by comparison with the Jahn-Teller distorted $[MnF_6]^{3-}$ ion. The electronic consequence is that in both [Mn(F)(salen-5H)], $[Mn(F)_2(salen-5H)]^-$ and $[MnF_6]^{3-}$, the unpaired electron in the σ-antibonding $e_g(O_h)$ level populates the d_{z2} orbital. Consequently, superhyperfine couplings are large to the axial ligands and much smaller to the equatorial ligands. For the $[MnF_6]^{3-}$, this was found (Paper 1) to lead the counter-intuitive situation that the more distantly bound ligands gave rise to the largest superhyperfine couplings. For the salen complexes, this also aids in explaining the absence of detectable couplings to the equatorial nitrogen ligators.When comparing the resolution of the two spectra I and II, a significantly better resolved spectrum is obtained in case II. This can be explained in terms of the absence of free coordination sites in *trans*-$[Mn(F)_2(salen-5H)]^-$ whereas [Mn(F)(salen-5H)] holds a free axial coordination site in *trans* position to the fluorido ligand. This site will be subject for coordination of trace amounts of water present in the nmf or possibly coordination of the solvent itself. The recorded EPR-spectrum of experiment I is therefore superimposed of the individual spectra of dominating five-coordinate [Mn(F)(salen-5H)] and spectra of six-coordinate by-products.
The spectrum of [Mn(F)(salen-5H)], contributed with the important information – that the solid state polymeric *catena*-[Mn(μ-F)(salen-5H)] is cleaved symmetrically upon dissolution without any detecable tendency to form $[Mn(F)_2(salen)]^-$ and $[Mn(salen)]^+$. This results is in good agreement with the quite symmetrical fluoride bridging observed in the solid state.

Quantitative description by Spin-Hamiltonian formalism

Although the EPR spectroscopic data can be understood based on the qualitative arguments provided above, it is customary to quantify the data by parametrization of the spectral data. This is commonly done within the framework of the Spin-Hamiltonian formalism (Chapter 3). The experimental spectra discussed above, have been fittet to the theoretical Spin-Hamiltonian applicable to [Mn(F)(salen-5H)] and *trans*-$[Mn(F)_2(salen-5H)]^-$ given in (4.17). The simulation was performed by use of the software "*SIM*" written by Weihe. [281, 282] The matrix diagonalisation has been performed with the software "*SIMMGU*" also written by Weihe. [283] The parameters obtained by the simulations are collected in Table 7.

	[Mn(F)(salen)] / cm^{-1}	*trans*-$[Mn(F)_2(salen)]^-$ / cm^{-1}
g_z	7.92	7.92
Δ	0.0555	0.07130
A_z	0.01725	0.01760
$A_{z,\mathrm{ax}}$	0.0145	0.01695

Table 7 Spin-Hamiltonian parameters for [Mn(F)(salen-5H)] and *trans*-$[Mn(F)_2(salen-5H)]^-$

The recorded EPR spectrum of $[Mn(F)_2(salen-5H)]^-$ was fittet ind the interval [0.0001 °, 90 °] in steps of 0.0001° to avoid computational noise. $\gamma \in [0\,°, 90\,°]$ in steps of 1 °. Fields 500 1500 6 1.

Spin-Hamiltonian for a non-Kramers doublet is gives by:

$$\hat{H} = \Delta\hat{S}_x + \mu_B g_z B_z \hat{S}_z + A_z \hat{S}_z \cdot \hat{I}_z^{Mn} + A_{z,ax} \sum_{i=1,2} \hat{S}_z \cdot \hat{I}_z^{F_i} \quad (4.17)$$

Magnetic behaviour of *catena*-[Mn(μ-F)(salen-5H)]

The molar magnetic susceptibility has been measured for the temperature interval T/K∈[1.8; 370] in a 1 kOe magnetic field. The stability of the compound exposed to this temperature variation has been verified by collection data both up and down in temperature on the same sample. The susceptibility reaches a global maximum value of ca. $6.0 \cdot 10^{-3}$ $cm^3 \cdot mol^{-1}$ at 160 K and global minimum value of ca. $4.2 \cdot 10^{-3}$ $cm^3 \cdot mol^{-1}$ at 10 K. The χT product has a global minimum at 1.8 K demonstrating lack of uncompensated moments at low temperature and thus lack of spin-canting. The global broad maximum in χ is frequent seen in this class of Mn(III) chain compounds and is taken as an indicative of antiferromagnetic intra chain coupling.

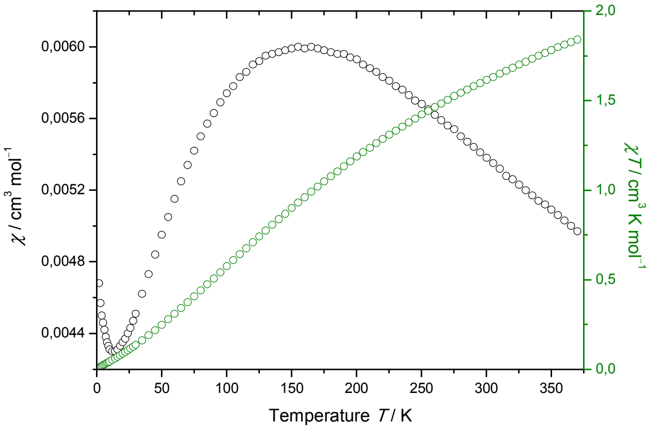

Figure 26 Temperature dependences of molar Magnetic Susceptibility of *catena*-[Mn(F)(salen-5H)]

Molar Magnetic Susceptibility in terms of χ and χT

The characterizing parameter for the magnetic properties of linear chain compounds is the intra chain coupling constant. Spin-spin interactions in a simple linear chain of spins can in some cases be described by a simple Ising model which is anisotropic by taking into account only the interaction (coupling) along the chain axis and only between neighboring spins:

$$\hat{H} = \mu_B \sum_{A}^{N} Bg\hat{S}_A + J_A \sum_{A}^{N} \vec{S}_A \cdot \vec{S}_{A+1} \quad (4.18)$$

With the sign convention used here, $J>0$ corresponds to antiferromagnetic interaction with favoured antiparallel spin alignment, $J<0$ to ferromagnetic interaction with favoured parallel spin alignment, and $J=0$ to absence of coupling. The Ising model was not used for the parameterization of the magnetic data for the present manganese chains. Mn(III) chains are commonly referred to as Heisenberg style and treated with an isotropic Spin-Hamiltonian (*cf.* Figure 27). [227]

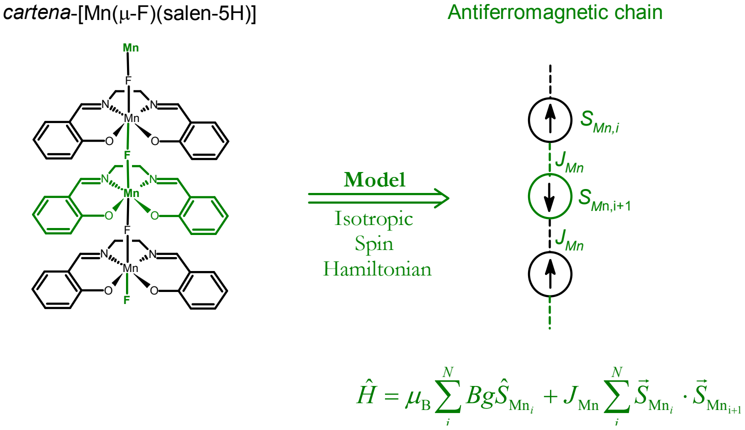

Figure 27 Model for description of magnetic behaviour of *catena*-[Mn(μ-F)(salen-5H)]

This is not directly applicable for infinite systems, but good approximations can be arrived by treating rings of increasing size by use of this Hamiltonian and employing exact diagonalization of the resulting models. This is one of the methods employed in Paper 5 for modelling the magnetic properties of *catena*-[Mn(μ-F)(salen-5H)]. Yet another approach is to seek an analytical solution to the Ising model. Consideration of rings of increasing size and extrapolation to infinite ring sizes leads to an analytical solution as shown by Fisher in 1964, [284, 285]:

$$\chi_m = \frac{N_A g^2 \mu_B^2}{3k_B T} S(S+1) \frac{1+u}{1-u} \qquad u = \coth\left(\frac{JS(S+1)}{k_B T}\right) - \frac{k_B T}{JS(S+1)} \tag{4.19}$$

The Bonner-Fisher expression can be modified to take account of *e.g.* paramagnetic impurities present with fraction $P \in [0,1]$.

$$\chi_{calc} = (1-P)\chi_m + P\chi_{para} \Rightarrow \chi_{calc} = \frac{N_A g^2 \mu_B}{3k_B T} S(S+1) \frac{1+u(1-2P)}{1-u} \tag{4.20}$$

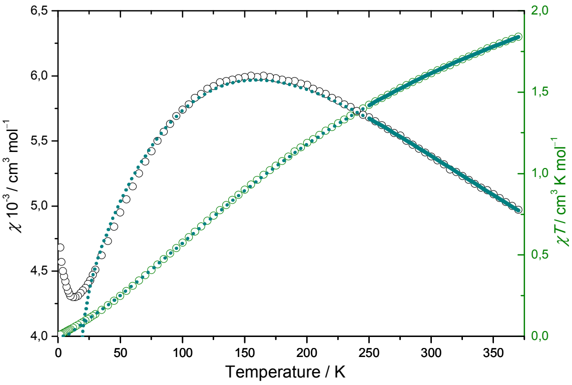

I: Exact diagonalization of rings of size up to 9

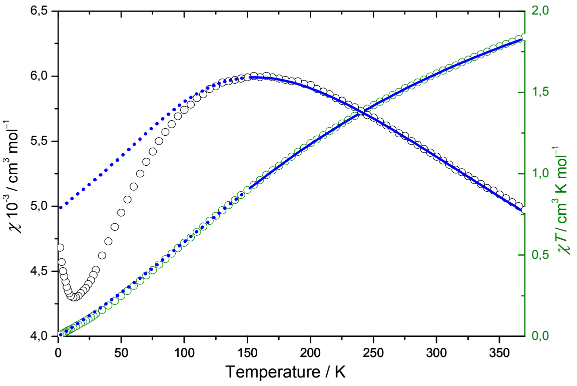

II: Parameterization by the Bonner-Fisher model

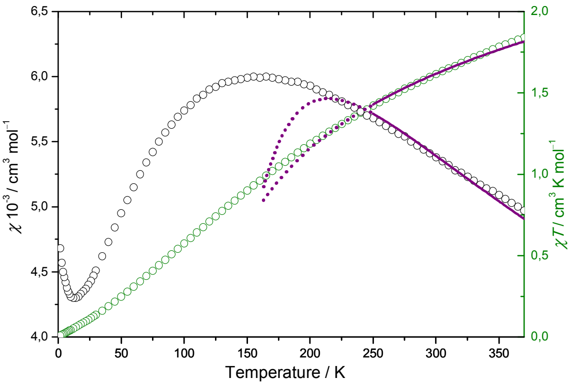

III: High-Temperature expansion

Figure 28 Modelling of the magnetic properties of *catena*-[Mn(μ-F)(salen-5H)]

As discussed below, the Fisher model is widely employed for modeling magnetic exchange in chains and the comparisons we will make, are based on this type of modeling. A final method was employed in Paper 5 for interpretation of the magnetic data of *catena*-[Mn(μ-F)(salen-5H)], namely, the so-called high-temperature expansion. This method is modelling based on a power series expansion of the Hamiltonian and is quite powerful for treating large systems at medium to high temperatures (hence the name), but fails for low temperatures. Details of the method are not suitable for discussion here, it suffices to mention, that the three different methods employed: direct diagonalization of rings, the Fisher model and the high temperature expansion method gave mutually agreeing results for the magnetic exchange in *catena*-[Mn(μ-F)(salen-5H)]. Analysis by the Fisher model has been done for several systems in the literature including the first complex with reported linear chain magnetism $Cs[MnCl_3]\cdot 2H_2O$. The model has also been used to estimated exchange coupling constants for magnetic interaction, M–M interaction for other transitions metals than manganese and especially a variety of different bridged systems as in the chromium with hydroxo-bridges, [171] such as the wheel $[Cr(OH)(OOCCMe_3)_2]_8$, [286] and the H_3O_2 bridged *trans*-$[Cr(py)_2(H_3O_2)_2]Cl$, [287]. The model of Bonner-Fischer was used on *e.g.* $Mn(COO)_2\cdot 2H_2O$ by Wagner and Friedberg. [288] The Bonner-Fisher expression will only reproduce experimental data for relatively high temperatues *e.g.* T/K∈[80-300]. [227] An improved fitting can be obtained by applying a corrected expression including terms representing inter-chain interaction and single ion anisotropy. Azide as bridging ligand is thoroughly studied both structurally and magnetically and frequently used as ligand in M(III)-salen systems. [216, 218, 229, 230, 232] The *catena*-[Mn(CN)(salen)] mentioned above differs somewhat from the other chain systems by consisting of one dimensional alternating chain of Mn(III) with $S = 1$ and $S = 2$ (carbon bound CN^- being a strong ligand field which stabilizes low-spin Mn(III). [228]

	g	$\mu_{eff,\,High}$ (300 K)	$\mu_{eff,\,Low}$ (T/K)	J cm^{-1}	zJ' cm^{-1}	Ref.
[Mn(OAc)(salen-5H)]	1.96	4.68	—	–1.4	≈ 0	[289]
	1.98	—	—	–1.54	—	[227]
[Mn(OAc)(salpen)]		—	1.65 (4.7)	–1.32	—	
[Mn(N_3)(salen-5H)]	2.05	—	—	–5.19	—	[232]
	1.98	—	—	–5.42	– 0.18	[227]
[Mn(N_3)(salen-5Br)]	—	—	—	—	—	[229]
[Mn(N_3)(salpn)]	—	—	—	—	—	
[Mn(NO_3)(salen-5H)]	2.01	4.79	3.80 (4)	–0.56	—	[279]
[Mn(NCS)(salpn)]	—	4.75	—	–3.2	—	
[Mn(HNCN)(salen-5Br)]	—	—	—	–0.99	–1.0	
[Mn(NCNCN)(salen)]	—	4.70	4.23 (5)	–0.24	—	

Table 8 Bonner-Fisher fitting parameters for Mn(III) chain systems

In Table 8 magnetic data for a range of chain complexes of Mn(III) have been tabulated. The values for the intra chain coupling constants have all been determined by use of the Bonner-Fisher model. It is worth to note that the compact fluoride bridges in [Mn(F)(salen-5H)] result in a sizable antiferromagnetic exchange compared to that observed for the polyatomic ligands. Thus, worries concerning the efficacy of fluoride in mediating magnetic exchange based on *e.g.* the Anderson model for magnetic exchange (Chapter 3) should not be given too much weight. It should also be mentioned that the Jahn-Teller effect, which has been a recurrent topic of this chapter also contributes to the efficient exchange interaction in the [Mn(F)(salen-5H)] chains by directing the σ-interacting spins along the chain direction. In conclusion, the interplay of electronic and structural effects are very beneficial the present chains, and fluoride is by no means to be dismissed as a useful bridge in tailoring magnetic systems, since it guides both geometric and electronic structure. A discrete analog of the 1D-systems discussed above, was obtained from MnF_3, PF_6^- and Me_3tacn in MeOH. The resulting orange compound is a dinuclear mono-fluorido bridged system, [MnF_2(Me_3tacn)(μ-F)MnF_2(Me_3tacn)]PF_6. The molecular structure of the complex is shown in Figure 29. Analogously to the chain structures, it is Jahn-Teller elongated along the Mn–F–Mn axis (∠Mn–F–Mn 180 °) as seen from the bond lengths: Mn–μF = 2.0498(10) Å; Mn–F_{cis} = 1.825(3)/1.826(4) Å. Similarly, the axial nitrogen donors have bond lengths of 2.267(5)Å and the others 2.090(5)/2.099(5) Å.

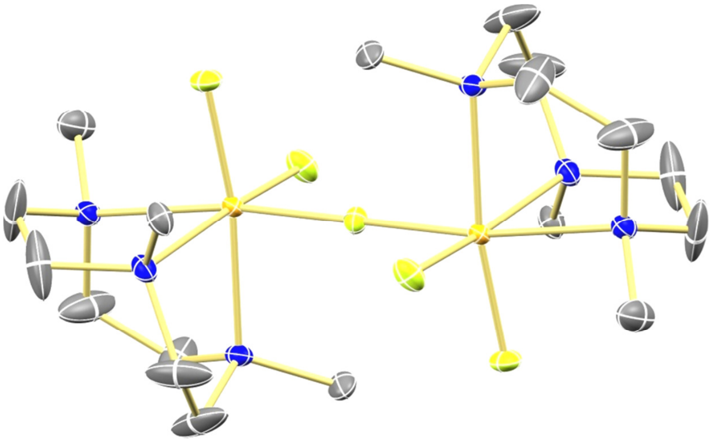

Figure 29 Molecular structure of [MnF_2(Me_3tacn)(μ-F)MnF_2(Me_3tacn)]PF_6

Hydrogen atoms and the PF_6^- counterion are omitted for clarity.

The magnetic susceptibility has been measured for the compound as has HF-EPR spectra and inelastic neutroc scattering spectra. The data could collectively be modelled with an exchange coupling constant of $J_{Mn\text{-}Mn}$ =32.7 cm^{-1}. The simplicity of the system invited an attempt at estimating the exchange coupling computationally. For this purpose a broken-symmetry calculation was performed using the experimental geometry, the PBE0 functional and a VTZ basis and the *ORCA* program package developed by Neese. [290] Quite satisfactorily, this standard approach yielded a computed exchange coupling constant of J_{calc}= 41.8 cm^{-1}. In conclusion the exchange via linearly bridging fluorido ligands on the Jahn-Teller axis is independent in magnitude of the nuclearity of the system and has a value of around 35 cm^{-1}.

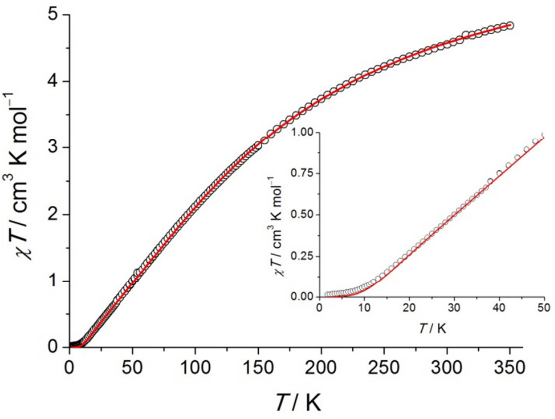

Figure 30 χT product and modelling thereof for [$MnF_2(Me_3tacn)(\mu\text{-}F)MnF_2(Me_3tacn)$]$PF_6$.

EXPERIMENTAL DETAILS

This section provides information on the details of the preparation and the characterization of the new one-dimensional fluoride-bridged complexes discussed in this chapter. A graphical overview of the complexes and reactivity discussed in the following is given in Scheme 2.

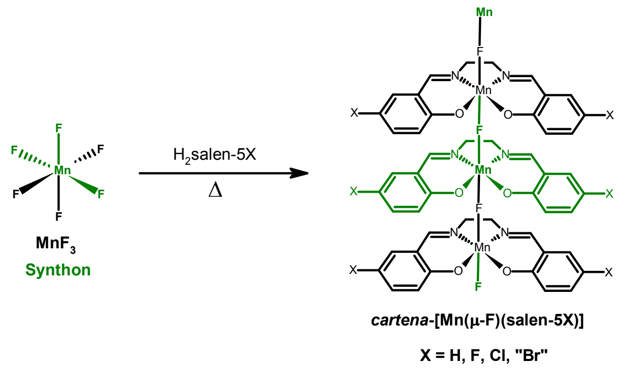

Scheme 2

- H_2salen-5R for R = H, F, Cl and Br
- *catena*-[Mn(μ-F)(salen-5R)] for R=H, F and Cl
- [Mn(F)(salen-5Br)]

General comments

The following preparations of ligands and complexes were all performed under normal laboratory conditions and chemicals and solvents were used as received without prior purification or drying.

The supplier and purity of a given chemical is specified where this chemical first appears in the preparation. A description of the techniques, equipment and detailed use in characterization is given in Appendix 2 “Instrumentarium”.

MnF_3 is a comercial and a relative unexpensive compound, which makes it usable as starting material. The compound may nevertheless be synthesized by a wide and diverse range of methods which may include proper fluorinations of MnF_2 or MnI_2 at 250 °C or alternativly and preferably fluorination of $(NH_3)_2MnF_5$. [111] Preparation by indirect fluorination involves evaporationg a solution of $Mn(IO_3)_2$ in BrF_3. Recently a method for preparation of MnF_3 at room temperature by fluorine oxidation of MnF_2 in anhydrous hydrofluoric acid has been reported. [291] Since MnF_3 is prone to hydrolysis by disproportionation to Mn(II) and MnO_2 when exposed to water the methanol used as solvent should have a relative low content of

water. However, the extent of hydrolysis was found to depend on reaction time to such way that it is possible to perform the synthesis in normal commercial-grade anhydrous methanol. The suitability of the methanol can be gauged before use by inspecting a suspension of solid MnF_3. Over time, only faint coloring of the solution phase should be observed.

Synthesis of H_2salen-5R for R=H, F, Cl, Br

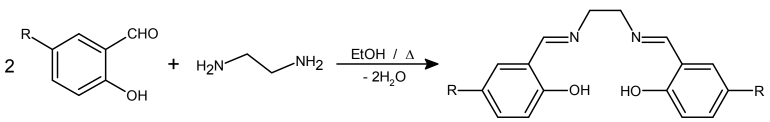

were R = H, F, Cl, Br.

R = H

The synthesis is based on the method from 1887 by Mason. [292]
Salicylaldehyde (122.30 g, 1.00 mol; Aldrich reagent 98 %) was placed i a 3 L beaker. Ethanol (96%; De Danske Spritfarbrikker) is added to a total volumen of 2 L. With stirring the mixture was heated to a temperature of approximately 60-65 °C. Subsequently ethan-1,2-diamine (30.031 g, 0.499 mol; Fluka, purum ≥ 99,0%) was added in small portions. An exothermic reaction takes place with formation of a intensly yellow-coloredlerd solution. After complete addition of ethan-1,2-diamine the beaker was left for crystallization. Soon, formation of a yellow crystaline product was observed. Precipitation is practically complete before the mixtures has reached room temperature. The product was isolated on a large a sintered glass funnel (No. 3). The product was dried by suction and washed by ice cold 96% ethanol. The product was recrystalized from ethanol (66.7 $g \cdot L^{-1}$) by heating to boiling and standing for 3 days.

Yellow crystal flakes, yield: 89.2 %.

- Elemental analysis: Calcd. (found) (%) for $H_{16}C_{16}N_2O_2$: H: 6.01 (5.76), C 71.62 (71.73), N: 10.44 (10.45).
- FT-IR (cm^{-1}): 3051(w), 3009(w), 2900(w), 2868(w), 2557(b), 1940(w), 1678(s), 1630(m), 1608(s), 1576(s), 1495(m), 1454(m), 1417(m), 1371(m), 1316 (w), 1281(s), 1248(m), 1219(m), 1199(m), 1149(s), 1113(m), 1041(s), 1020(s), 972(m), 936(w), 898(w), 855(s), 774(s), 748(s), 741(m), 647(m), 560(m), 472(m), 432(m).

R = F

The same general procedure as described for H_2salen was used. 5-fluor-2-hydroxybenzaldehyde was obtained from FluoroChem.

Yellow crystals, yield: 95.9 %.

- Elemental analysis: Calcd. (found) (%) for $H_{14}C_{16}N_2O_2F_2$: H 4.64 (4.53), C 63.15 (63.12), N 9.21 (9.18)
- FT-IR (cm^{-1}): 3081(w), 2939(w), 2910(w), 2853(w), 2611(b), 1890(w), 1722(w), 1633(s), 1580(m), 1525(s), 1497(w), 1463(w), 1453(w), 1440(m), 1396(m), 1364(m), 1319(m), 1271(m), 1245(m), 1224(m), 1183(m), 1139(s), 1040(s), 974(m), 958(m), 862(m), 824(s), 780(s), 669(m), 577(m), 516(w), 470(m), 463(m), 437(w), 402(m).

R = Cl

The same general procedure as described for H_2salen was used. 5-chlor-salicylaldehyde (98%) was obtained from Aldrich.

Yellow crystals, yield: 94.2 % .

- Elemental analysis: Calcd. (found) (%) for $H_{14}C_{16}N_2O_2Cl_2$: H 4.18 (4.15), C 56.99 (57.34), N 8.31 (8.34).
- FT-IR (cm^{-1}): 3081(w), 2941(w), 2904(w), 2850(w), 2624(b), 1991(w), 1899(w), 1733(s), 1631(s), 1572(w), 1482(s), 1395(m), 1361(s), 1320(w), 1273(s), 1225(w), 1208(w), 1196(w), 1180(m), 1139(w), 1120(w), 1085(w), 1034(s), 974(m), 959(w), 919(w), 868(w), 823(s), 776(s), 706(s), 669(w), 644(s), 577(w), 563(s), 500(w), 470(m), 438(m), 402(m).

R = Br

The same general procedure as described for H_2salen was used. 5-brom-salicylaldehyde (98%) was obtained from Aldrich.

Yellow crystals, yield: 93.4 %.

- Elemental analysis: Calcd. (found) (%) for $H_{14}C_{16}N_2O_2Br_2$: H 3.31 (3.13), C 45.10 (45.12), N 6.57 (6.42).
- FT-IR (cm^{-1}): 2631 (b), 1902(w), 1631(s), 1565(m), 1472(s), 1391(w), 1360(s), 1307(w), 1291(w), 1274(s), 1240(w), 1217(w), 1183(m), 1126(w), 1114(w), 1078(m), 1032(s), 977(m), 912(w), 895(w), 823(s), 776(s), 691(m), 627(s), 557(s), 479(m), 429(w).

Synthesis of *catena*-[Mn(μ-F)(salen-5H)]

$$MnF_3 + H_2\text{salen-5H} + 2\ Et_3N \xrightarrow[\Delta]{MeOH,\ H_2O} \textit{cartena}-[Mn(\mu\text{-}F)(\text{salen-5H})] + 2\ [Et_3HN]F$$

H_2salen-5H (1.34 g; 5.0 mmol) was partially dissolved in methanol (40 mL, Lab Scan Anhydroscan) in a Erlenmeyer flask (100 mL) and added solid manganese(III)fluoride (0.55 g, 4.91 mmol; Strem 98%) and triethylamine (1.02 g, 10.08 mmol; Merck - for synthesis). Mixing of reactants resulted in an immediate color change yielding a brownish suspension. The heterogeneous reaction mixture was heated to reflux for 35 min. added water (2.5 mL) and subsequently heated for 5 more minutes.
The intensely brown-colored reaction mixture was allowed to cool slowly to room temperature (ca. 25 min.) before being filtered through a paper filter. The brown solution was transferred to a Erlenmeyer flask (500 mL) and ^{t}BuOMe (250 mL; Sigma-Aldrich Chromasolv) was added drop-wise with simultaneous stirring. The addition time was 2 h. and 35 min. The brown crystalline product was loosened from the sides of the Erlenmeyer flask with a plastic spatula and isolated by filtration through a sintered glass funnel (No. 4), washed with ^{t}BuOMe (50 mL) and dried in a dynamic vacuum.

Recrystallization:
The product was recrystallized by dissolving *catena*-[Mn(μ-F)(salen-5H)] (1.01 g) in MeOH (100 mL). The resulting solution was filtered through a filter with pore size 0.45 μm and poured into a crystallization disc (115 mm Ø, volumen 500 mL) and placed ion a desiccator together with ^{t}BuMeO (350 mL). The desiccator was evacuated until visible boiling of the ether was observed. The time of diffusion was several weeks (Nevertheless, equilibrium seems established after ca. one week)

Yield of brown crystals 1.14 g (68 %)
- Elemental analysis: Calcd. (found) (%) for $H_{14}C_{16}N_2O_2F_1Mn_1$: H 4.15 (4.04), C 56.48 (56.39), N 8.23 (8.22), F 5.58 (5.38), Mn 16.15 (16.41).
- FT-IR (cm^{-1}): 3428(b), 1649(s) ν_{str}(CH=N), 1599(s), 1541(s), 1446(s), 1334(m), 1297(s) ν_{str}(C-O), 1195(m), 1147(m), 1127(m), 1029(m), 902(m), 744(s), 732(s), 630(m), 584(m), 460(s) ν_{str}(Mn-N/O). Assignment based on [293]
- UV/vis (MeOH): λ[nm] $\varepsilon[10^3\ M^{-1}\cdot cm^{-1}]$: 216 (33.57), 236 (37.58), 281 (17.08), 307 (12.35), 349 (60.45), 397 (46.76).
- MS(FAB+): *m*-NBA m/z=340.04 [M^+], 321.08 [M-F].
- MS(FBA–): *m*-NBA m/z=339.42 [M-H].

Synthesis of *catena*-[Mn(μ-F)(salen-5F)]

$$MnF_3 + H_2salen\text{-}5F + Et_3N \xrightarrow[\Delta]{MeOH} cartena-[Mn(\mu\text{-}F)(salen\text{-}5F)] + 2\,[Et_3HN]F$$

H_2salen-5F (0.625 g, 2.05 mmol) was placed in a Erlenmeyer flask (100 mL) and dissolved in methanol (50 mL, Lab Scan Anhydroscan) which initially was purged with nitrogen for 5 min. Solid manganese(III)fluoride (0.230 g, 2.05 mmol; Strem 98%) and triethylamine (0.415 g, 4.10 mmol; Merck - for synthesis) was added. The reaction mixture was heated to boiling in a nitrogen atmosphere for 30 min. During the time of heating a light brown solution with a precipitate was formed. The reaction mixture was allowed to cool slowly to room temperature for ca. 1 hour with simultaneous stirring before being filtered through a sintered glass funnel (No. 4) in a atmosphere of nitrogen. The filtration gave a brown filtrate and a yellow-green retentate. The retentate was dried by passing a nitrogen flow through the compound for 2 hours.

Yield of yellow-green powder: 0.050 g.

- Elemental analysis: Found (%) H 2.87, C 20.05, N 5.82.
- FT-IR (cm^{-1}): 2939(w), 2910(w), 2853(w), 2362(w), 1890(w), 1721(w), 1633(s), 1580(m), 1497(s), 1464(w), 1440(w), 1396(w), 1364(m), 1319(w), 1271(m), 1245(m), 1224(m), 1183(w), 1139(s), 1040(s), 974(m), 959(m), 862(m), 825(s), 781(s), 714(w), 669(m), 577(m), 516(w), 463(m), 401(s).

The brown filtrate from the filtration was transferred to a Erlenmeyer flask (500 mL) and added ^{t}BuOMe (Sigma-Aldrich Chromasolv) at regular speed (400 mL), which caused precipitation of a semi-crystalline product. After 5 min. additionally ^{t}BuOMe (100 mL) was layed on top of brown suspension and left for 30 min. for agglomeration. This resulted in dark brown crystals which could be isolated by filtration through a sintered glass funnel (No. 3) washed with ^{t}BuOMe (2·10 mL) and subsequently dried in a dynamic vacuum. Yield: 0.655 g (85 %).

Recrystallization:
In two consecutive recrystallizations. First, ^{t}BuOMe (250 mL) was added from a dropping funnel to a methanolic solution of raw *catena*-[Mn(F)(salen-5F)] (0.026 g·mL^{-1}) over a period of 45 min. The precipitated yellow-brown powder was isolated by filtration through a sintered glass funnel (No. 4). It was found to be greatly affected by static electricity, which complicated the handling. The powder was redissolved in MeOH (50 mL) and treated as described above by adding ^{t}BuOMe (400 mL) to the mixture followed after 5 min. by layering with a top layer of ^{t}BuOMe (100 mL).

Yield of brown crystals: 0.543 g (70 %).

- Elemental analysis: Calcd. (found) (%) for $H_{12}C_{16}N_2O_2F_3Mn_1$: H 3.22 (3.10), C 51.08 (50.90), N 7.45 (7.34).

Synthesis of *catena*-[Mn(μ-F)(salen-5Cl)]

$$MnF_3 + H_2salen\text{-}5Cl \xrightarrow[\Delta]{MeOH} \textit{cartena} - [Mn(\mu\text{-}F)(salen\text{-}5Cl)] + 2\ HF$$

H_2salen-5Cl (0.514 g, 1.52 mmol) and solid manganese(III)fluoride (0.179 g, 1.60 mmol; Strem 98%) was placed in a Erlenmeyer flask (100 mL) made of hard heat-resistant plastic and added MeOH (30 mL, Lab Scan Anhydroscan). The reaction mixture was heated on a waterbath with stirring to boiling in a nitrogen atmosphere for 30 min. While still lukewarm the reaction mixture was filtered through a sintered glass funnel (No. 4) into a Erlenmeyer flask (250 mL) made of hard plastic. From a dropping funnel, Et_2O (150 mL; Aldrich Puriss p.a.) was added drop-wise with simultaneous stirring. Precipitation of a yellow product commenced after addition of approx. 25 mL Et_2O. The product – susceptible to static electricity, was isolated on a sintered glass funnel (No. 4) and dried in a dynamic vacuum, before extraction on the filter with MeOH (30 mL). The solution thus obtained was placed in a desiccator together with Et_2O (100mL) for slow diffusion. A portion of the crystals with quality for single crystal diffraction could be harvested before the precipitation of a powder began.

Yield powder: Not determined because of the powders intrinsic susceptibility to static electricity.

- Crystals: Elemental analysis: Calcd. (found) (%) for $H_{18}C_{17}N_2O_4F_1Cl_2Mn_1$ ([Mn(F)(salen-5Cl)]·H_2O·MeOH): H 3.95 (3.13), C 44.47 (43.77), N 6.10 (6.01).
- Powder: Elemental analysis: Calcd. (found) (%) for $H_{12}C_{16}N_2O_2F_1Cl_2Mn_1$ ([Mn(F)(salen-5Cl)]): H 2.96 (2.88), C 46.97 (46.85), N 6.85 (6.60).
- FT-IR (cm^{-1}): 2921(w), 2359(w), 1971(w), 1641(s), 1597(w), 1529(m), 1452(s), 1421(m), 1370(s), 1326(w), 1287(s), 1237(w), 1203(w), 1178(s), 1134(w), 1095(w), 1046(m), 988(w), 966(w), 914(w), 896(w), 875(w), 827(s), 796(s), 746(w), 735(w), 707(s), 659(s), 596(m), 549(m), 486(s), 463(s).

Reprecipitation:

The raw product (0.200 g) was dissolved almost completely in MeOH (50 mL, Lab Scan Anhydroscan) was reprecipitated: The resulting yellow brown solution was filtered first through a paper filter and subsequently through a teflon filter with pore size 0.45 μm. The solution was evaporated to approx. half volume and added Et_2O until precipitation of a curry-colored solid took place. The product was isolated on a sintered glass funnel (No. 4) and dried in a dynamic vacuum.

- Elemental analysis: Calcd. (found) (%) for $H_{12}C_{16}N_2O_2F_1Cl_2Mn_1$ ([Mn(F)(salen-5Cl)]): H 2.96 (2.72), C 46.97 (46.54), N 6.85 (6.58).
- FT-IR (cm^{-1}): 2930(w), 2358(w), 1734(w), 1644(s), 1598(w), 1532(m), 1454(s), 1422(m), 1371(s), 1326(w), 1288(s), 1237(w), 1203(w), 1179(s), 1135(w), 1097(w), 1047(m), 988(w), 966(w), 914(w), 896(w), 875(w), 841(w), 827(s), 796(s), 746(w), 734(w), 708(s), 660(s), 597(m), 549(m), 486(s).

Synthesis of *catena*-[Mn(μ-F)(salen-5Br)]

$$MnF_3 + H_2salen\text{-}5Br \xrightarrow[\Delta]{MeOH} [Mn(F)(salen\text{-}5Br)] + 2\ HF$$

H_2salen-5Br (1.902, 4.46 mmol) and solid manganese(III)fluoride (0.502 g, 4.48 mmol; Strem 98%) was placed in a Erlenmeyer flask (250 mL) made of hard heat-resistant plastic and added MeOH (183 mL, Lab Scan Anhydroscan). The reaction mixture was heated on a waterbath with stirring to boiling in for 30 min. Precipitation of a moderate amount of brown solid was observed during the heating. After cooling to room temperature, additional MeOH (55 mL) was added followed by heating on a water bath. The reulting solution was left to cool slowly in the water bath, filtered through a sintered glass funnel (No. 4) into a Erlenmeyer flask (250 mL) made of hard plastic before being sealed and placed at 5 °C over night. The precipitated fibrous crystalline brown product was isolated on a sintered glass funnel (No. 4) and dried in a dynamic vacuum.

Yield of brown crystals 0.671 g (30 %).

- Elemental analysis: Calcd. (found) (%) for $H_{14}C_{16}N_2O_3F_1Br_2Mn_1$ ([Mn(F)(salen-5Br)]·H_2O): H 2.73 (2.28), C 37.24 (37.78), N 5.43 (5.37).
- FT-IR (cm^{-1}): 3398(w), 2358(w), 1969(w), 1749(w), 1623(s), 1591(m), 1529(m), 1455(m), 1417(w), 1372(m), 1326(w), 1285(s), 1268(s), 1201(w), 1180(m), 1138(w), 1086(w), 1041(w), 1006(w), 973(w), 885(m), 838(m), 826(m), 805(m), 772(m), 733(m), 714(m), 686(s), 655(s), 600(m), 550(m), 509(m), 492(s), 48(s)1, 462(s), 445(m), 407(s).

Crystals for X-ray diffraction of *catena*-[Mn(μ-F)(salen-5R)] for R=H, F, Cl

***catena*-[Mn(μ-F)(salen-5H)]**
Crystals of quality for single crystal diffraction have been shown to best be grown by slow diffusion of medium volatile ether as ^{t}BuOMe (Sigma-Aldrich Chromasolv) into a methanolic solution. The volatile Et_2O (Sigma-Aldrich Puriss p.a.) can be used but produces crystals of lowe quality.
catena-[Mn(μ-F)(salen-5H)] (0.090 g) was placed in a crystallization disk and completely dissolved by addtion of MeOH (20 mL, Lab Scan Anhydroscan). The disk is placed with a baker of ether in a desiccator without any drying material. The desiccator is evaluated with water suction. The pumping was stopped when visible boiling of ether was observed.

***catena*-[Mn(μ-F)(salen-5F)]**
Slow diffusion of ^{t}BuOMe in a desiccator to a solution of raw *catena*-[Mn(μ-F)(salen-5F)] in MeOH (3.42 $g{\cdot}L^{-1}$) gave, after 2 days, a homogeneus crystalline product. The crystals had a tendency to be intergrown, but crystals with quality for single crystal diffraction could be harvested. The crystals were isolated by decantation from the mother liquor and transferred to a sintered glass funnel (No. 4), washed with ^{t}BuOMe (2·10 mL), and dried in a dynamic vacuum.

Yield of brown crystals: ~78 %.
- Elemental analysis: Calcd. (found) (%) for $H_{12}C_{16}N_2O_2F_3Mn_1$: H 3.22 (3.10), C 51.08 (51.11), N 7.45 (7.38).
- FT-IR (cm^{-1}): 2923(w), 1734(w), 1650(s), 1548(s), 1458(s), 1430(s), 1369(m), 1323(w), 1288(s), 1249(s), 1213(s), 1141(s), 1085(w), 1042(m), 969(w), 961(w), 864(s), 835(w), 814(s), 800(s), 777(s), 733(w), 675(w), 615(m), 596(m), 556(s), 518(s), 469(m), 437(s), 404(s).

***catena*-[Mn(μ-F)(salen-5Cl)]**
See preparation details.

CHAPTER 5

ALKALI METAL CATION COMPLEXATION BY Cr(III) FLUORIDO COMPLEXES

INTRODUCTION

As cations, the alkali- and alkaline earth metals ions (hereafter referred to as *s*-blok ions) are generally considered as spectator/counter ions and perceived as being without influence on the chemical system. This description is often true and it is frequently meaningful to consider or discuss the chemistry of salts of coordination compounds without explicitly taking *s*-block ions into account. In the litterature one can find examples of structurally characterized compounds in which *s*-blok ions are included "unexpectedly" as an integrated chemical entity of the structure itself. These situations are sometimes regarded as curious artifacts. However, that it should be so in general cannot be taken for granted.

Based on the general qualitative considerations given in Chapter 2, with particular focus on the principles of HSAB reactivity, it must as a starting point be expected, that *s*-blok ions, which are classified as hard ions, have opportunity for energetically favorable interactions with ligands containing hard ligator aoms *e.g.* O and F.

The reason why such interactions are not dominating the chemistry of the *s*-block elements, is that most of the reactions involving *s*-blok ions are performed in water or water-containing solvents. The oxygen ligtors from water can coordinate to the *s*-blok ion, not only in the first coordination sphere but also in additional solvation shells forming semi-cluster structures, $[A(H_2O)_n]^{+/2+}$ whereby the *s*-blok ion is, to a certain extent, shielded from further coordination. The possibility for successful coordination of a hard ligand to a *s*-blok ion therefore requires an overcome of the solvation energy, of the hydrated ion to break the second coordination sphere (the shell) of water ligands in the vicinity of the *s*-blok ion and secondly competition with the bond energy for direct ligand substitution of the coordinated water in the first coordination

sphere. On this basis, synthesis of systems ontaining *s*-blok ions bridged by unsupported fluorido ligands to a transition metal ion may seem to depend more on luck rather than rational considerations.

The synthon approach

One possible approach to overcome the above mentioned synthesis-problem can be based on the general idea presented in Chapter 1 where the fluorido ligand is anchored to a kinetically robust complex (*e.g.* of Cr(III)) and will thus act as a synthon for the fluoride containing part of the final (unsupported) fluorido-bridged complex. This anchoring is especially important in the present situation due to the otherwise strong hydrogen bonding interaction between the free fluoride ion and the aqueous solutions which is likely to result in a modification of its reactivity. In addition, the anchoring of the fluorido ligand could guide formation of a stereochemically desirable product. To aid structural studies, this could be, if possible, a product of high-symmetry which generally favors crystallization. The choice of anchor in the synthon makes it possible to tune the properties of the fluorido ligand. Stereochemistry and product formation can similarly be changed by the number of fluorido ligands in the synthon, which in priciple can be varied between 1 and 6 (or even 8). A reasonable number of fluorido ligands in a building block seems to be 2 or 3 if a controllable relationship between the overall synthon charge and bridging/cluster stereochemistry of the product is desired. Here a range if mono-cationic difluorido synthons have been investigated. These are all of the type *trans*-$[Cr(py)_4F_2]^+$, *cis*-$[Cr(L'')_2F_2]^+$ for L''=phen, bpy, while the neutral triflurido synthons used are $[Cr(L''')F_3]$ for L'''=terpy, Me_3tacn.

As will be shown in this chapter alkali metal ions largely interacts with the robust Cr(III) fluoride synthon through second sphere coordination in solution. This interaction may, by suitable choice of first coordination sphere synthon, allow for isolation of *s*-block complexes with a genuine coordinative dative bond from bridging fluoride ligands. Some, structurally unprecedented systems containing the *s*-blok ions Na(I), Li(I) and Cr(III) bridged by the hard fluorido ligand, have thus been isolated and characterized. The product formed for the two different *s*-blok ions are quite different: For sodium, a 1D, infinite coordination polymer, *trans*-*catena*-poly$[Na(H_2O)_4(\mu\text{-}F)Cr(py)_4(\mu\text{-}F)](HCO_3)_2$ results, while for lithium discrete (0D) complexes of three different cations, *trans*-$[Cr(py)_4F(\mu\text{-}F)\{Li(H_2O)_{n=3,4}\}_{m=0,1}]Cl_5{\cdot}6H_2O$, were obtained. Despite the simple composition of the compounds synthezied, it is noteworthythat no immediate counterparts are found in the literature.

In connection with the synthesis it was observed that a significant interaction takes place between the Cr(III) fluorido complexes and the alkali metal ions in aqueous solution. This is visually detecable as hypsochromic shifts of the ligand field spectra of the chromium complexes. These interactions in solution have direct implications for the other studies of this

project regarding interactions between transition metals and other metal centers through fluorido bridges, and it was therefore attempted to further investigate these interactions.

SECOND SPHERE COORDINATION IN Cr(III) FLUORIDO COMPLEXES

Despite that several of the employed synthons are positively charged they react willingly with alkali metal cations. Based on electrostatic considerations this reaction behaviour could have been expected to be energetically unfavourable. However, spectral titration in solution shows that the formation of the complexes occurs with reasonably high formation constants and that the driving force for the reactivity is not just solid phase stabilizations.

Introduction

In general terms, an entity in coordination chemical context can be considered as having a number of different spheres available for coordination in which the incoming ligands interact with the cental ion in various ways. These coordination spheres are divided into the following:

- **First coordination sphere**
  In this sphere a number of ligands bind through coordinative dative bonds to the central metal ion and form the coordination polyhedron around the metal centre.

- **Second coordination sphere**
  To this sphere belong the more weakly bonded interactions of the coordinated ligands with other entities of the chemical system. The interaction normally, but not necessarily, involves non-coordinative dative bonding such as hydrogen bonding and Coulomb attraction. [294]

An illustration of both the first and second coordination sphere around the synthon *trans*-$[Cr(py)_4F_2]^+$ is shown on Figure 31.
From this qualitative division of the complete coordination entity it is expected that the bond strength between the central metal ion and ligand on one side and the ligand and other entities are significantly different. As a result the interaction with the second coordination sphere can figuratively be compared with the (reversible) binding of a substrate for the a suitable receptor.
Second coordination sphere complexation can obviously give rise to extented cluster structures of a varying and sometimes large sizes. In this respect, such systems has be studied within the area of metallosupramolecular chemistry focusing on structural and receptor properties. In this context, second sphere coordination is used in explanation of how simple complex ions such as $[Co(phen)_3](BF_4)_3 \cdot H_2O$, *trans*-$[Co(en)_2Cl_2]BF_4$ and *cis*-$[Co(phen)_2CO_3](C_6F_5COO) \cdot 6H_2O$ are joined together in the solid state beyond the purely electrostatic contribution. [295-297] A central second sphere coordination contribution and perhaps the most dominating one in this

kind of systems is hydrogen bond formation (D–H···A), involving both cations, anions and possibly solvents, but also other interactions as the aromatic C–H···π and π···π stacking may be of importance. In connection with the development of receptor systems for recognition of anions one of the requirements to a final receptor system may be that the substrate repeatingly can be absorbed and released. The cobalt systems mentioned above display only second sphere coordination with respect to the anions in the solid phase which is likely to complicate receptor-type application prospects. By some practitioners of the field it is hoped for that, control of hydrogen bond formation and use of this for systematic molecular assembly, will be come a future possibility. Despite some progress towards energetic and stereochemical controlby this approach, it is still a challenging task and much research is still required.

As already noted, second sphere coordination is not only a phenomenon of importance only in the solid phase. Phenomena such as solvatochromism occur in solution, where a pronounced change in position, and possibly intensity, of an electronic absorption/emission band, accompanies a change in the polarity of the medium. [298] Kaizaki *et al.* have investigated the second sphere complexation for particularly fluorido complexes in detail and tried to describe the interaction using the AOM formalism. [299-303]

However, let set out by discussing some of the examples where fluorido complexes have been shown to interact with *s*-block ions in the solid state.

FLUORIDO BRIDGES BETWEEN *d*- AND *S*-BLOCK METALS

The 3*d*-(μ-F)-n*s* bonding motif

The bonding motif, n'*d*–(μ-F)–n*s* of a transition metal ion bridged by a fluorido ligand to a alkali metal ion is relativily rarely observed. The total number of structurally characterized compounds of this type for Group 1 is about 50, which Li: 8, Na: 32, K: 5, Rb: 1 and Cs: 2. A general characteristic of these systems are a high polynuclear complexity and that the F–A bond for several cases has more character of a packing phenomenon than resembling a polynuclear complex. The transition metals in these systems must often be sought in the early transition metal groups of The Periodic Table. With the exception of the complexes described in this thesis all Li(I) complexes structurally characterized contain one of the Group 4. elements Ti or Zr, whereas the majority of Na(I) complexes contain Group 4. or Group 6. elements Ti, Zr, Mo, W or in a few cases V, Re, Cr, Ni and Cu.

The structurally characterized systems can be classified into two main groups. On the one hand polynuclear systems often belonging the sub classes of organometallic compounds *e.g.* $[(C_5Me_5)_6Ti_6Na_7F_{19}\cdot 2.5thf]$, $[Na\{Ti_2(C_5Me_5)_2F_7\}]$ and $[NaTi_6(C_5Me_5)_5F_{20}(H_2O)]\cdot thf$, [304, 305] and polyoxofluorometallates *e.g.* $[Mo_8Na_2O_{26}F_2(H_2O)_4]^{4-}$, $[\{Li\ or\ Na\}_2Cs_4Mo_6O_{18}F_6]\cdot 6H_2O$ and $[Na_2(Me_4N)_3Mo_7O_{22}F_3]\cdot 6H_2O$, [306, 307]. The occurrence of the organometallic systems is not

surprising, since this chemistry is frequently associated with solvents of low-dielectricity constants, which favors ion-pairing.

The second main group of compounds are all based on the cyclic polyethers (crown ethers) introduced by Nobel Prize winner Pedersen in 1967, who pawed the way for investigation of *s*-blok coordination chemistry with the introduction of this ligand type. [308, 309] These cyclic polyethers can act as a hosts for *s*-blok cation with an affinity directly related to the size giving Li(I) high affinity for 12-crown-4, Na(I) for 15-crown-5, and K(I) for 18-crown-6. The group containing these co-ligands, feature the simplest examples of fluorido bridging between transition metals and alkali metal ions. In contrast to the above-mentioned systems, practically all of these complexes are discrete with the alkal metal ion incorporated into a suitable crown ether. Even though Li(I) has affinity for 12-crown-4, no structurally characterized crown ether complexes of Li(I) are known. In contrast, 14 of the known Na(I) complexes are based on this type of ligand, similarly for K(I) with 3 known examples. Several of these crown ether complexes containing $[Na\text{-}15\text{-}crown\text{-}5]^+$ or $[K\text{-}18\text{-}crown\text{-}6]^+$ are structurally characterized by Dehnicke *et al.* Examples are given in Table 9.

	**$[Na(15\text{-}crown\text{-}5)]^+$**	Ref.	**$[K(18\text{-}crown\text{-}6)]^+$**	Ref.
**2nd period**	$[MoF_3(NCl)(\mu\text{-}F)_2]^-$	[310]		
	$[Mo(NO)(Cl)(F)(\mu_3\text{-}F)(\mu\text{-}F)_2]^{2-}$	[311]		
	dimeric-$[MoNF_4]^-$	[312]		
	$[Mo(\mu\text{-}F)_2Cl_2(N_3S_2)]^-$	[313]		
	$[\{MoF_{4-(0,1)}(\mu\text{-}F)_{1+(0,1)}\}_2(\mu_{1,3}\text{-}NSN)]^{2-}$	[314]	$[\{Mo(\eta^3\text{-}C_3H_4(Me)(CO)_2\}(\mu_2\text{-}F)_3]^-$	[315]
**3rd period**	$[WF_3(NCl)(\mu\text{-}F)_2]^-$	[316]	$[WF_3(NCl)(\mu\text{-}F)_2]^-$	[317]
	$[WF_3(Ph\text{-}(C{\equiv}C)_2\text{-}SiMe_3)(\mu\text{-}F)_2]^-$	[318]		
	$[WF_3(PhC{\equiv}CPh)(\mu\text{-}F)_2]^-$	[319]		
	$[WF_3(PhC{\equiv}CH)(\mu\text{-}F)_2]^-$	[320]	$[WF_2(PhC{\equiv}CH)(\mu\text{-}F)_3]^-$	[321]

Table 9 $[Na(15\text{-}crown\text{-}5)]^+$ and $[K(18\text{-}crown\text{-}6)]^+$complexes of fluorido complexes.

In addition to the $[Na(15\text{-}crown\text{-}5)]^+$ complexes of Group 6. metals given Table 9, also the Group 7. complexes $[Re(NO)_2(Cl)_2(\mu\text{-}F)_2]^-$, [322] and $[Re(NO)(MeCN)(Cl)_2(\mu\text{-}Cl)(\mu\text{-}F)]^{2-}$, [323] have been reported. As a consequence of the synthetic methods for the complexes in Table 9, these systems cannot be seen as synthons for the fluorido ligand in the context of the meaning used in this thesis. The syntheses all follow similar procedures where a suitable transition metal-containing starting material of Mo, W with labile ligands is brought to react with NaF and 15-crown-5 in acetonitrile solution. The ligand sphere is fully or partially substituted with fluoride. The substitution pattern can be identical for Mo and W as witnessed by the first example for each of the metals in Table 9. Of the complexes listed in Table 9 the bridging motif is completely dominated by double bridges, $M\text{–}(\mu\text{-}F)_2\text{–}Na$. Only for $[\{MoF_{4-}$

$_{(0,1)}(\mu\text{-F})_{1+(0,1)}\}_2(\mu_{1,3}\text{-NSN})]^{2-}$ is an additional unsupported fluorido bridge found. The bond angle for the $-(\mu\text{-F})_2-$ bridges are 102.3(5) ° and 113.8(5) °, respectively, while the unsupported bridges has a angle of 127.0(7) °. This last angle is so different from the prefered near linearity of fluorido bridges, that it cannot be excluded that in reality the bridge is an asymmetric double bridge. For [Na-crown-5][Mo(μ-F)$_2$Cl$_2$(N$_3$S$_2$)], two analogous complexes are known: the corresponding Mo-complex, but with [Na-benzo-15-crown-5]$^+$, [324] as a counter ion and the related vanadium complex, [Na-benzo-15-crown-5][V(μ-F)(μ-Cl)Cl(N$_3$S$_2$)], [325]. The latter systems are distinguished by their fluorido bridge being supported by a second fluorido bridge and a chlorido bridge, respectively. By comparison of the bond angles in the [Na-benzo-15-crown-5] complexes: ∠Na–F–Mo: 107.4(2) °, ∠Na–F–V: 122.58(7) °, ∠Na–Cl–V: 85.63(4) ° and in the [Na-crown-5] complex: ∠Na–F–Mo: 103.8(1) °, ∠105.7(1)°, it is seen that the fluorido bridge in conjunction with the chlorido bridge achieves maximum angle of the three. By considering the three system's average bridge angle (Mo-complexes: 107.4(2) ° and 104.75(1) °, V-complex: 104.22(6) °) is it seen that these averages in practice are identical in spite of the different transition metal center. It therefore seems reasonable to view this as a manifestation of the general tendency for fluorido bridges to tend towards approximate linear bridging if possible (*cf.* Chapter 2 and Chapter 4 in connection with *catena*-[Mn(μ-F)(salen-5R)]). The statement about linear fluorido bridges should not be taken as an ultimate requirement for bridging to occur, but an ideal bond angle the system will tend towards in balance with other sterical and electronic requirements. This situation is one of the main reasons for why it becomes possible to speak of an actual product design in connection with the fluoride synthon approach. Although the new Na(I) and Li(I) systems introduced above, differ structurally, (see structure description in subsequent section), they both fulfil the initial expectation, based on the selected synthon, of beeing either discrete or a chain and that with very large bond angles around fluoride.

SYNTHETIC STRATEGY FOR 3*d*-(μF)-n*s* BRIDGED SYSTEMS

That this type of complexes can be established may partly be rationalized based on the HSAB principle, identifying a favorable interaction between the hard fluorido ligand and hard cations. This type of interaction is well known for hard *p*- and *d*-block cations (see Chapter 6) but should in principle be equally valid for the hard *s*-block cations. In the specific situations, it is noted that interaction occurs between two cationic entities indicating that the interaction must be of a certain strength.

Synthons with two fluorido ligands, *cis*/*trans*-[Cr(L')$_2$F$_2$]

As previously indicated, the actual choice of synthon and the configuration of the fluorido ligands in it constitutes the initial, important step in product design by the synthon approach.

The compound selected, as the starting material for the investigation of interactions with the alkali metal ions was *trans*-[Cr(py)$_4$F$_2$]NO$_3$. The cationic chromium complex has been structurally characterized and has a the well defined geometry with the two fluorido ligands are located in *trans* position. No claims of, nor evidence for the exsistence of the *cis* isomer have been made. The *trans* configuration separates the potential second sphere interactions of the two fluorido ligands and reduce the risk of interference or formation of complicated network structures. A graphical representation of the likely first and second coordination sphere of *trans*-[Cr(py)$_4$F$_2$]NO$_3$ in alkalimetal ion containing solution is shown in Figure 31.

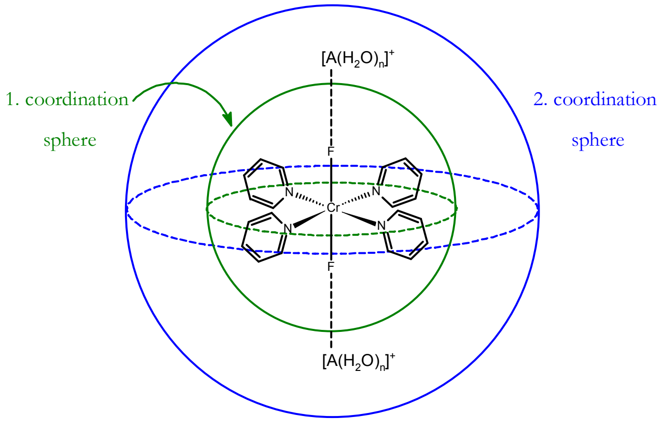

Figure 31 Representation of 1. and 2. coordination sphere around *trans*-[Cr(py)$_4$F$_2$]$^+$
[A(H$_2$O)$_n$]$^+$ represents general solvated alkali metal cation with A=Li, Na

It was observed that interaction between *trans*-[Cr(py)$_4$F$_2$]$^+$ and the alkali metal ion depends on several parameters. As expected, the choice of alkali metal cation, A=Li(I), Na(I) is important, but also of significance is the anion. This observation is in accordance with the studies regarding second sphere interaction between simple Co(III)-complexes and different anions. Reaction between *trans*-[CrF$_2$(py)$_4$]NO$_3$ and NaHCO$_3$ or LiCl, respectively in water yielded second sphere coordination leading to isolated products with different structures, as shown in (5.1) and (5.2).

- Reaction with Na(I):

$$\textit{trans}\text{-[CrF}_2\text{(py)}_4\text{]NO}_3 + 2\,\text{NaHCO}_3 \xrightarrow{\text{H}_2\text{O}} \textit{trans-cartena}\text{-poly[Na(H}_2\text{O)}_4(\mu\text{-F)Cr(py)}_4(\mu\text{-F)](HCO}_3)_2 + \text{NaNO}_3 \quad (5.1)$$

- Reaction with Li(I):

$$3\ \textit{trans}\text{-}[Cr(py)_4F_2]NO_3 + 5\ LiCl \xrightarrow{H_2O} \textit{trans}\text{-}[Cr(py)_4F(\mu\text{-}F)\{Li(H_2O)_{n=3,4}\}_{m=0,1}]Cl_5 \cdot 6H_2O + 3\ LiNO_3 \quad (5.2)$$

Certain characteristics of the syntheses are worth noting. Despite the fact that it is shown by spectroscopy and mass spectrometry that interaction in solution between the synthon and alkali metal ion is present, isolation is not “automatic” and driven by very low product solubilities. To achieve crystallization, considerable excess of alkali metal ions must be added. This can be seen as a result of a labile second sphere interaction wherein a continuously ligand exchange between the fluorido ligand on the synthon and the alkali metal ion take place. The position of the dynamic equilibrium is determined by a number of opposing effects including the repulsive electrostatic interaction between the cations, *trans*-$[Cr(py)_4F_2]^+$ and A(I) for A=Li, Na and attractive HSAB interaction between the hard F^- and Na(I) and solvation and further complexation of both cations. Another important attractive part of the interaction is the possibility of hydrogen bonding between both fluorido ligand, the coordinated water ligands and anions. The presence of hydrogen bonding and its non negligible influence on complex stability is indicated by the rapid formation of the original constituents *trans*-$[Cr(py)_4F_2]^+$ and solid $NaHCO_3$ when *trans-catena*-poly$[Na(H_2O)_4(\mu\text{-}F)Cr(py)_4(\mu\text{-}F)](HCO_3)_2$ is treated with methanol. This reaction support the description of the fluorido bridging between the two metal centers in term of an intermediate bonding situation between genuinely bridged first sphere coordination and more loose second sphere coordination.

Despite the similar reaction conditions, the resulting products set out in (5.1) and (5.2) differ significantly. With Na(I) the reaction give rise to a cationic infinite 1D coordination polymer in form of a linear chain consisting of *trans*-$[Cr(py)_4F_2]^+$ and $[Na(H_2O)_4]^+$ units connected through a linear μ-fluorido bridge. On the other hand reaction with Li(I) result in the formation of a simple 0D salt consisting of three different discrete cations all based on *trans*-$[Cr(py)_4F_2]^+$. In two of these cations a solvated Li(I) ions is coordinated through a linear μ-fluorido bridge forming a simple dimer. The number of water ligands coordinating to Li(I) differs in the two ions being respectively 3 and 4. In contrast to the infinite chain obtained in the reaction with Na(I), where both of the two available fluorido ligands in *trans*-$[Cr(py)_4F_2]^+$ are included in μ-F bridging only one of the fluorido ligands coordinates in case of Li(I). A reaction similar to that of Na(I) in which both fluorido ligands coordinates cannot be excluded. However, the concentration of Li(I) ions (ca. 2.5 M) necessary to initiate crystallization of the *trans*-$[Cr(py)_4F(\mu\text{-}F)\{Li(H_2O)_n\}_m]Cl_5{\cdot}6H_2O$ complex is so high that it is questionable whether formation of the "double" bridge complex is just a question of establishing a an appropriate concentration of alkali metal ion. Formation of these two complexes is initiated, as shown in Figure 31, by the establishment of a second sphere coordination between *trans*-$[Cr(py)_4F_2]^+$ and the hydrated alkali metal ion. The second sphere coordination requires a redistribution of the solvent sphere around the alkali ions possibly together with breaking a certain number of the coordinativebonds between the water ligands to the alkali metal ion. The degree by which the

redistribution of solvent sphere takes place depends, among other things by the ion size as well as the hydration energy of the alkali metal ion. The ion size increases generally down the group of alkali metals giving rise to increasing coordination number as found by the aqua ions which commonly ranges from tetrahedral four coordination in cases of Li(I) to six coordination for Na(I). [20] The effect of the change in ion size upon decent through the group is inversely related to the hydration energy of the ions. This energy coorespond to the change in Gibbs free energy when the (free) ion is brought from a vacuum to an aqueous solution with a significantly higher hydration energy for Li^+ (519 kJ mol^{-1}) than the rest of the group (Na^+: 406 kJ mol^{-1}, K^+. 322 kJ mol^{-1}). [20, 298] The product obtained by reaction with Na(I) ions corresponds well with the intuition of the inorganic chemist. The moderate hydration energy and intermediate ionic radius allow for a twofold ligand substitution forming the double fluorido bridge while maintaining coordination number and geometry around Na(I). By comparison with this, the reaction with Li(I) cations is more complex. The increased hydration energy of Li(I) is reflected by the parallel formation of two different cations in which Li(I) is coordinated by, respectively, three and four water ligands in addition to the fluorido bridges, *trans*-$[Cr(py)_4F(\mu\text{-}F)\{Li(H_2O)_{n=3,4}\}_{m=0,1}]^{(1+m)+}$. In case of *trans*-$[Cr(py)_4F(\mu\text{-}F)Li(H_2O)_4]^{2+}$ the hydration energy is sufficient for it to be energetically favorable to expand the coordination number from four in $[Li(H_2O)_4]^+$ to five even with the fifth ligand being the large chromium synthon. On the other hand the solvation energy contribution is not more important than to allow dissociation of water and preservation of the coordination number in *trans*-$[Cr(py)_4F(\mu\text{-}F)Li(H_2O)_3]^{2+}$. The absence of double coordination of the Li(I) cation by the chromium fluoride complex is not surprising considering the size of Li^+ and the charge density build-up associated with systems of higher nuclearity. The observed difference in reactivity for Li(I) and Na(I) cannot be explained solely based on parameters such as size and hydration energy also the energy of the bond between the alkali metal ions and fluorido ligands must be included as an important contribution, but quantification of the energetics of this interaction remains a task for the future. In an attempt to generalize these reactions and considerations, other simple alkalimetal salts have been investigated for potential reactivity with the *trans*-$[Cr(py)_4F_2]^+$ synthon. For several cases, reactivity leading to new, but incompletely characterized products was observed. This was however, not invariably the case and the natural attempt to extend the series by reaction with $KHCO_3$, failed employing similar conditions as for the synthesis of *trans-catena*-poly$[Na(H_2O)_4(\mu\text{-}F)Cr(py)_4(\mu\text{-}F)](HCO_3)_2$.

Synthons with three fluorido ligands, *mer*/*fac*-[Cr(L‴)F$_3$]

As mentioned in the introduction of this chapter the synthon based on kinetically robust Cr(III) can in principle hold everything from 1 to 6 fluorido ligands combined with an variable auxiliary ligand sphere. Change in the number of fluorido ligands will lead to changes in both both the overall charge and ability for the synthon to engage unsupported fluorido bridging. In addition to the complexes described in this chapter, which all are based on difluorido synthons the results were extended to encompass investigation of robust, neutral trifluorido complexes of

Cr(III) by other members of the research group. The systems employde were [Cr(L‴)F_3] for L‴=terpy, Me_3tacn in which the geometric configuration of the ligands is constrained by the auxiliary ligand to be meridional and facial, respectively. It could be shown that these neutral complexes react with Na(I) in weak donor solvents forming complicated cationic clusters. These investigations are discussed in detail in Paper 4.

STRUCTURAL DESCRIPTION

The molecular structure of the two new alkali metal Cr(III) fluorido complexes has been determined by single crystal X-ray diffraction. These are:

- *trans-catena*-poly[Na(H_2O)$_4$(μ-F)Cr(py)$_4$(μ-F)](HCO_3)$_2$
- *trans*-[Cr(py)$_4$F(μ-F){Li(H_2O)$_{n=3,4}$}$_{m=0,1}$]Cl_5·6H_2O

Detailed information about the single crystal diffraction experiment, data collection and refinement data are given in Appendix 3, "X-ray diffraction". See Table 37 and Table 38 respectively. The singlesingle crystal structures of both compounds have been published in Paper 4. In the following sections are the two structures described individually followed by a comparison of the general properties of these systems. Of the complexes known in literature, it is difficult with conviction to perform a comparison with the two described alkali metal complexes despite the relative simplicity are they to deviant. Unsupported bridging, M–(μ-F)–A are only known in very few cases in literature.

Single crystal structure of *trans-catena*-poly[Na(H_2O)$_4$(μ-F)Cr(py)$_4$(μ-F)](HCO_3)$_2$

Structure of the formula unit

The molecular structure of a dinuclear fragment of the chains has been depicted in Figure 32, while selected geometric parameters are given in Table 10.

The compound crystallizes in the tetragonal crystal system in the space group *P*4/*ncc*. The structure consists of octahedrally coordinated Cr(III) and Na(I) ions connected by fluorido bridges to form an infinite linear chain with a symmetry dictated bridging angle $\angle Cr_1–F_1–Na_1$ of 180 ° (refined 180.00(4) °). The coordination polyhedron around the Cr(III) central ion is besides the two bridging fluorido ligands formed by four equiavalent equatorial nitrogen ligators positioned with angles of 89.78(3) ° and 90.22(3) ° with respect to the two axial ligands and forming a 90.00 ° angle relative to each other. The pyridine rings from which the nitrogen ligators originate are tilted with 20.62 ° corresponding to the angle between the least square planes formed by the pyridine ring systems with symmetry label x,y,z and ½–x,½–y,z or equivalent x,½–y,z and ½x,y,z (spacegroup symmetry).

The Cr_1–N_1 bond distance with the value 2.0835(6) Å is directly comparable with the average Cr–N value of 2.090(2) Å found in the precursor derivative of *trans*-$[Cr(py)_4F_2]PF_6$ (space group *C*2/*c*). [326]

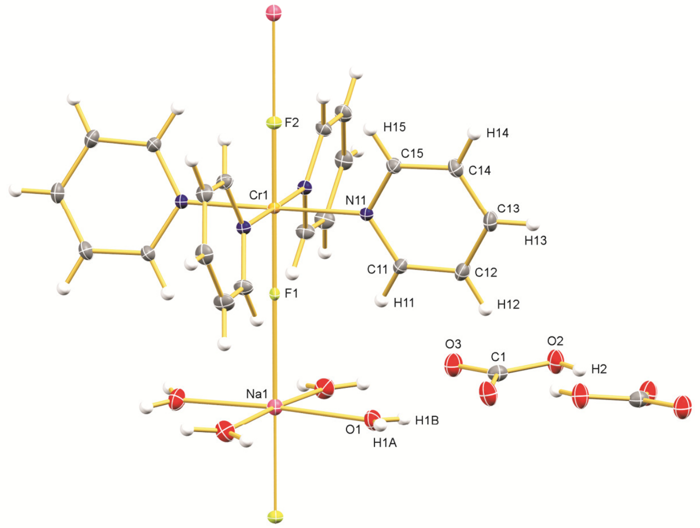

Figure 32 Molecular structure of *trans-catena*-poly$[Na(H_2O)_4(\mu\text{-}F)Cr(py)_4(\mu\text{-}F)](HCO_3)_2$

A similar comparison can be made of the two Cr–F bond distances with the values 1.8750(8) and 1.8635(8) Å, almost identical with a difference of only 0.0115 Å. The bond distances are also close to the corresponding distance of 1.853(2) Å found in *trans*-$[Cr(py)_4F_2]PF_6$. [326] This relationship is remarkable since the fluorido ligands in *trans-catena*-poly$[Na(H_2O)_4(\mu\text{-}F)Cr(py)_4(\mu\text{-}F)](HCO_3)_2$ and *trans*-$[Cr(py)_4F_2]PF_6$ would be considered to be quite different in terms of bonding. The octahedral coordination polyhedra around the Na(I) ions are formed by two axial fluorido ligands in angle linearly, *trans* disposed forming an angle of 180 ° and four equatorial water ligands positioned at an angle of 91.68(3) ° with the fluorido ligands. The bonding distance between the oxygen ligators in the water ligands and Na(I) ions is 2.3517(6) Å while the distances 2.490(1) and 2.407(1) Å to the two fluorido ligands are somewhat longer. The four water ligands including their hydrogen atoms are confined strictly to the equatorial plane around sodium(planar coordinating water ligands) with a angle of 89.95(2) ° from each other. The resulting pattern is one of a regular planar coordination with all hydrogen bonding possibilities (that is the O–H vectors of the water ligands) confined to be perpendicular to the Na–F vectors. A relatively large differences of 0.083 Å between the two Na–F distances, 2.490(1) vs. 2.407(1) Å is noted. This difference is significantly more pronounced than the one

observed for Cr–F distances. Given these differences in bond lengths the infinite chain can be understood as being built of relatively strong $[Na(H_2O)_4(\mu\text{-}F)Cr(py)_4(F)]^{2+}$ units.

Bond length (Å)			Bond angle (°)		
Cr_1-F_1	1.8750(8)	F_1-Cr_1-F_2	180.00(3)	F_2-Na_1-O_1	88.32(3)
Cr_1-F_2	1.8635(8)	F_1-Cr_1-N_1	90.22(3)	O_1-Na_1-O_1	176.64(3)
Cr_1-N_1	2.0835(6)	F_2-Cr_1-N_1	89.78(3)	O_1-Na_1-O_1	89.95(2)
Na_1-O_1	2.3517(6)	N_1-Cr_1-N_1	179.56(2)	Na_1-O_1-H_{1A}	126.87(5)
Na_1-F_1	2.490(1)	N_1-Cr_1-N_1	90.00(2)	Na_1-O_1-H_{1B}	119.02(5)
Na_1-F_2	2.407(1)	F_1-Na_1-F_2	180.00(3)	Cr_1-F_1-Na_1	180.00(4)
O_1-H_{1A}	0.8386(5)	F_1-Na_1-O_1	91.68(3)	Cr_1-F_2-Na_1	180.00(4)
O_1-H_{1B}	0.7930(5)				
O_2-H_2	0.8404(5)				

Table 10 Selected geometric parameters for *trans-catena*-poly$[Na(H_2O)_4(\mu\text{-}F)Cr(py)_4(\mu\text{-}F)](HCO_3)_2$

These units are by coordination linked in infinite chain through somewhat longer and probably weaker fluorido bridges. Both Na–F distances are significantly longer than the NaF gas-phase equilibrium bond length of 1.9260 Å found by microwave spectroscopy, [23] and somewhat longer than the distance of 2.3170 Å, seen in the rare NaF mineral Villiaumite. [52] This structure is one of the classical ones determined originally by Bragg in 1920. In his classic determination Na–F distance was found to be 2.39 Å. [327]
The fluorido bridge bonding motif between chromium and Na(I), Cr–F–$Na(OH_2)_n$ has only been reported for one other example in the literature; the mixed Cr(III/II) complex of bis(2-pyridyl)amine (dpa), $[Cr_3(dpa)_4F_2Na(H_2O)BF_4]BF_4{\cdot}CH_3OH$. [328] This complex consists of a linear trinuclear chromium core, $[Cr_3(dpa)_4]^{3+}$ with mixed oxidation state in sequence (III/II/II), accessible in endpoint to coordination of *e.g.* fluoride. The fluorido ligand attached to the Cr(II) end forms a bridge to a Na(I) cation with the additional coordination sphere yielding the entity $[Na(H_2O)(\mu\text{-}FBF_3)(\mu_2\text{-}F_2BF_2)]^-$. The presence of mixed oxidation states of chromium and simultaneous Cr(II)–Cr(II) quadruple bonding internally in the $[Cr_3(dpa)_4]^{3+}$ core, complicates a direct comparison with *trans-catena*-poly$[Na(H_2O)_4(\mu\text{-}F)Cr(py)_4(\mu\text{-}F)](HCO_3)_2$ despite the two structures contain the same structural elements. As expected, deviations are seen in respect to both bond lengths and angles between the two systems. This is seen in case of the Cr(II)–μ_2F distances, 1.979(2) Å in the dpa-complex which is much longer than the average 1.81693(8) Å found in *trans-catena*-poly$[Na(H_2O)_4(\mu\text{-}F)Cr(py)_4(\mu\text{-}F)](HCO_3)_2$. However, at the other terminal end of the $[Cr_3(dpa)_4]^{3+}$ core were a Cr(III) coordinate a fluorido ligand, a shorter, Cr(III)-like bond length of 1.843(2) Å is found. This demonstrates that the Cr–Cr quadrupel bonding in the $[Cr_3(dpa)_4]^{3+}$ core weakens the bonding strength to the axial ligands. On the other

hand the bonds between sodium and its ligators are correspondingly shorter with Na–F: 2.175(3) Å and Na–OH_2: 2.248(5) Å. The Na–F distance in this compound is even shorter than the the Na–F distance in the previously mentioned Villiaumite mineral. When comparing the complete packing around the central ions in two systems this leads to a description of the two systems as sterically quite uncrowded in *trans*-*catena*-poly[Na(H_2O)$_4$(μ-F)Cr(py)$_4$(μ-F)](HCO_3)$_2$ and sterically quite crowded in [Cr_3(dpa)$_4$$F_2$Na($H_2O$)$BF_4$]$BF_4$·$CH_3OH$. When comparing the ∠Cr–F–Na bond angles of 169.90(13) ° in the latter to the 180 ° found in *trans*-*catena*-poly[Na(H_2O)$_4$(μ-F)Cr(py)$_4$(μ-F)](HCO_3)$_2$ the difference could be an indication of sterically imposed conditions in the former and that the ideal bond angle for fluoride bridging between chromium and sodium is close to linear. This may appear a contrieved generalization based on comparison of only two structures for which many other factors could play a role, but it is in agreement with the general behavior of fluoride bridges.

Packing of unit cell

The packing of the unit cell of *trans*-*catena*-poly[Na(H_2O)$_4$(μ-F)Cr(py)$_4$(μ-F)](HCO_3)$_2$ is depicted in Figure 33. Two main characteristics are observed. First the parallel, side-by-side arrangement of the 1D dimensional coordination polymers and secondly, and most prominent, the division of the infinite chain into distinct layers containing *trans*-[Cr(py)$_4$$F_2$]$^+$ and *trans*-[Na(H_2O)$_4$]$^+$/ HCO_3^- respectively. The latter pair of counter ions and coordinated water ligands forms a extensive 2D hydrogen bonding network perpendicular to the axis of the polymer chain.

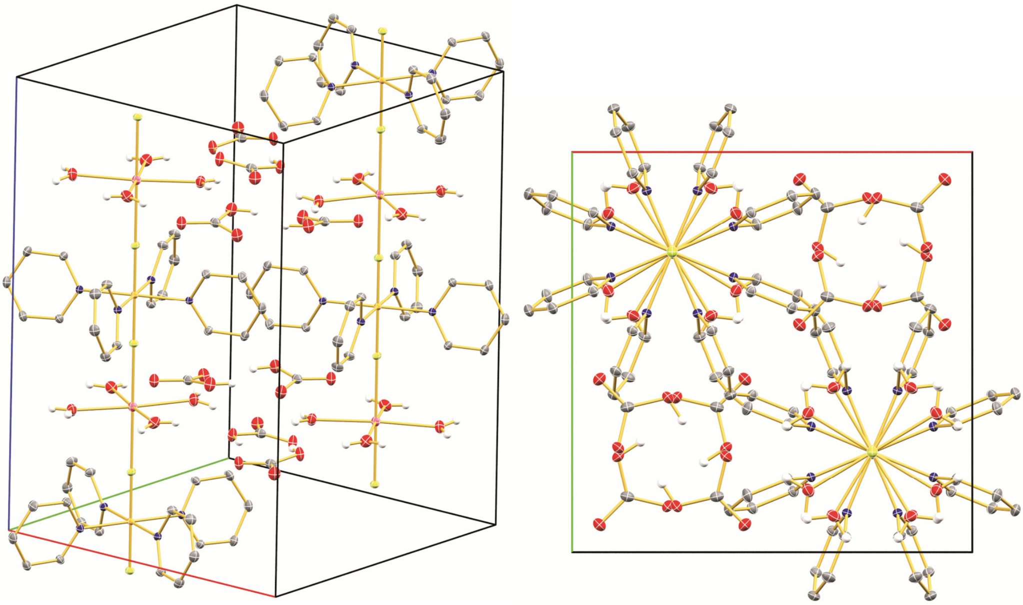

I: Oblique view of unit cell. **II:** View along unit cell *b*-axis

Figure 33 Unit cell of *trans-catena*-poly[Na(H_2O)$_4$(μ-F)Cr(py)$_4$(μ-F)](HCO_3)$_2$

Hydrogen atoms other than them originating from water and HCO_3^- have been omitted for clarity.

Hydrogen bonding

The hydrogen bond network is depicted in Figure 34 while values of bonding lengths and angles are given in Table 11. From these data, characterizing values for the hydrogen bond network fall in the intervals: D···A length: 2.6-2.9 Å and ∠D–H···A angle: 160-176 ° and it is concluded, that the strengths of all of the hydrogen bonds must be classified as moderately strong. The network is formed by hydrogen bonding of all water ligands in the *trans*-[Na(H_2O)$_4$]$^+$ entity to four HCO_3^- ions, in such a way that a single water ligand form hydrogen bonds to two different HCO_3^- ions. This bonding pattern is not symmetrical as evidenced by the bond lengths 2.8304(5) and 2.9142(8) Å although the chain in itself shows strict 4-fold symmetry. A characteristic feature of the network is the internal hydrogen bonds between two HCO_3^- ions thereby forming a symmetrical pair [$O_2C(OH^{\cdots}O)_2CO_2$]$^{2-}$ with a D···A distance, 2.6207(8) Å but somewhat bent hydrogen bonds.

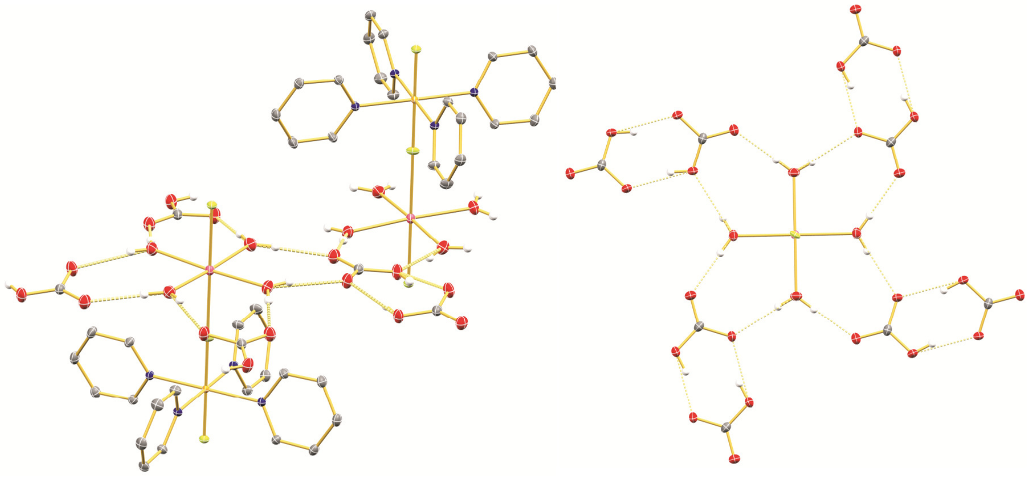

I: Oblique view. **II:** View along unit cell *b*-axis.

Figure 34 Hydrogen bonding in *trans-catena*-poly[Na(H_2O)$_4$(μ-F)Cr(py)$_4$(μ-F)](HCO_3)$_2$

Hydrogen atoms other than those originating from water and HCO_3^- have been omitted for clarity.

D–H···A (Å)	D–H (Å)	H···A (Å)	D···A (Å)	∠D–H···A (°)
O_1–H_{1B}···O_3	0.8386(5)	1.9932(2)	2.8304(5)	176.06(3)
O_1^{i}–H_{1A}···O_2	0.7930(5)	2.1358(5)	2.9142(8)	167.14(4)
O_2–H_2···O_2^{ii}	1.8126(5)	0.8404(5)	2.6207(8)	160.78(4)

Symmetry codes: (i)1/2–x, y, z; (ii) 1–x, 1–y, 1/2–z.

Table 11 Hydrogen bond geometry for *trans-catena*-poly[Na(H_2O)$_4$(μ-F)Cr(py)$_4$(μ-F)](HCO_3)$_2$

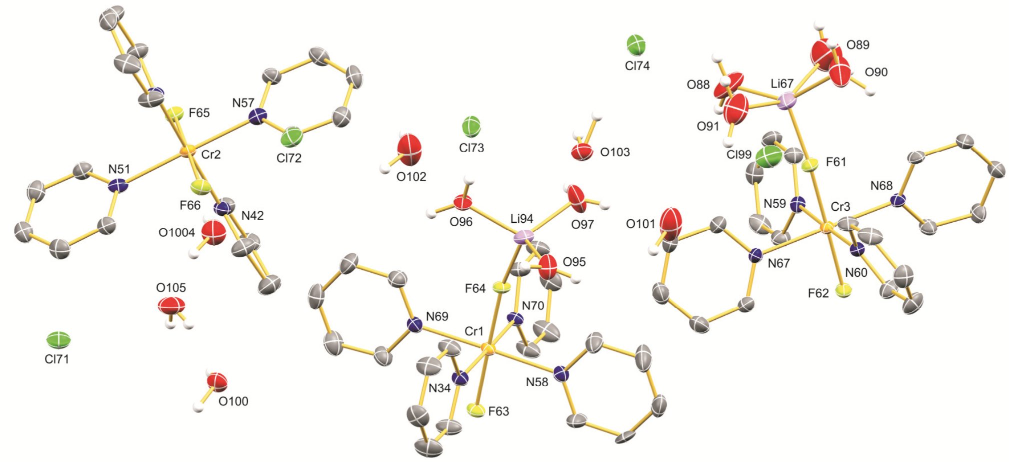

Figure 35 Molecular structure of *trans*-$[Cr(py)_4F(\mu\text{-}F)\{Li(H_2O)_{n=3,4}\}_{m=0,1}]Cl_5{\cdot}6H_2O$

Hydrogen atoms are assigned labels based on the oxygen atom they are bound *e.g.* the two hydrogen atoms bonded to O_{105} is named as H_{105A} and H_{105B}. Hydrogen atoms other than those originating from water are omitted for clarity.

Structural description of *trans*-$[Cr(py)_4F(\mu\text{-}F)\{Li(H_2O)_{n=3,4}\}_{m=0,1}]Cl_5{\cdot}6H_2O$

Molecular structure

The molecular structure of *trans*-$[Cr(py)_4F(\mu\text{-}F)\{Li(H_2O)_{n=3,4}\}_{m=0,1}]Cl_5{\cdot}6H_2O$ is shown in Figure 35 while a representative collection of geometric parameters for the complex are given in Table 12. The asymmetric unit consists of three cations are all derived from *trans*-$[Cr(py)_4F_2]^+$. Two of theses cations are coordinated through a fluorido bridge to a $[Li(H_2O)_3]^+$ and $[Li(H_2O)_4]^+$ respectively forming *trans*-$[Cr(py)_4F(\mu\text{-}F)Li(H_2O)_n]^{2+}$ were n=3,4. The coordination polyhedra around the three Cr(III) central ions are approximately octahedral and consists of four equatorial nitrogen ligators and two axial fluorido ligands where the average ∠N–Cr–N angles for the three cations are 89.98(1) °, 90.0(1) ° and 89.98(1) °. The coordination polyhedron around the four-coordinated Li(I) ions in *trans*-$[Cr(py)_4F(\mu\text{-}F)Li(H_2O)_3]^{2+}$ forms an slightly distorted tetrahedron with average ∠F–Li–O and ∠O–Li–O angles of 108.6(3) ° and 110.2(3) ° and with the Li(I) central ion placed 0.608 Å above the least square plane spanned by the three oxygen ligators. For the 5 coordinated Li(I) ion in *trans*-$[Cr(py)_4F(\mu\text{-}F)Li(H_2O)_4]^{2+}$, the coordination polyhedron is a square pyramid with the fluoride ligand at the apex and the oxygen ligators spanningthe basal plane with a average ∠O–Li–O angle of 86.03(3) °. The central Li(I) ion is placed in a distance of 0.531 Å over this square which contributes to a distortion of the ∠F–Li–O from the regular giving a average of 104.93(3) °. The bond distance between Li(I) and the O ligators

vary considerably between the two cations. Thus, the average Li–O distance in *trans*-$[Cr(py)_4F(\mu\text{-}F)Li(H_2O)_3]^{2+}$ is 1.910(6) Å whereas in *trans*-$[Cr(py)_4F(\mu\text{-}F)Li(H_2O)_4]^{2+}$ it is found to be 2.039(8) Å. The two Cr–F bond lengths in the isolated *trans*-$[Cr(py)_4F_2]^+$ ion are 1.851(1) and 1.874(2) Å with an average of 1.863(2) Å and a difference of 0.023 Å. This is slightly larger than the 1.853(2) Å determined in *trans*-$[Cr(py)_4F_2]PF_6$ (space group *C*2/*c*), [326] but close to the corresponding values 1.8750(8) and 1.8635(8) Å observed for *trans*-*catena*-poly$[Na(H_2O)_4(\mu\text{-}F)Cr(py)_4(\mu\text{-}F)](HCO_3)_2$. Coordination of the Li(I) ion through bridge formation with one of the two fluorido ligands has remarkably little influence in terms of perturbation of the Cr–F bond lengths. Similar is the sensitivity towards the number of water ligands coordinated small.

Bond length (Å)		Bond angle (°)					
Cr_1-F_{63}	1.854(2)	N_{58}-Cr_1-N_{34}	91.9(1)	O_{95}-Li_{94}-O_{96}	107.7(3)	F_{61}-Cr_3-N_{67}	90.09(8)
Cr_1-F_{64}	1.877(2)	N_{58}-Cr_1-F_{64}	90.45(9)	N_{42}-Cr_2-N_{39}	178.0(1)	F_{62}-Cr_3-N_{68}	90.06(9)
Cr_1-N_{34}	2.074(3)	N_{58}-Cr_1-F_{63}	88.80(9)	N_{42}-Cr_2-N_{57}	88.5(1)	F_{62}-Cr_3-N_{67}	89.33(8)
Cr_1-N_{58}	2.084(2)	N_{58}-Cr_1-N_{69}	179.0(1)	N_{42}-Cr_2-N_{51}	91.0(1)	N_{68}-Cr_3-N_{67}	179.4(1)
Cr_1-N_{69}	2.094(2)	N_{58}-Cr_1-N_{70}	89.1(1)	N_{42}-Cr_2-F_{65}	91.05(9)	Cr_3-F_{61}-Li_{87}	171.2(2)
Cr_1-N_{70}	2.083(2)	N_{34}-Cr_1-F_{64}	90.61(9)	N_{42}-Cr_2-F_{66}	89.20(9)	H_{91A}-O_{91}-Li_{87}	109.6(3)
Cr_2-F_{65}	1.874(2)	N_{34}-Cr_1-F_{63}	89.29(9)	N_{39}-Cr_2-N_{57}	90.6(1)	H_{91B}-O_{91}-Li_{87}	120.6(3)
Cr_2-F_{66}	1.851(1)	N_{34}-Cr_1-N_{69}	87.8(1)	N_{39}-Cr_2-N_{51}	89.9(1)	H_{90A}-O_{90}-Li_{87}	109.6(3)
Cr_2-N_{39}	2.088(3)	N_{34}-Cr_1-N_{70}	178.9(1)	N_{39}-Cr_2-F_{65}	90.73(9)	H_{90B}-O_{90}-Li_{87}	119.8(3)
Cr_2-N_{42}	2.094(3)	F_{64}-Cr_1-F_{63}	179.24(8)	N_{39}-Cr_2-F_{66}	89.02(9)	H_{88A}-O_{88}-Li_{87}	109.5(3)
Cr_2-N_{51}	2.074(2)	F_{64}-Cr_1-N_{69}	90.51(9)	N_{57}-Cr_2-N_{51}	179.4(1)	H_{88B}-O_{88}-Li_{87}	120.0(3)
Cr_2-N_{57}	2.070(2)	F_{64}-Cr_1-N_{70}	89.79(9)	N_{57}-Cr_2-F_{65}	90.33(9)	H_{89A}-O_{89}-Li_{87}	109.5(5)
Cr_3-F_{61}	1.867(2)	F_{63}-Cr_1-N_{69}	90.23(9)	N_{57}-Cr_2-F_{66}	89.97(9)	H_{89B}-O_{89}-Li_{87}	120.5(4)
Cr_3-F_{62}	1.862(2)	F_{63}-Cr_1-N_{70}	90.32(9)	N_{51}-Cr_2-F_{65}	89.93(9)	F_{61}-Li_{87}-O_{91}	108.3(3)
Cr_3-N_{59}	2.080(3)	N_{69}-Cr_1-N_{70}	91.1(1)	N_{51}-Cr_2-F_{66}	89.76(9)	F_{61}-Li_{87}-O_{90}	105.7(3)
Cr_3-N_{60}	2.084(2)	Cr_1-F_{64}-Li_{94}	164.2(2)	F_{65}-Cr_2-F_{66}	179.61(8)	F_{61}-Li_{87}-O_{88}	105.8(3)
Cr_3-N_{67}	2.092(2)	H_{97A}-O_{97}-Li_{94}	109.7(2)	N_{59}-Cr_3-N_{60}	179.0(1)	F_{61}-Li_{87}-O_{89}	99.9(3)
Cr_3-N_{68}	2.079(2)	H_{97B}-O_{97}-Li_{94}	120.1(2)	N_{59}-Cr_3-F_{61}	89.84(9)	O_{91}-Li_{87}-O_{90}	96.6(3)
Li_{87}-F_{61}	1.874(7)	H_{95A}-O_{95}-Li_{94}	109.6(3)	N_{59}-Cr_3-F_{62}	90.24(9)	O_{91}-Li_{87}-O_{88}	88.7(3)
Li_{94}-F_{64}	1.864(6)	H_{95B}-O_{95}-Li_{94}	113.0(3)	N_{59}-Cr_3-N_{68}	90.9(1)	O_{91}-Li_{87}-O_{89}	151.6(4)
Li_{87}-O_{88}	2.023(7)	H_{96A}-O_{96}-Li_{94}	109.5(3)	N_{59}-Cr_3-N_{67}	88.9(1)	O_{90}-Li_{87}-O_{88}	144.5(4)
Li_{87}-O_{89}	2.171(9)	H_{96B}-O_{96}-Li_{94}	114.6(3)	N_{60}-Cr_3-F_{61}	89.68(9)	O_{90}-Li_{87}-O_{89}	78.3(3)
Li_{87}-O_{90}	1.931(7)	F_{64}-Li_{94}-O_{97}	103.1(3)	N_{60}-Cr_3-F_{62}	90.25(9)	O_{88}-Li_{87}-O_{89}	80.5(3)
Li_{87}-O_{91}	2.029(7)	F_{64}-Li_{94}-O_{95}	110.6(3)	N_{60}-Cr_3-N_{68}	88.1(1)		
Li_{94}-O_{95}	1.939(6)	F_{64}-Li_{94}-O_{96}	112.1(3)	N_{60}-Cr_3-N_{67}	92.0(1)		
Li_{94}-O_{96}	1.885(6)	O_{97}-Li_{94}-O_{95}	105.8(3)	F_{61}-Cr_3-F_{62}	179.41(7)		
Li_{94}-O_{97}	1.907(6)	O_{97}-Li_{94}-O_{96}	117.2(3)	F_{61}-Cr_3-N_{68}	90.52(9)		

Table 12 Selected geometric parameters for *trans*-$[Cr(py)_4F(\mu\text{-}F)\{Li(H_2O)_{n=3,4}\}_{m=0,1}]Cl_5{\cdot}6H_2O$

In *trans*-[Cr(py)$_4$F(μ-F)Li(H$_2$O)$_3$]$^{2+}$ the lengths of the bridged and un-bridged Cr–F bond is 1.877(2) Å and 1.854(2) Å respectively while the Li–F is 1.864(6) Å. Similar found in *trans*-[Cr(py)$_4$F(μ-F)Li(H$_2$O)$_4$]$^{2+}$ with 1.867(2) and 1.862(2) Å respectively and Li–F 1.874(7) Å. Comparing the previously mentioned Li–O distances (1.910(6) and 2.039(8) Å) with the Li–F distances (1.864(6) and 1.874(7) Å) it is seen that the distance between the Li(I) ion and the fluorido ligand is in both cases the shortest. This indicate that Li–F interaction is significant. The fluorido bridges ∠Li–F–Cr are not completely linear with 164.2(2) ° and 171.2(2) ° in the two ions *trans*-[Cr(py)$_4$F(μ-F)Li(H$_2$O)$_3$]$^{2+}$, *trans*-[Cr(py)$_4$F(μ-F)Li(H$_2$O)$_4$]$^{2+}$ respectively. This sets the lithium systems apart from the sodium case discussed above, but might be related to the engagement of the alkali metal bound water in hydrogen bonding.

Hydrogen bonding

See Table 13 and Figure 36, 37 for the hydrogen bond geometry of *trans*-[Cr(py)$_4$F(μ-F){Li(H$_2$O)$_{n=3,4}$}$_{m=0,1}$] Cl$_5$·6H$_2$O

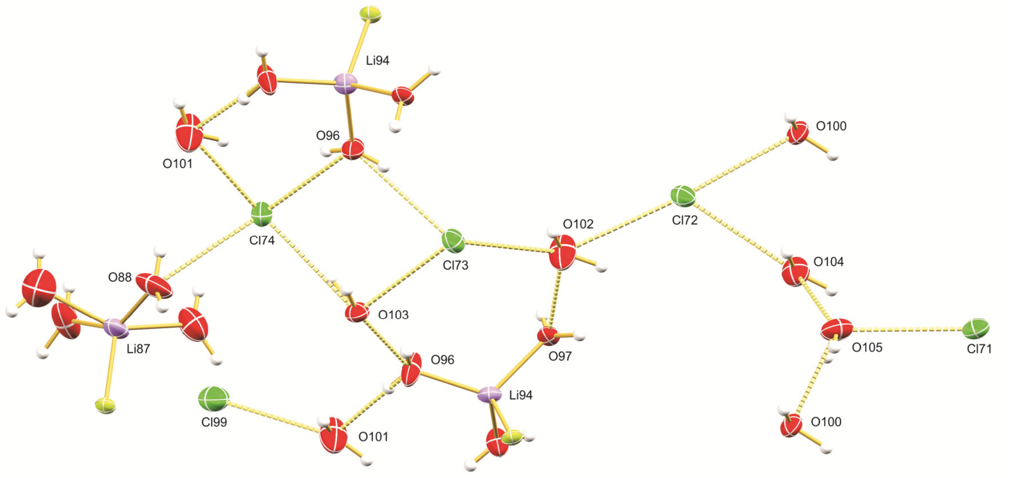

Figure 36 Hydrogen bonding and packing in *trans*-[Cr(py)$_4$F(μ-F) {Li(H$_2$O)$_{n=3,4}$}$_{m=0,1}$] Cl$_5$·6H$_2$O

It is noteworthy, that for both the Li(I) and the Na(I) derived systems, the water molecules coordinating the alkali metal engage to some extent in hydrogen bonding. In the Na(I) compound, this hydrogen bonding is exhaustive and involves all of the water bound hydrogen, while both fluoride-bridged cations of the Li(I) compound has only one coordinated water engaging in hydrogen bonding. The inferiority of the chloride counter ions for hydrogen bonding in *trans*-[Cr(py)$_4$F(μ-F){Li(H$_2$O)$_{n=3,4}$}$_{m=0,1}$]Cl$_5$·6H$_2$O results in formation of hydrogen bonded chains

wherein, the second fluoride of the Cr(III) precursor is used for hydrogen bonding rather than alkali metal coordination. The asymmetry of the hydrogen bonded chains with respect to the F–Cr–F axis for both $[Cr(py)_4F(\mu\text{-}F)Li(H_2O)_3]^{2+}$ and $[Cr(py)_4F(\mu\text{-}F)Li(H_2O)_4]^{2+}$ chains, may influence the ∠Li–F–Cr bond angle and cause the observed bending in these systems.

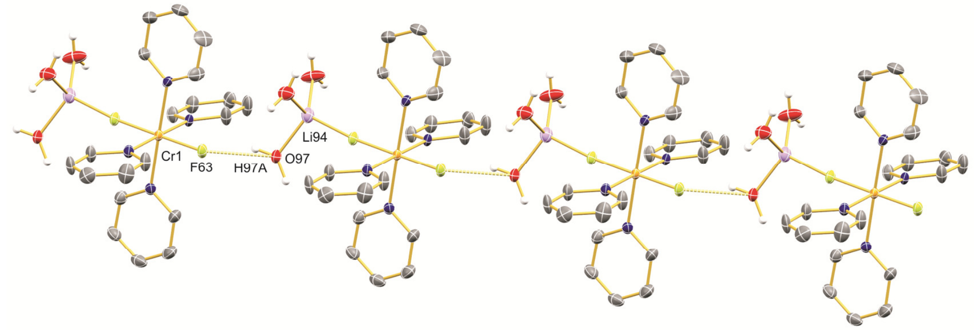

I: Hydrogen bonding in $[Cr(py)_4F(\mu\text{-}F)Li(H_2O)_3]^{2+}$

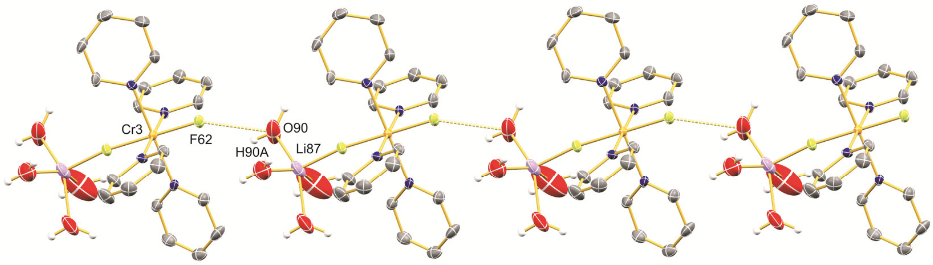

II: Hydrogen bonding in $[Cr(py)_4F(\mu\text{-}F)Li(H_2O)_4]^{2+}$

Figure 37 Hydrogen bonding in dimeric units of ***trans*****-$[Cr(py)_4F(\mu\text{-}F)\{Li(H_2O)_{n=3,4}\}_{m=0,1}]Cl_5{\cdot}6H_2O$**

D–H···A	D–H (Å)	H···A (Å)	D···A (Å)	∠D–H···A (°)
O_{97}-H_{97A}···F_{63}	0.820(2)	1.914(2)	2.683(3)	155.9(2)
O_{90}-H_{90A}···F_{62}	0.819(4)	2.143(2)	2.745(4)	130.2(2)
O_{105}-H_{105A}···O_{100}	0.820(3)	2.184(3)	2.839(4)	136.9(2)
O_{96}-H_{96B}···O_{101}	0.827(2)	1.906(3)	2.727(4)	
O_{95}-H_{95A}···Cl_{73}	0.818(2)	2.3350(9)	3.115(3)	
O_{103}-H_{103A}···Cl_{73}	0.820(2)	2.8333(9)	3.121(3)	
O_{103}-H_{103A}···Cl_{73}	0.820(2)	2.8333(9)	3.121(3)	
O_{101}-H_{101B}···Cl_{74}	0.961(3)	2.4095(9)	3.206(3)	
O_{95}-H_{95B}···Cl_{74}	0.810(2)	2.4871(9)	3.200(2)	
O_{103}-H_{103B}···Cl_{74}	1.043(3)	2.163(1)	3.187(3)	

Table 13 Hydrogen bond geometry for ***trans*****-$[Cr(py)_4F(\mu\text{-}F)\{Li(H_2O)_{n=3,4}\}_{m=0,1}]Cl_5{\cdot}6H_2O$**

EXPERIMENTAL DETAILS

This section provides specifications for the synthetic preparation as well as the characterization of the new fluorido bridged alkali metal complexes of Cr(III) discussed in this chapter. A graphical overview of the complexes and reactivity discussed in the following is given in Scheme 3.

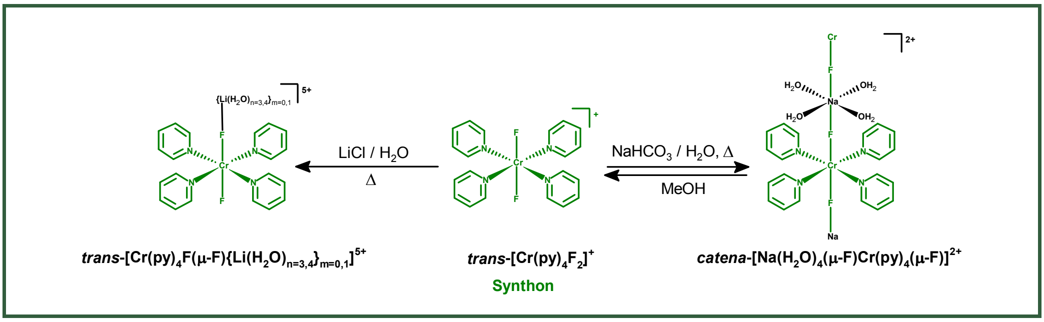

Scheme 3

- *trans-catena*-poly[$Na(H_2O)_4(\mu\text{-}F)Cr(py)_4(\mu\text{-}F)](HCO_3)_2$ (Synthesis and reactivity)
- *trans*-{[$Cr(py)_4F_2$][$Cr(py)_4(\mu\text{-}F)Li(H_2O)_{n=3,4}$]}$Cl_5{\cdot}6H_2O$ (Synthesis)

General comments

The following preparations of the complexes are all performed under normal laboratory conditions and the use of all chemicals and solvents direct from the supplier without prior purification or drying. The supplier of a given chemical is spcificeret where this first appeared in the preparation. Also indicated are the purity by use of the declaration used by the specefic supplier. The starting material *trans*-[$Cr(py)_4F_2$]NO_3 was prepared acording to the method given by Glerup *et al.* [6]

All the synthesized compounds has been analyzed by a variety of techniques (*e.g.* elementary elementary anaylsis, IR, MS, EPR,…). A technical description of the techniques, equipment and detailed use in characterization is given in Appendix 2, “Instrumentarium”.

Synthesis of *trans-catena*-poly[Na(H_2O)$_4$(μ-F)Cr(py)$_4$(μ-F)](HCO_3)$_2$

$$trans\text{-}[Cr(py)_4F_2]NO_3 + 2\ NaHCO_3 \xrightarrow[\Delta]{H_2O} trans\text{-}catena\text{-}poly[Na(OH_2)(\mu\text{-}F)Cr(py)_4(\mu\text{-}F)](HCO_3)_2 + NaNO_3$$

trans-[Cr(py)$_4$F$_2$]NO$_3$ (10.031g, 21.4 mmol) was dissolved in water (200 mL). The resulting violet solution was filtered through a wad of glass wool and heated to 30 °C with stirring before solid sodium hydrogenecarbonate (10.015 g, 119.2 mmol, anhydrous) was added in one batch. Dissolution of the sodium hydrogencarbonate gave a cloudy solution of a more pink color. Within a few minutes a pink solid precipitates, but dissolves shorthly after with the continued heating. At a temperature of 50 °C the beaker is transferred directly to an ice/water bath and left undisturbed for ca. 1 hour. An intensely pink, crystaline product was isolated by vacuum filtration on a sintered glass funnel (No. 3) and dried as much as possible by suction before the product is placed in air.

Yield: 10.886 g (81.5 % of theoretical based on chromium)

- Elemental analysis: Calcd. (found) (%) for $H_{30}C_{22}N_4O_{10}F_2Na_1Cr_1$: H 4.58(4.85), C 42.39(42.38), N 8.90(8.99), Na 3.69(3.26), Cr: 8.34(8.03).
- FT-IR (cm^{-1}): 3554(w), 3431(b), 3105(w), 3032(w), 2970(w), 2651(b), 2360(w), 1971(w), 1738(m), 1622(s), 1606(s), 1486(w), 1447(s), 1373(s), 1344(s), 1217(s), 1152(w), 1066(s), 1049(w), 1017(w), 990(m), 960(m), 892(w), 830(s), 766(s), 696(s), 649(s), 588(s), 441(s).
- MS (FAB+/3-Nitrobenzyl alcohol) *m/z* (% rel. Intensity): 406.0 [Cr(py)$_4$F$_2$]$^+$, 327.0 [Cr(py)$_3$F$_2$]$^+$, 248.0 [Cr(py)$_2$F$_2$]$^+$, 229.0 [Cr(py)$_2$F]$^{2+}$.
- MS (ESP+/MeOH) *m/z* (% rel. Intensity): 406.1 [Cr(py)$_4$F$_2$]$^+$, 345.1 [?] (also seen in Li-complex), 327.1 [Cr(py)$_3$F$_2$]$^+$, 248.0 [Cr(py)$_2$F$_2$]$^+$.
- MS (EI+) *m/z* (% rel. Intensity): 501.673 [Cr(py)$_4$F$_2$Na(H$_2$O)$_4$]$^{2+}$, 405.934 [Cr(py)$_4$F$_2$]$^+$.

Crystals for single crystal diffraction

Crystals suitable for single crystal diffraction were obtained by the above mentioned procedure with the modification that the synthesis was performed on a scale of 1:50 and that additional water was added to reaction mixure, so that the initially precipitated product could be dissolved completely by heating to ca. 30 °C. After filtering, the reaction mixture was left for crystallization at room temperature giving crystals after 2-3 h.

Reactivity and use of *trans-catena*-poly[$Na(H_2O)_4(\mu$-F)Cr(py)$_4(\mu$-F)]($HCO_3)_2$

This section consists of two connected parts:

- Degradation of *trans-catena*-poly[$Na(H_2O)_4(\mu$-F)Cr(py)$_4(\mu$-F)]($HCO_3)_2$
- Synthesis of *trans*-[Cr(py)$_4F_2$]X for X=ClO_4, Br from *trans*-[Cr(py)$_4F_2$]HCO_3

Degradation of *trans-catena*-poly[$Na(H_2O)_4(\mu$-F)Cr(py)$_4(\mu$-F)]($HCO_3)_2$ into constituent

$$\textit{trans-cartena}\text{-poly}[\mathrm{Na(H_2O)_4(\mu\text{-}F)Cr(py)_4(\mu\text{-}F)](HCO_3)_2} \xrightarrow{\mathrm{MeOH}}$$
$$\textit{trans}\text{-}[\mathrm{Cr(py)_4F_2]HCO_3} + \mathrm{NaHCO_3} + 4\,\mathrm{H_2O}$$

trans-catena-poly[$Na(H_2O)_4(\mu$-F)Cr(py)$_4(\mu$-F)]($HCO_3)_2$ (2.000 g, 3.21 mmol) was placed in a small Erlenmeyer flask and treated with methanol (15 mL, Lab Scan). During the next 5-10 min. the mixture was shaken accompanied by precipitation of white solid from the now dark violet solution. The solid was isolated by filtration through a sintered glass funnel (No. 3). The filtrate was saved before washing with methanol and dried in air.

Yield: 0.220 g (81.3 % of theoretical based on chromium)
- Elemental analysis: Calcd. (found) (%) for $H_1C_1O_3Na_1$: H 1.20(1.11), C 14.30(14.24), N 0.0(0.06).
- FT-IR(cm^{-1}): 2970(w), 2524(b), 1923(w,b), 1738(m), 1696(m), 1663(m), 1617(s), 1451(w), 1295(s), 1217(s,b), 1047(m), 1032(m), 994(s), 833(s), 693(s), 657(s), 528(w), 452(w).

The filtrate from the above described experiments contains *trans*-[Cr(py)$_4F_2$]HCO_3 which can be observed by the formation of carbon dioxide by addition of acid. This solution can be used for the preparation of *trans*-[Cr(py)$_4F_2$]X by a simple neutralization of the HCO_3^- with a suitable acid, HX. Generally, *trans*-[Cr(py)$_4F_2$]X with X=ClO_4^-, Br^- and I^- can be prepared by appropriate metathesis from the original NO_3^- salt followed by an appropriate number recrystallizations. [329] However, the neutralization reaction can in some cases be a suitable alternative. Use of the reaction has been tested for the two cases, X=ClO_4^-, Br^-.

Synthesis of *trans*-[Cr(py)$_4$F$_2$]X from *trans*-[Cr(py)$_4$F$_2$]HCO$_3$

$$trans\text{-}[Cr(py)_4F_2]HCO_3 + HX \xrightarrow{MeOH} trans\text{-}[Cr(py)_4F_2]X + CO_2 + H_2O$$

***trans*-[Cr(py)$_4$F$_2$]ClO$_4$**

The violet filtrate (from the previous experiment) was transferred to a small florentine flask and evaporated at 25 °C on a rotary evaporator to the point where the violet solution began to stick to the flask (volume ca. 3 mL). A mixture of $HClO_4$ (2 mL, 60% Riedel-de Haën) and H_2O (2 mL) was added to the evaporated solution until gas evolution stopped. This was accompanied by precipitation of a light violet substance, A solution of $NaClO_4$ (1 M, 7 mL) was added (the product is moderately soluble in this media). The product was isolated by filtration through a sintered glass funnel (No. 3) and washed first with $NaClO_4$ (1 M, 1·5 mL) and then with ice water (4·5 mL) before being dried in a dynamic vacuum.

Yield: 1.009 g (62.2 % of theoretical based on chromium).

- Elemental analysis: Calcd. (found) (%) for $H_{20}C_{20}N_4O_4F_2Cl_1Cr_1$: H 3.99(3.91), C 47.49(47.58), N 11.08(10.89).

***trans*-[Cr(py)$_4$F$_2$]Br·2H$_2$O**

Same procedure as for the ClO_4^- salt but with the following modifications that HBr (0.25 mL, Merck 47%) + ca. 3 drops for completed gas evolution was added directly to the the evaporated solution.

Yield: 1.188 g (70.9 % of theoretical based on chromium).

- Elemental analysis: Calcd. (found) (%) for $H_{24}C_{20}N_4O_2F_2Br_1Cr_1$: H 4.63(4.55), C 45.99(46.07), N 10.73(10.56).

Synthesis of *trans*-[Cr(py)$_4$F(μ-F){Li(H$_2$O)$_{n=3,4}$}$_{m=0,1}$]Cl$_5$·6H$_2$O

$$3\ [Cr(py)_4F_2]NO_3\ +\ 5\ LiCl\ \xrightarrow[\Delta]{H_2O}\ trans\text{-}[Cr(py)_4F_2][trans\text{-}Cr(py)_4F(\mu\text{-}F)Li(H_2O)_{n=3,4}]Cl_5\cdot 6H_2O\ +\ 3\ LiNO_3$$

trans-[Cr(py)$_4$F$_2$]NO$_3$ (10.071 g; 21.5 mmol) was dissolved in water (200 mL). The resulting violet solution was filtered through a wad of glass wool. Then LiCl (21.539 g; 508.0 mmol, Aldrich) was added in batches of approximately 7 g and in such a way that each added batch was completely dissolved before addition of the next. Then the solution was heated up to 50 °C before the conical flask was placed in an ice/water bath. After about 45 min. crystallisation of the pink product begins. Isolation as in case of *trans-catena*-poly[Na(H$_2$O)$_4$(μ-F)Cr(py)$_4$(μ-F)](HCO$_3$)$_2$.

Yield: 6.810 g (57.8 % of theoretical based on chromium)

- Elemental analysis: Calcd. (found) (%) for H$_{28}$Li$_1$C$_{20}$N$_4$O$_4$F$_2$Cr$_1$: H 5.07(5.04), C 43.18(43.48); N 10.07(10.27).
- FT-IR (cm^{-1}): 3735(w), 3339(b), 3106(w), 3029(w), 2970(w), 2362(w), 1970(w), 1738(s), 1635(w), 1606(s), 1485(w), 1446(s), 1365(m), 1216(s), 1155(w), 1065(s), 1047(m), 1015(m), 960(w), 887(w), 775(s), 762(s), 696(s), 647(s), 587(s), 528(s), 441(s).
- MS(ESP+/H$_2$O) *m/z* (% rel. Intensity): 406.2 [Cr(py)$_4$F$_2$]$^+$, 345.1 ? (also seen in Na-complex), 327.1 [Cr(py)$_3$F$_2$]$^+$.

Crystals for single crystal diffraction

Crystals suitable for single crystal diffraction were obtained directly from the preparation described above.

CHAPTER 6

FLUORIDE LABILITY: AQUATION OF *cis*-DI- FLUORIDO Cr(III) COMPLEXES

INTRODUCTION

A key point when targeting fluoride as a bridging ligand between transition metal centres is to obtain control over the process of bridging and limit polymerisation and formation of insoluble fluorides or fluoride containing side products. Therefore, certain requirements are to be met by the synthetic strategy: Most importantly, the used building blocks should be robust with respect to fluoride exchange. Secondly, only a small number of bridges should be available in order to prevent branched non-controllable polymerization. If the reaction is based on preformed, units containing the fluorido ligand coordinated terminally to one of the two metal centres to be connected by a bridge, then controlled bridging could be hoped for.
This approach to construction of chains or finite sized clusters was employed in the synthesis of hetero-metallic compounds of Cr(III) and Na(I)/Li(I), described in Chapter 5, where the metal-containing ligand *trans*-$[Cr(py)_4F_2]^+$, acted as a structure directing motif ensuring chain or linear cluster formation.

If discrete clusters are targeted, building blocks containing one or two fluorido ligands would be expected to be optimal. In addition it must be assumed that a *cis* configuration of two fluorido ligands in an octahedral building block could be desirable. In octahedral Cr(III) complexes, the fluoride ligand in itself imposes no preferences concerning geometric structure and consequently the configuration around the Cr(III) centre must be ensured through requirements of the remaining ligand sphere. In general, though less important for Cr(III), it is desirable that this additional ligand sphere is rigid so that retention of complex configuration is ensured.

Two classical, simple ligand systems which generally form robust complexes, are rigid and meet the requirements for structure directing properties are 1,10-phenantroline and 2,2′-bipyridine. In particular, the sterical properties of these two ligand types enforces *cis* configuration in bidentate complexes. In general terms Josephsen *et al.* states [330]:

> *"We conclude again that all known octahedral, bis(1,10-phenantroline) and bis(2,2'-bipyridine) complexes of chromium(III), cobalt(III), rhodium(III) and iridium(III) have the cis-configuration, and doubt whether trans-complexes are able to exist."*

The validity of this statement from 1969 has over time been further confirmed through numerous structure determinations, [331] in such way that the discussion must be regarded as settled. Thus, despite a small number of counter examples, [332] it can be regarded as a very firm rule that bis complexes of these ligands have *cis* configuration. In agreement with this, systems containing *cis*-$[Cr(L'')_2F_2]^+$ for L"=phen, bpy belong to the group of classic Cr(III) complexes and is well described in literature. [6] In studying the reactivity of these systems and their possible function as metal-based ligands towards the group of Ln(III) ions, it was found that the reaction product is strongly dependent on auxiliary parameters as *e.g.* solvent, counter ion and temperature.
Reacting lanthanoid nitrate, $Ln(NO_3)_3{\cdot}nH_2O$ with *cis*-$[Cr(L'')_2F_2]NO_3{\cdot}nH_2O$ in MeOH at elevated temperature results in formation of the cyclic cluster species such as *cyclo*-$[(NO_3)_4Ln(\mu\text{-}F)Cr(L'')_2(\mu\text{-}F)]$. This reactivity and behavior discussed in Chapter 7. Fairly small changes to the reaction conditions such as replacing the methanol solvent by a water/acetonitrile solvent mixture and replacing the counter ion with a non coordinating ion: ClO_4^- led to a completely different outcome of the reaction. Under these conditions the reaction exclusively proceed as a aquation of the *cis*-$[Cr(L'')_2F_2]^+$ cation in such a way that one and only one of the two fluorido ligands is replaced by water yielding cleanly *cis*-$[Cr(L'')_2(H_2O)(F)]^{2+}$. This quite surprising finding and the structures of two examples of classical mono nuclear Cr(III) complexes with potential as ligands are the subjects of this chapter.

The systems, described in this chapter has formed basis for two minor crystallographic papers. One deals with the single crystal structural characterization of the long known *cis*-$[Cr(phen)_2F_2]ClO_4{\cdot}H_2O$ (see Part II, Publications, Paper 2) and another with structural characterization of the aquated *cis*-$[Cr(bpy)_2(H_2O)(F)](ClO_4)_2{\cdot}2H_2O$ in combination with the lanthanoid synthetic strategy for aquation reactions (see Part II, Publications, Paper 3).

DIFLUORIDO COMPLEXES, $[Cr(L'')_2F_2]^+$

The systems considered in this chapter are all derived from a motif consisting of a central ion Cr(III), surrounded in octahedral fashion by two bidentate ligands, L" with nitrogen as ligator

atom. These species are in the following represented as $[Cr(L'')_2F_2]^+$ with bidentate ligands, L'', encompassing ethane-1,2-diamine (en), 1,10-phenantroline (phen) and 2,2′-bipyridine (bpy). These complexes are similar to classical, robust ammine complexes first studied around 1880-1900 by Jørgensen *e.g.* *trans*-$[Co(NH_3)_4Cl_2]Cl{\cdot}H_2O$, [333] and Werner *e.g.* *cis*-$[Co(NH_3)_4Cl_2]ClO_4$, [334] giving birth to the field of coordination chemistry. The phen and bpy complexes belong naturally in this group based on their synthesis, properties and kinetic behaviour. [335]

The proposed strategies for synthesis of compound belonging to the group of $[Cr(L'')_2F_2]^+$ are all based on treatment of an appropriate Cr(III) contaning material with the specific ligand, L'' in combination with a fluoride source, normaly hydrogen fluoride.
So far as known, the first successful method is given by Fehrmann *et al.* in 1960 and is based on reaction of $CrF_2{\cdot}nH_2O$ and ethane-1,2-diamine in a mixture of diethylether and 100% HF to yield *cis*-$[Cr(en)_2F_2]^+$, which can be isolated as its iodide. [336] Investigations of the *cis*-$[Cr(en)_2F_2]^+$ system was continued by Vaughn *et al.* and published in a series of papers grouped as "Fluorido containing complexes of Cr(III)", which still represent the broadest review of these systems. One of the main areas treated in those publications is how to achieve a reliable synthetic method for producing *cis*-$[Cr(en)_2F_2]^+$. A possible solution to this problem was given with the synthesis of, modestly pure, *cis*-$[Cr(en)_2F_2][Cr(en)F_4]{\cdot}H_2O$ from anhydrous CrF_3 and ethane-1,2-diamine. [337] A similar reaction was achieved with propane-1,2-diamine as co-ligand, but not with propane-1,3-diamine. The reaction product is surprising when compared with the corresponding reaction between the ethane-1,2-diamine and anhydrous $CrCl_3$ or $Cr_2(SO_4)_3$, which both gives $[Cr(en)_3]^{3+}$ in high yield and purity. [338] This difference in behaivior can be seen as a testimony to the strength of the bond between Cr and F. From the double salt, the cation and anion could be isolated separately as $Na[Cr(en)F_4]{\cdot}H_2O$ and *cis*-$[Cr(en)_2F_2]I$, respectively. [337, 339] Later it was shown, that *cis*-$[Cr(en)_2F_2]I$ could be obtained more simply, by letting $[Cr(H_2O)_4Cl_2]Cl$ and ethane-1,2-diamine react in 48% HF. [340]
However, at that point in the development of Cr(III)-fluoride chemistry, many of the synthetic strategies suffered from lack of generality and were not applicable to all diamine ligands. Currently, the most general procedure and the one preferred in the recent literature for synthesis of $[Cr(L'')_2F_2]^+$ with a wide range of bidentate ligands *e.g.* en, pn and chxn, is the one described by Glerup *et al.* in 1970. [329] That method relies on the ligand substitution of pyridine in *trans*-$[Cr(py)_4F_2]^+$ with an appropriate nitrogen containing ligand. [341, 342]

$$trans\text{-}[Cr(py)_4F_2]^+ + 2\,L'' \xrightarrow[\Delta]{MeOEtOH} [Cr(L'')_2F_2]^+ + 4\,py \quad (6.1)$$

The substitution is driven by the use of a high-boiling solvent, 2-methoxyethanol. The equilibrium between the coordinated pyridine and pyridine in the solution/gas phase is shifted towards the free pyridine by the boiling point of 2-methoxyethanol being higher than that of pyridine (115 °C). This method is quite general and extends beyond bidentate auxiliary ligands to synthesis of compounds with polydentate ligands as tris(2-aminoethyl)amine, 1,4,7,10-tetraazatetradecane [6]

and 1,4,8,12-tetraazacyclopentadecane [343]. The *trans*-$[Cr(py)_4F_2]^+$ as starting material has also been used in synthesis of *cis*-$[Cr(NH_3)_4F_2]ClO_4$ and *trans*-$[Cr(NH_3)_4F_2]I{\cdot}H_2O$ by reaction at 100 C with liquid NH_3. [344, 345] The importance of using a high-boiling solvent is demonstrated by Fehrmann, in relation to the first synthesis of a $[Cr(L'')_2F_2]^+$ species, because they had been unsuccessful in preparing the desired cation by reaction of *trans*-$[Cr(py)_4F_2]NO_3$ with ethane-1,2-diamine in water and subsequent addition of HF to the dry solid. [336]

AQUATION REACTIONS IN $[Cr(L'')_2F_2]^+$

An aquation reaction is defined as incorporation of water into a species with or without displacement of one or more other atoms or groups. [346] Based on this general definition the first and second aquation-step of $[Cr(L'')_2F_2]^+$ with respect to the fluorido ligands are given as:

$$\begin{gathered}[Cr(L'')_2F_2]^+ + H_2O \longrightarrow [Cr(L'')_2(H_2O)(F)]^{2+} + F^- \\ [Cr(L'')_2(H_2O)(F)]^{2+} + H_2O \longrightarrow [Cr(L'')_2(H_2O)_2]^{3+} + F^-\end{gathered} \tag{6.2}$$

Reaction kinetic in aquation reaction

Cr(III) complexes, being generally robust due to their d^3 electron configuration, have been extensively studied with respect to ligand exchange kinetics and mechanisms. Also aquation of *cis*-$[M^{III}(L'')_2X_2]^+$ and *cis*-$[M^{III}(L'')_2(X)(Y)]^{2+}$ were M(III)=Cr, Co and X=Y=halogen has been studied in detail in the literature. The reaction rate of the aquation of species $[Cr(L'')_2F_2]^+$ and $[Cr(L'')_2(F)(X)]^+$ to the product $[Cr(L'')_2(H_2O)(F)]^{2+}$ for L"=en, phen, bpy, tn has been investigated under both acidic and alkaline conditions, [347-349] as well as in photochemical processes, [350, 351]. The experimental basis is, however, still so limited that it is difficult to draw general conclusions concerning the importance of the degrees freedom of these systems: the nature of the bidentate ligand, its bond angles, and the nature of the leaving group on the substitution kinetics.

Traditionally the process is performed as an acidic hydrolysis and it is generally found that treatment of the reactant, $[Cr(L'')_2F_2]^+$ with strong acid, (6.2) gives rise to a relatively slow replacement of one of the two fluorido ligands with water forming $[Cr(L'')_2(H_2O)(F)]^{2+}$ and an even slower replacement of the second fluorido ligand. In practice, the second aquation of fluoride is so slow that competing processes dominate. An example of this is the of treatment of *trans*-$[Cr(tn)_2(H_2O)(F)]^{2+}$ with $HClO_4$ of various concentrations with reaction times of 3-7 days (at 50 °C), which yielded mixtures of $[Cr(tn)(F)(H_2O)_3]^{3+}$, *cis*-$[Cr(tn)_2(H_2O)_2]^{3+}$ and *cis*-$[Cr(tn)_2(H_2O)(F)]^{2+}$. [349] This demonstrate that the reaction conditions required for aquation of the second fluorido ligand, can, in the case of more labile bidentate ligands as en and tn, favour displacement of the chelating ligand!

The mechanism of the aquation reaction of $[Cr(L'')_2F_2]^+$ is assumed to proceed via a protonation of coordinated fluoride. [348, 349]

$$\begin{aligned}&[Cr(L'')_2F_2]^+ + H^+ \underset{k_{-1}}{\overset{k_1}{\rightleftarrows}} [Cr(L'')_2(F\cdots H)(F)]^{2+}\\&[Cr(L'')_2(F\cdots H)(F)]^{2+} + H_2O \underset{k_{-2}}{\overset{k_2}{\rightleftarrows}} [Cr(L'')_2(H_2O)(F)]^{2+} + HF\end{aligned} \tag{6.3}$$

Bond formation between H^+ and the lone pair of the fluorido ligand weakens the Cr–F bond and allows for the second step; a slow ligand substitution of HF with water. The substitution is likely to proceed with an initially coordination of H_2O through the lone pairs of oxygen to the chromium centre as well as the hydrogen on the fluorido containing leaving group. [347]

The effect of pressure with respect to kinetic for aquation of *trans*-$[Cr(tmd)_2F_2]^+$ and *trans*-$[Cr(tmd)_2(F)(Cl)]^+$ has been studied by Chung *et al.* and they find that the mechanism is interchange-associative for *trans*-$[Cr(tmd)_2F_2]^+$ and dissociative *trans*-$[Cr(tmd)_2(F)(Cl)]^+$. [352]

If steady state condition can be assumed for $[Cr(L'')_2(F\cdots H)(F)]^{2+}$ in the reaction mechanism (6.3) then the following rate law can be obtained:

$$\frac{d[Cr(L'')_2F_2^{\ +}]}{dt} = -k_{obs}[Cr(L'')_2F_2^{\ +}][H^+] \text{ were } k_{obs} = \frac{-k_1k_2[H_2O]}{k_{-1}+k_2[H_2O]} \tag{6.4}$$

The rate coefficient, k_{-2} for the reverse will in practice be extreme small $k_{-2}\rightarrow 0$ similar will the concentration of water during reaction be practically constant $d[H_2O]/dt \approx 0$.

The presence of fluoride ions has an effect on the reaction: It has been described that the importance of the second inverse step of the reaction mechanism increases with fluoride concentration. The effect occurs when approx. half of the coordinated fluoride is aquated. [348] The influence is small and will not prevent the aquation reaction to proceed. Presence of fluoride in moderate concentrations has also been described as having a catalytic effect on reaction rate. [347] This is attributed to interaction between HF/F^- and the F···H ligand in the protonated species $[Cr(L'')_2(F\cdots H)(F)]^{2+}$. It should be noted, though, that no structures involving bifluoride coordination to Cr(III) has ever been described. Alternatively, to the described thermal aquation in which the di-fluorido complex is treated with strong acid at moderately high temperatures, a similar reaction can be performed photolytically. [18] So far, all the work describing photoaquation is mechanistically orientated and it is still to be proven, whether the method is useful for preparative purposes. Among the systems which have been studied are: *trans*-$[Cr(tn)_2F_2]Cl\cdot H_2O$, [350] *cis*-$[Cr(cyclam)F_2]ClO_4$, [351], *trans*-$[Cr(1,4\text{-cyclam})F_2]ClO_4$ and *trans*-$[Cr(1,11\text{-cyclam})F_2]ClO_4$ [353].

Synthesis of mixed aqua-fluorido complexes

Aquated complexes of the general type $[Cr(L'')_2(H_2O)(F)]^{2+}$ can be obtained from $[Cr(L'')_2F_2]^+$. This class of complexes has been studied since the 1960s and the complexes are relatively well studied for systems contaning ethane-1,2-diamine or similar saturated bidentate ligands. [340, 354-356]. The analogous 1,10-phenantroline and 2,2′-bipyridine complexes have not been

structurally or preparatively characterized to any appreciable extent. Work on systems containing these ligands have mainly focussed on the kinetic behavior of the aquation. [347, 349]
Virtually all described aquation reactions of difluorido complexes, $[Cr(L'')_2F_2]^+$ according to (6.5) rely on hydrolysis in strongly acidic solutions at moderate temperatures and with long reaction times.

$$[Cr(L'')_2F_2]X + H_2O \xrightarrow{HX} [Cr(L'')_2(H_2O)(F)]X_2 + F^- \quad (6.5)$$

Synthesis of the two geometrical isomers of $[Cr(en)_2(H_2O)(F)]$ constitute examples of this approach. Thus, *trans*-$[Cr(en)_2(H_2O)(F)](ClO_4)_2{\cdot}H_2O$ is produced by aquation of *trans*-$[Cr(en)_2F_2]Cl$ in 72% $HClO_4$ for 1 hour. [340] In a parallel fashion *cis*-$[Cr(en)_2(H_2O)(F)]I_2{\cdot}H_2O$ was obtained by treating *cis*-$[Cr(en)_2F_2]I$ with 48% HI. [355] Further systems described in the literature are the propane-1,3-diamine and 2,2′-bipyridine analogues. [347] The general anion, X^- in (6.5) is usually perchlorate or iodide but complexes with different anions have been prepared by metathesis. [340] Exchange of two fluoride ligands to yield complexes with two coordinated water molecules as shown in (6.2) is exemplified by the somewhat related systems $[Cr(L'')(H_2O)_2(F)_2]Br{\cdot}nH_2O$ with L"=en, pn, chxn. These are obtained by aquation of $(enH_2)[Cr(en)F_4]Cl$ in 48% HBr at room temperature. [357]

Anation - Reactivity of $[Cr(L'')_2(H_2O)(F)]^{2+}$

Interest in the aquation reaction, which is understood as replacement of the ligand water by an anion in a coordination entity. [346] Is partly due to the fact that the aquated compounds, $[Cr(L'')_2(H_2O)(F)]^{2+}$ have potential as starting materials *e.g.* in the synthesis of mixed fluorido complexes as $[Cr(L'')_2(X)(F)]^+$ were X is a mono anionic ligand belonging to the group of halogens or halogenoids. The overall reaction consist of aquation of $[Cr(L'')_2(F)_2]^+$ followed by anation of $[Cr(L'')_2(H_2O)(F)]^{2+}$ as shown in (6.6) and is formally equivalent to a direct ligand substitution of F^- with X^-.

$$\begin{array}{l} [Cr(L'')_2F_2]^+ + H_2O \xrightarrow{\text{Aqution}} [Cr(L'')_2(H_2O)(F)]^{2+} + F^- \\ \underline{[Cr(L'')_2(H_2O)(F)]^{2+} + X^- \xrightarrow{\text{Anation}} [Cr(L'')_2(X)(F)]^{2+} + H_2O} \\ [Cr(L'')_2F_2]^+ + X^- \longrightarrow [Cr(L'')_2(X)(F)]^+ + F^- \end{array} \quad (6.6)$$

Synthesis of mixed complexes is often difficult to accomplish due to the fact that these intermediate species, under equilibrium conditions, exist in a narrower window conditions: ligand concentrations, pH etc. Synthesis based on the aquated complex allow more control of the ligand substitution than direct substitution and is therefore potentially a more versatile approach for $[Cr(L'')_2(X)(F)]^+$.

The possibility to perform the type of ligand substitution reaction as shown in (6.6) has been studied by Vaughn *et al.* in case of fluorido containing systems based on ethane-1,2-diamine. [340] By adjusting the reaction conditions the coordinated water ligand in $[Cr(en)_2(H_2O)(F)]Y_2$ could be replaced in an anation reaction to yield series of $[Cr(en)_2(X)(F)]Y$ complexes. An idealized reaction equation together with the reaction condition of anation is given by:

$$[Cr(en)_2(H_2O)(F)]Y_2 + X^- \xrightarrow[\text{-}H_2O]{\text{sol./con.}} [Cr(en)_2(X)(F)]X + Y^- \quad (6.7)$$

Y^-	X^-	Sol./cond.	$[Cr(L'')_2(X)(F)]Y$	Ref.
ClO_4^-	NH_4Cl	MeOH	*trans*-$[Cr(en)_2(Cl)(F)]ClO_4$	[354]
ClO_4^-	NH_4Br	MeOH	*trans*-$[Cr(en)_2F_2]Br$	[354]
ClO_4^-	NH_4Br	DMF	*trans*-$[Cr(en)_2(H_2O)(F)]Br_2$	[354]
I^-	NH_4SCN	$MeOH/H_2O$	*cis*-$[Cr(en)_2(NCS)(F)]I$	[355, 356]
ClO_4^-	$NaNO_2$	H_2O	*trans* $[Cr(en)_2(O_2N)(F)]ClO_4$	[340]
Cl^-		Δ	*cis*-$[Cr(en)_2(Cl)(F)]Cl$	[340]
Br^-		Δ	*cis*-$[Cr(en)_2(Br)(F)]Br$	[340]
I^-		Δ	*cis*-$[Cr(en)_2(I)(F)]I$	[340]
SCN^-		Δ	*cis*-$[Cr(en)_2(NCS)(F)]SCN$	[340]

Table 14 Synthesis of mixed fluoride complexes by anation of $[Cr(en)_2(H_2O)(F)]^{2+}$

Comment to table: Configuration isomerism and possible crystal solvent is not included. Thus, $[Cr(en)_2(H_2O)(F)]Br_2$ actually refers to the compound $[Cr(en)_2(H_2O)(F)]Br_2{\cdot}DMF{\cdot}H_2O$.

The results shown in Table 14 seem systematic, but with some irregularities such as in the synthesis of the *trans*-$[Cr(en)_2(X)(F)]^{2+}$ for X=Cl, Br where similar reaction conditions gives different outcomes for the two anions. Also, the corresponding reaction with NH_4Br gave a somewhat unexpected result depending on solvent: *trans*-$[Cr(en)_2F_2]Br$ in MeOH and *trans*-$[Cr(en)_2(H_2O)(F)]Br_2{\cdot}DMF{\cdot}H_2O$ in DMF. [354] For tetrafluorido complexes, $[Cr(L'')F_4]^+$ where L"=en, pn, chxn, which forms $[Cr(L'')(H_2O)_2(F)_2]Br$, as described above, thermolysis result in an anation reaction yielding $[Cr(L'')(H_2O)(Br)(F)_2]$. [357]

The aquated complexes react as Brønsted acid according to:

$$[Cr(L'')_2(H_2O)(F)]^{2+} + H_2O \longrightarrow [Cr(L'')_2(OH)(F)]^+ + H_3O^+ \quad (6.8)$$

AQUATION OF DIFLUORIDO COMPLEXES BY USE OF LANTHANOIDS

In the previous sections aquation reactions and their products, as shown in equation (6.2) and (6.6) have been described for acid catalysed reactions. Here shall discuss an entirely different approach using the high affinity of lanthanoids for fluoride to facilitate the conversion of *cis*-$[Cr(L'')_2F_2]^+$ to *cis*-$[Cr(L'')_2(H_2O)(F)]^{2+}$.

$$\begin{aligned} 3\ cis\text{-}[Cr(L'')_2F_2]ClO_4 + Ln(ClO_4)_3 &\xrightarrow[\Delta]{MeCN/H_2O} \\ &3\ cis\text{-}[Cr(L'')_2(H_2O)(F)](ClO_4)_2 + LnF_3 \end{aligned} \tag{6.9}$$

Based on high affinity of Ln(III) for fluoride it could be expected that treatment of a fluorido containg coordination complex as *cis*-$[Cr(L'')_2F_2]ClO_4$ with Ln(III) would simply result in unselective abstraction of all fluorido ligands. However, this proves not to be the case. The reaction was found to yield cleanly, in high yields *cis*-$[Cr(L'')_2(H_2O)(F)](ClO_4)_2$. LnF_3 can be removed by filtration or centrifugation due to the extremely low solubility in water/acetonitrile.

This type of metal-assisted substitution is relatively well known in the context preparative chemistry as well as in quantitative analysis. Most frequently, the reaction type is used for substitution of the softer ligands. Examples include the formation of $[Co(NH_3)_5(H_2O)]^{3+}$ when the halogenide complex $[Co(NH_3)_5(X)]^{2+}$ ($X \neq F$) is treated with soft metal ions as Ag(I), Tl(III) or Hg(II). [358] The hard fluoride ion this is untouched by the soft metal ions. However, kinetic studies have shown that fluoride can be completely substituted in *e.g.* PF_6^- and AsF_4^-, forming the corresponding oxo acids when these complex fluoride containing anions are treated with very hard Th(IV) or Al(III). [359] Conversely, an example employing metal assisted ligand exchange as a preparative tool, playing on differences in hardness of metal centres and ligands, is provided by the treatment of *trans*-$[Co(py)_4Cl_2]^+$ concomitantly with HF(*aq*) and $Hg(CH_3COO)_2$ resulting in formation of *trans*-$[Co(py)_4F_2]^+$ with precipitation of $HgCl_2$. [7] Another example is the Ag(I) assisted formation of *cis*-$[Cr(en)_2(H_2O)(F)]^{2+}$ from *cis*-$[Cr(en)_2(Cl)(F)]^+$. [340] This reactivity has been rationalized by using the HSAB principle (see Chapter 2). The metal ion assisted ligand substitution suffer generally from the problem that it is difficult to gauge, in advance, the reactivity in the specific chemical system and which reaction products this will give rise to. The balance between the hardness of the outgoing ligand and the metal centre to which it is bound before reaction on one hand, and the hardness of the metal ion or metal ion complex assisting the ligand substitution on the other hand, is delicate and will dependent on a number of conditions including temperature, hydrolysis and the solubility of the products. A classic example is from the quantitative analysis of halogenido complexes, where treatment with Ag(I) in the cold leads to precipitation of ionic halogenide alone, while a similar hot treatment leads to precipitation of both ionic and complex bound halogenide.

In terms of the HSAB principle H^+ ion is classified as “hard”, and share a similar reactivity with the metal ion in assisted ligand substitution reaction. In general, terms metal ion assisted ligand substitution reactions can be viewed as interaction between the lone pair of the coordinated ligand and the incoming metal (or group), thereby weakening the dative bond between the central atom and coordinated ligand and resulting in bond cleavage.

The exact pathway (the sum of elementary reactions) for the ligand substitution is not simply deducible from the overall reaction equation. In general, ligand substitution in octahedral complexes can proceed through a number of different reaction scenarios including a dissociative, associative or interchange mechanism. A tentative description of the lanthanoid assisted aquation reaction can be supposed to proceed in a way similar to the reaction mechanism of an acid catalysed aquation.

$$\begin{aligned}
&cis\text{-}[Cr(L'')_2F_2]^+ + \{LnF_n\}^{(3-n)+} \longrightarrow cis\text{-}[Cr(L'')_2(F\cdots LnF_n)(F)]^{(4\text{-}n)+} \\
&cis\text{-}[Cr(L'')_2(F\cdots LnF_n)(F)]^{(4\text{-}n)+} + H_2O \longrightarrow \\
&\qquad\qquad cis\text{-}[Cr(L'')_2(H_2O)(F)]^{2+} + \{LnF_{n+1}\}^{(2-n)+} \\
&n \leq 2
\end{aligned} \tag{6.10}$$

Similarly to the acid catalysed aquation reaction, an initial bond formation between one (or two) of the two fluorido ligands of *cis*-$[Cr(L'')_2F_2]ClO_4$ with the Ln(III) cation will result in an inner-sphere type intermediate: Cr(III)–F–Ln(III). By aquation the Cr(III)–F bond is cleaved with concomitant coordination of water giving *cis*-$[Cr(L'')_2(H_2O)(F)](ClO_4)_2$ as product and a non-investigated Ln(III) fluoride. The simplified description of the reaction in the scheme above disregards a complication, namely the likely formation of polynuclear fluoride bridged lanthanoid clusters as intermediates preceding the ultimate precipitation of LnF_3. It is characteristic that of the relatively few structurally characterized fluoride complexes of lanthanoids, many are polynuclear. In this respect, the reaction towards formation of LnF_3 may resemble hydrolysis of aqua ions yielding clusters of increasing size and complexity along the reaction path.

It is important to note that the aquation of fluoride complexes facilitated by Ln(III) ions is not, as the acid hydrolysis, catalytic in the classical meaning a stoichiometric amount of Ln(III) is required. On the other hand, the reaction is mild and allows *cis*-$[Cr(phen)_2(H_2O)(F)]^{2+}$ to be synthesized and isolated straightforwardly. [347]

STRUCTURAL DESCRIPTION OF *cis*-DI-FLUORIDO AQUATION COMPLEXES

Structural characterized complexes in literature

While 1,10-phenantroline and 2,2′-bipyridine are very well known ligands in coordination chemistry only a limited number of Werner type complexes as, $[M^{III}(L'')_2X_2]^+$, $[M^{III}(L'')_2(X)(Y)]^{2+}$ have been structurally characterized. Examples featuring different labile metal ions are:

	Group 6	Ref.	Group 7	Ref.
**1**	*cis*-$[Cr(bpy)_2Cl_2]$	[331, 360]	*cis*-$[Mn(bpy)_2Cl_2]\cdot 2H_2O\cdot EtOH$	[361]
			cis-$[Mn(bpy)_2Cl_2]\cdot SC(NH_2)_2$	[362]
			cis-$[Mn(bpy)_2(H_2O)(Cl)]ClO_4$	[363]
			cis-$[Mn(bpy)_2Br_2]$	[364]
**2**	*cis*-$[Mo(bpy)_2Cl_2]$ $[Mo(bpy)Cl_4]$	[365]	*cis*-$[Re(bpy)_2Cl_2]Cl\cdot H_2O$	[331]
			cis-$[Re(bpy)_2Cl_2]PF_6$	[366]
	**Group 8**		**Group 9**	
**1**	*cis*-$[Fe(bpy)_2Cl_2][FeCl_4]$	[365]	*cis*-$[Co(bpy)_2Cl_2]Cl_2\cdot H_2O$	[367]
**2**	*cis*-$[Ru(bpy)_2Cl_2]\cdot 3.5H_2O$	[368]	*cis*-$[Rh(bpy)_2Cl_2]Cl\cdot 2H_2O$	[369]
	cis-$[Ru(bpy)_2Cl_2]Cl\cdot 2H_2O$	[368]		
	**Group 10**		**Group 11**	
**1**	*cis*-$[Ni(bpy)_2Cl_2]$	[370]		
	cis-$[Ni(bpy)_2(H_2O)_2](ClO_4)_2$	[371]		
**2**			*cis*-$[Cd(bpy)_2Br_2]$	[372]
			cis-$[Cd(bpy)_2I_2]$	[373]

Table 15 Selection of structurally characterized *cis*-$[M(bpy)_2X_2]Y_n$ complexes

The very limited number of fluorido complexes complicates comparison within the group and with other halogens or halogenido complexes. The range of chromium complexes can be extended somewhat by including systems that besides the two fluorido ligands possess four nitrogen ligator atoms. They are collected in the following table together with their Cr–F bonding distances. Only one other *bis*-bpy complex with fluoride co-ligands has been described: *cis*-$[V(bpy)_2F_2]BF_4$. [374] Though Ni(II) forms a related dimeric complex $[(bpy)_2(F)Ni–F–Ni(F)(bpy)_2]F\cdot 3H_2O\cdot EtOH$. [375] The crystal structure of the binuclear nickel complex reveals an

extensive hydrogen bonding network between the anionic fluorine, crystal water and ethanol solvate. The propensity to form hydrogen-bonded networks is a recurrent motif in solid state chemistry of fluoride complexes. More distantly related systems encompass the Hf(IV) complex $[Hf(bpy)_2F_4]$, [383] and the triangular dodecahedral W(VI) cation $[W(bpy)_2F_4]^{2+}$ which has been isolated and structurally characterized with different counter anions, the μ_2-fluorido-bis(oxotetrafluorido)wolframate, $2[(O)WF_4–F–WF_4(O)]^{-}\cdot$¼HF with W–F bond in the range 1.79(3) to 1.87(2) Å and the $2[WF_7]^{-}\cdot WF_6$ with W–F bond length of 1.836(4) Å, [384, 385].

Two complexes related to the aqua-fluorido complex *cis*-$[Cr(bpy)_2(H_2O)(F)]^{2+}$ have been structurally characterized: *cis*-$[Cr(bpy)_2F_2]^{+}$ and *cis*-$[Cr(bpy)_2(H_2O)_2]^{3+}$. The former has been studied by Yamaguchi-Terasaki *et al.*, [303] and the latter diaqua complex has been structural characterized by Casellato *et al.*, [386] as its nitrate salt.

Complex	Cr-F$_1$ / Å	Cr-F$_2$ / Å	Cr-F$_3$ / Å	Cr-F$_4$ / Å	Ref.
cis-$[Cr(NH_3)_4F_2]ClO_4$	1.887(6)	Symm.			[345]
trans-$[Cr(NH_3)_4F_2]I\cdot H_2O$	1.894(3)	Symm.			[345]
trans-$[Cr(py)_4F_2]PF_6$	1.853(2)	Symm.			[326]
trans-$[Cr(en)_2F_2]ClO_4$	1.887(6)	1.878(6)			[376]
cis-$[Cr(en)_2F_2]ClO_4\cdot NaClO_4\cdot H_2O$	1.887(5)	1.868(4)			[377]
trans-$[Cr(en)(H_2O)_2F_2]Cl$	1.885(1)	Symm.			[378]
trans-$[Cr(pd)_2F_2]ClO_4$	1.876(3)	1.875(3)			[379]
cis-$[Cr(bpy)_2F_2]ClO_4$	1.8541(10)	1.8409(10)			[303]
trans-$[Cr(2,3,2\text{-tet})F_2]ClO_4$	1.90(2)	1.85(2)			[380]
trans-$[Cr(1,4\text{-}C_2\text{-cyclam})F_2]ClO_4$	1.892(3)	1.884(3)			[353]
trans-$[Cr(1,11\text{-}C_3\text{-cyclam})F_2]ClO_4$	1.884(2)	1.866(2)			[353]
trans-$[Cr([15]aneN_4)F_2]ClO_4\cdot H_2O$	1.8779(12)				[343]
$[Cr(bpy)(H_2O)F_3]\cdot 2H_2O$	1.8942(19)	1.8942(19)	1.8769(18)		[381]
trans-$(Hgdn)[Cr(py)_2F_4]$	1.893(1)	1.890(1)	1.883(1)	1.880(1)	[382]
$(H_2en)[Cr(en)F_4]Cl$	1.893(8)	1.930(4)	1.889(4)	1.885(10)	[357]

Table 16 Structural characterized fluorido complexes of Cr(III)

Single crystal structural description of two *cis*-di-Fluorido aquation

The molecular structures of *cis*-[Cr(phen)$_2$F$_2$]ClO$_4$·H$_2$O and *cis*-[Cr(bpy)$_2$(H$_2$O)(F)](ClO$_4$)$_2$·2H$_2$O have been determined by single crystal X-ray diffraction. The details of the crystal parameters, data collection and refinements for the complexes are summarized in Appendix 3, " X-ray diffraction" Table 39 and Table 40. The molecular structures for the two complexes are shown in Figure 38 and Figure 39, while selected geometric parameters can be found in Table 17 and Table 18.

Both structures are triclinic and belong to space group *P*-1. The asymmetric unit of both complexes contains a Cr(III) ion in center of a distorted octahedral coordination polyhedron with a *cis* arrangement of the two bidentate heteroaromatic amine ligands and the two monodentate ligands. The coordination angles around Cr(III) indicates a significantly distortion from a ideal octahedral geometry. In case of *cis*-[Cr(phen)$_2$F$_2$]ClO$_4$·H$_2$O the bite angles of the bidentate ligands are 79.72(6) ° and 79.95(6) ° and similarly for *cis*-[Cr(bpy)$_2$(H$_2$O)(F)](ClO$_4$)$_2$·2H$_2$O 79.46(6) ° and 78.99(6) °. This deviation from the idealized angles is, of course, a result of the locked bite in both phen and bpy. These values of bite angles are comparable to those found in similar compounds: *cis*-[Cr(bpy)$_2$F$_2$]$^+$, 78.72(5) ° and 78.35(5) °, [303] *cis*-[Cr(bpy)$_2$(H$_2$O)$_2$](NO$_3$)$_3$ 80.5 ° and 78.9 °, [386] and *cis*-[V(bpy)$_2$F$_2$]BF$_4$ 75.01(7) ° and 74.59(7) °, [374] but also close to data for unrelated complexes as *e.g.* the copper complex *cis*-[Cu(phen)$_2$(Cl)(NCS)] 80.6(2) ° and 75.6(2) °, [387]. In case of the more flexible bidentate ligands such as ethane-1,2-diamine and propane-1,3-diamine a wider range of bite angles is seen.

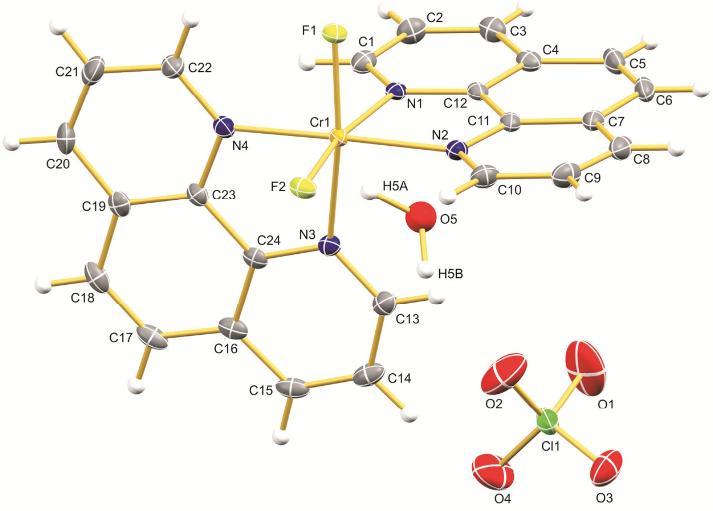

Figure 38 Molecular structure of *cis*-[Cr(phen)$_2$F$_2$]ClO$_4$·H$_2$O

cis-[Cr(en)$_2$F$_2$]$^+$ 82.9(3) ° and 81.3(3) °, [377] *trans*-[Cr(pd)$_2$F$_2$]$^+$ 87.7(2) ° and 88,1(2) °, [379]. The locked bite angle will naturally affect the remainding ligands. One must therefore be cautious with interpretation of the bonding angles and distances between these. Nevertheless, the angle between the two monodentate ligands in *cis*-[Cr(bpy)$_2$F$_2$]ClO$_4$·H$_2$O, *cis*-[Cr(bpy)$_2$(H$_2$O)(F)](ClO$_4$)$_2$·2H$_2$O and *cis*-[Cr(bpy)$_2$(H$_2$O)$_2$](NO$_3$)$_3$ are 95.43(5) °, 90.04(5) ° and 86.6 ° respectively, which is close to the expected idealized angle. The bond distances between the Cr(III) centres and fluorido ligands is 1.8444(10) Å and 1.8621(10) Å for *cis*-[Cr(phen)$_2$F$_2$]ClO$_4$·H$_2$O and 1.8614(8) Å for *cis*-[Cr(bpy)$_2$(H$_2$O)(F)](ClO$_4$)$_2$·2H$_2$O. In both cases they are clearly within the range of bond lengths 1.85-1.90 Å which is found in literature for analogous complexes. Generally, the bond length between Cr(III) and the fluorido ligand is relatively constant even when comparing complexes with even large variations in number of fluorido ligands and auxiliary ligand sphere. This is seen when comparing the average distances in the two very different complexes, *cis*-[Cr(NH$_3$)$_4$F$_2$]ClO$_4$ Cr–F$_{av}$: 1.887(6), [345] and [Cr(bpy)(H$_2$O)F$_3$]·2H$_2$O Cr–F$_{av}$: 1.8884(19) Å.

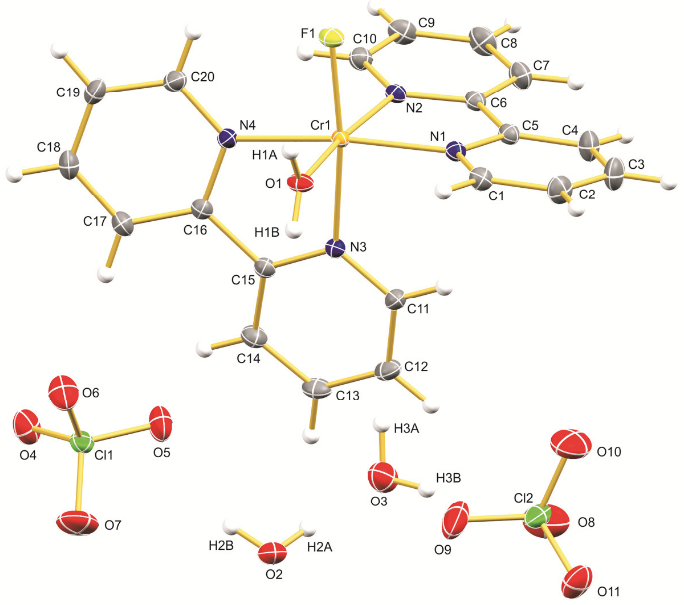

Figure 39 Molecular structure of *cis*-[Cr(bpy)$_2$(H$_2$O)(F)](ClO$_4$)$_2$·2H$_2$O

Hydrogen atoms are named in accordance with the parent carbon atom.

Bond length (Å)		Bond angle (°)			
Cr_1–F_2	1.8444(10)	F_2–Cr_1–F_1	95.92(5)	F_1–Cr_1–N_1	88.67(5)
Cr_1–F_1	1.8621(10)	F_2–Cr_1–N_4	92.33(5)	N_4–Cr_1–N_3	79.95(6)
Cr_1–N_4	2.0566(15)	F_2–Cr_1–N_3	89.38(5)	N_4–Cr_1–N_2	174.40(5)
Cr_1–N_3	2.0797(16)	F_2–Cr_1–N_2	91.83(6)	N_4–Cr_1–N_1	95.83(6)
Cr_1–N_2	2.0607(15)	F_2–Cr_1–N_1	170.54(5)	N_3–Cr_1–N_1	87.34(6)
Cr_1–N_1	2.0934(15)	F_1–Cr_1–N_4	91.42(5)	N_3–Cr_1–N_2	96.36(6)
		F_1–Cr_1–N_3	170.08(5)	N_2–Cr_1–N_1	79.72(6)
		F_1–Cr_1–N_2	91.86(5)		

Table 17 Selected geometric parameters for *cis*-[Cr(phen)$_2$F$_2$]ClO$_4$·H$_2$O

Bond length (Å)		Bond angle (°)			
Cr_1–F_1	1.8614 (8)	F_1–Cr_1–O_1	90.26 (4)	O_1–Cr_1–N_3	90.13 (4)
Cr_1–O_1	1.9579 (10)	F_1–Cr_1–N_1	94.23 (4)	O_1–Cr_1–N_4	88.50 (5)
Cr_1–N_1	2.0501 (12)	F_1–Cr_1–N_2	89.57 (4)	N_1–Cr_1–N_2	79.51 (5)
Cr_1–N_2	2.0456 (12)	F_1–Cr_1–N_3	172.26 (4)	N_1–Cr_1– N_3	93.46 (5)
Cr_1–N_3	2.0545 (12)	F_1–Cr_1–N_4	93.04 (4)	N_1–Cr_1–N_4	172.47 (5)
Cr_1–N_4	2.0571 (12)	O_1–Cr_1–N_1	93.41 (5)	N_2–Cr_1–N_3	90.99 (5)
		O_1–Cr_1–N_2	172.88 (5)	N_2–Cr_1–N_4	98.62 (5)
				N_3–Cr_1–N_4	79.24 (5)

Table 18 Selected geometric parameters for *cis*-[Cr(bpy)$_2$(H$_2$O)(F)](ClO$_4$)$_2$·2H$_2$O

Crystal packing

The crystal packing of the two complexes are shown in Figure 40 and Figure 41. *cis*-[Cr(phen)$_2$F$_2$]ClO$_4$·H$_2$O reveals a distinct, π-π interaction between the aromatic ring systems of the 1,10-phenantroline ligands. The interaction occurs between two of the aromatic rings in the ligand, shown by ring-centroids at Figure 40 thus forming a centrosymmetric dimer. The ring-centroids *Cg*1 and $Cg2^i$ formed by $N_3C_{13\text{-}16}C_{24}$ and $C_{16\text{-}19}C_{23\text{-}24}$ (symmetrycode (i): −x+1, −y+1, −z+1) between the two formula units in the unit cell is spaced at 3.532 Å, which is comparable to the π-stacking distances of 3.56(1) Å found in *cis*-[Cr(bipy)$_2$F$_2$]ClO$_4$, [303] and 3.56(2) Å in *cis*-[V(bipy)$_2$F$_2$]BF$_4$, [374]. This type of packing with π-π ring interaction is common in structures of complexes with bidentate aromatic ligands (as phen and bpy) and in many cases independent of the central atom, auxiliary ligands and counter ions. Conversely, in *cis*-[Cr(bipy)$_2$(H$_2$O)(F)](ClO$_4$)$_2$·2H$_2$O, the cations are isolated from each other by bands of crystal water and anions and do not show evidence of π-π interaction between the aromatic ring systems. This can be taken as evidence of the weakness of π-π packing interactions which become disfavored when other stronger interactions are possible.

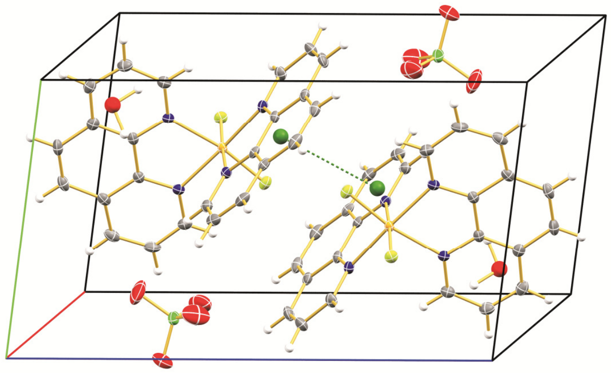

Figure 40 Crystal packing of *cis*-[Cr(phen)$_2$F$_2$]ClO$_4$·H$_2$O

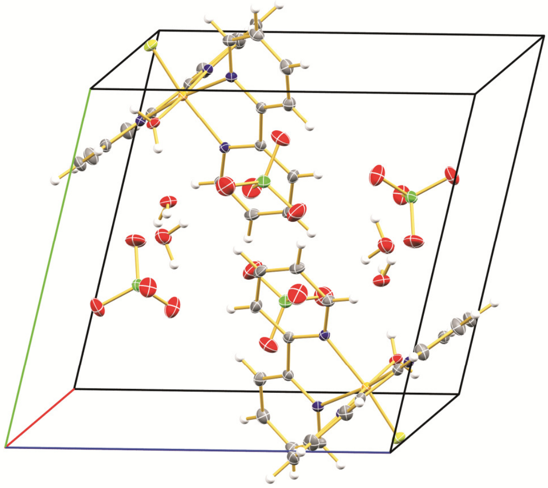

Figure 41 Crystal packing of *cis*-[Cr(bpy)$_2$(H$_2$O)(F)](ClO$_4$)$_2$·2H$_2$O

Trans influenced bond lengths in *cis*-[Cr(L")$_2$F$_2$]ClO$_4$

Comparing the bond distance from the central atom to the nitrogen ligator atoms, it is seen that the distances depend on their disposition realtive to the fluorido ligands. The trend seems to be that the bond length Cr–N *cis* to Cr–F is *shorter* then Cr–N *trans* to Cr–F. This variation is discussed by Yamaguchi-Terasaki *et al.* for *cis*-$[Cr(bpy)_2F_2]^+$, where Cr–N *cis* to Cr–F: 2.0480(14) Å, 2.0511(14) Å and Cr–N *trans* to Cr–F: 2.0680(13) Å, 2.0842(14) Å. [303] The same situation exists in the analogous V(III) complex *cis*-$[V(bpy)_2F_2]^+$. [374] Similarly, *trans* influenced bond lengths are found in *cis*-$[Cr(phen)_2F_2]^+$ were Cr–N *cis* to Cr–F, at 2.0607(15) Å and 2.0566(15) Å are considerable shorter than Cr–N *trans* to Cr–F at 2.0934(15) Å and 2.0797(16) Å. The described effect is very pronounced in this type of compounds but is not easy to generalize to systems with different bidentate ligands or systems with only one fluorido ligand as in seen for *cis*-$[Cr(bpy)_2(H_2O)(F)](ClO_4)_2{\cdot}2H_2O$. This system can be compared to the di-fluorido complexes either by comparing the fluorido ligand without concern for the coordinated water or by comparing with both fluorido ligand and water. On the basis of Figure 39 the first case shows that ligator atoms N_1, N_2 and N_4 are in *cis* position with respect to F_1 while N_3 is in *trans* position. The second description categorizes ligator atom N_1 and N_4 to be in *cis* positions with respect to F_1 and O_1 while N_2 and N_3 are in *trans* positions. Comparing this with Table 18 shows that none of the two cases displays distinctly *trans*-influenced Cr–N bond lengths. Also in *cis*-$[Cr(en)_2F_2]^+$ the variation in the Cr–N bond lengths is too large to allow for any conclusions concerning *trans*-influences (Cr–N *cis* to Cr–F: 2.083(8) Å, 2.073(9) Å and Cr–N *trans* to Cr–F: 2.067(6) Å, 2.100(8) Å). [377]

Hydrogen bonding and binuclear units

Hydrogen bonding in its general form D–H···A is seen in both complexes between the donor entity O–H originating from both coordinated and crystal water towards the accepting fluorido ligand. A graphic representation of the strong intermolecular hydrogen bonding motifs in the two structures are shown in Figure 42 and Figure 43.Values for the length and angles of the hydrogen bonds are given in Table 20 and Table 21. Based on its properties hydrogen bonds can be classified as strong, moderate or weak, and an approximate binding energy can be assigned. Values for the length and angles of the general hydrogen bonds D–H···A are given in Table 19. Typical hydrogen bond lengths for the O–H···F and O–H···O bonding motifs are 2.65-2.87 Å and 2.48-2.90 Å respectively.

The hydrogen bonding in *cis*-$[Cr(phen)_2F_2]ClO_4{\cdot}H_2O$, Figure 42 and *cis*-$[Cr(bpy)_2(H_2O)(F)](ClO_4)_2{\cdot}2H_2O$, Figure 43, is determined primarily by coordinated and, most importantly crystal water. Based on the classification principles shown of Table 19 hydrogen bonding in *cis*-$[Cr(phen)_2F_2]ClO_4{\cdot}H_2O$ in the form of O–H···F and O–H···O between the crystal water and the fluorido ligand/counter anion respectively, can be classified as moderate to strong.

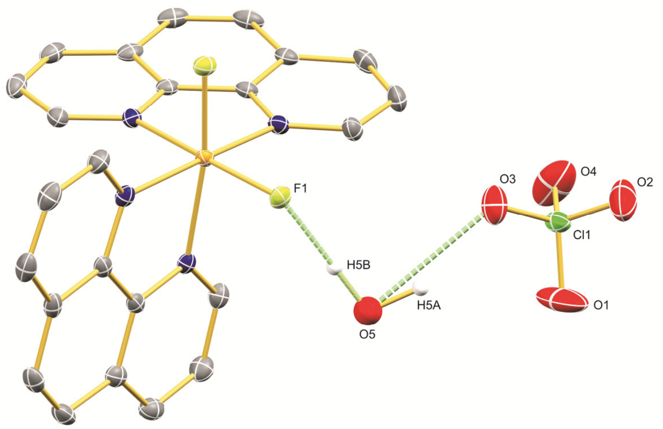

Figure 42 Hydrogen bonding in *cis*-[Cr(phen)$_2$F$_2$]ClO$_4$·H$_2$O

H-bond shown with dashed green line.
Hydrogen atoms of the bpy ligands are omitted for clarity.

Table 20 shows that a clear difference both in terms of bond lengths and angles exist between the hydrogen bond involving fluorine, 2.7183 (19) Å and oxygen, 3.006 (2) Å respectively as donor atom with the former as the strongest. It is notable that the hydrogen bond pattern found in *cis*-[Cr(phen)$_2$F$_2$]ClO$_4$·H$_2$O is not replicated in the analogues *cis*-[Cr(bpy)$_2$F$_2$]ClO$_4$, [303] and *cis*-[V(bpy)$_2$F$_2$]BF$_4$, [374] which both crystallize without crystal water. Both of those compounds instead show weak intermolecular hydrogen bonding (contact interaction) in the form of C–H···F and C–H···O between hydrogen on the aromatic ring systems and the fluorido ligand/counter anion respectively. In case of *cis*-[Cr(bpy)$_2$F$_2$]ClO$_4$ these interactions result in average H···F/C···F bond distances of 2.38 Å/3.152(2) Å and while they are somewhat longer in *cis*-[V(bpy)$_2$F$_2$]BF$_4$ at 2.45 Å/3.200(3) Å. Regarding hydrogen bonding with the counter ions average H···O/C···O (ClO_4^-) bond distances of 2.53 Å/3.293(3) Å are found in *cis*-[Cr(bpy)$_2$F$_2$]ClO$_4$ while *cis*-[V(bpy)$_2$F$_2$]BF$_4$ has 2.51 Å/3.303(3) Å for the average H···F/C···F (BF_4^-) distances.

	Strong	Moderate	Weak
D–H···A	covalent	mostly electrostatic	electrostatic
D–H (Å)	1.2-1.5	1.5-2.2	2.2-3.2
H···A (Å)	2.2-2.5	2.5-3.2	3.2-4.0
∠D–H···A (°)	175-180	130-180	90-150
Bond energy (kJ·mol^{-1})	59-167	17-63	<17

Table 19 Classification of hydrogen bonds according to properties Ref. [388]

The same type of interaction is found in *cis*-[Cr(phen)$_2$F$_2$]ClO$_4$·H$_2$O, for which values of the C–H···F and C–H···O bond are given in Table 21. Average values for comparison with literature are 2.433(3) Å/ 3.106 (2) for the H···F/C···F bonds and 2.555 (2) Å/ 3.365 (3) Å for H···O/C···O (ClO_4^-). It is difficult to generalize solely on the basis of the data above, thoughit is seen that the H···F in the C–H···F bond increase along the series: *cis*-[Cr(bpy)$_2$F$_2$]ClO$_4$ < *cis*-[Cr(phen)$_2$F$_2$]ClO$_4$·H$_2$O < *cis*-[V(bpy)$_2$F$_2$]BF$_4$, which be taken as evidence of decent Lewis basicity of the fluoride ligands coordinated to chromium. In *cis*-[Cr(phen)$_2$F$_2$]ClO$_4$·H$_2$O most of the tabulated hydrogen bonds are relatively long, when compared to *cis*-[Cr(bpy)$_2$(H$_2$O)(F)](ClO$_4$)$_2$·2H$_2$O, it is evident that the packing in the structure of the latter compound is determined by hydrogen bonding between neighbouring molecules as well as between the crystal water and perchlorate anions.

D–H···A (Å)	D–H (Å)	H···A (Å)	D···A (Å)	∠D–H····A (°)
O_5–H_{5b}···F_{1i}	1.029	1.692	2.7183 (19)	174.84
O_5–H_{5a}···O_3	1.044	2.023	3.006 (2)	155.83

Symmetry codes: (i) x−1, y, z.

Table 20 Strong hydrogen-bond geometry for *cis*-[Cr(phen)$_2$F$_2$]ClO$_4$·H$_2$O

D–H···A (Å)	D–H (Å)	H···A (Å)	D···A (Å)	∠D–H···A (°)
C_3–H_3···O_{1i}	0.950	2.441(2)	3.286(3)	148.1(1)
C_5–H_5···O_{1i}	0.950	2.515(2)	3.340(3)	145.2(1)
C_6–H_6···F_{1ii}	0.950	2.431(1)	3.198(2)	137.6(1)
C_8–H_8···O_{5i}	0.950	2.544(2)	3.421(3)	153.7(1)
C_9–H_9···O_{5iii}	0.950	2.539(2)	3.433(3)	156.8(1)
C_{14}–H_{14}···F_{2iv}	0.950	2.386(1)	2.983(2)	120.6(1)
C_{14}–H_{14}···O_4	0.950	2.628(2)	3.248(3)	123.4(1)
C_{15}–H_{15}···F_{2iv}	0.950	2.576(1)	3.073(2)	112.9(1)
C_{17}–H_{17}···F_{2ii}	0.950	2.3392(9)	3.168(2)	145.5(1)
C_{20}–H_{20}···O_{4ii}	0.950	2.531(2)	3.279(3)	135.8(1)
C_{21}–H_{21}···O_{4v}	0.950	2.568(2)	3.378(3)	143.4(1)
C_{22}–H_{22}···O_{3v}	0.950	2.672(2)	3.537(2)	151.7(1)

Symmetry codes: (i) 1–x,1–y,–z; (ii) 2–x,1–y,–z; (iii) 1+x,–1+y,z; (iv) –1+x,y,z; (v) 1+x,1+y,z

Table 21 Weak hydrogen-bond geometry for *cis*-[Cr(phen)$_2$F$_2$]ClO$_4$·H$_2$O

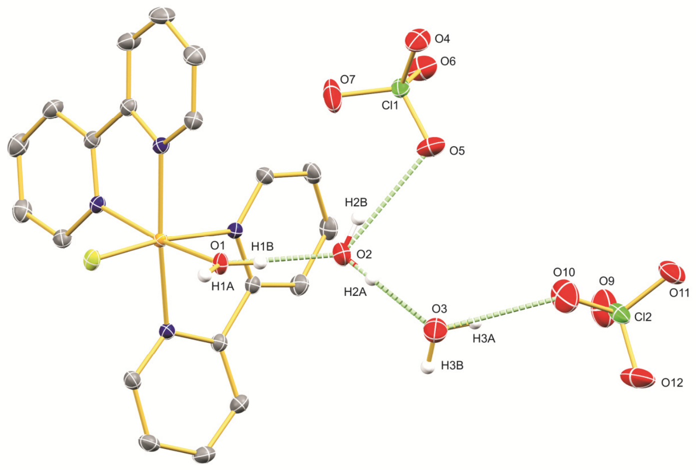

I: H-bonding network between water and ClO_4^- anions

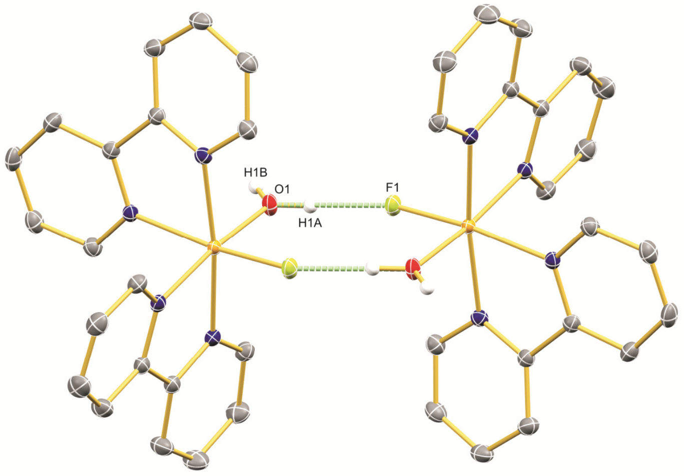

II: Dimerization between formula units, *cis*-$[Cr(bpy)_2F_2]^{2+}$ through H-bonding.

Figure 43 Hydrogen bonding in *cis*-$[Cr(bpy)_2(H_2O)(F)](ClO_4)_2 \cdot 2H_2O$

H atoms of the bpy ligands are omitted for clarity.

The crystal packing of *cis*-[Cr(bpy)$_2$(H_2O)(F)](ClO_4)$_2$·2H_2O is shown in Figure 43 together with the representation of hydrogen bonding patterns. The structure consists of dimeric entities established through hydrogen bonding between, the fluorido ligand and coordinated water in the next formula unit. In its most extreme form, these entities can be represented as a distinct binuclear unit *cis*-[(L")$_2$Cr(HO–H···F)$_2$Cr(L")$_2$]$^{4+}$ with a O–H···F hydrogen bond of length 2.5482(13) Å and angle 174 °. This hydrogen bond is considerably shorter than the the O–H···F bond in *cis*-[Cr(phen)$_2$$F_2$]$ClO_4$·$H_2O$ at 2.7183(19) Å. This could reflect a stabilization achieved by dimerization, but also the increased Brønsted acidity of coordinated water is likely to be important. Typical hydrogen bond lengths for the O–H···F bonding motif is 2.65-2.87 Å. This justifies a description of the bond as being quite short and thus probably also strong. (The strength of the hydrogen bond described by the enthalpy of dissociation, ΔH with respect to the constituents of the binding pair of the D–H···A shows for gas phase measurements that F–H···OH_2 38 kJ·mol^{-1}, HO–H···F^- 98 kJ·mol^{-1})

The configuration around the Cr(III) center with two bidentate ligands in *cis*-[Cr(bpy)$_2$(H_2O)(F)](ClO_4)$_2$·2H_2O means that the complex can occur in two different enantiomeric conformations, Δ and Λ. The product obtained by synthesis is a racemic mixture of these two forms. With a conjecture of two chiral centres, such as in the hydrogen bonded dimers the possibilities of isomerism become the chiral dimers (Δ, Δ) and (Λ, Λ) together with the achiral *meso* form (Δ, Λ). The structure of *cis*-[Cr(bpy)$_2$(H_2O)(F)](ClO_4)$_2$·2H_2O, Figure 43 reveals that the dimer has the (Δ, Λ) meso configuration ensured by the crystallographic inversion centre on which each of the dimers is located. This configuration may explain the slightly longer distance of 5.1394(6) Å between the two chromium centres compared to that observed in systems with similar composition, but where the units in the dimer have the same chirality *e.g.* the *cis*-[Cr(Δ,Δ/Λ,Λ–L")$_2$(H_2O)(OH)]$^{4+}$ complexes L"=bpy: 5.03 Å, [389] pico: 5.118(2) Å, [390] bispictn: 4.797(1) Å, [390] (all as iodides).

The crystallographic symmetry ensures also that *cis*-[Cr(bpy)$_2$(H_2O)(F)](ClO_4)$_2$·2H_2O has a torsion angle φ (Cr–F···F–Cr) of 180°, thus all atoms spanning the core of the dimer are coplanar. The meso configuration and the resulting planarity of the dimer is in agreement with what has been observed for analogous hydroxo-water bridged dimers. [47] The preference for this configuration could originate partly from unfavorable non-bonded interactions between hydrogens. However, the bridges in the present dimer are so long that it is probable that the driving force for crystallization of the meso form is the improved crystal packing of the centro-symmetric units.

Configuration of $[Cr(L'')_2F_2]^+$ and $[Cr(L'')_2(H_2O)(F)]^{2+}$

As mentioned in the beginning of the chapter, bidentate ligands like ethan-1,2-diamine, 1,10-phenantroline and 2,2′-bipyridine will in principle give rise to several configuration isomers in mixed ligand octahedral complexes of, $[M^{III}(L'')_2X_2]^+$, $[M^{III}(L'')_2(X)(Y)]^{2+}$ depending on the disposition of the ligands around the metal centre. Complexes can broadly be divided into two groups: systems for which only *cis* or *trans* configuration is observed, and those which exist in both configurations. It is found that the relative stabilities of such configuration isomers depend strongly on the nature of the bidentate ligands. In case of aromatic 1,10-phenantroline and 2,2′-bipyridine, the configuration will almost exclusively be *cis* while the complexes of ethane-1,2-diamine are commonly found in both forms. Early claims of synthesis and characterization of phen or bpy complexes with *trans* configuration have all been disproved. For example, the claimed "*trans*-$[Co(phen)_2Cl_2]Cl{\cdot}HCl{\cdot}3H_2O$", [391] was shown instead to be *cis*-$[Co(phen)_2Cl_2]_2[CoCl_4]{\cdot}2H_2O$. [330] However, since the work by Schäffer and Josephsen a few exceptions have established. Thus, both configurational isomers of $[Ru(bpy)_2Cl_2]$ are known. The *cis* complex, [392] is obtained by direct reaction of $RuCl_3$ and 2,2′-bipyridine whereas the *trans*-$[Ru(bpy)_2Cl_2]$, [393] is obtained in a obscure manner. Also examples of cationic Ru(II) complexes of both phen and bpy have been characterized. [332]

The *cis* arrangement of the ligands in *cis*-$[Cr(phen)_2F_2]^+$ and *cis*-$[Cr(bpy)_2(H_2O)(F)]^{2+}$ found here, thus agree with the expectations. Despite the fact that the configurations of these complexes appears trivial, it must be remembered that the generalizations referred to above have been made predominantly based on systems with chlorido ligand(s). Delavar *et al.* discuss the isomerism in the aquated species *cis*-$[Cr(L'')_2(H_2O)(F)]^{2+}$ with L''=en, phen, bpy and concludes on the basis of the preferred *cis* configuration in complexes as $[M^{III}(L'')_2(X)_2]^+$ that this will also be the preferred configuration of the aquated species. [347] While the conclusion probably is correct, it should be remembered that fluorine occupies a special position among the group of halogens and fluorido complexes frequently have properties deviating from those of the other halogenido complexes.
In summary, the structure of *cis*-$[Cr(phen)_2F_2]ClO_4{\cdot}H_2O$ is in line with the results of Yamaguchi-Terasaki *et al.*, [303] for the analogous bpy complex and corroborates the rule concerning the prevalence of *cis* configuration in bis-complexes of phenanthroline and bipyridine. The aquated bipyridine complex represents the first single crystal X-ray diffraction study of this type of structure. It also follows the rule and thereby bridges to the already known structure of the di-aqua complex.

X-ray powder analysis of *cis*-$[Cr(L'')_2(H_2O)(F)](ClO_4)_2$

Unlike *cis*-$[Cr(bpy)_2(H_2O)(F)](ClO_4)_2{\cdot}2H_2O$ the analogous phenanthroline complex proved very difficult to obtain in a crystalline form suitable for single crystal diffraction. Consequently, powder diffraction was performed on both compounds in order to decide if these could be isostructural. The details of the data collection for the complexes are summarized in Appendix 3 "X-ray diffraction". The powder patterns, Figure 44 shows that for *cis*-$[Cr(bpy)_2(H_2O)(F)](ClO_4)_2{\cdot}2H_2O$

the experimental powder spectrum can be simulated based on the crystal structure. Comparison of the X-ray powder diffractograms for the two complexes show distinct differences ruling out that the two complexes are isostructural. This may be due the large variation in the content of crystal water found in *cis*-[Cr(phen)$_2$(H_2O)(F)](ClO_4)$_2$·nH_2O (n=½-3½) and the ease with which the number lattice water can be changed. Loss of crystal water can be detected even at room temperature and is pronouncedly dependent on humidity. The single crystal structure of *cis*-[Cr(bpy)$_2$(H_2O)(F)](ClO_4)$_2$·2H_2O shows the importance of crystal water and the hydrogen bonding network it forms with the counter ions and the molecular entity. Modification of crystal water contents is therefore expected to have a significant influence on the packing pattern in the crystal.

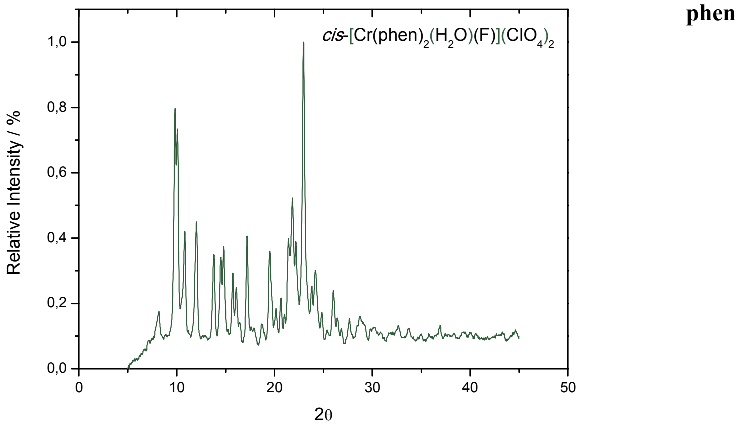

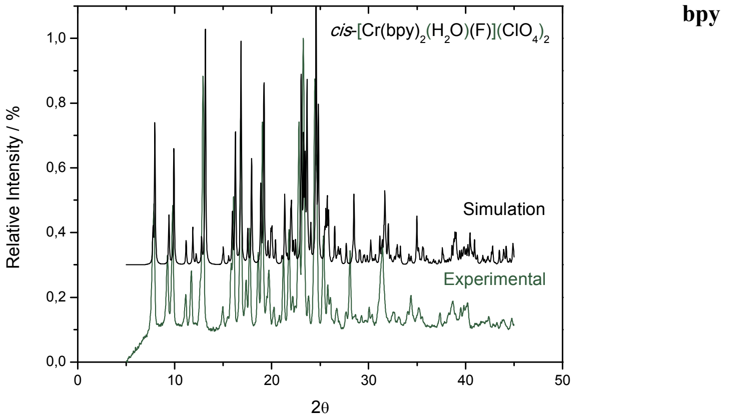

Figure 44 X-ray powder pattern of *cis*-[Cr(L")$_2$(H_2O)(F)](ClO_4)$_2$ for L"=phen, bpy

ATTEMPTS AT SYNTHESIS OF DI-μ-FLUORIDO BRIDGED COMPLEXES

As discussed above, the structure of *cis*-$[Cr(bpy)_2(H_2O)(F)](ClO_4)_2{\cdot}2H_2O$ is at least in the solid phase composed of dimeric entities, established through double-bridged hydrogen bonding between the coordinated water and fluorido ligands on two different molecular entities yielding *cis*-$[(L'')_2Cr(HO–H{\cdots}F)_2Cr(L'')_2]^{4+}$ building blocks. This structural dimeric motif is known in the literature for Cr(III) complexes with an isoelectronic ligand sphere formed by water and the hydroxido ligand, OH^-. The entity (Ardon-ligand) $[HO–H{\cdots}OH]^-$ is formed when an aqueous solution of *cis*-$[Cr(L'')_2(H_2O)_2]$ is treated with base. Some of these dimeric systems with HO–H···OH bridges exhibit interesting condensation reactions leading to much investigated dihydroxido-bridged dinuclear systems (diols). Therefore, a short introduction to oxygen bridged Cr(III) complexes is in its place.

Oxygen bridged Cr(III) complexes

The fluoride and hydroxide ions are isoelectronic giving rise to a presumption of similarity with respect to structure and reactivity. This is to some extent a valid as evidenced by the fact that many reactions which involve hydroxido species of Cr(III) have equivalent counterparts with fluoride. The two ligands are also seen to some extent to be interchangeable with each other by simple ligand substitution reactions.

However, it is important to note that the hydroxido ligand, in contrast to fluorido ligand can engage in protolysis reactions forming the water ligand, H_2O or oxido ligand, O^{2-}.

The large number of Cr(III) complexes with OH^- or O^{2-} ligands is due to the possibility of forming polynuclear systems through bridging. Formally, the polynuclear complexes can be considered as formed by deprotonation of aqua complexes. For example, the hexaaqua ion, $[Cr(H_2O)_6]^{3+}$ is quite acidic (pK_a=3.89) and deprotonation is facile even with quite weak bases. Initially leading to formation of the hydroxido complex $[Cr(H_2O)_5(OH)]^{2+}$ which can form polynuclear species such as the μ-OH or di-μ-OH bridged dimers $[(H_2O)_5Cr(\mu\text{-}OH)Cr(H_2O)_5]^{5+}$ and $[(H_2O)_4Cr(\mu\text{-}OH)_2Cr(H_2O)_4]^{4+}$ formed by condensation with $[Cr(H_2O)_6]^{3+}$ or itself, respectively.

Simplifications can be achieved in systems where the coordination sphere besides the bridging ligand has other non-bridging ligands serving as “terminators” or “capping ligands” for terminateon of the condensation processes. Most common in this role are ligands containing nitrogen ligators like NH_3, en, phen and bpy which allow for isolation of complexes such as $[(L'')_2Cr(\mu\text{-}OH)_2Cr(L'')_2]^{4+}$ for L''=en, phen, [394] bpy, [394]. Further deprotonation of the bridges is possible. Hence, reaction with 2 M NaOH or 10 M NaOH results in the formation of the mixed μ-OH-μ-O and di-μ-O bridged complex respectively. [394], [335] The most famous example of more complicated hydroxo-bridged structures is the tetra-nuclear Cr(III) complex, $[Cr_4(OH)_6(NH_3)_{12}]^{6+}$ (“Rhodoso”) synthesized by Jørgensen in 1892. [395] The structure of the bromide $[Cr_4(OH)_6(NH_3)_{12}]Br_6{\cdot}2H_2O$ shows that the four Cr(III) centres are connected through μ-

OH bridges in an rhomboid eight membered ring. Furthermore the Cr centres coordinates in pairs, either four NH_3 ligands or two μ-OH double bridges. [396]

The described reactivity can be summarized in some general reaction equations:

- Formation of di-aqua complex *cis*-$[Cr(L'')_2(H_2O)_2]^{3+}$ from a suitable starting material and the ligand, L''.

$$[Cr(H_2O)_6]^{3+} + 2\ L'' \rightleftharpoons cis\text{-}[Cr(L'')_2(H_2O)_2]^{3+} + 4\ H_2O \qquad (6.11)$$

- Deprotonization of one of the aqua ligands in *cis*-$[Cr(L'')_2(H_2O)_2]^{3+}$ to yield the aqua-hydroxido complex, *cis*-$[Cr(L'')_2(OH)(H_2O)]^{2+}$.

$$cis\text{-}[Cr(L'')_2(H_2O)_2]^{3+} + OH^- \rightleftharpoons cis\text{-}[Cr(L'')_2(OH)(H_2O)]^{2+} + H_2O \qquad (6.12)$$

- Condensation of two *cis*-$[Cr(L'')_2(OH)(H_2O)]^{2+}$ units to the di-μ-OH complex, $[(L'')_2Cr(\mu\text{-}OH)_2Cr(L'')_2]^{4+}$.

$$2\ cis\text{-}[Cr(L'')_2(OH)(H_2O)]^{2+} \rightleftharpoons [(L'')_2Cr(\mu\text{-}OH)_2Cr(L'')_2]^{4+} + 2\ H_2O \qquad (6.13)$$

- Formation of either the mixed μ-OH-μ-O, $[(L'')_2Cr(\mu\text{-}OH)(\mu\text{-}O)Cr(L'')_2]^{3+}$ or the di-μ-O bridged complex, $[(L'')_2Cr(\mu\text{-}O)_2Cr(L'')_2]^{2+}$ by deprotonization.

$$[(L'')_2Cr(\mu\text{-}OH)_2Cr(L'')_2]^{4+} + n\ OH^- \rightleftharpoons$$
$$[(L'')_2Cr(\mu\text{-}OH)_{2-n}(\mu\text{-}O)_nCr(L'')_2]^{(4-n)+} + n\ H_2O \qquad (6.14)$$
$$n = 1,2$$

It is well known that the reaction (6.13) is part of the complex series of pH dependent equilibria that exist in Cr(III)/ammine system and that the desired species will be stable in a limited pH-range. In a general equilibrium description, condensations like (6.13) will be favoured not only by the right degree of protonization, but also the total concentration will be important. This renders the possibility of solid state reactions interesting. The ability to perform the ligand substitution of water through thermolysis of the *cis*-$[Cr(L'')_2(OH)(H_2O)]^{2+}$ complex in the solid phase is one of the classic experiments from the childhood of coordination chemistry. [397]

$$2\ cis\text{-}[Cr(L'')_2(OH)(H_2O)]^{2+} \xrightarrow{\Delta} [(L'')_2Cr(\mu\text{-}OH)_2Cr(L'')_2]^{4+} + 2\ H_2O \qquad (6.15)$$

A range of *cis*-$[Cr(L'')_2(OH)(H_2O)]^{2+}$ complexes has in recent times been investigated structurally by single crystal diffraction. It was found that the solid state structure of these mixed hydroxide-aqua complexes was determining regarding their ability to engage in thermolysis reactions as the one above. Ardon, *et al.*, [389] states that:

> *"Olation of a hydroxoaqua-metal complex in the solid state may take place if and only if it is a dimer."*

The bridges formed by hydrogen bonding between the ligands H_2O and OH^- is frequently referred to as an independent entity $H_3O_2^-$, which is justified by the fact that the bond distances and angles in the bridging unit cannot be specifically attributed to one of the original ligands due to the strong hydrogen bonding. Examples of binuclear clusters with this bridging motif are *cis*-$[Cr(L'')_2(H_3O_2)]_2I_4{\cdot}2H_2O$ L"= bpy, [398, 399], pico, [389, 390], *cis*-$[Cr(cyclam)(H_2O)(OH)]^{2+}$, [400] and *cis*-$[Cr(L)(H_3O_2)]^{2+}$ for L=bispicen, bispicMe$_2$en, [400], bispictn, [390]. All of these except the bispicen complex, have been structurally characterized. *trans*–configuration in conjunction with the Ardon ligand leads to chain formation *e.g.* in *trans*-$[Cr(py)_2(H_3O_2)_2]Cl$, [287] and *trans*-$[Co(en)_2(H_3O_2)]ClO_4$, [399].

Conformation and possibility of fluorido bridged complexes

The dimeric structure of the unit *cis*-$[(bpy)_2Cr(HO\text{–}H\cdots F)_2Cr(bpy)_2]^{4+}$ as shown in Figure 43, places the monodentate ligands in such a manner that one by analogy to the oleation discussed above, could hope for condensation reactivity, for example by thermolysis. Possible reaction products of such a condensation could be the fluorido bridged $[(L'')_2Cr(\mu\text{-}F)_2Cr(L'')_2]^{4+}$ cation obtained by elimination of coordinated water, or hydroxido bridges formed by emission of hydrogen fluoride:

$$2\ cis\text{-}[Cr(L'')_2(H_2O)(F)](ClO_4)_2 \xrightarrow{\Delta} [(L'')_2Cr(\mu\text{-}OH)_n(\mu\text{-}F)_mCr(L'')_2](ClO_4)_4 + m\,H_2O + n\,HF \qquad (6.16)$$

$(n,m) = (0,2), (1,1), (2,0)$

Were (n,m) of (0,2), (1,1), (2,0) correspond to the di-μ-fluorido, μ-hydroxido-μ-fluorido and di-μ-hydroxido complexes respectively.

The feasibility of using thermally driven condensation reactions to yield fluoride-bridged chromium complexes deserved a little attention as it would represent a completely new route to this bridging ligand.

Thermolysis of *cis*-[Cr(L")$_2$(H$_2$O)(F)](ClO$_4$)$_2$·nH$_2$O

Samples of the *cis*-[Cr(L")$_2$(H$_2$O)(F)](ClO$_4$)$_2$·nH$_2$O L"=phen, bpy were heated in an oven at 140-150 °C for 4 days on a platinum plate. Heating resulted for both compounds in a color change from red/orange to brownish. The products after heating are homogeneous and show no signs of extensive decomposition.

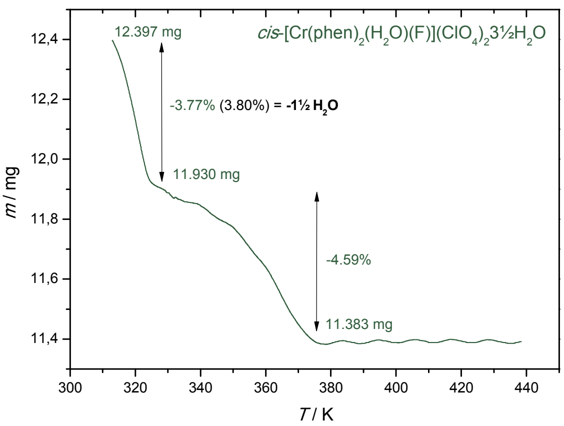

I: Thermolysis of *cis*-[Cr(phen)$_2$(H$_2$O)(F)](ClO$_4$)$_2$

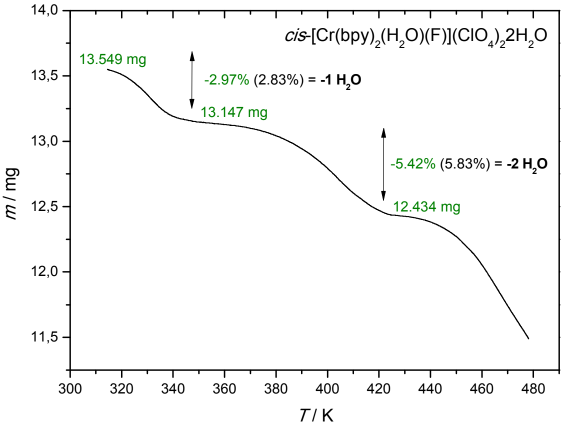

II: Thermolysis of *cis*-[Cr(bpy)$_2$(H$_2$O)(F)](ClO$_4$)$_2$

Figure 45 X-ray powder diffraction of thermolysis products

The treatment was additionally performed on a glass plate in order to identify if HF was evolved. Etching of the glass plate could not be observed and the reaction leading to hydroxo-bridged systems was therefore deemed less likely. In accordance with this, elemental analysis of reaction

products showed a complete dehydration response corresponding to a product stoichiometry of $[Cr(L'')_2(F)](ClO_4)_2$. This in combination with the structure of the reactants gives good reason to believe that the reaction products could be fluoride bridged dimers: $[(L'')_2Cr(\mu\text{-}F)_2Cr(L'')_2]^{4+}$.

To follow quantitatively, the release of water, *cis*-$[Cr(L'')_2(H_2O)(F)](ClO_4)_2{\cdot}nH_2O$ was subjected to a thermogravimetric analysis. Thermogravimetric responses of the two compounds are shown in Figure 45. As expected shows the X-ray powder diffractogram that the thermolyzed product has a different composition than the starting material. As found for other properties such as solubility and structure, also a difference in the behaviour of the phen and bpy complexes during thermolysis was observed: *cis*-$[Cr(phen)_2(H_2O)(F)](ClO_4)_2$ exhibits as compared with *cis*-$[Cr(bpy)_2(H_2O)(F)](ClO_4)_2$ a much greater sensitivity to humidity and lose crystal water quite easily.

The content of water released in several stages. Both complexes show the presence of crystal water released at different temperatures, reflecting the strength with which it is bound in the structure. The data for *cis*-$[Cr(bpy)_2(H_2O)(F)](ClO_4)_2{\cdot}2H_2O$ are the most readily interpretable. For this compound, water loss occurs in distinc stages reflecting the hydrogen bonding network between crystal water and anions identified by single crystal diffraction. Table 20 and Figure 43 show that the two crystal water in this complex is unevenly differently bound. This can also be demonstrated experimentally, by placing the complex at room temperature over concentrated sulphuric acid which leads to loss of one of the two crystal water molecules per formula unit. At temperatures of $\geq$100 °C the loss of the last crystal water and the coordinated water ligand occurs. The process is complete at approximately 150 °C. In the phen-complex, the first loss of water occurs already around room temperature and is complete at ca. 50 °C. This process corresponds approximately to a loss of 1½H_2O per formula unit. The data obtained for *cis*-$[Cr(phen)_2(H_2O)(F)](ClO_4)_2$ are not conclusive in the same way as for *cis*-$[Cr(bpy)_2(H_2O)(F)](ClO_4)_2$. However, it was found by elemental analysis, that the product obtained by thermolysis has a composition equivalent to a complete loss of both crystal and coordinated water.

Despite these promising results which lend some support to the idea of targeting fluoride bridges through thermally driven condensations, it has not so-far been possible to unambiguously characterize any fluoride bridged products by this approach.

EXPERIMENTAL DETAILS

Details for the synthesis of the following complexes are given in this section. An overview of the relationship between these compounds is shown in Scheme 4.

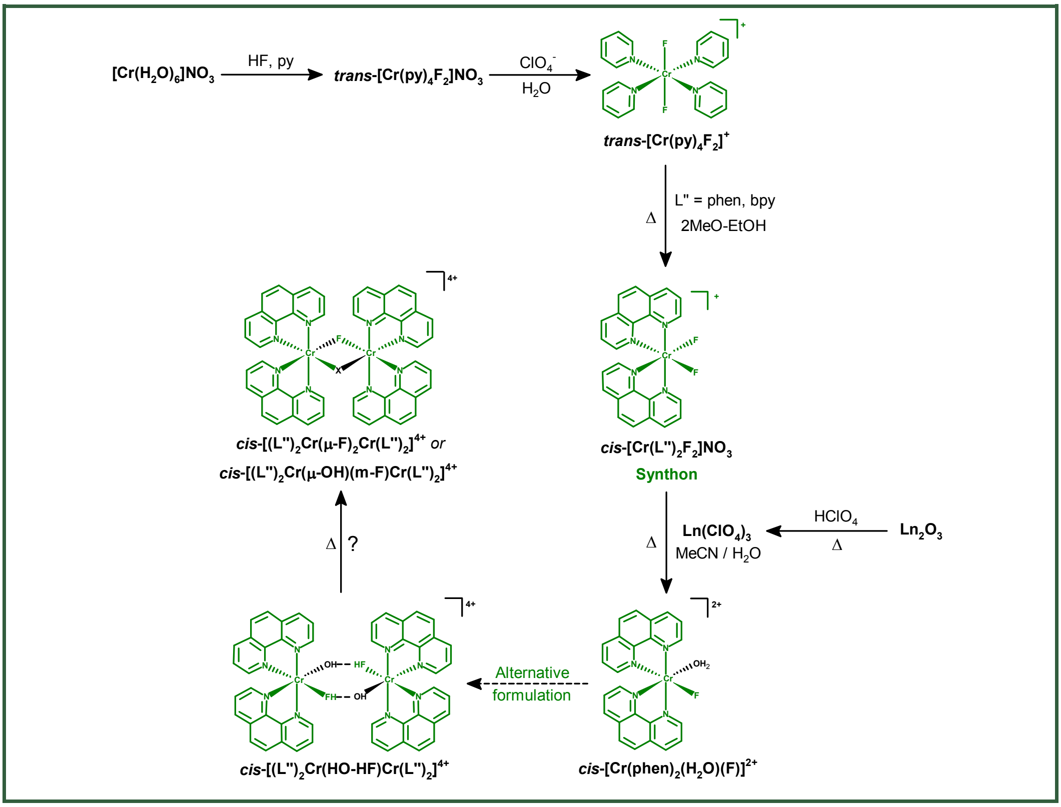

Scheme 4

- *cis*-$[Cr(phen)_2F_2]ClO_4{\cdot}H_2O$ (Single crystals, X-ray)
- *cis*-$[Cr(L'')_2(H_2O)(F)](ClO_4)_2$ for L" = phen, bpy. (Synthesis and single crystals, X-ray)
- *cis*-$[(L'')_2Cr(\mu\text{-}F)_2Cr(L'')_2](ClO_4)_4$ for L" = phen, bpy. (Synthesis)

General comments

The preparations of ligands and complexes were all performed under normal laboratory conditions and all chemicals and solvents were used direct as supplied without prior purification or drying. The supplier of a given chemical is specified where this first appeares in the

preparation. Also indicated is the purity as declared by the supplier. A description of the techniques, equipment and detailed use in characterization is given in Appendix 2 “Instrumentarium”. The starting material *cis*-$[Cr(L'')_2F_2]ClO_4$ for L"=phen, bpy was prepared acording to the method given by Glerup *et al.* [6]

> ***Safty remark:***
> *All compounds described are obtained with perchlorate as counterion, which imply some safety concerns. Perchloric acid and perchlorates are known to form potentionally explosives in connection with organic materials. Likewise, inorganic perchlorates can explode by thermal or mechanical impact. The sensitivity of the perchlorate salts obtained in the following has been tested by ignition in a gas flame and grinding a small amount of the compound in a mortar. Detonation has not been observed for any of them. But, it must be noted that even small quantities of* *cis*-$[Cr(L'')_2(F)(H_2O)](ClO_4)_2$ *burn with remarkable intensity. Note also, that Delavar, et al. refer to the 1,10-phenantroline complex as an explosive.* [347]

***In situ* generation of $Ln(ClO_4)_3 \cdot nH_2O$**

The lanthanoid perchlorate, $Ln(ClO_4)_3 \cdot nH_2O$ is not commercially available in solid state and is generated *in situ* by dissolving the corresponding oxide, Ln_2O_3 in a slight excess of perchloric acid.

$$Ln_2O_3 + 6\ HClO_4 \xrightarrow{\Delta} 2\ Ln(ClO_4)_3 + 3\ H_2O$$

This excess was approximetly 2 mol% and cannot independently form basis of the acid catalysed aquation reaction mentioned earlier. In principle, all lanthanoids, Ln(III) can be used in the aquation synthesis of *cis*-$[Cr(L'')_2(H_2O)(F)]^{2+}$. A representative selection, Pr, Nd, Dy, Ho and Er were tested and all found equally suitable for use in the synthesis. The lanthanoids forming oxides with mixed stoichiometriy *e.g.* the dark comercial Praeseodymium oxide, P_6O_{11} consting of Pr(III) and Pr(IV) were dissolved in a mixture of perchloric acid and hydrogenperoxide (35%) in order to reduce Pr(IV) to Pr(III). Below, the method is exemplified using the oxide of neodymium.

Synthesis of *cis*-$[Cr(L'')_2(F)(H_2O)](ClO_4)_2 \cdot nH_2O$ for L"=phen, bpy

$$3\ cis\text{-}[Cr(L'')_2F_2]ClO_4 + Ln(ClO_4)_3 + 3\ H_2O \xrightarrow[\Delta]{MeCN/H_2O} 3\ cis\text{-}[Cr(L'')_2(H_2O)(F)](ClO_4)_2 + LnF_3$$

***cis*-$[Cr(phen)_2(F)(H_2O)](ClO_4)_2 \cdot nH_2O$**

Nd_2O_3 (1.002 g, 2.98 mmol, 99 % Alfa Aesar) was placed in a 250 mL Erlenmeyer flask and added perchloric acid $HClO_4$ (2 mL, ~18.4 mmol, 60% Riedel-de Haën). The flask was placed on a water bath (~70°C) and gently shaken until the oxide dissolved. The resulting solution was light pink or colorless, depending on the light source. Water (80 mL) was added and the solution was quantitatively transferred to a 500 mL Erlenmeyer flask by use of MeCN (240 mL, HPLC Lab Scan). *cis*-$[Cr(phen)_2F_2]ClO_4$ (4.107 g, 7.47 mmol) was added and dissolved giving a violet-red solution. The solution was placed on a water bath with initial temperature of ~60°C and heated with stirring for at least 70 min. During this time, the temperature of the water bath was raised from the initial temperature to 90°C. The resulting unclear red solution (50 mL) with distinctive precipitation was placed at room temperature for 30 min.
The reddish mixture was extracted with a mixture of water and MeCN (60 mL in 1:2 ratio). The resulting unclear suspension was transferred to small centrifuge glass (~8) and centrifuged for 3 min. The intensely red supernatant was carefully removed from the white precipitate and left for evaporation in a stream of N_2 giving a red crystalline product. During evaporation a film of crystals is formed on the solution, this was broken repeatedly. Yield: 5.667 g.
The raw product was dissolved in mixture of water and MeCN (60 mL in 1:2 ratio) and is centrifuged as previously described. Giving a further small residue of white precipitate. The red solution was transferred to a crystallization dish, added water (500 mL) and left for crystallization (~3-4 days). The red crystal flakes were removed from the sides of the dish, suspended with the slightly colored supernatant and isolated by vacuum filtration, washed with water (2·20 mL) and dried in a dynamic vacuum for 2½ hours. The red, flaky, crystalline product was transferred to a desiccator over water. The crystal water content will be approximately $2H_2O$ to $3\frac{1}{2}H_2O$.

Yield: 4.309 g (*cis*-$[Cr(phen)_2(F)(H_2O)](ClO_4)_2 \cdot 3\frac{1}{2}H_2O$) (81.1% of theoretical based on Cr(III))

- Elemental analysis: Calcd. (found) (%) $H_{50}C_{48}N_8O_{25}F_2Cl_4Cr_2$: H 3.54(2.70), C 40.52(40.46), N 7.88(7.84).

Only slightly soluble in water, insoluble in MeOH, EtOH and CH_2Cl_2. The compound is soluble in $MeNO_2$. The solubility in MeCN depends on the number of crystal water.
If the compound is left to dry in high humidity air the crystal water content will be ~ $2H_2O$:

- Elemental analysis: Calcd. (found) (%) for $H_{22}C_{24}N_4O_{11}F_1Cl_2Cr_1$: H 3.24(2.73), C 42.12(42.07), N 8.19(8.02).

***cis*-[Cr(bpy)$_2$(F)(H$_2$O)](ClO$_4$)$_2$**

Nd_2O_3 (0.250 g, 0.743 mmol, 99 % Alfa Aesar) was placed in a 250 mL Erlenmeyer flask and added perchloric acid $HClO_4$ (0.5 mL, ~ 4.6 mmol, 60% Riedel-de Haën). The flask was placed on a water bath (~70°C) and gently shaken until the oxide dissolved. The resulting solution was light pink or colourless, depending on the light source.

cis-[Cr(bpy)$_2$F$_2$]ClO$_4$ (1.011 g, 2.015 mmol) was dissolved in a mixture of MeCN/water (60 mL/20 mL, HPLC Lab Scan) giving a red to violet solution and placed on a water bath with an initial temperature of ~70°C. With stirring, this solution was transferred to the Nd(III)-solution resulting in a color change to red. The solution was heated with stirring for 35 min. During this time, the temperature of the water bath was raised from 70°C to the boiling point. The resulting unclear, orange solution was placed at room temperature whereupon it solidifies orange crystalline mass. This product is extracted with a mixture of water and MeCN (30 mL in 1:2 ratio). The resulting unclear orange solution was transferred to small centrifuge glass and centrifuged. The red supernatant was carefully removed from the white precipitate and left for evaporation in a stream of nitrogen giving an orange, crystalline product. The product was isolated by vaccum filtration, washed with ice water (2·10 mL) and dried by suction in air for 45 min. The raw product was redissolved in a mixture of water and MeCN (30 mL in 1:2 ratio) and treated as described above. The product was left in air with high humidity to constant weight (*e.g.* at summer) the crystal water content will be approximately 2H_2O.

Yield: 1.073 g (83.7% of theoretical based on chromium)

- Elemental analysis: Calcd. (found) (%) for $H_{22}C_{20}N_4O_{11}F_1Cl_2Cr_1$: H 3.48(3.41), C 37.75(37.97), N 8.80(8.70).

cis-[Cr(bpy)$_2$(F)(H$_2$O)](ClO$_4$)$_2$·2H_2O is only slightly soluble in water and insoluble in MeOH, EtOH, CH_2Cl_2 and MeCN.

Comment on the water content of the compounds

The content of crystal water in case of both complexes, *cis*-[Cr(L")$_2$(H$_2$O)(F)](ClO$_4$)$_2$ is sensitive to reaction conditions and humidity during storage. This makes it difficult to obtain an easily interpretable elemental analysis, even for "pure" products. Taken directly from the reaction mixture, the crystal water content is 2H_2O and 3½H_2O for the two complexes.

This water content remains approximately constant by drying in moist air. Drying in air with low humidity, results in a variable water content of approximately n=1.4 to 2 for the bpy complex and n=¾ to 3½ for the phen complex. The variation in water content is studied by placing the two compounds in a desiccator over water, and then concentrated sulphuric acid, in both cases for one week. Elemental analyses for each of these experiments are as follows:

cis-[Cr(L")$_2$(F)(H_2O)](ClO_4)$_2$· nH_2O L"	Humidity		H%	C%	N%	Interpretation **n**H_2O
**bpy**	0%	Obtained	3.30	39.04	8.97	**1**
		Cal.	3.26	38.85	9.06	
	100%	Obtained	3.23	37.82	8.71	**2**
		Cal.	3.48	37.75	8.80	
**phen**	0%	Obtained	2.89	45.38	8.68	–
		Cal.	–	–	–	
	100%	Obtained	2.70%	40.46	7.84	**3½**
		Cal.	3.54	40.52	7.88	

Crystals for X-ray diffraction of aquation *cis*-di-fluorido Cr(III) complexes

***cis*-[Cr(phen)$_2$F$_2$]ClO$_4$·H$_2$O**

Crystal suitable for single crystal diffraction were obtained by the following method:
0.208 g *cis*-[Cr(phen)$_2$F$_2$]ClO$_4$·H$_2$O was dissolved in a solution of water/acetonitrile (20 mL/10 mL) and filtered though a filter paper into a smal backer. The backer was covered with a lid of paper and left undisturbed ar room temperature for crystallization (ca. 3-5 days). The crystals was harvested by gently scratching with a spatula and washed with the mother liquid.

***cis*-[Cr(bpy)(F)(H$_2$O)](ClO$_4$)$_2$·2H$_2$O**

Crystals of *cis*-[Cr(bpy)(F)(H$_2$O)](ClO$_4$)$_2$·2H$_2$O sutabel for single crystal diffraction were obtained directly form slow evapoation of supernantant obtained after removing LnF$_3$ as mentioned in the synthesis describtion.

Synthesis $[(L'')_2Cr(\mu\text{-}F)_2Cr(L'')_2](ClO_4)_4$ for L"=phen, bpy

$$2\ cis\text{-}[Cr(L'')_2(H_2O)(F)](ClO_4)_2 \cdot nH_2O \xrightarrow{\Delta} [(L'')_2Cr(\mu\text{-}F)_2Cr(L'')_2](ClO_4)_4 + 2(1+n)\ H_2O$$

$[(phen)_2Cr(\mu\text{-}F)_2Cr(phen)_2](ClO_4)_4$

Solid red *cis*-$[Cr(phen)(F)(H_2O)](ClO_4)_2{\cdot}3\frac{1}{2}H_2O$ (0.438 g, 0.616 mmol) was crushed thoroughly in a porcelain mortar to a fine and homogeneous powder, spread in a thin layer on a platinum plate and placed in an oven at 140 °C for 4 days. After heating the greyish brown product it was cooled in the oven to room temperature.

Yield: 0.379 g (mass loss: 0,059 g)

- Elemental analysis: Calcd. (found) (%) for $H_{32}C_{48}N_8O_{16}F_2Cl_4Cr_2$: H 2.56(2.47), C 45.73(45.92), N 8.89(8.86).

$[(bpy)_2Cr(\mu\text{-}F)_2Cr(bpy)_2](ClO_4)_4$

Solid *cis*-$[Cr(bpy)(F)(H_2O)](ClO_4)_2{\cdot}2H_2O$ (0.18 g, 0.37 mmol) was crushed thoroughly in a porcelain mortar to a fine and homogeneous powder, spread in a thin layer on a platinum plate and positioned in an oven at 140-150 °C for 4 days. After heating, the product was cooled in the oven to room temperature.

Yield: 0.15 g (mass loss: 0.03 g)

- Elemental analysis: Calcd. (found) (%) for $H_{32}C_{40}N_8O_{16}F_2Cl_4Cr_2$: H 2.77(2.74), C 41.25(42.73), N: 9.62(9.91).

CHAPTER 7

METHOXIDO-BRIDGED Ln(III)-Cr(III) SYSTEMS OBTAINED BY FLUORIDE ABSTRACTION

INTRODUCTION

In the previous chapter it was shown that (partial) solvolysis of the otherwise quite robust Cr(III) fluorido complexes could occur when these are allowed to react with lanthanoid ions under slightly forcing conditions. Serendipitously, it was found that this type of reactivity is more general, than could have been anticipated. Thus reactions of the same chromium(III) fluorido complexes studied in the previous chapter with lanthanoid ions in methanolic solution was found to yield heterobimetallic 1:1 complexes with the stochiometry $CrLn(phen)_2(CH_3O)_2(CH_3OH)_2(NO_3)_4$. The reaction conditions required were not very harsh, as the reactions were carried out at ambient temperature, but with long reaction times. The resulting solvolysis products are well crystallizing and even very large single crystals with volumes of several mm^3 could be obtained directly from the reaction mixtures. The molecular structure of the product was determined from single crystal diffraction and was found to be a dinuclear methoxido–bridged dimer. Thus, the reaction can be summerized by the balanced equation below:

$$\begin{aligned} &3\,[Cr(phen)_2F_2]NO_3 \;+\; 5\,Ln(NO_3)_3 \;+\; 6\,MeOH \\ &\xrightarrow{MeOH} 3\,[(phen)_2Cr(\mu\text{-}MeO)_2Ln(NO_3)_4]\cdot 2MeOH \;+\; 2\,LnF_3 \;+\; 6\,HNO_3 \end{aligned} \quad (6.17)$$

The reaction has been successfully carried out for Ln=Nd, Sm, Gd, Tb, and Dy (the latter two by co-workers) and the products have formed basis for paper 8, which focuses on the magnetic properties of this type of compounds.

STRUCTURAL DISCUSSION

The compounds are isostructural for the examined lanthanoid ions and crystallize in the orthorhombic space group *Pbcn* with four formula units in the unit cell and all molecules lying on special positions, two-fold rotation axes. The molecular structure of the product is shown in Figure 46 below and selected bond lengths have been collected in Table 22.

The coordination geometry around the chromium centre is the expected distorted octahedron with bond angles of 91.97(6)–96.10(6) ° between the methoxido bridges and the nitrogen donors and the expected small internal bite angle of phen of 79.93(6) °.

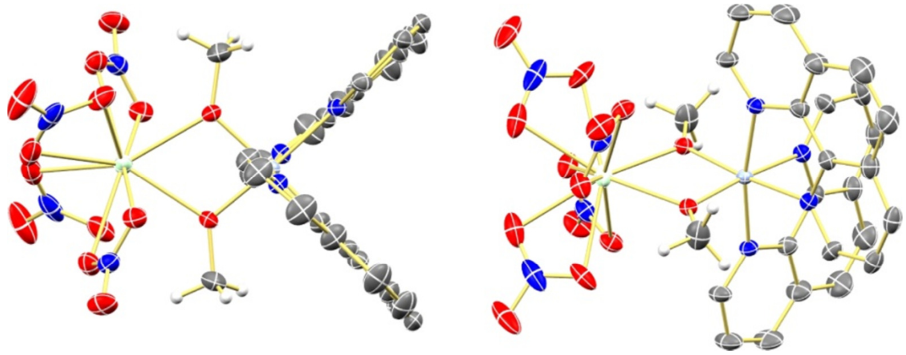

Figure 46 Molecular structure of the dinuclear complex in [(phen)$_2$Cr(μ-MeO)$_2$Nd(NO$_3$)$_4$]·2MeOH.

Top and side views. Hydrogens on the phen ligands and solvate molecules have been omitted for clarity.

Bond length (Å)								
**Nd**	Nd_1–O_{41}	2.3992 (15)	**Tb**	Tb_1–O_1	2.334 (2)	**Dy**	Dy_1–O_1	2.324 (6)
	Nd_1–O_{21}	2.5433 (17)		Tb_1–O_{31}	2.471 (2)		Dy_1–O_{42}	2.457 (8)
	Nd_1–O_{33}	2.555 (4)		Tb_1–O_{42}	2.45 (3)		Dy_1–O_{31}	2.465 (7)
	Nd_1–O_{31}	2.573 (4)		Tb_1–O_{41}	2.527 (3)		Dy_1–O_{41}	2.517 (7)
	Nd_1–O_{23}	2.5906 (16)		Tb_1–O_{32}	2.537 (2)		Dy_1–O_{32}	2.528 (6)
	Nd_1–O_{31}	2.593 (6)						
	Cr_1–O_{41}	1.9177 (14)		Cr_1–O_1	1.915 (2)		Cr_1–O_1	1.921 (6)
	Cr_1–N_1	2.0601 (16)		Cr_1–N_2	2.056 (2)		Cr_1–N_2	2.064 (7)
	Cr_1–N_{12}	2.0832 (17)		Cr_1–N_1	2.084 (2)		Cr_1–N_1	2.076 (7)

Table 22 Selected bond lengths for [(phen)$_2$Cr(μ-MeO)$_2$Ln(NO$_3$)$_4$]·2MeOH

Importantly, the O_{41}–Cr–O_{41}* angle is at 82.40(9) ° significantly below the 90 ° of the regular octahedron, which would be expected to lead to diminished electronic interaction relative to the idealized octahedral geometry due to less efficient overlap between chromium and methoxido orbitals. Cr–N bond lengths fall in the range: 2.0601(16)–2.0832(17) Å which is in line with what is usually observed in Cr(III)-phen complexes. The longest Cr–N bonds are those approximately *trans* relative to the strongly donating methoxido ligands. The coordination number of the lanthanoid ions is 10 and the symmetry fairly high with the two-fold axis bisecting the methoxido-bridges and passing through the lanthanoid centre. All of the nitrate ions coordinate in a bidentate fashion to the lanthanoid. One of these nitrate ligands is disordered over two slightly different positions for all for compounds. Bond lengths between Nd and the bidentate nitrate ligands fall in the range: 2.5433(17)–2.593(6) Å and are thus significantly longer than the bond length between neodymium and the bridging methoxido ligand at 2.3992(15) Å.
The packing of the molecules in the unit cell is depicted in Figure 47, from which a structure with channels containing weakly bound and not particularly well refined methanol solvate molecules. The weak binding of the solvate molecules is evident not only from the structure, but also from the propensity of the crystals to effloresce and the varying solvent content found in elemental analyses (*cf.* experimental details section). The structure features no important intermolecular interactions, neither between solvate molecules nor between individual dinuclear complexes.

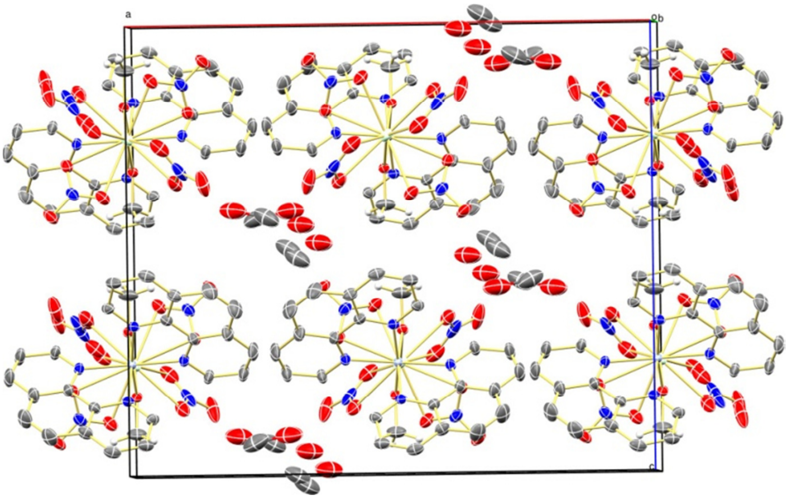

Figure 47 Unit cell contents of [(phen)$_2$Cr(μ_2-MeO)$_2$Nd(NO_3)$_4$]·2MeOH

Viewed approximately along the crystallographic *b*-axis. Hydrogens on the phen ligands and solvate molecules have been omitted for clarity. Channels holding the – partially disordered – methanol solvate molecules run parallel to the viewing direction.

The structural motif represented by $[(phen)_2Cr(\mu_2\text{-}MeO)_2Nd(NO_3)_4]\cdot 2MeOH$ is novel for 3*d*-4*f* systems hence direct comparison with other structures is not possible. However, the di-μ_2-methoxy bridging motif is a fairly common bridging arrangement for transition metals on their own with more than 350 reported structures of dinuclear species. These range in oxidation state from 0 in $(Et_4N)_2[(CO)_4W((\mu_2\text{-}MeO)_2W(CO)_4]$, [401] to +6 in $[(acac)Mo(O)_2(\mu_2\text{-}MeO)_2Mo(O)_2(acac)]$, [402] and for paramagnetic systems from Group 4. represented by $(cp)_2Ti(\mu_2\text{-}MeO)_2Ti(cp)_2$, [403] to Group 11. represented $[(py)_2Cu(\mu_2\text{-}MeO)_2Cu(py)_2](ClO_4)_2$, [404]. The system resembling most closely the present in terms of auxiliary ligand sphere around the transition metal is $[(bpy)_2Ru(\mu_2\text{-}MeO)_2Ru(bpy)_2]^{2+}$, [405]. Also approximately, a handful of dinuclear Cr(III) complexes with this bridging motif has been reported. The simplest of these is $[(acac)_2Cr(\mu_2\text{-}MeO)_2Cr(acac)_2]$, [406] In that system Cr–OCH_3 bond lengths range from 1.950 Å to 1.973 Å and are thus significantly longer that those found in the present systems. Simultaneously, the angles between the two methoxy groups are 78.58 ° and 79.34 ° measured from each of the two chromium centers. The methyl groups of the bridging methoxy ligands form an angle of ca. 30 ° with the central Cr_2O_2 plane. This is important since the magnetic exchange has been shown to depend strongly on this parameter. [407] For the dinuclear chromium system, an value for the exchange coupling constant of J=9.8 cm^{-1} was determined. This value is at the lower end of the range of values determined (0–43 cm^{-1}) for related hydroxo-bridged systems, [407] but direct comparisons are hampered by the absence of systems with identical ligand spheres and bridging geometries.

Although without precedence among mixed 3*d*-4*f* systems, the structure of the $[(phen)_2Cr(\mu_2\text{-}MeO)_2Ln(NO_3)_4]\cdot 2MeOH$ systems can be considered to resemble the classical "di-ol" or di-μ-hydroxido structures, which were introduced in the comparison with $[(acac)_2Cr(\mu_2\text{-}MeO)_2Cr(acac)_2]$ complex above. The "di-ols" are richly represented among the transition metals. In particular for Cr(III) and Co(III). Much of the interest in the chromium systems has been fuelled by studies of their magnetic properties serving often as reference systems for discussions of magnetic exchange phenomena. The resemblance to these classical systems prompted an examination of the magnetic exchange in the dinuclear methoxy-bridged chromium-lanthanoid complexes made during this project.

MAGNETIC PROPERTIES

As discussed in Chapter 3, the most common probe into the magnetic properties of coordination compounds is measurement of magnetic susceptibilities. However, in order to parametrize the susceptibility data and translate these into exchange coupling constants, it is required, that a reasonable Spin-Hamiltonian is constructed, which accounts for all of the states of relevance to the magnetic properties of the system. For transition metal complexes of relatively low

nuclearities this is usually a quite simple task. However, for the lanthaniod ions this does hold true. For the 4*f* systems, even mono-nuclear complexes can pose problems due to the unquenched angular momenta of the lanthanoid ions and resulting complicated ligand field splittings. Consequently, parametrization of magnetic data for 4*f* systems is often difficult and sensitive to the model employed. Exempt from these problems is of course Gd(III), which has a spin-only ground state $^8S_{7/2}$ isolated by several 10.000 cm^{-1} from the closest excited states. This ion can therefore be treated well by use of a Spin-Hamiltonian taking into account, locally, only the eight spin components of the ground state. However, the overall cluster of poly nuclear systems can be augmented by techniques such as XMCD, which probe the system in question at the various X-ray absorption edges, and thereby allow for element-specific measurement of magnetizations. In the dinuclear Cr–Ln systems it is thus possible to measure the magnetization of the two metal centers individually. This has proven a very useful technique, [408] and it was also applied to $[(phen)_2Cr(\mu_2\text{-}MeO)_2Dy(NO_3)_4]\cdot 2MeOH$ in conjunction with normal susceptibility measurements. The results are summarized in Figure 48, which is taken directly from paper 8, data by Dr. Dreiser. The slightly disappointing result is that the magnetic exchange between Cr(III) and Dy(III) in the methoxido-bridged system is negligble (J=0.04(3) cm^{-1}) to the extent that is is difficult to quantify even when using XMCD.

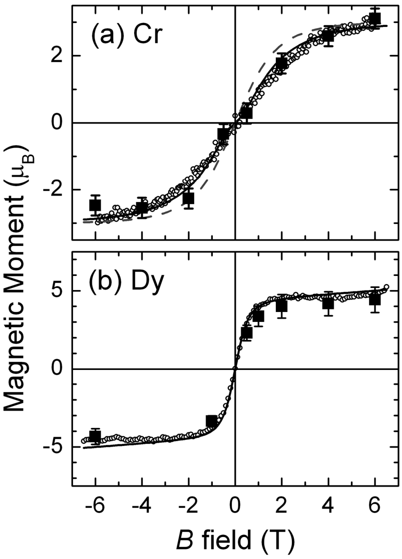

Figure 48 Magnetization of Cr and Dy in $[(phen)_2Cr(\mu\text{-}MeO)_2Dy(NO_3)_4]$

Measured independently by use of XMCD.

PERSPECTIVES

The reaction type described in this chapter is quite intriguing, since it obviously holds potential for extension of its scope. This applies not only to other alcoholate-derived ligands, but generally to systems with bridging ligands of lower affinity than fluoride for the lanthanoid ions. The potential importance lies in the use of the easily accessible fluorido complexes as precursors for other chromium containing metalloligands, with predetermined geometries and auxiliary ligands, which do not need to be isolated or even possible to isolate. By this approach it should be possible to directly target a wide range of hetero-metallic Cr(III)–Ln(III) systems or even Co(III)–Ln(III) systems, but time has not allowed for a more detailed investigation of these possibilities within the framework of this thesis work.

EXPERIMENTAL DETAILS

This section provides specifications for the synthetic preparation as well as the characterization of the methoxido bridged complex, *cis*-[(phen)$_2$Cr(μ-MeO)$_2$Ln(NO$_3$)$_4$] discussed in this chapter. A graphical representation of the reaction is given in Scheme 5.

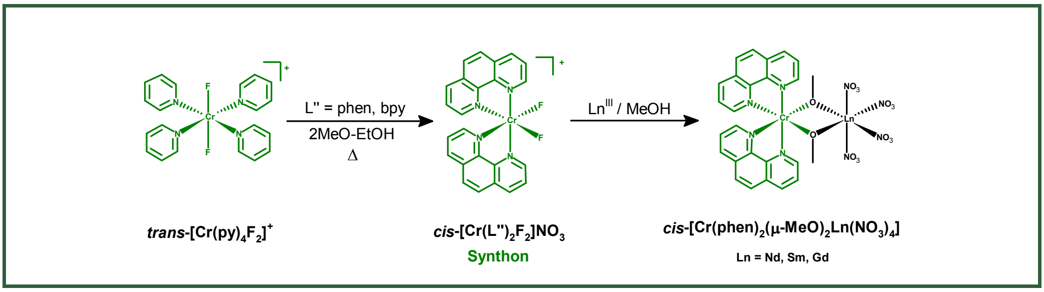

Scheme 5

- *cis*- [(L")$_2$Cr(μ-MeO)$_2$Ln(NO$_3$)$_4$] for Ln=Nd, Sm, Gd

General comments

All complexes were prepared at room temperature by slow mixing through diffusion together of methanolic solutions of the two reactants, *cis*-[Cr(phen)$_2$F$_2$]NO$_3$ and Ln(NO$_3$)$_3$·nH$_2$O.
The syntheses were carried out in custom-made diffusion cells consisting of three consecutive chambers separated by two porous glass frits (No. 4). Each chamber has a volume of approx. 20 mL and can be sealed. It is of importance that the employed solutions are sufficiently dilute to minimize problems with precipitation of insoluble by-products during the course of reaction.
In addition to the 1,10-phenantroline complexes discussed here also the corresponding 2,2′-bipyridine complexes have been investigated for this type of reactivity. In all cases formation of a white powder of LnF$_3$ has been observed, but no crystalline products were obtained from the bipyridine systems. Characterization of the obtained product has proven somewhat problematic because of an immediate degradation of the crystal surface when exposed to air. To confirm that the reaction is roughly independent of reaction time and other parameters, with regard to the nature of the product, two independent single crystal X-ray diffraction experiments have been carried out on different preparations.

Synthesis of *cis*-[Cr(phen)$_4$F$_2$]NO$_3$·nH$_2$O

$$trans\text{-}[Cr(py)_4F_2]NO_3 + 2\text{ phen} \xrightarrow[\Delta]{\text{2-MeO-EtOH}} cis\text{-}[Cr(phen)_2F_2]NO_3 + 4\text{ py}$$

trans-[Cr(py)$_4$F$_2$]NO$_3$ (36.7 g; 0.078 mol) and 1,10-phenantroline (34.8 g; 0.19 mol, Alfa Aesar) is placed in an Erlenmeyer flask (500 mL) and added 2-methoxyethanol (250 mL, Alfa Aesar, 99.3+ %). The flask is equipped with an Ahlin-condenser. With stirring and reflux is the mixture heated to the boiling point, whereby a violet solution is formed. Shortly after precipitation of a red-violet solid are observed. The heating is continued for 1 hour. The flask is cooled to room temperature before the red-violet product is isolated on a sintered glass filter funnel (No. 3). The raw product is washed with ethanol (2·100 mL, De Danske Spritfabrikker, 99 %) and dried by suction.

Yield of raw product: 31.7 g (79.0% of theoretical based on *cis*-[Cr(phen)$_2$F$_2$]NO$_3$).

- Elemental analysis: Calcd. (found) (%) for $H_{17}C_{24}N_5O_4F_2Cr_1$ (·½H_2O): H 3.29(3.16); C 55.28(55.25); N 13.43(13.30).
- TOF MS ES$^+$ (MeOH): m/z: 450.5 ([Cr(phen)$_2$F$_2$]$^+$)

Synthesis of *cis*-[Cr(bpy)$_4$F$_2$]NO$_3$

$$trans-[CrF_2(py)_4]^+ + 2\text{ bpy} \xrightarrow[\Delta]{\text{2MeO-EtOH}} cis-[CrF_2(bpy)_2]^+ + 4\text{ py}$$

trans-[Cr(py)$_4$F$_2$]NO$_3$ (21.1 g; 0.045 mol) and 2,2'-bipyridine (15.6 g; 0.100 mol) is placed in a conical flask (500 mL) and add 2-methoxyethanol (100 mL). With stirring and reflux the violet mixture is heated to boiling temperature, whereby reddish solution is formed. The heating is continued for 1.5 hour, during this time the solution becomes more turbid of a precipitated solid. The flask is placed on ice for 30 min. before a brownish red product is isolated on a a sintered glass filter funnel (No. 3). The raw product is washed with ethanol (2·50 mL) and dried by suction.

Yield of raw product: 11.1 g (53.1% of theoretical based on chromium)

Recrystallisation:
The raw product of *cis*-[Cr(bpy)$_2$F$_2$]NO$_3$ is recrystallized from boiling methanol (0.083 g·mL^{-1}). Slow evaporation during approx. 2 days of the subsequent filtered solution lads to the formation of violet red crystalline product.

Yield: 7.2 g (34.5% of theoretical based on chromium). A fraction more can be gained by further evaporation of the mother liquor.

Synthesis of $[(phen)_2Cr(\mu\text{-}MeO)_2Ln(NO_3)_4]$ for Ln=Nd, Sm, Gd

$$3\ [Cr(phen)_2F_2]NO_3\ +\ 5\ Ln(NO_3)_3\ +\ 6\ MeOH\ \xrightarrow{MeOH}\ 3\ [(phen)_2Cr(\mu\text{-}MeO)_2Ln(NO_3)_4]\ +\ 2\ LnF_3\ +\ 6\ HNO_3$$

In the two outer chambers solutions of *cis*-$[Cr(phen)_2F_2]NO_3$ (0.6 mmol) in MeOH (20 mL, Lab-Scan; Anhydroscan) and $Ln(NO_3)_3{\cdot}nH_2O$ (0.6 mmol, $Nd(NO_3)_3{\cdot}6H_2O$, $Sm(NO_3)_2{\cdot}6H_2O$, $Gd(NO_3)_2{\cdot}H_2O$, 99.9%, Alfa Aesar) in MeOH (20 mL) was placed respectively, while the middle chamber was filled with methanol. The diffusion cell was sealed and left undisturbed until no visible changes in the cell was observed over a period of 3 months. (total time of synthesis: 6-12 months). During this time of crystallization, large red crystals together with a fine white powder were formed in all three chambers. In the chamber where *cis*-$[Cr(phen)_2F_2]NO_3$ was originally placed, the white byproduct dominated considerably and the content of this chamber was discarded. The red crystals from the remaining chambers were harvested by gentle scratching with a spatula and repeatedly washed by decantation with methanol.

$[(phen)_2Cr(\mu\text{-}MeO)_2Nd(NO_3)_4]$:
Yield: 0.141 g (46.1 % of theoretical based on Nd(III)).

- Elemental analysis: Calcd. (found) (%) for $H_{22}C_{26}N_8O_{14}Cr_1Nd_1$(dried): H 2.30(2.33), C 35.58(35.16), N 12.51(12.39).

$[(phen)_2Cr(\mu\text{-}MeO)_2Sm(NO_3)_4]$:

- Elemental analysis: Calcd. (found) (%) for $H_{22}C_{26}N_8O_{14}Cr_1Sm_1$(dried): H 2.54(2.25), C 35.78(34.10), N 12.84(12.01).

$[(phen)_2Cr(\mu\text{-}MeO)_2Gd(NO_3)_4]$:

- Elemental analysis: Calcd. (found) (%) for $H_{22}C_{26}N_8O_{14}Cr_1Gd_1$(dried): H 2.52(2.50); C 35.50(35.39), N 12,74(13.30)

CHAPTER 8

FLUORIDE BRIDGING AS A STRUCTURE-DIRECTING MOTIF IN 3*d*-4*f* COMPLEXES

INTRODUCTION

In the two preceding chapters, solvolysis of Cr(III)-fluorido complexes has been discussed, and it has been demonstrated to be a viable and multi-facetted synthetic method. However, it should be admitted, that the goal, which produced these useful scientific by-products was the rational synthesis of fluoride-bridged chromium-lanthanoid complexes by analogy with the results obtained with the hard alkali metal ions (Chapter 5). This was achieved by relatively minor modifications of the procedures used for the solvolytic reactions. Again, the Cr(III) systems of choice, *cis*-$[Cr(L'')_2F_2]^+$ (L''=phen, bpy) were used.

Upon mixing of solutions of *cis*-$[Cr(L'')_2F_2]^+$ and lanthanoid ions, a clearly discernible color change from pink towards orange takes place. Spectroscopically, the process can be shown to be a simple conversion of the Cr(III) starting material to one product with clear isosbestic points as shown in the Figure 49. The direction of the spectral change may be considered counterintuitive, since perturbation of the fluoride ligands by a Lewis acid would be expected to lead to an overall smaller ligand field at the Cr(III) center. However, the same effect was observed when the alkali metal ions were used (Chapter 5) and it has been rationalized in Paper 4 as a breakdown of the transferability of ligand field parameters between the unperturbed system and the ones with Lewis acids coordinating to the chromium bound fluorido ligands.

The association of the Ln(III) ions and the Cr(III) complexes could also be demonstrated by use of electro spray mass spectrometry. This soft inonization technique gave signals from a range of polynuclear complexes which all could be related to structure determined for the crystalline product (*cf.* supporting material to Paper 7).

In constrast to the methoxido-bridged systems discussed in the previous chapter, the structures of the crystalline products obtained with fluorido bridges were found to be tetra-nuclear. These systems form the basis for Paper 6, and together with related systems derived from the same synthetic strategy they are investigated in more detail in Paper 7. While the difference in product structure compared to the methoxido systems at a first glance might seem surprising, it is, in hindsight quite, reasonable and bordering on being predictable. The reason for the repeated emphasis throughout this thesis, on fluoride's preference for forming linear bridges, is the understanding gained by comparing structures of different polynuclear Cr(III)-4*f* systems obtaing with different ligand spheres on chromium, different solvents, and different co-ligands in the lanthanoid centers. Invariably, we have found structures, which are compatible with the assumption of a quite pronounced energetic preference of fluoride for avoiding forming bent bridges. Thus, although more than a dozen of different structures have been obtained using the synthetic strategy described here, chelation has not yet been observed, neither for di- nor tri-fluorido complexes.

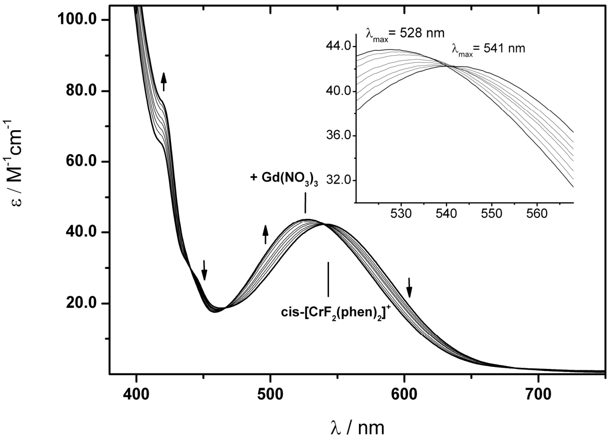

Figure 49 Titration of *cis*-[Cr(phen)$_2$F$_2$]$^+$ with Gd(III)

In MeOH/MeCN solution.

STRUCTURAL DISCUSSION

The molecular structure of the tetra-nuclear complex *cyclo*-[(μ-F)(phen)$_2$Cr(μ-F)Gd(NO$_3$)$_4$] is shown in Figure 50 and selected geometric data for the neodymium and the gadolinium complexes have been listed in Table 23. The two compounds are seen to have nearly identical structures, the slightly (ca. 2 %) smaller size of the Gd(III) has practically no consequences. The near linearity of the fluoride bridge has as a derived consequence that the ∠F–Cr–F angle, at 91.40 °, becomes significantly larger than the ∠MeO–Cr–OMe angle in the chelating methoxido complex discussed in Chapter 7.

As mentioned in that chapter, magnetic exchange would be expected to diminish with increasing deviation from the octahedral 90 ° angle at Cr(III). It could thus be expected that magnetic exchange in the tetranuclear systems would be larger that in the chelating di-nuclear ones.

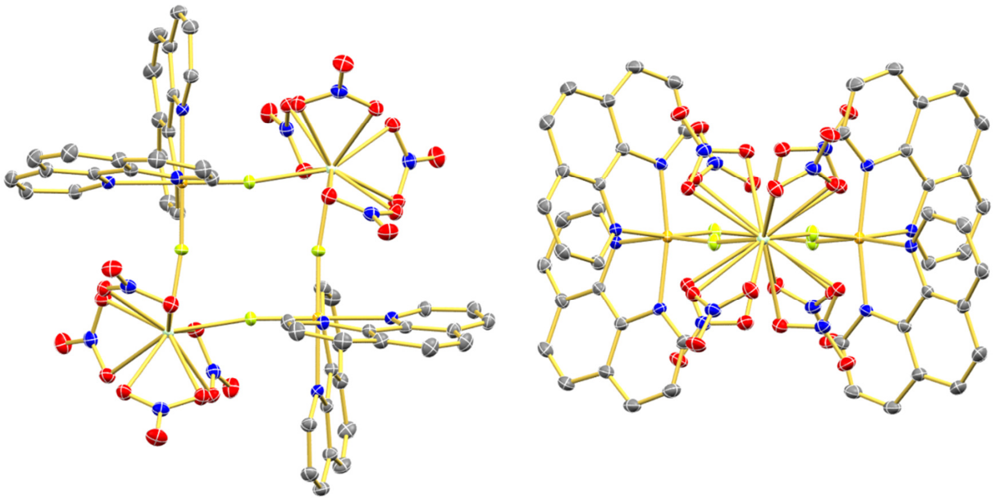

Figure 50 Molecular structure of *cyclo*-[(μ-F)(phen)$_2$Cr(μ-F)Gd(NO$_3$)$_4$]

Hydrogens on the phen ligands have been omitted for clarity

Bond length (Å)			Bond angle (°)		
Nd_1–F_1	2.3348(14)	Cr_1–N_1	2.0615(19)	F_1–Cr_1–F_1	91.40(9)
Nd_1–O_1	2.5574(19)	Gd_1–F_1	2.2844(16)	F_1–Cr_1–F_1	168.74(8)
Nd_1–O_2	2.5651(18)	Gd_1–O_1	2.506(2)	Cr_1–F_1–Nd_1	91.41(10)
Nd_1–O_4	2.5326(19)	Gd_1–O_2	2.521(2)	Cr_1–F_1–Gd_1	168.61(9)
Nd_1–O_5	2.5648(19)	Gd_1–O_4	2.488(2)		
Cr_1–F_1	1.8816(14)	Gd_1–O_5	2.533(2)		
Cr_1–N_2	2.0550(17)				

Table 23 Selected bond lengths and angles for *cyclo*-[(μ-F)(phen)$_2$Cr(μ-F)Ln(NO$_3$)$_4$]

This is indeed the case. For *cyclo*-[(μ-F)(phen)$_2$Cr(μ-F)Gd(NO$_3$)$_4$] a value for $J_{Gd\text{-}Cr}$ of 0.71 cm^{-1} was determined. Although the Ln–F bonds are quite short, this bond formation has only a quite small effect on the Cr–F bonds, which are elongated from 1.8444 (10), and 1.8621(10) Å in the parent complex (*cf.* Paper 2) to just above 1.88 Å in the tetranuclear systems.

When it was attempted to vary the lanthanoid ion across the complete range of 4*f*-elements, it was found that the yield depended strongly on the position of the lanthanoid ion. For the early elements, good yield of up to 87 % were obtained, but with increasing atomic number, the yield diminished and it was only possible to isolate products up til Ho. The diminishing yields were

accompanied with increased formation of slightly colored by-products precipitating as very fine powders. The by-products are carbon and nitrogen containing, and thus not simple LnF_3. In the cases where crystalline products could be obtained, their isomorphism to the structurally characterized members was verified by powder X-ray diffraction *cf.* Figure 51.

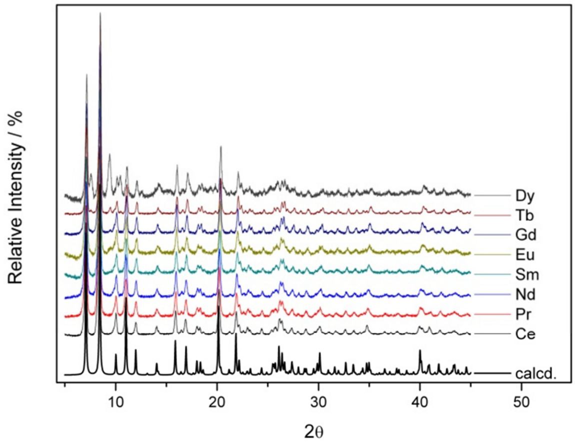

Figure 51 Powder X-ray diffractograms of the isomorphous series of *cyclo*-[(μ-F)(phen)$_2$Cr(μ-F)Ln(NO$_3$)$_4$] for Ln=Ce to Dy

EXPERIMENTAL DETAILS

This section provides specifications for the synthetic preparation as well as the characterization of the tetra-nuclear complexes *cyclo*-[(μ-F)(L")$_2$Cr(μ-F)Ln(NO$_3$)$_4$] for L"=phen, bpy discussed in this chapter. A graphical representation of the reaction is given in Scheme 6.

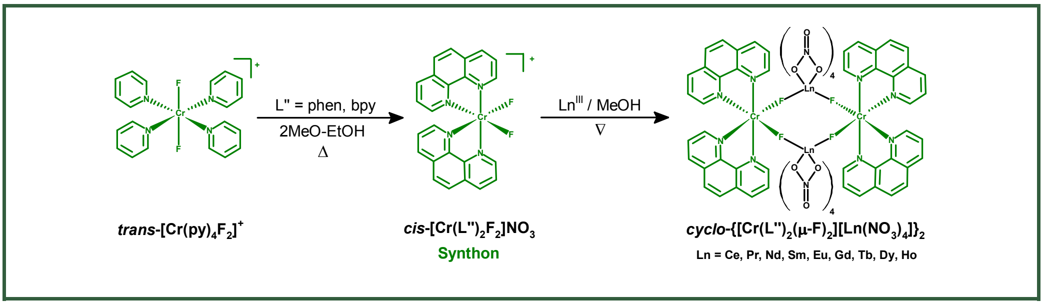

Scheme 6

- complexes *cyclo*-[(μ-F)(L")$_2$Cr(μ-F)Ln(NO$_3$)$_4$] for L"=phen, bpy

General comments

The following preparations were all performed under normal laboratory conditions and the all chemicals and solvents used directly from the supplier without prior purification or drying. The supplier of a given chemical is specified where this first appeares. Also indicated is the purity as declared by the supplier.

All the synthesized compounds has been analyzed by a variety of techniques (*e.g.* elementary elementary anaylsis, IR, MS, EPR,….) The level at which a given compound is characterized depend on the necessity of this. In cases of complete analogous compounds, a representative number of compounds have been selected and extensively characterized, the remaining are then compared with the representative set and characterized in less detail. A description of the techniques, equipment and detailed use in characterization is given in Appendix 2 “Instrumentarium”.

General synthesis of *cyclo*-[(μ-F)(phen)$_2$Cr(μ-F)Ln(NO$_3$)$_4$]

$$2\ trans\text{-}[Cr(L'')_2F_2]NO_3 + 2\ Ln(NO_3)_3 \xrightarrow[\nabla]{MeOH} cyclo\text{-}[(\mu\text{-}F)(L'')_2Cr(\mu\text{-}F)Ln(NO_3)_4]$$

All of the tetranuclear clusters were prepared by the same general protocol given here. A solution of *cis*-[CrF$_2$(phen)$_2$]NO$_3$(0.82 mmol) in MeOH (20 mL) was stirred at room temperature for 30 min. and filtered twice through syringe filters with pore size 0.45 μm. This resulting solution (I) was diluted with an additional 20 mL of MeOH (for prep. of Pr and Nd compounds) or 10 mL of MeOH (for the remaining lanthanoids). A second solution (II) was made up of Ln(NO$_3$)$_3$·nH$_2$O (0.81 mmol) in MeOH (10 mL). The two solutions were mixed and left for crystallization for 24 h at room temperature. Yields do not improve by preparation at 0 °C. The resulting mass of red crystals and white powder (the ratio between these depends on lanthanoid in question) was loosened and brought into suspension. Crystals were harvested by decantation of the byproduct in suspension. The crystals were washed repeatedly with MeOH by decantation until all the pale colored by-product was removed, and dried on a sintered glass filter (No. 3) in a dynamic vacuum. Upon drying solvent loss causes the crystals to lose their luster. A faster precipitation of the product in microcrystalline form can be achieved for Pr, Nd and Sm by not adjusting the volume of solution I with additional MeOH. The suspension of by-product in MeOH is transferred to centrifuge tubes and centrifuged, washed thoroughly with MeOH and dried in a dynamic vacuum.

Yields and analytical data for the individual compounds are given below. Note that drying has resulted in partial solvent loss. For all compounds except that of Gd, the calculated values are given for the solvent free composition:.

cyclo-[(μ-F)(phen)$_2$Cr(μ-F)Ce(NO$_3$)$_4$]$_2$:
Yield: 74 % of theoretical based on Ce(III).
- Elemental analysis calcd. (found) (%) for $H_{32}C_{48}N_{16}O_{24}F_4Cr_2Ce_2$:
  H 1.92(1.72), C 34.38(32.83), N 13.36(12.36).
- By-product: None.

cyclo-[(μ-F)(phen)$_2$Cr(μ-F)Pr(NO$_3$)$_4$]$_2$:
Yield: 82 % of theoretical based on Pr(III).
- Elemental analysis calcd. (found) (%) for $H_{32}C_{48}N_{16}O_{24}F_4Cr_2Pr_2$:
  H 1.92(1.94), C 34.34(33.44), N 13.35(12.44).
- By-product: Present but small amount.

cyclo-[(μ-F)(phen)$_2$Cr(μ-F)Nd(NO$_3$)$_4$]$_2$:
Yield: 87 % of theoretical based on Nd(III).
- Elemental analysis calcd. (found) (%) for $H_{32}C_{48}N_{16}O_{24}F_4Cr_2Nd_2$:
  H 1.91(2.05), C 34.21(34.24), N 13.30(12.69).
- By-product: Present but small amount.

cyclo-[(μ-F)(phen)$_2$Cr(μ-F)Sm(NO$_3$)$_4$]$_2$:
Yield: 87 % of theoretical based on Sm(III).
- Elemental analysis calcd.(found) (%) for $H_{32}C_{48}N_{16}O_{24}F_4Cr_2Sm_2$:
  H 1.90(2.08), C 33.96(33.79), N 13.20(12.52).
- By-product: 0.035 g (Analysis: Found (%): H 1.62, C 24.41, N 7.99).

cyclo-[(μ-F)(phen)$_2$Cr(μ-F)Eu(NO$_3$)$_4$]$_2$:
Yield: 37 % of theoretical based on Eu(III).
- Elemental analysis calcd. (found) (%) for $H_{32}C_{48}N_{16}O_{24}F_4Cr_2Eu_2$:
  H 1.90(1.97), C 33.90(33.59), N 13.18(12.10).
- By-product: 0.070 g (Analysis: Found (%): H, 1.76; C, 26.85; N, 9.16).

cyclo-[(μ-F)(phen)$_2$Cr(μ-F)Gd(NO$_3$)$_4$]$_2$·CH$_3$OH·H$_2$O:
Yield: 27 % of theoretical based on Gd(III).
- Elemental analysis calcd. (found) (%) for $H_{38}C_{49}N_{16}O_{26}F_4Cr_2Gd_2$:
  H 2.17(2.06), C 33.41(33.53), N 12.72(12.25).
- By-product: 0.05 g (Analysis: Found (%): H 1.64, C 25.02, N 7.99).

cyclo-[(μ-F)(phen)$_2$Cr(μ-F)Tb(NO$_3$)$_4$]$_2$:
Yield: 5.5 % of theoretical based on Tb(III).
- Elemental analysis calcd. (found) (%) for $H_{32}C_{48}N_{16}O_{24}F_4Cr_2Tb_2$:
  H 1.88(1.85), C 33.62(33.07), N 13.07(12.06).
- By-product: 0.082 g (Analysis: Found (%): H, 1.63; C, 24.41; N, 7.81).

cyclo-[(μ-F)(phen)$_2$Cr(μ-F)Dy(NO$_3$)$_4$]$_2$:
Yield: 1.8 % of theoretical based on Dy(III).
- Elemental analysis calcd. (found) (%) for $H_{32}C_{48}N_{16}O_{24}F_4Cr_2Eu_2$:
  H 1.87(1.79), C 33.48(32.09), N, 13.02(12.19).
  The isolated crystalline product is contaminated with a crystalline impurity.
- By-product: 0.097 g (Analysis: Found (%): H, 1.67; C, 24.36; N, 7.81).

cyclo-[(μ-F)(phen)$_2$Cr(μ-F)Ho(NO$_3$)$_4$]$_2$:
Yield: Few crystals.
- By-product: 0.107 g (Analysis: Found (%): H, 1.61; C, 24.09; N, 7.75).

cyclo-[(μ-F)(phen)$_2$Cr(μ-F)Er(NO$_3$)$_4$]$_2$: Attempted synthesis,
Yield: None.
- By-product: 0.093 g (Analysis: Found (%): H, 1.64; C, 23.72; N, 7.65).

cyclo-[(μ-F)(phen)$_2$Cr(μ-F)Yb(NO$_3$)$_4$]$_2$: Attempted synthesis.
Yield: None.
- By-product: 0.016 g (Analysis: Found (%): H, 1,24; C, 13.36; N, 5.11).

Genearl synthesis of *cyclo*-$[(NO_3)_4Ln(\mu\text{-}F)Cr(bpy)_2(\mu\text{-}F)]_2$

$$2\ cis\text{-}[Cr(bpy)_2F_2]NO_3 + 2\ Ln(NO_3)_3 \xrightarrow[\nabla]{MeOH} cyclo\text{-}[(NO_3)_4Ln(\mu\text{-}F)Cr(bpy)_2(\mu\text{-}F)]_2$$

cis-$[Cr(bpy)_2F_2](NO_3)$ (1.00 g, 2.15 mmol) was dissolved in methanol (50 ml, VWR Technical) and the resulting orange-red solution was filtered through teflon filter with 0.22 µm pore size. $Ln(NO_3)_3{\cdot}nH_2O$ (ca. 1.1 g, 2.15 mmol, Alfa Aesar 99.9%) was dissolved in methanol (5 ml). Both solutions were cooled on an ice bath for 30 min. and subsequently mixed. Directly upon mixing, precipitation of the pink-orange product commences. The reaction mixture was left stirring for 30 min and the product isolated by filtration followed by repeated washing with methanol (4 ·5 ml). The washing and suction must be performed in such a way that the product at no time dry out and subsequently dried in a dynamic vacuum.

cyclo-$[(NO_3)_4Ln(\mu\text{-}F)Cr(bpy)_2(\mu\text{-}F)]_2$
Yield: 1.370 g (80%)

- Elemental analysis calcd. (found) (%) for $H_{32}C_{40}N_{16}O_{24}F_4Cr_2Nd_2$:
  H 2.03(2.02), C 30.23(30.10), N 14.10(13.55).

cyclo-$[(NO_3)_4Ln(\mu\text{-}F)Cr(bpy)_2(\mu\text{-}F)]_2$
Yield: 1.270 g (74%)

- Elemental analysiscalcd. (found) (%) for $H_{32}C_{40}N_{16}O_{24}F_4Cr_2Sm_2$:
  H 2.01(2.05), C 30.00(29.69), N 13.99(13.56.

cyclo-$[(NO_3)_4Ln(\mu\text{-}F)Cr(bpy)_2(\mu\text{-}F)]_2$
Yield: 1.558g (90%)

- Elemental analysiscalcd. (found) (%) for $H_{32}C_{40}N_{16}O_{24}F_4Cr_2Eu_2$:
  H 2.01(2.06), C 29.94(29.48), N 13.97(13.10).

cyclo-$[(NO_3)_4Ln(\mu\text{-}F)Cr(bpy)_2(\mu\text{-}F)]_2$
Yield: 1.202g (69%)

- Elemental analysiscalcd. (found) (%) for $H_{32}C_{40}N_{16}O_{24}F_4Cr_2Gd_2$:
  H 2.00(2.06), C 29.74(29.32), N 13.87(13.41).

KAPITEL 9

CONCLUSION AND OUTLOOK

CONCLUSION

The synthon and disconnection approach has for a long time been used in organic chemistry - perhaps this approach is one of the reasons for the great success with design and synthesis of new organic structures. Inorganic chemistry do not have a similar general approach, thereto embraces the area too large and diverse part of the elements in The Periodic Table. Nevertheless one is still allowed to be inspired by fundamental idea behind the synthon and disconnection approach in organic chemistry.
In the meaning of a structural units within a molecule which are related to possible synthetic operations, a synthon approach was introduced during the project work to summarize and rationalize the synthesis, reactivity and product stereochemistry for the many different new compounds that were prepared. As a comprehensive and unifying element in the presented reactions was fluoride as either terminal or bridging ligand. The fluoride ligand was anchored to a kinetically robust Cr(III) complex as *trans*-$[Cr(py)_4F_2]^+$ and *cis*-$[Cr(L'')F_2]^+$ for L''=phen, bpy and incorporated through bridging into the final molecule.

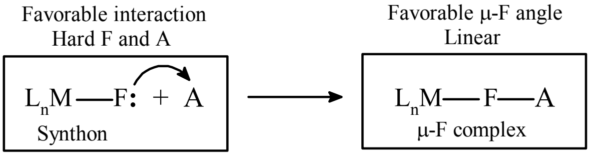

The combined entity of the Cr(III) complex and the fluorido ligand is in this context the "inorganic synthon for the fluorido ligand" (in the final bridged complex). The introduction of the synthon approach is not relevant if it was merely a new word. Nevertheless, the concept proves useful when combined with basic knowledge about the properties of fluoride as ligand *e.g.* the characteristics preference for hard metal ions and preferred linear bridging. It was possible to rationalize product formation of reaction between the fluorido synthons and a series of hard metal ions from different blocks of The Periodic Table (*s*-block, *d*-block, and *f*-block.). The reaction resulted in different products types. The fluorido ligand appeared as both terminal and bridging ligand in both 0D discrete and 1D infinite coordination chain polymers.

FINAL COMMENT AND OUTLOOK

The Phoenix is reborn from the ashes in an eternal cycle, Figure 52. In the Aberdeen Bestiary from ca. 1200 is given the following description of the Phoenix death and rebirth:

Hec quingentos ultra annos vivens, dumse viderit senuisse, collectis aromatum virgultis, rogum sibi instruit, et conversa ad radium solis alarum plausu voluntarium sibi incendium nutrit, seque urit. Postea vero die nona avis de cineribus suis surgit. [409]

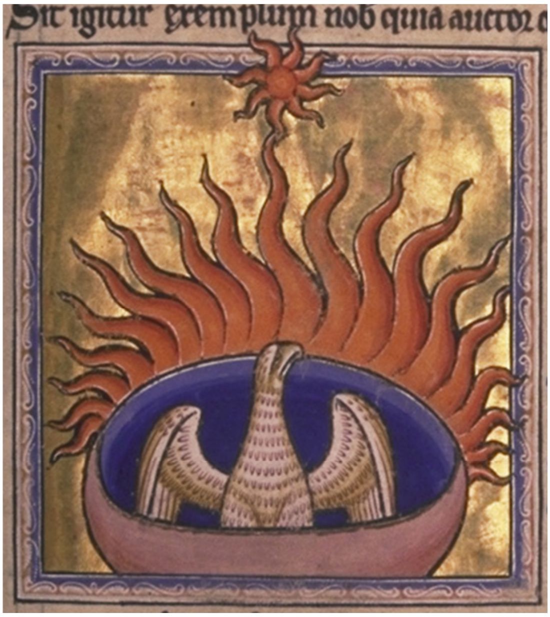

Figure 52 Phoenix, The Aberdeen Bestiary ca. 1200

This ancient creature has been a symbol of chemistry for centuries. [410, 411] Much can happen during the course of time, not at least in an area as chemistry that has moved from the metaphysical and philosophical concepts of alchemy to the logical science of today. Nevertheless, the Phoenix has been able to maintain its meaning and significance. The eternal regeneration cycle is for the chemist of today not an abstract metaphysical idea as for the alchemist - but a direct and concrete concept. A picture of the chemist work with nature's building blocks - breaking them down to ashes and building them together in new forms being different from the origin and yet composed of the same material - as the Phoenix.

This thesis and the year's of work it represents is in many ways related to the picture of the Phoenix, Figure 52. As an inorganic coordination chemist with special interest in synthesis it is the hope that this thesis reflects this picture and that the presented results in a more general way tells the story of the joy of working with nature's building blocks - trying to understand their properties, beeing amazed of their many abilities, inspired if they tease and happy when they “talk” to and tell news from their world.

On a more direct plan this thesis has hopefully shown that fluoride are capable of forming a rich variety of products both as terminal- and bridging ligand. Due to the cohernet nature of the results they were grouped together under what was presented as the “synthon approach”. It would be arogant to say that the method is completely general and that it will predict the outcome of a reaction with certainty. This was not the intention. The approach is intended as part of the synthetic chemist toolbox - a way of thinking that can provide inspiration and guidance. Nevertheless, it is not unreasonable to claim that the method in case of relatively simple systems gives words like “design” a more rational meaning. For these systems the synthon approach gives high stereochemical- and reactive control of the fluorido ligand. This makes it possible to vary and optimize on a large number of parameters without the characteristic and undesirable side reactions of fluoride need to be a problem. Parameters that can be varied are solvent, hardness of the incoming metal ions and presence of other ligands.

After the successful synthesis of *cyclo*-$[(NO_3)_4Ln(\mu\text{-}F)Cr(L'')_2(\mu\text{-}F)]$ and the potential of the reaction was recognized, other people at the Department of Chemistry University of Copenhagen adopted the method and initiated their own investigations in prolongation of the results. [412, 413] These studies include, among other things parameter variations which gave rise to several new complexes which have formed the basis for among other things magnetic studies.

A huge and interesting task lies before those how are interested in fluoride as ligand. Many possibilities exists to continue the work initiated with this project. As a final overview is given a few ideas for futher work.

- With the synthesis and magnetic study of *catena*-[Mn(μ-F)(salen-5H)] was given the first magnetic description of the unsupported fluorido ligand in a type of compounds that play a central role in the development of new magnetic materials. A number of analogue systems can be synthesized on basis of the described method and systematic studies of the fluorido ligands magnetic interaction can be done. (See chapter 4)

- As it has been shown with work from University of Copenhagen some of the Ln^{III}–F–Cr^{III} complexes possess SMM properties which are an area of much attention. By expanding the synthesis method of the synthon *cis*-$[Cr(L'')(H_2O)(F)]^{2+}$ to other robust

fluorido precursors of Cr(III) *e.g.* with three or more fluorido ligands. It is possible that new synthons with unfamiliar coordination geometry can be obtained. Additionally, other kinetic robust metal ions than Cr(III) could be tested as a metal centre for the synthon. (See chapter 6)

- Hydrogen bonding network both in solution and in solid state has received much interest in literature in connection with model and practical receptor systems. The two fluorido bridged complexes of Na(I), Li(I) and Cr(III) suggest that a large number of similar systems can be prepared by varying the alkali metal ion, counter ion and solvent. (See Chapter 5)

It is my hope that the work and results presented in this thesis, in form of both synthesis of a number of new fluorido complexes as well as the methodological approach behind will lead to renewed interest in fluoride as a ligand in both terminal and bridge systems.

Copenhagen March 30, 2012

Torben Birk

APPENDIX 1: ABBREVIATIONS & UNITS

GENERAL COMMENTS

The following tables in this appendix lists the majority of abbreviations and symbols used in the thesis. The abbreviations are devided into subgroups such as “functional groups”, “solvent” etc. in order to enhance readability. It is assumed that the reader, based on his chemical knowledge, can decipher in which table a given abbreviation should be sought.

The abbreviations used in the thesis are arranged in the following groups:

- General symbols for chemical entities (Table 26)
- Acronyms for *e.g.* concepts and experimental techniques (Table 25)
- Abbreviations for functional groups and solvent (Table 27 and Table 28)
- Abbreviations for ligands and other entities (Table 29)
- Abbreviations of symbols (Table 30)

In these lists occur primarily abbreviations used repeatedly in the thesis. Abbreviations used only one or a few times are translated at the specific place(s) they occur. If both the common and systematic name of a chemical species are too complicated for practical use, abbreviations have been introduced in the text. The list can therefore be used to “translate” the name and abbreviation to a structural formula.

Abbreviations of ligands
The abbreviations of ligands follows either directly from the “*International Union of Pure and Applied Chemistry*” (IUPAC) list of the most common abbreviation or is constructed in agreement with the general recommendations. [346] There are examples in the literature with

regard to inconsistent use of abbreviations so that their meanings may be different or exchanged between two different works (among other the guidelines of IUPAC). Examples of replacement can be seen *e.g.* in case of propan-1,2-diamine and propan-1,3-diamine and their abbreviations pn and tn.
It cannot be hidden that name for ligands of a certain size based on systematic nomenclatur becomes unmanageable. As an example, the imino ligand with the common name "*N,N'*-bis(salicylidene)-ethylenediamine" and abbreviation "H_2salen" has the systematic name "2,2'-[ethane-1,2-diylbis(azanylylidenemethanylylidene)]-diphenole". As a consequence the tables of abbreviations include both the common and systematic name. Also wherever possible in the text, such ligand will be referred to by its ligand abbrevation and not by the name.
Many ligands exist both in a neutral and a charged form linked, typically, by a deprotonation. In connection with the new systems presented in the thesis it has been attempted to apply the following destinction between the different forms of the ligand: The free form of the ligand is given *e.g.* by "H_2salen" wereas the coordinating, deprotonated ligand is abbreviated as "salen". This convention is not used on systems from the literaure in order to avoid confusion between the notation of the thesis and the literature. This is exemplified by "dpa", which is applied in the litterature as representation for both the protonated and non protonated form. A more systematic approach (with or without a charge indication) should involve "H_2dpa^+", "Hdpa" and "dpa^-".

Physical quantities and their units
The symbols used in the thesis are attempted whenever possible to be in accordance with the IUPAC recommendations. [414] The system of units used for physical quantities in the thesis is to the greatest extent possible in accordance with the "*International System of Units*", SI. [415] Nevertheless, there are regrettably in several fields of chemistry tradition for using an alternative selection of units. For example, bond lengths are traditionally given in units of ångstroms, Å (10^{-10} m) in connection with structural descriptions and in spectroscopy, the wavenumber in unit of cm^{-1} is used as an derived quantity of the wavelength and simultaneously as a unit of energy. These traditions are respected.
Especially the area of magnetochemistry represents a challenge in terms of units. Pragmatic formulas will follow the SI unit system, but experimental magneto-chemical data will be listed in the Gauss- or emu-unit system, due to the fact that measured values, are directly determined in these units. See Table 24 for the conversion between different units.

Physical Quantity	Symbol	CGS & emu-unit	Factor	SI-unit
Wavenumber	$\tilde{\nu}$	cm^{-1}	10^{2}	m^{-1}
Length	l	Å, Ångstrom	10^{-10}	m
Magnetic induction	B	G, Gauss	10^{-4}	T, Tesla
Magnetic field strength	H	Oe, Oersted	$1/4\pi\cdot10^{3}$	$A\cdot m^{-1}$
Volumen Magnetisation	M	$emu\cdot cm^{-3}$	10^{3}	$A\cdot m^{-1}$
Magnetic moment	μ	emu, $erg\cdot G^{-1}$	10^{-3}	$J\cdot T^{-1}$
Mass Susceptibility	χ_{mass}	$cm^{3}\cdot g^{-1}$, $emu\cdot g^{-1}$	$4\pi\cdot10^{-3}$	$m^{3}\cdot kg^{-1}$
Molar Susceptibility	χ_{mol}	$cm^{3}\cdot mol^{-1}$, $emu\cdot mol^{-1}$	$4\pi\cdot10^{-6}$	$m^{3}\cdot mol^{-1}$
Volumen Susceptibility	χ_{vol}	dim. less, $emu\cdot cm^{-3}$	4π	dim. less

Table 24 Conversion between units in different unit system

TABLES OF ABBREVIATIONS

Abbreviation	Meaning
AOM	Angular overlap model
DFT	Density functional theory
EI^{+} / EI^{-}	Electron impact mass spectrometry
EPR	Electron paramagnetic resonance spectroscopy
ES^{+} / ES^{-}	Pneumatically assisted electrospray mass spectrometry
FAB^{+} / FAB^{-}	Fast-atom bombardment mass spectrometry
HOMO	Highest occupied molecular orbital
HSAB	Hard-soft-acid-base
IR	Infrared spectroscopy
LMCT	Ligand to metal charge transfer
LUMO	Lowest unoccupied molecular orbital
MS	Mass spectrometry
ROS	Reactive oxygen species
UV/vis	Ultraviolet-visible spectrophotometry
X-ray	Single crystal X-ray diffraction (Röntgen)
SMM	Single-molecule magnets
SQUID	Superconducting quantum interference device

Table 25 Acronyms. Including experimental and calculation techniques

Abbreviation	Meaning
A	General element
L	General monodentate ligand
L", L‴	General bi- or tri-dentate ligand (with nitrogen as ligators)
Ln / Ln(III)	General Lanthanoid (ion)
M^{n+}	General transition metal with formal charge +n
S	General unspecified coordinating species *e.g.* solvent
SB	General tetradentate Schiff base
X, Y	General monodentate ligand, atom or anion
Δ	Heating - used in reaction equations
∇	Cooling - used in reaction equations

Table 26 General symbols used in reaction equations and for chemical entities

Functional groups	Radikal	Structural formula
Me	Methyl	CH_3–
Et	Ethyl	CH_3CH_2–
Pr	Propyl	$CH_3CH_2CH_2$–
^{i}Pr	*iso*-propyl	$(CH_3)_2CH$–
^{t}Bu	*tert*-butyl	$(CH_3)_3C$–
^{sec}Bu	*sec*-butyl	$CH_3CH_2CH(CH_3)$ –
Bz	Benzyl	$C_6H_5CH_2$–
Ph	Phenyl	C_6H_5–

Table 27 Abbreviations for functional groups

Solvent	Name	Structural formula
MeOH / MeO^-	Methanol / Methoxido	CH_3OH / CH_3O^-
MeCN	Acetonitrile	CH_3CN
$MeNO_2$	Nitromethane	CH_3NO_2
EtOH	Ethanol	CH_3CH_2OH
2MeO-EtOH	2-methoxyethanol	$CH_3O(CH_2)_2OH$
Et_2O	Diethylether	$(CH_3CH_2)_2O$
dmf	*N*,*N*-dimethylformide	$(CH_3)_2NCOH$
AcOH / AcO^-	Acetic acid / Acetate	CH_3COOH / CH_3COO^-
nmf	*N*-Methylformamide	CH_3NHCHO
thf	Tetrahydrofuran (Oxolane)	C_4H_8O

Table 28 Abbreviations for solvents

Abbreviation	Name	Connecting formula
bispicen	*N,N′*-bis(2-pyridylmethyl)-1,2-ethanediamine	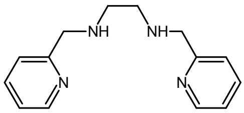
bispicMe$_2$en	*N,N′*-bis(2-pyridylmethyl)-*N,N′*-dimethyl-1,2-ethanediamine	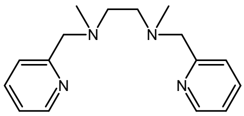
bispictn	*N,N′*-bis(2-pyridylmethyl)-1,3-propanediamine	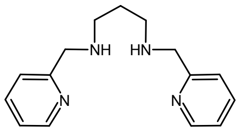
bpea	*N,N′*-bis(2-pyridylmethyl)ethylamine	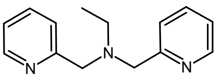
2,2′-bpy *or* bpy	2,2′-bipyridine	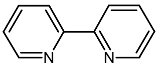
4,4′-bpy	4,4′-bipyridine	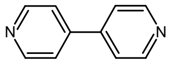
chxn	Cyclohexane-1,2-diamine	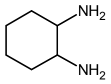
12-crown-4	1,4,7,10-tetraoxacyclododecane	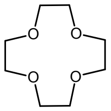
15-crown-5	1,4,7,10,13-pentaoxacyclopentadecane	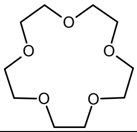

Table 29 Abbreviations for ligands and other entities

Abbreviation	Name	Connecting formula
18-crown-6	1,4,7,10,13,16-hexaoxacyclooctadecane	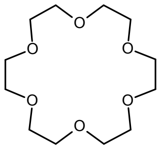
$[14]aneN_4$ *or* cyclam	1,4,8,11-tetraazacyclotetradecane	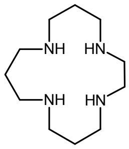
1,4-C_2-cyclam	1,5,8,12-tetraazabicyclo[10.2.2]hexadecane	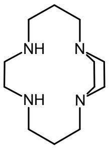
1,11-C_3-cyclam	1,4,8,11-tetraazabicyclo[9.3.3]heptadecane	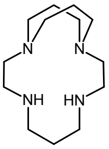
dpa	Bis(2-pyridyl)amine	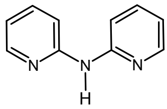
en	Ethan-1,2-diamine	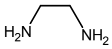
gdn	Guanidine/guanidinum ion	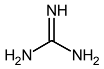
Hacac	Pentan-2,4-dione, acetylacetone	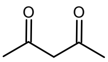

Table 29 (*continued*) Abbreviations for ligands and other entities

Abbreviation	Name	Connecting formula
salen	2,2′-[ethane-1,2-diylbis (azanylylidenemethanylylidene)]diphenole	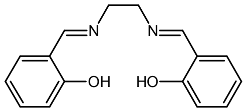
salpn *or* saltn	2,2′-[ethane-1,3-diylbis (azanylylidenemethanylylidene)]diphenole	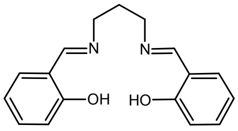
salmen	2,2′-[propane-1,2-diylbis (azanylylidenemethanylylidene)]diphenole	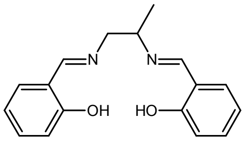
saldmen	2,2′-[2-methylpropane-1,2-diylbis (azanylylidenemethanylylidene)]diphenole	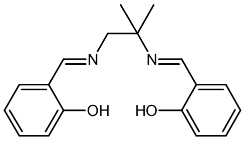
saltmen	2,2′-[2,3-dimethylbutane-2,3-diylbis (azanylylidenemethanylylidene)]diphenole	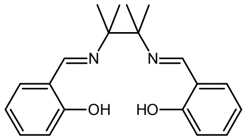
salphen *or* saloph	2,2′-[benzen-1,2-diylbis (azanylylidenemethanylylidene)]diphenole	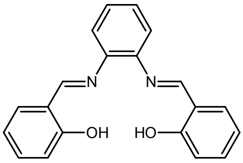
saldphen	2,2′-[1,2-diphenylethane-1,2-diylbis(azanylylidenemethanylylidene)]diphenole	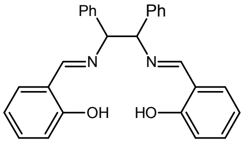
salcy	2,2′-[cyclohexane-1,2-diylbis (azanylylidenemethanylylidene)]diphenole	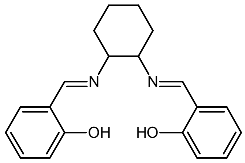

Table 29 (*continued*) Abbreviations for ligands and other entities

Abbreviation	Name	Connecting formula
acphen	Bis(*o*-hydroxyacetophenone)ethylenediimine	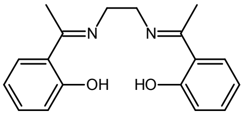
Me_3tacn	1,4,7-trimethyl-1,4,7-triazacyclononane	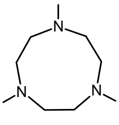
Mn_{12}	$[Mn_{12}(OAc)_{16}(H_2O)_4O_{12}]\cdot 2AcOH\cdot 4H_2O$	See Figure 11
pipz	Piperazine	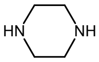
pn	Propan-1,2-diamine	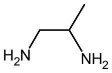
tn	Propan-1,3-diamine	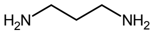
py	Pyridine	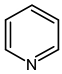
phen	1,10-phenantroline	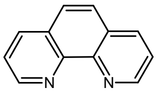
pico	2-(Aminomethyl)pyridine	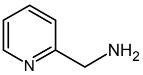
terpy	2,2′;6,2″-terpyridine *or* terpyridine	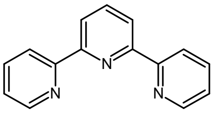
2,3,2-tet	*N,N′*-bis(2-aminoethyl)propane-1,3-diamine	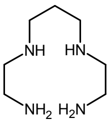

Table 29 (*continued*) Abbreviations for ligands and other entities

Symbol	Meaning	Symbol for SI unit
A	Hyper- /Superhyperfine coupling constant	Hz
B	Magnetic induction / Magnetic flux density	T
C	Curie constant	$m^3 \cdot K \cdot mol^{-1}$
D	Axial zero field splitting parameter	
D	Bond strength or Bond dissociation energy	J
E_{ea}	Electron affinity	J
E	Rhombic zero field splitting parameter	
E_i	Energy of state	J
E_i	Ionization energy	J
e_σ	σ-bonding parameter	
e_π	π-bonding parameter	
F	Helmholtz free energy	J
g	g-factor	dim. less
$\hat{H}$	Hamiltonian	J
H	Magnetic field strength / magnetizing force	$A \cdot m^{-1}$
$\hbar$	Planck constant / 2π 1.054571628(53) $\cdot 10^{-34}$	J·s
$\Delta_f H$	Enthalpy of formation	$J \cdot mol^{-1}$
$\Delta_{sub} H$	Enthalpy of sublimation	$J \cdot mol^{-1}$
I	Nuclear spin quantum number	dim. less
J	Exchange coupling constant	J
J	Total angular momentum qn.	dim. less
k_B	Boltzmann constant: 1.3806504(24)$\cdot 10^{-23}$	$J \cdot K^{-1}$
L	Total electron orbital angular momentum qn.	dim. less
l	Electron orbital angular momentum qn.	dim. less
M	Magnetization	$A \cdot m^{-1}$
M_I	Nuclear magnetic spin qn.	dim. less
M_J	Total z-axis angular momentum qn.	dim. less
M_L	Total z-axis electron orbital qn.	dim. less
M_S	Total z-axis electron spin qn.	dim. less
m_l	z-axis electron orbital qn.	dim. less

Table 30 Symbols used in the thesis

Symbol	Meaning	Symbol for SI unit
m_s	z-axis electron spin qn.	dim. less
N_A	Avogadro constant $6.02214179(3)\cdot10^{23}$	mol^{-1}
Q	Heat	J
S	Entropy	$J\cdot mol^{-1}\cdot K^{-1}$
S	Total electron spin angular momentum qn.	dim. less
s	Electron spin angular momentum qn	dim. less
T	Thermodynamic Temperature	K (or °C)
U	Internal energy	J
U	Lattice energy	J
w	Work	J
Z	Partition function	1
Δ	Enantiomeric conformation	–
Δ	Ligand filed splitting	J
φ	Torsion angle	rad (or °)
Λ	Enantiomeric conformation	–
η	Chemical hardness	J (or eV)
χ	Electronegativity (Mulliken scale)	J (or eV)
χ	Electronegativity (Pauling scale)	dim. less
χ_{mass}	Mass magnetic susceptibility	$m^3\cdot kg^{-1}$
χ_{mol}	Molar magnetic susceptibility	$m^3\cdot mol^{-1}$
χ_{vol}	Volume magnetic susceptibility	dim. less
μ_0	Magnetic constant: $4\pi\cdot10^{-7}$	$H\cdot m^{-1}$
μ_B	Bohr magneton: $9.27400915(23)\cdot10^{-24}$	$J\cdot T^{-1}$
σ	Chemical softness	J^{-1} (or eV^{-1})
θ	Scattering angle	rad (or °)
θ	Weiss temperature	K
ν	Frequency	Hz
$\tilde{\nu}$	Wavenumber.	m^{-1} or J
~	Approximately	–

Table 30 (*continued*) Symbols used in the thesis

SYSTEMATIC NAMING OF COMPLEXES DESCRIBED IN THESIS

Several of the new complexes described in the thesis are bridged complexes or polymeric in nature, which gives rise to complicated naming according to the systematic IUPAC nomenclature. Consequently, the complexes are only referred to by their formulas in the thesis. Moreover, the used formulas are often simplified relative to the strict IUPAC guidelines. In the following it is *attempted* to assign systematic names and formula for the given complexes. Principal Scientist, PhD Ture Damhus is appreciated for help with the naming *cyclo*-$[(NO_3)_4Nd(\mu\text{-}F)Cr(phen)_2(\mu\text{-}F)]_2$.

- ***catena*-[Mn(μ-F)(salen-5H)]**
  catena-poly[μ-Fluorido-2,2′-[ethane-1,2-diylbis(azanylylidenemethanylylidene)]diphenolato] manganese(III)

- ***trans-catena*-poly$[Na(H_2O)_4(\mu\text{-}F)Cr(py)_4(\mu\text{-}F)](HCO_3)_2$**
  trans-catena-poly[[tetraaquasodium(I)]-μ-fluorido-[(tetrakis(pyridine)chromium(III))]-μ-fluorido] bis(hydrogencarbonate)]

- ***trans*-$[Cr(py)_4F(\mu\text{-}F)\{Li(H_2O)_{n=3,4}\}_{m=0,1}]Cl_5\cdot 6H_2O$**
  trans-(difluoridotetrakis(pyridine-1κ*N*)chromium(III))(triaqua-2κ*O*-μ-fluorido-fluorido-1κ-tetrakis(pyridine-1κ*N*)chromium(III)lithium(I))(tetraaqua-2κ*O*-μ-fluorido-fluorido-1κ-tetrakis(pyridine-1κ*N*)chromium(III)lithium(I))chloride hexahydrate

- ***cis*-$[Cr(phen)_2F_2]ClO_4\cdot H_2O$**
  cis-difluoridobis(1,10-phenantroline-κ^2*N,N′*)chromium(III) perchlorate monohydrate

- ***cis*-$[Cr(bpy)_2(H_2O)(F)](ClO_4)_2\cdot 2H_2O$**
  cis-aquabis(2,2′-bipyridine-κ^2*N,N′*)fluoridochromium(III) bis(perchlorate) dihydrate

- ***cyclo*-$[(NO_3)_4Nd(\mu\text{-}F)Cr(phen)_2(\mu\text{-}F)]_2$**
  tetra-μ-fluorido-1:3κ^2*F*;1:4κ^2*F*;2:3κ^2*F*;2:4κ^2*F*-tetrakis(nitrato-1κ^2*O,O′*)tetrakis(nitrato-2κ^2*O,O′*)bis(1,10-phenantroline-3κ^2*N,N′*)bis(1,10-phenantroline-4κ^2*N,N′*)dineodymium(III)dichromium(III)

  alternatively

  tetra-μ-fluorido-1:3κ^2*F*;1:4κ^2*F*;2:3κ^2*F*;2:4κ^2*F*-tetra(nitrato-1κ^2*O,O′*)tetra(nitrato-2κ^2*O,O′*)di(1,10-phenantroline-3κ^2*N,N′*)di(1,10-phenantroline-4κ^2*N,N′*)-quadro-dineodymium(III)dichromium(III)

APPENDIX 2: INSTRUMENTARIUM

GENERAL COMMENTS

This appendix sum up the general procedure and technical specifications of the equipment used in the characterization and analysis of the compounds obtained during the work described in this thesis.
Measurements and analyses were mostly done at the University of Copenhagen, performed by the undersigned, in collaboration with scientific colleagues or by the technical staff. Some measurements were made with help from international collaborators.

- Elementary analysis (C, H, N, F, Na, Cr, Mn)
- Electron Paramagnetic Resonance spectroscopy (EPR)
- Infrared spectroscopy (IR)
- Ultraviolet / visual spectroscopy (UV/vis)
- Mass spectrometric methods (FAB, EI, ESP)
- Magnetic susceptibility (SQUID)
- Thermogravimetry
- Single crystal- and powder- X-ray diffraction

Details of single crystal- and powder- X-ray diffraction are given separately in Appendix 3.

Elementary analysis

Carbon, Hydrogen and Nitrogen

Performed at the section for microanalysis at University of Copenhagen by laboratory technician Mrs. Brigitta Kegel by use of a CE Instruments FLASH 1112 series EA.
It belongs to the standard procedure to treat samples containing fluorine with V_2O_5 before elemental analysis. In a number of cases, double determination both with and without added V_2O_5 have been performed. These tests showed that it is usually not necessary to add V_2O_5 before analysis. Nevertheless, in a few isolated cases a clear difference was observed and treatment in the presence of V_2O_5 was required in order to get a reliable test results.

Other elements

- **Sodium, Na**
  Quantitative determination of Na was performed by flame emission on a Kipp H45 emission photometer.

- **Chromium, Cr**
  Quantitative determination of Cr was performed by atomic absorption spectrophotometry by use of a Perkin-Elmer 2280 Atomic Absorption Spectrophotometer.

- **Manganese, Mn and Fluorine, F**
  Performed by the commercial Mikroanalytisches Laboratorium KOLBE.
  Höhenweg 17, D-45470 Mülheim an der Ruhr.

Electron Paramagnetic Resonance spectroscopy (EPR)

X-band spectra were obtained on a Bruker Elexsys E 500 spectrometer equipped with a dual mode cavity ER 4116DM, ER 035 M NMR-Gaussmeter, and an EIP 538B frequency counter. The system was cooled with an Oxford Instruments cryostat ESR-900 controlled by an Oxford Instruments ITC 503 temperature unit. The magnetic field data were calibrated by use of the NMR-Gaussmeter. The data obtained were analysed using the software "*SIM*" written by Weihe. [283]

Infrared spectroscopy (IR)

Mid-range FTIR spectroscopy of the compounds was recorded as KBr-pellets using a Bio-Rad Excalibur Series FTS 30000MX FT-IR Fourier transform infrared spectrometer within the frequency range of 4200–400 cm^{-1}. The sampels for mesurrment were prepared as a pellet in a matrix of KBr by pressing a powdered mixture of the samples in KBr in vacuo using a hydraulic

press applying a pressure of ca. 10.000 kg·cm^{-2} for 5 min. The measurements was performed at room temperature and in an atmosphere of nitrogen.
I addition a compact Bruker ALPHA FT-IR spectrometer was used. Spectra obtained by the Bio-Rad Excalibur apparatus and the Bruker ALPHA were compared and found to be identical.

Wave numbers are assigned to each band by visual inspection of the local minimum in the spectrum using the program "*Origin Pro*" Version 8. The bands intensities are indicated qualitatively using the following abbreviations: *w* = weak, *m* = medium, *s* = strong and *b* = broad signal shape. In accordance with normal practice, the wave numbers are given as nearest whole wave number.

Ultraviolet / visual spectroscopy (UV/vis)
UV/vis spectra were measured in a 1 cm path-length quartz cell using a Perkin Elmer UV/vis Lambda 2 spectrophotometer.
Measuring solutions were prepared by transferring the substance directly to the volumetric flask (10-20 mL) placed on the analytical balance before the substance was dissolve quantitatively. Use of the stock solutions have been avoided to eliminate any errors.

Mass spectrometric methods

- **Fast-atom bombardment mass spectrometry (FAB+ / FAB–)**
  Fast atom bombardment mass spectra, with Xe ions accelerated by 6 kV were recorded on a Jeol JMS-HX 110A Tandem Mass Spectrometer with positive and negative ion detection using *m*-nitrobenzyl alcohol as matrix. [416]

- **Pneumatically assisted electrospray mass spectrometry (ESP+ / ESP–)**
  Recorded on a Micromass Q-TOF spectrometer. Cone-voltages in the range 20–45 V from solutions with 5-50 μM nominal concentrations.

- **Electron impact mass spectrometry (EI+ / EI–)**

Magnetic susceptibility
Quantum Design MPMS XL SQUID magnetometer equipped with a 5 T dc magnet. Quantum Design, Inc. 6325 Lusk Boulevard San Diego, CA 92121.
Susceptibility measurements were conducted at H_{dc} = 1 kOe in the temperature range 1.8 or 3.0–300 K on a polycrystalline sample in a polycarbonate capsule immobilized in a frozen *n*-eicosane matrix to avoid torqueing. The susceptibility was corrected for diamagnetic contributions by means of Pascal constants. For magnetisation measurements the field was sweeped to ±5 T at 1.8 K at an average rate of 160 Oe·min^{-1}. The Ac experiments were

conducted at selected frequencies between 50 Hz and 1500 Hz with H_{ac} = 3.8 Oe with or in the absence of a static dc field (H_{dc}< 2 kOe).
The obtained magnetic susceptibility data was analyzed using the program "*MagProp*" there is a integret part of the program package DAVE from developed by NIST Center for Neutron Research. [417]

Preparation of sample
A portion of the compound is placed in a small agate mortar and vigorously grinded to a fine homogeneous powder. The powder is placed in capsule as shown in Figure 54. A polycarbonate capsule, **1** is cut so the two parts gets nearly same length, **2**. and so the assembled capsule has a length of 5-6 mm The weights of the two halves is determined whereupon ~20 mg is placed in the capsule part with smallest diameter, **3**. The capsule is assembled and weighed and then pressed with a metal spatula in X shaped pattern in top and bottom to give a approximated cylinder, **4**.
The sample is placed in a plastic straw and fixed with sewing cotton as shown in Figure 54. The number sequence (1–8) in Figure 54 represents a workable way to sew from start to end.

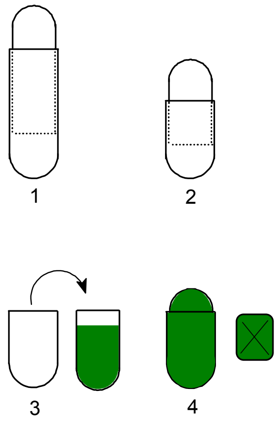

Figure 53 Part 1: Preparation of the sample for SQUID measurement.

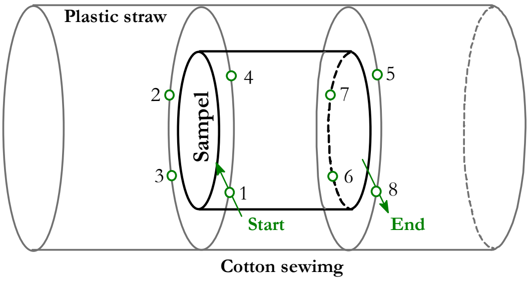

Figure 54 Part 2: Preparation of the sample for SQUID measurement.

Pascal's constants
Diamagnetic correction of the measured molar magnetic susceptibility is standard practice and will often be in order of 10^{-10} to 10^{-11} $m^3 \cdot mol^{-1}$, alternatively in the Gaussian-cgs system of units 10^{-5} to 10^{-6} $emu \cdot mol^{-1}$. See Appendix 1 "Abbreviations & Units". Correction of temperature independent diamagnetism in the measured molar magnetic susceptibility for a molecule is done by acceptance of the additivity of diamagnetic

corrections, the Pascal constants, for the component of the molecule. In this approximation it is assumed that a general molecule can be divided into M atoms connected by N bonds. The diamagnetic correction is then given as a sum of individual contributions from the M atoms, $\chi_{D,i}$ and N bonds λ_i.

$$\chi_D = \sum_{i=1}^{N} \chi_{D,i} + \sum_{i=1}^{M} \lambda_i \tag{6.18}$$

It is not general practice to give references to the used Pascal's constants for the correction. This practice is problematic because these corrections over time must be considered to change when measurements that are more accurate are available. The small numerical value of the correction compared with the paramagnetic contribution means that the sensitivity to small variations in the former is limited. Values for the diamagnetic contribution are given in various textbooks and monographs but frequently without references. [83] Many of the measurements dates from Pascals monumental work from around 1910. [418] A new detailed review of the topic can be found in [419].

Comment

The measurements were performed at the Department of Chemistry, University of Copenhagen by Kasper S. Pedersen or in collaboration.

Associate professor Dr. Susanne Mossin performed a series of measurements of magnetic data for the *cyclo*–$[(NO_3)_4Ln(\mu\text{-}F)Cr(bpy)_2(\mu\text{-}F)]_2$ class of compounds during her former residence at Friedrich-Alexander-Universty of Erlangen-Nuremberg, Germany. As a result of changes in the method of synthesis of these compounds, the measurements has since been repeated in Copenhagen. It is these latter measurements that are presented in this thesis.

Thermogravimetric analysis (TGA)

Thermogravimetric analysis was performed at Department of Chemistry, Technical University of Denmark, DK-2800 Kgs. Lyngby Denmark with assistance from associate professor Dr. Susanne Mossin.

The equipment used was a Mettler Toledo Thermogravimetric Analyzer, TGA/DSC 1 SF/566. The samples were placed in ceramic capsules of alumina (70 µL) and heated in an atmosphere of N_2 (flow of 20.0 $mL\cdot min^{-1}$) from an initial temperature of 25.0 °C to 160.0 (or 180.0) °C with a heating rate of 2.00 (or 1.00) $°C\cdot min^{-1}$.

APPENDIX 3: X-RAY DIFFRACTION

GENERAL COMMENTS

Single crystal X-ray diffraction and X-ray powder diffraction has been performed on a number of the complexes described in this thesis. This appendix provides general information about the method and equipment used for data collection and how the structures have been solved and refined. The appendix also includes tables of single crystal X-ray diffraction data for the individual complexes.

The following complexes have been characterized by X-ray diffraction:

**1**	*catena*-[Mn(μ-F)(salen-5H)]	**6**	*cis*-$[Cr(phen)_2F_2]ClO_4{\cdot}H_2O$
**2**	*catena*-[Mn(μ-F)(salen-5F)]	**7**	*cis*-$[Cr(bpy)_2(F)(H_2O)](ClO_4)_2{\cdot}2H_2O$
**3**	*catena*-[Mn(μ-F)(salen-5Cl)]	**8**	*cyclo*-$[(NO_3)_4Nd(\mu\text{-}F)Cr(phen)_2(\mu\text{-}F)]_2$
**4**	*trans-catena*-poly$[Na(H_2O)_4(\mu\text{-}F)Cr(py)_4(\mu\text{-}F)]\ (HCO_3)_2$	**9**	$[(NO_3)_4Nd(\mu\text{-}MeO)_2Cr(phen)_2]$
**5**	*trans*-$[Cr(py)_4F(\mu\text{-}F)\{Li(H_2O)_{n=3,4}\}_{m=0,1}]Cl_5{\cdot}6H_2O$		

Table 31 Complexes characterized by single crystal X-ray diffraction

SINGLE CRYSTAL DIFFRACTION

The structure dertermination by single crystal diffraction has been performed partly at the Department of Chemistry, University of Copenhagen, Denmark and partly at the Max-Planck Institut für Bioanorganische Chemie, Germany.
Technical assistance regarding collection of the X-ray diffraction data in Copenhagen has been given by Flemming Hansen (Centre of Crystallographic Studies, University of Copenhagen), Magnus Schau-Magnussen and Johan Vibenholt regarding the complexes (**1**) to (**7**). The undersigned has solved the structures of (**6**) and (**7**), and partially solved the structures of (**1**), (**4**), and (**5**) with the assistance of Jesper Bendix and Schau-Magnussen. Johan Vibenholt has been responsible for solving the structure of complexes (**2**) and (**3**) while Dr. Thomas Weyhermüller (Max-Planck-Institut für Bioanorganische Chemie. Stiftstrasse 34-36 /D-45470 Mülheim an der Ruhr. PO Box 10 13 65 /D-45413 Mülheim an der Ruhr) has been responsible for data collection and solving the structures in case of the complexes (**8**) and (**9**). These structures have subsequently been determined again in Copenhagen, and it is these determinations that are used in Papers 7 and 8.

The following Table 32 summarize the type of apparatus, software and methods used in data collection and refinement. In all cases the structures were solved using direct methods and all non-hydrogen atoms were refined using anisotropic displacement parameters.

Crystallograhical data of the compounds published have been desposited with the Cambridge Crystallographic Data Center allocated with the deposit numbers found in connection with the X-ray data of the compunds given in this section. Copy of the data can be obtained free of charge on application to:

Cambridge Crystallographic Data Center
12 Union Road, Cambridge C2B 1EZ, UK,
fax: +44 1223 336033
e-mail:deposit@ccdc.cam.ac.uk

		Specifications	Ref.
Data collection	Apparatus	Nonius Kappa CCD area detector diffractometer	
	Radiation source	Fine-focus sealed tube	
	Monochromator	Graphite	
	Radiation type	Mo $K\alpha$	
	Radiation wavelength, λ	0.71073 Å	
	Absorption correction	Gaussian integration	[420]
Refinement	Refinement	Anisotropically on F^2	
	Least-squares matrix	Full	
	Primary atom site location	Structure-invariant direct methods	
	Secondary atom site location	Difference Fourier map	
	Hydrogen site location	Inferred from neighbouring sites	
	H-atoms parameters	Constrained	
	Extinction correction	None	
Computing details	Data collection	COLLECT	[421]
	Cell refinement	COLLECT	[421]
	Data reduction	EVALCCD	[422]
	Structure solving	SHELXS-97	[423]
	Refine structure	SHELXL-97	[423]

Table 32 Collection and refinement for single crystal diffraction for complex 1-9

Special details for the refinement and presentation

Geometry

All e.s.d.'s (except the e.s.d. in the dihedral angle between two l.s. planes) are estimated using the full covariance matrix. The cell e.s.d.'s are taken into account individually in the estimation of e.s.d.'s in distances, angles and torsion angles; correlations between e.s.d.'s in cell parameters are only used when they are defined by crystal symmetry. An approximate (isotropic) treatment of cell e.s.d.'s is used for estimating e.s.d.'s involving l.s. planes.

Refinement

Refinement was performed of F^2 against ALL reflections. The weighted R-factor w R and goodness of fit, S, are based on F^2. Conventional R-factors R are based on F, with F set to zero for negative F^2. The threshold expression of $F^2 > 2\sigma(F^2)$ is used only for calculating R-factors(gt) etc. and is not relevant to the choice of reflections for refinement. R-factors based on F^2 are statistically about twice as large as those based on F, and R-factors based on ALL data will be even larger.

Comparison of crystallographic data

Crystallographic data as bond lengths and angles is commonly given, as the value $t_i(\delta_i)$ were t_i is a scalar with a certain uncertainty, δ_i. Must a number of crystallographic data be compared; it may be advantageous to do this using a simple arithmetic mean. This introduces, however, some problems in determination of the uncertainty of the geometric mean that cannot be solved trivially. In this work the average values are used only for comparison and to determine any trend in the data given material. The uncertainties are therefore not of direct relevance and the geometric mean, $\langle t \rangle$ of a set of n values, $t_i(\delta_i)$ is given as:

$$\langle t \rangle = \tau(\omega) \quad \text{were} \quad \tau = \frac{1}{n}\sum_{i=1}^{n} t_i \quad \text{and} \quad \omega = \frac{1}{n}\sum_{i=1}^{n} \delta_i \tag{6.19}$$

The average uncertainty, ω is in contrast to the average scalar, τ not exact - but in the context of use, it still indicates a certain tendency.

Molecular graphics and presentation

The graphic representation of molecules and asymmetric units as well as representation of hydrogen bonding geometry in connection with single crystal X-ray diffraction has been performed by use of "*Mercury* CSD" Version 2.4" from The Cambridge Crystallographic Data Centre. This program is used to determine distances, angles and similar which are not found directly in the given CIF files.

The following applies generally, and not be indicated at the individual structure:

- Displacement ellipsoids are drawn at 50% probability
- Hydrogen atoms are drawn with arbitrary radii.

Hydrogen atoms, *e.g.* on aromatic ring systems will in some representations be omitted for clarity, this will be indicated onr each individual structure. All representations of crystallographic data in the thesis uses the color code given in Table 33 for the various elements, bonds and units cell axis.

Element		Bond	Unit cell axis
**Cr and Mn**	**C**	**General bond**	***a***
**Li and Na**	**N**	**H-bond**	***b***
**F**	**O**	**Centroid**	***c***
**Cl**			

Table 33 Colour codes used in graphical representation of the crystal structures

TABELLARIUM: SINGLE CRYSTAL X-RAY DIFFRACTION DATA

X-ray data No. 1: *catena*-[Mn(μ-F)(salen-5H)]

Molecular formula	$H_{14}C_{16}N_2O_2F_1Mn_1$
Molecular weight (g·mol^{-1})	340.237
T (K)	122(1)
Crystal system	Triclinic
Space group	P-1
a (Å)	10.0400(9)
b (Å)	15.4490(12)
c (Å)	16.0420(17)
α (°)	108.164(9)
β (°)	103.874(10)
γ (°)	101.140(7)
V (Å^3)	2196.3(3)
Z	6
F_{000}	1044
D_{cal} (Mg m^{-3})	1.543
μ (mm^{-1})	0.920
Crystal size (mm)	0.68 · 0.33 · 0.13
Colour	Brown
θ range (°)	2.18 - 40.06
h, k, l	-18→18, -27→27, -28→29
Absorb. correction T_{min}, T_{max}	0.684, 0.899
No. measured reflections	123564
No. independent reflections	23068
No. reflections with I>2σ(I)	23058
R_{int}	0.0506
Number of ref. parameters	598
$R[F^2>2\ \sigma(F^2)]$	0.0384
$wR(F^2)$	0.0815
S (Goodness of fit)	1.194
$(\Delta/\sigma)_{max}$	0.001
$\Delta\rho_{min}$, $\Delta\rho_{max}$(e Å^{-3})	–0.706, 0.914
CCDC	796782
$w = 1/[\sigma^2(F_o^2)+(0.0227P)^2+1.0599P]$, $P = (F_o^2+2F_c^2)/3$	

Table 34 X-ray data No. 1: *catena*-[Mn(μ-F)(salen-5H)]

X-ray data No. 2: *catena*-[Mn(μ-F)(salen-5F)]

Molecular formula	$H_{16}C_{16}N_2O_2F_3Mn_1$
Molecular weight (g·mol^{-1})	380.25
T (K)	122(2)
Crystal system	Triclinic
Space group	*P*-1
a (Å)	10.236(2)
b (Å)	11.598(4)
c (Å)	13.705(4)
α (°)	66.720(17)
β (°)	80.836(18)
γ (°)	84.77(2)
V (Å^3)	1474.8(7)
Z	4
F_{000}	776
D_{cal} (Mg m^{-3})	1.713
μ (mm^{-1})	0.942
Crystal size (mm)	
Colour	Brown
θ range (°)	1.63 - 30.25
h, k, l	–14→14, –16→16, –19→19
Absorb. correction T_{min}, T_{max}	
No. measured reflections	61908
No. independent reflections	8669
No. reflections with $I>2\sigma(I)$	5441
R_{int}	0.1894
Number of ref. parameters	436
$R[F^2>2\ \sigma(F^2)]$	0.0754
$wR(F^2)$	0.1597
S (Goodness of fit)	1.082
$(\Delta/\sigma)_{max}$	<0.001
$\Delta\rho_{min}$, $\Delta\rho_{max}$(e Å^{-3})	–1.222, 1.022
CCDC	Unpublished
$w = 1/[\sigma^2(F_o^2)+(0.0182P)^2+5.6225P]$, $P = (F_o^2+2F_c^2)/3$	

Table 35 X-ray data No. 2: *catena*-[Mn(μ-F)(salen-5F)]

X-ray data No. 3: *catena*-[Mn(μ-F)(salen-5Cl)]

Molecular formula	$H_{36}C_{48}N_6O_6F_3Cl_6Mn_3 \cdot 2(CH_4O) \cdot CH_2O$
Molecular weight (g·mol^{-1})	1321.46
T (K)	122(1)
Crystal system	Triclinic
Space group	P-1
a (Å)	13.0310(11)
b (Å)	13.5910(12)
c (Å)	16.8360(12)
α (°)	75.628(7)
β (°)	70.948(8)
γ (°)	83.250(8)
V (Å^3)	2728.0(4)
Z	2
F_{000}	1340
D_{cal} (Mg m^{-3})	1.610
μ (mm^{-1})	1.05
Crystal size (mm)	0.45 × 0.21 × 0.13
Colour	Brown
θ range (°)	1.3 – 30.0
h, k, l	–18→18, –19→19, –23→23
Absorb. correction T_{min}, T_{max}	0.663, 0.915
No. measured reflections	115160
No. independent reflections	15925
No. reflections with I>2σ(I)	12910
R_{int}	0.076
Number of ref. parameters	715
$R[F^2>2\ \sigma(F^2)]$	0.037
$wR(F^2)$	0.098
S (Goodness of fit)	1.08
$(\Delta/\sigma)_{max}$	0.001
$\Delta\rho_{min}$, $\Delta\rho_{max}$(e Å^{-3})	−0.82, 0.74
CCDC	Unpublished
$w = 1/[\sigma^2(F_o^2)+(0.032P)^2+2.7995P]$, $P = (F_o^2+2F_c^2)/3$	

Table 36 X-ray data No. 3: *catena*- [Mn(μ-F)(salen-5Cl)]

X-ray data No. 4: *trans-catena*-poly[$Na(H_2O)_4$(μ-F)Cr(py)$_4$(μ-F)]$(HCO_3)_2$

Molecular formula	$H_{30}C_{22}N_4O_{10}F_2Na_1Cr_1$
Molecular weight ($g \cdot mol^{-1}$)	623.485
T (K)	122(1)
Crystal system	Tetragonal
Space group	$P4/ncc$
a (Å)	12.5740(19)
b (Å)	12.5740(19)
c (Å)	17.271(3)
α (°)	90.000(9)
β (°)	90.000(10)
γ (°)	90.000(7)
V (Å^3)	2730.6(6)
Z	4
F_{000}	1292
D_{cal} (Mg m^{-3})	1.517
μ (mm^{-1})	0.509
Crystal size (mm)	0.46 · 0.23 · 0.20
Colour	Pink
θ range (°)	2.9 - 45.6
h, k, l	–20→25, –25→25, –34→30
Absorb. correction T_{min}, T_{max}	0.858, 0.919
No. measured reflections	147452
No. independent reflections	5784
No. reflections with I>2σ(I)	4307
R_{int}	0.0639
Number of ref. parameters	91
$R[F^2>2\ \sigma(F^2)]$	0.0330
$wR(F^2)$	0.0892
S (Goodness of fit)	1.038
$(\Delta/\sigma)_{max}$	<0.001
$\Delta\rho_{min}$, $\Delta\rho_{max}$(e Å^{-3})	–0.805, 1.047
CCDC	767058

$w = 1/[\sigma^2(F_o^2)+(0.03868P)^2 +0.7578P]$, $P = (F_o^2+2F_c^2)/3$

Table 37 X-ray data No. 4: *trans-catena*-poly[$Na(H_2O)_4$(μ-F)Cr(py)$_4$(μ-F)]$(HCO_3)_2$

X-ray data No. 5: *trans*-[Cr(py)$_4$F(μ-F){Li(H$_2$O)$_{n=3,4}$}$_{m=0,1}$]Cl$_5$·6H$_2$O

Molecular formula	$H_{86}C_{60}N_{12}O_{13}F_6Cl_5Li_2Cr_3$
Molecular weight (g·mol^{-1})	1644.585
T (K)	122(1)
Crystal system	Orthorhombic
Space group	$Pna2_1$
a (Å)	17.8140 (15)
b (Å)	9.036 (4)
c (Å)	47.539 (5)
α (°)	90.00
β (°)	90.00
γ (°)	90.00
V (Å^3)	7652 (3)
Z	12
F_{000}	3660
D_{cal} (Mg m^{-3})	1.548
μ (mm^{-1})	0.721
Crystal size (mm)	0.34 · 0.31 ·0.24
Colour, shape	Pink
θ range (°)	1.71 - 30.07
h, k, l	–25→25, –12→11, –66→66
Absorb. Correction T_{min}, T_{max}	0.820, 0.886
No. Measured reflections	69341
No. independent reflections	19719
No. reflections with I>2σ(I)	14647
R_{int}	0.0385
Number of ref. parameters	910
R[F^2>2 σ(F^2)]	0.0463
wR(F^2)	0.1171
S (Goodness of fit)	1.006
$(\Delta/\sigma)_{max}$	0.050
$\Delta\rho_{min}$, $\Delta\rho_{max}$(e Å^{-3})	–0.761, 0.966
CCDC	767059
$w = 1/[\sigma^2(F_o^2)+(0.0581P)^2]$, $P = (F_o^2+2F_c^2)/3$	

Table 38 X-ray data No. 5: *trans*-[Cr(py)$_4$F(μ-F){Li(H$_2$O)$_{n=3,4}$}$_{m=0,1}$]Cl$_5$·6H$_2$O

X-ray data No. 6: *cis*-$[Cr(phen)_2F_2]ClO_4{\cdot}H_2O$

Molecular formula	$H_{18}C_{24}N_4O_5F_2Cl_1Cr_1$
Molecular weight ($g{\cdot}mol^{-1}$)	567.87
T (K)	122(1)
Crystal system	Triclinic
Space group	P-1
a (Å)	7.6930 (10)
b (Å)	9.4640 (8)
c (Å)	16.0610(17)
α (°)	79.750 (7)
β (°)	83.228 (12)
γ (°)	88.115 (8)
V (Å^3)	1142.6 (2)
Z	2
F_{000}	578
D_{cal} (Mg m^{-3})	1.651
μ (mm^{-1})	0.681
Crystal size (mm)	0.44 · 0.41 · 0.16
Colour	Red
θ range (°)	2.34 - 25.04
h, k, l	$-9\rightarrow9$, $-11\rightarrow11$, $-18\rightarrow19$
Absorb. correction T_{min}, T_{max}	0.794, 0.913
No. measured reflections	28606
No. independent reflections	4014
No. reflections with I>2σ(I)	3851
R_{int}	0.0249
Number of ref. Parameters	329
$R[F^2>2\ \sigma(F^2)]$	0.0310
$wR(F^2)$	0.1454
S (Goodness of fit)	1.410
$(\Delta/\sigma)_{max}$	0.078
$\Delta\rho_{min}$, $\Delta\rho_{max}$(e Å^{-3})	–0.513, 0.791
CCDC	
$w = 1/[\sigma^2(F_o^2)+(0.1P)^2]$, $P = (F_o^2+2F_c^2)/3$	

Table 39 X-ray data No. 6: *cis*-$[Cr(phen)_2F_2]ClO_4{\cdot}H_2O$

X-ray data No. 7: *cis*-[Cr(bpy)$_2$(H$_2$O)(F)](ClO$_4$)$_2$·2H$_2$O

Molecular formula	$H_{22}C_{20}N_4O_{11}F_1Cl_2Cr_1$
Molecular weight (g·mol^{-1})	636.31
T (K)	122(1)
Crystal system	Triclinic
Space group	P-1
a (Å)	9.5770(10)
b (Å)	11.4050(6)
c (Å)	11.8150(11)
α (°)	77.273(6)
β (°)	79.427(9)
γ (°)	83.590(5)
V (Å^3)	1234.01(18)
Z	2
F_{000}	650
D_{cal} (Mg m^{-3})	1.713
μ (mm^{-1})	0.756
Crystal size (mm)	0.41 · 0.24 · 0.14
Colour	Orange
θ range (°)	1.79 - 27.58
h, k, l	–12→12, –14→11, –15→15
Absorb. correction T_{min}, T_{max}	0.805, 0.925
No. measured reflections	27824
No. independent reflections	5691
No. reflections with I>2σ(I)	5244
R_{int}	0.0210
Number of ref. parameters	352
$R[F^2>2\sigma(F^2)]$	0.0257
$wR(F^2)$	0.0703
S (Goodness of fit)	1.031
$(\Delta/\sigma)_{max}$	0.001
$\Delta\rho_{min}$, $\Delta\rho_{max}$(e Å^{-3})	–0.424, 0.554
CCDC	
$w = 1/[\sigma^2(F_o^2)+(0.0293P)^2+1.0694P]$, $P = (F_o^2+2F_c^2)/3$	

Table 40 X-ray data No. 7: *cis*-[Cr(bpy)$_2$(F)(H$_2$O)](ClO$_4$)$_2$·2H$_2$O

X-ray data No. 8: *cyclo*-[$(NO_3)_4$Nd(μ-F)Cr$(phen)_2$(μ-F)$]_2$

Molecular formula	$H_{50}C_{52}N_{16}O_{29}F_4Cr_2Nd_2$
Molecular weight (g·mol^{-1})	1831.530
T (K)	100(2)
Crystal system	Tetragonal
Space group	$P4/ncc$
a (Å)	17.583(2)
b (Å)	17.583(2)
c (Å)	20.881(3)
α (°)	90.00
β (°)	90.00
γ (°)	90.00
V (Å^3)	6255.6(14)
Z	4
F_{000}	3640
D_{cal} (Mg m^{-3})	1.884
μ (mm^{-1})	2.023
Crystal size (mm)	0.08 · 0.05 · 0.04
Colour	Red
θ range (°)	2.77 - 36.51
h, k, l	–30→30, –30→30, –35→35
Absorb. correction T_{min}, T_{max}	0.8760, 0.9239
No. measured reflections	462151
No. independent reflections	8486
No. reflections with I>2σ(I)	6533
R_{int}	0.0430
Number of ref. parameters	256
$R[F^2>2\sigma(F^2)]$	0.0346
$wR(F^2)$	0.1031
S (Goodness of fit)	1.320
$(\Delta/\sigma)_{max}$	0.006
$\Delta\rho_{min}$, $\Delta\rho_{max}$(e Å^{-3})	–1.138, 2.354
CCDC	
$w = 1/[\sigma^2(F_o^2)+(0.0232P)^2+18.1457P]$, $P = (F_o^2+2F_c^2)/3$	

Table 41 X-ray data No. 8: *cyclo*-[$(NO_3)_4$Nd(μ-F)Cr$(phen)_2$(μ-F)$]_2$

X-ray data No. 9: $[(NO_3)_4Nd(\mu\text{-}MeO)_2Cr(phen)_2]$

Molecular formula	$H_{30}C_{28}N_8O_{16}Cr_1Nd_1$
Molecular weight (g·mol^{-1})	930.84
T (K)	100(2)
Crystal system	Orthorhombic
Space group	*Pbcn*
a (Å)	20.439 (4)
b (Å)	10.379 (2)
c (Å)	17.293 (3)
α (°)	90.00
β (°)	90.00
γ (°)	90.00
V (Å^3)	3668.5(12)
Z	4
F_{000}	1864
D_{cal} (Mg m^{-3})	1.685
μ (mm^{-1})	1.777
Crystal size (mm)	0.05 · 0.05 · 0.02
Colour	Red/violet
θ range (°)	3.034 - 36.59
h, k, l	–34→34, –16→17, –28→28
Absorb. correction T_{min}, T_{max}	0.8952, 0.9411
No. measured reflections	131233
No. independent reflections	8817
No. reflections with I>2σ(I)	6849
R_{int}	0.0305
Number of ref. Parameters	274
$R[F^2>2\sigma(F^2)]$	0.0356
$wR(F^2)$	0.1034
S (Goodness of fit)	1.140
$(\Delta/\sigma)_{max}$	0.001
$\Delta\rho_{min}$, $\Delta\rho_{max}$(e Å^{-3})	–1.195 – 1.712
CCDC	
$w = 1/[\sigma^2(F_o^2)+(0.0338P)^2+6.2972P]$, $P = (F_o^2+2F_c^2)/3$	

Table 42 X-ray data No. 9: $[(NO_3)_4Nd(\mu\text{-}MeO)_2Cr(phen)_2]$

POWDER X-RAY DIFFRACTION

Powder X-ray diffraction were obtained at room temperature using a STOE Stadi-P powder diffractometer equipped with PSD-detector. Collection parameters for the Powder X-ray diffraction experiment are summarized in Table 43.

The samples are initially crushed to a fine powder using an agate mortar and mounted between two sheets of plastic film coated with a thin layer of mineral oil.

- **Data processing**
  The powder X-ray diffraction data were corrected by subtracting a background. In a few isolated cases were the diffraction data smoothed.
  Both type of correction was made with the software "*STOE Win XPOW*" Version 1.10.

- **Generation of theoretical powder diffractogram**
  The theoretical powder diffractogram was generated from the solved single crystal structure (CIF file). The software used was "*Mercury* CSD" Version 2.4" provided from The Cambridge Crystallographic Data Centre. [424-428]

		Specifications
Data collection	Radiation type	Cu
	Radiation wavelength, λ	1. 54060 Å
	Generator	40 kV, 40 mA
	Monochromator	Curved germanium (*111*)
	2θ	0-50 °
	Step	0.1
	Step time	50

Table 43 Collection parameters for Powder X-ray diffraction

BIBLIOGRAPHY

References in the text are given by number in squared braked [–]. The references in the bibliography are organized as follows (Minor deviations in the organization, occurs in references to monographs, dissertations, etc.):

- Family name,
- First name initials:
- Title.
- *Journal*,
- **Year**,
- Volume(Issue),
- Start page – End page

The name of the journal, without the use of abbreviations is listed in its full length (*e.g. The Chemical Educator* versus *Chem. Educator*).

Example of references: Birk, T.: Matrix Formulation of Complex Equilibria and Acid-Base Equilibria by use of the Extent of Reaction. *The Chemical Educator*, **2009**, 14(3), 91-95.

In general, a reference relating to a homepages on the internet is attempted avoided due to problematic accessibility over time. In cases were at homepage references is used are they marked with time for access. As an example of a homepage used in the treatise is "*IUPAC. Compendium of Chemical Terminology*." 2nd ed also known as the "*Gold Book*".

Some journal has over time changed name and content, or merged with other journals. References is based on the journal name it had at the time the reference was published. *e.g.* The journal of The Deutsche Chemische Gesellschaft has changed names several times since its founding . 1868-1928: "*Berichte der Deutschen Chemischen Gesellschaft*" (*Ber. Dtsch. Chem. Ges.*), 1947-1996: "*Chemische Berichte*" (*Chem. Ber.*) and now (1998-) the journal can found under the name "*European Journal of Inorganic Chemistry*" (*Eur. J. Inorg. Chem.*).

[1] Kitagawa, S.; Noro, S., 7.5 - Coordination Polymers: Infinite Systems. In *Comprehensive Coordination Chemistry II*, McCleverty, J. A.; Meyer, T. J., Eds. Pergamon: Oxford, 2003; pp 231-261.

[2] Rosseinsky, M. J.: Recent developments in metal–organic framework chemistry: design, discovery, permanent porosity and flexibility. *Microporous and Mesoporous Materials*, **2004,** 73(1–2), 15-30.

[3] Rosi, N. L.; Eckert, J.; Eddaoudi, M.; Vodak, D. T.; Kim, J.; O'Keeffe, M.; Yaghi, O. M.: Hydrogen Storage in Microporous Metal-Organic Frameworks. *Science*, **2003,** 300(5622), 1127-1129.

[4] Glerup, J.; Moensted, O.; Schaeffer, C. E.: Nonadditive and additive ligand fields and spectrochemical series arising from ligand field parameterization schemes. Pyridine as a nonlinearly ligating π-back-bonding ligand toward chromium(III). *Inorganic Chemistry*, **1976,** 15(6), 1399-1407.

[5] Bendix, J.; Bøgevig, A.: Synthesis and Characterization of a Stable *trans*-Dioxo Tungsten(IV) Complex and Series of Mono-Oxo Molybdenum(IV) and Tungsten(IV) Complexes. Structural and Electronic Effects of π-Bonding in trans-$[M(O)(X)(dppe)_2]^{+/0}$ Systems. *Inorganic Chemistry*, **1998,** 37(23), 5992-6001.

[6] Glerup, J.; Josephsen, J.; Michelsen, K.; Pedersen, E.; Schaeffer, C. E.: Preparation of chromium(III) complexes with two fluorine atoms and four nitrogen atoms as ligands. *trans*-Difluorotetrakis(pyridine)chromium(III) salts as starting materials. *Acta Chemica Scandinavica* **1970,** 24(1), 247-254.

[7] Glerup, J.; Schaffer, C. E.; Springborg, J.: Synthesis of *trans*-dichloro- and *trans*-difluorotetrakis(pyridine)cobalt(III) salts. *Acta Chemica Scandinavica Ser. A*, **1978,** A32(7), 673-674.

[8] van Slageren, J.; Sessoli, R.; Gatteschi, D.; Smith, A. A.; Helliwell, M.; Winpenny, R. E. P.; Cornia, A.; Barra, A.-L.; Jansen, A. G. M.; Rentschler, E.; Timco, G. A.: Magnetic Anisotropy of the Antiferromagnetic Ring $[Cr_8F_8Piv_{16}]$. *Chemistry – A European Journal*, **2002,** 8(1), 277-285.

[9] Larsen, F. K.; McInnes, E. J. L.; Mkami, H. E.; Overgaard, J.; Piligkos, S.; Rajaraman, G.; Rentschler, E.; Smith, A. A.; Smith, G. M.; Boote, V.; Jennings, M.; Timco, G. A.;

Winpenny, R. E. P.: Synthesis and Characterization of Heterometallic {Cr_7M} Wheels. *Angewandte Chemie International Edition*, **2003,** 42(1), 101-105.

[10] Larsen, F. K.; Overgaard, J.; Parsons, S.; Rentschler, E.; Smith, A. A.; Timco, G. A.; Winpenny, R. E. P.: Horseshoes, Rings and Distorted Rings: Studies of Cyclic Chromium-Fluoride Cages. *Angewandte Chemie International Edition*, **2003,** 42(48), 5978-5981.

[11] Pearson, R. G.: Hard and Soft Acids and Bases. *Journal of the American Chemical Society*, **1963,** 85(22), 3533-3539.

[12] Pearson, R. G.: Hard and soft acids and bases, HSAB, part 1: Fundamental principles. *Journal of Chemical Education*, **1968,** 45(9), 581-587.

[13] Pearson, R. G., *Hard and soft acids and bases*. 1 ed.; Dowden, Hutchinson and Ross, Inc.: Stroudsburg, Pennsylvania, 1973.

[14] Pevec, A.; Mrak, M.; Demsar, A.; Petricek, S.; Roesky, H. W.: Coordination number 12 in praseodymium and 11 in neodymium complexes with organofluorotitanate ligands. *Polyhedron*, **2003,** 22(4), 575-579.

[15] Perdih, F.; Demsar, A.; Pevec, A.; Petricek, S.; Leban, I.; Giester, G.; Sieler, J.; Roesky, H. W.: Synthesis and the crystal structures of a monoanionic tetrafluorodentate ligand and its complex with lanthanum ion. *Polyhedron*, **2001,** 20(15-16), 1967-1971.

[16] McRobbie, A.; Sarwar, A. R.; Yeninas, S.; Nowell, H.; Baker, M. L.; Allan, D.; Luban, M.; Muryn, C. A.; Pritchard, R. G.; Prozorov, R.; Timco, G. A.; Tuna, F.; Whitehead, G. F. S.; Winpenny, R. E. P.: Chromium chains as polydentate fluoride ligands for lanthanides. *Chemical Communications*, **2011,** 47(22), 6251-6253.

[17] Corey, E. J.: General methods for the construction of complex molecules. *Pure and Applied Chemistry* **1967,** 14(1), 19-38.

[18] Geoffrey Wilkinson, G.; Gillard, R. D.; McCleverty, J. A., *Comprehensive Coordination Chemistry. The Synthesis, Reactions, Properties & Applications of Coordination Compounds*. Pergamon Press: New York, 1987.

[19] Greenwood, N. N.; Earnshaw, A., *Chemistry of the Elements*. 2 ed.; Butterwort-Heinemann: Oxford, 2001.

[20] Cotton, F. A.; Wilkinson, G.; Murillo, C. A.; Bochmann, M., *Advanced Inorganic Chemistry* 6ed.; Wiley-Interscience: New York, 1999.

[21] Emeléus, H. J., *The chemistry of fluorine and its compounds*. 1 ed.; Academic Press: New York-London, 1969.

[22] Politzer, P.: Anomalous properties of fluorine. *Journal of the American Chemical Society*, **1969,** 91(23), 6235-6237.

[23] Lide, D. R.; Frederikse, H. P. R., *CRC Handbook of Chemistry and Physics*. 78 ed.; CRC Press: New York, 1997-1998.

[24] Khriachtchev, L.; Pettersson, M.; Runeberg, N.; Lundell, J.; Rasanen, M.: A stable argon compound. *Nature*, **2000,** 406(6798), 874-876.

[25] Smith, D. W.: The antibonding effect. *J. Chem. Educ.*, **2000,** 77(6), 780-784.

[26] Caldow, G. L.; Coulson, C. A.: Bond energies of halogen molecules. *Transactions of the Faraday Society*, **1962,** 58, 633-641.

[27] Mulliken, R. S.: Structures of the Halogen Molecules and the Strength of Single Bonds1. *Journal of the American Chemical Society*, **1955,** 77(4), 884-887.

[28] Pauling, L., *The Nature of the Chemical Bond and the Structure of Moleculaes and Crystals: An Introduction to Modern Structural Chemistry*. 3. edition ed.; Cornell University Press: Ithaca, New York, 1960.

[29] Mulliken, R. S.: A New Electroaffinity Scale; Together with Data on Valence States and on Valence Ionization Potentials and Electron Affinities. *The Journal of Chemical Physics*, **1934,** 2(11), 782-793.

[30] Bratsch, S. G.: Revised Mulliken electronegativities: I. Calculation and conversion to Pauling units. *Journal of Chemical Education*, **1988,** 65(1), 34-null.

[31] Parr, R. G.; Donnelly, R. A.; Levy, M.; Palke, W. E.: Electronegativity: The density functional viewpoint. *The Journal of Chemical Physics*, **1978,** 68(8), 3801-3807.

[32] Geerlings, P.; De Proft, F.; Langenaeker, W.: Conceptual Density Functional Theory. *Chemical Reviews*, **2003,** 103(5), 1793-1874.

[33] Pearson, R.: Chemical hardness and density functional theory. *Journal of Chemical Sciences*, **2005,** 117(5), 369-377.

[34] Parr, R. G.; Pearson, R. G.: Absolute hardness: companion parameter to absolute electronegativity. *Journal of the American Chemical Society*, **1983,** 105(26), 7512-7516.

[35] Roy, R.; Chandra, A. K.; Pal, S.: Correlation of Polarizability, Hardness, and Electronegativity: Polyatomic Molecules. *The Journal of Physical Chemistry*, **1994,** 98(41), 10447-10450.

[36] Ahrland, S.; Chatt, J.; Davies, N. R.: The relative affinities of ligand atoms for acceptor molecules and ions. *Quarterly Reviews, Chemical Society*, **1958,** 12(3).

[37] Ayers, P. W.: An elementary derivation of the hard/soft-acid/base principle. *The Journal of Chemical Physics*, **2005,** 122(14), 141102-141103.

[38] Ayers, P. W.; Parr, R. G.; Pearson, R. G.: Elucidating the hard/soft acid/base principle: A perspective based on half-reactions. *The Journal of Chemical Physics*, **2006,** 124(19), 194107-194108.

[39] Jørgensen, C. K., *Inorganic Complexes*. Academic Press: London, 1963.

[40] Jones, M. M.; Clark, H. R.: The hard and soft acid-base principle and metal ion assisted ligand substitution processes. *Journal of Inorganic and Nuclear Chemistry*, **1971,** 33(2), 413-419.

[41] Huheey, J. E.; Keiter, E. A.; Keiter, R. L., *Inorganic Chemistry. Principles of Structure and Reactivity*. 4 ed.; HarperCollins College Publishers: New York, 1993.

[42] Glerup, J.; Monsted, O.; Schaeffer, C., E.: Transferability of ligand field parameters and nonlinear ligation in chromium(III) complexes. *Inorganic Chemistry*, **1980,** 19(9), 2855-2857.

[43] Wells, A. F., *Structural Inorganic Chemistry*. 5 ed.; Oxford University Press: 1984.

[44] Manson, J. L.; Lapidus, S. H.; Stephens, P. W.; Peterson, P. K.; Carreiro, K. E.; Southerland, H. I.; Lancaster, T.; Blundell, S. J.; Steele, A. J.; Goddard, P. A.; Pratt, F. L.; Singleton, J.; Kohama, Y.; McDonald, R. D.; Sesto, R. E. D.; Smith, N. A.; Bendix, J.; Zvyagin, S. A.; Kang, J.; Lee, C.; Whangbo, M.-H.; Zapf, V. S.; Plonczak, A.: Structural, Electronic, and Magnetic Properties of Quasi-1D Quantum Magnets $[Ni(HF_2)(pyz)_2]X$ (pyz = pyrazine; X = PF_6^-, SbF_6^-). Exhibiting Ni-FHF-Ni and Ni-pyz-Ni Spin Interactions. *Inorganic Chemistry*, **2011,** 50(13), 5990-6009.

[45] Brown, S.; Cao, J.; Musfeldt, J. L.; Conner, M. M.; McConnell, A. C.; Southerland, H. I.; Manson, J. L.; Schlueter, J. A.; Phillips, M. D.; Turnbull, M. M.; Landee, C. P.: Hydrogen Bonding and Multiphonon Structure in Copper Pyrazine Coordination Polymers. *Inorganic Chemistry*, **2007,** 46(21), 8577-8583.

[46] Manson, J. L.; Schlueter, J. A.; Funk, K. A.; Southerland, H. I.; Twamley, B.; Lancaster, T.; Blundell, S. J.; Baker, P. J.; Pratt, F. L.; Singleton, J.; McDonald, R. D.; Goddard, P. A.; Sengupta, P.; Batista, C. D.; Ding, L.; Lee, C.; Whangbo, M.-H.; Franke, I.; Cox, S.; Baines, C.; Trial, D.: Strong H···F Hydrogen Bonds as Synthons in Polymeric Quantum Magnets: Structural, Magnetic, and Theoretical Characterization of $[Cu(HF_2)(pyrazine)_2]SbF_6$, $[Cu_2F(HF)(HF_2)(pyrazine)_4](SbF_6)_2$, and $[CuAg(H_3F_4)(pyrazine)_5](SbF_6)_2$. *Journal of the American Chemical Society*, **2009,** 131(19), 6733-6747.

[47] Čižmár, E.; Zvyagin, S. A.; Beyer, R.; Uhlarz, M.; Ozerov, M.; Skourski, Y.; Manson, J. L.; Schlueter, J. A.; Wosnitza, J.: Magnetic properties of the quasi-two-dimensional S=1/2 Heisenberg antiferromagnet $[Cu(pyz)_2(HF_2)]PF_6$. *Physical Review B*, **2010,** 81(6), 064422.

[48] Sengupta, P.; Batista, C. D.; McDonald, R. D.; Cox, S.; Singleton, J.; Huang, L.; Papageorgiou, T. P.; Ignatchik, O.; Herrmannsdörfer, T.; Manson, J. L.; Schlueter, J. A.; Funk, K. A.; Wosnitza, J.: Nonmonotonic field dependence of the Néel temperature in the quasi-two-dimensional magnet $[Cu(HF_2)(pyz)_2]BF_4$. *Physical Review B*, **2009,** 79(6), 060409.

[49] Musfeldt, J. L.; Vergara, L. I.; Brinzari, T. V.; Lee, C.; Tung, L. C.; Kang, J.; Wang, Y. J.; Schlueter, J. A.; Manson, J. L.; Whangbo, M. H.: Magnetoelastic Coupling through the Antiferromagnet-to-Ferromagnet Transition of Quasi-Two-Dimensional $[Cu(HF_2)(pyz)_2]BF_4$ Using Infrared Spectroscopy. *Physical Review Letters*, **2009,** 103(15), 157401.

[50] Manson, J.; Schlueter, J.; McDonald, R.; Singleton, J.: Crystal Structure and Antiferromagnetic Ordering of Quasi-2D $[Cu(HF_2)(pyz)_2]TaF_6$ (pyz=pyrazine). *Journal of Low Temperature Physics*, **2010,** 159(1), 15-19.

[51] Manson, J. L.; Conner, M. M.; Schlueter, J. A.; Lancaster, T.; Blundell, S. J.; Brooks, M. L.; Pratt, F. L.; Papageorgiou, T.; Bianchi, A. D.; Wosnitza, J.; Whangbo, M.-H.:

$[Cu(HF_2)(pyz)_2]BF_4$ (pyz = pyrazine): long-range magnetic ordering in a pseudo-cubic coordination polymer comprised of bridging HF_2^- and pyrazine ligands. *Chemical Communications*, **2006,** (47), 4894-4896.

[52] Deshpande, V. T.: Thermal expansion of sodium fluoride and sodium bromide. *Acta Crystallographica*, **1961,** 14, 794-794.

[53] Wyckoff, R. W. G., *Fluorite structure*. Second ed.; Interscience Publishers: New York, 1963.

[54] Afanasiev, M. L.; Habuda, S. P.; Lundin, A. G.: The symmetry and basic structures of LaF_3, CeF_3, PrF_3 and NdF_3. *Acta Crystallographica Section B*, **1972,** 28(10), 2903-2905.

[55] Wang, Q.-M.; Mak, T. C. W.: Novel layer-type triple salts of silver(I), AgCN AgF $4AgCF_3CO_2$ 2L (L = MeCN or H_2O). *Chemical Communications*, **2000,** (15), 1435-1436.

[56] Donath, H.; Avtomonov, E. V.; Sarraje, I.; von Dahlen, K. H.; El-Essawi, M.; Lorberth, J.; Seo, B. S.: Organoplatinum compounds VII: Trimethylplatinum fluoride $[(CH_3)_3PtF]_4$, the missing link in organoplatinum cluster chemistry: its synthesis, crystal structure and a comparison to the crystal structure of $[(CH_3)_3PtOH]_4$. *Journal of Organometallic Chemistry*, **1998,** 559(1-2), 191-196.

[57] Tereshchenko, D. S.; Morozov, I. V.; Boltalin, A. I.; Kemnitz, E.; Troyanov, S. I.: Trinuclear Co(II) and Ni(II) Complexes with Tridentate Fluorine, $[M_3(\mu_3\text{-}F)(CF_3COO)_6(CF_3COOH)_3]^-$: Synthesis and Crystal Structure *Russian Journal of Inorganic Chemistry (Zhurnal Neorganicheskoi Khimii)*, **2004** 49(6), 836.

[58] Buchholz, N.; Mattes, R.: $(Me_4N)_3[Mo_4O_{12}F_3]\cdot0.8H_2O$, A Salt with a Novel Fluorooxo Polyanion. *Angewandte Chemie International Edition in English*, **1986,** 25(12), 1104-1105.

[59] Stalke, D.; Liu, F.-Q.; Roesky, H. W.: Synthesis and x-ray crystal structure of an asymmetric mixed metal $[\{(\eta^5\text{-}C_5H_4SiMe_3)TiF_2\}_5AlF_3(THF)]$ complex containing an $AlTi_5F_{13}$ core. *Polyhedron*, **1996,** 15(17), 2841-2843.

[60] Crabtree, R. H.; Hlatky, G. G.; Holt, E. M.: Bidodecahedral coordination geometry in a dimolybdenum tetrahydride containing three fluoride bridges: crystal and molecular

structure of μ-trifluoro-bis[tris(methyldiphenylphosphine)dihydromolybdenum] tetrafluoroborate ([((PMePh$_2$)$_3$H$_2$Mo)$_2$(μ-F)$_3$]BF$_4$). *Journal of the American Chemical Society*, **1983,** 105(25), 7302-7306.

[61] Dawson, D. M.; Henderson, R. A.; Hills, A.; Hughes, D. L.: Preparation of dimolybdenum carbonyl and ditungsten carbonyl complexes containing triple fluoro bridges. *Journal of the Chemical Society, Dalton Transactions: Inorganic Chemistry* **1992,** (6), 973-976.

[62] Kiriazis, L.; Mattes, R.: Mono- and dinuclear fluoro complexes of titanium(III), chromium(III), and iron(III). Syntheses and structures of (NMe$_4$)(Ti(H$_2$O)$_4$F$_2$)TiF$_6$·H$_2$O, (NMe$_4$)$_3$Cr$_2$F$_9$, and (NMe$_4$)$_3$Fe$_2$F$_9$. *Zeitschrift fuer Anorganische und Allgemeine Chemie*, **1991,** 593, 90-98.

[63] Krämer, K. W.; Schenker, R.; Hauser, J.; Weihe, H.; Güdel, H. U.; Bürgi, H.-B.: Crystal Structures of (Et$_4$N)$_3$M$_2$F$_9$ (M=V, Cr, Fe) determined by X-Ray Single-Crystal and Powder Diffraction: A New Structure Type for A$_3$M$_2$X$_9$ Compounds. *Zeitschrift für anorganische und allgemeine Chemie*, **2001,** 627(11), 2511-2516.

[64] Figgis, B. N.; Hitchman, M. A., *Ligand field theory and its applications*. Wiley-VCH: New York :, 2000.

[65] Jørgensen, C. K., *Absorption Spectra and Chemical Bonding in Complexes*. 1 ed.; Pergamon Press: Oxford-London-New York-Paris, 1962.

[66] Bendix, J. Aspects of Strong π-Bonding in Systems with Partly Filled d-Shells. University of Copenhagen, Copenhagen, 1998.

[67] Lever, A. B. P., *Inorganic Electronic Spectroscopy*. Elsevier: Amsterdam, 1984.

[68] Müller, A.; Diemann, E.; Jørgensen, C., Electronic spectra of tetrahedral oxo, thio and seleno complexes formed by elements of the beginning of the transition groups. In *Structure & Bonding*, Springer: Berlin / Heidelberg, 1973; Vol. 14, pp 23-47.

[69] Cornia, A.; Mannini, M.; Sainctavit, P.; Sessoli, R.: Chemical strategies and characterization tools for the organization of single molecule magnets on surfaces. *Chemical Society Reviews*, **2011,** 40(6), 3076-3091.

[70] Dechambenoit, P.; Long, J. R.: Microporous magnets. *Chemical Society Reviews*, **2011,** 40(6), 3249-3265.

[71] Miller, J. S.: Magnetically ordered molecule-based materials. *Chemical Society Reviews*, **2011,** 40(6), 3266-3296.

[72] Miller, J. S.; Gatteschi, D.: Molecule-based magnets. *Chemical Society Reviews*, **2011,** 40(6), 3065-3066.

[73] Nakano, M.; Oshio, H.: Magnetic anisotropies in paramagnetic polynuclear metal complexes. *Chemical Society Reviews*, **2011,** 40(6), 3239-3248.

[74] Novoa, J. J.; Deumal, M.; Jornet-Somoza, J.: Calculation of microscopic exchange interactions and modelling of macroscopic magnetic properties in molecule-based magnets. *Chemical Society Reviews*, **2011,** 40(6), 3182-3212.

[75] Sanvito, S.: Molecular spintronics. *Chemical Society Reviews*, **2011,** 40(6), 3336-3355.

[76] Sorace, L.; Benelli, C.; Gatteschi, D.: Lanthanides in molecular magnetism: old tools in a new field. *Chemical Society Reviews*, **2011,** 40(6), 3092-3104.

[77] Talham, D. R.; Meisel, M. W.: Thin films of coordination polymer magnets. *Chemical Society Reviews*, **2011,** 40(6), 3356-3365.

[78] Train, C.; Gruselle, M.; Verdaguer, M.: The fruitful introduction of chirality and control of absolute configurations in molecular magnets. *Chemical Society Reviews*, **2011,** 40(6), 3297-3312.

[79] Troiani, F.; Affronte, M.: Molecular spins for quantum information technologies. *Chemical Society Reviews*, **2011,** 40(6), 3119-3129.

[80] Wang, X.-Y.; Avendano, C.; Dunbar, K. R.: Molecular magnetic materials based on 4*d* and 5*d* transition metals. *Chemical Society Reviews*, **2011,** 40(6), 3213-3238.

[81] Weng, D.-F.; Wang, Z.-M.; Gao, S.: Framework-structured weak ferromagnets. *Chemical Society Reviews*, **2011,** 40(6), 3157-3181.

[82] Casellas, H.; Pevec, A.; Kozlevcar, B.; Gamez, P.; Reedijk, J.: An unprecedented μ_4-SiF_6^{2-} – bridged supramolecular polymer consisting of bis-μ-F^- – bridged dinuclear Cu(II) dications. *Polyhedron*, **2005,** 24(12), 1549-1554.

[83] Kahn, O., *Molecular Magnetisme*. VCH Publishers, Inc.: New York, Weinheim, Cambridge, 1993.

[84] Anderson, P. W.: New Approach to the Theory of Superexchange Interactions. *Physical Review*, **1959,** 115(1), 2-13.

[85] Pebler, J.; Massa, W.; Lass, H.; Ziegler, B.: Intrachain exchange energies in 1-dimensional magnetic fluoromanganates(III) as a function of Mn–F–Mn bridge angle and crystal structure of Li_2MnF_5. *Journal of Solid State Chemistry*, **1987,** 71(1), 87-94.

[86] Velthuizen, W. C.; Haasnoot, J. G.; Kinneging, A. J.; Rietmeijer, F. J.; Reedijk, J.: Synthesis, structure, and magnetic properties of a novel difluoro-bridged copper(II) dimer with unusually strong antiferromagnetic coupling. *Journal of the Chemical Society, Chemical Communications*, **1983,** (22), 1366-1368.

[87] Oosterling, A. J.; de Graaff, R. A. G.; Haasnoot, J. G.; Keij, F. S.; Reedijk, J.; Pedersen, E.: Spectroscopic and magnetic identification of difluoro-bridged zigzag copper chains with substituted 5-phenylpyrazole ligands. Structural identification of catena-poly [bis(3-methyl-4-ethyl-5-phenylpyrazole)copper-di-μ-fluoro]. *Inorganica Chimica Acta*, **1989,** 163(1), 53-58.

[88] Rietmeijer, F. J.; De Graaff, R. A. G.; Reedijk, J.: Synthesis, structure, and magnetic properties of fluoride-bridged copper(II) dimers. Crystal and molecular structures of bis(μ-fluoro)bis[tris(3,4,5-trimethylpyrazole-N2)copper(II)] bis(tetrafluoroborate) and bis(μ-fluoro)bis[(5-methylpyrazole-N2)bis(3,5-dimethylpyrazole-N2)copper(II)] bis(tetrafluoroborate). *Inorganic Chemistry*, **1984,** 23(2), 151-156.

[89] Davies, G.: Some aspects of the chemistry of manganese(III) in aqueous solution. *Coordination Chemistry Reviews*, **1969,** 4(2), 199-224.

[90] Levason, W.; McAuliffe, C. A.: Higher oxidation state chemistry of manganese. *Coordination Chemistry Reviews*, **1972,** 7(4), 353-384.

[91] Mangan. In *Gmelins Handbuch der anorganischen Chemie*, 8 ed.; Katscher, H., Ed. Springer-Verlag: Berlin, Heidelberg, New Yourk, 1977; Vol. Teil C4 Verbindungen mit Mangans mit Fluor, pp 202-215.

[92] Mangan. In *Gmelins Handbuch der anorganischen Chemie*, 8 ed.; Köttelwesch , H., Ed. Springer-Verlag: Berlin, Heidelberg, New Yourk, 1979; Vol. Teil D1 Koordinationsverbindungen 1, pp 17-22.

[93] Lower, J. A.; Frenelius, W. C., Potassium Hexacyanomanganate(III). In *Inorganic Synthesis*, Fernelius, W. C., Ed. McGraw-Hill Book Company, Inc.: 1946; Vol. 2, p 213.

[94] Wieghardt, K.; Siebert, H.: Zur Kenntnis der Hexafluoromanganate(III). *Zeitschrift für anorganische und allgemeine Chemie*, **1971,** 381(1), 12-20.

[95] Stief, R.; Massa, W.: Jahn-Teller-Ordnung in pipzH_2[Mn_2F_8], einem Fluoromanganat(III) mit neuer Schichtstruktur. *Zeitschrift für anorganische und allgemeine Chemie*, **2006,** 632(5), 797-800.

[96] Stief, R.; Massa, W.: Fluoromanganat(III)-Anionen mit neuer tetramerer und Kettenstruktur in (pipzH_2)$_3$[Mn_4F_{18}(H_2O)]·(H_2O) und (pipzH_2)$_4$[Mn_2F_9]$_2$[MnF_4(H_2O)$_2$][MnF_4(HF)$_2$]. *Zeitschrift für anorganische und allgemeine Chemie*, **2004,** 630(13-14), 2502-2507.

[97] Christensen, O. T.: Undersøgelser over Manganforbindelser. II Manganiacetat og Manganets Aluner. *Oversigt over det Kgl. Danske Videnskabernes selskabs forhandlinger 1900*, **1900,** 6.

[98] Christensen, O. T.: Untersuchungen über Manganverbindungen. II. Manganiacetat und Alaune des Mangans. *Zeitschrift für anorganische Chemie*, **1901,** 27(1), 321-340.

[99] Biju, A. R.; Rajasekharan, M. V.: Fluoromanganese(III) complex of phenanthroline. Distortion isomers of Mn(phen)F_3(H_2O) stabilised by intermolecular interactions – Crystal structures, electronic spectra and DFT calculations. *Journal of Molecular Structure*, **2008,** 875(1–3), 456-461.

[100] Nunez, P.; Elias, C.; Fuentes, J.; Solans, X.; Tressaud, A.; Carmen Marco de Lucas, M.; Rodriguez, F.: Synthesis, structure and polarized optical spectroscopy of two new fluoromanganese(III) complexes. *Journal of the Chemical Society, Dalton Transactions*, **1997,** (22), 4335-4340.

[101] Bhattacharjee, M. N.; Chaudhuri, M. K.; Purkayastha, R. N. D.: Fluoride-assisted stabilization of manganese(III) in aqueous medium. A general approach to the synthesis of mixed-ligand fluoro complexes of manganese(III). *Inorganic Chemistry*, **1989,** 28(19), 3747-3752.

[102] Smith, R. M.; Marytell, A. E., *Critical Stability Constants*. 1 ed.; Plenum Press: New York, London, 1975; Vol. 2: Amines.

[103] Darriet, J.; Massa, W.; Pebler, J.; Stief, R.: 4,4′-bipyMnF_3, a modulated hybrid layer structure with 1D magnetic properties. *Solid State Sciences*, **2002,** 4(11–12), 1499-1508.

[104] Nunez, P.; Ruiz-Morales, J. C.; Lozano-Gorrin, A. D.; Gili, P.; Rodriguez, V. D.; Gonzalez-Platas, J.; Barriuso, T.; Rodriguez, F.: Synthesis, X-ray structure, polarized optical spectra and DFT theoretical calculations of two new organic-inorganic hybrid fluoromanganates(iii): $(bpaH_2)[MnF_4(H_2O)_2]_2$ and $(bpeH_2)[MnF_4(H_2O)_2]_2$. *Dalton Transactions*, **2004,** (2), 273-278.

[105] Mantel, C.; Hassan, A. K.; Pécaut, J.; Deronzier, A.; Collomb, M.-N.; Duboc-Toia, C.: A High-Frequency and High-Field EPR Study of New Azide and Fluoride Mononuclear Mn(III) Complexes. *Journal of the American Chemical Society*, **2003,** 125(40), 12337-12344.

[106] Leo, R.; Massa, W.: Starker Pseudo-Jahn–Teller-Effekt in der Struktur von $pyH[MnF_2(C_2O_4)(H_2O)_2]$. *Zeitschrift für anorganische und allgemeine Chemie*, **2010,** 636(9-10), 1687-1690.

[107] Stief, R.; Massa, W.: Einfluss von H-Brückenbindungen auf die Jahn–Teller-Verzerrung in den Kettenstrukturen von 2,2′-bipy$Mn(H_2PO_4)F_2 \cdot H_2O$ und 2,2′-bipy$Mn(H_2PO_4)_2F$. *Zeitschrift für anorganische und allgemeine Chemie*, **2010,** 636(15), 2617-2621.

[108] Dittrich, G.; Hoppe, R.: Zur Kristallstruktur von $LiMnO_2$. *Zeitschrift für anorganische und allgemeine Chemie*, **1969,** 368(5-6), 262-270.

[109] Scholder, R.; Kyri, H.: Über die Oxydation von Mangan(II)-hydroxyd mit Sauerstoff in konzentrierten Laugen. *Zeitschrift für anorganische und allgemeine Chemie*, **1952,** 270(1-4), 56-68.

[110] Zhao, H.; Berlinguette, C. P.; Bacsa, J.; Prosvirin, A. V.; Bera, J. K.; Tichy, S. E.; Schelter, E. J.; Dunbar, K. R.: Structural Characterization, Magnetic Properties, and Electrospray Mass Spectrometry of Two Jahn−Teller Isomers of the Single-Molecule Magnet $[Mn_{12}O_{12}(CF_3COO)_{16}(H_2O)_4]$. *Inorganic Chemistry*, **2004,** 43(4), 1359-1369.

[111] Brauer, G., *Handbook of Preparative Inorganic Chemistry*. 2 ed.; Academic Press: New York, 1963; Vol. 1.

[112] Meyer, J.; Marek, J.: Zur Kenntnis des dreiwertigen Mangans. V. *Zeitschrift für anorganische und allgemeine Chemie*, **1924,** 133(1), 325-340.

[113] Lis, T.; Matuszewski, J.: Structure of potassium tris(oxalato)manganate(III) trihydrate. *Acta Crystallographica Section B*, **1980,** 36(8), 1938-1940.

[114] Bullock, J. I.; Patel, M. M.; Salmon, J. E.: Manganese(III) complexes of the malonate ion. *Journal of Inorganic and Nuclear Chemistry*, **1969,** 31(2), 415-423.

[115] Baikie, A. R. E.; Hursthouse, M. B.; New, D. B.; Thornton, P.: Preparation, crystal structure, and magnetic properties of a trinuclear mixed-valence manganese carboxylate. *Journal of the Chemical Society, Chemical Communications*, **1978,** (2).

[116] Lis, T.: Preparation, structure, and magnetic properties of a dodecanuclear mixed-valence manganese carboxylate. *Acta Crystallographica Section B*, **1980,** 36(9), 2042-2046.

[117] Eppley, H. J.; Christou, G., Synthesis of dodecaoxohexadecacarboxylatotetraaquo-dodecanganese $[Mn_{12}O_{12}(O_2CR)_{16}(H_2O)_4]$ (R=Me, Et, Ph, Cr) Complexes. In *Inorganic Synthesis*, John Wiley & Sons, Inc. Acdemic Press, Inc.: 2002; Vol. 33, pp 61-66.

[118] Sessoli, R.; Tsai, H. L.; Schake, A. R.; Wang, S.; Vincent, J. B.; Folting, K.; Gatteschi, D.; Christou, G.; Hendrickson, D. N.: High-spin molecules: $[Mn_{12}O_{12}(O_2CR)_{16}(H_2O)_4]$. *Journal of the American Chemical Society*, **1993,** 115(5), 1804-1816.

[119] Cartledge, G. H.: Equilibrium Between the Complexes of Tervalent Manganese with 2,4-Pentanedione. *Journal of the American Chemical Society*, **1951,** 73(9), 4416-4419.

[120] Davis, T. S.; Fackler, J. P.; Weeks, M. J.: Spectra of manganese(III) complexes. Origin of the low-energy band. *Inorganic Chemistry*, **1968,** 7(10), 1994-2002.

[121] Fay, R. C.; Piper, T. S.: Coordination Compounds of Trivalent Metals with Unsymmetrical Bidentate Ligands. I. Benzoylacetonates. *Journal of the American Chemical Society*, **1962,** 84(12), 2303-2308.

[122] Fay, R. C.; Piper, T. S.: Coördination Compounds of Trivalent Metals with Unsymmetrical Bidentate Ligands. II. Trifluoroacetylacetonates. *Journal of the American Chemical Society*, **1963,** 85(5), 500-504.

[123] Barra, A.-L.; Gatteschi, D.; Sessoli, R.; Abbati, G. L.; Cornia, A.; Fabretti, A. C.; Uytterhoeven, M. G.: Electronic Structure of Manganese(III) Compounds from High-Frequency EPR Spectra. *Angewandte Chemie International Edition in English*, **1997,** 36(21), 2329-2331.

[124] Cartledge, G. H.: Bispentanedione Diaquo Manganese(III) Compounds. *Journal of the American Chemical Society*, **1952,** 74(23), 6015-6018.

[125] Romain, S.; Duboc, C.; Neese, F.; Rivière, E.; Hanton, L. R.; Blackman, A. G.; Philouze, C.; Leprêtre, J.-C.; Deronzier, A.; Collomb, M.-N.: An Unusual Stable Mononuclear Mn^{III} Bis-terpyridine Complex Exhibiting Jahn–Teller Compression: Electrochemical Synthesis, Physical Characterisation and Theoretical Study. *Chemistry – A European Journal*, **2009,** 15(4), 980-988.

[126] Anderson, P. A.; Creaser, II; Dean, C.; Harrowfield, J. M.; Horn, E.; Martin, L. L.; Sargeson, A. M.; Snow, M. R.; Tiekink, E. R. T.: Synthesis, Resolution and Kinetics of Electron Self-Exchange of High-Spin Manganese(II)/(III) Cage Complexes. *Australian Journal of Chemistry*, **1993,** 46(4), 449-463.

[127] Creaser, II; Engelhardt, L. M.; Harrowfield, J. M.; Sargeson, A. M.; Skelton, B. W.; White, A. H.: Syntheses and Structures of Manganese(II) and Manganese(III) Nitrate Diaminosarcophagine Complexes. *Australian Journal of Chemistry*, **1993,** 46(4), 465-476.

[128] Goodwin, H. A.; Sylva, R. N.: The magnetic properties of some complexes of higher-valent manganese. *Australian Journal of Chemistry*, **1967,** 20(4), 629-637.

[129] Funk, H.; Kreis, H.: Zur Kenntnis des dreiwertigen Mangans: Verbindungen des Mangan(III)-chlorids mit Aminen und einigen Äthern. *Zeitschrift für anorganische und allgemeine Chemie*, **1967,** 349(1-2), 45-49.

[130] Levason, W.; McAuliffe, C. A.: The coordination chemistry of manganese—IV. The reaction of manganese(III) chloride with group VB ligands. *Journal of Inorganic and Nuclear Chemistry*, **1975,** 37(1), 340-342.

[131] Ray, M. M.; Adhya, J. N.; Biswas, D.; Poddar, S. N.: Tris chelates of manganese(III) with picolinic acid and 8-hydroxyquinoline. *Australian Journal of Chemistry*, **1966,** 19(9), 1737-1740.

[132] Schiff, H.: Untersuchungen über Salicinderivate. *Annalen der Chemie und Pharmacie*, **1869,** 149, 193-200.

[133] Mason, A. T.: Ueber Condensationsderivate des Aethylendiamins. *Berichte der Deutschen Chemischen Gesellschaft*, **1887,** 20 (1), 267-277.

[134] Pfeiffer, P.; Breith, E.; Lübbe, E.; Tsumaki, T.: Tricyclische orthokondensierte Nebenvalenzringe. *Justus Liebigs Annalen der Chemie*, **1933,** 503, 84-130.

[135] Pfeiffer, P.; Thielert, H.: Innerkomplexe Titansalze. *Berichte der Deutschen Chemischen Gesellschaft*, **1938,** 12(1), 119-123.

[136] Pfeiffer, P.; Hesse, T.; Pfitzner, H.; Scholl, W.; Thielert, H.: Innere Komplexsalze der Aldimin- und Azoreihe. *Journal für praktische Chemie*, **1937,** 149(8-10), 215-296.

[137] Yamada, S.: Recent aspects of the stereochemistry of schiff-base-metal complexes. *Coordination Chemistry Reviews*, **1966,** 1(4), 415-437.

[138] Asmussen, R. W.; Soling, H.: The Magnetic Properties of Manganous Complexes. Studies in Magnetochemistry 18. *Acta Chemica Scandinavica* **1957,** 11(8), 1331-1339.

[139] Earnshaw, A.; King, E. A.; Larkworthy, L. F.: Transition metal-Schiff's base complexes. Part IV. Investigations of some iron(II) and manganese(II) systems. *Journal of the Chemical Society A: Inorganic, Physical, Theoretical*, **1968**, 1048-1052.

[140] Boreham, C. J.; Chiswell, B.: The multidentate chemistry of manganese(II). V. Non-charged complexes of salen-type ligands and their reaction with oxygen. *Inorganica Chimica Acta*, **1977,** 24(0), 77-83.

[141] Thielert, H.; Pfeiffer, P.: Zur Kenntnis der Luminescenzerscheinungen bei der Oxydation des Luminols. *Berichte der Deutschen Chemischen Gesellschaft*, **1938,** 7, 1399-1403.

[142] Miyasaka, H.; Saitoh, A.; Abe, S.: Magnetic assemblies based on Mn(III) salen analogues. *Coordination Chemistry Reviews*, **2007,** 251(21–24), 2622-2664.

[143] Zhang, W.; Jacobsen, E. N.: Asymmetric olefin epoxidation with sodium hypochlorite catalyzed by easily prepared chiral manganese(III) salen complexes. *The Journal of Organic Chemistry*, **1991,** 56(7), 2296-2298.

[144] Pospisil, P. J.; Carsten, D. H.; Jacobsen, E. N.: X-Ray Structural Studies of Highly Enantioselective Mn(salen) Epoxidation Catalysts. *Chemistry – A European Journal*, **1996,** 2(8), 974-980.

[145] Jacobsen, E. N.; Zhang, W.; Muci, A. R.; Ecker, J. R.; Deng, L.: Highly enantioselective epoxidation catalysts derived from 1,2-diaminocyclohexane. *Journal of the American Chemical Society*, **1991,** 113(18), 7063-7064.

[146] Brandes, B. D.; Jacobsen, E. N.: Highly Enantioselective, Catalytic Epoxidation of Trisubstituted Olefins. *The Journal of Organic Chemistry*, **1994,** 59(16), 4378-4380.

[147] Covell, D. J.; White, M. C.: A chiral Lewis acid strategy for enantioselective allylic C-H oxidation. *Angewandte Chemie International Edition*, **2008,** 47(34), 6448-6451.

[148] Doyle, A. G.; Jacobsen, E. N.: Enantioselective alkylation of acyclic α,α-disubstituted tributyltin enolates catalyzed by a {Cr(salen)} complex. *Angewandte Chemie International Edition*, **2007,** 46(20), 3701-3705.

[149] Loy, R. N.; Jacobsen, E. N.: Enantioselective Intramolecular Openings of Oxetanes Catalyzed by (salen)Co(III) Complexes: Access to Enantioenriched Tetrahydrofurans. *Journal of the American Chemical Society*, **2009,** 131(8), 2786-2787.

[150] Mazet, C.; Jacobsen, E. N.: Dinuclear {(salen)Al} complexes display expanded scope in the conjugate cyanation of α,β-unsaturated imides. *Angewandte Chemie International Edition*, **2008,** 47(9), 1762-1765.

[151] Huang, J.; Lian, B.; Yong, L.; Qian, Y.: Syntheses of zirconium (IV) complexes with Schiff-base ligand and their catalytic activities for polymerization of ethylene. *Inorganic Chemistry Communications*, **2001,** 4(8), 392-394.

[152] Yun, X.; Hu, X.; Jin, Z.; Hu, J.; Yan, C.; Yao, J.; Li, H.: Copper-salen catalysts modified by ionic compounds for the oxidation of cyclohexene by oxygen. *Journal of Molecular Catalysis A: Chemical*, **2010,** 327(1–2), 25-31.

[153] Lawrence, G. D.; Sawyer, D. T.: The chemistry of biological manganese. *Coordination Chemistry Reviews*, **1978,** 27(2), 173-193.

[154] Barber, J.; Murray, J. W.: Revealing the structure of the Mn-cluster of photosystem II by X-ray crystallography. *Coordination Chemistry Reviews*, **2008,** 252(3–4), 233-243.

[155] Cady, C. W.; Crabtree, R. H.; Brudvig, G. W.: Functional models for the oxygen-evolving complex of photosystem II. *Coordination Chemistry Reviews*, **2008,** 252(3–4), 444-455.

[156] Dau, H.; Haumann, M.: The manganese complex of photosystem II in its reaction cycle—Basic framework and possible realization at the atomic level. *Coordination Chemistry Reviews*, **2008,** 252(3–4), 273-295.

[157] Esa, T.: Photoinhibition of Photosystem II and photodamage of the oxygen evolving manganese cluster. *Coordination Chemistry Reviews*, **2008,** 252(3–4), 361-376.

[158] Herrero, C.; Lassalle-Kaiser, B.; Leibl, W.; Rutherford, A. W.; Aukauloo, A.: Artificial systems related to light driven electron transfer processes in PSII. *Coordination Chemistry Reviews*, **2008,** 252(3–4), 456-468.

[159] Mullins, C. S.; Pecoraro, V. L.: Reflections on small molecule manganese models that seek to mimic photosynthetic water oxidation chemistry. *Coordination Chemistry Reviews*, **2008,** 252(3–4), 416-443.

[160] Rappaport, F.; Diner, B. A.: Primary photochemistry and energetics leading to the oxidation of the $(Mn)_4Ca$ cluster and to the evolution of molecular oxygen in Photosystem II. *Coordination Chemistry Reviews*, **2008,** 252(3–4), 259-272.

[161] Raymond, J.; Blankenship, R. E.: The origin of the oxygen-evolving complex. *Coordination Chemistry Reviews*, **2008,** 252(3–4), 377-383.

[162] Sauer, K.; Yano, J.; Yachandra, V. K.: X-ray spectroscopy of the photosynthetic oxygen-evolving complex. *Coordination Chemistry Reviews*, **2008,** 252(3–4), 318-335.

[163] Sproviero, E. M.; Gascón, J. A.; McEvoy, J. P.; Brudvig, G. W.; Batista, V. S.: Computational studies of the O2-evolving complex of photosystem II and biomimetic oxomanganese complexes. *Coordination Chemistry Reviews*, **2008,** 252(3–4), 395-415.

[164] Doctrow, S. R.; Huffman, K.; Marcus, C. B.; Tocco, G.; Malfroy, E.; Adinolfi, C. A.; Kruk, H.; Baker, K.; Lazarowych, N.; Mascarenhas, J.; Malfroy, B.:

Salen−Manganese Complexes as Catalytic Scavengers of Hydrogen Peroxide and Cytoprotective Agents: Structure−Activity Relationship Studies. *Journal of Medicinal Chemistry*, **2002,** 45(20), 4549-4558.

[165] Lippard, S. J.; Berg, J. M., *Principles of Bioinorganic Chemistry*. 1 ed.; University Science Books: Mill Vally, California, 1994.

[166] Baudry, M.; Etienne, S.; Bruce, A.; Palucki, M.; Jacobsen, E.; Malfroy, B.: Salen-Manganese Complexes Are Superoxide Dismutase-Mimics. *Biochemical and Biophysical Research Communications*, **1993,** 192(2), 964-968.

[167] Baker, K.; Marcus, C. B.; Huffman, K.; Kruk, H.; Malfroy, B.; Doctrow, S. R.: Synthetic Combined Superoxide Dismutase/Catalase Mimetics Are Protective as a Delayed Treatment in a Rat Stroke Model: A Key Role for Reactive Oxygen Species in Ischemic Brain Injury. *Journal of Pharmacology and Experimental Therapeutics*, **1998,** 284(1), 215-221.

[168] Gonzalez, P. K.; Zhuang, J.; Doctrow, S. R.; Malfroy, B.; Benson, P. F.; Menconi, M. J.; Fink, M. P.: EUK-8, a synthetic superoxide dismutase and catalase mimetic, ameliorates acute lung injury in endotoxemic swine. *Journal of Pharmacology and Experimental Therapeutics*, **1995,** 275(2), 798-806.

[169] Ansari, K. I.; Kasiri, S.; Grant, J. D.; Mandal, S. S.: Apoptosis and anti-tumour activities of manganese(III)-salen and -salphen complexes. *Dalton Transactions*, **2009,** (40), 8525-8531.

[170] Gravert, D. J.; Griffin, J. H.: Specific DNA cleavage mediated by manganese complex $[SalenMn(III)]^+$. *The Journal of Organic Chemistry*, **1993,** 58(4), 820-822.

[171] Fucassi, F.; Lowe, J. E.; Pavey, K. D.; Shah, S.; Faragher, R. G. A.; Green, M. H. L.; Paul, F.; O'Hare, D.; Cragg, P. J.: α-Lipoic acid and glutathione protect against the prooxidant activity of SOD/catalase mimetic manganese salen derivatives. *J. Inorg. Biochem.*, **2007,** 101(Copyright (C) 2010 American Chemical Society (ACS). All Rights Reserved.), 225-232.

[172] Hargittai, M.; Réffy, B.; Kolonits, M.; Marsden, C. J.; Heully, J.-L.: The Structure of the Free MnF_3 MoleculeA Beautiful Example of the Jahn−Teller Effect. *Journal of the American Chemical Society*, **1997,** 119(38), 9042-9048.

[173] Hepworth, M. A.; Jack, K. H.: The crystal structure of manganese trifluoride, MnF_3. *Acta Crystallographica*, **1957,** 10(5), 345-351.

[174] Tregenna-Piggott, P. L. W.; Andres, H.-P.; McIntyre, G. J.; Best, S. P.; Wilson, C. C.; Cowan, J. A.: Aqua Ions. 2. Structural manifestations of the Jahn-Teller effect in the beta-alums. *Inorganic Chemistry*, **2003,** 42(4), 1350-1365.

[175] Kallies, B.; Meier, R.: Electronic structure of 3d[$M(H_2O)_6$](3+) ions from Sc(III) to Fe(III): a quantum mechanical study based on DFT computations and natural bond orbital analyses. *Inorganic Chemistry*, **2001,** 40(13), 3101-3112.

[176] Griffel, M.; Stout, J. W.: Preparation of Single Crystals of Manganous Fluoride. The Crystal Structure from X-Ray Diffraction. The Melting Point and Density. *Journal of the American Chemical Society*, **1950,** 72(10), 4351-4353.

[177] Stout, J. W.; Reed, S. A.: The Crystal Structure of MnF_2, FeF_2, CoF_2, NiF_2 and ZnF_2. *Journal of the American Chemical Society*, **1954,** 76(21), 5279-5281.

[178] Hepworth, M. A.; Jack, K. H.; Nyholm, R. S.: Interatomic Bonding in Manganese Trifluoride. *Nature*, **1957,** 179, 211-212.

[179] Palacio, F.; Andres, M.; Rodriguez-Carvajal, J.; Pannetier, J.: Magnetic structures of the Mn^{III} weak ferromagnets $AMnF_4 \cdot H_2O$ (A=Rb and K). *Journal of Physics: Condensed Matter*, **1991,** 3(14), 2379.

[180] Pebler, J.; Massa, W.; Lass, H.; Ziegler, B.: Intrachain exchange energies in 1-dimensional magnetic fluoromanganates(III) as a function of Mn-F-Mn bridge angle and crystal structure of Li_2MnF_5. *Journal of Solid State Chemistry*, **1987,** 71(1), 87-94.

[181] Massa, W.: Struktur von Natrium-pentafluoromanganat(III). *Acta Crystallographica Section C*, **1986,** 42(6), 644-647.

[182] Sears, D. R.; Hoard, J. L.: Crystal Structure of $(NH_4)_2MnF_5$. *The Journal of Chemical Physics*, **1969,** 50(3), 1066-1071.

[183] Edwards, A. J.: Fluoride crystal structures. Part XVII. Dipotassium pentafluoromanganate(III) hydrate. *Journal of the Chemical Society A: Inorganic, Physical, Theoretical*, **1971**, 2653-2655.

[184] Bukovec, P.; Kaucic, V.: Rubidium pentafluoromanganate(III) monohydrate. *Acta Crystallographica Section B*, **1978,** 34(11), 3339-3341.

[185] Englich, U.; Massa, W.; Tressaud, A.: Structure of trisodium hexafluoromanganate(III). *Acta Crystallographica Section C: Cryst. Struct. Commun.*, **1992,** C48(1), 6-8.

[186] Palacio, F.; Morón, M. C., Magneto-Structural Correlations in Mn(III) Fluorides. In *Research Frontiers in Magnetochemistry*, O' Connor, C. J., Ed. World Scientific: 1993; pp 227-281.

[187] Halcrow, M. A.: Interpreting and controlling the structures of six-coordinate copper(ii) centres - When is a compression really a compression? *Dalton Transactions*, **2003,** (23), 4375-4384.

[188] Docherty, R.; Tuna, F.; Kilner, C.; McInnes, E. J. L.; Halcrow, M. A.: Suppression of the Jahn-Teller Distortion in a Six-Coordinate Copper(II) Complex by Doping it into a Host Lattice. *Chemical Communications*, **2012**.

[189] Veidis, M. V.; Schreiber, G. H.; Gough, T. E.; Palenik, G. J.: Jahn-Teller distortions in octahedral copper(II) complexes. *Journal of the American Chemical Society*, **1969,** 91(7), 1859-1860.

[190] Nieuwenhuijse, B.; Reedijk, J.: Jahn-teller distortions in copper(II) complexes as determined from ESR powder spectra. *Chemical Physics Letters*, **1973,** 22(1), 201-203.

[191] Stebler, M.; Buergi, H. B.: Dynamic processes in crystals examined through difference displacement parameters .DELTA.U: pseudo-Jahn-Teller distortion in *cis*-Cu(II)N_4O_2 coordination octahedra. *Journal of the American Chemical Society*, **1987,** 109(5), 1395-1401.

[192] Schultz, A. J.; Hitchman, M. A.; Jorgensen, J. D.; Lukin, S.; Radaelli, P. G.; Simmons, C. J.; Stratemeier, H.: Hysteresis of the Pressure-Induced Jahn−Teller Switch in Deuterated Ammonium Copper(II) Tutton Salt, $(ND_4)_2[Cu(D_2O)_6](SO_4)_2$. *Inorganic Chemistry*, **1997,** 36(15), 3382-3385.

[193] Kundu, T. K.; Bruyndonckx, R.; Daul, C.; Manoharan, P. T.: A Density Functional Approach to the Jahn−Teller Effect of $[Cu(en)_3]^{2+}$ as a Model for a Macrobicyclic Cage Complex of Copper(II). *Inorganic Chemistry*, **1999,** 38(17), 3931-3934.

[194] Henning, R. W.; Schultz, A. J.; Hitchman, M. A.; Kelly, G.; Astley, T.: Structural and EPR Study of the Dependence on Deuteration of the Jahn−Teller Distortion in

Ammonium Hexaaquacopper(II) Sulfate, $(NH_4)_2[Cu(H_2O)_6](SO_4)_2$. *Inorganic Chemistry*, **2000,** 39(4), 765-769.

[195] Dick, A.; Krausz, E. R.; Hadler, K. S.; Noble, C. J.; Tregenna-Piggott, P. L. W.; Riley, M. J.: The Jahn−Teller Effect in Cu(II) Doped MgO. *The Journal of Physical Chemistry C*, **2008,** 112(37), 14555-14562.

[196] Ammeter, J.; Buergi, H. B.; Gamp, E.; Meyer-Sandrin, V.; Jensen, W. P.: Static and dynamic Jahn-Teller distortions in CuN_6 complexes. Crystal structures and EPR spectra of complexes between copper(II) and rigid, tridentate *cis,cis*-1,3,5-triaminocyclohexane. tach: $Cu(tach)_2(ClO_4)_2$, $Cu(tach)_2(NO_3)_2$. Crystal structure of $Ni(tach)_2(NO_3)_2$. *Inorganic Chemistry*, **1979,** 18(3), 733-750.

[197] Figgis, B. N.; Sobolev, A. N.; Simmons, C. J.; Hitchman, M. A.; Stratemeier, H.; Riley, M. J.: Bonding effects and the crystal structures of $(NH_4)_2[Cu(H_2O)_6](SO_4)_2$ and its $H_2{}^{18}O$ substituted form at 9.5 K. *Acta Crystallographica Section B*, **2000,** 56(3), 438-443.

[198] Ballirano, P.; Belardi, G.; Bosi, F.: Redetermination of the Tutton's salt $Cs_2[Cu(H_2O)_6](SO_4)_2$. *Acta Crystallographica Section E*, **2007,** 63(7), i164-i165.

[199] Nyberg, B.: The crystal structure of $(NH4)_2[Cu(H_2O)_6][CuSO_3]_4$. *Acta Crystallographica Section B*, **1978,** 34(5), 1418-1421.

[200] Bjerrum, J., *Metal Ammine Formation in Aqueous Solution. Theory of the reversible step reactions.* P. Haase and Son.: Copenhagen, 1957.

[201] Elliott, H.; Hathaway, B. J.: The Hexaammine Complexes of the Copper(II) Ion. *Inorganic Chemistry*, **1966,** 5(5), 885-889.

[202] Distler, T. M.; Vaughan, P. A.: Crystal structures of the hexaamminecopper(II) halides. *Inorganic Chemistry*, **1967,** 6(1), 126-129.

[203] Clifton, J. R.; Yoke, J. T.: Coordination and oxidation of ethylamine and diethylamine by copper(II) chloride. *Inorganic Chemistry*, **1968,** 7(1), 39-46.

[204] Barendregt, F.; Schenk, H.: The crystal structure of $CuCl_4(NH_3C_3H_7)_2$. *Physica*, **1970,** 49(3), 465-468.

[205] Beattie, J. K.; Best, S. P.; Skelton, B. W.; White, A. H.: Structural studies on the caesium alums, $CsM(SO4)_2$ $12H_2O$. *Journal of the Chemical Society, Dalton Transactions*, **1981,** (10), 2105-2111.

[206] Morosin, B.; Brathovde, J. R.: The crystal structure and molecular configuration of trisacetylacetonatomanganese(III). *Acta Crystallographica*, **1964,** 17(6), 705-711.

[207] Fackler, J. P.; Avdeef, A.: Crystal and molecular structure of tris(2,4-pentanedionato)manganese(III), $Mn(O_2C_5H_7)_3$, a distorted complex as predicted by Jahn-Teller arguments. *Inorganic Chemistry*, **1974,** 13(8), 1864-1875.

[208] Koenig, E.; Kremer, S.: Octahedral d^4, d^6 ligand field spin-orbit energy level diagrams. *The Journal of Physical Chemistry*, **1974,** 78(1), 56-59.

[209] Raghavacharyulu, I. V. V.: Simple proof of the Jahn-Teller theorem *Journal of Physics C: Solid State Physics* **1973,** 6(24), L455-L457.

[210] Jahn, H. A.: Stability of Polyatomic Molecules in Degenerate Electronic States. II. Spin Degeneracy. *Proceedings of the Royal Society of London. Series A - Mathematical and Physical Sciences*, **1938,** 164(916), 117-131.

[211] Jahn, H. A.; Teller, E.: Stability of Polyatomic Molecules in Degenerate Electronic States. I. Orbital Degeneracy. *Proceedings of the Royal Society of London. Series A, Mathematical and Physical Sciences*, **1937,** 161(905), 220-235.

[212] Henkel, H.; Hoppe, R.: Zur Kenntnis des Kryolith-Typs. Über Na_3NiF_6 und andere Kryolithe. *Zeitschrift für anorganische und allgemeine Chemie*, **1969,** 364(5-6), 253-262.

[213] Weinland, R. F.; Dinkelacker, P.: Beitrag zur Kenntnis der Einwirkung von Salzsäure auf Permanganate. *Zeitschrift für anorganische Chemie*, **1908,** 60(1), 173-177.

[214] Stults, B. R.; Marianelli, R. S.; Day, V. W.: Distortions of the coordination polyhedron in high-spin manganese(III) complexes. I. Synthesis and characterization of a series of five- and six-coordinate bis(acetylacetonato)manganese(III) complexes. Crystal structure of azidobis(acetylacetonato)manganese(III). *Inorganic Chemistry*, **1975,** 14(4), 722-730.

[215] Prabhakaran, C. P.; Patel, C. C.: *N,N'* ethylene bis (salicylideneimine) complexes of manganese (III) halides. *Journal of Inorganic and Nuclear Chemistry*, **1969,** 31(10), 3316-3319.

[216] Yuan, M.; Zhao, F.; Zhang, W.; Wang, Z.-M.; Gao, S.: Azide-Bridged One-Dimensional Mn^{III} Polymers: Effects of Side Group of Schiff Base Ligands on Structure and Magnetism. *Inorganic Chemistry*, **2007,** 46(26), 11235-11242.

[217] Ashmawy, F. M.; McAuliffe, C. A.; Parish, R. V.; Tames, J.: Some new manganese(III) complexes of the tetradentate schiff base ligand 1,3-*N,N'*-propylenebis(salicydeneaminato), [Mn(ligand)X]. *Inorganica Chimica Acta*, **1985,** 103(2), 133-136.

[218] Darensbourg, D. J.; Frantz, E. B.: X-Ray crystal structures of five-coordinate (salen)MnN_3 derivatives and their binding abilities towards epoxides: chemistry relevant to the epoxide-CO_2 copolymerization process. *Dalton Transactions*, **2008,** (37), 5031-5036.

[219] Jutz, F.; Grunwaldt, J.-D.; Baiker, A.: Mn(III)(salen)-catalyzed synthesis of cyclic organic carbonates from propylene and styrene oxide in "supercritical" CO_2. *Journal of Molecular Catalysis A: Chemical*, **2008,** 279(1), 94-103.

[220] Pecoraro, V. L.; Butler, W. M.: Structure of *N,N'*-ethylenebis(salicylideneiminato)manganese(III) chloride acetonitrile solvate. *Acta Crystallographica Section C: Cryst. Struct. Commun.*, **1986,** C42(9), 1151-1154.

[221] Martínez, D.; Motevalli, M.; Watkinson, M.: Aquachloro[*N,N'*-ethylenebis(salicylideneiminato)]manganese(III). *Acta Crystallographica Section C: Cryst. Struct. Commun.*, **2002,** C58(4), m258-m260.

[222] Mitra, K.; Biswas, S.; Lucas, C. R.; Adhikary, B.: Manganese(III) complexes of N_2O_2 donor 5-bromosalicylideneimine ligands: Combined effects of electron withdrawing substituents and chelate ring size variations on electrochemical properties. *Inorganica Chimica Acta*, **2006,** 359(7), 1997-2003.

[223] Shyu, H.-L.; Wei, H.-H.; Wang, Y.: Structure and magnetic properties of dinuclear $[Mn(III)(salen)(H_2O)]_2(ClO_4)_2$ and polynuclear $[Mn(III)(salen)(NO_3)]_n$. *Inorganica Chimica Acta*, **1999,** 290(1), 8-13.

[224] Zhang, W.; Loebach, J. L.; Wilson, S. R.; Jacobsen, E. N.: Enantioselective epoxidation of unfunctionalized olefins catalyzed by salen manganese complexes. *Journal of the American Chemical Society*, **1990,** 112(7), 2801-2803.

[225] Yuan, M.; Zhao, F.; Zhang, W.; Pan, F.; Wang, Z.-M.; Gao, S.: Hydrogencyanamide-Bridged One-Dimensional Polymers Built on MnIII–Schiff Base Fragments: Synthesis, Structure, and Magnetism. *Chemistry – A European Journal*, **2007,** 13(10), 2937-2952.

[226] Sailaja, S.; Reddy, K. R.; Rajasekharan, M. V.; Hureau, C.; Rivière, E.; Cano, J.; Girerd, J. J.: Synthesis, Structure, and Magnetic Properties of $[Mn^{III}(salpn)NCS]_n$, a Helical Polymer, and the Dimer $[Mn^{III}(salpn)NCS]_2$. Weak Ferromagnetism in $[Mn^{III}(salpn)NCS]_n$ Related to the Strong Magnetic Anisotropy in Jahn−Teller Mn(III) ($salpnH_2$ = *N,N'*-Bis(salicylidene)-1,3-diaminopropane). *Inorganic Chemistry*, **2002,** 42(1), 180-186.

[227] Kennedy, B. J.; Murray, K. S.: Magnetic properties and zero-field splitting in high-spin manganese(III) complexes. 1. Mononuclear and polynuclear Schiff-base chelates. *Inorganic Chemistry*, **1985,** 24(10), 1552-1557.

[228] Matsumoto, N.; Sunatsuki, Y.; Miyasaka, H.; Hashimoto, Y.; Luneau, D.; Tuchagues, J.-P.: $[\{Mn(salen)CN\}_n]$: The First One-Dimensional Chain with Alternating High-Spin and Low-Spin Mn^{III} Centers Exhibits Metamagnetism. *Angewandte Chemie International Edition*, **1999,** 38(1-2), 171-173.

[229] Ko, H. H.; Lim, J. H.; Kim, H. C.; Hong, C. S.: Coexistence of Spin Canting and Metamagnetism in a One-Dimensional Mn(III) Complex Bridged by a Single End-to-End Azide. *Inorganic Chemistry*, **2006,** 45(22), 8847-8849.

[230] Hui, L.; Zhuang Jin, Z.; Chun-Ying, D.; Xiao-Zeng, Y.; Mak, T. C. W.; Bown, W.: Synthesis, structure and magnetic properties of one-dimensional azide-bridged manganese(III) uniform chain complex $Mn(salpn)N_3$. *Inorganica Chimica Acta*, **1998,** 271(1-2), 99-104.

[231] Boucher, L. J.; Farrell, M. O.: Manganese Schiff's base complexes I: Synthesis and spectroscopy of some anion complexes of (4-sec-butylsalicylaldehydeethylenediiminato) manganese(III). *Journal of Inorganic and Nuclear Chemistry*, **1973,** 35(11), 3731-3738.

[232] Panja, A.; Shaikh, N.; Vojtisek, P.; Gao, S.; Banerjee, P.: Synthesis, crystal structures and magnetic properties of 1D polymeric $[Mn(salen)N_3]$ and $[Mn(salen)Ag(CN)_2]$ complexes. *New Journal of Chemistry*, **2002,** 26(8), 1025-1028.

[233] Rand, L.; Swisher, J. V.; Cronin, C. J.: Reactions Catalyzed by Potassium Fluoride. III. The Knoevenagel Reaction. *The Journal of Organic Chemistry*, **1962,** 27(10), 3505-3507.

[234] Jones, L. F.; Raftery, J.; Teat, S. J.; Collison, D.; Brechin, E. K.: Manganese (III) fluoride as a new synthon in Mn cluster chemistry. *Polyhedron*, **2005,** 24(16-17), 2443-2449.

[235] Matsushita, T.; Kono, H. K.; Shono, T.: The Preparation and Characterization of Dichloromanganese(IV) Schiff Base Complexes. *Bulletin of the Chemical Society of Japan* **1981,** 54(9), 2646-2651.

[236] Asada, H.; Fujiwara, M.; Matsushita, T.: Structures of dichloromanganese(IV) complexes with bidentate and tetradentate Schiff base ligands. *Polyhedron*, **2000,** 19(18-19), 2039-2048.

[237] Fackler, J. P.; Chawla, I. D.: Spectra of Manganese(III) Complexes. I. Aquomanganese(III) Ion and Hydroxide, Fluoride, and Chloride Complexes. *Inorganic Chemistry*, **1964,** 3(8), 1130-1134.

[238] Hernández-Molina, R.; Mederos, A.; Gili, P.; Domínguez, S.; Núñez, P.: Complexation equilibria of *N,N'*-o-phenylenebis(salicylideneimine). *Polyhedron*, **1997,** 16(24), 4191-4196.

[239] Kitko, D. J.; Wiegers, K. E.; Smith, S. G.; Drago, R. S.: A kinetic study of the reaction of *N,N'*-ethylenebis(salicylideneiminato)cobalt(II) with bis(hexafluoroacetylacetonato)copper(II). *J. Am. Chem. Soc.*, **1977,** 99(5), 1410-1416.

[240] Lloret, F.; Mollar, M.; Faus, J.; Julve, M.; Castro, I.; Diaz, W.: Solution chemistry of *N,N'*-ethylenebis(salicylideneimine) and its copper(II), nickel(II) and iron(III) complexes. *Inorganica Chimica Acta*, **1991,** 189(2), 195-206.

[241] Prasad, D. R.; Ramasami, T.; Ramaswamy, D.; Santappa, M.: Kinetics and mechanism of the equilibration reactions of diaquachromium(III)-Schiff base derivatives, Cr(Schiff base)$(H_2O)^{2+}$, and their conjugate bases with thiocyanate, azide, imidazole, pyridine and nicotinic acid as ligands. *Inorg. Chem.*, **1982,** 21(3), 850-854.

[242] Oki, A. R.; Hodgson, D. J.: Synthesis, characterization and catalytic properties of manganese(III) schiff base complexes. *Inorganica Chimica Acta*, **1990,** 170(1), 65-73.

[243] Yoon, J. W.; Yoon, T. S.; Lee, S. W.; Shin, W.: (1*R*,2*R*)-(-)-[Bis(3,5-di-tert-butylsalicylidene)-1,2-cyclohexanediamine]chloromanganese(III), an (*R*,*R*)-Jacobsen catalyst. *Acta Crystallographica Section C*, **1999,** 55(11), 1766-1769.

[244] Miyasaka, H.; Clerac, R.; Ishii, T.; Chang, H.-C.; Kitagawa, S.; Yamashita, M.: Out-of-plane dimers of Mn(iii) quadridentate Schiff-base complexes with saltmen^{2-} and naphtmen^{2-} ligands: structure analysis and ferromagnetic exchange. *Journal of the Chemical Society, Dalton Transactions*, **2002,** (7).

[245] Hirotsu, M.; Nakajima, K.; Kojima, M.; Yoshikawa, Y.: Manganese(III) Complexes Containing Optically Active Tetradentate Schiff Base Ligands. Effect of Phenyl Substituents. *Inorganic Chemistry*, **1995,** 34(24), 6173-6178.

[246] Panja, A.; Shaikh, N.; Ali, M.; Vojtíšek, P.; Banerjee, P.: Structural characterization of a new manganese(III)–salen complex [H_2salen=*N,N′*-bis(salicylidene)ethane-1,2-diamine] and study of its electron transfer kinetics with hydroquinone and catechol. *Polyhedron*, **2003,** 22(9), 1191-1198.

[247] Horwitz, C. P.; Dailey, G. C.; Tham, F. S.: Aquachloro[bis(5-chlorosalicylidene)ethylenediaminato]manganese(III). *Acta Crystallographica Section C*, **1995,** 51(5), 815-817.

[248] Dang, L.-L.; Huo, Y.-Q.; Wang, W.; Li, J.: Aqua[bis(5-bromosalicylidene)ethylenediaminato]chloromanganese(III). *Acta Crystallographica Section E*, **2005,** 61(2), m332-m334.

[249] Hwang, I.-C.; Ha, K.: Crystal structure of aquachloro[*N,N′*-bis(3-ethoxysalicylidene)ethylenediiminato]manganese(III) dihydrate, $[Mn(C_{20}H_{22}N_2O_4)Cl(H_2O)]\cdot 2H_2O$. *Zeitschrift für Kristallographie - New Crystal Structures* **2006,** 221(3), 363.

[250] Bermejo, M. R.; Castineiras, A.; Garcia-Monteagudo, J. C.; Rey, M.; Sousa, A.; Watkinson, M.; McAuliffe, C. A.; Pritchard, R. G.; Beddoes, R. L.: Electronic and steric effects in manganese Schiff-base complexes as models for the water oxidation complex in photosystem II. The isolation of manganese-(II) and -(III) complexes of 3- and 3,5-substituted *N,N′*-bis(salicylidene)ethane-1,2-diamine (H_2salen) ligands. *Journal of the Chemical Society, Dalton Transactions*, **1996,** (14), 2935-2944.

[251] Hwang, I.-C.; Ha, K.: Crystal structure of aquachloro[*N,N'*-bis(3-ethoxysalicylidene)propylenediiminato]manganese(III) dihydrate, [$Mn(C_{21}H_{24}N_2O_4)Cl(H_2O)$] · $2H_2O$. *Zeitschrift für Kristallographie - New Crystal Structures* **2006,** 221(3), 365.

[252] Korendovych, I. V.; Rybak-Akimova, E. V.: Chloro{2,2'-[(1*S*,2*S*)-1,2-diphenyl-1,2-ethanediylbis(nitrilomethylidyne)]diphenolato-κ^4*O,N,N',O'*}(ethanol-*kO*)manganese(III). *Acta Crystallographica Section C*, **2004,** 60(2), m82-m84.

[253] Das, D.; Pyeng Cheng, C.: Synthesis and characterization of mono- and bi-metallic Mn(III) complexes containing salen type ligands. *Journal of the Chemical Society, Dalton Transactions*, **2000,** (7), 1081-1086.

[254] Lee, S. M.; Lo, K. M.; Ng, S. W.: Aqua{6,6'-dimethoxy-2,2'-[ethane-1,2-diylbis(nitrilomethylidyne)]diphenolato-κ^4*O,N,N',O'*}(formato-κ*O*)manganese(III) dihydrate. *Acta Crystallographica Section E*, **2011,** 67(6), m746.

[255] Watkinson, M.; Fondo, M.; R. Bermejo, M.; Sousa, A.; A. McAuliffe, C.; G. Pritchard, R.; Jaiboon, N.; Aurangzeb, N.; Naeem, M.: Further attempts to rationalise the co-ordination chemistry of manganese with Schiff base ligands and supplementary carboxylate donors. *Journal of the Chemical Society, Dalton Transactions*, **1999,** (1), 31-42.

[256] Ha, K.: Crystal structure of acetato[*N,N'*-bis(3-ethoxysalicylidene)propane-1,3-diiminato]manganese(III) - acetonitrile (1:1), $Mn(C_{21}H_{24}N_2O_4)(CH_3COO)$ · CH_3CN. *Zeitschrift für Kristallographie – New Crystal Structures* **2010,** 225(2), 257.

[257] Oyaizu, K.; Nakagawa, T.; Tsuchida, E.: Crystal structures of dimeric manganese(III) complexes of tetradentate Schiff-base ligands with ancillary axial donors. *Inorganica Chimica Acta*, **2000,** 305(2), 184-188.

[258] Reshma, R.; Soumya, P. V.; Simi, S. M.; Thampidas, V. S.; Pike, R. D.: Aqua{6,6'-dimethoxy-2,2'-[ethane-1,2-diylbis(nitrilomethylidyne)]diphenolato}(4-hydroxybenzoato)manganese(III). *Acta Crystallographica Section E*, **2009,** 65(9), m1110-m1111.

[259] Thampidas, V. S.; Radhakrishnan, T.; Pike, R. D.: Aqua{2,2-[ethane-1,2-diylbis(nitrilomethylidyne)]diphenolato}(3-nitrobenzoato)manganese(III). *Acta Crystallographica Section E*, **2008,** 64(8), m990-m991.

[260] Liu, Y.: Azido{4,4'-dibromo-2,2'-[ethane-1,2-diylbis(nitrilomethanylylidene)]diphenolato-κ^4*O,N,N′,O′*}manganese(III). *Acta Crystallographica Section E*, **2011,** 67(3), m322.

[261] Khalaji, A.; Hadadzade, H.; Fejfarova, K.; Dusek, M.: Synthesis, characterization, and X-ray crystal structure of the manganese(III) complex $Mn(Sal_2hn)(CH_3OH)(N_3)$ [Sal_2hn = *N,N′*-bis(salicylidene)-1,2-hexanediamine]. *Russian Journal of Coordination Chemistry*, **2010,** 36(8), 618-621.

[262] Kurahashi, T.; Fujii, H.: Chiral Distortion in a $Mn^{IV}(salen)(N_3)_2$ Derived from Jacobsen's Catalyst as a Possible Conformation Model for Its Enantioselective Reactions. *Inorganic Chemistry*, **2008,** 47(17), 7556-7567.

[263] Aurangzeb, N.; Hulme, C. E.; McAuliffe, C. A.; Pritchard, R. G.; Watkinson, M.; Bermejo, M. R.; Garcia-Deibe, A.; Rey, M.; Sanmartin, J.; Sousa, A.: Crystallographic characterisation of a possible model for photosystem II. *Journal of the Chemical Society, Chemical Communications*, **1994,** (9), 1153-1155.

[264] Akitsu, T.; Takeuchi, Y.; Einaga, Y.: Diaqua[*N,N′*-bis(5-methoxysalicylidene)ethylenediaminato-κ^4*O,N,N′,O′*]manganese(III) perchlorate, aqua[*N,N′*-bis(3,5-dichlorosalicylidene)ethylenediaminato-κ^4*O,N,N′,O′*](methanol-κ*O*)manganese(III) perchlorate and bis[μ-*N,N′*-bis(3-methoxysalicylidene)ethylenediaminato-κ^5*O,N,N′,O′:O*]bis[(methanol-κ*O*)manganese(III)] diperchlorate. *Acta Crystallographica Section C*, **2005,** 61(7), m324-m328.

[265] Akitsu, T.; Takeuchi, Y.; Einaga, Y.: Diaqua[(1*R*,2*R*)-*N,N′*-bis(3,5-dibromosalicylidene)cyclohexane-1,2-diamine-[kappa]4*O*,*N*,*N′*,*O′*]manganese(III) perchlorate dihydrate. *Acta Crystallographica Section E*, **2005,** 61(4), m772-m774.

[266] Cheng, S.-C.; Chang, C.-W.; Wei, H.-H.; Lee, G.-H.; Wang, Y.: Mononuclear Iron(III) and Manganese(III) Complexes with Substituted Salicylaldimine Ligands: Structure, Magnetic Properties, and Catalytic Activity of Olefins-Epoxidation. *Journal of the Chinese Chemical Society*, **2003,** 50(1), 41-46.

[267] Chattopadhyay, T.; Islam, S.; Nethaji, M.; Majee, A.; Das, D.: Mono- and bi-metallic Mn(III) complexes of macroacyclic salen type ligands: Syntheses, characterization and studies of their catalytic activity. *Journal of Molecular Catalysis A: Chemical*, **2007,** 267(1–2), 255-264.

[268] Korupoju, S. R.; Mangayarkarasi, N.; Ameerunisha, S.; Valente, E. J.; Zacharias, P. S.: Formation of dinuclear macrocyclic and mononuclear acyclic complexes of a new trinucleating hexaaza triphenolic Schiff base macrocycle: structure and NLO properties. *Journal of the Chemical Society, Dalton Transactions*, **2000,** (16).

[269] Banu, K. S.; Chattopadhyay, T.; Banerjee, A.; Mukherjee, M.; Bhattacharya, S.; Patra, G. K.; Zangrando, E.; Das, D.: Mono- and dinuclear manganese(III) complexes showing efficient catechol oxidase activity: syntheses, characterization and spectroscopic studies. *Dalton Transactions*, **2009,** (40).

[270] Kara, H.: Crystal Structure of Diaqua[*N,N′*-bis(3-methoxysalicylidene)propane-1,2-diaminato]manganese(III) nitrate monohydrate. *Analytical Sciences X-ray Structure Analysis Online*, **2008,** 24, x263-x264.

[271] Bermejo, M. R.; Fernández, M. I.; Gómez-Fórneas, E.; González-Noya, A.; Maneiro, M.; Pedrido, R.; Rodríguez, M. J.: Self-Assembly of Dimeric Mn^{III}–Schiff-Base Complexes Tuned by Perchlorate Anions. *European Journal of Inorganic Chemistry*, **2007,** 2007(24), 3789-3797.

[272] Butcher, R. J.; Towns, W.: Aqua[*N,N′*-ethylenebis(5-nitrosalicylideneiminato)]methanolmanganese(III) perchlorate. *Acta Crystallographica Section E*, **2005,** 61(12), m2618-m2620.

[273] Kara, H.: Crystal Structure of Aqua*[N,N′*-bis(3-methoxysalicylidene)propane-1,2-diaminato]methanolmanganese(III) Perchlorate. *Analytical Sciences X-ray Structure Analysis Online*, **2008,** 24, x79-x80.

[274] Miyasaka, H.; Nezu, T.; Sugimoto, K.; Sugiura, K.-i.; Yamashita, M.; Clérac, R.: (5-Rsaltmen)$_2$Ni^{II}(pao)$_2$(L)]$^{2+}$: An S_T=3 Building Block for a Single-Chain Magnet That Behaves as a Single-Molecule Magnet. *Chemistry – A European Journal*, **2005,** 11(5), 1592-1602.

[275] Brown, M. K.; Blewett, M. M.; Colombe, J. R.; Corey, E. J.: Mechanism of the Enantioselective Oxidation of Racemic Secondary Alcohols Catalyzed by Chiral Mn(III)−Salen Complexes. *Journal of the American Chemical Society*, **2010,** 132(32), 11165-11170.

[276] Gohdes, J. W.; Armstrong, W. H.: An unstable manganese(III) complex incorporating ligand donor types proposed for an acid phosphatase from sweet potato: (p-nitrobenzenethiolato)[N,N'-ethylenebis(salicylideneaminato)]manganese(III). *Inorganic Chemistry*, **1988,** 27(11), 1841-1842.

[277] Zhang, D.: Monoclinic form of (cyanato-κ*N*){2,2'-[ethane-1,2-diylbis(nitrilomethylidyne)]diphenolato-κ^4*O,N,N',O'*}manganese(III). *Acta Crystallographica Section E*, **2010,** 66(12), m1656.

[278] Li, H.; Zhong, Z. J.; Duan, C.-Y.; You, X.-Z.; Mak, T. C. W.; Wu, B.: Synthesis and Crystal Structure og Manganese(III) Complex with Tetradentate Schiff base *N,N'*-ethylenebis(salicylideneiminato). *Journal of Coordination Chemistry*, **1997,** 41(3), 183-189.

[279] Shyu, H. L.; Wei, H. H.; Wang, Y.: Structure and magnetic properties of dinuclear $[Mn(III)(salen)(H_2O)]_2(ClO_4)_2$ and polynuclear $[Mn(III)(salen)(NO_3)]_n$. *Inorganica Chimica Acta*, **1999,** 290(1), 8-13.

[280] Liu, Y.; Dou, J.; Niu, M.; Zhang, X.: μ-Oxido-bis({4,4'-dibromo-2,2'-[ethane-1,2-diylbis(nitrilomethylidyne)]diphenolato}manganese(III)). *Acta Crystallographica Section E, Structure Reports*, **2007,** 63(11), m2771.

[281] Glerup, J.; Weihe, H.: Magnetic Susceptibility and EPR Spectra of μ-Cyano-bis[pentaamminechromium(III)] Perchlorate. *Acta Chemica Scandinavica*, **1991,** 45, 444-448.

[282] Jacobsen, C. H.; Pedersen, E.; Villadsen, J.; Weihe, H.: ESR Characterization of *trans*-$V^{II}(py)_4X_2$ and *trans*-$Mn^{II}(py)_4X_2$ (X = NCS, Cl, Br, I; py = pyridine). *Inorganic Chemistry*, **1993,** 32(7), 1216-1221.

[283] Weihe, H., Simulation of EPR. In University of Copenhagen: Copenhagen 2002.

[284] Fisher, M. E.: Magnetism in One-Dimensional Systems---The Heisenberg Model for Infinite Spin. *American Journal of Physics*, **1964,** 32(5), 343-346.

[285] Bonner, J. C.; Fisher, M. E.: Linear Magnetic Chains with Anisotropic Coupling. *Physical Review*, **1964,** 135(3A), A640.

[286] Christian, P.; Rajaraman, G.; Harrison, A.; McDouall, J. J. W.; Raftery, J. T.; Winpenny, R. E. P.: Structural, magnetic and DFT studies of a hydroxide-bridged $\{Cr_8\}$ wheel. *Dalton Transactions*, **2004,** (10), 1511-1512.

[287] Ardon, M.; Bino, A.; Michelsen, K.; Pedersen, E.; Thompson, R. C.: Chain-Structured *cis*-Hydroxoaquachromium(III) Complexes and Their Magnetic Properties. *Inorganic Chemistry*, **1997,** 36(19), 4147-4150.

[288] Wagner, G. R.; Friedberg, S. A.: Linear chain antiferromagnetism in Mn(HCOO)2 · 2H2O. *Physics Letters*, **1964,** 9(1), 11-13.

[289] Davies, J. E.; Gatehouse, B. M.; Murray, K. S.: Crystal and molecular structure and magnetic properties of *catena*-μ-acetato-[*N,N'*-ethylenebis(salicylaldiminato)]manganese(III). A linear-chain complex containing a single *anti-anti* acetate bridge. *Journal of the Chemical Society, Dalton Transactions*, **1973,** (22), 2523-2527.

[290] Neese, F. *ORCA* 2.8, revision 2131; Institut für Physikalische und Theoretische Chemie, Universitaet Bonn, Germany.: 2010.

[291] Mazej, Z.: Room temperature syntheses of MnF_3, MnF_4 and hexafluoromanganete(IV) salts of alkali cations. *Journal of Fluorine Chemistry*, **2002,** 114(1), 75-80.

[292] Mason, A. T.: Über Condensationsderivate des Aethylendiamins. *Chem. Ber.*, **1887,** 20, 267-277.

[293] Chakraborty, J.; Samanta, B.; Pilet, G.; Mitra, S.: Synthesis, structure and spectral characterisation of a hydrogen-bonded polymeric manganese(III) Schiff base complex. *Structural Chemistry*, **2006,** 17(6), 585-593.

[294] Beauchamp, D. A.; Loeb, S. J.: Hydrogen-Bonded Networks through Second-Sphere Coordination. *Chemistry – A European Journal*, **2002,** 8(22), 5084-5088.

[295] Sharma, R.; Sharma, R. P.; Kariuki, B. M.: Second sphere interaction in fluoroanion binding: Synthesis, spectroscopic and X-ray structural study of *trans*-dichlorobis(ethylenediamine) cobalt(III) terafluoroborate. *Journal of Fluorine Chemistry*, **2008,** 129(5), 325-331.

[296] Sharma, R. P.; Singh, A.; Brandão, P.; Felix, V.; Venugopalan, P.: Second sphere coordination in binding of fluoroanions: Synthesis, spectroscopic characterization and single crystal X-ray structure determination of $[Co(phen)_3](BF_4)_3 \cdot H_2O$ and $[Co(phen)_3](PF_6)_3 \cdot CH_3COCH_3$. *Journal of Molecular Structure*, **2009,** 920(1-3), 119-127.

[297] Singh, A.; Sharma, R. P.; Aree, T.; Venugopalan, P.: Second sphere coordination in fluoroanion binding: Synthesis, spectroscopic and X-ray structural study of $[Co(phen)_2(CO)_3](Pfbz) \cdot 6H_2O$. *Journal of Fluorine Chemistry*, **2009,** 130(7), 650-655.

[298] McNaught, A. D.; Wilkinson, A., Solvatochromism. In *IUPAC. Compendium of Chemical Terminology*, 2 ed.; Blackwell Scientific Publications: Oxford, 1997.

[299] Kaizaki, S.; Takemoto, H.: Solvent-dependent deuteron NMR spectra and solvatochromism in ligand field absorption bands for *trans*-$[CrX_2(3,2,3\text{-tet})]^+$: correlation with the angular overlap model parametrization. *Inorganic Chemistry*, **1990,** 29(24), 4960-4964.

[300] Terasaki, Y.; Fujihara, T.; Kaizaki, S.: Mutual Influences of Ligands as Revealed by the 2H NMR Chemical Shifts and the Angular Overlap Model Parameters: *cis*-$[CrX_2(N)_4]$ and *fac*-$[CrX_3(N)_3]$-Type Complexes with Aromatic Amines. *European Journal of Inorganic Chemistry*, **2007,** 2007(21), 3400-3404.

[301] Terasaki, Y.; Kaizaki, S.: Solvent-dependent 2H nuclear magnetic resonance spectra and solvatochromism in ligand-field absorption bands of *cis*-$[CrF_2N_4]$-type complexes with triethylenetetramine, pyridine or 2,2'-bipyridine. *Journal of the Chemical Society, Dalton Transactions: Inorganic Chemistry*, **1995,** 17, 2837-2841.

[302] Yamaguchi-Terasaki, Y.; Fujihara, T.; Kaizaki, S.: Mutual Influences of Ligands as Revealed by ^2H NMR Shifts and the Angular Overlap Model Parameters: *trans*- and *cis*-$[CrX_2(N)_4]$-Type Complexes with Aliphatic Amine Ligands. *European Journal of Inorganic Chemistry*, **2007,** 2007(21), 3394-3399.

[303] Yamaguchi-Terasaki, Y.; Fujihara, T.; Nagasawa, A.; Kaizaki, S.: *cis*-Bis(2,2'-bipyridine)difluorochromium(III) perchlorate. *Acta Crystallographica Section E, Structure Reports*, **2007,** E63(2), m593.m595.

[304] Liu, F.-Q.; Kuhn, A.; Herbst-Irmer, R.; Stalke, D.; Roesky, H. W.: Molecular Solids as Ligands in Organometallic Chemistry: $[Cp_6^*Ti_6Na_7F_{19} \cdot 2.5thf]$ ($Cp^* = C_5Me_5$) and

[Cp_4*$Ti_4Mg_2F_{12}\cdot 7thf$], Links Between Ionic Solids and Organometallic Compounds. *Angewandte Chemie International Edition in English*, **1994,** 33(5), 555-556.

[305] Perdih, F.; Pevec, A.; Košmrlj, J.; Demšar, A.: X-ray crystal structures and solution dynamics of sodium organofluorotitanates [$Na\{Ti_2(C_5Me_5)_2F_7\}$] and [$NaTi_6(C_5Me_5)_5F_{20}(H_2O)$]·(THF). *Journal of Fluorine Chemistry*, **2006,** 127(10), 1289-1293.

[306] Hubbard, D. J.; Johnston, A. R.; Casalongue, H. S.; Sarjeant, A. N.; Norquist, A. J.: Synthetic Approaches for Noncentrosymmetric Molybdates. *Inorganic Chemistry*, **2008,** 47(19), 8518-8525.

[307] Michailovski, A.; Rüegger, H.; Sheptyakov, D.; Patzke, G. R.: Synthesis and Characterization of Novel Fluorinated Poly(oxomolybdates). *Inorganic Chemistry*, **2006,** 45(14), 5641-5652.

[308] Pedersen, C. J.: Cyclic polyethers and their complexes with metal salts. *Journal of the American Chemical Society*, **1967,** 89(26), 7017-7036.

[309] Pedersen, C. J.: Cyclic polyethers and their complexes with metal salts. *Journal of the American Chemical Society*, **1967,** 89(10), 2495-2496.

[310] Görge, A.; Dehnicke, K.: Synthese und Kristallstruktur von [Na-15-Krone-5][WF_5(NCl)]. *Zeitschrift für Naturforschung B*, **1989,** 44b, 117-120.

[311] Rentschler, E.; Massa, W.; Vogler, S.; Dehnicke, K.; Fenske, D.; Baum, G.: Halogeno-Nitrosylkomplexe von Molybdän und Wolfram. Die Kristallstrukturen von [Na_2(15-Krone-5)$_2$(CH_3CN)][$MoCl_4(NO)_2$] und [Na(15-Krone-5)]$_2$[$MoF_4Cl(NO)$]. *Zeitschrift für anorganische und allgemeine Chemie*, **1991,** 592(1), 59-72.

[312] Völp, K.; Dehnicke, K.; Fenske, D.: Synthese und Kristallstruktur des Nitridokomplexes [Na-15-Krone-5]$_2$[$MoNF_4$]$_2$·2 CH_3CN. *Zeitschrift für anorganische und allgemeine Chemie*, **1989,** 572(1), 26-32.

[313] Völp, K.; Baum, G.; Massa, W.; Dehnicke, K.: [Na-15-Krone-5][$MoF_2Cl_2(N_3S_2)$]; Synthese und Kristallstruktur. *Zeitschrift für Naturforschung B*, **1988,** 43b, 1235-1239.

[314] El-Kholi, A.; Völp, K.; Müller, U.; Dehnicke, K.: Synthese und Kristallstruktur des µ-Dinitridosulfato(II)-Komplexes [Na-15-Krone-5]$_2$[µ-(NSN)(MoF_5)$_2$]. *Zeitschrift für anorganische und allgemeine Chemie*, **1989,** 572(1), 18-25.

[315] Borgmann, C.; Limberg, C.; Kaifer, E.; Pritzkow, H.; Zsolnai, L.: Syntheses and X-ray structures of complexes with (η^3-allyl)Mo-units in oxygen-rich coordination spheres. *Journal of Organometallic Chemistry*, **1999,** 580(2), 214-224.

[316] Fenske, D.: Synthese und Kristallstruktur von [Na-15-Krone-5][MoF_5(NCl)]. *Zeitschrift für Naturforschung B*, **1988,** 43b, 1125-1129.

[317] Stenger, H.; Dehnicke, K.; Hiller, W.: [K(18-Krone-6)][WF_5(NCl)]: Synthese und Kristallstruktur. *Zeitschrift für Naturforschung B*, **1992,** 47, 1054-1056

[318] Werth, A.; Dehnicke, K.; Fenske, D.; Baum, G.: Zur Chemie von unsymmetrisch substituierten Alkinkomplexen des Wolframs Die Kristallstruktur von [Na-15-Krone-5][WF5(Ph-C≡C-C≡C-SiMe3)]·CH3CN. *Zeitschrift für anorganische und allgemeine Chemie*, **1990,** 591(1), 125-136.

[319] Neumann, P.; Dehnicke, K.; Bäuml, P.; Hiller, W.: Fluoroderivate von Diphenylacetylenkomplexen des Wolframs Die Kristallstruktur von [Na-15-Krone-5][WF5(PhC≡CPh)]. *Zeitschrift für anorganische und allgemeine Chemie*, **1990,** 582(1), 7-14.

[320] Neumann, P.; El-Kholi, A.; Müller, U.; Dehnicke, K.: Synthese, IR- und Kernresonanzspektren sowie Kristallstruktur von [Na-15-Krone-5][WF5(PhC≡CH)]. *Zeitschrift für anorganische und allgemeine Chemie*, **1989,** 577(1), 185-194.

[321] Neumann, P.; Dehnicke, K.; Fenske, D.; Baum, G.: Fluoro-, Chloro- und Bromo-Wolfram(VI)-Alkinkomplexe. Die Kristallstrukturen von [K(18-crown-6)][WF_5(Ph−C≡C−H)]·CH_3CN und [K(18-Krone-6)][WF_5(Ph-C≡C-Se-n-C_4H_9)(CH_3CN)]. *Zeitschrift für Naturforschung B*, **1991,** 46(8), 999-1010

[322] Vogler, S.; Dehnicke, K.: [Na-15-Krone-5][$ReF_2Cl_2(NO)_2$]; Synthese, IR-Spektrum und Kristallstruktur. *Zeitschrift für Naturforschung B*, **1989,** 44b, 1393-1396.

[323] Vogler, S.; Massa, W.; Dehnicke, K.: [Na(15-Krone-5)][$ReFCl_3$(NO)(CH_3CN)] Synthese, IR-Spektrum und Kristallstruktur. *Zeitschrift für anorganische und allgemeine Chemie*, **1992,** 610(4), 112-116.

[324] Borgholte, H.; Dehnicke, K.; Goesmann, H.; Fenske, D.: Synthese und Kristallstruktur von [Na-Benzo-15-Krone-5][$WF_2Cl_2(N_3S_2)$]. *Zeitschrift für anorganische und allgemeine Chemie*, **1990,** 586(1), 159-165.

[325] Rehder, D.; Fenske, D.; Baum, G.; Borgholte, H.; Dehnicke, K.: Spectroskopische Untersuchung des $[FCl_2V(^{15}N_3S_2]^-$-ion; Kristallstruktur von [Na-Benzo-15-Krone-5]$[FCl_2V(N_3S_2)]$. *Zeitschrift für Naturforschung B*, **1989,** 44b, 1385-1392.

[326] Fochi, G.; Straehle, J.; Gingl, F.: Disproportionation of the bis(benzene)chromium cation induced by pyridine. Crystal and molecular structure of two tetrapyridine complexes containing chromium(II) and chromium(III). *Inorganic Chemistry*, **1991,** 30(24), 4669-4671.

[327] Bragg, W. L.: Crystal Structure 1. *Nature*, **1920,** 105, 646-648

[328] Li, H.; Lee, G.-H.; Peng, S.-M.: Synthesis and crystal structure of trichromium metal string complex. *Journal of Molecular Structure*, **2004,** 707(1-3), 179-186.

[329] Glerup, J.; Josephsen, J.; Michelsen, K.; Pedersen, E.; Schäffer, C. E.: Preparation of Chromium(III) Complexes with Two Fluorine Atoms and Four Nitrogen Atoms as Ligators. trans-Difluorotetrakis(pyridine)chromium(III) Salts as Initial Materials. *Acta Chemica Scandinavica* **1970,** 24, 247-254.

[330] Josephsen, J.; Schäffer, C. E.: "*trans*-Dichlorobis(1,10-phenanthroline)cobalt(III) Chloride Hydrochloride Trihydrate" - Composition and Configuration. *Acta Chemica Scandinavica* **1969,** 23, 2206-2207.

[331] Andersen, P.; Josephsen, J.: Configurational Correlations of *cis*-Bis(2,2'-bipyridine) and of *cis*-Bis(1,10-phenanthroline) Complexes of Trivalent Metals by Means of X-Ray Powder Photographs. *Acta Chemica Scandinavica* **1971,** 25(9), 3255-3260.

[332] Bendix, J. Aspects of Strong π-Bonding in Systems witj Partly Filled *d*-Shells. University of Copenhagen, Copenhagen, 1998.

[333] Jöurgensen, S. M.: Zur Konstitution der Kobalt-, Chrom- und Rhodiumbasen. *Zeitschrift für anorganische Chemie*, **1897,** 14(1), 404-422.

[334] Werner, A.: Über 1.2-Dichloro-tetrammin-kobaltisalze. (Ammoniak-violeosalze). *Berichte der Deutschen Chemischen Gesellschaft*, **1907,** 40(4), 4817-4825.

[335] Hancock, M. P.; Josephsen, J.; Schaffer, C. E.: Bis(2,2'-bipyridine) and bis(1,10-phenanthroline) complexes of chromium(III) and cobalt(III). *Acta Chemica Scandinavica Ser. A*, **1976,** A30(2), 79-97.

[336] Fehrmann, K. R. A.; Garner, C. S.: Synthesis and Spectrum of *cis*-Difluoro-bis-(ethylenediamine)-chromium(III) Iodide. *Journal of the American Chemical Society*, **1960,** 82(24), 6294-6296.

[337] Vaughn, J. W.; Krainc, B. J.: The Synthesis and Resolution of *cis*-Difluorobis(ethylenediamine)chromium (III) Iodide. *Inorganic Chemistry*, **1965,** 4(7), 1077-1080.

[338] Pedersen, E.: Preparation of Bis- and Tris(diamine)chromium(III) Complexes via Dimethyl Sulfoxide and Dimethylformamide Complexes. The Novel Series of *cis*-Bis(trimethylene-diamine)- and *cis*-Bis[(-)trans-1,2-cyclohexane-Diamine]chromium(III) Complexes. *Acta Chemica Scandinavica* **1970,** 24, 3362-3372.

[339] Vaughn, J. W.; Coward, L. C.; Winter, B. K.: Fluoro-Containing Complexes of Chromium(III). II. The Reaction of Sodium Tetrafluoroethylenediaminechromate(III) Monohydrate with Ethylenediamine. *Inorganic Chemistry*, **1966,** 5(11), 2061-2063.

[340] Vaughn, J. W.; Stvan, O. J.; Magnuson, V. E.: Fluoro-containing complexes of chromium(III). III. The synthesis and characterization of some fluoroacidobis(ethylenediamine)chromium(III) complexes. *Inorganic Chemistry*, **1968,** 7(4), 736-741.

[341] Costachescu, N.: *Annales Scientifiques de l´Universite de Jassy*, **1913,** 7, 87-100.

[342] Chrom. In *Gmelins Handbuch der anorganischen Chemie*, Verlag Chemie: Weinheim/Bergstr., 1965; Vol. Col. Teil C Chrom, pp 211-212.

[343] Choi, J.-H.; Oh, I.-G.; Ryoo, K. S.; Lim, W.-T.; Park, Y. C.; Habibi, M. H.: Structural and spectroscopic properties of *trans*-difluoro(1,4,8,12-tetraazacyclopentadecane)chromium(III) perchlorate hydrate. *Spectrochimica Acta Part A: Molecular and Biomolecular Spectroscopy*, **2006,** 65(5), 1138-1143.

[344] Glerup, J.; Schäffer, C. E.: Chroimium(III) Complexes of the *trans*-Tetraammine Series. *Inorganic Chemistry*, **1976,** 15(6), 1408.

[345] Brenčič, J. V.; Čeh, B.; Leban, I.: Fluorine containing coordination compounds of Cr(III). *Monatshefte für Chemie / Chemical Monthly*, **1981,** 112(12), 1359-1368.

[346] Coonnelly, N. G.; Damhus, T.; Hartshorn, R. M.; Hutton, A. T., *Nomenclature of Inorganic Chemistry, IUPAC recommendation 2005*. RCS publisching: Cambridge, UK, 2005.

[347] Delaver, M.; Staples, P. J.: The aquation of *trans*-bis(ethylenediamine)difluorochromium(III) and related ions in aqueous solutions of strong acids. *Journal of the Chemical Society, Dalton Transactions*, **1981,** (4), 981-985.

[348] Fehrmann, K. R. A.; Garner, C. S.: Kinetics of Hydrolysis of *cis*-Difluoro-bis-(ethylenediamine)-chromium(III) Cation. *Journal of the American Chemical Society*, **1961,** 83(6), 1276-1279.

[349] DeJovine, J. M.; Mason, W. R.; Vaughn, J. W.: Acid hydrolysis of *trans*-difluoro-, *trans*-fluorochloro-, and *trans*-fluorobromobis(trimethylenediamine)chromium(III), *trans*-difluoro-, and *trans*-fluorochlorobis(ethylenediamine)chromium(III) cations. *Inorganic Chemistry*, **1974,** 13(1), 66-73.

[350] Kirk, A. D.; Namasivayam, C.; Ward, T.: Photoaquation of trans-difluorobis(1,3-propanediamine)chromium(1+) in acidic aqueous solution. *Inorganic Chemistry*, **1986,** 25(13), 2225-2229.

[351] Kane-Maguire, N. A. P.; Wallace, K. C.; Speece, D. G.: Synthesis, characterization, and photobehavior of macrocyclic difluoro complexes of chromium(III). *Inorganic Chemistry*, **1986,** 25(26), 4650-4654.

[352] Chung, J.-J.; Kim, H.-T.; Bek, S.-O.: Pressure Effect on the Aquation of *trans*-$[Cr(tmd)_2F_2]^+$ and *trans*-$[Cr(tmd)_2FCl]_+$ Ions. *Journal of the Korean Chemical Society* **1989,** 33(2), 164-167.

[353] Vagnini, M. T.; Rutledge, W. C.; Hu, C.; VanDerveer, D. G.; Wagenknecht, P. S.: Effects of steric constraint on chromium(III) complexes of tetraazamacrocycles, 4: Comparison of the *trans*-difluoro-complexes of tet a, 1,4-C_2-cyclam, and 1,11-C_3-cyclam. *Inorganica Chimica Acta*, **2007,** 360(5), 1482-1492.

[354] Vaughn, J. W.; DeJovine, J. M.; Seiler, G. J.: Fluoro-containing complexes of chromium(III). IV. Reactions of *trans*-fluoroaquobis(ethylenediamine)-chromium(III)

perchlorate monohydrate with ammonium chloride and ammonium bromide. *Inorganic Chemistry*, **1970,** 9(3), 684-685.

[355] Vaughn, J. W.; Yeoman, A. M.: Fluoro-containing complexes of chromium(III). 7. Isolation and some reactions of the *cis*-fluoroaquobis(ethylenediamine)chromium(III) cation. *Inorganic Chemistry*, **1976,** 15(9), 2320-2322.

[356] Vaughn, J. W.; Seiler, G. J.: Fluoro-containing complexes of chromium(III). 9. Resolution and some anation reactions of the *cis*-fluoroaquobis(ethylenediamine)chromium(III) cation. *Inorganic Chemistry*, **1979,** 18(6), 1509-1511.

[357] Díaz, C.; Seguí, A.; Ribas, J.; Solans, X.; Font-Altaba, M.; Solans, A.; Casabó, J.: Systematic synthesis and characterization of the derivative complexes of the tetrafluoroaminechromate(III) Anions. — Crystal structure of the (en-H_2)[CrF_4(en)]Cl. *Transition Metal Chemistry*, **1984,** 9(12), 469-473.

[358] Posey, F. A.; Taube, H.: The Mechanisms of Substitution Reactions of Octahedral Complexes: The Induced Aquation of the Halogenopentamminecobaltic Ions by Metal Cations. *Journal of the American Chemical Society*, **1957,** 79(2), 255-262.

[359] Clark, H. R.; Jones, M. M.: Ligand substitution catalysis via hard acid-hard base interaction. *Journal of the American Chemical Society*, **1970,** 92(4), 816-822.

[360] Andersen, P.; Josephsen, J.; Nord, G.; Schaeffer, C. E.; Tranter, R. L.: Configurational correlations of *cis*-bis(2,2'-bipyridine) and *cis*-bis(1,10-phenanthroline) complexes of trivalent metals. *Journal of the Chemical Society D: Chemical Communications*, **1969,** (8), 408-409.

[361] McCann, S.; McCann, M.; Casey, M. T.; Jackman, M.; Devereux, M.; McKee, V.: Syntheses and X-ray crystal structures of *cis*-[Mn(bipy)$_2$$Cl_2$]·2$H_2$O·EtOH and *cis*-[Mn(phen)$_2$$Cl_2$] (bipy = 2,2′-bipyridine; phen = 1,10-phenanthroline); catalysts for the disproportionation of hydrogen peroxide. *Inorganica Chimica Acta*, **1998,** 279(1), 24-29.

[362] Choudhury, S. R.; Dutta, A.; Mukhopadhyay, S.; Lu, L.-P.; Zhu, M.-L.: *cis*-(2,2'-Bipyridyl)dichloromanganese(II)-thiourea (1/1). *Acta Crystallographica Section E*, **2006,** 62(7), m1489-m1491.

[363] Chen, X. M.; Shi, K. L.; Mak, T. C. W.; Luo, B. S.: Aquabis(2,2'-bipyridine)chloromanganese(II) Perchlorate. *Acta Crystallographica Section C*, **1995,** 51(3), 358-361.

[364] Hwang, I.-C.; Ha, K.: Crystal structure of bis(2,2'-bipyridine-N,N')dibromomanganese(II), $MnBr_2(C_{10}H_8N_2)_2$. *Zeitschrift für Kristallographie - New Crystal Structures* **2007,** 222 (3), 209-210.

[365] Hey, E.; Weller, F.; Simon, B.; Becker, G.; Dehnicke, K.: Über die Reaktion von 2,2-Dimethylpropylidinphosphan mit Molybdänpentachlorid; die Kristallstruktur von $[Mo_2Cl_6(\alpha,\alpha'\text{-Dipyridyl})_3]$. *Zeitschrift für anorganische und allgemeine Chemie*, **1983,** 501(6), 61-68.

[366] Helberg, L. E.; Orth, S. D.; Sabat, M.; Harman, W. D.: Coordination Chemistry of Low-Valent Rhenium Polypyridyl Complexes: Synthesis, Reactivity, and Electrochemistry. *Inorganic Chemistry*, **1996,** 35(19), 5584-5594.

[367] I. Strenger, T. R. a. M. N.; : Refinement of the crystal structure of *cis*-bis(2,2'-bipyridyl)dichlorocobalt(III) chloride dihydrate, $[C_{20}H_{16}N_4CoCl_2]Cl\cdot 2H_2O$. *Zeitschrift für Kristallographie - New Crystal Structures*, **2000,** 215(4), NCS 137678.

[368] Eggleston, D. S.; Goldsby, K. A.; Hodgson, D. J.; Meyer, T. J.: Structural variations induced by changes in oxidation state and their role in electron transfer. Crystal and molecular structures of *cis*-$[Ru(bpy)_2Cl_2]\cdot 3.5H_2O$ and *cis*-$[Ru(bpy)_2Cl_2]Cl\cdot$2H2O. *Inorganic Chemistry*, **1985,** 24(26), 4573-4580.

[369] Lahuerta, P.; Latorre, J.; Martinez-Manez, R.; Garcia-Granda, S.; Gomez-Beltran, F.: Structure of bis(2,2'-bipyridine)dichlororhodium(III) chloride dihydrate. *Acta Crystallographica Section C*, **1991,** 47(3), 519-522.

[370] Fontaine, F.: *cis*-Bis(2,2-bipyridine)dichloronickel(II) methanol solvate. *Acta Crystallographica Section E*, **2001,** 57(7), m270-m271.

[371] Rodriguez-Martin, Y.; Gonzalez-Platas, J.; Ruiz-Perez, C.: Diaquabis(2,2'-bipyridine-*N,N'*)nickel(II) diperchlorate. *Acta Crystallographica Section C*, **1999,** 55(7), 1087-1090.

[372] Zhu, J.-W.; Yang, E.; Song, X.-C.; Lin, Y.-D.: Bis(2,2'-bipyridine-κ^2*N,N'*)dibromidocadmium(II). *Acta Crystallographica Section E*, **2007,** 63(4), m1044-m1045.

[373] Guo, H.-X.; Lin, H.-B.; Wang, Q.-H.: *cis*-Bis(2,2'-bipyridine)diiodocadmium(II). *Acta Crystallographica Section E*, **2006,** 62(6), m1239-m1240.

[374] Kavitha, S. J.; Panchanatheswaran, K.; Low, J. N.; Glidewell, C.: Racemic *cis*-bis(2,2'-bipyridyl)difluorovanadium(III) tetrafluoroborate. *Acta Crystallographica Section E, Structure Reports*, **2005,** E61(10), m1965-m1967.

[375] Emsley, J.; Arif, M.; Bates, P. A.; Hursthouse, M. B.: Structure of μ-fluoro-bis[bis(2,2'-bipyridyl)fluoronickel(II)] fluoride-ethanol (1/2) trihydrate, revealing several unique features including strong hydrogen bonds between fluoride ions and ethanol molecules. *Journal of the Chemical Society, Dalton Transactions*, **1989,** (7), 1273-1276.

[376] Brenčič, J. V.; Leban, I.: Fluorine Containing Coordination Compounds of Cr^{III}. I. Crystal and Molecular Structure of *trans*-$[Cr(en)_2F_2]ClO_4$. *Zeitschrift für anorganische und allgemeine Chemie*, **1981,** 480(9), 213-219.

[377] Brenčič, J. V.; Leban, I.; Polanc, I.: Fluoro-containing coordination compounds of chromium(III). V. Preparation and crystal structure of the Racemic *cis*-$[Cr(en)_2F_2]ClO_4 \cdot NaClO_4 \cdot H_2O$. *Zeitschrift für anorganische und allgemeine Chemie*, **1987,** 551(8), 109-115.

[378] Vaughn, J. W.: Fluoro-containing complexes of chromium (III). II. Crystal structure of *trans*-difluorodiaquo-ethylenediaminechromium(III) chloride. *Journal of Chemical Crystallography*, **1983,** 13(3), 231-239.

[379] Vaughn, J. W.; Rogers, R. D.: Structure of *trans*-difluorobis(1,3-propanediamine)chromium(III) perchlorate, *trans*-$[Cr(N_2C_3H_{10})_2F_2](ClO_4)$. *Journal of Chemical Crystallography*, **1985,** 15(3), 281-287.

[380] Bang, E.; Pedersen, E.: The Crystal Structure of *trans*-Difluoro(1,4,8,11-tetraazaundecane)chromium(III) Perchlorate. *Acta Chemica Scandinavica* **1978,** 33A, 297-304.

[381] Liu, H.-X.: Aqua(2,2'-bipyridine)trifluoridochromium(III) dihydrate. *Acta Crystallographica Section E, Structure Reports*, **2009,** E65(9), m1093-m1093.

[382] Thoma, S. G.; Bonhomme, F.; Nyman, M.; Rodriguez, M. A.; Nenoff, T. M.: Synthesis and crystal structure of guanidinium tetrafluoro(*trans-*)dipyridinechromate(III), $[CrF_4(C_5H_5N)_2]^- \cdot CN_3H_6^+$. *Journal of Fluorine Chemistry*, **2001,** 108(1), 73-77.

[383] Il'in, E. G.; Kovalev, V. V.; Aleksandrov, G. G.; Sergeev, A. V.: Molecular Complex of Hafnium Tetrafluoride $HfF_4(\alpha,\alpha'\text{-dipy})_2$ *Doklady Chemistry*, **2004,** 398(4), 200-203.

[384] Arnaudet, L.; Bougon, R.; Ban, B.; Lance, M.; Navaza, A.; Nierlich, M.; Vigner, J.: Structure of the new fluoro complex of tungsten(VI): $[WF_4(bipy)_2]^{2+} \cdot 2[W_2O_2F_9]^- \cdot 0.25HF$ (bipy = 2,2'-bipyridyl). *Journal of Fluorine Chemistry*, **1992,** 59(1), 141-152.

[385] Arnaudet, L.; Bougon, R.; Ban, B.; Lance, M.; Navaza, A.; Nierlich, M.; Vigner, J.: 2,2'-Bipyridyl fluoro complexes of tungsten(VI): preparation, characterization and crystal structure of $[WF_4(bipy)_2]^{2+} \cdot 2[WF_7]^- \cdot WF_6$ and $[WF_4(bipy)_2]^{2+} \cdot 2[WF_7]^- \cdot CH_3CN$; preparation and characterization of WF_6 bipy. *Journal of Fluorine Chemistry*, **1994,** 67(1), 17-25.

[386] Casellato, U.; Graziani, R.; Maccarrone, G.; di Bilio, A. J.: Crystal structure of *cis*-diaqua bis(2,2′-bipyridyl)chromium(III) nitrate, $[Cr(bipy)_2(H_2O)_2]\ (NO_3)_3$. *Journal of Chemical Crystallography*, **1986,** 16(5), 695-702.

[387] Parker, O. J.; Breneman, G. L.: *cis*-Chloro(isothiocyanato)bis(1,10-phenanthroline)copper(II), *cis*-[CuCl(NCS)(phen)$_2$]. *Acta Crystallographica Section C*, **1995,** 51(8), 1529-1531.

[388] Jeffrey, G. A., *An Introduction to Hydrogen Bonding*. Oxford University Press: New York, Oxford, 1997.

[389] Ardon, M.; Bino, A.; Michelsen, K.: Olation and structure. *Journal of the American Chemical Society*, **1987,** 109(7), 1986-1990.

[390] Ardon, M.; Bino, A.; Michelsen, K.; Pedersen, E.: Long-distance magnetic exchange between chromium(III) atoms bridged by $H_3O_2^-$ ligands. *Journal of the American Chemical Society*, **1987,** 109(19), 5855-5856.

[391] Miller, J. D.; Prince, R. H.: The proton magnetic resonance spectra of some bis-o-phenanthroline-cobalt(III) complexes. *Journal of the Chemical Society A: Inorganic, Physical, Theoretical*, **1969**, 519-520.

[392] Al-Noaimi, M.; Haddad, S. F.: *cis*-Bis(2,2'-bipyridyl)dichloridoruthenium(II) dichloromethane solvate. *Acta Crystallographica Section E*, **2007,** 63(9), m2332.

[393] Klufers, P.; Zangl, A.: *trans*-Bis(2,2'-bipyridine)dichloridoruthenium(II). *Acta Crystallographica Section E*, **2007,** 63(12), m3088.

[394] Josephsen, J.; Schäffer, C. E.: Oxygenbridged Binuclear Chromium(III) Complexes with 2,2'-Bipyridine and 1,10-Phenanthroline. *Acta Chemica Scandinavica* **1970,** 24, 2929-2942.

[395] Jörgensen, S. M.: Beiträge zur Chemie der Chromammoniakverbindungen. X. Ueber die Rhodosochromsalze. *Journal für praktische Chemie*, **1892,** 45(1), 260-274.

[396] Bang, E.; Narasimhayya, T.: The Crystal Structure of Di-μ-hydroxotetrakis-μ-hydroxobis(tetraminechromium(III)) bis(diamminechromium(III)) Bromide Dihydrate. *Acta Chemica Scandinavica* **1970,** 24, 275-284.

[397] Pfeiffer, P.: Zur Stereochemie des Chroms VI. Über mehrkernige Chromsalze. *Zeitschrift für anorganische Chemie*, **1908,** 58(1), 272-296.

[398] Ardon, M.; Bino, A.: Hydrogen oxide bridging ligands in a classical coordination compound. *Journal of the American Chemical Society*, **1983,** 105(26), 7747-7748.

[399] Ardon, M.; Bino, A.: Role of the H_3O_2 bridging ligand in coordination chemistry. 1. Structure of hydroxoaquametal ions. *Inorganic Chemistry*, **1985,** 24(9), 1343-1347.

[400] Goodson, P. A.; Glerup, J.; Hodgson, D. J.; Michelsen, K.; Rychlewska, U.: Magnetic exchange through hydrogen bonds: structural and magnetic characterization of cis-hydroxoaquachromium(III) complexes of tetradentate and monodentate ligands. *Inorganic Chemistry*, **1994,** 33(2), 359-366.

[401] Darensbourg, Donald J.; Frost, Brian J.; Larkins, David L.; Reibenspies, Joseph H.: Organometallic Complexes of Uracil and Orotic Acid Derivatives: Coordination Mode, Structure, and Reactivity. *European Journal of Inorganic Chemistry*, **2000,** 2000(12), 2487-2495.

[402] Werndrup, P.; Seisenbaeva, G. A.; Westin, G.; Persson, I.; Kessler, V. G.: A Single-Source-Precursor Approach to Late Transition Metal Molybdate Materials: The Structural Role of Chelating Ligands in the Formation of Heterometallic Heteroleptic Alkoxide Complexes. *European Journal of Inorganic Chemistry*, **2006,** 2006(7), 1413-1422.

[403] Samuel, E.; Harrod, J. F.; Gourier, D.; Dromzee, Y.; Robert, F.; Jeannin, Y.: Zero field splitting and exchange interactions in bis(cyclopentadienyl)titanium(III) dinuclear compounds with short metal-metal distances: synthesis, x-ray structure, and EPR spectroscopy of methoxo- and ethoxo-bridged dimers. *Inorganic Chemistry*, **1992,** 31(15), 3252-3259.

[404] Wang, J.; Tong, M.-L.: Di-μ-methoxo-bis[dipyridinecopper(II)] diperchlorate. *Acta Crystallographica Section E*, **2004,** 60(9), m1223-m1224.

[405] Bardwell, D. A.; Horsburgh, L.; Jeffery, J. C.; Joulie, L. F.; Ward, M. D.; Webster, I.; Yellowlees, L. J.: Dinuclear alkoxide-bridged ruthenium(II) complexes with class III mixed-valence states: a structural and spectroelectrochemical study. *Journal of the Chemical Society, Dalton Transactions*, **1996,** (12), 2527-2531.

[406] Fischer, H. R.; Glerup, J.; Hodgson, D. J.; Pedersen, E.: Structural and magnetic characterization of the alkoxo-bridged chromium(III) dimer bis(μ-methoxo)bis[bis(2,4-pentanedionato)chromium(III)], $[(acac)_2Cr(OCH_3)]_2$. *Inorganic Chemistry*, **1982,** 21(8), 3063-3066.

[407] Glerup, J.; Hodgson, D. J.; Pedersen, E.: A Novel Correlation between Magnetism and Structural Parameters in Superexchange Coupled Chromium(III) Dimers. *Acta Chemica Scandinavica* **1983,** 37A(2), 161-164.

[408] Dreiser, J.; Pedersen, K. S.; Piamonteze, C.; Rusponi, S.; Salman, Z.; Ali, M. E.; Schau-Magnussen, M.; Thuesen, C. A.; Piligkos, S.; Weihe, H.; Mutka, H.; Waldmann, O.; Oppeneer, P.; Bendix, J.; Nolting, F.; Brune, H.: Direct observation of a ferri-to- ferromagnetic transition in a fluoride-bridged 3d-4f molecular cluster. *Accepted for publication in Chemical Science*, **2011**.

[409] The Aberdeen Bestiary In Aberdeen University Library Aberdeen, 1200.

[410] Libavius, A., *Alchymia Andreae Libavii, recognita, emendata, et aucta, tum dogmatibus et experimentis nonnullis : tum commentario medico physico chymico : qui exornatus est variis instrumentorum chymicorum picturis, partim aliunde translatis, partim planè novis : in gratiam eorum, qui arcanorum naturalium cupidi, on absque involucris elementarium et aenigmaticarum sordium, intueri gaudent.* 1 ed.; Petrus Kopff: Frankfurt: , 1606

[411] Arnold, D. A.: *Chemistry in Britain*, **1981**, 551

[412] Thuesen, C. A. Fluoride-bridged chromium(III)-lanthanoide(III) clusters - High-temperature expansion for finite spin cluster - Magnetic characterization of iron-containing zeolites. University of Copenhagen, Copenhagen, 2011.

[413] Pedersen, K. S. Variations in Molecule-Based Magnetism Vertically and Horizontally in The Periodic Table. University of Copenhagen, Copenhagen, 2011.

[414] Quack, M.; Stohner, J.; Strauss, H. L.; Takami, M.; Thor, A. J.; Cohen, E. R.; Cvitas, T.; Frey, J.; Holström, G. B.; Kuchitsu, K.; Marquardt, R.; Mills, I.; Pavese, F., *Quantities, Units and Symbols in Physical Chemistry*. 3 ed.; RSC Publishing: Cambridge, UK, 2007.

[415] *Le Système international d'unités (SI) The International System of Units (SI)*; Bureau International des Poids et Mesures: 2006.

[416] Aubagnac, J. L.: Use of *m*-Nitrobenzyl Alcohol as a Matrix in Fast-atom Bombardment Negative-ion Mass Spectrometry of Polar Compounds. *Rapid Communications in Mass Spectrometry*, **1990,** 4(4), 114-116.

[417] Azuah, R. T.; Kneller, L. R.; Qiu, Y.; Tregenna-Piggott, P. L. W.; Brown, C. M.; Copley, J. R. D.; Dimeo, R. M.: DAVE: A comprehensive software suite for the reduction, visualization, and analysis of low energy neutron spectroscopic data. *Journal of research of the National Institute of Standards and Technology*, **2009,** 114, 341.

[418] Pascal, P.: Recherches Magnétochimiques. *Annales de Chimie et de Physique*, **1910,** 19, 5-70.

[419] Bain, G. A.; Berry, J. F.: Diamagnetic Corrections and Pascal's Constants. *Journal of Chemical Education*, **2008,** 85(4), 532.

[420] Coppens, P., Crystallographic Computing. In Ahmed, F. R.; Hall, S. R.; Huber, C. P., Eds. Munksgaard: Copenhagen, 1970; pp 255-270.

[421] Nonius *COLLECT.*, Nonius BV: Delft, The Netherlands, 1996.

[422] Duisenberg, A. J. M.; Kroon-Batenburg, L. M. J.; Schreurs, A. M. M.: An intensity evaluation method: EVAL-14T. *J. Appl. Cryst.*, **2003,** 36, 220-229.

[423] Sheldrick, G. M.: A short history of SHELX. *Acta Crystallographica Section A*, **2008,** A64, 112-122.

[424] van, J.: Searching the Cambridge Structural Database for the 'best' representative of each unique polymorph. *Acta Crystallographica Section B*, **2006,** 62(4), 567-579.

[425] Taylor, R.; Macrae, C. F.: Rules governing the crystal packing of mono- and dialcohols. *Acta Crystallographica Section B*, **2001,** 57(6), 815-827.

[426] Bruno, I. J.; Cole, J. C.; Edgington, P. R.; Kessler, M.; Macrae, C. F.; McCabe, P.; Pearson, J.; Taylor, R.: New software for searching the Cambridge Structural Database and visualizing crystal structures. *Acta Crystallographica Section B*, **2002,** 58(3 Part 1), 389-397.

[427] Macrae, C. F.; Edgington, P. R.; McCabe, P.; Pidcock, E.; Shields, G. P.; Taylor, R.; Towler, M.; van de Streek, J.: Mercury: visualization and analysis of crystal structures. *Journal of Applied Crystallography*, **2006,** 39(3), 453-457.

[428] Macrae, C. F.; Bruno, I. J.; Chisholm, J. A.; Edgington, P. R.; McCabe, P.; Pidcock, E.; Rodriguez-Monge, L.; Taylor, R.; van de Streek, J.; Wood, P. A.: Mercury CSD 2.0 - new features for the visualization and investigation of crystal structures. *Journal of Applied Crystallography*, **2008,** 41(2), 466-470.

PART II: PUBLICATIONS

GENERAL COMMENTS

The following list summarizes the scientific papers that have been based on parts of the work presented in this thesis. The papers have all been published, accepted or submitted for publication in international journals with peer review.
Besides the main topic work has there also been carried out on smaller side projects. The result of these have not been subject for the thesis, but the publications relating to these are listed at the end as “other publications”. The two lists are ordered chronologically and given DOI number in the cases where it is assigned, in order to allow easy web-based literature search. All articles are also attached to the thesis in their full length. However, supporting material is included only in those cases where it was deemed appropriate for the thesis (*e.g.* synthetic procedures, magnetic measurements, but not crystallographic information files and general computer files)

List of publication related to thesis

- Scheifele, Q., Birk, T., Bendix, J., Tregenna-Piggott, P. L., Weihe, H.:
  Superhyperfine Interaction in $[MnF_6]^{3-}$.
  Angew. Chem. Int. Ed., **2008**, 47(1), 148-150. DOI: 10.1002/anie.200703771

- Birk, T., Bendix, J., Weihe, H.:
  cis-Difluoridobis(1,10-phenanthroline)chromium(III) perchlorate monohydrate.
  Acta Cryst., **2008**, E64, m369–m370. DOI: 10.1107/S1600536808001153

- Birk, T., Bendix, J.:
  cis-Aquabis(2,2′-bipyridine-κ^2-*N,N′*)-fluoridochromium(III) bis(perchlorate) dihydrate.
  Acta Cryst., **2010**, E66, m121-m122. DOI: 10.1107/S1600536810000127

- Birk, T., Magnussen, M., Piligkos, S., Weihe, H., Bendix, J.:
  Alkali metal cation complexation and solvent interactions by robust chromium(III) fluoride complexes.
  J. Fluorine Chem., **2010**, 131, 898-906. DOI: 10.1016/j.jfluchem.2010.06.003.

- Birk, T., Pedersen, K. S., Piligkos, S., Thuesen, C., Weihe, H., Bendix, J.:
  Magnetic properties of a manganese(III) chain with mono-atomic bridges: *catena*-Mn(F)(salen).
  Inorg. Chem., **2011**, 50(12), 5312–5314. DOI: 10.1021/ic2002699

- Birk, T., Schau-Magnussen, M., Weyhermüller, T., Bendix, J.:
  cyclo-Tetra-μ-fluorido-1:2κ^2*F*;2:3κ^2*F*;3:4κ^2*F*;1:4κ^2*F*-octanitrato-1κ^8*O*,*O*';3κ^8*O*,*O*'-tetrakis(1,10-phenanthroline)-2κ^4*N*,*N*';4κ^4*N*,*N*'-2,4-dichromium(III)-1,3-dineodymium(III) methanol tetrasolvate monohydrate.
  Acta Cryst., **2011**, E67, m1561-m1562. DOI: 10.1107/S1600536811042383

- Birk, T., Pedersen, K. S., Thuesen, C. Aa, Weyhermüller, T., Schau-Magnussen, M., Piligkos, S., Weihe, H., Mossin, S., Evangelisti, M., Bendix, J.:
  Fluoride Bridges as Structure-Directing Motifs in 3d-4f Cluster Chemistry
  Inorg. Chem., **2012**, 51(9). 5435–5443. DOI: 10.1021/ic034777f

- Dreiser, J., Pedersen, K. S., Birk, T., Schau-Magnussen, M., Piamonteze, C., Rusponi, S., Weyhermüller, T., Brune, H., Nolting, F., Bendix. J.:
  XMCD Study of a Methoxide-Bridged Dy^{III}–Cr^{III} Cluster Obtained by Fluoride Abstraction from *cis*-$[Cr^{III}F_2(phen)_2]^+$.
  Submitted to *The Journal of Physical Chemistry*.

List of other publications

- Birk, T., Weinberger, P., Mereiter, K.:
  1,3-bis(1H-tetrazol-1-ylmethyl)-benzene.
  Cambridge Crystallographic Data Centre (CCDC), **2008**, 700671.

- Birk, T., Weihe, H.:
  The First Coordination Compound with 5-methylisoxazole-3-carboxylat: Synthesis and Structural Characterization of $[Cu(L_2)(H_2O)]\cdot H_2O$.
  J. Chem. Crystallogr., **2009**, 39(10), 766-771. DOI: 10.1007/s10870-009-9563-7.

- Birk, T.:
  Matrix Formulation of Complex Equilibria and Acid-Base Equilibria by use of the Extent of Reaction.
  Chem. Educator, **2009**, 14(3), 91-95. DOI 10.1333/s00897092206a

PAPER 1

Superhyperfine Interaction in $[MnF_6]^{3-}$

Quirin Scheifele, Torben Birk, Jesper Bendix, Philip L. W. Tregenna-Piggott and Høgni Weihe

Angew. Chem. Int. Ed., 2008, 47(1), 148-150

EPR Spectroscopy

DOI: 10.1002/anie.200703771

Superhyperfine Interaction in $[MnF_6]^{3-}$

Quirin Scheifele, Torben Birk, Jesper Bendix, Philip L. W. Tregenna-Piggott,* and Høgni Weihe**

The modification of the EPR response by the superhyperfine interaction is the primary source of information from which metal-ligand bonding characteristics of paramagnetic centers are determined. The plethora of techniques developed to elucidate this quantity have found particular application in the field of biochemistry, and enzymes containing copper(II), iron(III), and manganese(II) centers are regularly probed by pulsed EPR techniques. Owing to the importance of monomeric manganese(III) centers to biocoordination chemistry in metalloenzymes such as superoxide dismutases,[1] and to processes such as catalytic epoxidation[2] and aziridination,[3] the spectroscopic properties of this center have been thoroughly investigated. Nevertheless, there are no reports of superhyperfine interaction in monomeric manganese(III) complexes. Herein, we show that the manganese(III)–fluorine superhyperfine interaction can be observed by using a conventional spectrometer equipped with a parallel-mode X-band cavity. Data from two systems are presented from which principal values of the superhyperfine tensor in the hexafluoromanganate(III) anion are determined. The geometry inferred from the superhyperfine coupling constants does not correlate simply with the Mn–F bond lengths. Apart from this being the first observation of superhyperfine interactions in manganese(III) complexes, this paper provides a textbook example of how the electronic structure of the central ion is reflected in the superhyperfine interaction with the surroundings.

The salt $[Rh(NH_3)_6][GaF_6]_{(1-x)}[MnF_6]_x$ was synthesized according to reference [4], based on the method for the synthesis of $[Rh(NH_3)_6][MnF_6]$ reported by Wieghardt and Siebert.[5] At temperatures below 140 K, the high-frequency high-field EPR spectra of salts with $0.05 \leq x \leq 1$, as well as temperature-dependent inelastic neutron scattering (INS) spectra of the fully deuterated concentrated salt, could be interpreted in terms of the spin Hamiltonian for an $S=2$ spin system [Eq. (1)] with $g_x=g_y=1.993$, $g_z=1.980$, and $D=-3.968\ cm^{-1}$.

[*] T. Birk, Prof. J. Bendix, Dr. H. Weihe
Department of Chemistry, University of Copenhagen
Universitetsparken 5, 2100 Copenhagen (Denmark)
Fax: (+45) 3532-0133
E-mail: bendix@kiku.dk
weihe@kiku.dk

Q. Scheifele
Departement für Chemie und Biochemie, Universität Bern
Freiestrasse 3, 3000 Bern (Switzerland)

Dr. P. L. W. Tregenna-Piggott
Laboratory for Neutron Scattering, ETHZ and Paul Scherrer Institute
5232 Villigen PSI (Switzerland)
E-mail: philip.tregenna@psi.ch

$$\hat{H} = \mu_B(g_x B_x \hat{S}_x + g_y B_y \hat{S}_y + g_z B_z \hat{S}_z) + D\hat{S}_z^2 \quad (1)$$

The sign of the D parameter and the fact that $g_z < g_x, g_y$ are both consistent with an axially elongated[6] structure of the anion. Hence, the $S=2$ ground state is split, and the $M_S=\pm 2$ components are left as the lowest energetic states, separated by approximately $12\ cm^{-1}$ from the $M_S=\pm 1$ components, which in turn are $4\ cm^{-1}$ lower than the $M_S=0$ component.

The X-band parallel-mode spectrum of the $[MnF_6]^{3-}$ anion as a 0.1% dopant in $[Rh(NH_3)_6][GaF_6]$ (Figure 1)

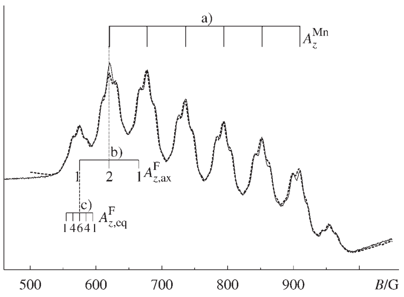

Figure 1. EPR spectrum of finely ground $[Rh(NH_3)_6][GaF_6]_{(1-x)}[MnF_6]_x$ with $x=0.001$. The experimental spectrum (solid line) was obtained at $T=1.5$ K with $B_1 \parallel B$ and $\nu=9.31771$ GHz. The dotted line represents the best fit from Equations (2) and (3) with the parameters given just after Equation (3). a) Illustration of the splitting of the six Mn hyperfine lines. b) Each of these is split into three lines by strong interaction with the two axial fluoride ligands; see also Figure 2. c) Each of these lines is finally split by interaction with the four equatorial fluoride ligands into five lines.

shows a complicated pattern arising from the combination of Zeeman, hyperfine, and superhyperfine splittings. The spectrum in Figure 1 could be confidently interpreted only after having recorded a spectrum of the $[Mn(salen)F_2]^-$ anion (Figure 2), which exhibits eight lines with approximate relative intensities of 1:3:4:4:4:4:3:1. These eight lines are consistent with six lines resulting from the hyperfine interaction involving the Mn nucleus ($I^{Mn}=5/2$), split further by the presence of two fluoride ligands ($I^F=1/2$). Note that the splitting due to the superhyperfine interaction is almost as large as that due to the hyperfine interaction. The spectrum can be satisfactorily reproduced by the effective spin Hamiltonian for a non-Kramers doublet [Eq. (2)] which is valid for the lowest energy $M_S=\pm 2$ electronic components.[7]

$$\hat{H} = \Delta \hat{S}_x + \mu_B g'_z B_z \hat{S}_z + A'_z \hat{S}_z \hat{I}_z^{Mn} + A'_{z,ax} \sum_{i=1,2} \hat{S}_z \hat{I}_z^{F,i} \quad (2)$$

 InterScience® © 2008 Wiley-VCH Verlag GmbH & Co. KGaA, Weinheim

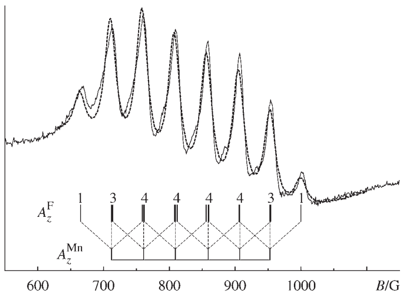

Figure 2. EPR spectrum of $[Mn(salen)F_2]^-$ in frozen *N*-methyl form amide (NMF) glass. The experimental spectrum (solid line) was recorded at 5 K with $B_1 \parallel B$ and $\nu = 9.44157$ GHz. The dotted line represents the best fit from Equation (2) and the parameters given in the text. The same spectrum was obtained from [Mn(salen)Br] and an excess of $(NEt_4)F$ dissolved in NMF.

In Equation (2) Δ is the zero-field splitting of the two levels and g'_z, A', and $A'_{\parallel,ax}$ designate the g factor, the hyperfine coupling constant, and the superhyperfine coupling constant, respectively, for this non-Kramers doublet. Here, and in the following, the subscript z refers to the component parallel to the long Mn–F bond; primed parameters refer to an effective $S = ^1\!/_2$ spin Hamiltonian and are four times as large as the unprimed parameters, which refer to an $S = 2$ spin Hamiltonian.

Fitting a theoretical spectrum based on Equation (2) to the experimental spectrum resulted in the parameters $\Delta = 0.0712(11)\ \text{cm}^{-1}$, $A'_z = 0.0180(3)\ \text{cm}^{-1}$, and $A'_{z,ax} = 0.0169(13)\ \text{cm}^{-1}$. The fact that the superhyperfine interaction with the fluorine nuclei is almost as large as the hyperfine interaction with the manganese nucleus results in the intensity pattern indicated in Figure 2. The fit, superimposed on the experimental spectrum in Figure 2, gives a good reproduction of the experimental data considering that the frozen glass may contain other species in lower concentrations. Furthermore, terms describing unresolved superhyperfine interactions with the salen nitrogen atoms have not been included.

Returning now to the spectrum in Figure 1, we see that the major difference from that in Figure 2 is an additional splitting of the lines producing a pattern with relative intensities of 1:4:6:4:1. The spectrum can be accurately reproduced by Equation (2) augmented with the term (3), which accounts for the interaction between the electronic spin and the equatorial fluoride ligands.

$$A'_{z,eq} \sum_{i=3}^{6} \hat{S}_z \hat{I}_z^{F,i} \tag{3}$$

The experimental data did not warrant the use of different superhyperfine coupling constants for the equatorial fluoride ligands; consequently, they were all set equal.

From a fitting of the experimental spectrum the following parameters were obtained: $\Delta = 0.1308(1)\ \text{cm}^{-1}$, $A'_z = 0.02151(3)\ \text{cm}^{-1}$, $A'_{z,ax} = 0.01622(10)\ \text{cm}^{-1}$, and $A'_{z,eq} = 0.00395(7)\ \text{cm}^{-1}$. The g' factor was fixed to 7.92, obtained as $4g_z$ [see Eq. (1) and the text just after Equation (2)]. Comparison of the experimental and calculated spectra in Figure 1 shows that they are virtually identical, in contrast to the spectra in Figure 2. The better agreement in this case is ascribed to the fact that only one spin system is present here, and that the operator Equations (2) and (3) account for all spins that are present.

The axial component of the manganese hyperfine coupling constant $A_z = A'_z/4 = 0.00538\ \text{cm}^{-1}$ is consistent with that recently reported for the tetragonally elongated hexaaqua ion ($0.0053\ \text{cm}^{-1}$).[8] On the other hand, the superhyperfine coupling constants have magnitudes opposing what could be inferred directly from the coordination geometry, from which we would have expected that $A_{z,ax} < A_{z,eq}$ since $d(\text{Mn–F}_{ax}) > d(\text{Mn–F}_{eq})$. The parameter $A_{z,ax} = A'_{z,ax}/4 = 0.00406\ \text{cm}^{-1}$ describing the interaction with the more distant axial fluoride ligands is more than four times as large as that for the interaction to the equatorial fluoride ligands [Eq. (4); $A_{z,eq} = A'_{z,eq}/4 = 0.00099\ \text{cm}^{-1}$].

$$\left(\frac{A_{z,ax}}{A_{z,eq}}\right)_{exp} \approx 4.1 \tag{4}$$

The ratio can be interpreted in terms of the covalency model outlined in reference [9]; see also reference [10]. Writing the $M_S = 2$ component of the ground state function as $|d^+_{yz} d^+_{zx} d^+_{xy} d^+_{z^2}|$, and using the notation of reference [9], we obtain Equations (5) and (6) for the superhyperfine coupling constants of the axial and equatorial ligands.

$$A_{z,ax} = A_{\parallel,ax} = A_s + 2A_{op} - 2A_t + 2A_d \tag{5}$$

$$A_{z,eq} = A_{\perp,eq} = \frac{1}{4}A_s - \frac{1}{4}A_{op} + A_t - A_d \tag{6}$$

Here, as in reference [9], the subscripts $\parallel$ and $\perp$ refer to parallel and perpendicular to the individual bonds, respectively. The three parameters A_s, A_{op}, and A_t are the contributions from the fluorine 2s, $2p_\sigma$, and $2p_\pi$ orbitals, respectively. The model parameters A_s, A_{op}, and A_t all have the same sign, determined by the product of the electronic g factor and the g_N factor of the ligand nucleus.[9] In the present case this is positive. The contact term A_s provides an isotropic contribution to the superhyperfine interaction. The contribution from the remaining terms is anisotropic.

The term A_d is the contribution from the direct dipole–dipole interaction between the electron spin and the fluorine nucleus. Its magnitude can be estimated by using the classical expression for the interaction energy between two magnetic dipoles separated by the distance R [Eq. (7)].

$$A_d = \frac{\mu_0\, g\, \mu_B\, g_N\, \mu_N}{4\pi R^3} \tag{7}$$

Using the bond lengths for hexaaqua ion in the axially distorted phase, $R_{ax} = 2.06$ Å and $R_{eq} = 1.86$ Å, as an estimate for the bond lengths in the hexafluoro anion, we obtain values of 0.00028 and $0.00039\ \text{cm}^{-1}$ for the axial and equatorial ligands, respectively. Hence, by using Equations (5) and (6)

 © 2008 Wiley-VCH Verlag GmbH & Co. KGaA, Weinheim

the contribution from the direct dipole–dipole interaction to $A_{z,ax}$ and $A_{z,eq}$, being small and almost equal, cannot explain the observed values and observed ratio of Equation (4). Therefore, on the basis of the magnitude of the experimentally determined $A_{z,ax}$ and $A_{z,eq}$ values, we conclude that the main contributions to the observed superhyperfine coupling constants come from A_s, $A_{\sigma p}$, and A_t, and not from the direct dipole–dipole interaction.

Experimentally, we have extracted but two superhyperfine parameters from the experimental spectra. Therefore, we cannot independently deduce the values of A_s, $A_{\sigma p}$, and A_t. By comparison with values obtained for other di- and trivalent hexafluoro complexes we see that $A_s > A_{\sigma p} > A_t > A_d$. The difference $A_{\sigma p} - A_t$ was in all cases found to be small and comparable to A_d. From accurate Q-band EPR measurements[11] on $[FeF_5]^{2-}$ as an impurity in $(NH_4)_2SbF_5$ it was concluded that $A_s \gg A_{\sigma p}, A_t, (A_{\sigma p} - A_t)$. As $A_{\sigma p}$ and A_t appear with opposite signs in both Equations (5) and (6), the superhyperfine coupling constants may be approximated by the first term in these expressions, which results in a theoretical ratio given by Equation (8).

$$\left(\frac{A_{z,ax}}{A_{z,eq}}\right)_{\text{theory}} = 4 \qquad (8)$$

The factor of four in Equation (8) reflects the geometry of the linear combination of ligand orbitals transforming as and contributing to the molecular orbital predominantly being d_{z^2}. In this linear combination, axial and equatorial ligand orbitals occur with relative weights of $+2$ and -1, respectively, and the superhyperfine parameters depend on the squared amplitudes.

The almost exact agreement with the experimentally found ratio of Equation (4) is probably fortuitous, as the superhyperfine interaction reported for $[CuF_6]^{4-}$ is clearly anisotropic.[12] Nevertheless, the experiment yields an unambiguous determination of the electronic and molecular structure of the complex. If the $[MnF_6]^{3-}$ anion were tetragonally compressed, the HOMO would be predominately $d_{x^2-y^2}$, in which case the contribution from the A_s term to $A_{z,ax}$ would be zero.

In summary, we have presented the first EPR spectrum of a manganese(III) complex in which the superhyperfine interaction is clearly resolved. We have shown that this is an important quantity, as the nature of the Jahn–Teller distortion and the concomitant electronic structure can then be deduced from a single EPR spectrum obtained at one temperature.

Received: August 16, 2007
Published online: November 8, 2007

Keywords: EPR spectroscopy · fluorine · manganese · superhyperfine interactions

[1] F. Yamakura, K. Kobayashi, H. Ue, M. Konno, *Eur. J. Biochem.* **1995**, *227*, 700–706; J. P. Renault, C. Verchere-Beaur, I. Morgenstern-Badarau, F. Yamakura, M. Gerloch, *Inorg. Chem.* **2000**, *39*, 2666–2675.
[2] W. Zhang, J. L. Loebach, S. R. Wilson, E. N. Jacobsen, *J. Am. Chem. Soc.* **1990**, *112*, 2801–2803.
[3] T.-S. Lai, C.-M. Che, H.-L. Kwong, S.-M. Peng, *Chem. Commun.* **1997**, 2373–2374.
[4] Preparation of $[Rh(NH_3)_6][GaF_6]_{1-x}[MnF_6]_x$: Three 20-mL 7% aqueous HF solutions A, B, and C were prepared by adding $GaCl_3$ (322 mg, 1.83 mmol), $MnCO_3$ (9.21 mg, 0.08 mmol) followed by 0.02 M $KMnO_4$ (1 mL, 0.02 mmol), and $[Rh(NH_3)_6]Cl_3$ (405 mg, 1.30 mmol), respectively. Solutions A and B were mixed and solution C was added dropwise with immediate precipitation of the product. After cooling in ice, the supernatant was decanted and the microcrystalline light violet product washed with HF (7%), ethanol and diethyl ether. These amounts correspond to Mn/Ga ≈ 5%.
[5] K. Wieghardt, H. Siebert, *Z. Anorg. Allg. Chem.* **1971**, *381*, 12–20.
[6] J. S. Griffith, *The Theory of Transition Metal Ions*, Cambridge University Press, Cambridge, **1961**.
[7] I. Krivokapič, C. Noble, S. Klitgaard, P. L. W. Tregenna-Piggott, H. Weihe, A.-L. Barra, *Angew. Chem.* **2005**, *117*, 3679–3682; *Angew. Chem. Int. Ed.* **2005**, *44*, 3613–3616.
[8] P. L. W. Tregenna-Piggott, H.-P. Andres, G. J. McIntyre, S. P. Best, C. C. Wilson, J. A. Cowan, *Inorg. Chem.* **2003**, *42*, 1350–1365.
[9] A. Abragam, B. Bleaney, *Electron Paramagnetic Resonance of Transition Ions*, Oxford University Press, New York, **1970**. See Chap. 20 for a discussion of covalency and the superhyperfine interaction.
[10] J. Owen, J. H. M. Thornley, *Rep. Prog. Phys.* **1966**, *29*, 675–728.
[11] C. J. Radnell, J. R. Pilbrow, S. Subramanian, M. T. Rogers, *J. Chem. Phys.* **1975**, *62*, 4948–4952.
[12] J. D. Swalen, B. Johnson, H. M. Gladney, *J. Chem. Phys.* **1970**, *52*, 4078–4086.

 © 2008 Wiley-VCH Verlag GmbH & Co. KGaA, Weinheim

PAPER 2

cis-Difluoridobis(1,10-phenanthroline)chromium(III) perchlorate monohydrate

Torben Birk, Jesper Bendix and Høgni Weihe

Acta Cryst., 2008, E64, m369–m370

Acta Crystallographica Section E
Structure Reports Online
ISSN 1600-5368

cis-Difluoridobis(1,10-phenanthroline)-chromium(III) perchlorate monohydrate

Torben Birk,* Jesper Bendix and Högni Weihe

Department of Chemistry, University of Copenhagen, Universitetsparken 5, DK-2100 København Ø, Denmark
Correspondence e-mail: Birk@kiku.dk

Received 5 January 2008; accepted 11 January 2008

Key indicators: single-crystal X-ray study; T = 122 K; mean σ(C–C) = 0.003 Å; R factor = 0.032; wR factor = 0.146; data-to-parameter ratio = 12.2.

The title complex, $[CrF_2(C_{12}H_8N_2)_2]ClO_4 \cdot H_2O$, displays a slightly distorted octahedral coordination geometry around the central chromium(III) ion. The Cr environment is composed of a *cis* arrangement of two 1,10-phenanthroline [average Cr^{III}—N = 2.0726 (10) Å] and two fluoride [average Cr^{III}—F = 1.8533 (6) Å] ligands. The water molecule forms a hydrogen bond to fluorine in a neighbouring cation.

Related literature

For details of the general synthesis of amine-containing difluorido complexes of chromium(III), see: Glerup *et al.* (1970). For the structure of the analogous 2,2′-bipyridine complex, see: Yamaguchi-Terasaki *et al.* (2007). For related literature, see: Brenčič *et al.* (1981, 1987); Delavar & Staples (1981); Kaizaki & Takemoto (1990); Kane-Maguire *et al.* (1986).

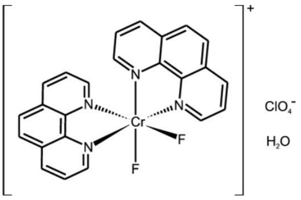

Experimental

Crystal data

$[CrF_2(C_{12}H_8N_2)_2]ClO_4 \cdot H_2O$
M_r = 567.87
Triclinic, $P\overline{1}$
a = 7.6930 (10) Å
b = 9.4640 (8) Å
c = 16.0610 (17) Å
α = 79.750 (7)°
β = 83.228 (12)°
γ = 88.115 (8)°
V = 1142.6 (2) Å^3
Z = 2
Mo $K\alpha$ radiation
μ = 0.68 mm^{-1}
T = 122 (1) K
0.44 × 0.41 × 0.16 mm

Data collection

Nonius KappaCCD area-detector diffractometer
Absorption correction: Gaussian integration (Coppens, 1970)
T_{min} = 0.794, T_{max} = 0.913
28606 measured reflections
4014 independent reflections
3851 reflections with $I > 2\sigma(I)$
R_{int} = 0.025

Refinement

$R[F^2 > 2\sigma(F^2)]$ = 0.031
$wR(F^2)$ = 0.145
S = 1.41
4014 reflections
329 parameters
H-atom parameters constrained
$\Delta\rho_{max}$ = 0.79 e Å^{-3}
$\Delta\rho_{min}$ = −0.51 e Å^{-3}

Table 1
Selected geometric parameters (Å, °).

Cr1—F2	1.8444 (10)	Cr1—N2	2.0607 (15)
Cr1—F1	1.8621 (10)	Cr1—N3	2.0797 (16)
Cr1—N4	2.0566 (15)	Cr1—N1	2.0934 (15)
F2—Cr1—F1	95.92 (5)	N4—Cr1—N3	79.95 (6)
F2—Cr1—N4	92.33 (5)	N2—Cr1—N3	96.36 (6)
F1—Cr1—N4	91.42 (5)	F2—Cr1—N1	170.54 (5)
F2—Cr1—N2	91.83 (6)	F1—Cr1—N1	88.67 (5)
F1—Cr1—N2	91.86 (5)	N4—Cr1—N1	95.83 (6)
N4—Cr1—N2	174.40 (5)	N2—Cr1—N1	79.72 (6)
F2—Cr1—N3	89.38 (5)	N3—Cr1—N1	87.34 (6)
F1—Cr1—N3	170.08 (5)		

Table 2
Hydrogen-bond geometry (Å, °).

D—H···A	D—H	H···A	D···A	D—H···A
O5—H5B···F1^{i}	1.03	1.69	2.7183 (19)	175

Symmetry code: (i) $x - 1, y, z$.

Data collection: *COLLECT* (Nonius, 1999); cell refinement: *COLLECT*; data reduction: *EvalCCD* (Duisenberg *et al.*, 2003); program(s) used to solve structure: *SHELXS97* (Sheldrick, 2008); program(s) used to refine structure: *SHELXL97* (Sheldrick, 2008); molecular graphics: *ORTEP-3* (Farrugia, 1997); software used to prepare material for publication: *SHELXL97*.

The authors are grateful to Mr Flemming Hansen (Centre of Crystallographic Studies, University of Copenhagen) for collection of the X-ray diffraction data.

Supplementary data and figures for this paper are available from the IUCr electronic archives (Reference: WK2075).

References

Brenčič, J. V., Čeh, B. & Leban, I. (1987). *Z. Anorg. Allg. Chem.* **551**, 109–115.
Brenčič, J. V., Leban, I. & Polanc, I. (1981). *Monatsh. Chem.* **112**, 1359–1368.
Coppens, P. (1970). *Crystallographic Computing*, edited by F. R. Ahmed, S. R. Hall & C. P. Huber, pp. 255–270. Copenhagen: Munksgaard.
Delavar, M. & Staples, P. J. (1981). *J. Chem. Soc. Dalton Trans.* pp. 981–985.
Duisenberg, A. J. M., Kroon-Batenburg, L. M. J. & Schreurs, A. M. M. (2003). *J. Appl. Cryst.* **36**, 220–229.
Farrugia, L. J. (1997). *J. Appl. Cryst.* **30**, 565.
Glerup, J., Josephsen, J., Michelsen, K., Pedersen, E. & Schäffer, C. E. (1970). *Acta Chem. Scand.* **24**, 247–254.
Kaizaki, S. & Takemoto, H. (1990). *Inorg. Chem.* **29**, 4960–4964.

electronic reprint

Kane-Maguire, N. A. P., Wallace, K. C. & Speece, D. G. (1986). *Inorg. Chem.* **25**, 4650–4654.
Nonius (1999). *COLLECT*. Nonius BV, Delft, The Netherlands.
Sheldrick, G. M. (2008). *Acta Cryst.* A**64**, 112–122.
Yamaguchi-Terasaki, Y., Fujihara, T., Nagasawa, A. & Kaizaki, S. (2007). *Acta Cryst.* E**63**, m593–m595.

electronic reprint

Acta Cryst. (2008). E**64**, m369-m370 [**doi:10.1107/S1600536808001153**]

cis-Difluoridobis(1,10-phenanthroline)chromium(III) perchlorate monohydrate

T. Birk, J. Bendix and H. Weihe

Comment

Difluoro complexes of chromium(III) with various amine ligands have received a steady interest in the literature. Areas of interest have been *e.g.* kinetic behavior (Delavar & Staples, 1981), solvatochromism (Kaizaki & Takemoto, 1990) and photochemical/photophysical properties (Kane-Maguire *et al.*, 1986). From a synthetic point of view simple fluoro containing complexes exhibit some advantageous properties for synthesis in non-acidic media. The strong coordination of the small and basic fluoro ligand makes it suitable as an "inorganic" protection group, easily removed and substituted by other ligands. Only a limited number of complexes belonging to this group have been structural characterized *e.g.* *cis*-$[Cr(NH_3)_4F_2]ClO_4$ (Brenčič *et al.*, 1981), *cis*-$[Cr(en)_2F_2]ClO_4 \cdot NaClOO_4 \cdot H_2O$ (Brenčič *et al.*, 1987) and *cis*-$[Cr(bipy)_2F_2]ClO_4$ (Yamaguchi-Terasaki *et al.*, 2007). In this report we present the crystal structure of *cis*-Difluoro(1,10-phenanthroline)chromium(III) perchlorate monohydrate (1).

The structure of (1) shows a octahedral coordination geometry around the central chromium(III) ion consisting of a *cis* arrangment of two fluorine and two nitrogen ligator atoms (Figure 1). Comparison of the Cr—N bond distances in *trans* position relative to the fluoro ligand [N_1: 2.0934 (15) Å and N_3: 2.0797 (16) Å] show a slightly elongation compared to the corresponding in *cis* postion [N_2: 2.0607 (15) Å and N_4: 2.0566 (15) Å]. This pattern of bond lengths are similar to that found in the analogous bipyridine complex *cis*-Difluoro(2,2'-bipyridine)chromium(III) perchlorate, *cis*-$[Cr(bipy)_2F_2]ClO_4$.

The overall crystal packing is predominately determined by the approximately perpendicular orientation of the two planar 1,10-phenanthroline ligands [N_3—Cr—N_1:87.34 (6) °, N_3—Cr—N_2: 96.36 (6) °] and the presence of crystal water connecting each asymmetric unit with another through hydrogen bonding from water to fluorine (Figure 2).

Experimental

The title complex was synthesized by reflux of *trans*-difluorotetrakis(pyridine)chromium(III) perchlorate and 1,10-phenanthroline in 2-methoxyethanol according to the published method (Glerup *et al.*, 1970).

Crystal suitable for X-ray diffraction were obtained by the following method: 0.208 g of the compound was dissolved in a solution of water/acetonitrile (20 ml/10 ml) and filtered though a filter paper into a small beaker. The beaker was covered with a lid of paper and left undisturbed at room temperature for crystallization (*ca* 3–5 days). The crystals was harvested by gently scratching with a spatula and washed with the mother liquid.

Refinement

All H atoms were identified in a difference Fourier map and incorporated in the refinement in a riding model, with C–H = 0.95 Å and $U_{iso}(H) = 1.2U_{Eq}$.

electronic reprint

Figures

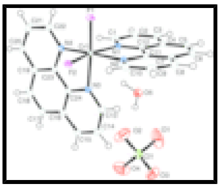

Fig. 1. The molecular structure and atom labeling scheme of *cis*-$[Cr(phen)_2F_2]ClO_4^{\cdot}$ H_2O. Displacement ellipsoids are drawn at 50% probability. H atoms with arbitrary radii.

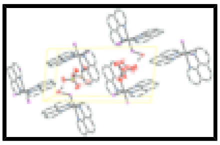

Fig. 2. The crystal packing in *cis*-$[Cr(phen)_2F_2]ClO_4^{\cdot}$ H_2O. Displacement ellipsoids are drawn at 50% probability. H atoms except the one originated from cystal water have been omitted.

cis-Difluorido(1,10-phenanthroline)chromium(III) perchlorate monohydrate

Crystal data

$[CrF_2(C_{12}H_8N_2)_2]ClO_4{\cdot}H_2O$	$Z = 2$
$M_r = 567.87$	$F_{000} = 578$
Triclinic, $P\bar{1}$	$D_x = 1.651$ Mg m^{-3}
Hall symbol: -P 1	Mo $K\alpha$ radiation<br>$\lambda = 0.71073$ Å
$a = 7.6930$ (10) Å	Cell parameters from 26598 reflections
$b = 9.4640$ (8) Å	$\theta = 2.3$–$25.0°$
$c = 16.0610$ (17) Å	$\mu = 0.68$ mm^{-1}
$\alpha = 79.750$ (7)°	$T = 122$ (1) K
$\beta = 83.228$ (12)°	Block, red
$\gamma = 88.115$ (8)°	0.44 × 0.41 × 0.16 mm
$V = 1142.6$ (2) Å^3	

Data collection

Nonius KappaCCD area-detector diffractometer	4014 independent reflections
Radiation source: fine-focus sealed tube	3851 reflections with $I > 2\sigma(I)$
Monochromator: graphite	$R_{int} = 0.025$
$T = 122.0(10)$ K	$\theta_{max} = 25.0°$
ω and φ scans	$\theta_{min} = 2.3°$
Absorption correction: gaussian integration (Coppens, 1970)	$h = -9 \rightarrow 9$
$T_{min} = 0.794$, $T_{max} = 0.913$	$k = -11 \rightarrow 11$
28606 measured reflections	$l = -18 \rightarrow 19$

electronic reprint

Refinement

Refinement on F^2

Least-squares matrix: full

$R[F^2 > 2\sigma(F^2)] = 0.031$

$wR(F^2) = 0.145$

$S = 1.41$

4014 reflections

329 parameters

H-atom parameters constrained

$w = 1/[\sigma^2(F_o^2) + (0.1P)^2]$
where $P = (F_o^2 + 2F_c^2)/3$

$(\Delta/\sigma)_{max} = 0.078$

$\Delta\rho_{max} = 0.79$ e Å^{-3}

$\Delta\rho_{min} = -0.51$ e Å^{-3}

Extinction correction: none

Special details

Geometry. All e.s.d.'s (except the e.s.d. in the dihedral angle between two l.s. planes) are estimated using the full covariance matrix. The cell e.s.d.'s are taken into account individually in the estimation of e.s.d.'s in distances, angles and torsion angles; correlations between e.s.d.'s in cell parameters are only used when they are defined by crystal symmetry. An approximate (isotropic) treatment of cell e.s.d.'s is used for estimating e.s.d.'s involving l.s. planes.

Refinement. Refinement of F^2 against ALL reflections. The weighted *R*-factor *wR* and goodness of fit S are based on F^2, conventional *R*-factors *R* are based on F, with F set to zero for negative F^2. The threshold expression of $F^2 > 2\text{sigma}(F^2)$ is used only for calculating *R*-factors(gt) *etc*. and is not relevant to the choice of reflections for refinement. *R*-factors based on F^2 are statistically about twice as large as those based on F, and R– factors based on ALL data will be even larger.

Fractional atomic coordinates and isotropic or equivalent isotropic displacement parameters (Å^2)

	x	*y*	*z*	U_{iso}*/U_{eq}
Cr1	0.86046 (3)	0.55137 (3)	0.266315 (15)	0.01130 (17)
Cl1	0.42169 (6)	0.04581 (5)	0.27329 (3)	0.02027 (19)
F1	1.08731 (13)	0.60734 (11)	0.22192 (6)	0.0170 (3)
F2	0.92467 (13)	0.41293 (11)	0.35383 (6)	0.0175 (3)
N1	0.76931 (19)	0.68048 (16)	0.16003 (9)	0.0124 (3)
N3	0.6003 (2)	0.52586 (16)	0.31812 (9)	0.0136 (3)
N4	0.83917 (19)	0.70717 (16)	0.34149 (9)	0.0146 (3)
N2	0.8597 (2)	0.40348 (16)	0.18644 (10)	0.0144 (3)
C12	0.7592 (2)	0.60779 (19)	0.09479 (11)	0.0137 (4)
C1	0.7256 (2)	0.81930 (19)	0.14890 (12)	0.0171 (4)
H1	0.7315	0.8707	0.1942	0.020*
C24	0.5533 (2)	0.61063 (18)	0.37709 (10)	0.0138 (4)
C23	0.6826 (2)	0.70727 (19)	0.39112 (10)	0.0139 (4)
C13	0.4814 (2)	0.43556 (19)	0.30366 (12)	0.0173 (4)
H13	0.5134	0.3751	0.2629	0.021*
C15	0.2655 (2)	0.5115 (2)	0.40692 (12)	0.0197 (4)
H15	0.1510	0.5056	0.4367	0.024*
C11	0.8100 (2)	0.45883 (19)	0.10871 (11)	0.0140 (4)
C10	0.9151 (2)	0.26736 (19)	0.20079 (12)	0.0191 (4)
H10	0.9493	0.2277	0.2551	0.023*
C18	0.4782 (3)	0.7862 (2)	0.50181 (12)	0.0202 (4)

electronic reprint

PAPER 3

cis-Aquabis(2,2′-bipyridine-κ^2-_N,N’_) fluoridochromium(III) bis(perchlorate) dihydrate

Torben Birk and Jesper Bendix

Acta Cryst., 2010, E66, m121-m122

Acta Crystallographica Section E
Structure Reports
Online
ISSN 1600-5368

cis-Aquabis(2,2′-bipyridine-κ^2N,N')-fluoridochromium(III) bis(perchlorate) dihydrate

Torben Birk* and Jesper Bendix

Department of Chemistry, University of Copenhagen, Universitetsparken 5, DK.2100 Copenhagen Ø, Denmark
Correspondence e-mail: birk@kiku.dk

Received 21 December 2009; accepted 3 January 2010

Key indicators: single-crystal X-ray study; T = 122 K; mean σ(C–C) = 0.002 Å; R factor = 0.026; wR factor = 0.070; data-to-parameter ratio = 16.2.

The title mixed aqua–fluoride complex, $[CrF(C_{10}H_8N_2)_2(H_2O)](ClO_4)_2\cdot 2H_2O$, has been synthesized by aquation of the corresponding difluoride complex using lanthanide(III) ions as F^- acceptors. The complex crystallizes with a Cr^{III} ion at the center of a distorted octahedral coordination polyhedron with a *cis* arrangement of ligands. The crystal packing shows a hydrogen-bonding pattern involving water molecules, the coordinated F atom and the perchlorate anions

Related literature

For related difluoride complexes, see: Birk *et al.* (2008); Brenčič *et al.* (1987); Brenčič & Leban (1981); DeJovine *et al.* (1974); Delavar & Staples (1981); Kavitha *et al.* (2005); Vaughn *et al.* (1968); Vaughn & Seiler (1979); Yamaguchi-Terasaki *et al.* (2007). For related structures, see: Casellato *et al.* (1986); Liu (2009). For details of the synthesis, see: Glerup *et al.* (1970).

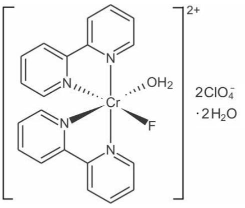

Experimental

Crystal data

$[CrF(C_{10}H_8N_2)_2(H_2O)](ClO_4)_2\cdot 2H_2O$
M_r = 636.32
Triclinic, $P\bar{1}$
a = 9.577 (1) Å
b = 11.4050 (6) Å
c = 11.8150 (11) Å
α = 77.273 (6)°
β = 79.427 (9)°
γ = 83.590 (5)°
V = 1234.01 (19) Å^3
Z = 2
Mo $K\alpha$ radiation
μ = 0.76 mm^{-1}
T = 122 K
0.41 × 0.24 × 0.14 mm

Data collection

Nonius KappaCCD diffractometer
Absorption correction: Gaussian (Coppens, 1970)
T_{min} = 0.805, T_{max} = 0.925
27824 measured reflections
5691 independent reflections
5244 reflections with $I > 2\sigma(I)$
R_{int} = 0.021

Refinement

$R[F^2 > 2\sigma(F^2)]$ = 0.026
$wR(F^2)$ = 0.070
S = 1.03
5691 reflections
352 parameters
H-atom parameters constrained
$\Delta\rho_{max}$ = 0.55 e Å^{-3}
$\Delta\rho_{min}$ = −0.42 e Å^{-3}

Table 1
Selected bond lengths (Å).

Cr1—F1	1.8614 (8)	Cr1—N2	2.0456 (12)
Cr1—O1	1.9579 (10)	Cr1—N3	2.0545 (12)
Cr1—N1	2.0501 (12)	Cr1—N4	2.0571 (12)

Table 2
Hydrogen-bond geometry (Å, °).

D—H···A	D—H	H···A	D···A	D—H···A
O1—H1A···F1^{i}	0.83	1.73	2.5482 (13)	174
O1—H1B···O2^{ii}	0.83	1.73	2.5548 (15)	176
O2—H2A···O3	0.90	1.89	2.7887 (18)	179
O2—H2B···O5	0.84	2.14	2.9380 (17)	158
O3—H3A···O10^{iii}	0.91	2.00	2.890 (2)	167
O3—H3B···O8	0.87	2.19	3.050 (2)	168
O3—H3B···O9	0.87	2.48	3.123 (2)	132

Symmetry codes: (i) $-x+1, -y, -z+2$; (ii) $-x+1, -y+1, -z+1$; (iii) $-x, -y+1, -z+1$.

Table 3
M—F bond distances (Å) for related *cis/trans*-$[M(L)_2F_2]^+$ complexes.

(I)	(II)	(III)	(IV)	(V)
1.8621 (10)	1.8541 (10)	1.887 (6)	1.887 (5)	1.7389 (15)
1.8444 (10)	1.8409 (10)	1.878 (6)	1.868 (4)	1.7232 (15)

Notes: (I) *cis*-$[Cr(phen)_2F_2]ClO_4\cdot H_2O$ (Birk *et al.*, 2008); (II) *cis*-$[Cr(bipy)_2F_2]ClO_4\cdot H_2O$ (Yamaguchi-Terasaki *et al.*, 2007); (III) *trans*-$[Cr(en)_2F_2]ClO_4$ (Brenčič & Leban, 1981); (IV) *cis*-$[Cr(en)_2F_2]ClO_4\cdot NaClO_4\cdot H_2O$ (Brenčič *et al.*, 1987); (V) *cis*-$[V(bipy)_2F_2]BF_4$ (Kavitha *et al.*, 2005). en = ethane-1,2-diamine; bipy = 2,2′-bipyridine; phen = 1,10-phenanthroline.

Data collection: *COLLECT* (Nonius, 1998); cell refinement: *COLLECT*; data reduction: *EVALCCD* (Duisenberg *et al.*, 2003); program(s) used to solve structure: *SHELXS97* (Sheldrick, 2008); program(s) used to refine structure: *SHELXL97* (Sheldrick, 2008); molecular graphics: *ORTEP-3* (Farrugia, 1997); software used to prepare material for publication: *SHELXL97*.

The authors are gratful to Mr Flemming Hansen (Centre of Crystallographic Studies, University of Copenhagen) for collecting the X-ray diffraction data.

Supplementary data and figures for this paper are available from the IUCr electronic archives (Reference: HY2268).

electronic reprint

References

Birk, T., Bendix, J. & Weihe, H. (2008). *Acta Cryst.* E**64**, m369–m370.
Brenčič, J. V. & Leban, I. (1981). *Z. Anorg. Allg. Chem.* **480**, 213–219.
Brenčič, J. V., Leban, I. & Polanc, I. (1987). *Z. Anorg. Allg. Chem.* **551**, 109–115.
Casellato, U., Graziani, R., Maccarrone, G. & Bilio, G. M. (1986). *J. Crystallogr. Spectrosc. Res.* **16**, 695–702.
Coppens, P. (1970). *Crystallographic Computing*, edited by F. R. Ahmed, S. R. Hall & C. P. Huber, pp. 255–270. Copenhagen: Munksgaard.
DeJovine, J., Mason, W. R. & Vaughn, J. W. (1974). *Inorg. Chem.* **13**, 66–73.
Delavar, M. & Staples, P. J. (1981). *J. Chem. Soc. Dalton Trans.* pp. 981–985.
Duisenberg, A. J. M., Kroon-Batenburg, L. M. J. & Schreurs, A. M. M. (2003). *J. Appl. Cryst.* **36**, 220–229.
Farrugia, L. J. (1997). *J. Appl. Cryst.* **30**, 565.
Glerup, J., Josephsen, J., Michelsen, K., Pedersen, E. & Schäffer, C. E. (1970). *Acta Chem. Scand.* **24**, 247–254.
Kavitha, S. J., Panchanatheswaran, K., Low, J. N. & Glidewell, C. (2005). *Acta Cryst.* E**61**, m1965–m1967.
Liu, H.-X. (2009). *Acta Cryst.* E**65**, m1093.
Nonius (1998). *COLLECT*. Nonius BV, Delft, The Netherlands.
Sheldrick, G. M. (2008). *Acta Cryst.* A**64**, 112–122.
Vaughn, J. W. & Seiler, G. J. (1979). *Inorg. Chem.* **18**, 1509–1511.
Vaughn, J. W., Stvan, O. J. & Magnuson, V. E. (1968). *Inorg. Chem.* **7**, 736–741.
Yamaguchi-Terasaki, Y., Fujihara, T., Nagasawa, A. & Kaizaki, S. (2007). *Acta Cryst.* E**63**, m593–m595.

electronic reprint

Acta Cryst. (2010). E**66**, m121-m122 [**doi:10.1107/S1600536810000127**]

cis-Aquabis(2,2'-bipyridine-κ^2N,N')fluoridochromium(III) bis(perchlorate) dihydrate

T. Birk and J. Bendix

Comment

Water and fluoride are both hard donor ligands favoring the same central ions. Nevertheless, no transition metal complexes of the general type *cis*-[M(*L*)$_2$(H$_2$O)F]$^{2+}$ with *L* being a bidentate ligand such as 2,2'-bipyridine (bipy), 1,10-phenanthroline (phen) or ethane-1,2-diamine (en) have previously been characterized by X-ray diffraction. This report presents the synthesis and crystal structure of such a system exemplified by the title complex *cis*-[Cr(bipy)$_2$(H$_2$O)F](ClO$_4$)$_2$.2H$_2$O.

All trivalent lanthanid ions, Ln^{3+} are known to be hard Lewis acids. This Lewis acidity gives rise to favorable bond formation with ligands containing oxygen and fluorine ligator atoms. The interactions between lanthanid ions and coordinated fluoride have not received much attention compared to the plethora of oxygen bridged systems reported. We have initiated a study on the reactivity of lanthanid ions towards coordinated fluoride ligands to assess if new fluoride contaning complexes can be synthesized this way. In the context of these studies, we found that the title complex, as well as the corresponding phen complex, can be synthesized by a lanthanid ion assisted aquation of the difluoride complex *cis*-[Cr(bipy)$_2$F$_2$]ClO$_4$. The normal aquation reaction is performed in strong acid and has been studied preparatively and kinetic for difluoride complexes as *trans*-[Cr(en)$_2$F$_2$]$^+$ and *cis*-[Cr(bipy)$_2$F$_2$]$^+$ (DeJovine *et al.*, 1974; Delavar & Staples, 1981; Vaughn *et al.*, 1968; Vaughn & Seiler, 1979). However, the present synthetic approach is more general, as it can be applied also to systems with an acid-labile auxillary ligand sphere.

The most important structural element in the title compound is the *cis* arrangement of ligators in a distorted octahedral coordination polyhedron around the central Cr^{III} ion (Fig. 1). Distortion from ideal geometry is dictated by the nearly fixed bite angles of the two bipy ligands [79.51 (5) and 79.24 (5)°]. This pattern is also seen in the structurally related difluoride complexes *cis*-[V(bipy)$_2$F$_2$]BF$_4$ (Kavitha *et al.*, 2005) and *cis*-[Cr(bipy)F$_2$]ClO$_4$.H$_2$O (Yamaguchi-Terasaki *et al.*, 2007). The Cr1—F1 bond distance of 1.8614 (8) Å (Table 1) is in accordance with the structurally characterized difluoride complexes, as shown in Table 3. The Cr1—O1 bond distance of 1.9579 (10)Å is shorter than what is seen in both the tricationic, diaqua complex *cis*-[Cr(bipy)$_2$(H$_2$O)$_2$](NO$_3$)$_3$ [2.00 (1) and 1.98 (1) Å] (Casellato *et al.*, 1986), as well as in the uncharged [Cr(bipy)(H$_2$O)F$_3$].2H$_2$O [1.979 (2) Å] (Liu, 2009). In the structure of *cis*-[Cr(bipy)$_2$F$_2$]$^+$, a *trans* influence leading to the Cr—N bond distances *trans* to the fluorido ligand being longer that the corresponding *cis* distances was identified by Yamaguchi-Terasaki *et al.* (2007). This situation was also found in *cis*-[Cr(phen)$_2$F$_2$]$^+$ (Birk *et al.*, 2008), but is not discernible in the structure reported here.

The crystal packing in the title complex (Fig. 2) shows a hydrogen bonding pattern involving water molecules, coordinated F atom and perchlorate anions (Table 2).

electronic reprint

Experimental

Safety remark: Perchlorate complexes of metal ions are potentially explosive. The title complex burns with high intensity when ignited in a gas flame. According to Delavar & Staples (1981), the corresponding phen complex is explosive.

All chemicals were used as received. *cis*-[Cr(bipy)$_2$F$_2$]ClO$_4$ was synthesized by reflux of *trans*-difluorotetrakis(pyridine)chromium(III) perchlorate and bipy in 2-methoxyethanol according to the published method (Glerup *et al.*, 1970).

Nd_2O_3 (0.251 g, 0.746 mmol) was dissolved in 0.5 ml $HClO_4$ (60%) by gentle heating giving a pink solution to which was added a mixture of *cis*-[Cr(bipy)$_2$F$_2$]ClO$_4$ (1.011 g, 2.015 mmol) in acteonitrile (60 ml) and water (20 ml). Mixing gave rise to a slight color change from purple to red. The solution was placed in a water bath (~70°C) and was stirred for 30–35 min. The resulting muddy red suspension was solidified by cooling to room temperature. Extraction with a mixture of acetonitrile and water (2:1, 30 ml) gave an orange turbid solution which was separated into a clear solution and white precipitate by centrifugation. Slow evaporation gave an orange crystalline product which contained crystals of suitable quality for X-ray diffraction. The product was isolated by filtering, washed with ice water and dried in air (yield 1.073 g, 83.7%). Analysis, calculated for $C_{20}H_{22}Cl_2CrFN_4O_{11}$: C 37.75, H 3.48, N 8.80%; found: C 37.97, H 3.41, N 8.70%.

The corresponding phen complex, *cis*-[Cr(phen)$_2$(H$_2$O)F](ClO$_4$)$_2$.H$_2$O can be synthesized from *cis*-[Cr(phen)$_2$F$_2$]ClO$_4$ (1.034 g, 1.88 mmol) by a similar procedure (yield 0.781 g, 60.7%). Analysis, calculated for $C_{24}H_{22}Cl_2CrFN_4O_{11}$: C 42.12, H 3.24, N 8.19%; found: C 42.07, H 2.73, N 8.02%.

Refinement

The aromatic H atoms were placed in geometrically idealized positions and refined as riding atoms, with C—H = 0.95 Å and with U_{iso}(H) = 1.2U_{eq}(C). H atoms of water molecules were identified in a difference Fourier map and refined as riding with U_{iso}(H) = 1.2U_{eq}(O).

Figures

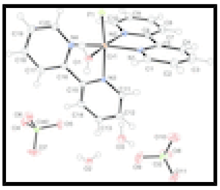

Fig. 1. The molecular structure of the title compound. Displacement ellipsoids are drawn at the 50% probability.

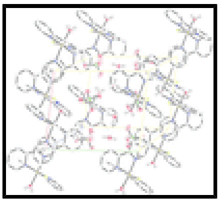

Fig. 2. The crystal packing in the title compound. Displacement ellipsoids are drawn at the 50% probability. H atoms except those of water molecules have been omitted for clarity.

electronic reprint

cis-Aquabis(2,2'-bipyridine-κ^2*N*,*N*')fluoridochromium(III) bis(perchlorate) dihydrate

Crystal data

$[CrF(C_{10}H_8N_2)_2(H_2O)](ClO_4)_2\cdot 2H_2O$	$Z = 2$
$M_r = 636.32$	$F(000) = 650$
Triclinic, $P\bar{1}$	$D_x = 1.712$ Mg m^{-3}
Hall symbol: -P 1	Mo $K\alpha$ radiation, $\lambda = 0.71073$ Å
$a = 9.577$ (1) Å	Cell parameters from 19362 reflections
$b = 11.4050$ (6) Å	$\theta = 1.8$–$27.6°$
$c = 11.8150$ (11) Å	$\mu = 0.76$ mm^{-1}
$\alpha = 77.273$ (6)°	$T = 122$ K
$\beta = 79.427$ (9)°	Block, orange
$\gamma = 83.590$ (5)°	0.41 × 0.24 × 0.14 mm
$V = 1234.01$ (19) Å^3	

Data collection

Nonius KappaCCD diffractometer	5691 independent reflections
Radiation source: fine-focus sealed tube	5244 reflections with $I > 2\sigma(I)$
graphite	$R_{int} = 0.021$
ω and φ scans	$\theta_{max} = 27.6°$, $\theta_{min} = 1.8°$
Absorption correction: gaussian (Coppens, 1970)	$h = -12\rightarrow 12$
$T_{min} = 0.805$, $T_{max} = 0.925$	$k = -14\rightarrow 11$
27824 measured reflections	$l = -15\rightarrow 15$

Refinement

Refinement on F^2	Primary atom site location: structure-invariant direct methods
Least-squares matrix: full	Secondary atom site location: difference Fourier map
$R[F^2 > 2\sigma(F^2)] = 0.026$	Hydrogen site location: inferred from neighbouring sites
$wR(F^2) = 0.070$	H-atom parameters constrained
$S = 1.03$	$w = 1/[\sigma^2(F_o^2) + (0.0294P)^2 + 1.0724P]$ where $P = (F_o^2 + 2F_c^2)/3$
5691 reflections	$(\Delta/\sigma)_{max} = 0.001$
352 parameters	$\Delta\rho_{max} = 0.55$ e Å^{-3}
0 restraints	$\Delta\rho_{min} = -0.42$ e Å^{-3}

Fractional atomic coordinates and isotropic or equivalent isotropic displacement parameters (Å^2)

	x	*y*	*z*	U_{iso}*/U_{eq}
Cr1	0.35121 (2)	0.123062 (19)	0.848099 (18)	0.00991 (6)

electronic reprint

PAPER 4

Alkali metal cation complexation and solvent interactions by robust chromium(III) fluoride complexes

Torben Birk, Magnus J. Magnussen, Stergios Piligkos, Högni Weihe, Anders Holten and Jesper Bendix

J. Fluorine Chem., 2010, 131(9), 898-906.

Journal of Fluorine Chemistry 131 (2010) 898–906

Contents lists available at ScienceDirect

Journal of Fluorine Chemistry

journal homepage: www.elsevier.com/locate/fluor

Alkali metal cation complexation and solvent interactions by robust chromium(III) fluoride complexes

Torben Birk, Magnus J. Magnussen, Stergios Piligkos, Högni Weihe, Anders Holten, Jesper Bendix *

Department of Chemistry, University of Copenhagen, Universitetsparken 5, DK-2100 Copenhagen, Denmark

ARTICLE INFO

Article history:
Received 27 February 2010
Received in revised form 7 June 2010
Accepted 8 June 2010
Available online 15 June 2010

Keywords:
Bridging
Cr(III)
Robust
Linear
Chain

ABSTRACT

Interaction of robust chromium(III) fluoride complexes with sodium or lithium cations in solution lead to hypsochromic spectral shifts of increasing magnitude along the series: *trans*-$[CrF_2(py)_4]^+$, *mer*-$[CrF_3(terpy)]$, and *fac*-$[CrF_3(Me_3tacn)]$. Crystalline products isolated from solution exhibit μ_2-bridging by the fluoride ligands in a linear fashion between Na^+-ions and chromium centres in *catena*-$[Na(H_2O)_4(\mu\text{-F})$-*trans*-$\{CrF(py)_4\}](HCO_3)_2$ and in the dimers $[Li(H_2O)_n(\mu\text{-F})$-*trans*-$\{CrF(py)_4\}]^{2+}$ ($n = 3, 4$). The uncharged chromium complexes *fac*-$[CrF_3(Me_3tacn)]$ and *mer*-$[CrF_3(terpy)]$ have been synthesized from *mer*-$[CrF_3(py)_3]$ and shown to precipitate sodium salts from solution, of which $3[CrF_3(Me_3tacn)]\cdot 2Na(Bph_4)\cdot$solv and $6[CrF_3(terpy)]\cdot 4Na(Bph_4)\cdot$solv have been crystallographically characterized. In these clusters, the neutral fluoride complexes bring the Na^+ cation separation down to 3.610 Å and 3.369 Å, respectively, which is much closer than the inter-cation distance in NaCl and comparable to that of NaF. DFT calculations support the notion of a strong interaction between Na^+ ions and neutral chromium(III) fluoride complexes. The calculations reproduce the magnitude and the counter-intuitive sign of the spectral shifts induced by second sphere complexation in solution, which originates in a breakdown of the assumption of parameter transferability in ligand-field descriptions.

© 2010 Elsevier B.V. All rights reserved.

1. Introduction

The high propensity of fluoride for hydrogen bonding [1–4] and interactions with hard (or type-A) cations [5–9] makes fluoride complexes candidates for investigation of second sphere coordination as well as possible structure-directing building blocks in the synthesis of framework structures. Interaction of fluoride complexes with protic solvents leading to solvatochromism is well established and has been investigated especially by Kaizaki and co-workers [10–13]. However, spectral consequences of more general second sphere interactions have not been reported for fluoride complexes and the possibility of spectroscopic cation sensing by fluoride complexes has not been investigated. Also in the solid state, fluoride is known to bridge between metal ions in molecular entities in a multitude of ways: linear μ_2 [14], bent μ_2 [15], symmetrical μ_3 [16], unsymmetrical μ_3 [17], μ_4 [18], and μ_6 [19], but with a quite distinct preference for (almost) linear μ_2-bridging.

Here, we show that robust chromium(III)fluoride complexes interact not only with protic solvents, but also with alkali metal ions in solution. The second sphere coordination results in clustering or chain formation in the solid state where novel types of fluoride-bridged heterometallic dimers (Li^+) or chain polymers (Na^+) can be isolated starting from the *trans*-$[CrF_2(py)_4]^+$ cation. The ability of fluoride to tightly assemble cations is further demonstrated by co-crystallization of the uncharged chromium complexes *fac*-$[CrF_3(Me_3tacn)]$ and *mer*-$[CrF_3(terpy)]$ with sodium tetraphenylborate leading to sodium cation clustering with notably short separations between the sodium cations. The apparent, counter-intuitive increase in donor strength of fluoride upon second sphere complexation is studied by DFT calculations, which show ligand synergies and thus illuminate the problems associated with ligand-field descriptions of the phenomenon.

2. Results and discussion

2.1. Syntheses

Direct reaction between *trans*-$[CrF_2(py)_4]^+$ and lithium or sodium salts in water yields crystalline products containing partially solvated alkali metal ions which also coordinate fluoride(s) from the chromium complex. It is noteworthy, that even a cationic fluoride complex competes efficiently with water as ligand for the Li^+ and Na^+ ions. The specific salts in consideration here were selected as some of those yielding crystalline products among a range of attempted reactions all executed under similar

* Corresponding author. Tel.: +45 35320101.
E-mail address: bendix@kiku.dk (J. Bendix).

0022-1139/$ – see front matter © 2010 Elsevier B.V. All rights reserved.
doi:10.1016/j.jfluchem.2010.06.003

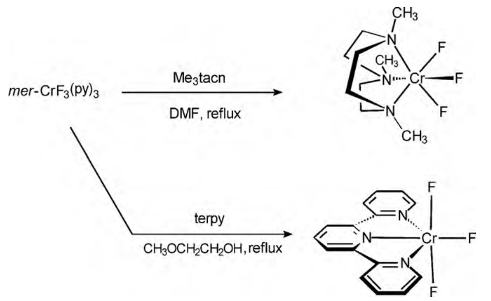

Scheme 1. Synthesis of facial and meridional neutral chromium(III) fluoride complexes.

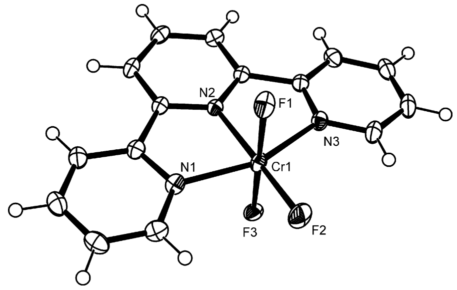

Fig. 1. Molecular structure of $[CrF_3(terpy)]$ in **3**. Bond lengths (Å) around the chromium(III) centre: Cr–F1 1.9033(16), Cr–F2 1.8671(16), Cr–F3 1.8915(16), Cr–N1 2.080(2) Cr–N2 2.009(2), Cr–N3 2.078(2). Bond angles (°): F2–Cr1–F3 92.48(8), F2–Cr1–F1 89.93(8), F3–Cr1–F1, 177.10(7); N2–Cr1–N3, 78.19(8); N2–Cr1–N1, 78.14(8).

conditions. The use of chromium fluoride complexes as ligands for hard cations is of wide scope, but naturally limited by interactions between counter ions from the chromium complex and the targeted hard metal ions. Thus, fluoride complexes with weakly coordinating counter ions or ideal complexes without counter ions altogether are preferable. To realize the latter situation we targeted neutral chromium(III) fluoride complexes, which at the same time are as robust as possible by using tridentate auxiliary ligands. The two possible configurations *fac* and *mer* were imposed on the chromium centre by the use of the geometrically constrained ligands 1,4,7-trimethyl-1,4,7-triazacyclononane (Me_3tacn) and 2,2′;6′,2″-terpyridine (terpy), respectively. The two new complexes were synthesized as outlined in Scheme 1 from the same starting material employing reflux in a solvent with a higher boiling point than the expelled pyridine.

The uncharged complexes were found to precipitate sodium salts such as NaI or $Na(Bph_4)$ efficiently from weak donor solvents. Again the reaction is of wide scope, complicated mainly by the need for carefully chosen conditions to obtain well-crystallized products. In general, the fluoride complexes are poorly soluble in non-protic solvents while in most cases, though not for the tetraphenylborate salts, water competes too well and prevents the isolation of fluoride-bridged compounds. Accordingly, alcohols were found suitable for the isolation of polynuclear M^{I}–F–Cr^{III} systems.

2.2. X-ray crystallography

2.2.1. Coordination geometry and bonding

In $[CrF_3(terpy)]\cdot 2.5H_2O$ (**3**) the chromium centre is coordinated with a distorted octahedral environment with meridional geometry imposed by the terpyridine ligand. The complex is shown in Fig. 1 and relevant metric data are given in the figure caption.

There are no other structurally characterized, monomeric, uncharged six-coordinate chromium(III) complexes with terminal fluoride ligands. Chromium fluoride bond lengths are slightly longer than those found for structurally characterized cationic, cis-$[Cr(phen)_2F_2](ClO_4)\cdot H_2O$ with Cr–F at 1.844 Å and 1.862 Å [20] and (1.857 Å) in *trans*-$[CrF_2(py)_4](PF_6)$ [21], but close to the Cr–F distances in anionic $[CrF_4(py)_2]^-$ (1.881 Å; 1.893 Å) [22]. The chromium nitrogen bond distances are close to those found in $[CrCl_3(terpy)]\cdot$dmso (1.992 Å, 2.071 Å, 2.078 Å) [23].

The double salt *trans*-$[CrF_2(py)_4][Cr(py)_4F(\mu\text{-}F)Li(H_2O)_3][Cr(py)_4F(\mu\text{-}F)Li(H_2O)_4]Cl_5\cdot 6H_2O$ (**1**) contains, in addition to the parent cation *trans*-$[CrF_2(py)_4]^+$, two different dimeric, fluoride-bridged complexes, with 4 and 5 coordinate lithium ions, respectively. The structures are depicted in Fig. 2 with bond lengths given in the figure text.

The fluoride bridges in the Li–F–Cr dimers are almost linear at 164° and 171° and there is very little (0.00–0.02 Å) elongation of the Cr–F bonds upon coordination to the lithium cation. In the

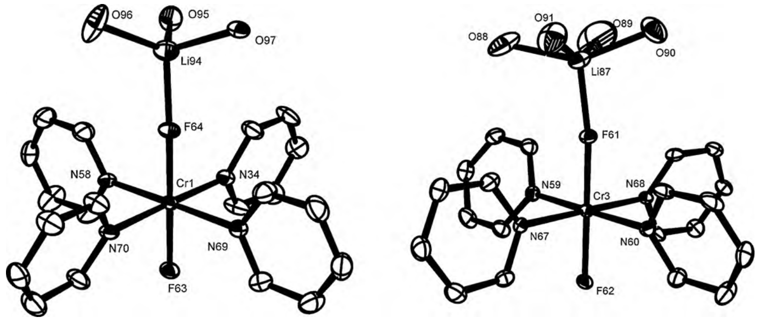

Fig. 2. Ortep drawings of the two heterometallic dimers in **1**, illustrating **4** and **5** coordinate Li^+ cations in *trans*-$[Cr(py)_4F(\mu\text{-}F)Li(H_2O)_3]^{2+}$ and *trans*-$[Cr(py)_4F(\mu\text{-}F)Li(H_2O)_4]^{2+}$, respectively. Hydrogen atoms are omitted for clarity. Selected bond lengths (Å): Cr1–N(av) 2.086(3), Cr1–F63 1.852(2), Cr1–F64 1.8754(19), Li94–F64 1.862(6), Li94–O95 1.934(6), Li94–O96 1.887(6), Li94–O97 1.905(6), Cr3–N(av) 2.083(2), Cr3–F61 1.8699(19), Cr3–F62 1.8623(19), Li87–F61 1.874(6), O89–Li87 2.176(9), O88–Li87 2.016(7), O90–Li87 1.930(7), O91–Li87 2.037(8). Selected bond angles (°): Li94–F64–Cr1 164.12(19), Cr3 F61 Li87 170.7(2).

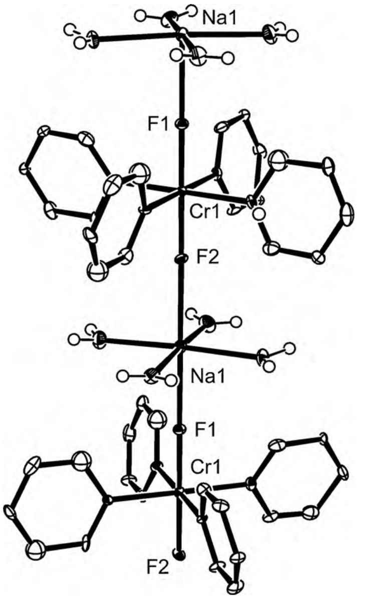

Fig. 3. Part of the chain in **2**, encompassing more than one asymmetric unit. All metal atoms lie on fourfold axes. Hydrogen atoms on the pyridine ligands are omitted for clarity. Selected bond lengths (Å): Cr1–F1 1.8750(8), Cr1–F2 1.8635(7), Cr1–N 2.0834(5), Na1–F1 2.4904(9), Na1–F2 2.4066(9), Na1–O 2.3516(6). Selected bond angles (°): F1–Cr1–F2 180, F2–Cr1–N1 89.779(13), F1–Cr1–N1 90.221(13).

coordination spheres of the Li^+ ions, the average bond lengths to the water ligands depend quite strongly (>0.1 Å) on the coordination number of the Li^+, whereas the Li–F distance (1.862(6) Å and 1.874(6) Å, respectively) is independent of the number of water ligands. Notably, both Li–F bonds in this double salt are shorter than the lithium–water bonds. The fact that the fluoride ion is part of a very weakly structurally perturbed, cationic complex makes this an unexpected observation and demonstrates a quite pronounced donor functionality of the coordinated fluoride towards hard metal ions.

In *catena*-$[Na(H_2O)_4][Cr(py)_4F_2](HCO_3)_2$ (**2**), the tendency towards linear fluoride bridges results in an unprecedented one-dimensional chain polymer containing linear fluoride bridges to the sodium cations. Other examples of linear fluoride bridging among transition metals only include $[pyH]_2[Cu(py)_4(MX_6)_2]$ ($MX_6 = ZrF_6^{2-}$, $NbOF_5^{2-}$, $MoO_2F_4^{2-}$; py = pyridine) [24a] and *catena*-(bis(μ_2-fluoro)-bis(μ_2-oxo)-octafluoro-octakis(pyridine)-di-cadmium-di-niobium) [24b]. In Fig. 3 the structure of the chain without the counter ions is depicted.

As for the structure of **1** described above, the chromium coordination sphere is only slightly perturbed with Cr–F bond length elongations <0.02 Å upon Na^+ complexation. Cr–N bond lengths vary only little and their average is identical to that found in the two lithium cation complexed dimers in **1**. The octahedral *trans* configuration around the sodium cations is elongated along the chain axis with Na–F on average being 0.1 Å longer than the Na–O bonds. The water molecules coordinated to the sodium cations are coordinated in a planar (sp^2-hybrized) fashion with the planes of all four water molecules perpendicular to the chain direction. Normally, this coordination mode is found only when the metal is a good π-acceptor, which obviously is not the case here, where this geometry is imposed by hydrogen bonding to the hydrogen carbonate counter ions (*vide supra*).

Precipitation of $[CrF_3(terpy)]$ (**3**) with $NaBph_4$ in methanol yields the decanuclear cluster shown in Fig. 4.

The cluster contains six uncharged chromium complexes and four sodium cations. Surrounding the cluster, but not shown in the figure are four essentially non-interacting Bph_4^- counter ions. The most prominent feature of the structure is the very short Na^+–Na^+ contacts (3.369 Å and 3.550 Å), which means that the bridging chromium complexes bring the Na^+ ions significantly closer than in NaCl (3.98 Å). As the cluster is situated at a centre of inversion there are only two distinct environments for the Na^+ ions in the structure. The terminal Na^+ ions are five coordinate in a distorted trigonal bipyramid geometry with three fluoride and two methanol (equatorial) ligands. Fluoride bond lengths to the terminal Na^+ ions vary from 2.202 Å to 2.312 Å and sodium–oxygen distances are 2.285 Å and 2.329 Å. The middle Na^+ ions are coordinated by six fluorides in a very irregular geometry with large variation also in bond lengths with Na–F ranging from 2.199 Å to 2.590 Å. The strong interaction between the Na^+ ions and the chromium complexes is also evident from very short Na–Cr distances of 3.248–3.419 Å. Within each cluster the terpyridine ligands are arranged in three distinct layers (Fig. 4, top). With inter-layer distances of 3.5–4.0 Å, π-stacking may stabilize this arrangement, but most likely the rigidity of the chromium complexes in combination with electrostatic repulsion between Na^+ ions determine the structure. As for the structures involving *trans*-$[CrF_2(py)_4]^+$, the Cr–F distances in the terpyridine complexes are at 1.851–1.919 Å only little perturbed by coordination to the Na^+ ions. Actually, Cr–F distances in **4** are on average (1.882 Å) slightly shorter than in the parent chromium complex, **3** (1.887 Å).

With the facial fluoride complex $[CrF_3(Me_3tacn)]$ (**5**), the pentameric cluster in Fig. 5 results.

Also in this cluster, the Na^+ ions are tightly bound with a Na^+–Na^+ distance of 3.61 Å. In **6**, both Na^+ ions are six-coordinated with distorted octahedral geometries. One (Na99) is coordinated by four fluorides, a water molecule and a 2-propanol molecule; the other (Na100) by five fluorides and one water molecule. For both sodium centres, one of the Na–F interactions is very long: 2.857(2) Å for Na99 and 2.989(2) Å for Na100, while the other distances are similar to those found in **4**: 2.194–2.391 Å at Na99 and 2.229–2.335 Å at Na100. The central fluoride (F10) is coordinated in an almost planar T-shaped geometry, bridging Cr3 and Na100 by an angle of 159.7°. As in **4** the tight bonding of the sodium cations also in **5** results in short Cr–Na distances (3.238–3.481 Å).

2.2.2. Crystal packing

Hydrogen bonding assembles the dimers containing four and five coordinate lithium cations into two distinct, parallel chains along the crystallographic *b*-axis. The hydrogen bonds of both chains connect one of the water ligands to Li^+ with the distant terminal fluoride at the chromium centre of the respective dimers (cf. Fig. 6).

All ligating water molecules in **1** are coordinated in a planar fashion with all water ligands on the five coordinate lithium centres oriented parallel to the dimer axis and the chain axis. At the four-coordinate lithium centres two of the water molecules are oriented perpendicular to the dimer axis and only the one engaged in the hydrogen bonds along the chain has its plane parallel to the Li–F–Cr axis. The hydrogen bonds are moderately strong gauged by their lengths (2.681 Å; 2.751 Å) and illustrate again that the donor character of fluoride by no means are quenched by coordination to a trivalent chromium centre, not even when the resulting complex is cationic. This situation differs

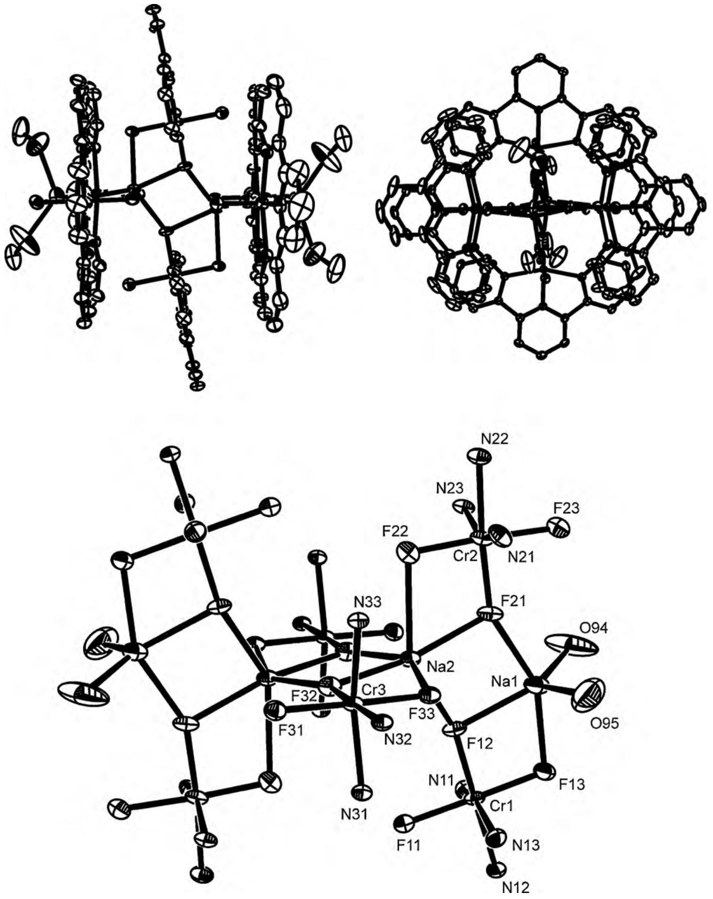

Fig. 4. Three orthogonal views of the cationic cluster $\{6[CrF_3(terpy)]\cdot 4Na\ 4MeOH\}^{4+}$ in **4**. Hydrogen atoms are omitted for clarity in the two top views. In the bottom view all atoms except the metal centres and the directly ligating atom are omitted. The cluster is situated at an inversion centre in the structure. Selected bond lengths (Å): Cr1–F(av) 1.882, Cr2–F(av) 1.884, Cr3–F(av) 1.881, F12–Na2 2.273(3), F1–Na1 2.312(3), F13–Na1 2.258(3), F21–Na1 2.202(3), F21–Na2 2.300(3), F22–Na2 2.589(3), F32–Na2 2.199(3), F33–Na2 2.398(3), Na1–O94 2.285(6), Na1–O95 2.328(6). Selected bond angles (°): O94–Na1–F12 127.3(3), O94–Na1–O95 121.6(3), F12–Na1–O95 110.9(2).

from the tendency of oxygen as a donor which differs markedly between cationic *trans*-$[M(O)F(dppe)_2]^+$ and neutral *trans*-$[M(O)_2(dppe)_2]$ [25].

In **1**, the Li^+ ions show no interaction with the chloride counter ions; the latter participating instead in a hydrogen bond pattern illustrated in Fig. 7.

In **2**, the linear Cr–F–Na–F–Cr… chains are held together by a layer structure in the perpendicular directions. All sodium cations are thus equatorially coordinated by four planar ligating water molecules which each hydrogen bond to two different hydrogencarbonate dimers (cf. Fig. 8). Each hydrogencarbonate dimer is held together by moderately strong hydrogen bonds with lengths at 2.621 Å. The hydrogen bonds between the water molecules ligating the sodium cation and the counter ions are longer at 2.830–2.914 Å, but almost linear (167.14°, 176.06°) and thus also to be classified as moderately strong.

2.3. Spectroscopic characterization

Solvatochromism of chromium(III) fluoride complexes is well established and attributed to solvent interactions with coordinated fluoride. In order to gauge whether the second sphere interaction demonstrated in the solid state structures persist in solution, the influence of Li^+ and Na^+ ions, as well as that of the solvent was examined for the absorption spectra of *trans*-$[CrF_2(py)_4]^+$, *mer*-$[CrF_3(terpy)]$ (**3**), and *fac*-$[CrF_3(Me_3tacn)]$ (**5**). The spectrum of *trans*-$[CrF_2(py)_4]^+$ is only very weakly solvent dependent and even high concentrations (1 M) of Li^+ produce only marginal shifts (ca. 2 nm; $<100\ cm^{-1}$) of the first spin-allowed transition, which for d^3-systems directly measure the octahedral component (Δ_O) of the ligand-field. The spectra of the trifluoro complexes are significantly more sensitive towards the solvent as well as towards Li^+ and Na^+ ions in solution. Data are shown in Fig. 9.

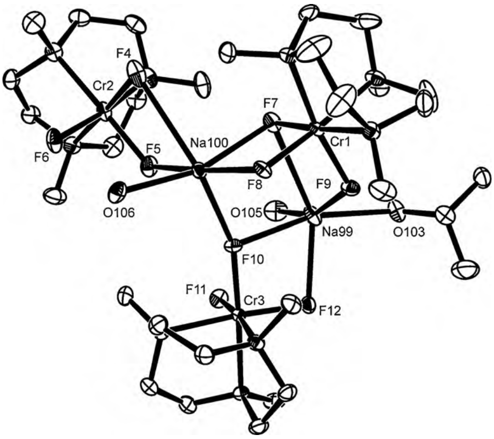

Fig. 5. Structure of the metal ion cluster in **6**. All hydrogen atoms including those of the water molecule are omitted for clarity. Selected bond lengths (Å): Cr1–F(av) 1.8779, Cr2–F(av) 1.8750, Cr3–F(av) 1.8904, Na99–F7 2.857(2), Na99–F9 2.194(2), Na99–F10 2.391(2), Na99–F12 2.341(2), Na99–O105 2.359(3), Na99–O103 2.390(2), Na100–F4 2.989(2), Na100–F5 2.229(2), Na100–F7 2.329(2), Na100–F8 2.3348(19), Na100–F10 2.2680(19), Na100–O106 2.373(2). Selected bond angles (°): Cr3–F10–Na100 159.70(9), Cr3–F10–Na99 98.75(8), Na100–F10–Na99 101.55(7), Cr1–F9–Na99 114.41(9), Cr1–F7–Na100 99.82(8), Cr1–F7–Na99 89.81(7), Na100–F7–Na99 87.62(6), Cr1–F8–Na100 99.78(8), Cr3–F12–Na99 100.17(8), Cr2–F5–Na100 115.91(9), Cr2–F4–Na100 88.19(8).

It is seen that for *mer*-[CrF_3(terpy)] (**3**), going from 2-propanol to water produces a hypsochromic shift of the $^4A_2(O) \rightarrow {}^4T_2(O)$ transition of 25 nm (ca. 800 cm^{-1}), while Li^+ ions (0.25 M) shift the absorption by only 8 nm. Most sensitive is the facial complex, [CrF_3(Me_3tacn)] (**5**), where the spectral shift of the first spin-allowed absorption band between water and acetonitrile is 34 nm (1050 cm^{-1}), turning the blue acetonitrile solution pink–red upon addition of water. Staying in one solvent, 2-propanol, addition of Na^+ ions to a concentration of 0.5 M, produces a shift half as large (600 cm^{-1}) as that observed between water and acetonitrile. The second spin-allowed transition (at ca. 400 nm) and the spin-forbidden $^4A_2(O) \rightarrow {}^2E(O)$ transition (at ca. 700 nm), which is observed for the trifluoride complexes are both less sensitive to solvent/cation perturbation. In all cases increasing hydrogen bond propensity and second sphere coordination by metal ions produce shifts of the first band towards shorter wavelengths. This is unexpected if only the metal fluoride interaction is considered, since both effects would be expected to decrease the donor strength of the fluoride. It has been argued that the spectro-

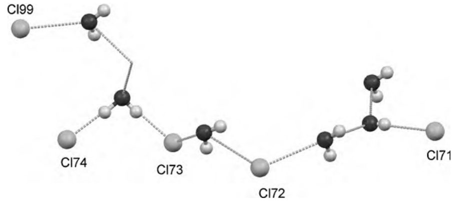

Fig. 7. Chloride-water packing in **1**.

chemical parameter Δ_O is encompassing both σ- and π-antibonding effects through the relation $\Delta_O = 3e_\sigma - 4e_\pi$ and that the increase in Δ_O can reflect a simultaneous decrease in σ- and π-perturbation with the latter decreasing most. An independent test of this interpretation can be provided by DFT which allows for independent study of the effects of the solvent and the second sphere complexation.

2.4. DFT calculations

Mer-[$CrF_3(NH_3)_3$] and *fac*-[$CrF_3(NH_3)_3$] were chosen as computational models for *mer*-[CrF_3(terpy)] and *fac*-[CrF_3(Me_3tacn)] which are the systems exhibiting the largest spectral sensitivity towards solvent and alkali metal cation perturbations. Geometries of the two model systems were optimized computationally without any symmetry constraints in vaccum as well as in water modeled as a continuum (COSMO) [26]. In addition the geometry of the putative adduct {Na•*fac*-[$CrF_3(NH_3)_3$]}$^+$ was optimized in vacuum as well as solvated. The optimized structure of the adduct has the chromium complex functioning as a symmetrically coordinating η^3-ligand towards the Na^+ ion (Fig. 10).

While this structure is intuitively satisfying based on electrostatics it should be noted that this coordination mode with three μ_2-fluoride bridges has never been observed in heterobimetallic systems, the only known examples are a few $[M_2F_9]^{n-}$ ions. [27] Data for the optimized structures are collected in Table 1.

The thermodynamics of the adduct formation between *fac*-[$CrF_3(NH_3)_3$] and Na^+ was found to be strongly dependent on solvation. Calculations yielded a bonding energy of 269 kJ/mol in vacuum which was reduced to 80 kJ/mol when solvation (water, COSMO) was included.

The optimized structures show some general variations: solvation makes the structure more regularly octahedral for the facial as well as the meridional isomer; solvation and complexation with Na^+ has similar effects on the structure of *fac*-[$CrF_3(NH_3)_3$]; solvation results in longer Cr–F bonds and significantly shorter Cr–N bonds in all cases. The more regular structures obtained with solvation are expected based on dielectric shielding and concomi-

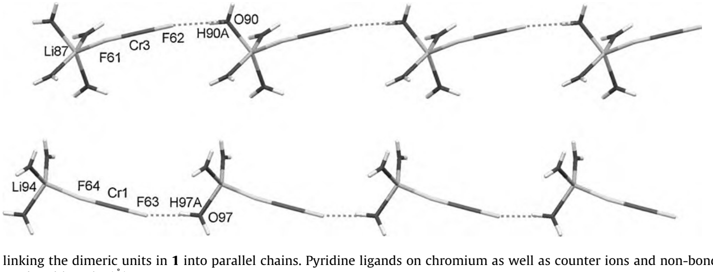

Fig. 6. Hydrogen bonding linking the dimeric units in **1** into parallel chains. Pyridine ligands on chromium as well as counter ions and non-bonded water molecules are omitted for clarity. Hydrogen bond lengths (Å): F62–O97 2.681, F62–O90 2.751.

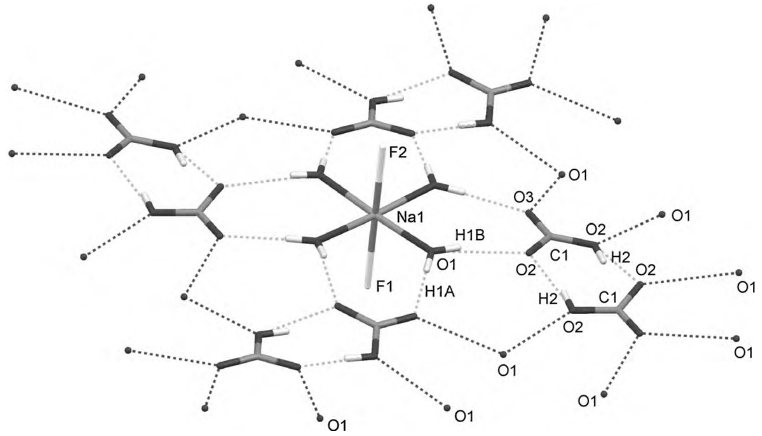

Fig. 8. Packing of the sodium-hydrogencarbonate layers in **2**. Only half of the 50% populated hydrogen positions in the hydrogencarbonate dimers are shown in order to reflect the compound composition. Hydrogen bond lengths (Å): O2–O2 2.621, O1–O2 2.914, O1–O3 2.830.

tant lower repulsion between the fluoride ligands. The similar response of the Cr–F bond length in the *fac*-isomer towards solvation and complexation is also expected since both phenomena results in less ionic contribution to the Cr–F bond. However, the pronounced contraction of the Cr–N bond lengths in *fac*-$[CrF_3(NH_3)_3]$ upon either solvation or complexation was not to be expected and demonstrates an inverse ligand–metal bond length correlation between the two types of ligands, which has the potential to invalidate the common assumption of transferability of ligand-field parameters between related complexes. Actually, the calculated Cr–N bond length contraction of 1.5–2.5% upon solvation (complexation) would lead to an increase of $e_\sigma{}^N$ of ca. 10% (assuming $\Delta_O \propto r^{-5}$) or ca $2000\,cm^{-1}$, which is close in magnitude to the observed shifts.

Direct calculation of the solvent dependence of the spectra was attempted. TDDFT yields a poor reproduction of data producing a position of the first spin-allowed band which is 5000–7000 cm^{-1} too high in energy. The spectral shift upon going from vacuum to water is calculated ca. three times too large with the correct sign for the meridional complex, but with the wrong sign for the facial complex. It is, however, well known that TDDFT has pronounced shortcomings in reproducing positions of *d*–*d* transitions [28]. The

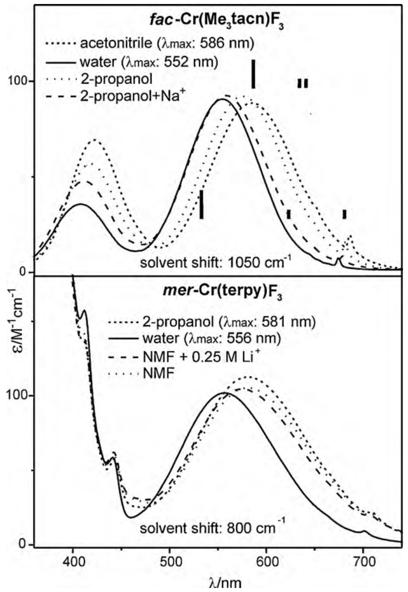

Fig. 9. Absorption spectra of *fac*-$[CrF_3(Me_3tacn)]$ (top) and *mer*-$[CrF_3(terpy)]$ (bottom) in various solvents and with addition of cations. The listed solvent shifts are maximal differences in the position of the $^4A_2(O) \rightarrow {}^4T_2(O)$ transition. In the top figure, the calculated band positions (SLT, *vide supra*) are shown by vertical lines. The upper lines are without and the lower ones with solvation (water, COSMO).

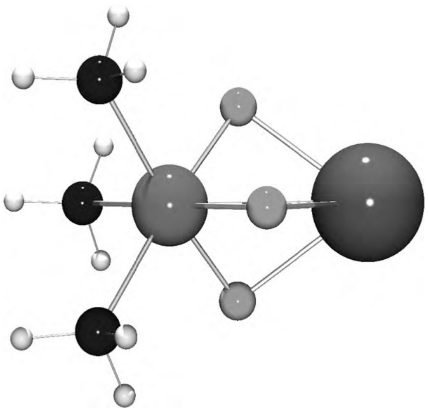

Fig. 10. DFT-optimized geometry for the adduct $\{Na\bullet fac\text{-}[CrF_3(NH_3)_3]\}^+$ in solution. No symmetry constraints were imposed on the optimization.

Table 1
DFT-optimized geometries for *mer*-$[CrF_3(NH_3)_3]$, *fac*-$[CrF_3(NH_3)_3]$ and {Na•*fac*-$[CrF_3(NH_3)_3]$}$^+$.

	mer-$[CrF_3(NH_3)_3]$		*fac*-$[CrF_3(NH_3)_3]$		{Na•*fac*-$[CrF_3(NH_3)_3]$}$^+$	
	Vacuum	Water (COSMO)	Vacuum	Water (COSMO)	Vacuum	Water (COSMO)
$(Cr–F)_{av.}$	1.873 Å	1.904 Å	1.860 Å	1.899 Å	1.877 Å	1.910 Å
$(Cr–N)_{av.}$	2.133 Å	2.106 Å	2.172 Å	2.114 Å	2.124 Å	2.094 Å
$(Na–F)_{av.}$	–	–	–	–	2.325 Å	2.308 Å
$(F–Cr–F)^{cis}{}_{av.}$	95.8°	91.6°	99.1°	92.7°	89.5°	88.0°
$(N–Cr–N)^{cis}{}_{av.}$	96.0°	90.6°	97.3°	90.6°	96.5°	91.9°

Slater Transition State method (SLT) has, on the other hand, been remarkably successful in calculation of *d*–*d* spectra and it does reasonably well also in this case. Band positions are calculated ca. 1000 cm^{-1} too high in water and with solvent shifts of the right sign between vaccum and water. The shifts calculated are 2100 cm^{-1} and 2600 cm^{-1} for the *mer*- and *fac*-complexes, respectively. The overestimated magnitude of the solvent shifts together with the slightly high energy calculated for the $^4A_2(O) \rightarrow {}^4T_2(O)$ transition in water has the consequence that the calculations in vacuum actually yield the experimental band positions in the lower polarity solvents very well. Data for the SLT calculations with and without solvation for the first spin-allowed and the first two spin-forbidden transitions are included as vertical lines in Fig. 9.

3. Experimental

3.1. Materials

The chromium complexes *trans*-$[CrF_2(py)_4](NO_3)$ [29], $CrF_3(py)_3$ [30] and the ligand Me_3tacn [31] were synthesized according to the literature methods. terpy, LiCl (Aldrich) and $Na(Bph_4)$ (Merck) were obtained commercially and used as received. All solvents were used as received.

3.2. Syntheses

3.2.1. trans-$[CrF_2(py)_4][Cr(py)_4F(\mu\text{-}F)Li(H_2O)_3][Cr(py)_4F(\mu\text{-}F)Li(H_2O)_4]Cl_5{\cdot}6H_2O$ (1)

trans-$[CrF_2(py)_4]NO_3$ (10.071 g; 21.5 mmol) was dissolved in water (200 ml). The resulting violet solution was filtered through a wad of glass wool and solid LiCl (21.539 g; 0.508 mol) was added in batches of approximately 7 g, each batch being added only after the prior was completely dissolved. The solution was heated to 50 °C and subsequently cooled in an ice-water bath. After ca. 45 min crystallization of pink product commences. The product was isolated on a fritted filter funnel. Yield: 6.810 g (57.8% of theoretical based on chromium). Analysis: calcd. for $C_{60}H_{86}Li_2N_{12}O_{13}F_6Cl_5Cr_3$: H, 5.27%; C, 43.82%; N, 10.22%. Found: H, 5.04.%; C, 43.48%; N, 10.27%.

3.2.2. catena-$[Na(H_2O)_4][Cr(py)_4F_2](HCO_3)_2$ (2)

trans-$[CrF_2(py)_4]NO_3$ (10.031 g, 21.4 mmol) was dissolved in water (200 ml). The resulting violet solution was filtered through a wad of glass wool before heating up to 30 °C with stirring whereupon anhydrous $NaHCO_3$ (10.015 g, 119.2 mmol) was added. The addition of $NaHCO_3$ resulted in a cloudy reaction mixture and a change in color towards pink. Within a few minutes a pink solid precipitated, but dissolved again shortly after with the

Table 2
Refinement data for 1, 2, 3, 4, and 6. All data measured at 122(1) K.

	**1**	**2**	**3**	**4**	**6**
Molecular formula	$H_{86}C_{60}N_{12}O_{13}F_6Cl_5Li_2Cr_3$	$H_{30}C_{22}N_4O_{10}F_2Na_1Cr_1$	$C_{15}H_{16\cdot11}CrF_3N_3O_{2.56}$	$C_{198.84}H_{197.36}B_4Cr_6F_{18}N_{18}Na_4O_{13.61}$	$C_{78}H_{114}B_2N_9O_3F_9Na_2Cr_3$
Molecular weight	1644.585	623.485	388.32	3846.12	1620.38
Crystal system	Orthorhombic	Tetragonal	Monoclinic	Triclinic	Triclinic
Space group	$Pna2_1$	$P4/ncc$	$P21/c$	$P\bar{1}$	$P\bar{1}$
a (Å)	17.8140 (15)	12.5740(19)	10.2811(5)	16.126(3)	17.228(2)
b (Å)	9.036 (4)	12.5740(19)	9.7077(6)	17.1530(18)	17.417(2)
c (Å)	47.539 (5)	17.271(3)	16.1058(10)	20.433(2)	17.5370(18)
α (°)	90.00	90.00	90.00	104.670(9)	91.713(15)
β (°)	90.00	90.00	100.101(5)	103.809(8)	118.118(13)
γ (°)	90.00	90.00	90.00	112.454(10)	115.164(7)
V (Å^3)	7652 (3)	2730.6(6)	1582.54(15)	4684.1(12)	4022.0(8)
Z	12	4	4	1	2
F_{000}	3660	1292	794.0	1798	1708
D_{cal} (Mg m^{-3})	1.548	1.517	1.630	1.363	1.338
μ (mm^{-1})	0.72	0.51	0.777	0.419	0.482
Crystal size (mm)	0.34 × 0.31 × 0.24	0.46 × 0.23 × 0.20	0.282 × 0.074 × 0.059	0.177 × 0.165 × 0.104	0.068 × 0.079 × 0.155
Color	Pink	Pink	Violet	Light purple	Purple
θ range (°)	1.7–30.1	2.9–45.6	2.0–30.0	1.39–26.54	1.35–30.13
h	−25 → 25	−20 → 25	−14 → 14	−20 → 20	−24 → 24
k	−12 → 11	−25 → 25	−13 → 13	−21 → 21	−24 → 24
l	−66 → 66	−25 → 34	−22 → 22	−25 → 25	−24 → 24
Absorb. correction T_{min}, T_{max}	0.820, 0.886	0.858, 0.919	0.847, 0.961	0.916, 0.965	0.781, 0.942
No. measured reflections	69341	79590	47110	153838	104189
No. independent reflections	19719	5784	4600	19468	23630
No. reflections with $I > 2\sigma(I)$	14647	4307	3698	14813	15354
R_{int}	0.039	0.0639	0.0611	0.0683	0.0743
Number of ref. parameters	910	91	227	1205	1097
$R[F^2 > 2\sigma(F^2)]$	0.047	0.0330	0.0504	0.0708	0.0601
$wR(F^2)^{\dagger}$	0.116	0.0802	0.1251	0.1656	0.1277
S (Goodness of fit)	1.00	1.038	1.09	1.132	1.075
$\Delta\rho_{min}$, $\Delta\rho_{max}$(e Å^{-3})	−0.76, 0.97	−0.805, 1.047	−0.73, 0.94	−0.798, 1.904	−0.841, 1.525

continued warming. At a temperature of 50 °C the beaker was placed on ice and left for crystallization for ca. 1 h.

An intensely pink, crystalline product was isolated by filtration through a fritted filter funnel (porosity G3) and dryed by suction before placing it in a dessicator with conc. sulfuric acid. Yield: 10.886 g (81.5% of theoretical based on chromium). Analysis: calcd. for $C_{22}H_{30}N_4O_{10}F_2Na_1Cr_1$: H, 4.85%; C, 42.38%; N, 8.99%. Found: H, 4.58%; C, 42.39%; N, 8.90%.

MS (FAB + /m-NBA) *m*/*z* (relative intensity): 406.0 ($[Cr(py)_4F_2]^+$), 327.0 ($[Cr(py)_3F_2]^+$), 248.0 ($[Cr(py)_2F_2]^+$), 229.0 ($[Cr(py)_2F]^{2+}$).

Crystals suitable for X-ray were obtained by a modification of the above procedure: the reaction was performed at a smaller scale (ca. 1/50) and with further addition of water so that the reactants could be dissolved completely by heating to 65 °C on a water bath. Upon filtering and cooling, the reaction mixture was left for crystallization giving crystals of X-ray quality upon 2–3 h standing.

3.2.3. $[CrF_3(terpy)]\cdot 2.5H_2O$ (3)

Crude *mer*-$CrF_3(py)_3$ (0.870 g, 2.51 mmol) and 2,2′;6′,2″-terpyridine (0.600 g, 2.57 mmol) were suspended in 2-methoxyethanol (30 ml) and heated to reflux for 2 h. The solution was evaporated to 1/3 of the volume and cooled to RT. Water (0.5 ml) and acetone (30 ml) was added resulting in precipitation of a violet crystalline product. Yield 0.801 g (82%). The procedure yielded directly crystals suitable for X-ray diffraction. Analysis: calcd. for $C_{15}H_{16}N_3O_{2.5}F_3Cr$: H, 4.16%; C, 46.52%; N, 10.85%. Found: H, 4.09.%; C, 46.07; N, 10.81%.

3.2.4. $6[CrF_3(terpy)]\cdot 4Na(Bph_4)\cdot 6MeOH$ (4)

Solutions of $[CrF_3(terpy)]\cdot 2.5H_2O$ (40 mg, 0.10 mmol) in methanol (2 ml) and $Na(Bph_4)$ (40 mg, 0.11 mmol) in methanol (2 ml) were allowed to diffuse slowly together resulting in reddish crystals suitable for X-ray diffraction. Yield 44 mg (71%). The crystals loose methanol on standing. Analysis: calcd. for $C_{192}H_{170}B_4N_{18}O_6F_{18}Na_4Cr_6$: C, 63.80%; H, 4.74%; N, 6.97%. Found: C, 63.55%; H, 4.68%; N, 7.03%.

3.2.5. $[CrF_3(Me_3tacn)]\cdot 3.5H_2O$ (5)

Crude *mer*-$CrF_3(py)_3$ (1.00 g, 2.89 mmol) and Me_3tacn (0.55 g, 3.21 mmol) were dissolved in DMF (7 ml) and refluxed for 20 min. The resulting dark blue solution was cooled to 5 °C and filtered to yield 0.74 g (91%) of dark blue microcrystals which turn pinkish upon exposure to moisture. The crude product was dissolved in water (30 ml) whereto acetone (600 ml) was slowly added yielding 0.66 g of reddish needle shaped crystals. Analysis: calcd. for $C_9H_{28}N_3O_{3.5}F_3Cr$: H, 8.22%; C, 31.49%; N, 12.24%; Cr, 15.14%. Found: H, 8.24.%; C, 30.64; N, 11.89%; Cr, 16.45%. The discrepancies between elemental analyses and calculated composition of the X-ray structure reflects the varying degree of hydration of this compound, which can also be obtained in an anhydrous purple form.

3.2.6. $3[CrF_3(Me_3tacn)]\cdot 2Na(Bph_4)\cdot 2H_2O\cdot 2$-propanol (6)

Dilute solutions of $[CrF_3(Me_3tacn)]\cdot 3.5H_2O$ (12 mg, 0.035 mmol) in 2-propanol (5 ml) and $Na(Bph_4)$ (10 mg, 0.029 mmol) in 2-propanol (5 ml) were allowed to diffuse slowly together resulting in pink needle shaped crystals suitable for X-ray diffraction. Yield 15 mg (79%). Analysis: calcd. for $C_{78}H_{115}B_2N_9O_3F_9Na_2Cr_3$: C, 57.78%; H, 7.15%; N, 7.77%. Found: C, 58.02%; H, 7.13%; N, 7.68%.

3.3. Instruments and measurement

UV/vis spectra were recorded on a Perkin-Elmer, Lambda 2 UV/vis spectrophotometer. Elemental analysis for C, H and N was performed with a CE Instrument: FLASH 1112 series EA, at the microanalytic laboratory, University of Copenhagen. Elemental analysis for Cr and Na was done by AAS on a Perkin-Elmer 2280, and by flame emission on a Kipp H45 emission photometer, respectively. Fast-atom Bombardment Mass spectrometry (FAB+) was done on a Jeol JMS-HX 110 tandem mass spectrometer using *m*-nitrobenzyl alcohol as matrix. X-ray crystallographic data were collected on a Nonius KappaCCD diffractometer using graphite-monochromated Mo $K\alpha$ radiation. Crystal structure and refinement data for 1, 2, 3, 4 and 6 are summarized in Table 2. For all five compounds, all non-hydrogen atoms were refined with anisotropic temperature factors. The molecular structure diagrams were made with the ORTEP-3 program [32].

3.4. DFT calculations

Calculations were performed with the Amsterdam Density Functional (ADF) program suite version 2003.02 or 2007.01 [33]. Slater-type orbital basis sets of triple-ζ quality for the valence orbitals were employed with polarization functions on the ligand atoms and additional valence p orbitals on Cr (ADF basis set TZ2P). Calculations were unrestricted, all-electron calculations with no frozen cores. Calculations were done using gradient corrected functionals employing the VWN LDA exchange-correlation functional supplemented with the non-local, Becke exchange [34] and Perdew correlation [35] functionals. No use of symmetry was made. All charge and spin densities were based on Mulliken analyses. Transition energies were evaluated by either Slater's transition state method [36] or the built-in TDDFT facilities of the program.

4. Conclusions

Several examples of minerals are known which contain fluoride complexes bridged by alkali metals *e.g.* $NaBF_4$ (ferruccite), Na_3AlF_6 (cryolite), K_2NaAlF_6 (elpasolite), Na_2LiAlF_6 (simmonsite). Here, it has been shown that similar structures can be targeted synthetically. Fluoride coordinated to chromium(III) interacts with protic solvents and small unpolarizable ions in solution as well as in the solid state. Efficient clustering of alkali metal cations by bridging uncharged complexes constitutes a new approach towards heterometallic systems. The pronounced tendency towards linear bridging by fluoride in these systems may render these interactions important in crystal engineering and in geometric control of polynuclear systems. Currently efforts along these lines are being undertaken. A strong effect on the auxiliary ligands by perturbation of coordinated fluoride ligands has been identified by DFT modeling. This result signals a warning regarding interpreting solvatochromism and spectral interaction with alkali metal cations in the framework of the traditional additive ligand-field models.

Supplementary data

Crystallographical data of *catena*-$[Na(H_2O)_4][Cr(py)_4F_2](HCO_3)_2$, *trans*-$[CrF_2(py)_4][Cr(py)_4F(\mu\text{-}F)Li(H_2O)_3][Cr(py)_4F(\mu\text{-}F)Li(H_2O)_4]Cl_5\cdot 6H_2O$, $[CrF_3(terpy)]\cdot 2.5H_2O$, $6[CrF_3(terpy)]\cdot 4Na(Bph_4)\cdot 6MeOH$, and $3[CrF_3(Me_3tacn)]\cdot 2Na(Bph_4)\cdot 2H_2O\cdot 2$-propanol, and have been deposited with the Cambridge Crystallographic Data Centre allocated with the deposit numbers CCDC 767058, CCDC 767059, CCDC 767060, CCDC 767062, and CCDC 767061, respectively. Copy of the data can be obtained free of charge on application to CCDC, 12 Union Road, Cambridge C2B 1EZ, UK, fax: +44 1223 336033, e-mail: deposit@ccdc.cam.ac.uk. Input and partial ADF output for geometry optimization on *fac*-$CrF_3(NH_3)_3$ as well as input for the Slater-TS calculation of the lowest quartet–quartet transition is available as supplementary information.

Acknowledgements

Prof. C.E. Schäffer and assoc. prof. J. Glerup are thanked for valuable discussions. The authors gratefully acknowledges financial support from the Danish Research Councils (JB, SP and HW grants).

Appendix A. Supplementary data

Supplementary data associated with this article can be found, in the online version, at doi:10.1016/j.jfluchem.2010.06.003.

References

[1] G.A. Jeffrey, An Introduction to Hydrogen Bonding, Oxford University Press, Oxford, UK, 1997.
[2] C.J.D. Craig, M.H. Brooker, J. Sol. Chem. 29 (2000) 879–888.
[3] J. Emsley, D.J. Jones, R.S. Osborn, J. Chem. Soc., Chem. Commun. 15 (1980) 703–704.
[4] L. Cheng, X. Xu, Y. Xu, Acta Crystallogr. E 64 (2008) m82.
[5] A.J. Norquist, C.L. Stern, K.R. Poeppelmeier, Inorg. Chem. 38 (1999) 3448–3449.
[6] N.N. Greenwood, A. Earnshaw, Chemistry of the Elements, 2nd ed., Butterworth & Heinemann, Amsterdam, 2006.
[7] M.R. Marvel, R.A.F. Pinlac, J. Lesage, C.L. Stern, K.R. Poeppelmeier, Z. Anorg. Allg. Chem. 635 (2009) 869–877.
[8] M.R. Marvel, J. Lesage, J. Baek, P.S. Halasyamani, C.L. Stern, K.R. Poeppelmeier, J. Am. Chem. Soc. 129 (2007) 13963–13969.
[9] R.Z. LeGeros, R. Kijkowska, W. Jia, J.P. LeGeros, J. Fluorine Chem. 41 (1988) 53–64.
[10] S. Kaizaki, H. Takemoto, Inorg. Chem. 29 (1990) 4960–4964.
[11] Y. Terasaki, S. Kaizaki, J. Chem. Soc., Dalton Trans. (1995) 2837–2841.
[12] Y. Terasaki, T. Fujihara, T. Schönherr, S. Kaizaki, Inorg. Chim. Acta 259 (1999) 84–90.
[13] R.J. Bianchini, U. Geiser, H. Place, S. Kaizaki, Y. Morita, J.I. Legg, Inorg. Chem. 25 (1986) 2129–2134.
[14] A. Bodner, P. Jeske, T. Weyhermuller, K. Wieghardt, E. Dubler, H. Schmalle, B. Nuber, Inorg. Chem. 31 (1992) 3737–3748.
[15] P. Yu, E.F. Murphy, H.W. Roesky, P. Lubini, H.-G. Schmidt, M. Noltemeyer, Organometallics 16 (1997) 313–316.
[16] B.F. Straub, F. Rominger, P. Hofmann, Inorg. Chem. 39 (2000) 2113–2119.
[17] L.F. Jones, C.A. Kilner, M.P. de Miranda, J. Wolowska, M.A. Halcrow, Angew. Chem., Int. Ed. 46 (2007) 4073–4076.
[18] D. Riou, F. Taulelle, G. Ferey, Inorg. Chem. 35 (1996) 6392–6395.
[19] D. Stalke, F.-Q. Liu, H.W. Roesky, Polyhedron 15 (1996) 2841–2843.
[20] T. Birk, J. Bendix, H. Weihe, Acta Crystallogr. E64 (2008) m369–m370.
[21] G. Fochi, J. Strahle, F. Gingl, Inorg. Chem. 30 (1991) 4669–4671.
[22] S.G. Thoma, F. Bonhomme, M. Nyman, M.A. Rodriguez, T.M. Nenoff, J. Fluorine Chem. 108 (2001) 73–77.
[23] N. Cloete, H.G. Visser, A. Roodt, Acta Crystallogr. E63 (2007) m45–m47.
[24] (a) K.R. Heier, J.A. Norquist, C.G. Wilson, C.L. Stern, K.R. Poeppelmeier, Inorg. Chem. 37 (1998) 76–80;
(b) P.C.R. Guillory, J.E. Kirsch, H.K. Izumi, C.L. Stern, K.R. Poeppelmeier, Cryst. Growth Des. 6 (2006) 382–389.
[25] (a) J. Bendix, A. Bøgevig, Inorg. Chem. 37 (1998) 5992–6001;
(b) J. Bendix, A. Bøgevig, Acta Crystallogr. C 54 (1998) 206–208.
[26] (a) C.C. Pye, T. Ziegler, Theor. Chem. Acc. 101 (1999) 396–408;
(b) A. Klamt, G. Schüürmann, J. Chem. Soc., Perkin Trans. 2 (1993) 799–805.
[27] (a) N. Buchholz, M. Leimkuhler, L. Kiriazis, R. Mattes, Inorg. Chem. 27 (1988) 2035–2039;
(b) L. Kiriazis, R. Mattes, Z. Anorg. Allg. Chem. 593 (1991) 90–98.
[28] F. Neese, J. Biol. Inorg. Chem. 11 (2006) 702–711.
[29] J. Glerup, J. Josephsen, E. Michelsen, E. Pedersen, C.E. Schäffer, Acta Chem. Scand. 24 (1970) 247–254.
[30] N. Costachescu, J. Chem. Soc. 102 (1912) 493–499.
[31] K. Wieghardt, P. Chaudhuri, B. Nuber, J. Weiss, Inorg. Chem. 21 (1982) 3086–3090.
[32] L.J. Farrugia, J. Appl. Crystallogr. 30 (1997) 565–566.
[33] (a) G. te Velde, E.J. Baerends, Comput. Phys. 99 (1992) 84–98;
(b) G. te Velde, F.M. Bickelhaupt, S.J.A. van Gisbergen, C.F. Guerra, E.J. Baerends, J.G. Snijders, T. Ziegler, J. Comp. Chem. 22 (2001) 931–967.
[34] A.D. Becke, Phys. Rev. A38 (1988) 3098–3100.
[35] (a) J.P. Perdew, Phys. Rev. B33 (1986) 8822–8824;
(b) J.P. Perdew, Phys. Rev. B34 (1987) 7406 (erratum).
[36] J.C. Slater, Quantum Theory of Molecules and Solids, McGraw-Hill, New York, 1974.

PAPER 5

Magnetic properties of a manganese(III) chain with mono-atomic bridges: *catena*-Mn(F)(salen)

Torben Birk, Kasper Steen Pedersen, Stergios Piligkos, Christian Thuesen, Högni Weihe and Jesper Bendix

Inorg. Chem., 2011, 50(12), 5312–5314.

Inorganic Chemistry
COMMUNICATION
pubs.acs.org/IC

Magnetic Properties of a Manganese(III) Chain with Monoatomic Bridges: *catena*-MnF(salen)

Torben Birk, Kasper S. Pedersen, Stergios Piligkos, Christian Aa. Thuesen, Högni Weihe, and Jesper Bendix*

Department of Chemistry, University of Copenhagen, Universitetsparken 5, DK-2100 Copenhagen, Denmark

S Supporting Information

ABSTRACT: In the solid state, MnF(salen) forms chains wherein fairly linear fluoride bridges between high-spin Mn^{III} centers are observed. We interpret the magnetic properties of these chains by use of the classical Fisher model and by use of the high-temperature expansion approach, as well as by exact matrix diagonalization of the spin Hamiltonian, of model rings. In solution, electron paramagnetic resonance shows the chains to be symmetrically cleaved to monomeric MnF(salen).

In recent years, the fluoride ligand has received increasing focus as a bridging ligand for incorporation in magnetic materials because of its spectroscopic and redox innocence and because it exhibits a distinct preference to forming linear bridges, providing thus the means for cluster design.[1] Surprisingly, the most ubiquitous building block[2] in molecule-based magnetic systems such as single-molecule[3,4b] and single-chain magnets,[4] Mn^{III}(salen)$^{+}$ has not been structurally or magnetically investigated with fluoride coligands. The synthesis of the title compound from MnF_3 and H_2salen, in methanol, is straightforward (Supporting Information, SI). The chain structure of **1** is shown in Figure 1. In **1**, the equatorial Schiff-base coordination resembles that found in other Mn^{III}(salen) complexes.[2,5] The propensity of fluoride for forming linear bridges and the steric demands of the salen ligand result in the linear chain structure of **1** in which three crystallographically inequivalent Mn^{III} atoms exist in the unit cell ($P\bar{1}$) and three different bridging Mn−F−Mn angles are observed, namely, 150.43(4)° (Mn2−F3−Mn3), 151.72(3)° (Mn1−F2−Mn2), and 180.0° (Mn1−F1−Mn1 and Mn3−F4−Mn3). In the crystal structure of **1**, the F atoms labeled F1 and F4 are located on inversion centers. Similar linear fluoride bridges are observed in [V(O)(salen)(μ-F)V(O)(salen)](BF_4), wherein V−F−V angles range from 171.96° to 173.50°.[6] A comparison of the Mn−F bond lengths in **1** with the bond lengths in MnF_3 shows that the Mn^{III} centers in **1** are tetragonally elongated by Jahn−Teller distortion.[7]

The formation of chains from the association of Mn^{III}(salen)$^{+}$ fragments with polyatomic bridges has been frequently observed, e.g., in Mn(X)(salen) (where X = CH_3COO, NO_3, CN, N_3).[8] However, only a few examples of dimers of Mn^{III}(salen) units linked by monatomic bridges have been characterized, and chains of M(salen) complexes with monatomic bridges have not been reported for any metal centers.[9]

The magnetic moment of **1** was measured in the temperature range 1.8−370 K in a 1 kOe direct-current magnetic field. The obtained data are shown in Figure 2 in the form of magnetic susceptibility, χ, and of the χT product. In Figure 2 are also shown the results obtained from modeling of the magnetic data by use of various approaches. The high-temperature value of the χT product (1.84 cm^3 K mol^{-1} at 370 K) is significantly lower than the value expected for an isolated high-spin Mn^{III} ion (3.00 cm^3 K mol^{-1} for $g = 2$). Upon cooling, the χT product steadily decreases to a minimum value of 0.01 cm^3 K mol^{-1} at 1.8 K. These two observations imply dominant antiferromagnetic intrachain interactions between the manganese centers. This conclusion is further supported by the observation that magnetization of **1** at $T = 1.8$ K does not saturate at a field of 50 kOe (see the SI, Figure SI1), as was expected for strongly antiferromagnetically coupled systems.

The χT product of **1** shows no evidence of spin-canting behavior, which would be manifested by a slight increase of χT at low temperatures.[10] In fact, the existence of the inversion center in the chain structure of **1**, in conjuction with the topology of the three inequivalent Mn^{III} centers in the asymmetric unit of **1**, results in the complete cancellation of local magnetic moments. This is further explained by qualitative arguments in Figure SI2 of the SI. However, a residual magnetization persists at low temperatures, as can be seen in Figures 2 and SI1 of the SI. This residual magnetization can be due to small amounts of monomeric Mn^{III} impurities. Finally, alternating-current susceptibility measurements down to 1.8 K do not reveal slow relaxation of the magnetization behavior or the presence of a magnetic phase transition.

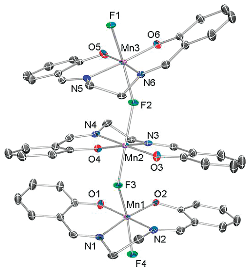

Figure 1. Molecular structure of MnF(salen). Displacement ellipsoids are drawn at 50% probability. H atoms are omitted for clarity. Bond length intervals (Å): Mn−O 1.8925(8)−1.9119(9), Mn−N 1.9949(9)−2.0059(8), Mn−F 2.049(2)−2.096(2). Angle intervals (deg): Mn−F−Mn 150.43(4)−180.0.

Received: February 8, 2011
Published: May 19, 2011

ACS Publications © 2011 American Chemical Society

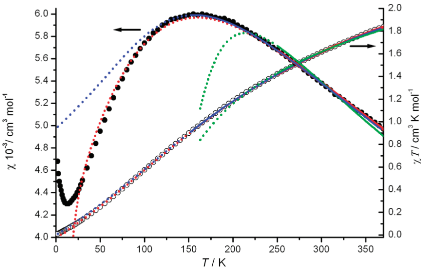

Figure 2. Magnetic susceptibility and χT product data for **1**. Solid and hollow circles correspond to experimental susceptibility and χT product data, respectively. The theoretical best-fit curves obtained in the temperature range 370−250 K by full-matrix diagonalization of nonanuclear models and by high-temperature expansion and in the temperature range 370−100 K by the Fisher model are shown as solid red, green, and blue lines, respectively. Extensions of these curves at lower temperatures, using the best-fit J parameter, are shown as dotted lines.

We used various models for interpretation of the magnetization data of **1**, namely, the classical Fisher model[11] and the quantum-mechanical models of high-temperature expansion[12] and of the exact matrix diagonalization of the spin Hamiltonian, of model systems. Within the Fisher model, the analytical expression for the magnetic susceptibility of an infinite chain of classical spins is derived. The magnetic susceptibility of **1** was fitted to the isotropic spin Hamiltonian (1)

$$\hat{H} = J\sum_{1}^{N}\hat{S}_i\cdot\hat{S}_{i+1} + \sum_{1}^{N}\mu_B B g\hat{S}_i \qquad (1)$$

by use of the analytical expression (2), giving the magnetic susceptibility of an infinite chain of classical spins, derived by Fisher.

$$\chi = \frac{N_A g^2 \mu_B S(S+1)}{3k_B T}\frac{1+u}{1-u};$$
$$u = \coth\left(-\frac{6J}{k_B T}\right) + \frac{k_B T}{6J} \qquad (2)$$

A satisfactory fit to the experimental data was obtained only down to temperatures of about 100 K (Figure 2). At temperatures lower than 100 K, the classical Fisher model fails because at low temperatures quantum effects gain importance. The exchange-coupling interaction was found to be $J = 38$ cm^{-1} (for $g = 2$), comparable to purely inorganic one-dimensional fluoride-bridged Mn^{III} systems based on *trans*-$[MnF_4F_{2/2}]^{2-}$ units.[13] The only structurally characterized example of chloride-bridged Mn^{III} chains is *catena*-Mn(bipy)Cl_3, which has longer (2.503−2.763 Å) and more bent (135.09°) bridges with concomitant weaker coupling: $J \approx 24$ cm^{-1}.[14]

Interpretation of the magnetic properties of **1** by exact quantum treatment is impossible, given that, except in the case of antiferromagnetic spin half-chains,[15] no analytical solution exists for this problem for infinite systems. Thus, we followed an approach based on extrapolation to infinity of the results obtained, by exact or high-temperature expansion treatments, on increasing dimension model systems. This general strategy was first presented in pioneering work by Bonner and Fisher.[11b] The model systems that we use herein are antiferromagnetically

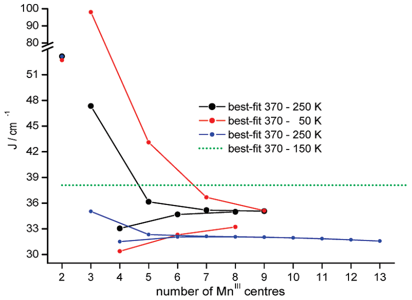

Figure 3. Isotropic exchange, J, values determined by fitting the experimental data to various ring sizes and models (black and red, full-matrix diagonalization; blue, high-temperature expansion; green, Fisher model).

coupled rings consisting of Mn^{III} ions presenting only first-neighbor interactions, as expressed by (1).

The high-temperature expansion approach consists of computation of the partition function of the system by use of a series expansion of the exponential of the thermally weighted spin Hamiltonian of the system. A brief description of this approach is given in the SI. The details of our implementation of this approach have been discussed elsewhere.[16] By use of the high-temperature expansion up to the 13th order, for our model ring systems, we were able to fit the experimental data in the temperature range 370−250 K (Figure 2). At lower temperatures, the 13th-order high-temperature expansion is not sufficient to accurately describe the magnetic properties of **1**. Higher order expansions are needed for this purpose. However, this is computationally very demanding. The best-fit J parameters (for $g = 2$) determined by the high-temperature expansion approach for model systems of nuclearity ranging from 2 to 13 are shown in Figure 3. The theoretical curves obtained from the best-fit J parameter in the case of a nonanuclear ring are shown as solid lines in Figure 2, where is also shown the extension of these theoretical curves at lower temperatures (dotted lines). The best-fit J parameters determined by the high-temperature expansion approach asymptotically converge to a limit of about 32 cm^{-1}, as the nuclearity increases. This limit corresponds to the antiferromagnetic exchange interaction in the infinite chain. However, with increasing nuclearity, a slight divergence from this limit is observed (Figure 3). This reflects the fact that, at constant expansion order (here the 13th order), the model becomes less accurate as the nuclearity increases. This is further illustrated by the fact that only for a dinuclear system the result obtained by the high-temperature expansion exactly matches the solution obtained by full-matrix diagonalization.

The best-fit J parameters (for $g = 2$) determined, in two different temperature ranges, by full-matrix diagonalization of the spin Hamiltonian (1) for model systems of nuclearity ranging from 2 to 9 are also shown in Figure 3. Our analysis was limited to nuclearity 9 because we block-diagonalized the isotropic exchange spin Hamiltonian (1) by exploiting only the symmetries related to the total spin, S, and its projections along the quantization axis, S_z. As in the case of the high-temperature expansion, the determined values of J converge asymptotically toward a limit with increasing nuclearity. In the case that the fit has been performed in the same temperature range as that for the high-temperature

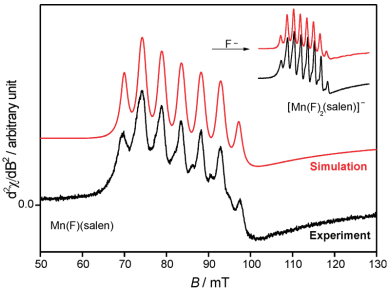

Figure 4. Parallel-mode EPR spectrum of Mn(F)(salen) in frozen NMF glass at T = 5.65 K and ν = 9.416 425 GHz. The inset shows the effect of the addition of excess F^-. Simulation parameters (for Hamiltonian, see ref 16): [Mn(F)(salen)], g_z = 7.92 cm^{-1}, Δ = 0.0555 cm^{-1}, A_z = 0.017 25 cm^{-1}, $A_{z,ax}$ = 0.0145 cm^{-1}; *trans*-[MnF_2(salen)]$^-$, g_z = 7.92 cm^{-1}, Δ = 0.071 30 cm^{-1}, A_z = 0.017 60 cm^{-1}, $A_{z,ax}$ = 0.016 95 cm^{-1}.

expansion (370−250 K), the antiferromagnetic exchange interaction is estimated at around 35 cm^{-1}. In the case that the fit is extended at lower temperatures (370−50 K), the determined antiferromagnetic exchange interaction is estimated at around 34 cm^{-1}. For the sake of clarity, only the curves related to the full-matrix diagonalization fit in the temperature range 370−250 K are shown in Figure 3, in the case of a nonanuclear ring. However, the corresponding curves obtained by full-matrix diagonalization fit in the temperature range 370−50 K are very similar to the ones shown in Figure 2 because the J values determined for the nonanuclear rings in the two temperature ranges are very similar (Figure 3).

We recently found that parallel-mode electron paramagnetic resonance (EPR) on frozen glasses is capable of resolving superhyperfine couplings to fluoride bound to Mn^{III} in MnF_6^{3-}.[17] These Jahn−Teller-distorted systems are characterized by strongly anisotropic superhyperfine couplings, with the coupling to the distant fluorides on the Jahn−Teller-elongated axis being much larger (by a factor of ca. 4) than the coupling to the more closely bound equatorial fluorides. Application of this knowledge to the parallel-mode EPR of NMF solutions of **1** clearly demonstrates that the chain upon dissolution forms neutral [MnF(salen)] with undetectable amounts of complexes with more than one fluoride ligand (cf. Figure 4). The addition of excess F^- to the NMF solution before freezing yields clean conversion to *trans*-[MnF_2(salen)]$^-$.

■ ASSOCIATED CONTENT

Ⓢ Supporting Information. X-ray structure data (CCDC 796782), outline of the methodology used in the high-temperature expansions, additional magnetic data, and experimental procedures. This material is available free of charge via the Internet at http://pubs.acs.org.

■ AUTHOR INFORMATION

Corresponding Author
*E-mail: bendix@kiku.dk.

■ ACKNOWLEDGMENT

K.S.P. thanks DANSCATT for financial support. S.P. thanks the Danish Natural Science Research Council for a Steno grant.

■ REFERENCES

(1) (a) Larsen, F. K.; McInnes, E. J. L.; Mkami, H. E.; Overgaard, J.; Piligkos, S.; Rajaraman, G.; Rentschler, E.; Smith, A. A.; Smith, G. M.; Boote, V.; Jennings, M.; Timco, G. A.; Winpenny, R. E. P. *Angew. Chem., Int. Ed.* **2003**, *42*, 101–105. (b) Affronte, M.; Carretta, S.; Timco, G. A.; Winpenny, R. E. P. *Chem. Commun.* **2007**, 1789–1797. (c) Meally, S. T.; Mason, K.; McArdle, P.; Brechin, E. K.; Ryder, A. G.; Jones, L. F. *Chem. Commun.* **2009**, 7024–7026. (d) Birk, T.; Schau-Magnussen, M.; Piligkos, S.; Weihe, H.; Holten, A.; Bendix, J. *J. Fluorine Chem.* **2010**, *131*, 898–906.

(2) Miyasaka, H.; Saitoh, A.; Abe, S. *Coord. Chem. Rev.* **2007**, *251*, 2622–2664.

(3) (a) Gatteschi, D.; Sessoli, R. *Angew. Chem.* **2003**, *115*, 278–309. (b) Miyasaka, H.; Clérac, R.; Wernsdorfer, W.; Lecren, L.; Bonhomme, C.; Sugiura, K.-I.; Yamashita, M. *Angew. Chem.* **2004**, *116*, 2861–2865. (c) Pedersen, K. S.; Schau-Magnussen, M.; Bendix, J.; Weihe, H.; Palii, A. V.; Klokishner, S. I.; Ostrovsky, S.; Reu, O. S.; Mutka, H.; Tregenna-Piggott, P. L. W. *Chem.—Eur. J.* **2010**, *16*, 13458–13464.

(4) (a) Clérac, R.; Miyasaka, H.; Yamashita, M.; Coulon, C. *J. Am. Chem. Soc.* **2002**, *124*, 12837–12844. (b) Ferbinteanu, M.; Miyasaka, H.; Wernsdorfer, W.; Nakata, K.; Sugiura, K.; Yamashita, M.; Coulon, C.; Clérac, R. *J. Am. Chem. Soc.* **2005**, *127*, 3090–3099.

(5) (a) Darensbourg, D. J.; Frantz, E. B. *Dalton Trans.* **2008**, 5031–5036. (b) Ni, Z.-H.; Kou, H.-Z.; Zhang, L.-F.; Ge, C.; Cui, A.-L.; Wang, R.-J.; Li, Y.; Sato, O. *Angew. Chem., Int. Ed.* **2005**, *44*, 7742–7745.

(6) Fairhurst, S. A.; Hughes, D. L.; Leigh, G. J.; Sanders, J. R.; Weisner, J. *Dalton Trans.* **1994**, 2591–2598.

(7) Wells, A. F. *Structural Inorganic Chemistry*, 5th ed.; Clarendon Press: Oxford, U.K., 1984; p 324.

(8) (a) Davies, J. E.; Gatehouse, B. M.; Murray, K. S. *J. Chem. Soc., Dalton Trans.* **1973**, 2523–2527. (b) Shyu, H.-L.; Wei, H.-H.; Wang, Y. *Inorg. Chim. Acta* **1999**, *290*, 8–13. (c) Matsumoto, N.; Sunatsuki, Y.; Miyasaka, H.; Hashimoto, Y.; Luneau, D.; Tuchagues, J.-P. *Angew. Chem., Int. Ed.* **1999**, *38*, 171–173. (d) Ko, H. H.; Lim, J. H.; Kim, H. C.; Hong, C. S. *Inorg. Chem.* **2006**, *45*, 8847–8849. (e) Yuan, M.; Gao, S.; Sun, H.-L.; Su, G. *Inorg. Chem.* **2004**, *43*, 8221–8223.

(9) Liu, Y.; Dou, J.; Niu, M.; Zhang, X. *Acta Crystallogr., Sect. E* **2007**, *63*, m2771–m2771.

(10) Mossin, S.; Weihe, H.; Sorensen, H. O.; Lima, N.; Sessoli, R. *Dalton Trans.* **2004**, 632–639.

(11) (a) Fisher, M. E. *J. Am. Phys.* **1964**, *32*, 343–346. (b) Bonner, J. C.; Fisher, M. E. *Phys. Rev. A* **1964**, *135*, 640.

(12) (a) Rushbrooke, G. S.; Wood, P. J. *Mol. Phys.* **1958**, *1*, 257–283. (b) Eifert, T.; Hüning, F.; Lueken, H.; Schmidt, P.; Thiele, G. *Chem. Phys. Lett.* **2002**, *364*, 69–74. (c) Schmidt, H.-J.; Schnack, J.; Luban, M. *Phys. Rev. B* **2001**, *64*, 224415.

(13) Palacio, F.; Morón, M. C. In *Research Frontiers in Magnetochemistry*; O'Connor, C. J., Ed.; World Scientific: Singapore, 1993.

(14) (a) Perlepes, S. P.; Blackman, A. G.; Huffman, J. C.; Christou, G. C. *Inorg. Chem.* **1991**, *30*, 1665–1668. (b) Granroth, G. E.; Meisel, M. W.; Chaparala, M.; Jolicoeur, T.; Ward, B. H.; Talham, D. R. *Phys. Rev. Lett.* **1996**, *77*, 1616–1619.

(15) Bethe, H. A. *Z. Phys.* **1931**, *71*, 205–226.

(16) Thuesen, C. A.; Weihe, H.; Bendix, J.; Piligkos, S.; Mønsted, O. *Dalton Trans.* **2010**, *39*, 4882–4885.

(17) Scheifele, Q.; Birk, T.; Bendix, J.; Tregenna-Piggott, P. L. W.; Weihe, H. *Angew. Chem., Int. Ed.* **2008**, *47*, 148–150.

Supplementary information for

Magnetic properties of a manganese(III) chain with mono-atomic bridges: *catena*-MnF(salen)

Torben Birk, Kasper S. Pedersen, Stergios Piligkos, Christian Aa. Thuesen, Högni Weihe and Jesper Bendix

Experimental procedures:

All chemicals and solvents were purchased from Sigma-Aldrich or Fluka and used as received. The Schiff base ligand H_2(salen) (*N*,*N*'-ethylene-bis-salicylideneimine or 2,2'-[ethane-1,2-diylbis(azanylylidenemethanylyl-idene)]diphenol) was synthesized as described in Mason, T. *Ber. Dtsch. Chem. Ges.*, **1887**, *20(1)*, 267.

Synthesis: H_2(salen) (10 mmol, prepared by literature procedure) was treated with methanol (80 mL) and added solid MnF_3 (10 mmol, Aldrich) and Et_3N (20 mmol). The reaction mixture was heated to reflux for 35 min. added water (5 mL) followed by 5 min. of heating. The reaction mixture was allowed to cool slowly to room temperature before being filtered through a filter of paper and then added ^{t}BuOMe (500 mL) drop-wise with simultaneous stirring. The brown crystalline product was isolated by filtration. Yield 68%. The product was recrystallized from methanol by diffusion of ^{t}BuOMe over a period of several days. Elemental analysis: Elemental analysis calcd. (%) for $H_{14}C_{16}N_2O_2FMn$: H 4.15, C 56.48, N 8.23, F 5.58, Mn 16.15; found H 4.00, C 56.38, N 8.15, F 5.38, Mn 16.41.

The magnetic characterisation was performed on a Quantum Design MPMS-XL SQUID magnetometer equipped with a 5 T dc magnet. Susceptibility measurements were conducted at H_{dc} = 1 kOe in the temperature range 1.8–300 K on a polycrystalline sample in a polycarbonate capsule. The susceptibility was corrected for diamagnetic contributions by means of Pascal constants. For magnetization measurements the field was sweeped to ±5 T at 1.8 K at an average rate of 160 Oe min^{-1}. Ac experiments were conducted at selected frequencies between 50 Hz and 1500 Hz with H_{ac} = 3.8 Oe in the absence of a dc field.

Elemental analysis for C, H and N was performed with a CE Instrument: FLASH 1112 series EA, at the microanalytical laboratory, University of Copenhagen. Whereas F and Mn analysis was performed at the Mikroanalytisches Laboratorium KOLBE, Höhenweg 17, D-45470 Mülheim an der Ruhr.

UV/vis spectra were measured in a 1cm path-length quartz cell using a Perkin Elmer UV/vis lambda 2 spectrophotometer. Mid-range FTIR spectra of the compounds were recorded as KBr-pellets using a Bio-Rad Excalibur Series FTS 30000MX FT-IR spectrometer within the range of 4400–450 cm^{-1}. Fast atom bombardment (FAB, Xe ions, accelerated by 6 kV) mass spectra were recorded on a JEOL JMS-HX/HX110A tandem mass spectrometer (positive and negative ion detection). Matrix for FAB: *m*-nitrobenzyl alcohol (*m*-NBA).

Experimental data from X-ray structure determination

Mn(F)(salen) M_r = 340.237, T = 122(1) K, Triclinic, Space group *P-1*, a = 10.0400(9), b = 15.4490(12), c = 16.0420(17) Å, α =108.164(9), β =103.874(10), γ = 101.140(7)°, V = 2196.3(3) Å^3, Z = 6, D_c = 1.543 g·cm^{-1}, 123564 measured reflections of which 23068 (R_{int} = 0.0506), $R[F^2>2\ \sigma(F^2)]$ = 0.0384, $wR(F^2)$ = 0.0815, CCDC 796782.

Supplementary data:

FT-IR (cm^{-1}): 3428(broad), 1649(s) ν_{str}(C=N) , 1599(s), 1541(s), 1446(s), 1334(m), 1297(s) ν_{str}(C-O), 1195(m), 1147(m), 1127(m), 1029(m), 902(m), 744(s), 732(s), 630(m), 584(m), 460(s) ν_{str}(Mn-N/O). Assignment based on: J. Chakraborty, B. Samanta, G. Pilet, S. Mitra, *Struct. Chem.*, 2006, **17**, 585.

UV/vis (MeOH): λ[nm] $\varepsilon[10^3\ M^{-1}\cdot cm^{-1}]$: 216 (33.57), 236 (37.58), 281 (17.08), 307 (12.35), 349 (60.45), 397 (46.76). MS(FAB+): *m*-NBA *m*/*z* = 340.04 [M^+], 321.08[M-F]. MS(FBA-): *m*-NBA *m*/*z* = 339.42[M-H].

Crystals grown for X-ray by: Mn(F)(salen) in MeOH (0.090 g / 20 mL) is placed together with Et_2O in a vacuum dessicator.

Field-dependence of the magnetic moment of Mn(F)(salen):

The fast rise of the residual molecular magnetization of Mn(F)(salen) with magnetic field at 1.8 K shown in Figure ESI1, indicates the presence of a paramagnetic impurity in the measured polycrystalline sample. Assuming that the magnetic moment saturates at 0.15 μ_B at a magnetic field of about 1 T (linear region) at 1.8 K, this leads to a χT product of the order of 0.17 cm^3 K mol^{-1} at this field and temperature. This is about 5 % of that expected for a Mn(III) paramagnetic species (3 cm^3 K mol^{-1}). In the case that the detected residual magnetization came from the thermal population of excited spin states of total spin different than zero, the molecular magnetization would rise slowly with the magnetic field, eventually presenting a step behavior, in contrast to what observed for Mn(F)(salen).

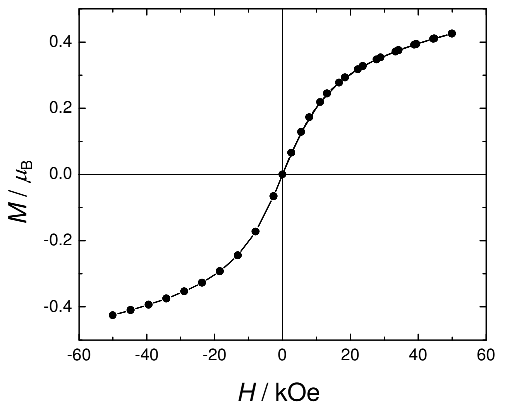

Figure SI1: Field dependence of the molecular magnetization of a polycrystalline sample of Mn(F)(salen) measured at 1.8 K.

Spin canting in Mn(F)(salen):

In Mn(F)(salen), the Mn(III) centers are tetragonally elongated by Jahn-Teller distortion, as discussed in the main manuscript text. Assuming that the local magnetic moment vectors are aligned with the Jahn-Teller distortion axes, there are two possible orientations for the local magnetic moments of the Mn(III) sites. These two directions are defined by the Mn-F bonds. The assumed orientations of the local magnetic moments of the Mn(III) centers in Mn(F)(salen) are depicted in red in Figure SI2. The vector sums of the local magnetic moments of two adjacent Mn(III) sites are depicted in blue in Figure ESI2. One can easily see that the vector sum of the two Mn(III) centers located in the immediate proximity of the inversion center is null. The same is true for the vector sum of the summed magnetic moments resulting from centers Mn1 and Mn2 and centers Mn5 and Mn6. Thus, the macroscopic

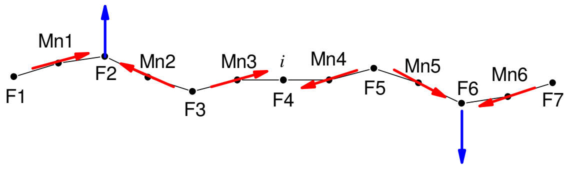

Figure SI2: Cartoon of spin canting in Mn(F)(salen). The origin of the reference system, xyz, is placed on an inversion center, *i*, of the crystal structure of Mn(F)(salen), that also coincides with the location of an F^- bridging ligand (F4). The orientations of the local magnetization vectors are depicted in red and the orientations of the magnetization vectors resulting from the vector sum of two adjacent Mn(III) centers are shown in blue.

magnetic moment in the chain structure of Mn(F)(salen) is zero because of the existence of the inversion center in conjunction with the topology of the Mn(III) centers.
In Figure ESI2 we have assumed some particular orientations for the local magnetic moments. In principle these can be oriented along arbitrary orientations, that have however to be related by the inversion center. Thus, the arguments presented above are also valid for arbitrary orientations of the local magnetic moments. These symmetry considerations also rationalize the absence of ordering in Mn(F)(salen), which is observed for some Mn(III) chains with longer Mn-Mn distances.[1]

High-temperature expansions

For large spin systems, it is computationally impossible to diagonalise the full energy matrix. A useful method to extract spin Hamiltonian parameters from experimental data is instead through the high temperature expansion (HTE).[2-5] In general, the partition function Z can be rewritten[2]:

$$Z = \sum_i \exp(-\beta E_i) = tr\left\{\exp\left(-\beta \hat{H}\right)\right\}$$

Where $\beta = (k_B T)^{-1}$. The summation is over all states and “tr” stands for the trace of the matrix representation. To avoid any diagonalisation, in the HTE, the exponential is evaluated as a power series around $\beta = 0$:

$$Z = \sum_{n=0}^{\infty} \frac{(-\beta)^n}{n!} \mathrm{tr}\{\hat{H}^n\}$$

From this, the susceptibility may be calculated in the usual way[3]:

$$\chi_\mathrm{m} = \mu_0 N_\mathrm{A} \beta^{-1} \frac{\partial^2}{\partial B^2} \ln Z$$

Via in-house software[4], we have calculated the expansion up to order 13 for rings with 2 to 13 centers. Large J makes the expansion-based susceptibilities diverge at rather high temperatures. As increased nuclearity has the same effect, it was not possible to model rings with higher nuclearity in

the temperature region where experimental data were available. The calculated expressions for χ_m/N were fit to the experimental susceptibility per ion in the temperature region 250 – 370 K. g was fixed to 2.

1. Arthur, J. L.; Moore, C. E. ; Rheingold, A. L.; Miller, J.S. *Inorg. Chem.*, **2011**, *50* , 2735–2737.
2. Rushbrooke, G. S.; Wood, *P. J. Mol. Phys.*, **1958**, *1*, 257-283.
3. Eifert, T.; Hüning, F.; Lueken, H.; Schmidt, P.; Thiele, G. *Chem. Phys. Lett.*, **2002**, *364*, 69-74.
4. Thuesen, C. A.; Weihe, H.; Bendix, J.; Piligkos, S.; Mønsted, O. *Dalton Trans.* **2010**, *39*, 4882-4885.
5. Schmidt, H.-J.; Schnack, J.; Luban, M. *Phys. Rev. B*, **2001**, *64*, 224415.

PAPER 6

cyclo-Tetra-μ-fluorido-1:2κ^2*F*;2:3κ^2*F*;3:4κ^2*F*;1:4κ^2*F*-octanitrato-1κ^8*O*,*O*';3κ^8*O*,*O*'-tetrakis(1,10-phenanthroline)-2κ^4*N*,*N*';4κ^4*N*,*N*'-2,4-dichromium(III)-1,3-dineodymium(III) methanol tetrasolvate monohydrate

Torben Birk, Magnus Schau-Magnussen, Thomas Weyhermüller and Jesper Bendix

Acta Cryst., 2011, E67, m1561-m1562

Acta Crystallographica Section E
Structure Reports
Online
ISSN 1600-5368

cyclo-Tetra-*μ*-fluorido-1:2*κ*^{2}*F*;2:3*κ*^{2}*F*;3:4*κ*^{2}*F*;1:4*κ*^{2}*F*-octanitrato-1*κ*^{8}*O*,*O′*;3*κ*^{8}*O*,*O′*-tetrakis(1,10-phenanthroline)-2*κ*^{4}*N*,*N′*;4*κ*^{4}*N*,*N′*-2,4-dichromium(III)-1,3-dineodymium(III) methanol tetrasolvate monohydrate

Torben Birk,[a] Magnus Schau-Magnussen,[a] Thomas Weyhermüller[b] and Jesper Bendix[a]*

[a]Department of Chemistry, University of Copenhagen, Universitetsparken 5, DK-2100 Copenhagen, Denmark, and [b]MPI für Bioanorganische Chemie, Stiftstrasse 34–36, PO Box 101365, D-45413 Mülheim an der Ruhr, Germany
Correspondence e-mail: bendix@kiku.dk

Received 30 September 2011; accepted 13 October 2011

Key indicators: single-crystal X-ray study; T = 122 K; mean σ(C–C) = 0.003 Å; H-atom completeness 97%; R factor = 0.036; wR factor = 0.102; data-to-parameter ratio = 42.4.

In the title compound, $[Cr_2Nd_2F_4(NO_2)_8(C_{12}H_8N_2)_4]\cdot 4CH_3OH\cdot H_2O$, two *cis*-difluoridobis(1,10-phenanthroline)chromium(III) fragments containing octahedrally coordinated chromium(III) bridge *via* fluoride ions to two tetranitratoneodymate(III) fragments, forming an uncharged tetranuclear square-like core. The fluoride bridges are fairly linear, with Cr—F—Nd angles of 168.74 (8)°. Cr—F bond lengths are 1.8815 (15) Å, slightly elongated compared to those of the parent chromium(III) complex, which has bond lengths ranging from 1.8444 (10) to 1.8621 (10) Å. The tetranuclear complex is centered at a fourfold rotoinversion axis, with the Cr and Nd atoms situated on two perpendicular twofold rotation axes. The uncoordinated water molecule resides on a fourfold rotation axis. The four methanol solvent molecules are located around this axis, forming a cyclic hydrogen-bonded arrangement. The title compound is the first structurally characterized example of unsupported fluoride bridges between lanthanide and transition metal ions.

Related literature

For related structures of second sphere interactions with robust chromium(III) fluoride complexes, see: Birk *et al.* (2010); Terasaki *et al.* (1999); Kaizaki & Takemoto (1990). For other examples of fluoride bridges between 3*d* and 4*f* metal atoms, see: Pevec *et al.* (2003); McRobbie *et al.* (2011). For the structure of the cationic chromium precursor complex, see: Birk *et al.* (2008). For the synthesis of the precursor, see: Glerup *et al.* (1970). For importance of the title compound in the context of magnetic materials, see: Kahn (1985, 1987); Sessoli & Powell (2009). For crystallographic background, see: Coppens (1970).

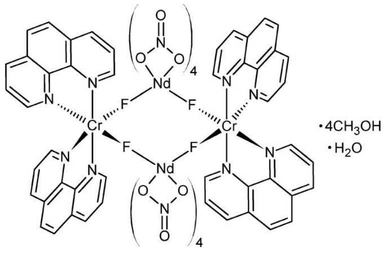

Experimental

Crystal data

$[Cr_2Nd_2F_4(NO_2)_8(C_{12}H_8N_2)_4]\cdot 4CH_4O\cdot H_2O$
M_r = 1831.56
Tetragonal, $P4/ncc$
a = 17.632 (4) Å
c = 20.955 (3) Å
V = 6515 (2) Å^3
Z = 4
Mo $K\alpha$ radiation
μ = 2.01 mm^{-1}
T = 122 K
0.35 × 0.29 × 0.24 mm

Data collection

Nonius KappaCCD area-detector diffractometer
Absorption correction: integration (Gaussian; Coppens, 1970)
T_{min} = 0.601, T_{max} = 0.718
339826 measured reflections
10126 independent reflections
6979 reflections with $I > 2\sigma(I)$
R_{int} = 0.047

Refinement

$R[F^2 > 2\sigma(F^2)]$ = 0.036
$wR(F^2)$ = 0.102
S = 1.27
10126 reflections
239 parameters
H-atom parameters constrained
$\Delta\rho_{max}$ = 2.41 e Å^{-3}
$\Delta\rho_{min}$ = −1.78 e Å^{-3}

Table 1
Hydrogen-bond geometry (Å, °).

D—H···A	D—H	H···A	D···A	D—H···A
O20—H20···O20^{i}	0.84	1.89	2.700 (4)	161

Symmetry code: (i) $y, -x+\frac{1}{2}, z$.

Data collection: *COLLECT* (Nonius, 1999); cell refinement: *COLLECT*; data reduction: *EVALCCD* (Duisenberg *et al.*, 2003); program(s) used to solve structure: *SHELXS97* (Sheldrick, 2008); program(s) used to refine structure: *SHELXL97* (Sheldrick, 2008); molecular graphics: *ORTEP-3* (Farrugia, 1997) and *Mercury* (Macrae *et al.*, 2006); software used to prepare material for publication: *SHELXL97*.

JB thanks the Danish Research Council (FNU) for financial support (grant No. 272–08–0491).

Supplementary data and figures for this paper are available from the IUCr electronic archives (Reference: WM2538).

electronic reprint

References

Birk, T., Bendix, J. & Weihe, H. (2008). *Acta Cryst.* E**64**, m369–m370.
Birk, T., Magnussen, M. J., Piligkos, S., Weihe, H., Holten, A. & Bendix, J. (2010). *J. Fluorine Chem.* **131**, 898–906.
Coppens, P. (1970). *Crystallographic Computing*, edited by F. R. Ahmed, S. R. Hall & C. P. Huber, pp. 255–270. Copenhagen: Munksgaard.
Duisenberg, A. J. M., Kroon-Batenburg, L. M. J. & Schreurs, A. M. M. (2003). *J. Appl. Cryst.* **36**, 220–229.
Farrugia, L. J. (1997). *J. Appl. Cryst.* **30**, 565.
Glerup, J., Josephsen, J., Michelsen, K., Pedersen, E. & Schäffer, C. E. (1970). *Acta Chem. Scand.* **24**, 247–254.
Kahn, O. (1985). *Angew. Chem. Int. Ed.* **24**, 834–850.
Kahn, O. (1987). *Struct. Bond.* **68**, 89–167.
Kaizaki, S. & Takemoto, H. (1990). *Inorg. Chem.* **29**, 4960–4964.
Macrae, C. F., Edgington, P. R., McCabe, P., Pidcock, E., Shields, G. P., Taylor, R., Towler, M. & van de Streek, J. (2006). *J. Appl. Cryst.* **39**, 453–457.
McRobbie, A., Sarwar, A. R., Yeninas, S., Nowell, H., Baker, M. L., Allan, D., Luban, M., Muryn, C. A., Pritchard, R. G., Prozorov, R., Timco, G. A., Tuna, F., Whitehead, G. F. & Winpenny, R. E. (2011). *Chem. Commun.* **47**, 6251–6253.
Nonius (1999). *COLLECT*. Nonius BV, Delft, The Netherlands.
Pevec, A., Mrak, M., Demsar, A., Petricek, S. & Roesky, H. W. (2003). *Polyhedron*, **22**, 575–579.
Sessoli, R. & Powell, A. K. (2009). *Coord. Chem. Rev.* **253**, 2328–2341.
Sheldrick, G. M. (2008). *Acta Cryst.* A**64**, 112–122.
Terasaki, Y., Fujihara, T., Schönherr, T. & Kaizaki, S. (1999). *Inorg. Chim. Acta*, **259**, 84–90.

electronic reprint

Acta Cryst. (2011). E**67**, m1561-m1562 [**doi:10.1107/S1600536811042383**]

cyclo-Tetra-μ-fluorido-1:2κ^2*F*;2:3κ^2*F*;3:4κ^2*F*;1:4κ^2*F*-octanitrato-1κ^8*O*,*O*';3κ^8*O*,*O*'-tetrakis(1,10-phenanthroline)-2κ^4*N*,*N*';4κ^4*N*,*N*'-2,4-dichromium(III)-1,3-dineodymium(III) methanol tetrasolvate monohydrate

T. Birk, M. Schau-Magnussen, T. Weyhermüller and J. Bendix

Comment

The magnetic properties of polynuclear, mixed lanthanoide transition metal complexes have received much attention (Sessoli & Powell, 2009). Since early suggestions by Kahn (1985, 1987) that exchange interactions involving *d*- and *f*-electrons were likely to lead to ferromagnetic coupling, due to vanishing orbital overlaps, many such systems have been synthesized and studied structurally and magnetically. Despite the high activity in this field, there are still simple types of bridging ligands, which have not been studied in this context. Thus, fluoride, which is known to bind strongly to lanthanoides has not been known as a bridging ligand between paramagnetic transition metal ions and lanthanoide ions until the very recent introduction of fluoride in heterometallic wheels by McRobbie *et al.* (2011). However, in those systems, fluoride bridges are always supported by carboxylate groups connecting the same metal ions. Based on those systems it is very difficult or impossible to make deductions concerning the geometric preferences of fluoride as a bridging ion and concerning magnetic exchange over fluoride bridges. This problem is remedied by a system such as the title compound, which is the first example of unsupported fluoride bridges between 3*d* and 4*f* metals.

In the title compound the solvate water molecule is located on a proper fourfold axis, whereas the tetranuclear $Cr_2Nd_2F_4$ fragment is centered on a fourfold rotoinversion axes. Consequently, all the metal ions are required to lie in the same plane perpendicular to the tetragonal axes (Fig. 1). The complexation of the neodymium atom induces a slight elongation of the Cr—F bonds by *ca* 0.03 Å in comparison with the parent compound (Birk *et al.*, 2008). The neodymium atom is 10-coordinated with its coordination sphere completed by bidentate nitrate ions coordinating with unexceptional bond lengths and bite angles. The uncoordinated water molecule is located on a fourfold axis and has no direct partner for hydrogen bonding (the next nearest atom is C5 in a distance of 3.816 (3) Å), which explains the high thermal displacement parameters for its oxygen atom. Around the same fourfold axis, the methanol solvate molecules form a cyclic tetrameric arrangement held together by hydrogen bonds (Table 1, Fig. 2).

Studies of the magnetic properties of this system and the possible generalization of this route to fluoride-bridged systems are currently being undertaken.

Related structures of second sphere interactions with robust chromium(III) fluoride complexes were presented by Birk *et al.* (2010); Terasaki *et al.* (1999); Kaizaki & Takemoto (1990). For other examples of fluoride bridges between 3*d* and 4*f* metal atoms, see: Pevec *et al.* (2003).

Experimental

trans-$[Cr(py)_4F_2]NO_3$ is synthesized by the literature method (Glerup *et al.*, 1970). 1,10-phenantroline (Alfa Aesar), $Nd(NO_3)_3{\cdot}6H_2O$ (Alfa Aesar; 99.9%), 2-methoxyethanol (Sigma-Aldrich; 99.3+%) and methanol (Lab-Scan; Anhydroscan)

electronic reprint

were all used as received. The synthesis of *cis*-[Cr(phen)$_2$F$_2$]NO$_3$ proceeds in many ways analogous to the method described by Glerup *et al.* (1970) for the synthesis of *cis*-[Cr(phen)$_2$F$_2$]ClO$_4$. As a result of a significant difference in solubility of the two salts, some modification with respect to solvent volume and isolation procedure has been introduced. It should also be noted that the nitrate can be crystallized with a variable number of crystal water and that this number can change depending on whether the substance is stored in dry or moist air. Elemental analysis for C, H and N was performed with an CE Instrument: FLASH 1112 series EA, at the microanalytic laboratory, University of Copenhagen. Electrospray (ES) mass spectra were recorded on a Micromass Q-TOF apparatus with positive ion detection.

i) Syntesis of the starting material *cis*-[Cr(phen)$_2$F$_2$]NO$_3$

trans-[Cr(py)$_4$F$_2$]NO$_3$ + 1,10-phenantroline = *cis*-[Cr(phen)$_2$F$_2$]NO$_3$ + 4py

trans-[Cr(py)$_4$F$_2$]NO$_3$ (36.7 g; 0.078 mol) and 1,10-phenantroline (34.8 g; 0.19 mol) were placed in a conical flask (500 ml) with 2-methxyethanol (250 ml). The mixture was heated to boiling temperature, whereby a violet solution formed, followed shortly by precipitation of a red-violet solid. The heating was continued for 1 h followed by cooling to room temperature before a purple red product was isolated. The raw product was washed with ethanol (2x100 ml) and dried by suction.

Yield of raw product: 31.7 g (79.0% of theoretical based on Cr^{III}). Analysis: Calcd. for H$_{17}$C$_{24}$N$_5$O$_4$F$_2$Cr$_1$: H, 3.29%; C, 55.28%; N, 13.43%. Found: H, 3.16%; C, 55.25%; N, 13.30% (sesqui hydrat). TOF MS ES$^+$ (MeOH): m/*z*: 450.5 ([Cr(phen)$_2$F$_2$]$^+$)

ii) Syntesis of the title compound {[Cr(phen)$_2$(μ-F)$_2$][Nd(NO$_3$)$_4$]}$_2$·CH$_3$OH·H$_2$O

2 *cis*-[Cr(phen)$_2$F$_2$]NO$_3$ + 2 Nd(NO$_3$)$_3$ = {[Cr(phen)$_2$(μ-F)$_2$][Nd(NO$_3$)$_4$]}$_2$

The title compound was prepared by reaction of a methanolic solution of *cis*-[Cr(phen)$_2$F$_2$](NO$_3$) (210 mg, 0.41 mmol in 10 ml) with a methanolic solution of Nd(NO$_3$)$_3$·6H$_2$O (175 mg, 0.40 mmol in 5 ml). Before combination, both solutions were filtered through filters with pore size 0.45 μm. Crystals formed over a period of 2–12 h. The yield was 284 mg (82% based on Nd). Crystals suitable for single-crystal X-ray diffraction were obtained directly using the concentrations given above. Upon drying, the crystals loose solvent and deteriorate. For the diffraction experiment, a crystal was taken from the mother liquor, covered with paraffin oil and cooled directly.

Refinement

H atoms were found in a difference Fourier map and were included in the refinement as constrained idealized protons riding the parent atom, with *X*—H = 0.84 Å (OH); 0.95 Å (aromatic CH); 0.98 Å (CH$_3$) with U_{iso} equal to 1.2×U_{eq} of the parent C atom (1.5×U_{eq} of the parent atom in MeOH). No resonable assignment of the H atoms of the water of crystallization could be obtained. Consequently, these H atoms were excluded from the refinement. The maximum residual electron density is found at 1.04 Å from O20, the minimum residual electron density is at 0.37 Å from the same atom.

electronic reprint

Figures

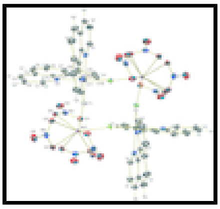

Fig. 1. A view of the tetranuclear molecular structure of the title compound with the atom-labelling scheme. Displacement ellipsoids are drawn at the 50% probability level. Solvent methanol and water molecules were omitted.

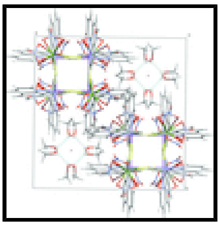

Fig. 2. A packing diagram in projection along [00$\bar{1}$] showing hydrogen bonds between the methanol solvent molecules (dotted lines).

cyclo-Tetra-μ-fluorido-1:2κ^2*F*;2:3κ^2*F*; 3:4κ^2*F*;1:4κ^2*F*-octanitrato-1κ^8*O,O'*; 3κ^8*O,O'*-tetrakis(1,10-phenanthroline)- 2κ^4*N,N'*;4κ^4*N,N'*-2,4-dichromium(III)- 1,3-dineodymium(III) methanol tetrasolvate monohydrate

Crystal data

$[Cr_2Nd_2F_4(NO_2)_8(C_{12}H_8N_2)_4]\cdot 4CH_4O\cdot H_2O$	D_x = 1.867 Mg m^{-3}
M_r = 1831.56	Mo *K*α radiation, λ = 0.71073 Å
Tetragonal, *P*4/*ncc*	Cell parameters from 120466 reflections
Hall symbol: -P 4a 2ac	θ = 2.3–40.1°
a = 17.632 (4) Å	μ = 2.01 mm^{-1}
c = 20.955 (3) Å	*T* = 122 K
V = 6515 (2) Å^3	Prism, pink
Z = 4	0.35 × 0.29 × 0.24 mm
F(000) = 3640	

Data collection

Nonius KappaCCD area-detector diffractometer	10126 independent reflections
Radiation source: fine-focus sealed tube	6979 reflections with $I > 2\sigma(I)$
graphite	R_{int} = 0.047
ω and φ scans	θ_{max} = 40.1°, θ_{min} = 2.3°
Absorption correction: integration (Gaussian; Coppens, 1970)	*h* = −31→31
T_{min} = 0.601, T_{max} = 0.718	*k* = −29→31
339826 measured reflections	*l* = −37→37

electronic reprint

Refinement

Refinement on F^2	Primary atom site location: structure-invariant direct methods
Least-squares matrix: full	Secondary atom site location: difference Fourier map
$R[F^2 > 2\sigma(F^2)] = 0.036$	Hydrogen site location: inferred from neighbouring sites
$wR(F^2) = 0.102$	H-atom parameters constrained
$S = 1.27$	$w = 1/[\sigma^2(F_o^2) + (0.0144P)^2 + 22.4316P]$ where $P = (F_o^2 + 2F_c^2)/3$
10126 reflections	$(\Delta/\sigma)_{max} = 0.002$
239 parameters	$\Delta\rho_{max} = 2.41$ e Å^{-3}
0 restraints	$\Delta\rho_{min} = -1.78$ e Å^{-3}

Special details

Geometry. All esds (except the esd in the dihedral angle between two l.s. planes) are estimated using the full covariance matrix. The cell esds are taken into account individually in the estimation of esds in distances, angles and torsion angles; correlations between esds in cell parameters are only used when they are defined by crystal symmetry. An approximate (isotropic) treatment of cell esds is used for estimating esds involving l.s. planes.

Refinement. Refinement of F^2 against ALL reflections. The weighted R-factor wR and goodness of fit S are based on F^2, conventional R-factors R are based on F, with F set to zero for negative F^2. The threshold expression of $F^2 > 2\sigma(F^2)$ is used only for calculating R-factors(gt) *etc*. and is not relevant to the choice of reflections for refinement. R-factors based on F^2 are statistically about twice as large as those based on F, and R-factors based on ALL data will be even larger.

Fractional atomic coordinates and isotropic or equivalent isotropic displacement parameters (Å^2)

	x	y	z	U_{iso}*/U_{eq}
Nd1	0.120550 (5)	0.879450 (5)	0.2500	0.01191 (3)
Cr1	0.142468 (17)	0.642468 (17)	0.2500	0.01199 (6)
F1	0.14142 (8)	0.74891 (8)	0.24362 (7)	0.0179 (2)
N1	0.14262 (10)	0.52607 (10)	0.24070 (8)	0.0148 (3)
N2	0.13452 (11)	0.63381 (11)	0.15243 (8)	0.0154 (3)
N3	0.05724 (12)	0.80814 (11)	0.36783 (10)	0.0194 (3)
N4	−0.00247 (12)	0.89135 (13)	0.15390 (10)	0.0200 (3)
O1	0.12382 (11)	0.83605 (11)	0.36641 (9)	0.0224 (3)
O2	0.01948 (10)	0.81071 (10)	0.31637 (8)	0.0194 (3)
O3	0.03125 (13)	0.77993 (12)	0.41658 (9)	0.0280 (4)
O4	0.02408 (11)	0.94903 (11)	0.18224 (10)	0.0237 (3)
O5	0.01769 (11)	0.82711 (11)	0.17550 (9)	0.0227 (3)
O6	−0.04596 (12)	0.89763 (14)	0.10853 (9)	0.0297 (4)
C1	0.15642 (14)	0.47330 (13)	0.28428 (11)	0.0186 (3)
H1	0.1678	0.4886	0.3267	0.022*
C2	0.15473 (15)	0.39539 (14)	0.26990 (12)	0.0222 (4)
H2	0.1663	0.3590	0.3019	0.027*
C3	0.13624 (15)	0.37223 (13)	0.20931 (12)	0.0217 (4)

electronic reprint

PAPER 7

Fluoride Bridges as Structure-Directing Motifs in 3d-4f Cluster Chemistry

Torben Birk, Kasper Steen Pedersen, Christian Aa. Thuesen, Thomas Weyhermüller, Magnus Schau-Magnussen, Stergios Piligkos, Høgni Weihe, Susanne Mossin,
Marco Evangelisti and Jesper Bendix

Inorg. Chem., **2012**, 51(9), 5435–5443.

Inorganic Chemistry
Article
pubs.acs.org/IC

Fluoride Bridges as Structure-Directing Motifs in 3d-4f Cluster Chemistry

Torben Birk,[†] Kasper S. Pedersen,[†] Christian Aa. Thuesen,[†] Thomas Weyhermüller,[‡] Magnus Schau-Magnussen,[†] Stergios Piligkos,[†] Högni Weihe,[†] Susanne Mossin,[§] Marco Evangelisti,[⊥] and Jesper Bendix*[,†]

[†]Department of Chemistry, University of Copenhagen, Universitetsparken 5, DK-2100 Copenhagen, Denmark

[‡]Max Planck Institute for Bioinorganic Chemistry, D-45470 Mülheim an der Ruhr, Germany

[§]Department of Chemistry, Technical University of Denmark, DK-2800 Kgs. Lyngby, Denmark

[⊥]Instituto de Ciencia de Materiales de Aragón, Departamento de Física de la Materia Condensada, CSIC-Universidad de Zaragoza, 50009 Zaragoza, Spain

Ⓢ *Supporting Information*

ABSTRACT: The use of kinetically robust chromium(III) fluorido complexes as synthons for mixed 3d-4f clusters is reported. The tendency toward linear $\{Cr^{III}{-}F{-}Ln^{III}\}$ units dictates the cluster topology. Specifically, we show that reaction of *cis*-$[Cr^{III}F_2(NN)_2]NO_3$ (NN = 1,10-phenanthroline ("phen") or 2,2′-bipyridine ("bpy")) with $Ln(NO_3)_3{\cdot}xH_2O$ produces isostructural series of molecular $\{Ln_2Cr_2\}$ squares (**1**−**9**) with linear fluoride bridges. In a parallel fashion, *fac*-$[Cr^{III}F_3L]$, where L = *N*,*N*′,*N*″-trimethyl-1,4,7-triazacyclononane ("Me_3tacn"), reacts with $Nd(NO_3)_3{\cdot}6H_2O$ to form a fluoride-centered penta-nuclear complex and *fac*-$[Cr^{III}F_3L']$, with L′ = 1,1,1-tris-((methylamino)methylethane) ("Me_3tame"), reacts with $[Ln(hfac)_3(H_2O)_2]$ (hfacH = 1,1,1,5,5,5-hexafluoroacetylacetone) to yield an isostructural series of $\{Ln_3Cr_2\}$ (**10**−**14**) trigonal bipyramids with no central ligand. The formation of the latter is accompanied by a partial solvolysis of the Cr(III) precursor but without formation of insoluble LnF_3. The magnetic properties of the gadolinium containing clusters allow quantification of fluoride-mediated, antiferromagnetic Gd−Cr exchange interactions of magnitude between 0.14 cm^{-1} and 0.71 cm^{-1} ($\hat{H} = J_{12}\hat{\mathbf{S}}_1{\cdot}\hat{\mathbf{S}}_2$ formalism) and vanishingly small J_{Gd-Gd} of 0.06(0) cm^{-1}. The large spin and small anisotropy together with weak exchange interactions in the $\{Gd_3Cr_2\}$ (**11**) cluster give rise to a very large magneto-caloric effect of $-\Delta S_m$ = 28.7 J kg^{-1} K^{-1} ($\mu_0 H$ = 90 to 0 kOe).

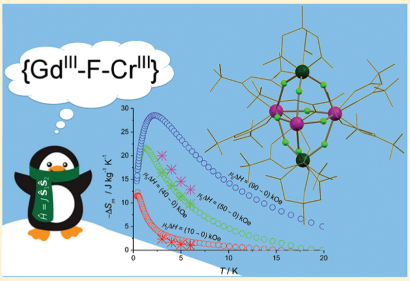

■ INTRODUCTION

Interest in high-nuclearity clusters incorporating lanthanoid ions has been boosted by the quest for magnetically anisotropic molecular systems as single-molecule magnets,[1] and, more recently, the increasing focus on nanoscopic coolers[2] has established a need for molecular entities exhibiting very-large spin ground states. Presently, the vast majority of mixed 3d-4f clusters are bridged by large organic ligands or smaller entities such as hydroxide or especially alkoxides whereas valence-isoelectronic fluoride bridges are exceedingly scarce.[3] Reported examples of fluoride-bridging included, until recently, only systems with diamagnetic Ti(IV) ions, namely, $[La\{(C_5Me_4Et)_2Ti_2F_7\}_3]$[4] and $[Ln\{(C_5Me_5)_2Ti_2F_7\}_3]$ (Ln = Pr, Nd)[5] in which the 12-coordinate lanthanoid ion is exclusively surrounded by fluoride ions. Recently,[6] fluoride-bridged systems were augmented by lanthanoid-containing wheels or fused wheels featuring fluoride bridges in conjunction with pivalate bridges between chromium(III) and lanthanoids. As discussed by Winpenny and co-workers, the synthesis of fluoride-bridged 3d-4f clusters is hampered by the strong affinity of lanthanoid(III) ions for fluoride resulting in competitive formation of highly insoluble LnF_3. The above-mentioned titanium complexes probably owe their existence to the comparable affinity of "hard"[7] Ti(IV) and the lanthanide ion for fluoride. Thus, the successful isolation of those polynuclear systems is most likely irrelevant for the majority of other transition metal ions. However, relying on kinetics rather than thermodynamics the use of kinetically robust transition metal (TM) fluoride complexes, for example, of chromium(III), may be a generally feasible approach provided the synthesis can be carried out relatively fast and at moderate temperatures. In the synthesis of mixed 3d-4f clusters topological control is difficult to achieve because of the flexibility in coordination number and geometry of the lanthanoid ions and frequent solvent coordination. There are

Received: February 24, 2012
Published: April 12, 2012

 © 2012 American Chemical Society dx.doi.org/10.1021/ic300421x | *Inorg. Chem.* 2012, 51, 5435−5443

very few accessible mono- or diatomic bridges which have not been extensively explored. Its simplicity, spectroscopic innocence, relatively low basicity, and preference for "hard" metal ions make fluoride an attractive bridging ligand for mixed transition metal-lanthanoid complexes. Furthermore, the tendency toward pseudolinear bridging established in transition metal chemistry could facilitate prediction and design of specific molecular topologies, if it can be shown to carry over into lanthanoid chemistry. To exploit the latter property of the fluoride ligand, it is necessary to target simple systems with unsupported fluoride bridges. This will also allow for a quantification of the magnetic interaction via the fluoride bridge, which was not possible in the wheels wherein the fluoride bridge coexists with one or two pivalate bridges.

Useful building blocks for polynuclear fluoride-bridged 3d-4f systems would be robust di- or trifluorido complexes of Cr(III) preferably with polydentate coligands and soluble in organic solvents. Such difluorido precursors are well described in literature with the auxiliary ligand sphere consisting of amines or imines, for example, *cis*-$[CrF_2(phen)_2]NO_3$.[8] Additionally, neutral trifluorido analogues can be obtained by minor modifications of published synthetic procedures.[9] Here we describe the syntheses and properties of a class of simple chromium(III)-lanthanide clusters starting from di- and trifluorido complexes of chromium: *cis*-$[CrF_2(NN)_2]^+$ (NN = phen, bpy), *fac*-$[CrF_3(Me_3tame)]$, and *fac*-$[CrF_3(Me_3tacn)]$.

■ RESULTS AND DISCUSSION

We have recently, demonstrated the formation of bimetallic fluoride bridged linear rods or squares obtained by using robust *trans*-$[CrF_2(py)_4]^+$ (py = pyridine)[10] or *cis*-$[CrF_2(phen)_2]^+$,[11] respectively, as building blocks. This type of reactivity is generalizable, and 1:1 assembly reactions in methanolic solution of *cis*-$[CrF_2(phen)_2]NO_3$ and $Ln(NO_3)_3{\cdot}aq$ yield tetranuclear clusters, which crystallize as solvates with the general formula $[\{CrF_2(phen)_2\}_2\{Ln(NO_3)_4\}_2]{\cdot}4MeOH{\cdot}H_2O$ (Ln = Ce−Nd, Sm−Ho (**1**−**9**)). Yields diminish pronouncedly with increasing atomic number of the lanthanide and no product is obtained at all for the heaviest ones (Er−Yb). The decreasing yield with increasing atomic number is accompanied by an increasing amount of an easily separable byproduct, which is not LnF_3, as determined by analysis and powder diffraction. The exact nature of the byproduct has however not been determined. Freshly precipitated, and not too intensely dried, the tetranuclear compounds are isomorphous and crystallize in the tetragonal space group *P*4/*ncc* as demonstrated by the powder diffraction data for the series Ce−Dy (except *Pm*) in Figure 1. Upon thorough drying the crystal solvents are lost, as witnessed by elemental analyses and deterioration of the crystals.

The *cis*-coordination of the two fluoride ions imposes a square structure on the resulting tetranuclear cluster with almost linear (169°) Ln−F−Cr bridges (cf. Figure 2). The lanthanide ion is deca-coordinated with four bidentate nitrate ligands and two bridging fluoride ligands. The coordination number is smaller than usual in bidentate nitrate complexes where coordination numbers of 11 and 12 are common, but it is also found in, for example, $[Nd(NO_3)_4(CH_3OH)_2]^-$.[12] The coordination around chromium is very similar to that in the parent complex, but with slightly elongated Cr−F bond lengths 1.8816(14)/1.8844(17) Å as compared to 1.8444(10)−1.8621(10) Å in $[CrF_2(phen)_2](ClO_4){\cdot}H_2O$.[13]

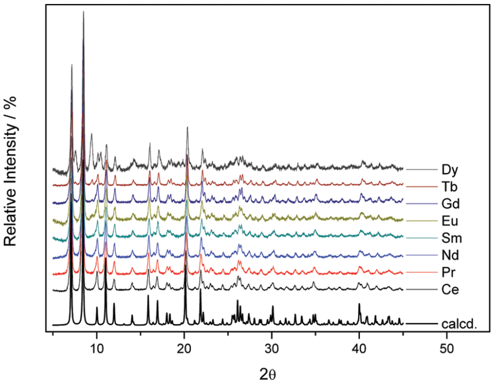

Figure 1. Powder diffraction patterns for $[\{CrF_2(phen)_2\}_2\{Ln(NO_3)_4\}_2]{\cdot}4MeOH{\cdot}H_2O$ (**1**−**8**). The lower trace is calculated on basis of the single crystal diffraction data for the gadolinium compound. The yields diminish steeply with increasing atomic weight of the lanthanoid and amounts to less than 2% for the Dy-compound (cf. Experimental Section). The low-angle part of the powder diffraction also demonstrates the Dy-compound to contain a crystalline impurity.

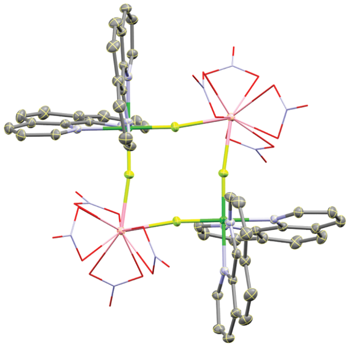

Figure 2. X-ray molecular structure of the isostructural metal clusters in **6** and **3**, for comparison shown with thermal ellipsoids (50% probability). Hydrogens and solvent molecules are omitted and nitrate ligands have been shown as wireframes for clarity. Color code: pink, Ln; green, Cr; yellow, F; red, O; blue, N; gray, C. Selected bond lengths (Å) and angles (deg), for **6**: Cr−F: 1.8844(17); Cr−N: 2.058(2)−2.063(2); Gd−F: 2.2844(16); Gd−O: 2.506(2)−2.533(2); Cr−F−Gd: 168.61(9). For **3**: Cr−F: 1.8816(14); Cr−N: 2.0550(17)−2.0615(19); Nd−F: 2.3348(15); Nd−O: 2.5326(19)−2.5651(18); Cr−F−Nd: 168.74(8).

The Cr−N distances are at 2.0550(17)−2.063(2) Å in the same range as found in the monomeric precursor: 2.0566(15)−2.0934(15) Å. The crystallographic symmetry, which connects different clusters by 4-fold screw-axes ensures all clusters to be oriented with parallel Cr_2Ln_2-planes in the packing (see the Supporting Information).

The closely related *cis*-$[CrF_2(bpy)_2]^+$ reacts analogously and furnishes structurally similar fluoride-bridged tetranuclear clusters. However, these systems are void of solvents of crystallization and crystallize with lower symmetry in orthorhombic *I*222 (cf. Supporting Information). It is note-

worthy, that the *cis* configuration of the fluoride ligands in $[CrF_2(phen)_2]^+$ does not suffice to introduce chelate binding toward the lanthanoid ions. A similar result has recently been observed for a homometallic tetranuclear manganese(II) cluster: $[Mn^{II}_4F_4(phen)_8](NO_3)_4\cdot 12H_2O$[14] and parallels the tetra-nuclear structures with approximately linear fluoride bridges found for many metal pentafluorides. Thus, while many examples are known with doubly bridging hydroxide, oxide, and the heavier halides, fluoride clearly disfavors this structural motif. This conclusion becomes even more evident when the products resulting from reaction between trifluorido complexes of Cr(III) and lanthanoid complexes are considered. Scheme 1 depicts the structures obtained with the di- and

Scheme 1. Structure Diagrams of the Polynuclear Complexes Obtained from Di- and Trifluorido Chromium(III) Building Blocks[a]

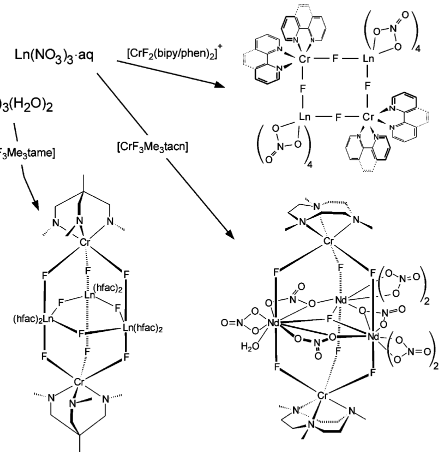

[a]The complete absence of chelating binding towards the lanthanoid centers illustrates the tendency of fluoride bridges to avoid bending.

trifluorido complexes. Thus, for both *fac*-$[CrF_3(Me_3tame)]$ and *fac*-$[CrF_3(Me_3tacn)]$, pentanuclear structures were obtained from acetonitrile solutions using respectively $[Ln(hfac)_3(H_2O)_2]$ (Ln = Sm (**10**), Gd (**11**), Ho (**12**), Yb (**13**); hfacH = 1,1,1,5,5,5-hexafluoroacetylacetone) and $Nd(NO_3)_3\cdot 6H_2O$ as lanthanoid sources. Both types of structures consist of triangular arrangements of the lanthanoid ions bridged in the equatorial plane by either bidentate and bridging nitrate (μ-nitrato-1,2κO:1$\kappa O'$) ligands or fluoride ligands. These triangles are then capped above and below the plane by tridentate, but nonchelating, $\{LCrF_3\}$ moieties. Additionally, the system derived from *fac*-$[CrF_3(Me_3tacn)]$ features a most unusual central μ_3-fluorido ligand bridging all three neodymium centers symmetrically. This bridging mode is very rare, but not completely unprecedented in transition metal chemistry where examples of planar μ_3-F bridging fluoride exist in, for example, $[Ni_3(\mu_3\text{-}F)(CF_3CO_2)_6(CF_3CO_2H)_3]^-$.[15] The overall 3-fold symmetry of the clusters is broken by different equatorial coordination environments of the lanthanoids and associated small variations in Ln−F bond lengths toward the $\{CrLF_3\}$ moieties.

Crystal structures of the pentanuclear systems are shown in Figure 3. An important feature of the structures is the

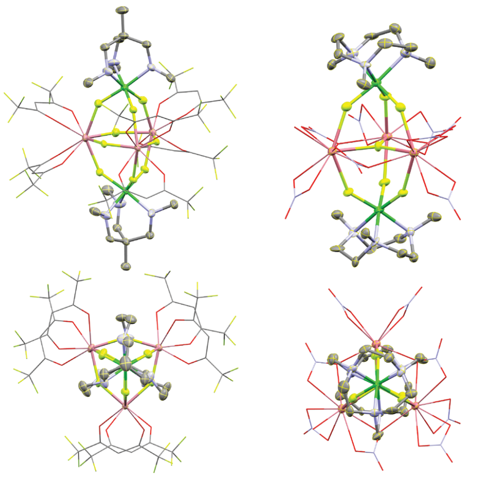

11,12 **14**

Figure 3. Side and top views of crystal structures of $[\{CrF_3(Me_3tame)\}_2Ln_3(hfac)_6(\mu\text{-}F)_3]\cdot 7CH_3CN$ (Ln = Gd (**11**), Ho (**12**); left) and $[\{CrF_3(Me_3tacn)\}_2Nd_3(NO_3)_8(\mu_3\text{-}F)(H_2O)]\cdot 6CH_3CN$ (**14**; right). Solvent molecules and hydrogens have been omitted for clarity. Thermal ellipsoids (50% probability) have only been given for the inner cores, peripheral ligands have been shown as wireframes for clarity. Color code: pink, Ln; green, Cr; yellow, F; red, O; blue, N; gray, C. Selected bond lengths (Å) and angles (deg), **11**: Cr1−F: 1.903(7)−1.915(7), Cr2−F: 1.903(6)−1.912(7), Cr1−N: 2.043(12)−2.053(12), Cr2−N: 2.057(11)−2.066(12), Gd−$F_{\mu\text{-Cr}}$: 2.295(6)−2.338(7), Gd−$F_{\mu\text{-Gd}}$: 2.216(6)−2.244(7), Gd−O: 2.39(3)−2.49(2), Cr−F−Gd: 142.8(3)−146.6(3), Gd−F−Gd: 142.3(3)−143.9(4),**12**: Cr1−F: 1.893(4)−1.904(4), Cr2−F: 1.892(4)−1.915(4), Cr1−N: 2.055(8)−2.062(7), Cr2−N: 2.044(7)−2.055(7), Ho−$F_{\mu\text{-Cr}}$: 2.256(4)−2.311(4), Ho−$F_{\mu\text{-Ho}}$: 2.189(4)−2.200(4), Ho−O: 2.35(3)−2.463(19), Cr−F−Ho: 143.3(2)−145.5(2), Ho−F−Ho: 143.3(2)−146.9(2),**14**: Cr1−F: 1.894(8)−1.936(8), Cr2−F: 1.918(9)−1.938(8), Cr1−N: 2.040(12)−2.095(12), Cr2−N: 2.075(12)−2.094(13), Nd1−F120(μ_3): 2.365(8), Nd2−F120(μ_3): 2.434(8), Nd3−F120(μ_3): 2.410(7), Nd1−O: 2.489(11)−2.542(10), Nd2−O: 2.508(11)−2.652(11), Nd3−O: 2.499(10)−2.626(10), Nd1−$F_{\mu\text{-Cr}}$: 2.297(8)−2.326(8), Nd2−$F_{\mu\text{-Cr}}$: 2.342(8)−2.353(8), Nd3−$F_{\mu\text{-Cr}}$: 2.372(8)−2.386(8). Cr−F−Nd: 138.5(4)−146.8(4), Nd−O−Nd: 109.4(4)−110.0(4).

additional bridging fluoride ligands (μ_3 or μ_2), which demonstrates that even quite robust Cr(III) fluoride complexes are susceptible to fluoride abstraction by lanthanoid ions under mild conditions (cf. Experimental Section). However, despite of this, formation of very insoluble LnF_3 was never observed under the experimental conditions used to prepare these polynuclear complexes. Although the Cr−F−Ln bridges in **11**, **12**, and **14** are significantly bent at angles of 138.6−146.8°, it is striking that the facial trifluorido complexes avoid chelate binding of a single lanthanoid ion despite the lability and size of the lanthanoid ions. This is again paralleled by the transition metal fluoride chemistry where tri-μ_2-fluorido complexes are scarce in comparison with the abundance of tri-μ_2-hydroxido

and tri-μ_2-chlorido complexes. In combination these observations attest to a pronounced preference for linear bridging by fluoride, which pertains to systems with lanthanoid ions. This geometric preference is reminiscent of the behavior of cyanide as a bridging ligand and it might, therefore, render robust fluoride complexes, appealing building blocks for assembly of polynuclear systems with targeted topologies.

In the structures of **11** and **12**, Cr–F–Ln and Ln–F–Ln bridging angles are very similar and both close to 140°. However, this similarity should not be overinterpreted since the trigonal bipyramidal structures in combination with the metal-fluoride bond lengths restrict the bridging angles, which are thus compromises between linear bridging and optimal bond lengths. Since the preparations invariably involve hydrated starting materials, it could be speculated, that some of the fluorides alternatively could be assigned as isoelectronic hydroxide ligands. The X-ray structures of **11** and **12** were both solved with hydroxide in place of the equatorial fluoride bridges with very little effect on the goodness of the modeling, which only slightly, but not statistically significantly favors fluoride bridges. However, electrospray mass spectrometry (vide infra) only reveals intact clusters and fragments with fluoride bridges and thus rules out the presence of hydroxide bridges.

The polynuclear solid state structures described above are not merely consequences of crystal packing, but reflect association also in solution. When methanolic solutions of *cis*-$[CrF_2(phen)_2]NO_3$ and $Gd(NO_3)$·aq are mixed to yield solutions with different ratios of Gd/Cr (ranging from 0 to 2), there is an associated color change from red-purple to orange. The ligand-field (LF) spectrum of Cr(III) changes, resulting in a blue shift of the first spin-allowed band, $^4T_2(O)\leftarrow{}^4A_2(O)$, of 460 cm^{-1} (cf. Figure 4). Since the position

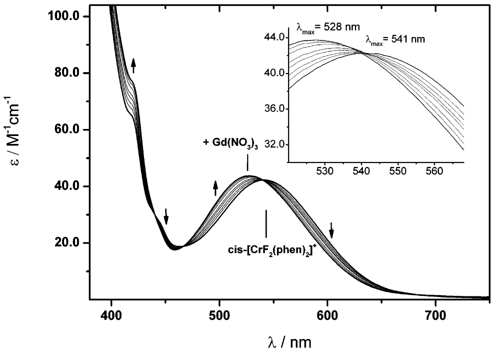

Figure 4. UV–vis spectral changes upon addition of $Gd(NO_3)_3$ to *cis*-$[CrF_2(phen)_2]NO_3$ in methanol/acetonitrile (1:4). Spectra were recorded over 30 min at room temperature (RT). Prolonged storage or further addition of $Gd(NO_3)_3$ results in precipitation of **6**.

of this band directly measures the octahedral component of the ligand-field, the direction of the shift toward higher energies might seem counterintuitive. However, shifts of the same sign and even larger magnitude were found for *fac*-$[CrF_3(Me_3tacn)]$ interacting with Na^+ ions in 2-propanol.[9] It has also generally been found that Cr(III) fluoride complexes exhibit solvatochromism with hypsochromic shifts upon moving to more strongly proton donating solvents.[16] It has been suggested that this effect stems from a different radial dependence of the LF σ- and π-parameters for fluoride. However, DFT modeling of the interaction between neutral trifluorido complexes of Cr(III) and Na^+ in vacuum as well as in solution revealed a pronounced breakdown of the commonly assumed additivity of ligand-field contributions.[9] Thus the actual decrease of the donor strength of fluoride upon ligation or solvation is more than compensated by an increased donation from the auxiliary amine, or as demonstrated here, imine ligands. The change in absorption spectrum results in isosbestic points suggesting only two spectroscopically distinct coordination environments of the chromium(III) as the Gd/Cr-ratio is increased.

The nature of the species in solution can be gauged by electrospray mass spectrometry of reaction mixtures before precipitation. For the tetranuclear compounds, this technique identifies fragments of composition $[\{CrF_2(phen)_2\}_2Ln(NO_3)_3Ln(NO_3)_4]^+$ and $[CrF_2(phen)_2Ln(NO_3)_3]^+$ as the most prominent peaks in positive ion detection mode (Supporting Information, Figure S5.1) and $[CrF_2(phen)_2Ln(NO_3)_5]^-$ in negative ion detection mode for Ln = Ce (**1**), Sm (**4**), and Gd (**6**). These ions are all likely fragments of the tetranuclear structures determined in the solid state and in agreement with the UV–vis spectroscopy points to extensive association in solution before precipitation.

Although very insoluble in acetonitrile, the isolated pentanuclear complexes (**10**–**13**) can be redissolved to a small extent in this solvent, and the resulting solutions yield mass spectra, which can unambiguously be correlated to the solid state structure. Thus, three peaks are prominent in positive ion detected electrospray mass spectra of such solutions of **10**–**13** (cf. Supporting Information, Figure S6.1–S6.4). These peaks correspond to compositions of $\{M\text{-hfac}\}^+$, $\{M+Na\}^+$, and $\{M\text{-}LnF_2(hfac)_2\}^+$, where M denotes the neutral pentanuclear cluster. The isotope pattern of the $\{M\text{-hfac}\}^+$-peak for **12** is shown in Figure 5 together with that of the intact cluster plus a sodium ion. For both entities simulations corresponding to all bridging ligands being fluoride have been included. The perfect reproduction of the isotope pattern (as found for the isostructural clusters with different lanthanoid ions: **10**, **12**, and **13**) rules out hydroxide ligands and demonstrate exclusive fluoride bridging in these clusters.

As the first examples of unsupported fluoride bridges between 3d and 4f ions, the magnetic properties of these systems are of interest. The temperature dependencies of the χT product for some of the tetranuclear square structures are shown in Figure 6. At high-temperature (300 K) the χT values (**2**: 6.3 cm^3 K mol^{-1}; **3**: 6.2 cm^3 K mol^{-1}; **4**: 4.0 cm^3 K mol^{-1}) are in agreement with the values expected for two chromium(III) ($S = 3/2$, $g \approx 2$) and two lanthanoid ions (Pr: $^3H_4$, $g_J = 4/5$; Nd: $^4I_{9/2}$, $g_J = 8/11$; Sm: $^6H_{5/2}$, $g_J = 2/7$). With decreasing temperature the value for χT drops slightly for all compounds because of the progressive depopulations of ligand-field states (Stark sublevels).

Furthermore, for **2** a steep drop occurs below ~10 K which is not observed for **3** and **4** where the χT goes to a local minimum (at ~13 K) and then increases rapidly on lowering temperature. For TM compounds this behavior is a signature of a ferrimagnetic spin arrangement; however, in this case it may be a result of weak ferromagnetic Ln–Cr (Ln = Nd, Sm) interactions masked by the above-mentioned decrease of χT on descending temperature. Notably, this behavior parallels that observed for oxalate-bridged Cr–Ln systems with the same lanthanoid ions.[17] The magnetization curves do not show any

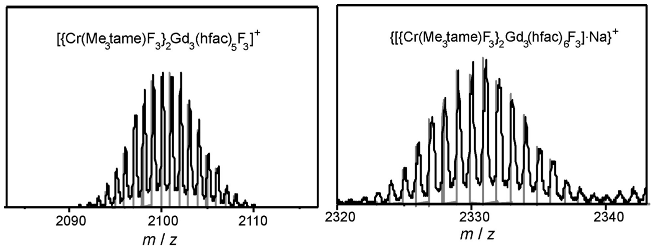

Figure 5. Extracts of the positive ion detected electrospray mass spectrum of **11** (black) together with simulations thereof (gray). Other important fragments observed for these complexes of all lanthanoid ions are $[\{Cr(Me_3tame)F_3\}_2Ln_2(hfac)_4F]^+$ and $[\{Cr(Me_3tame)F_3\}_2Ln_2(hfac)_5]^+$ (see Supporting Information).

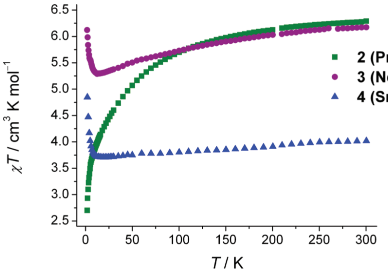

Figure 6. Temperature dependence of the χT product for **2**, **3**, and **4**. The expected high-temperature values calculated from the free ion Curie constants are **2**: 6.88 cm^3 K mol^{-1}, **3**: 6.95 cm^3 K mol^{-1}, **4**: 3.86 cm^3 K mol^{-1}.

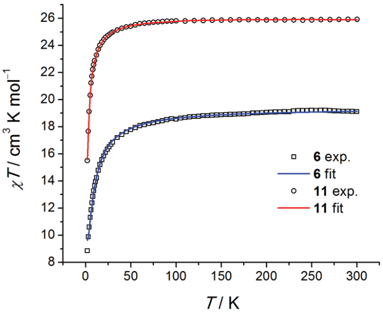

Figure 7. Fits of the χT product for **6** (Cr_2Gd_2-square) and **11** (Cr_2Gd_3-bipyramid) to the spin-Hamiltonian of eq 1. The resulting parameter values are **6**, J_{Cr-Gd} = 0.71 cm^{-1}; **11**, J_{Cr-Gd}= 0.14(0) cm^{-1}, J_{Gd-Gd}= 0.06(0) cm^{-1}. The g factors were fixed to $g_{Cr} = g_{Gd} = 2.0$.

signs of saturation at the highest available fields $H_{dc} \leq 5$ T (Supporting Information). This behavior is expected for systems with low-lying excited states and/or strong magnetic anisotropy. However, alternating current (ac) susceptibility measurements were conducted with and without a static field component and did not show any χ'' signal ruling out the possibility that any of the compounds behave as single-molecule magnets. This applies also to the dysprosium compound (**8**). Theoretical modeling of the magnetic properties of lanthanoid-containing clusters is inherently complicated because of the unknown ligand-field splittings and the exchange interactions which may be anisotropic in nature. For this reason, Gd^{3+}, with its orbitally nondegenerate $^8S_{7/2}$ ground state is the preferred lanthanoid ion for modeling of magnetic properties. In Figure 7 are shown the χT products for the tetranuclear (**6**) and pentanuclear (**11**) gadolinium systems together with the modeling of these data by the spin-Hamiltonians of eq 1:

For Cr_2Gd_2 (**6**):

$$\hat{H} = \mu_B \mathbf{B}\cdot[g_{Cr}(\hat{\mathbf{S}}_{Cr1} + \hat{\mathbf{S}}_{Cr2}) + g_{Gd}(\hat{\mathbf{S}}_{Gd1} + \hat{\mathbf{S}}_{Gd2})] + J_{Gd-Cr}(\hat{\mathbf{S}}_{Cr1}\cdot\hat{\mathbf{S}}_{Gd1} + \hat{\mathbf{S}}_{Cd1}\cdot\hat{\mathbf{S}}_{Cr2} + \hat{\mathbf{S}}_{Cr2}\cdot\hat{\mathbf{S}}_{Gd2} + \hat{\mathbf{S}}_{Gd2}\cdot\hat{\mathbf{S}}_{Cr1})$$

For Cr_2Gd_3 (**11**):

$$\hat{H} = \mu_B \mathbf{B}\cdot[g_{Cr}(\hat{\mathbf{S}}_{Cr1} + \hat{\mathbf{S}}_{Cr2}) + g_{Gd}(\hat{\mathbf{S}}_{Gd1} + \hat{\mathbf{S}}_{Gd2} + \hat{\mathbf{S}}_{Gd3})] + J_{Cr-Gd}(\hat{\mathbf{S}}_{Cr1} + \hat{\mathbf{S}}_{Cr2})\cdot(\hat{\mathbf{S}}_{Gd1} + \hat{\mathbf{S}}_{Gd2} + \hat{\mathbf{S}}_{Gd3}) + J_{Gd-Gd}(\hat{\mathbf{S}}_{Gd1}\cdot\hat{\mathbf{S}}_{Gd2} + \hat{\mathbf{S}}_{Gd1}\cdot\hat{\mathbf{S}}_{Gd3} + \hat{\mathbf{S}}_{Gd2}\cdot\hat{\mathbf{S}}_{Gd3}) \quad (1)$$

The g factors were fixed to $g_{Cr} = g_{Gd} = 2.0$. The χT and reduced magnetization (M vs $\mu_B B/k_B T$) data (Supporting Information) were fitted simultaneously. No zero-field splitting (ZFS) terms were included in the models but fitting of the χT data only, gave the same J parameter values indicating that the Cr(III) and Gd(III) magnetic anisotropies are vanishing. The fitting yields J_{Gd-Cr} = 0.71 cm^{-1} for **6** and J_{Gd-Cr} = 0.14(0) cm^{-1} and J_{Gd-Gd} = 0.06(0) cm^{-1} for **11**.[18] The magnetic exchange coupling across the fluoride bridges is found to be comparable in magnitude (0.14–0.71 cm^{-1}) to that observed for 3d-Gd interactions with other small bridges as cyanide[19] or oxalate.[20] To the best of our knowledge, only one example of a Gd(III)–Cr(III) cluster with solely monatomic bridges is reported.[21] In this hydroxide bridged $\{GdCr_4\}$ cluster the Gd–Cr interaction is indeed antiferromagnetic, but its magnitude was not extracted. The very pronounced difference in magnitude of the coupling in **6** and **11** in conjunction with other results obtained by us, suggests that coupling decreases steeply with bending of the fluoride bridges.[22]

The quite weak couplings in **11** together with isotropic Gd(III) and Cr(III) with holohedrized octahedral coordination

dx.doi.org/10.1021/ic300421x | Inorg. Chem. 2012, 51, 5435–5443

environment and concomitant small ZFS suggest that these systems should possess a large number of electronic states which are nearly degenerate with the ground state. This situation makes **11** a good candidate for the observation of a large Magneto-Caloric Effect (MCE), that is, the change of the magnetic entropy and related adiabatic temperature following a change in the applied magnetic field. This effect is at the basis of the magnetic refrigeration technique and therefore a large MCE is industrially in demand.

The magnetocaloric properties of **11** have been investigated by means of heat capacity (C) experiments, which represent the most suitable probe for the indirect determination of MCE.[2a] In Figure 8 is shown the experimental C, normalized to the gas

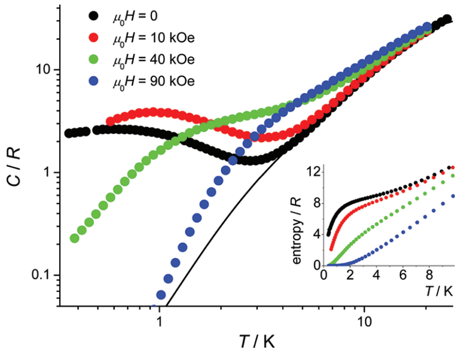

Figure 8. Temperature dependencies of the heat capacity (C) of **11** normalized to the gas constant R, collected for μ_0H = 0, 10, 40, and 90 kOe. Inset: temperature dependencies of the experimental entropy for several H, as obtained from the respective heat capacity data.

constant R, as function of temperature for selected applied magnetic fields (H). At high temperatures the heat capacity is dominated by nonmagnetic contributions arising from thermal vibrations of the lattice, which can be modeled with the Debye function (solid line in Figure 8) yielding a value of Φ_D = 18.0 K for the Debye temperature, which is in the range of values observed for this class of molecular compounds.[23] At low temperatures the heat capacity is dominated by an applied-field sensitive contribution, which shifts to higher temperatures by increasing H. From the experimental heat capacity, the temperature dependence of the entropy is obtained by integrating $\int C/T\, dT$, leading to the temperature dependencies of the entropy which are depicted in the inset of Figure 8 for the corresponding applied fields. In the cases of μ_0H = 0 and 10 kOe, the lack of data points below approximately 0.3 K forced us to add a constant value to the corresponding entropy curves to match the limiting values at high temperature. This procedure is justified by the entropy calculations performed on the C data obtained for higher fields, whose temperature dependencies are well within our experimentally accessible temperature window. As we shall see below, this procedure does not jeopardize our evaluation of the MCE of **11**. We also notice that the so-obtained zero-field entropy increases sharply reaching an approximate value of 8R at low temperatures. The 1 K < T < 5 K temperature range is characterized by a slow increase of the zero-field entropy, passing from ~8R to ~9R, respectively. Above roughly 5 K, the zero-field entropy starts again to steadily increase because of the dominant lattice contribution (see Figure 8). The 8−9 R plateau could be understood assuming that, for this temperature range, all magnetic interactions are decoupled to large extent. Therefore, under this assumption, we expect the entropy to approach the maximum value for noninteracting single-ion spins, that is, $3 \times R\ln(2S_{Gd} + 1) + 2 \times R\ln(2S_{Cr} + 1) = 9R$, where $S_{Gd} = 7/2$ and $S_{Cr} = 3/2$, in good agreement with the experimental data.

From the temperature and field dependencies of the entropy (Figure 8, inset), we can easily obtain the magnetic entropy change (ΔS_m) for selected applied field changes (ΔH). Note that the estimation of the lattice contribution is irrelevant for our calculations, since we deal with differences between total entropies at different magnetic field strengths. The resulting magnetic entropy changes are summarized in Figure 9. Besides

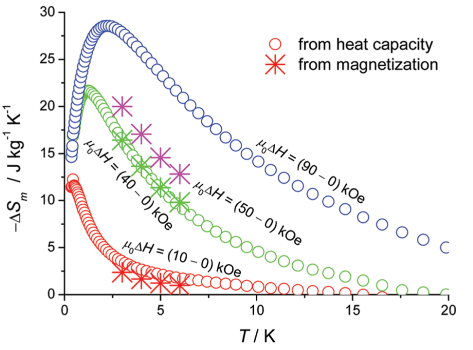

Figure 9. Temperature dependencies of the magnetic entropy change (ΔS_m) for **11**, as obtained from the respective heat capacity (○) and magnetization (*) data, for selected field changes (ΔH), as labeled.

heat capacity, magnetization (M) data can also be employed for estimating the magnetic entropy change by making use of the Maxwell relation, $\Delta S_m(T) = \int [\partial M/\partial T]\, dH$. From the isothermal $M(H)$ curves (Supporting Information, Figure S3.4) the so-obtained temperature-dependencies of ΔS_m for $\mu_0\Delta H$ = 10, 40, and 50 kOe are displayed in Figure 9. The nice agreement with the data obtained from C, proves that our experimental uncertainty in the low-temperature zero-field entropy does not affect the evaluation of the MCE of **11**.

We experimentally observe $-\Delta S_m$ to reach the maximum value of 28.7 J kg^{-1} K^{-1} for T = 2.2 K and ΔH = (90−0) kOe (Figure 9). This is a remarkable MCE that sets **11** among the best performing molecular magnetic refrigerants recently reported in the literature.[2,24] Our results suggest that **11** could potentially be employed as a magnetic refrigerant for the low-temperature range starting from 2 K and downward, that is, the starting temperature for an adiabatic demagnetization process.[2a] This range is of considerable technological interest because it is easily reachable by pumping liquid ^{4}He.

CONCLUSION

In conclusion, assembly of polynuclear lanthanoid complexes employing robust chromium(III)-fluoride complexes is a convenient route to small heterometallic complexes with fluoride bridges. These polynuclear complexes constitute the first examples of unsupported fluoride-bridges between a paramagnetic transition metal and a lanthanide ion. The propensity of fluoride for linear bridging established for

polynuclear transition metal systems is manifest also in mixed 3d-4f systems. The resulting, structurally simple systems allowed for modeling of the magnetic properties and for the first quantification of magnetic coupling between 3d and 4f centers across a fluoride bridge. This coupling was found to be numerically similar to that for cyanide or oxalate bridges, while the lack of systems with isoelectronic hydroxide or alkoxide bridges between chromium and gadolinium prevents direct comparison with these bridging ligands. The coupling appears to decrease pronouncedly with the bending of the fluoride bridges. This can be favorably exploited for achieving a large MCE, since a relatively large number of magnetic degrees of freedom become available at low temperature because of the low-lying excited spin states promoted by the weak coupling. The weakest intracluster interactions are achieved in the bipyramidally shaped {Gd_3Cr_2} (**11**) complex. Indeed, for **11** we report a remarkably large MCE at low temperatures, which results also from the large net magnetic moment of the molecule, combined with its negligible anisotropy. Furthermore, **11** has a relatively large metal/ligand mass ratio that is an important advantage since the nonmagnetic ligands contribute passively to the MCE.

■ EXPERIMENTAL SECTION

Synthesis: Materials. $Ce(NO_3)_3·6H_2O$ (Puriss. p.a., Fluka), $Pr(NO_3)_3·H_2O$ (99.9%, Alfa Aesar), $Nd(NO_3)_3·6H_2O$ (99.9%, Alfa Aesar), $Sm(NO_3)_3·6H_2O$ (99.9%, Alfa Aesar), $Eu(NO_3)_3·6H_2O$ (99.9%, Alfa Aesar), $Gd(NO_3)_3·H_2O$ (99.9%, Alfa Aesar), $Tb(NO_3)_3·5H_2O$ (99.9%, Aldrich), $Dy(NO_3)_3·5H_2O$ (99.9%, Alfa Aesar), $Ho(NO_3)_3·5H_2O$ (99.9%, Aldrich), $Er(NO_3)_3·5H_2O$ (99.9%, Alfa Aesar), $Yb(NO_3)_3·H_2O$ (99.9%, Alfa Aesar), 1,10-phenanthroline (Alfa Aesar), and the solvents MeOH (Lab Scan.), 2-methoxyethanol (ACS 99.3+%, Alfa Aesar) were used as received.

cis-$[CrF_2(phen)_2]NO_3$ was synthesized using a slightly modified version of the method for synthesis of perchlorate salt.[8a] *trans*-$[CrF_2(py)_4]NO_3$ (0.13 mol) and 1,10-phenanthroline (0.25 mol) were refluxed in 2-methoxyethanol (200 mL) for 1 h. The resulting pink-orange product was collected by filtration and washed repeatedly with EtOH (99%, 4×100 mL). Yield: ∼85%. *fac*-$[CrF_3(Me_3tame)]·H_2O$ and *fac*-$[CrF_3(Me_3tacn)]$ were synthesized by the general protocol applied in the above reference using 2-methoxyethanol and DMF as solvents, respectively. Yields ranged from 65 to 75%. $[Ln(hfac)_3(H_2O)_2]$ (Ln = Sm, Gd, Ho, Y) was synthesized as described in literature.[25]

General Synthesis of {$[CrF_2(phen)_2][Ln(NO_3)_4]$}$_2$. All of the tetranuclear clusters were prepared by the same general protocol given here. A solution of *cis*-$[CrF_2(phen)_2]NO_3$(0.82 mmol) in MeOH (20 mL) was stirred at RT for 30 min and filtered twice through syringe filters with pore size 0.45 μm. This resulting solution (I) was diluted with an additional 20 mL of MeOH (for prep. of Pr and Nd compounds) or 10 mL of MeOH (for the remaining lanthanoids). A second solution (II) was made up of $Ln(NO_3)_3·aq$ (0.81 mmol) in MeOH (10 mL). The two solutions were mixed and left for crystallization for 24 h at RT. Yields do not improve by preparation at 0 °C. The resulting mass of red crystals and white powder (the ratio between these depends on lanthanoid in question) was loosened and brought into suspension. Crystals were harvested by decantation of the byproduct in suspension. The crystals were washed repeatedly with MeOH by decantation until all the pale colored byproduct was removed, and dried on a sintered glass filter in a dynamic vacuum. Upon drying, solvent loss causes the crystals to lose their luster. A faster precipitation of the product in microcrystalline form can be achieved for Pr, Nd, and Sm by not adjusting the volume of solution I with additional MeOH. The suspension of byproduct in MeOH was transferred to centrifuge tubes and centrifuged, washed thoroughly with MeOH, and dried in a dynamic vacuum.

Yields and analytical data for the individual compounds are given below. Note, that drying has resulted in partial solvent loss. For all compounds except that of Gd, the calculated values are given for the solvent free composition.

{$[CrF_2(phen)_2][Ce(NO_3)_4]$}$_2$ (**1**): Yield: 74% of theoretical based on Ce^{III}. Elemental analysis calcd (%) for $H_{32}C_{48}N_{16}O_{24}F_4Cr_2Ce_2$: H 1.92, C 34.38, N 13.36; found: H 1.72, C 32.83, N 12.36. By-product: None.

{$[CrF_2(phen)_2][Pr(NO_3)_4]$}$_2$ (**2**):Yield: 82% of theoretical based on Pr^{III}. Elemental analysis calcd (%) for $H_{32}C_{48}N_{16}O_{24}F_4Cr_2Pr_2$: H 1.92, C 34.34, N 13.35; found: H 1.94, C 33.44, N 12.44. By-product: Present, but small amount.

{$[CrF_2(phen)_2][Nd(NO_3)_4]$}$_2$ (**3**): Yield: 87% of theoretical based on Nd^{III}. Elemental analysis calcd (%) for $H_{32}C_{48}N_{16}O_{24}F_4Cr_2Nd_2$: H 1.91, C 34.21, N 13.30; found: H 2.05, C 34.24, N 12.69. By-product: Present, but small amount.

{$[CrF_2(phen)_2][Sm(NO_3)_4]$}$_2$ (**4**): Yield: 87% of theoretical based on Sm^{III}. Elemental analysis calcd (%) for $H_{32}C_{48}N_{16}O_{24}F_4Cr_2Sm_2$: H 1.90, C 33.96, N 13.20; found: H 2.08, C 33.79, N 12.52. By-product: 0.035 g (Analysis: Found (%): H, 1.62; C, 24.41; N, 7.99).

{$[CrF_2(phen)_2][Eu(NO_3)_4]$}$_2$ (**5**): Yield: 37% of theoretical based on Eu^{III}. Elemental analysis calcd (%) for $H_{32}C_{48}N_{16}O_{24}F_4Cr_2Eu_2$: H 1.90, C 33.90, N 13.18; found (%): H 1.97, C 33.59, N 12.10. By-product: 0.070 g (Analysis: Found (%): H, 1.76; C, 26.85; N, 9.16).

{$[CrF_2(phen)_2][Gd(NO_3)_4]$}$_2·CH_3OH·H_2O$ (**6**): Yield: 27% of theoretical based on Gd^{III}. Elemental analysis calcd (%) for $H_{38}C_{49}N_{16}O_{26}F_4Cr_2Gd_2$: H 2.17, C 33.41, N 12.72; found: H 2.06, C 33.53, N 12.25. By-product: 0.05 g (Analysis: Found (%): H, 1.64; C, 25.02; N, 7.99).

{$[CrF_2(phen)_2][Tb(NO_3)_4]$}$_2$ (**7**): Yield: 5.5% of theoretical based on Tb^{III}. Elemental analysis calcd (%) for $H_{32}C_{48}N_{16}O_{24}F_4Cr_2Tb_2$: H 1.88, C 33.62, N 13.07; found: H 1.85, C 33.07, N 12.06. By-product: 0.082 g (Analysis: Found (%): H, 1.63; C, 24.41; N, 7.81).

{$[CrF_2(phen)_2][Dy(NO_3)_4]$}$_2$ (**8**): Yield: 1.8% of theoretical based on Dy^{III}. Elemental analysis calcd (%) for $H_{32}C_{48}N_{16}O_{24}F_4Cr_2Eu_2$: H 1.87, C 33.48, N, 13.02; found: H 1.79, C 32.09, N 12.19. The isolated crystalline product is contaminated with a crystalline impurity (cf. Figure 1) By-product: 0.097 g (Analysis: Found (%): H, 1.67; C, 24.36; N, 7.81).

{$[CrF_2(phen)_2][Ho(NO_3)_4]$}$_2$ (**9**): Yield: Few crystals. By-product: 0.107 g (Analysis: Found (%): H, 1.61; C, 24.09; N, 7.75).

Attempted synthesis of {$[CrF_2(phen)_2][Er(NO_3)_4]$}$_2$: Yield: None. By-product: 0.093 g (Analysis: Found (%): H, 1.64; C, 23.72; N, 7.65).

Attempted synthesis of {$[CrF_2(phen)_2][Yb(NO_3)_4]$}$_2$: Yield: None. By-product: 0.016 g (Analysis: Found (%): H, 1,24; C, 13.36; N, 5.11).

Synthesis of {$[CrF_2(bpy)_2][Ln(NO_3)_4]$}$_2$ (Ln = Nd, Sm, Eu, Gd). This synthesis proceeds similarly as for the phenanthroline analogues. Details are provided in the Supporting Information.

Synthesis of [{$CrF_3(Me_3tame)$}$_2Ln_3(hfac)_6(\mu$-$F)_3$]$·7CH_3CN$ (Ln = Sm (10), Gd (11), Ho (12), Yb(13)). $[Ln(hfac)_3(H_2O)_2]$ (0.61 mmol) was dissolved in MeCN (5 mL), and the resulting solution was added to a suspension of *fac*-$[CrF_3(Me_3tame)]·3H_2O$ (0.41 mmol) in MeCN (5 mL). The mixture was stirred for 2 min and filtered. The filtrate was kept for 24 h to yield red-purple, X-ray quality crystals. Yields: 20–25% (based on Ln). When the crystals are removed from the mother liquor, desolvation takes place and the crystals deteriorate. Thoroughly dried products yield the following elemental analyses: **10:** calcd (%) for $H_{48}C_{44}N_6O_{12}F_{45}Cr_2Sm_3$: H 2.14, C 23.35, N 3.71; found: H 2.12, C 23.15, N 3.38. **11:** calcd (%) for $H_{48}C_{44}N_6O_{12}F_{45}Cr_2Gd_3$: H 2.12, C 23.14, N 3.68; found: H 2.07, C 23.12, N 3.37. **12:** calcd (%) for $H_{48}C_{44}N_6O_{12}F_{45}Cr_2Ho_3$: H 2.10, C 22.91, N 3.64; found: H 1.92, C 23.17, N 3.52. **13:** calcd (%) for $H_{48}C_{44}N_6O_{12}F_{45}Cr_2Yb_3$: H 2.08, C 22.68, N 3.61; found: H 1.98, C 22.60, N 3.30.

Synthesis of [{$CrF_3(Me_3tacn)$}$_2Nd_3(NO_3)_8(\mu_3$-$F)(H_2O)$]$·6CH_3CN$ (14). A solution of *fac*-$[CrF_3(Me_3tacn)]$ (0.100 g, 0.36 mmol) in MeCN (10 mL) was allowed to diffuse through a porous glass frit (D4) into a solution of $Nd(NO_3)_3·6H_2O$ (0.205 g, 0.47 mmol) in

MeCN (20 mL) at 5 °C. Over a period of days pink rod-shaped crystals develop. These are very often hollow and encapsulate some of the mother liquor. Upon drying they lose solvent and crumble. The best elemental analyses were obtained by gently crushing of the crystalline product, repeated washing with MeCN, and storage below 0 °C. Elemental analysis calcd (%) for $H_{62}C_{30}N_{20}O_{25}F_7Cr_2Nd_3$: H 3.53, C 20.33, N 15.80; found: H 3.58, C 20.08, N 15.47.

Physical Measurements. Elemental analysis for C, H, and N was performed with a CE Instrument: FLASH 1112 series EA, at the microanalytical laboratory, University of Copenhagen. UV/vis spectra were recorded on a Perkin-Elmer, Lambda 2 UV/vis spectrophotometer. Electrospray mass spectra were recorded on a micromass Q-Tof spectrometer employing cone-voltages in the range 20−45 V from solutions with concentrations in the nominal range 5−50 μM.

X-ray Crystallography. All single-crystal X-ray diffraction data were collected at 122(1) K on a Nonius KappaCCD area-detector diffractometer, equipped with an Oxford Cryostreams low-temperature device, using graphite-monochromated Mo$K\alpha$ radiation (λ = 0.71073 Å). The structures were solved using direct methods (SHELXS97) and refined using the SHELXL97 software package.[26] Crystals suitable for X-ray diffraction were obtained direct from the synthetic procedure. All non-hydrogen atoms were refined anisotropically. Hydrogen atoms were located in the difference Fourier map and refined isotropically and constrained riding their parent atom in a fixed geometry. In **3** and **6** the water of crystallization is located on a crystallographic 4-fold axis, and the hydrogen atoms could not be located. Crystals of **11** and **12** were extremely fragile, thin plates, which had to be transferred directly from the mother liquor to mineral oil to prevent rapid solvent loss. Hence, only low quality data could be obtained for **11** and **12**; in both structures the hfac$^-$ ligands coordinated to one of the Ln-atoms are disordered. The disorder was resolved by refining the hfac$^-$ ligands in two positions, equally populated, with one set angled 69° with respect to the other. Crystal structure and refinement data for **6**, **11**, **12**, **14**, and$\{[CrF_2(bpy)_2][Nd(NO_3)_4]\}_2$ are summarized in Supporting Information, Tables S1a and S1b. CCDC reference numbers: 837951 (**6**), 838473 (**11**), 838474 (**12**), 861406 (**14**), and 860725 ($\{[CrF_2(bpy)_2][Nd(NO_3)_4]\}_2$). These data can be obtained free of charge from The Cambridge Crystallographic Data Centre via www.ccdc.cam.ac.uk/data_request/cif. The molecular structure diagrams were produced with the Mercury program ver. 2.4 from The Cambridge Crystallographic Data Center. Powder X-ray crystallographic data were collected on a STOE Stadi-P powder diffractometer equipped with PSD-detector using Cu (1.54060 Å) radiation monochromated with curved germanium. Data were subtracted a background by use of the STOE WinXPOW software ver. 1.10. Theoretical powder diffractograms were generated from the single crystal structure by use of the software "Mercury CSD 2.2" from The Cambridge Crystallographic Data Center.[27]

Magnetic Measurements. The magnetic measurements were conducted on a MPMS-XL Quantum-Design SQUID magnetometer located at University of Copenhagen. All measurements were performed on polycrystalline samples immobilized in a frozen *n*-eicosane matrix to avoid torqueing. The susceptibilities were corrected for diamagnetic contributions from the sample holder, *n*-eicosane, and the sample by means of Pascal's constants. Alternating current (ac) susceptibility measurements were measured with various frequencies in the range 1−1500 Hz with an ac field amplitude of 3 Oe with and without an applied static field (H_{dc}< 2 kOe). The modeling was performed with MagProp[28] and home-written software. Heat capacity measurements using the relaxation method down to ~0.3 K on powder samples were carried out by means of a commercial setup for the 0 < B_0 < 9 T magnetic field range.

ASSOCIATED CONTENT

Supporting Information

Table of crystallographic data for **6**, **11**, **12**, and **14** (Table S1a) as well as for $\{[CrF_2(bpy)_2][Nd(NO_3)_4]\}_2$ (Table S1b). Powder diffraction data for $\{[CrF_2(bpy)_2][Ln(NO_3)_4]\}_2$ (Ln = Pr, Nd, Sm, Eu, Gd). Magnetization data for compounds **2**, **3**, **4**, and **11**. Syntheses and analytical data for $\{[CrF_2(bpy)_2]$-$[Ln(NO_3)_4]\}_2$ (Ln = Nd, Sm, Eu, Gd). Electrospray mass spectra for compounds **1**, **4**, and **6** as well as for compounds **10**−**13**. This material is available free of charge via the Internet at http://pubs.acs.org.

AUTHOR INFORMATION

Corresponding Author

*Fax: +45 35320212. E-mail: bendix@kiku.dk.

Notes

The authors declare no competing financial interest.

ACKNOWLEDGMENTS

J.B. and H.W. thank the Danish Research Councils for support under Grant FNU: 272-08-0491. S.P. thanks FNU Sapere Aude (Grant 10-081659).

REFERENCES

(1) (a) Sessoli, R.; Gatteschi, G.; Caneschi, A.; Novak, M. A. *Nature* **1993**, *45*, 141−143. (b) Gatteschi, D.; Caneschi, A.; Pardi, L.; Sessoli, R. *Science* **1994**, *265*, 1054−1058. (c) Ishikawa, N.; Sugita, M.; Ishikawa, T.; Koshihara, S.; Kaizu, Y. *J. Am. Chem. Soc.* **2003**, *125*, 8694−8695. (d) Ishikawa, N. *Polyhedron* **2007**, *26*, 2147−2153. (e) Milios, C. J.; Vinslava, A.; Wernsdorfer, W.; Moggach, S.; Parsons, S.; Perlepes, S. P.; Christou, G.; Brechin, E. K. *J. Am. Chem. Soc.* **2007**, *129*, 2754−2755. (f) AlDamen, M. A.; Clemente-Juan, J. M.; Coronado, E.; Marti-Gastaldo, C.; Gaita-Arino, A. *J. Am. Chem. Soc.* **2008**, *130*, 8874−8875. (g) Sessoli, R.; Powell, A. K. *Coord. Chem. Rev.* **2009**, *253*, 2328−2341. (h) Sorace, L.; Benelli, C.; Gatteschi, D. *Chem. Soc. Rev.* **2011**, *40*, 3092−3104. (i) Rinehart, J. D.; Long, J. R. *Chem. Sci.* **2011**, *2*, 2078−2085. (j) Jiang, S.-D.; Wang, B.-W.; Sun, H.-L.; Wang, Z.-M.; Gao, S. *J. Am. Chem. Soc.* **2011**, *133*, 4730−4733. (k) Hewitt, I. J.; Tang, J.; Madhu, N. T.; Anson, C. E.; Lan, Y.; Luzon, J.; Etienne, M.; Sessoli, R.; Powell, A. K. *Angew. Chem., Int. Ed.* **2010**, *49*, 6352−6356. (l) Lin, P.-H.; Burchell, T. J.; Ungur, L.; Chibotaru, L. F.; Wernsdorfer, W.; Murugesu, M. *Angew. Chem., Int. Ed.* **2009**, *48*, 9489−9492. (m) Blagg, R. J.; Muryn, C. A.; McInnes, E. J. L.; Tuna, F.; Winpenny, R. E. P. *Angew. Chem., Int. Ed.* **2011**, *50*, 6530−6533.

(2) (a) Evangelisti, M.; Brechin, E. K. *Dalton Trans.* **2010**, *39*, 4672−4676. (b) Karotsis, G.; Evangelisti, M.; Dalgarno, S. J.; Brechin, E. K. *Angew. Chem., Int. Ed.* **2009**, *48*, 9928−9921. (c) Sharples, J. W.; Zheng, Y.-Z.; Tuna, F.; McInnes, E. J. L. *Chem. Commun.* **2011**, *47*, 7650−7652. (d) Evangelisti, M.; Roubeau, O.; Palacios, E.; Camón, A.; Hooper, T. N.; Brechin, E. K.; Alonso, J. J. *Angew. Chem., Int. Ed.* **2011**, *50*, 6606−6609. (e) Zheng, Y.-Z.; Evangelisti, M.; Winpenny, R. E. P. *Angew. Chem., Int. Ed.* **2011**, *50*, 3692−3695. (f) Peng, J.-B.; Zhang, Q.-C.; Kong, X.-J.; Ren, Y.-P.; Long, L.-S.; Huang, R.-B.; Zheng, L.-S.; Zheng, Z. *Angew. Chem., Int. Ed.* **2011**, *50*, 10649−10652. Langley, S. K.; Chilton, N.; Moubaraki, B.; Hooper, T.; Brechin, E. K.; Evangelisti, M.; Murray, K. S. *Chem. Sci.* **2011**, *2*, 1166−1169.

(3) (a) Winpenny, R. E. P. *Chem. Soc. Rev.* **1998**, *27*, 447−452. (b) Benelli, C.; Gatteschi, D. *Chem. Rev.* **2002**, *102*, 2369−2388. (c) Andruh, M.; Costes, J.-P.; Diaz, C.; Gao, S. *Inorg. Chem.* **2009**, *48*, 3342−3359. (d) Osa, S.; Kido, T.; Matsumoto, N.; Re, N.; Pochaba, A.; Mrozinski, J. *J. Am. Chem. Soc.* **2004**, *126*, 420−421. (e) Mishra, A.; Wernsdorfer, W.; Abboud, K. A.; Christou, G. *J. Am. Chem. Soc.* **2004**, *126*, 15648−15649. (f) Mori, F.; Ishida, T.; Nogami, T. *Polyhedron* **2005**, *24*, 2588−2592. (g) Costes, J.-P.; Dahan, F.; Wernsdorfer, W. *Inorg. Chem.* **2006**, *45*, 5−7. (h) Aronica, C.; Pilet, G.; Chastanet, G.; Wernsdorfer, W.; Jacquot, J. F.; Luneau, D. *Angew. Chem., Int. Ed.* **2006**, *45*, 4659−4662. (i) Chandrasekhar, V.; Pandian, B. M.; Azhakar, R.; Vittal, J. J.; Clérac, R. *Inorg. Chem.* **2007**, *46*, 5140−5142. (j) Mereacre, V.; Ako, A. M.; Clérac, R.; Wernsdorfer, W.; Hewitt, I. J.; Anson, C. E.; Powell, A. K. *Chem.—Eur. J.* **2008**, *14*, 3577−3584. (k) Ke, H.; Xu, G.-F.; Zhao, L.; Tang, J.; Zhang, X.-Y.; Zhang, H.-J. *Chem.—Eur. J.* **2009**, *15*, 10335−10338. (l) Rinck, J.; Novitchi, G.; Van den Heuvel, W.; Ungur, L.; Lan, Y.; Wernsdorfer, W.; Anson, C.

E.; Chibotaru, L. F.; Powell, A. K. *Angew. Chem., Int. Ed.* **2010**, *49*, 7583−7587. (m) Yamaguchi, T.; Costes, J.-P.; Kishima, Y.; Kojima, M.; Sunatsuki, Y.; Bréfuel, N.; Tuchagues, J.-P.; Vendier, L.; Wernsdorfer, W. *Inorg. Chem.* **2010**, *49*, 9125−9135. (n) Schray, D.; Abbas, G.; Lan, Y.; Mereacre, V.; Sundt, A.; Dreiser, J.; Waldmann, O.; Kostakis, G. E.; Anson, C. E.; Powell, A. K. *Angew. Chem., Int. Ed.* **2010**, *49*, 5185−5188. (o) Mereacre, V.; Baniodeh, A.; Anson, C. E.; Powell, A. K. *J. Am. Chem. Soc.* **2011**, *133*, 15335−15337. (p) Papatriantafyllopoulou, C.; Abboud, K. A.; Christou, G. *Inorg. Chem.* **2011**, *50*, 8959−8966.

(4) Perdih, F.; Demsar, A.; Pevec, A.; Petricek, S.; Leban, I.; Giester, G.; Sieler, J.; Roesky, H. W. *Polyhedron* **2001**, *20*, 1967−1971.

(5) Pevec, A.; Mrak, M.; Demsar, A.; Petricek, S.; Roesky, H. W. *Polyhedron* **2003**, *22*, 575−579.

(6) McRobbie, A.; Sarwar, A. R.; Yeninas, S.; Nowell, H.; Baker, M. L.; Allan, D.; Luban, M.; Muryn, C. A.; Pritchard, R. G.; Prozorov, R.; Timco, G.; Tuna, F.; Whitehead, G. F. S.; Winpenny, R. E. P. *Chem. Commun.* **2011**, *47*, 6251−6253.

(7) Pearson, R. G. *J. Am. Chem. Soc.* **1963**, *85*, 3533−3539.

(8) (a) Glerup, J.; Josephsen, J.; Michelsen, K.; Pedersen, E.; Schäffer, C. E. *Acta Chem. Scand.* **1970**, *24*, 247−254. (b) Andersen, P.; Døssing, A.; Glerup, J.; Rude, M. *Acta Chem. Scand.* **1990**, *44*, 346−352. (c) Böttcher, A.; Elias, H.; Glerup, J.; Neuburger, M.; Olsen, C. E.; Paulus, H.; Springborg, J.; Zehnder, M. *Acta Chem. Scand.* **1994**, *48*, 967−980. (d) Böttcher, A.; Elias, H.; Glerup, J.; Neuburger, M.; Olsen, C. E.; Springborg, J.; Weihe, H.; Zehnder, M. *Acta Chem. Scand.* **1994**, *48*, 981−988.

(9) Birk, T.; Magnussen, M. J.; Piligkos, S.; Weihe, H.; Holten, A.; Bendix, J. *J. Fluorine Chem.* **2010**, *131*, 898−906.

(10) Dreiser, J.; Pedersen, K. S.; Piamonteze, C.; Rusponi, S.; Salman, Z.; Ali, Md. E.; Schau-Magnussen, M.; Thuesen, C. Aa.; Piligkos, S.; Weihe, H.; Mutka, H.; Waldmann, O.; Oppeneer, P.; Bendix, J.; Nolting, F.; Brune, H. *Chem. Sci.* **2012**, *3*, 1024−1032.

(11) Birk, T.; Schau-Magnussen, M.; Weyhermüller, T.; Bendix, J. *Acta Crystallogr.* **2011**, *E67*, m1561−m1562.

(12) Rajendiran, T. M.; Kahn, O.; Golhen, S.; Ouahab, L.; Honda, Z.; Katsumata, K. *Inorg. Chem.* **1998**, *37*, 5693−5696.

(13) Birk, T.; Bendix, J.; Weihe, H. *Acta Crystallogr.* **2008**, *E64*, m369−m370.

(14) Meally, S. T.; Mason, K.; McArdle, P.; Brechin, E. K.; Ryder, A. G.; Jones, L. F. *Chem. Commun.* **2009**, 7024−7026.

(15) Tereshchenko, D. S.; Morozov, I. V.; Boltalin, A. I.; Kemnitz, E.; Troyanov, S. I. *Zh. Neorg. Khim.* **2004**, *49*, 919−927.

(16) (a) Kaizaki, S.; Takemoto, H. *Inorg. Chem.* **1990**, *29*, 4960−4964. (b) Terasaki, Y.; Kaizaki, S. *J. Chem. Soc., Dalton Trans.* **1995**, 2837−2841. (c) Terasaki, Y.; Fujihara, T.; Schönherr, T.; Kaizaki, S. *Inorg. Chim. Acta* **1999**, *295*, 84−90.

(17) Decurtins, S.; Gross, M.; Schmalle, H. W.; Ferlay, S. *Inorg. Chem.* **1998**, *37*, 2443−2449.

(18) Although numerically small, inclusion of both parameters, $J_{Cr\text{-}Gd}$ and $J_{Gd\text{-}Gd}$ is statistically warranted since they by the fitting both have relative uncertainties well below 10% and a mutual correlation coefficient of 0.078.

(19) (a) Figuerola, A.; Diaz, C.; El Fallah, M. S.; Ribas, J.; Maestro, M.; Mahía, J. *Chem. Commun.* **2001**, 1204−1205. (b) Kou, H.-Z.; Gao, S.; Li, C.-H.; Liao, D.-Z.; Zhou, B.-C.; Wang, R.-J.; Li, Y. *Inorg. Chem.* **2002**, *41*, 4756−4762. (c) Akitsu, T.; Einaga, Y. *Inorg. Chim. Acta* **2006**, *359*, 1421−1426. (d) Sun, X.-R.; Chen, Z.-D.; Wang, M.-W.; Wang, B.-W.; Yan, F.; Cheung, K.-K. *Chin. J. Chem.* **2007**, *25*, 329−336.

(20) Sanada, T.; Suzuki, T.; Yoshida, T.; Kaizaki, S. *Inorg. Chem.* **1998**, *37*, 4712−4717.

(21) Hodgson, D. J.; Michelsen, K.; Pedersen, E. *J. Chem. Soc., Chem. Commun.* **1988**, *23*, 1558−1559.

(22) Pedersen, K. S.; Thuesen, C. Aa.; Schau-Magnussen, M.; Bendix, J., manuscript in preparation.

(23) Evangelisti, M.; Luis, F.; de Jongh, L. J.; Affronte, M. *J. Mater. Chem.* **2006**, *16*, 2534−2549.

(24) Sessoli, R. *Angew. Chem., Int. Ed.* **2012**, *51*, 43−45.

(25) Richardson, M. F.; Wagner, W. F.; Sands, D. E. J. *Inorg. Nucl. Chem.* **1968**, *30*, 1275−1289.

(26) Sheldrick, G. M. *Acta Crystallogr.* **2008**, *A64*, 112−122.

(27) Macrae, C. F.; Bruno, I. J.; Chisholm, J. A.; Edgington, P. R.; McCabe, P.; Pidcock, E.; Rodriguez-Monge, L.; Taylor, R.; van de Streek, J.; Wood, P. A. *J. Appl. Crystallogr.* **2008**, *41*, 466−470.

(28) Tregenna-Piggott, P. L. W.; Sheptyakov, D.; Keller, L.; Klokishner, S. I.; Ostrovsky, S. M.; Palii, A. V.; Reu, O. S.; Bendix, J.; Brock-Nannestad, T.; Pedersen, K.; Weihe, H.; Mutka, H. *Inorg. Chem.* **2009**, *48*, 128−137.

PAPER 8

XMCD Study of a of Methoxide-Bridged Dy^{III}–Cr^{III} Cluster Obtained by Fluoride Abstraction from *cis*-$[Cr^{III}(phen)_2F_2]^+$

Jan Dreiser, Kasper Steen Pedersen, Torben Birk, Magnus Schau-Magnussen, Cinthia Piamonteze, Stefano Rusponi, Thomas Weyhermüller, Harald Brune, Frithjof Nolting and Jesper Bendix

Submitted to *The Journal of Physical Chemistry*.

XMCD Study of a Methoxide-Bridged Dy^{III}–Cr^{III} Cluster Obtained by Fluoride Abstraction from *cis*-$[Cr^{III}F_2(phen)_2]^+$

Jan Dreiser, [†]* *Kasper S. Pedersen,*[‡] *Torben Birk,*[‡] *Magnus Schau-Magnussen,*[‡] *Cinthia Piamonteze,*[†] *Stefano Rusponi,*[¶] *Thomas Weyhermüller,*[#] *Harald Brune,*[¶] *Frithjof Nolting,*[†] *and Jesper Bendix*[‡]*

[†]Swiss Light Source, Paul Scherrer Institut CH-5232 Villigen PSI, Switzerland, [‡]Department of Chemistry, University of Copenhagen, DK-2100 Copenhagen, Denmark, [¶]Institute of Condensed Matter Physics, Ecole Polytechnique Fédérale de Lausanne, CH-1015 Lausanne, Switzerland, [#]Max Planck Institute for Bioinorganic Chemistry, D-45470 Mülheim an der Ruhr, Germany.

*Authors to whom correspondence should be addressed. Email: jan.dreiser@psi.ch, bendix@kiku.dk

RECEIVED 12-April-12

Abstract

An isostructural series of dinuclear chromium(III)-lanthanide(III) clusters is formed by fluoride abstraction of *cis*-$[CrF_2(phen)_2]^+$ by Ln^{3+} resulting in LnF_3 and methoxide-bridged Cr–Ln clusters (Ln = Nd (**1**), Tb (**2**), Dy (**3**)) of formula $[Cr^{III}(phen)_2(\mu\text{-}MeO)_2Ln(NO_3)_4]\cdot x$MeOH ($x$ = 2–2.73). In contrast to fluoride, methoxide bridges in a non-linear fashion, which facilitates chelation. For **3**, X-ray magnetic circular dichroism (XMCD) provides element-specific magnetization curves which are compared to cluster magnetization and susceptibility data acquired by SQUID magnetometry. The combination of XMCD and SQUID is able to resolve very small magnetic coupling values and reveals a weak Cr^{III} – Dy^{III} coupling of j = –0.04(3) cm^{-1}. The Dy^{III} ion has a ground-state Kramers doublet of m_J = ±13/2, and the first excited doublet is found to be m_J = ±11/2 at an energy of δ = 57(21) cm^{-1}. The Cr^{III} ion exhibits a uniaxial anisotropy of D_{Cr} = −1.7(1.0) cm^{-1}. Further, we observe that a weak anisotropic coupling of dipolar origin is sufficient to model the data, suggesting that methoxide bridges do not play a significant role in the magnetic coupling for the present systems.

Keywords: lanthanides, chromium, clusters, magnetic exchange interaction

Introduction

Molecular nanomagnets[1] have attracted a lot of interest because of their fascinating properties and their prospects in future applications in quantum information processing and molecular spintronics.[2] In particular, there is increasing activity regarding 3d-4f single-molecule magnets, and numerous species have been reported so far.[3,4] The complete understanding of their magnetic behavior is, however, lagging behind and for instance the key to the necessary ingredients that mediate properties such as slow relaxation of magnetization in 3d-4f clusters is still missing.[5] Difficulties in finding this key are aggravated by the complexity of the magnetism of 4f ions with orbital contribution to their ground state.[6] In particular, it is difficult to obtain precise values of the magnetic exchange coupling between 3d and 4f ions: In 3d clusters, this information can be readily extracted from dc magnetic susceptibility measurements, however, the large anisotropy splittings resulting from the 4f ligand-field states can strongly influence the temperature dependence of the magnetic susceptibility. Hence exchange coupling deduced solely from susceptibility data may be subject to large uncertainties. The problem can be circumvented by using additional methods such as diamagnetic substitution,[7, 8] electron paramagnetic resonance[8] or inelastic neutron scattering studies.[9] X-ray magnetic circular dichroism[10] (XMCD) is able to obtain absolute values of element-specific magnetization, which can in turn be exploited to obtain a sensitive measurement of very small magnetic coupling values, and thus it is among the techniques of choice to quantify such coupling in 3d-4f clusters.[11] Precise knowledge about the magnetic exchange coupling is the prerequisite for obtaining its dependence along the homologous series of lanthanides and for establishing magneto-structural correlations,[3a,12] and thus it is of importance for the rational design of polynuclear 3d-4f complexes. The recent detailed studies of 3d-4f magnetic interactions have revealed a number of non-isotropic interaction modes such as anisotropic or antisymmetric interactions. Disregarding the interaction mode, most studies have reported weak 3d-4f interactions ($j_{3d\text{-}4f} < 1$ cm^{-1}). However, also for systems with weakly interacting magnetic centers and concomitant high ground state degeneracies, the quantification of the interaction strength is important. Such systems are receiving rapidly growing interest due to their large magnetocaloric effects and

potential applications in magnetic refrigeration.[13] Recently, we reported the first examples of 3d-4f clusters assembled by unsupported fluoride bridges.[11,14] Here we report the synthesis and the structural characterization of a novel series of methoxide-bridged clusters: $[Cr^{III}(phen)_2(\mu\text{-}MeO)_2Ln(NO_3)_4]\cdot x$MeOH with Ln = Nd (**1**), Tb (**2**) and Dy (**3**). As for the previously reported, fluoride-bridged systems, the chromium precursor in the present study is the robust *cis*-$[Cr^{III}F_2(phen)_2]^+$-ion. However, instead of targeting the kinetic product in form of tetranuclear $[\{CrF_2(phen)_2\}_2\{Ln(NO_3)_4\}_2]$ squares, we have found that the reaction can be driven more towards thermodynamic products, by use of more dilute solutions and longer reaction times. The approach relies on the high affinity of the lanthanides for fluoride, which leads to eventual abstraction of fluoride from the Cr(III) precursor complex and introduction of solvent-derived bridges. This may constitute a generalizable synthetic approach to systems with alkoxide-bridged lanthanide clusters, some of which have recently been shown to posess SMM properties with relatively high relaxation barriers.[15] For **3** a detailed magnetic study involving XMCD and SQUID magnetometry has been employed to characterize the exchange coupling and magnetic anisotropies.

Experimental Section

Synthesis: *cis*-$[CrF_2(phen)_2]NO_3$ was prepared according to the literature.[14a] $Ln(NO)_3\cdot$aq and methanol were obtained by commercial sources (Alfa Aesar and Lab Scan HPLC, respectively) and used without further purification.

General synthesis of $[(phen)_2Cr(\mu\text{-}CH_3O)_2Ln(NO_3)_4]$: (Ln = Nd (**1**), Tb (**2**), Dy (**3**)): All complexes were prepared at room temperature by slow diffusion of methanol solutions of the two reactants. The synthesis was carried out in a custom-made diffusion cell consisting of three consecutive chambers separated by two porous glass frits (No. 4). Each chamber has a volume of approx. 20 mL and can be sealed. In the two outer chambers solutions of *cis*-$[CrF_2(phen)_2]NO_3$ (0.6 mmol) in MeOH (20 mL) and $Ln(NO_3)_3\cdot$aq (0.6 mmol) in MeOH (20 mL) was placed respectively, while the middle chamber was

filled with methanol. The diffusion cell was sealed and left undisturbed until no visible changes in the cell was observed over a period of 3 months. (total time of synthesis: 6-12 months). During this time of crystallization, large red crystals together with a fine white powder were formed in all three chambers. In the chamber where *cis*-$[CrF_2(phen)_2]NO_3$ was originally placed, the white byproduct dominated considerably and the content of this chamber was discarded. The red crystals from the remaining chambers were harvested by gentle scratching with a spatula and repeatedly washed by decantation with methanol. This procedure directly gave crystals suitable for single crystal diffraction. (**1**): Yield: 0.141 g (46.1 % of theoretical based on Nd^{III}). Slightly lower yields were obtained for **2** and **3**. Elemental analysis calcd(%) for $H_{22}C_{26}N_8O_{14}CrNd$ (dried) (**1**): H, 2.30; C, 35.58; N, 12.51. Found (%): H, 2.33; C, 35.16; N, 12.39. Elemental analysis calcd (%) for $H_{22}C_{26}N_8O_{14}CrDy$ (dried): H, 2.51; C, 35.29; N,12.66. Found (%): H, 2.2; C, 34.7; N, 12.3.

Crystallography: Single crystals for X-ray structure determination were obtained by the synthetic procedure outlined above. Diffraction data for **1**, **2** and **3** were collected with a Nonius KappaCCD area-detector diffractometer at T = 122 K (Oxford Cryostreams low-temperature device) employing Mo Kα (λ = 0.71073 Å) X-rays. Structures were solved by direct methods (SHELXS97) and refined with the SHELXL97 program package. Hydrogens were kept in fixed positions and all non-hydrogen atoms were treated anisotropically. Traces of co-crystallized methanol molecules were localized and the population refined. Crystallographic data are given in Table S1. CCDC-862729 (**1**), CCDC-862730 (**2**) and CCDC-862731 (**3**) contain the supplementary crystallographic data for this article. These data can be obtained free of charge from The Cambridge Crystallographic Data Centre via www.ccdc.cam.ac.uk/data_request/cif.

SQUID measurements: All magnetic measurements were conducted on a Quantum Design MPMS-XL SQUID magnetometer. The crystalline sample was ground and mechanically immobilized in *n*-eicosane to avoid orientation effects. The magnetization was measured from 1.8 K to 300 K in an

applied dc field of $\mu_0 H_0$ = 100 mT. Reduced magnetization data were obtained at temperatures below 7 K in fields up to 5 T. Susceptibility was calculated using the relation $\chi = M/H_0$ and magnetization values were corrected using Pascal constants. The ac measurements utilized an ac field of $\mu_0 h_{ac}$ = 0.3 mT with and without a static field (selected fields between 10 mT and 300 mT).

XMCD measurements: X-ray absorption measurements were performed at the X-Treme endstation and beamline at the Swiss Light Source, Paul Scherrer Institut, Switzerland.[16] X-ray absorption spectra were recorded on a powder sample of **3** at a temperature of 2 K in total electron yield mode. Magnetic fields of up to $\mu_0 H = \pm 6$ T along the beam direction were applied. The beam was defocused to a spot size of approximately 1×1 mm^2 and kept at very low intensity to exclude radiation damage. Photon-energy scans were recorded "on-the-fly", that is, the monochromator and insertion device were moving continuously while the data were acquired.[17] To measure magnetization curves, a full magnetic-field loop was performed at one circular polarization while measuring X-ray absorption at the energy of maximum dichroism and at the pre-edge. Then, the helicity of the X-rays was switched and another loop was run.

Spin-Hamiltonian calculations and fits: Element-specific and cluster magnetization curves as well as dc magnetic susceptibility were simulated by full diagonalization of Hamiltonian eq 1. Powder average over the magnetization curves and the susceptibility was calculated by use of a 16-point Lebedev-Laikov grid.[18] All fits shown in this work are least-squares fits obtained by minimizing the sum of squared deviations between the measured and calculated curves. The calculations were performed using a home-written Matlab® code.

Results

Synthesis and Structural Analysis

Cr(III) complexes are generally kinetically robust due to the ligand-field stabilization of the octahedral d^3 configuration leading to frequent cases of isomerism and isolatable kinetic products. Here, we demonstrate that for fluoride complexes, this intrinsic robustness of Cr(III) complexes can be overcome by reaction with sufficiently strong fluoride acceptors such as lanthanide ions. Consequently, fluoride complexes are also viable and practical precursors for solvent-bridged polynuclear Cr(III)-4f systems. The one-to-one stoichiometric reaction of $Ln^{III}(NO_3)_3{\cdot}aq$ and *cis*-$[CrF_2(phen)_2]^+$ in methanol produces $[Cr^{III}(phen)_2(\mu\text{-}MeO)_2Ln(NO_3)_4]$ over a timescale of months with the possible balanced reaction

$$3\ [CrF_2(phen)_2]NO_3 + 5\ Ln(NO_3)_3 + 6\ CH_3OH \rightarrow 3\ [(phen)Cr(\mu\text{-}CH_3O)_2Ln(NO_3)_4](s) + 2\ LnF_3(s) + 6\ H^+ + 6\ NO_3^-.$$

The formation of $[(phen)Cr(\mu\text{-}CH_3O)_2Ln(NO_3)_4]$ is accompanied by a precipitation of a fine white powder. This, however, is easily removed by successive decantation. The powder is mainly lanthanide(III) fluoride as evidenced by powder x-ray diffraction and elemental analysis (see Supporting Information). Lanthanide(III) fluorides are extremely insoluble owing to the very large lattice enthalpy. This fact complicates the isolation of fluoride bridged clusters and hence only very few examples are known.[14,19]

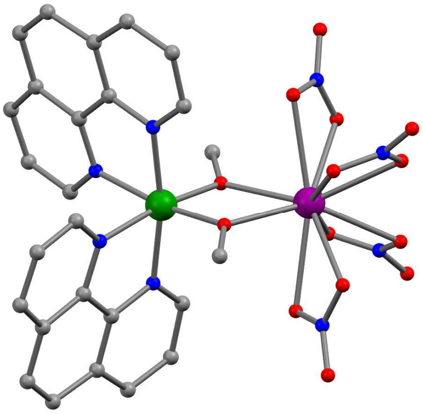

Figure 1. Ball-and-stick representation of **3**. Selected bond lengths and angles: Dy–O_{MeO} 2.324(6) Å, Dy–O_{NO_3} 2.457(8)–2.528(6) Å, Cr–O 1.921(6) Å, Cr–N 2.064(7) Å, 2.076(7) Å, Cr–O–Dy 106.8(3)°, O–Cr–O 81.3(4)°, O_{MeO}–Dy–O_{MeO} 65.2(3)°, Cr···Dy 3.42 Å.

Although alkoxide-bridged 3d-4f clusters are common, methoxide bridges are relatively rare, but the present approach might provide a generalizable approach to such systems.[20]All three members (**1**–**3**) are isostructural and crystallize in the orthorhombic *Pbcn* space group with $Z = 4$. Crystallographic data are given in Table S1. In **3**, the bridging angle imposed by the methoxide ligands is Dy–O–Cr = 106.8(3)° which is in the range of bond angles in similar systems.[15] Other relevant bond lengths and angles are given in the caption of Figure 1. Interestingly, the chelation is opposed to 3d-4f systems with unsupported fluoride bridges. In **1** and **2**, bond lengths and angles are only slightly different.

X-ray Absorption and X-ray Magnetic Circular Dichroism

Polarization-dependent X-ray absorption spectra (XAS) of **3** are shown in Figures 2a and 3a. They were recorded at 6 T and 2 K at the Cr $L_{2,3}$ and Dy $M_{4,5}$ edges, respectively. For both elements, the

spectra are strongly dichroic as reflected in the XMCD spectra shown in Figures 2b and 3b, indicating the presence of large magnetic moments.

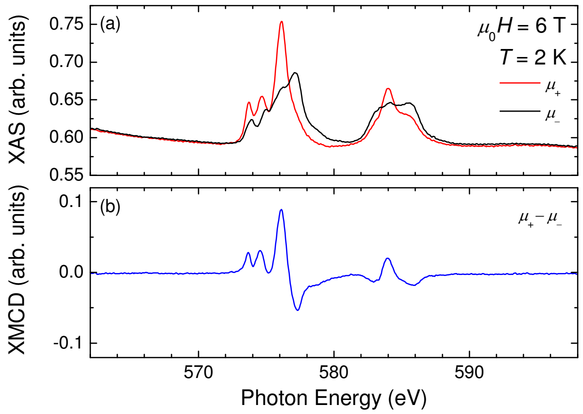

Figure 2. (a) XAS of **3** recorded at the Cr $L_{2,3}$ edges as a function of circular polarization. (b) XMCD spectrum obtained from the XAS shown in panel (a).

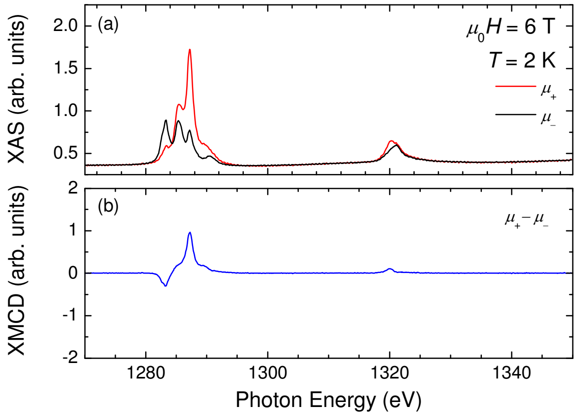

Figure 3. (a) Polarization dependent XAS of **3** recorded at the Dy $M_{4,5}$ edges as a function of circular polarization. (b) XMCD spectrum obtained from the XAS shown in panel (a).

The element-specific magnetization curves depicted in Figures 4a and 4b reveal that the magnetic moments of the Dy^{III} and Cr^{III} ions are always oriented parallel to the applied magnetic field. Blue squares in Figure 4 indicate the values of the total (spin and orbital) magnetic moments found by sum rule analysis[21] assuming ion-like hole numbers, that is N_{eff} = 7 and N_{eff} = 5 for Cr^{III} and Dy^{III}, respectively. For Dy^{III}, the average ratio of orbital versus spin angular momentum was found to be $\langle L_z \rangle / \langle S_z \rangle$ = 1.8(2), in excellent agreement with the expected value of 2.0 from Hund's rules. Details of the sum rule analysis are given in the Supporting Information. The dashed line in Figure 4a represents the calculated magnetization of an isotropic paramagnet with S = 3/2 described by the Brillouin function.

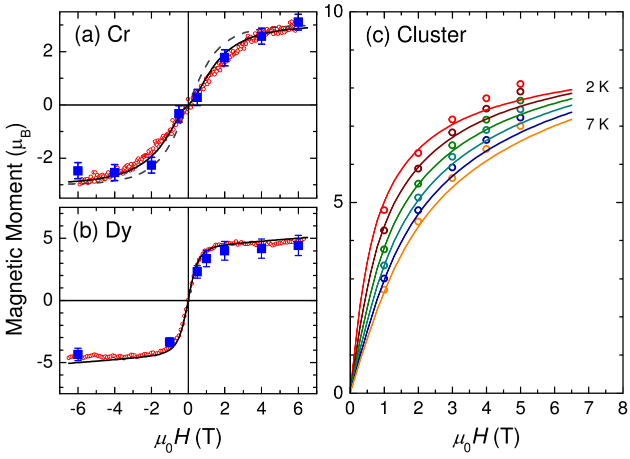

Figure 4. Element-specific magnetization curves for the Cr^{III} (a) and Dy^{III} (b) ions obtained on **3** by XMCD. The dashed line in (a) indicates the calculated magnetic moment for an isotropic S = 3/2 paramagnet using the Brillouin function. The squares mark the total (spin + orbital) magnetic moments obtained from sum rule analysis applied to couples ($\mu_+ - \mu_-$) of spectra acquired at several H fields. (c) Cluster magnetization obtained by SQUID magnetometry. Solid lines in (a,b,c) indicate best-fit curves with parameters given in the text.

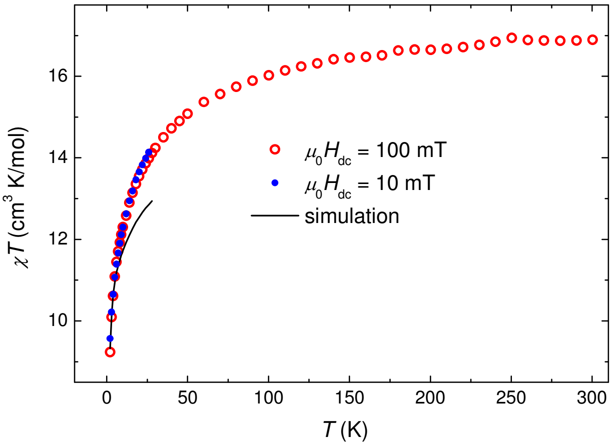

Figure 5. Dc magnetic susceptibility of **3** shown as χT product. Circles correspond to the data, and the solid line is the best-fit curve obtained using the parameters given in the text. The deviation of the model curve from the experimental data at higher temperatures is a consequence of the restricted function space of the Dy^{III} ion used in the modeling (see text for a detailed discussion).

SQUID Magnetometry

The cluster magnetization curves of **3** for temperatures of 2–7 K are plotted in Figure 4c. Clearly the magnetization does not reach saturation at the highest field of 5 T, indicating the presence of significant anisotropy and/or low-lying excited energy levels. The temperature-dependent dc magnetic susceptibility is plotted as χT product in Figure 5. At room temperature χT = 16.9 cm^3 K mol^{-1}. From this value one can directly derive the sum of the magnetic moments per molecule, since the coupling between the magnetic ions is much smaller than the thermal energy at 300 K, and hence it plays no role. We find that this value is consistent with the presence of a Dy^{III} ion and a Cr^{III} ion in the cluster, which yields a theoretical value of 16.05 cm^3 K mol^{-1}, using $g_{Dy} = 4/3$ and $g_{Cr} = 2$. Upon decreasing the temperature the χT product drops moderately at intermediate temperatures, and decreases steeply at low temperatures. Such behavior can arise from antiferromagnetic interactions within the cluster and/or the depopulation of the Dy ligand-field states. Ac susceptibility measurements at 1.8 K $\leq T \leq$ 20 K with and without a dc

magnetic field revealed no out-of-phase (χ'') component excluding the possibility of slow magnetic relaxation and the presence of minor magnetic impurities of *e.g.* DyF_3.

Spin-Hamiltonian Model and Fits

To obtain quantitative information about magnetic exchange coupling and anisotropy in **3**, we have used a spin-Hamiltonian approach. While the Cr^{III} ion can be modeled by a spin S = 3/2, the magnetic behavior of the Dy^{III} ion is more difficult to describe because of the orbital contribution to its magnetic moment. According to Hund's rules, the ground-state multiplet of the Dy^{III} ion is J = 15/2 with the first excited multiplet separated by more than 4000 cm^{-1}. The ground-state multiplet is split into eight Kramers doublets extending over an energy range of typically several hundreds of cm^{-1}. The $\langle J_z \rangle$ of the ground-state doublet depends on the exact geometry and strength of the ligand field. In order to speed up calculations and avoid overparameterization, we have restricted the basis set describing the Dy^{III} ion to the lowest two Kramers doublets. Further, we have assumed collinear orientation of the Dy and Cr anisotropy axes, which is justified in case of weak magnetic coupling. The spin-Hamiltonian operating on the restricted magnetic configuration space is given by

$$\hat{H} = -j\hat{\mathbf{J}}_{\mathrm{Dy}} \cdot \hat{\mathbf{S}}_{\mathrm{Cr}} + \left\{ D_{\mathrm{Dy}}[\hat{J}^2_{\mathrm{Dy},z} - \frac{1}{3} J_{\mathrm{Dy}}(J_{\mathrm{Dy}}+1)] + d_{\mathrm{Dy}} \right\}$$
$$+ D_{\mathrm{Cr}}[\hat{S}^2_{\mathrm{Cr},z} - \frac{1}{3} S_{\mathrm{Cr}}(S_{\mathrm{Cr}}+1)] + \mu_{\mathrm{B}}(g_{\mathrm{Dy}}\hat{\mathbf{J}}_{\mathrm{Dy}} + g_{\mathrm{Cr}}\hat{\mathbf{S}}_{\mathrm{Cr}}) \cdot \mathbf{B} \quad (1).$$

Here, $\hat{\mathbf{J}}_{\mathrm{Dy}}$ and $\hat{\mathbf{S}}_{\mathrm{Cr}}$ denote the total and spin angular momentum operators of the Dy^{III} and Cr^{III} ions, respectively, with J_{Dy} = 15/2 and S_{Cr} = 3/2. The first term represents an isotropic exchange coupling, the second and third terms are uniaxial anisotropies, and the last term is the interaction with the applied magnetic field. In the Dy anisotropy term, the parameters D_{Dy} and d_{Dy} are used to generate an energy splitting δ between the ground state doublet and the first excited-state doublet while keeping the whole Dy anisotropy term traceless. This notation is of course valid in the restricted space only. The g-factors are taken to be isotropic and fixed to g_{Dy} = 4/3 (Landé g-factor) and g_{Cr} = 2. An isotropic Dy g-factor is

justified because in eq 1 the $\hat{\mathbf{J}}_{Dy}$ operator has the property of a 15/2 angular momentum, in contrast to an effective spin-1/2 model. However, the latter can be directly derived from the Hamiltonian eq 1.[11] The best-fit parameters and uncertainties are determined as

j = $-0.04(3)$ cm^{-1}

D_{Cr} = $-1.7(1.0)$ cm^{-1}

δ = 57(21) cm^{-1}

$m_{J,GS}$ = ±13/2

$m_{J,ES}$ = ±11/2

and the corresponding calculated element-specific and cluster magnetization as well as the χT product are plotted as solid lines in Figs. 4 and 5. During the fitting process we have observed that there is a strong preference for the $m_{J,GS}$ = ±13/2 ground-state doublet of the Dy^{III} ion. Regarding the first-excited state, the fits are slightly better when using $m_{J,ES}$ = ±11/2, but $m_{J,ES}$ = ±15/2 yields an almost equally good result. We have tried a biaxial anisotropy term for the Cr^{III} ion, however, it did not improve the fits and resulted in the same D_{Cr} value. In view of the small magnetic coupling j we have tested whether our model would also be consistent with pure Dy-Cr dipolar coupling. For this we have replaced the isotropic exchange coupling in eq 1 by an anisotropic coupling matrix with ($2j_{dip}$, $-j_{dip}$, $-j_{dip}$) on its diagonal and zeros otherwise. The new fit result was j_{dip} = 0.043 cm^{-1}, in good agreement with the calculated value of $j_{dip,calc}$ = 0.029 cm^{-1} for **3**, using the Dy···Cr spatial separation of $r_{Dy\text{-}Cr}$ = 3.42 Å from the structural analysis and the isotropic g-factors given before and assuming a collinear orientation of the Dy and Cr easy magnetization axes. For comparison, the closest inter-cluster metal ion distances are approximately 9 Å, meaning that inter-cluster dipolar interactions are negligible. The other fit parameters turned out to be identical to the case of isotropic j. Details of the calculation are given in the Experimental Section.

Discussion

As mentioned in the Introduction, it is important to characterize 3d-4f exchange coupling for a systematic approach to the design of 3d-4f clusters. An accurate characterization solely based on SQUID measurements is likely to fail for clusters containing 4f ions with orbital contribution to the magnetic moment. XMCD has the power to resolve element-specific magnetization curves, which we have used here to overcome the above mentioned difficulties in the understanding of the magnetism of 3d-4f clusters. In the following we will give qualitative arguments based on the XMCD data why the Dy–Cr magnetic coupling in **3** can only be very small. The magnetic moment of the Cr^{III} ion is smaller than the one of Dy^{III}, hence in the case of a hypothetical antiferromagnetic coupling and at small fields compared to the coupling the Dy moment would be parallel and the Cr moment antiparallel to the magnetic field. In contrast, at large enough fields this coupling would break up, and both moments would be parallel to the field, leading to a wiggle shape in the Cr magnetization curve as seen for example in ref 11b. Since this wiggle shape is absent in **3**, a conservative estimate of a lower bound for the hypothetical antiferromagnetic Dy–Cr coupling $j \geq -\mu_B g_{Cr} B_0 / J_{Dy,z} = -0.13$ cm^{-1} can be obtained, with B_0 the field at which the Cr magnetic moment would flip its sign. Since no such wiggle shape is observed in the experiment, we have used a B_0 of 0.15 T. On the other hand, a significant ferromagnetic coupling is inconsistent with the data, too, since then the Cr magnetization curve close to zero field would be steeper than the paramagnetic one, the latter being indicated by the dashed line in Figure 4a. These qualitative arguments advocate that the absolute value of the magnetic coupling j is close to zero in agreement with the fitting results. As observed in the fits, simple dipolar coupling between Dy^{III} and Cr^{III} ions can fully account for the observed magnetic interaction, and its strength is consistent with the calculated values from the Dy–Cr spatial separation. Hence, there is essentially no magnetic exchange coupling mediated by the methoxide bridges. In the reported examples of Cr^{III}–Dy^{III} clusters or extended networks, the Cr–Dy exchange interaction appears to be weak and antiferromagnetic irrespective of the bridging ligand being fluoride, hydroxide or cyanide.[22] Even though so far there is no report on alkoxide-bridged Cr(III)-lanthanide clusters, our studies suggest that such bridges do not lead to an

exception to these findings. The fitting result of the Cr^{III} uniaxial anisotropy $D_{Cr} = -1.7(1.0)$ cm^{-1} appears large, but in view of the experimental uncertainty a value of $D_{Cr} \sim -1.0$ cm^{-1}, very reasonable for Cr^{III}, is also compatible with the data. The obtained separation between ground-state doublet and first-excited doublet of $\delta = 57(21)$ cm^{-1} is in excellent agreement with values reported in the literature.[23] Also, the m_J values of ±13/2 and ±11/2 for the ground-state doublet and the first-excited state, respectively, are in very good agreement with these studies. Regarding the comparison of the magnetic moments obtained by SQUID magnetometry and XMCD, despite an excellent overall agreement we observe a slight deviation of the cluster magnetic moment from the sum of the element-specific magnetic moments on the order of a few percent as visible in Figure 4. This deviation may be due to the fact that a SQUID magnetometer probes the entire bulk magnetic moment whereas XMCD only detects the targeted elemental magnetic moments, and even after taking into account diamagnetic contributions, ligand spin polarization (as, *e.g.*, seen in ref 11a) may give rise to the observed deviations between SQUID and XMCD measurements. Restricting the Hilbert space of the Dy^{III} ion to two Kramers doublets has the advantage that the whole ligand-field is reduced to three parameters, which are the m_J values of the ground state and excited state and the separation δ. This avoids overparameterization, however, it implies that the model can only be used for low enough temperatures at which all other Kramers doublets can be neglected. This becomes obvious in Figure 5: Clearly, there is perfect agreement at the lowest temperatures, however, at elevated temperatures $T > 20$ K the model curve lies below the measurement. The influence of temperature can be rationalized using a simple two-state model with an energy separation of 60 cm^{-1} assuming a Boltzmann distribution of populations. Exemplarily, at 2 K the population of the excited state amounts to a negligible 1.7×10^{-19}, whereas at 20 K it is 0.013, and similar populations can be expected if further excited states close in energy were added. This leads to deviations in the few-percent range which is indeed observed in the experimental data in Figure 5. To improve the model, other Kramers doublets could be added, however, such a model would again be prone to overparameterization.

Conclusions

We have synthesized a novel methoxide-bridged Cr^{III}–Dy^{III} dimer and performed a detailed magnetic study. The element-specific and cluster magnetization curves obtained from XMCD and SQUID magnetometry as well as the dc magnetic susceptibility were fitted using a spin-Hamiltonian model yielding excellent agreement with the data. The Dy^{III} and Cr^{III} ions are magnetically almost isolated from each other with an exchange coupling of $j = -0.04(3)$ cm^{-1}. Further analysis reveals that our observations are consistent with the presence of purely dipolar coupling between Dy^{III} and Cr^{III} ions suggesting that the double-methoxide bridge does not play any role in mediating exchange coupling. Furthermore, we determine the ground-state doublet of the Dy^{III} ion to be $m_{J,\mathrm{GS}} = \pm 13/2$ and the first-excited doublet is $m_{J,\mathrm{ES}} = \pm 11/2$ with an energy separation of $\delta = 57(21)$ cm^{-1}. The Cr^{III} ion exhibits a uniaxial anisotropy $D_{\mathrm{Cr}} = -1.7(1.0)$ cm^{-1}.

Acknowledgements

We acknowledge M. Schmidt for technical support. Part of this work was performed at the X-Treme beamline of the Swiss Light Source, Paul Scherrer Institut, Villigen, Switzerland. We gratefully acknowledge financial support for the XMCD endstation from Ecole Polytechnique Fédérale de Lausanne and from the Swiss National Science Foundation.

References

[1] a) Kahn, O. in *Molecular Magnetism*, Wiley-VCH, Weinheim, Germany, **1993**; b) Gatteschi, D.; Sessoli, R.; Villain, J. in *Molecular Nanomagnets*, Oxford University Press, **2006**.

[2] a) Leuenberger, M. N.; Loss, D. *Nature* **2001**, *410*, 789-793; b) Bogani, L.; Wernsdorfer, W. *Nat. Mater.* **2008**, *7*, 179–186.

[3] a) Winpenny, R. E. P. *Chem. Soc. Rev.* **1998**, *27*, 447-452; b) Benelli, C.; Gatteschi, D. *Chem. Rev.* **2002**, *102*, 2369-2388; c) Sessoli, R.; Powell, A. K. *Coord. Chem. Rev.* **2009**, *253*, 2328-2341; d) Andruh, M.; Costes, J.-P.; Diaz, C.; Gao, S. *Inorg. Chem.* **2009**, *48*, 3342-3359.

[4] a) Osa, S.; Kido, T.; Matsumoto, N.; Re, N.; Pochaba, A.; Mrozinski, J. *J. Am. Chem. Soc*. **2004**, *126*, 420-421; b) Zaleski, C. M.; Depperman, E. C.; Kampf, J. W.; Kirk, M. L.; Pecoraro, V. L. *Angew. Chem. Int. Ed.* **2004**, *43*, 3912-3914; c) Mishra, A.; Wernsdorfer, W.; Abboud, K. A.; Christou, G. *J. Am. Chem. Soc*. **2004**, *126*, 15648-15649; d) Mori, F.; Ishida, T.; Nogami, T. *Polyhedron* **2005**, *24*, 2588-2592; e) Costes, J.-P.; Dahan, F.; Wernsdorfer, W. *Inorg. Chem*. **2006**, *45*, 5-7; f) Aronica, C.; Pilet, G.; Chastanet, G.; Wernsdorfer, W.; Jacquot, J.-F.; Luneau, D. *Angew. Chem. Int. Ed.* **2006**, *45*, 4659-4662; g) Pointillart, F.; Bernot, K.; Sessoli, R.; Gatteschi, D. *Chem. Eur. J.* **2007**, *13*, 1602-1609; h) Mereacre, V.; Ako, A. M.; Clérac, R.; Wernsdorfer, W.; Hewitt, I. J.; Anson, C. E.; Powell, A. K. *Chem. Eur. J.* **2008**, *14*, 3577-3584; i) D. Schray, G. Abbas, Y. Lan, V. Mereacre, A. Sundt, J. Dreiser, O. Waldmann, G. Kostakis, C. Anson, A. Powell, *Angew. Chem. Int. Ed.* **2010**, *49*, 5185-5188; j) M. Holynska, D. Premuzic, I.-R. Jeon, W. Wernsdorfer, R. Clerac, S. Dehnen, *Chem. Eur. J.* **2011**, *17*, 9605-9610;

[5] a) Klokishner, S. I.; Ostrovsky, S. M.; Reu, O. S.; Palii, A. V.; Tregenna-Piggott, P. L. W.; Brock-Nannestad, T.; Bendix, J.; Mutka, H. *J. Phys. Chem. C* **2009**, *113*, 8573-8582; b) Ungur, L.; Chibotaru, L. F. *Phys. Chem. Chem. Phys.* **2011**, *13*, 20086-20090.

[6] a) Bünzli, J. C. G.; Piguet, C. *Chem. Rev.* **2002**, *102*, 1897–1928; b) Rinehart, J. D.; Long, J. R. *Chem. Sci.* **2011**, *2*, 2078-2085; c) Car, P.-E.; Perfetti, M.; Mannini, M.; Favre, A.; Caneschi, A.; Sessoli, R. *Chem. Commun.* **2011**, *47*, 3751-3753; d) Cucinotta, G.; Perfetti, M.; Luzon, J.; Etienne, M.; Car, P.-E.; Caneschi, A.; Calvez, G.; Bernot, K.; Sessoli, R. *Angew. Chem. Int. Ed.* **2012***, 51,* 1606-1610.

[7] a) Kahn, M. L.; Sutter, J. P.; Golhen, S.; Guionneau, P.; Ouahab, L.; Kahn, O.; Chasseau, D. *J. Am. Chem. Soc.* **2000**, *122*, 3413-3421; b) Lukens, W. W.; Walter, M. D. *Inorg. Chem.* **2010**, *49*, 4458-4465.

[8] a) Figuerola, A.; Tangoulis, V.; Sanakis, Y. *Chem. Phys.* **2007**, *334*, 204-215; b) Okazawa, A.; Nogami, T.; Nojiri, H.; Ishida, T. *Inorg. Chem.* **2008**, *47*, 9763-9765; c) Sorace, L.; Sangregorio, C.; Figuerola, A.; Benelli, C.; Gatteschi, D. *Chem. Eur. J.* **2009**, *15*, 1377-1388.

[9] a) Güdel, H. U.; Furrer, A.; Blank, H. *Inorg. Chem.* **1990**, *29*, 4081-4084; b) Aebersold, M. A.; Güdel, H. U.; Hauser, A.; Furrer, A.; Blank, H.; Kahn, R. *Phys. Rev. B* **1993**, *48*, 12723.

[10] a) van der Laan, G.; Thole, B. T. *Phys. Rev. B* **1991**, *43*, 13401-13411; b) Stöhr, J. *J. Magn. Magn. Mater.* **1999**, *200*, 470-497; c) Funk, T.; Deb, A.; George, S. J.; Wang, H.; Cramer, S. P. *Coord. Chem. Rev.* **2005**, *249*, 3-30.

[11] a) Hamamatsu, T.; Yabe, K.; Towatari, M.; Osa, S.; Matsumoto, N.; Re, N.; Pochaba, A.; Mrozinski, J.; Gallani, J.-L.; Barla, A.; Imperia, P.; Paulsen, C.; Kappler, J.-P. *Inorg. Chem.* **2007**, *46*, 4458-4468; b) Dreiser, J.; Pedersen, K. S.; Piamonteze, C.; Rusponi, S.; Salman, Z.; Ali, Md. E.; Schau-Magnussen, M.; Thuesen, C. Aa.; Piligkos, S. *et al. Chem. Sci.* **2012**, *3*, 1024-1032.

[12] a) Paulovic, J.; Cimpoesu, F.; Ferbinteanu, M.; Hirao, K. *J. Am. Chem. Soc.* **2004**, *126*, 3321-3331; b) Rajaraman, G.; Totti, F.; Bencini, A.; Caneschi, A.; Sessoli, R.; Gatteschi, D. *Dalton Trans.*

2009, 3153-3161; c) Shimada, T.; Okazawa, A.; Kojima, N.; Yoshii, S.; Nojiri, H.; Ishida, T. *Inorg. Chem.* **2011**, *50*, 10555-10557.

[13] a) Evangelisti, M.; Brechin, E. K. *Dalton Trans*. **2010**, *39*, 4672-4676; b) Sessoli, R. *Angew. Chem. Int. Ed.* **2012**, *51*, 43-45; c) Evangelisti, M.; Roubeau, O.; Palacios, E.; Camón, A.; Hooper, T. N.; Brechin, E. K.; Alonso, J. J. *Angew. Chem. Int. Ed.* **2011**, *50*, 6606-6609; d) Sharples, J. W.; Zheng, Y.-Z.; Tuna, F.; McInnes, E. J. L. *Chem. Commun.* **2011**, *47*, 7650-7652; e) Zheng, Y.-Z.; Evangelisti, M.; Winpenny, R. E. P. *Angew. Chem. Int. Ed.* **2011**, *50*, 3692-3695.

[14] a) Birk, T.; Schau-Magnussen, M.; Weyhermüller, T.; Bendix, J. *Acta Cryst.* **2011**, *E67*, m1561-m1562; b) Birk, T.; Pedersen, K. S.; Thuesen, C. Aa.; Weyhermüller, T.; Schau-Magnussen, M.; Piligkos, S.; Weihe, H.; Mossin, S.; Evangelisti, M.; Bendix, J. *submitted*.

[15] a) Blagg, R. J.; Muryn, C. A.; McInnes, E. J. L.; Tuna, F.; Winpenny, R. E. P. *Angew. Chem. Int. Ed.* **2011**, 50, 6530-6533; b) Blagg, R. J.; Tuna, F.; McInnes, E. J. L.; Winpenny, R. E. P. *Chem. Commun*. **2011**, *47*, 10587–10589.

[16] Piamonteze, C.; Flechsig, U.; Rusponi, S.; Dreiser, J.; Heidler, J.; Schmidt, M.; Wetter, R.; Schmidt, T.; Pruchova, H.; Krempasky, J.; Quitmann, C.; Brune, H.; Nolting, F. *submitted*.

[17] Krempasky, J.; Flechsig, U.; Korhonen, T.; Zimoch, D.; Quitmann, C.; Nolting, F. *AIP Conf. Proc*. **2010**, *1234*, 705-708.

[18] Lebedev, V. I.; Laikov, D. N. *Dokl. Math.* **1999**, *59*, 477.

[19] a) Perdih, F.; Demsar, A.; Pevec, A.; Petricek, S.; Leban, I.; Giester, G.; Sieler, J.; Roesky, H. W. *Polyhedron* **2001**, *20*, 1967-1971; b) Pevec, A.; Mrak, M.; Demsar, A.; Petricek, S.; Roesky, H. W. *Polyhedron* **2003**, *22*, 575-579; c) McRobbie, A.; Sarwar, A. R.; Yeninas, S.; Nowell, H.; Baker, M. L.;

Allan, D.; Luban, M.; Muryn, C. A.; Pritchard, R. G.; Prozorov, R.; Timco, G.; Tuna, F.; Whitehead, G. F. S.; Winpenny, R. E. P. *Chem. Commun.* **2011**, *47*, 6251-6253.

[20] a) Murugesu, M.; Mishra, A.; Wernsdorfer, W.; Abboud, K. A.; Christou, G. *Polyhedron*, **2006**, *25*, 613-625; b) Lin, P.-H.; Korobkov, I.; Wernsdorfer, W.; Ungur, L.; Chibotaru, L. F.; Murugesu, M. *Eur. J. Inorg. Chem.* **2011**, 1535-1539; c) Shiga, T.; Hoshino, N.; Nakano, M.; Nojiri, H.; Oshio, H. *Inorg. Chim. Acta* **2008**, *361*, 4113-4117; d) Mishra, A.; Tasiopoulos, A. J.; Wernsdorfer, W.; Abboud, K. A.; Christou, G. *Inorg. Chem.* **2007**, *46*, 3105-3115; e) Shiga, T.; Onuki, T.; Matsumoto, T.; Nojiri, H.; Newton, G. N.; Hoshono, N.; Oshio, H. *Chem. Commun.* **2009**, 3568-3570; f) Mishra, A.; Wernsdorfer, W.; Abboud, K. A.; Christou, G. *J. Am. Chem. Soc.* **2004**, *126*, 15648-15649; g) Murugesu, M.; Mishra, A.; Wernsdorfer, W.; Abboud, K. A.; Christou, G. *Polyhedron* **2006**, *25*, 613–625.

[21] a) Thole, B. T.; Carra, P.; Sette, F.; van der Laan, G. *Phys. Rev. Lett.* **1992**, *68*, 1943-1946; b) Carra, P.; Thole, B. T.; Altarelli, M.; Wang, X. *Phys. Rev. Lett.* **1993**, *70*, 694-697; c) Chen, C. T.; Idzerda, Y. U.; Lin, H.-J.; Smith, N. V.; Meigs, G.; Chaban, E.; Ho, G. H.; Pellegrin, E.; Sette, F. *Phys. Rev. Lett.* **1995**, *75*, 152-155.

[22] a) Estrader, M.; Ribas, J.; Tangoulis, V.; Solans, X.; Font-Bardia, M:; Maestro, M.; Diaz, C. *Inorg. Chem.* **2006**, *45*, 8239-8250; b) Rinck, J.; Novitchi, G.; van den Heuvel, W.; Ungur, L.; Lan, Y., Wernsdorfer, W.; Anson, C. E.; Chibotaru, L. F.; Powell, A. K. *Angew. Chem., Int. Ed.* **2010**, *49*, 7583-7587.

[23] a) Ishikawa, N.; Sugita, M.; Okubo, T.; Tanaka, N.; Iino, T.; Kaizu, Y. *Inorg. Chem.* **2003**, *42*, 2440-2446; b) Luzon, J.; Bernot, K.; Hewitt, I. J.; Anson, C. E.; Powell, A. K.; Sessoli, R. *Phys. Rev. Lett.* **2008**, *100*, 247205;

PAPER 9

The First Coordination Compound with 5-methylisoxazole-3-carboxylat: Synthesis and Structural Characterization of $[Cu(L_2)(H_2O)]\cdot H_2O$

Torben Birk and Høgni Weihe

J. Chem. Crystallogr., 2009, 39(10), 766-771

J Chem Crystallogr (2009) 39:766–771
DOI 10.1007/s10870-009-9563-7

ORIGINAL PAPER

The First Coordination Compound with 5-methylisoxazole-3-carboxylate: Synthesis and Structural Characterization of $[Cu(L_2)(H_2O)] \cdot H_2O$

Torben Birk · Høgni Weihe

Received: 6 May 2008 / Accepted: 23 March 2009 / Published online: 5 April 2009
© Springer Science+Business Media, LLC 2009

Abstract The title compound, aquabis(5-methylisoazole-3-carboxylato-*O*,*N*)copper(II) monohydrate, $[Cu(L_2)(H_2O)] \cdot H_2O$ is synthesized and characterized by X-ray diffraction, elementary analysis, and IR. The ligand coordinates to the copper center in a bidentate *O*,*N* fashion through the carboxylic acid moiety and the heteroaromatic nitrogen atom. The complex crystallizes in the triclinic space group $\bar{P}1$ with unit cell parameters $a = 8.4080(12)$Å, $b = 8.810(3)$, $c = 10.318(2)$Å, $\alpha = 110.08(3)°$, $\beta = 104.81(2)°$, $\gamma = 103.149(16)°$, $V = 650.9(3)$Å^3, Z = 2. In addition, the synthesis of the free acid 5-methylisoazole-3-carboxylic acid and pyridine containing complex bis(5-methylisoazole-3-carboxylato)bispyridinecopper(II) monohydrate is described.

Keywords Aquabis(5-methylisoazole-3-carboxylato-*O*,*N*)copper(II) monohydrate · Crystal structure · Synthesis

Introduction

The isoxazole system finds wide applications in organic chemistry, due to its versatile chemical properties as being stable but, depending on experimental conditions and substitution pattern also easily cleavable [1]. Classically, the isoxazole system is synthesized by 1,3-dipolar cycloaddition of either hydroxylamine to a 1,3-dicarbonyl compound, or a nitrile oxide to an acetylene [2].

As an interesting curiosity, 5-methylisoxazole-3-carboxylic acid (Scheme 1) can be synthesized in a one-pot high-yield synthesis by reaction of 2,5-hexanedione with hot nitric acid. This method was first described by A. Angeli in 1891 [3]. In historical context, the acid occupies an important part in connection with establishment of the monoamine hypothesis for the pathology of depression. In connection with the search for alternative anti tuberculosis drugs after the discovery of pyridine-4-carbohydrazide (isoniazid) in the early 1950s, the hydrazide of 5-methylisoxazole-3-carboxylic acid and derivates were synthesized [4]. In spite of the fact that anti tuberculosis properties were absent, the benzyl hydrazide derivate (isocarboxazid) was found to exhibit effective suppression of depression [5]. Simultaneously, it was found that the compound was a potent inhibitor of the monoamine oxidase enzyme. As a result of these two observations the monoamine hypothesis was introduced, explaining the connection between the general emotional mood and the concentration of neuron transmitter, e.g., serotonin, noradrenalin [6].

Besides its application in medical chemistry, 5-methylisoxazole-3-carboxylic acid has potential application from a coordination chemistry point of view because of its ability of acting as a multidentate ligand due to the content of carboxylic acid moiety and the isoxazolic nitrogen atom.

Hitherto, no coordination compounds with this acid are known. However, a limited number of transition metal complexes with isoxazole derivates have been structurally characterized.

Pure isoxazole complexes are known in salts of Fe^{II} in $[Fe(isoxazole)_6]^{2+}$ which show spin-crossover magnetic behavior, and $[Fe_3O(OAc)_6(isoxazole)_3][ClO_4]$ [7]. Other simply substituted isoxazolic ligands are represented by the following coordination compounds:

T. Birk (✉) · H. Weihe
Department of Chemistry, University of Copenhagen, Universitetsparken 5, 2100 Copenhagen Ø, Denmark
e-mail: Birk@kiku.dk

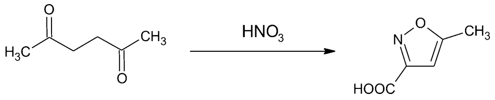

Scheme 1 Synthesis of 5-methylisoxazole-3-carboxylic acid

[Pt(5-methylisoxazole)$_2$(Cl)$_2$], [Pd(3,5-dimethyl-isoxazole)$_2$(Cl$_2$)], [Fe(3-amino-5-methylisoxazole)$_2$(H$_2$O)$_2$][ClO]$_2$, [Zn(3-amino-5-methylisoxazole)$_2$ · (Cl$_2$)] [8–11].

The isoxazole derivate of the antibacterial sulfanilamide, sulfamethoxazole (5-methyl-3-isoxazolyl sulfanilamide), has been the subject of some interest, due to the fact that coordination to various transition metal ions increases the pharmacological activity [12, 13]. Monodentate coordination of the isoxazole system is seen in [Zn(sulfamethoxazole)$_2$(py)$_2$(H$_2$O)$_2$] [14] and [Cu$_2$(sulfamethoxazole)$_2$ (μ-CH$_3$COO)$_4$], bidentate coordination in [Hg(sulfamethoxazole)$_2$] · 2DMSO [12] and catenation in [Ni(sulfamethoxazole)$_2$(H$_2$O)$_2$]$_\infty$ [15].

In this work the synthesis and characterization of the first coordination compound of 5-methylisoxazole-3-carboxylate is presented.

Experimental Section

Copper(II) nitrate-3-hydrate (Riedel-de-Haën), 2,5-hexanedione (Alfa Aesar, 97%), NaHCO$_3$ and Pyridine (Lab-Scan, HPLC) were all used as received.

Elementary analyses of C, H and N were performed with a CE Instruments Flash 112 series EA at the section for microanalysis, University of Copenhagen by Birgitta Kegel.

Infrared spectra were recorded in the frequency range 400–4,000 cm^{-1} with a Perkin-Elmer 2000 FTIR spectrophotometer by use of KBr technique.

The synthesis of 5-methylisoxazole-3-carboxylic acid is described in [3] and as patents [4, 16]. The method described herein follows the essence of the patent literature.

Synthesis of 5-Methylisoxazole-3-Carboxylic Acid

Nitric acid (0.2 L; 5.2 M), placed in a conical flask (1,000 mL) provided with a reflux condenser, is initially heated to the boiling point. Then the heating source is turned off and 2,5-hexanedione (45.751 g, 0.40 mol) is added through the reflux condenser. At the beginning with a speed of 2 drops/s until evolution of brown NO$_2$ is observed then with 1 drop/s. When steady reflux is obtained a gentle heat is switch on. After addition of the 2,6-hexanedione (~1 h) the heat is increased and the solution is boiled for at least 1½ h.

The light yellow solution is added to 200 g of crushed ice, and placed in ice/water bath for ½ h. The precipitated crystals are separated by filtration and washed with ice water (200 mL) and dried by air.

Calculated for H$_5$C$_5$N$_1$O$_3$: H 3.97%, C 47.25%; N 11.02%. Obtained: H 3.94%, C 47.45%; N 11.03%. Yield: 14.952 g (30% based on 2,5-hexanedione).

Synthesis of [Cu(L)$_2$(H$_2$O)] · H$_2$O

A solution of Cu(NO$_3$)$_2$ · 3H$_2$O (0,645 g) in water (20 mL) is added to a solution of 5-methylisoxazole-3-carboxylic acid (0,665 g; 5,23 mmol) and NaHCO$_3$ (0,440 g; 5,24 mmol) in water (10 mL). Slow evaporation resulted in large pillar shaped blue crystals.

Table 1 Crystallographic—and structure refinement data for title complex

Molecular formula	H$_{12}$C$_{10}$N$_2$O$_8$Cu$_1$
CCDC deposit no.	CCDC 685636
Molecular weight	352.76
T (K)	122(1)
Crystal system	Triclinic
Space group	$P-1$
a (Å)	8.4080 (12)
b (Å)	8.810 (3)
c (Å)	10.318 (2)
α (°)	110.08 (3)
β (°)	104.81 (2)
γ (°)	103.149 (16)
V (Å^3)	650.9 (3)
Z	2
F_{000}	358
D_{cal} (mg m^{-3})	1.795
Radiation type	Mo $K\alpha$
μ (mm^{-1})	1.72
Crystal size (mm)	0.6 × 0.16 × 0.15
Color, shape	Blue, Needle
θ range (°)	2.6–25.0
Absorb. correction T_{min}, T_{max}	0.565, 0.860
No. measured reflections	13,365
No. independent reflections (R_{int})	2,291
No. reflections with $I > 2\sigma(I)$	2,232
R_{int}	0.025
Number of ref. parameters	190
$R[F^2 > 2\sigma(F^2)]$	0.023
$wR(F^2)$	0.101
S (Goodness of fit)	0.97
$(\Delta/\sigma)_{max}$	0.001
$\Delta\rho_{min}$, $\Delta\rho_{max}$ (e Å^{-3})	0.52, −0.52

$w = 1/[\sigma^2(F_o^2) + (0.1P)^2]$ where $P = (F_o^2 + 2F_c^2)/3$

Calculated for $H_{12}C_{10}N_2O_8Cu_1$: H 3.44%, C 34.14%; N 7.96%. Obtained: H 3.34%, C 33.91%; N 7.94%.

Synthesis of $[Cu(L)_2(py)_2] \cdot H_2O$

Same method as with $[Cu(L)_2(H_2O)] \cdot H_2O$ but with substitution of $NaHCO_3$ with pyridine in excess.

Blue needle like crystals precipitates within 30 min. Isolated by filtration washed with water and ethanol and dried in air.

Calculated for $H_{20}C_{20}N_4O_7Cu_1$: H 4.10%, C 48.83%; N 11.39%. Obtained: H 4.09%, C 49.07%; N 11.37%.

Table 2 Fractional atomic coordinates and isotropic or equivalent isotropic displacement parameters (Å^2)

	x	*y*	*z*	U_{iso}*/U_{eq}
Cu1	0.57051 (2)	0.22445 (2)	0.985583 (19)	0.00960 (16)
C2	−0.1201 (3)	0.1491 (3)	0.7312 (2)	0.0178 (5)
H2A	−0.1850	0.1058	0.7851	0.021*
H2B	−0.1943	0.0980	0.6260	0.021*
H2C	−0.0866	0.2748	0.7717	0.021*
O3	0.95094 (17)	0.22238 (17)	1.11658 (15)	0.0120 (3)
C4	0.0825 (3)	−0.0278 (2)	0.6644 (2)	0.0113 (4)
H4	0.0101	−0.1207	0.5708	0.014*
C5	1.2546 (3)	0.2947 (3)	1.2631 (2)	0.0172 (4)
H5A	1.3116	0.3288	1.2007	0.021*
H5B	1.2274	0.1708	1.2340	0.021*
H5C	1.3335	0.3586	1.3672	0.021*
O6	0.54232 (17)	0.00238 (17)	0.83433 (15)	0.0132 (3)
O7	0.18074 (18)	0.21042 (17)	0.87527 (15)	0.0131 (3)
O8	0.58406 (18)	0.42524 (19)	1.15433 (16)	0.0114 (3)
O9	0.76662 (18)	0.63139 (18)	1.37812 (15)	0.0162 (3)
O10	0.65374 (16)	0.38474 (15)	0.87452 (14)	0.0159 (3)
H10A	0.6150	0.3174	0.7796	0.019*
H10B	0.5952	0.4614	0.8856	0.019*
O11	0.35531 (18)	−0.20847 (18)	0.61434 (15)	0.0145 (3)
N12	0.3153 (2)	0.1447 (2)	0.87141 (19)	0.0128 (4)
C14	0.8659 (2)	0.4183 (2)	1.2445 (2)	0.0101 (4)
N15	0.8111 (2)	0.27919 (19)	1.12174 (17)	0.0103 (4)
C16	1.0905 (2)	0.3352 (2)	1.2449 (2)	0.0121 (4)
C17	0.3938 (2)	−0.0771 (2)	0.7264 (2)	0.0114 (4)
C18	0.0400 (3)	0.1011 (3)	0.7471 (2)	0.0126 (4)
C19	0.2583 (2)	0.0064 (2)	0.7480 (2)	0.0101 (4)
C20	0.7295 (2)	0.5016 (2)	1.2647 (2)	0.0107 (4)
C21	1.0431 (3)	0.4589 (3)	1.3283 (2)	0.0126 (4)
H21	1.1136	0.5527	1.4218	0.015*
O25	0.53353 (19)	−0.19086 (19)	0.41754 (16)	0.0226 (4)
H25A	0.6064	−0.2493	0.4222	0.027*
H25B	0.4828	−0.2211	0.4610	0.027*

* The isotropic displacement parameters

X-ray Crystallographic Analysis

Single crystal data were obtained for the title compound at 122(1) K by use of a Nonius KappaCCD area-detector diffractometer (graphite-monochromated Mo-Kα radiation). The program COLLECT [17] was used for data collection. Cell refinement was performed with EvalCCD [18]. The structure was solved by direct methods with SHELXS-97 and refined on F^2 against all reflections with SHELXL-97 [19]. Non-hydrogen atoms were refined anisotropically; all hydrogen atoms were identified in a difference Fourier map and subsequently introduced as a riding model in the refinement.

Crystallographic data and refinement data for the title compound are given in Tables 1, 2 and 4.

Further information about the Crystallographic analysis is deposited in the "Cambridge Crystallographic Data Center" with registration number CCDC 685636. These data can be obtained free of charge via www.ccdc.cam.ac.uk/data_request/cif, by e-mailing data request@ccdc.cam.ac.uk, or by contacting The Cambridge Crystallographic Data Centre, 12 Union Road, Cambridge CB2 1EZ, UK; fax +44(0)1223-336033.

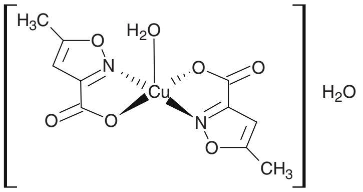

Fig. 1 Aquabis(5-methylisoazole-3-carboxylato-*O*,*N*)copper(II) monohydrate

Table 3 Selected bond lengths (Å) and angles (°)

Bond	
Cu1–O6	1.9553(16)
Cu1–O8	1.9662(17)
Cu1–N12	1.9847(19)
Cu1–N15	1.9840(17)
Cu1–O10	2.2064(13)
Angles	
O6–Cu1–O8	170.44(5)
O6–Cu1–N12	80.60(7)
O8–Cu1–N12	97.84(7)
O6–Cu1–N15	98.83(6)
O8–Cu1–N15	80.84(7)
N12–Cu1–N15	168.75(7)
O6–Cu1–O10	97.41(6)
N12–Cu1–O10	96.10(6)

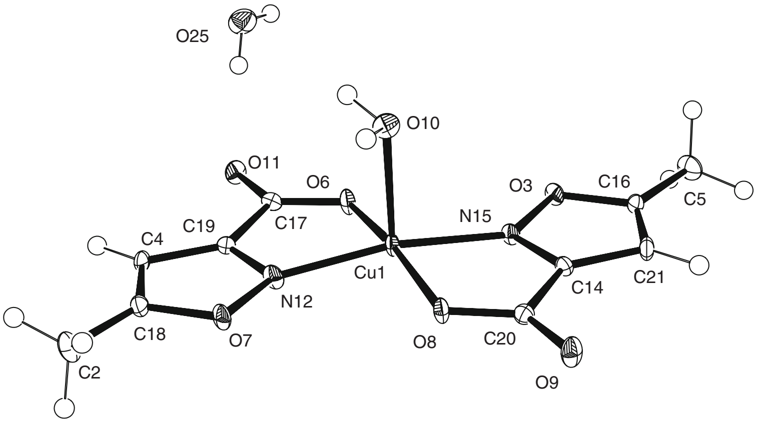

Fig. 2 The molecular structure and atom labeling scheme of aquabis(5-methylisoazole-3-carboxylato-*O*,*N*)copper(II) monohydrate. Displacement ellipsoids are drawn at 50% probability. H atoms with arbitrary radii

Results and Discussion

The molecular structure of the title compound $[Cu(L)_2(H_2O)] \cdot H_2O$ is shown in Fig. 1. A selection of bond distances and bond angles are given in Table 3.

The coordination polyhedron around the five coordinated central Cu^{II} ion is formed by two equatorial 5-methylisoxazole-3-carboxylates in an *O*,*N* bidentate fashion through the isoxazolic donor nitrogen and the carboxylic oxygen ligator atoms and one axial water ligand.

The coordination polyhedron is described as a square pyramid rather than a trigonal pyramid, due to the angles between two equatorial ligands e.g., O6–Cu–N12: 80.60(7)° and O8–Cu–N12: 97.84(7)° and angels between equatorial and apical ligands: O10–Cu–O6: 97.41(6)° and O10–Cu–N12: 96.10(6)° Fig. 2. The square pyramid geometry gives opportunity for yet another axial

Table 4 Atomic displacement parameters (Å^2)

	U^{11}	U^{22}	U^{33}	U^{12}	U^{13}	U^{23}
Cu1	0.0063 (2)	0.0088 (2)	0.0083 (2)	0.00279 (14)	0.00006 (14)	−0.00063 (14)
C2	0.0113 (10)	0.0177 (10)	0.0219 (11)	0.0065 (8)	0.0041 (9)	0.0062 (9)
O3	0.0080 (6)	0.0156 (7)	0.0144 (7)	0.0077 (5)	0.0039 (5)	0.0066 (6)
C4	0.0085 (9)	0.0109 (9)	0.0080 (9)	−0.0011 (8)	−0.0003 (8)	0.0022 (8)
C5	0.0123 (10)	0.0224 (10)	0.0229 (11)	0.0105 (8)	0.0073 (8)	0.0127 (9)
O6	0.0075 (7)	0.0123 (6)	0.0124 (7)	0.0032 (5)	−0.0001 (5)	−0.0001 (5)
O7	0.0081 (6)	0.0154 (7)	0.0142 (7)	0.0072 (5)	0.0026 (5)	0.0036 (5)
O8	0.0068 (7)	0.0111 (7)	0.0108 (7)	0.0034 (5)	0.0000 (5)	0.0005 (5)
O9	0.0133 (7)	0.0133 (7)	0.0133 (7)	0.0054 (6)	0.0014 (6)	−0.0021 (6)
O10	0.0188 (7)	0.0144 (6)	0.0155 (7)	0.0077 (5)	0.0076 (5)	0.0053 (5)
O11	0.0146 (7)	0.0109 (7)	0.0107 (7)	0.0030 (5)	0.0033 (5)	−0.0016 (6)
N12	0.0097 (8)	0.0125 (8)	0.0144 (9)	0.0065 (7)	0.0050 (7)	0.0016 (7)
C14	0.0076 (9)	0.0120 (9)	0.0111 (9)	0.0037 (7)	0.0033 (7)	0.0053 (7)
N15	0.0085 (8)	0.0107 (8)	0.0115 (8)	0.0056 (6)	0.0039 (6)	0.0028 (7)
C16	0.0086 (9)	0.0161 (9)	0.0101 (9)	0.0017 (7)	0.0012 (7)	0.0074 (7)
C17	0.0109 (9)	0.0119 (9)	0.0129 (10)	0.0041 (7)	0.0060 (7)	0.0057 (8)
C18	0.0086 (9)	0.0149 (10)	0.0125 (9)	0.0011 (8)	0.0018 (7)	0.0071 (8)
C19	0.0093 (9)	0.0116 (9)	0.0098 (9)	0.0027 (7)	0.0036 (7)	0.0053 (7)
C20	0.0103 (9)	0.0111 (9)	0.0106 (9)	0.0040 (7)	0.0039 (7)	0.0044 (8)
C21	0.0084 (9)	0.0132 (10)	0.0103 (9)	−0.0004 (8)	−0.0015 (7)	0.0045 (8)
O25	0.0251 (8)	0.0293 (8)	0.0193 (7)	0.0155 (7)	0.0121 (6)	0.0102 (6)

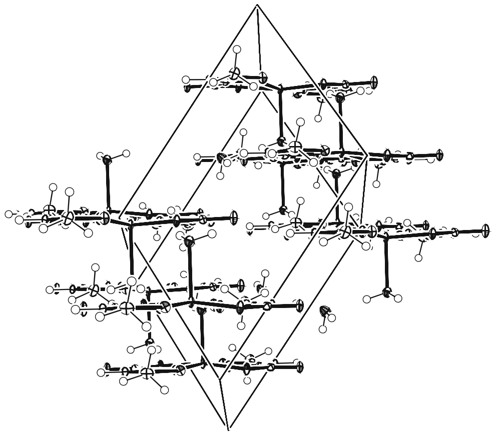

Fig. 3 The crystal packing in aquabis(5-methylisoazole-3-carboxylato-*O*,*N*)copper(II) monohydrat. Displacement ellipsoids are drawn at 50% probability

coordination site. This is not seen in the title compound, but could be found in the analogous pyridine complex $[Cu(L)_2(py)_2] \cdot H_2O$.

The bond between Cu^{II} and the axial water ligand is quite elongated 2.2064(13) Å in comparison to the equatorial Cu^{II} to ligand distances (average Cu–$N_{Isoxazole}$: 1.9844 Å and Cu–$O_{Carboxylate}$: 1.9608 Å) and also quite long compared to equivalent distances 2.051(3) Å and 2.071(3) Å in $[Cu(sulfisoxazole)_2(H_2O)_4] \cdot 2H_2O$ [20]. This elongation interprets with a Jahn–Teller distortion in the system (Table 4).

The bond distances between Cu^{II} and the carboxylate oxygen (Cu–$O_{Carboxylate}$: 1.9553(16) Å and 1.9662(17) Å) match the copper acetate distances in the binuclear complexes of the general type $[Cu_2(\mu\text{-}CH_3COO)_4(L)_2]$. For L ≡ sulfamethoxazole (1.963(3)Å–1.991(3)Å) and 3-amino-5-methylisoxazole (1.959(4)Å–1.974(4)Å) [12], [21].

The crystal packing of the title compound, Fig. 3, shows a parallel packing of the individual molecular units.

Infrared spectra of the title compound and the free acid are shown in Fig. 4. The infrared spectrum of 5-methylisoxazole-3-carboxylic acid consists of vibrations from the 3,5-disubstituted isoxazole ring by it self, the methyl group and carboxylic acid moiety. Vibrations originating from the 3,5-disubstituted isoxazole ring were assigned by comparison with the known fundamental IR vibrations for isoxazole and 5-methylisoxazole [1].

The O–H stretching vibration originating from the carboxylic acid group, dominates the spectrum with a broad band at 3,200–2,600 cm^{-1}. The C=O and C–O stretching vibrations and the out-of-plan bending is seen at

Fig. 4 IR spectra of 5-methylisoxazole-3-carboxylic acid and title complex. Bands emphasized in the text are indicated with an arrow

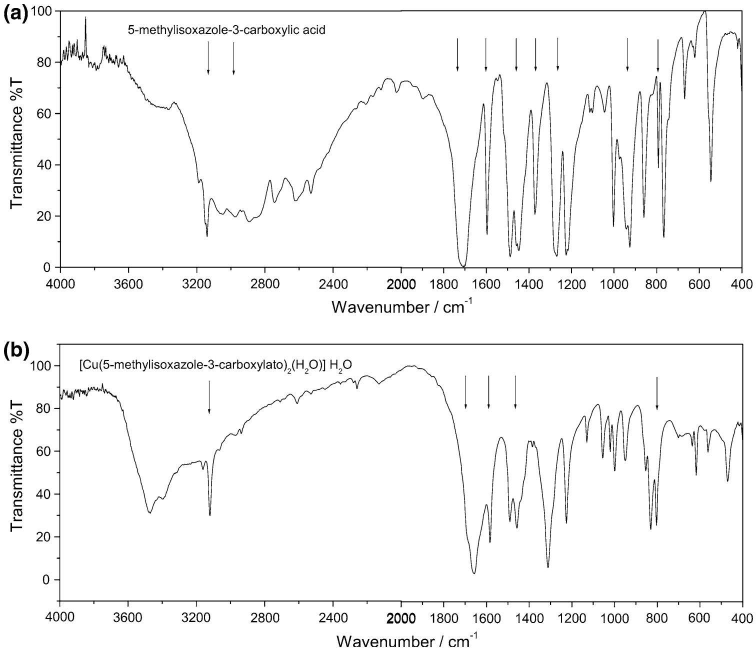

1,708 cm^{-1}, 1,269 cm^{-1} and 926 cm^{-1}, respectively. By coordination, the frequency of the C=O stretching is lowered to 1,656 cm^{-1}.

The methyl group at the isoxazole ring is seen in the infrared spectra by its anti symmetric bending vibration 1,371 cm^{-1}. The symmetric bending vibration of $-CH_3$ at 1,446 cm^{-1} is overlapped with the C=C stretching vibration from the isoxazole ring.

The isoxazole ring system gives rice to a C4–H stretching vibration at 3,139 cm^{-1}, a C=N stretching vibration at 1,596 cm^{-1} and a C4–H out-of-plane bending vibration at 792 cm^{-1}. By coordination, the bands are shifted to 3,120 cm^{-1}, 1,583 cm^{-1} and 803 cm^{-1}, respectively.

Acknowledgments The authors are grateful to Mr. Flemming Hansen (Centre of Crystallographic Studies, University of Copenhagen) for collection of the X-ray diffraction data.

References

1. Taylor EC, Weissberger A (eds) (1991) The chemistry of heterocyclic compounds: isoxazoles. Wiley-Interscience: New York, Part I, vol 49, pp 1–416
2. Hansen TV, Wu P, Fokin VV (2005) J Org Chem 70:7761. doi:10.1021/jo050163b
3. Angeli A (1891) Chem Ber 24:1305
4. Gardner TS, Lee J, Wenis E (Hoffmann-La Roche Inc.) (1959) Patent US 2,908,688 19,591,013
5. Gardner TS, Wenis E, Lee J (Hoffmann La-Roche Inc.) (1960) J Med Pharm. Chem. 2:133 doi:10.1021/jm50009a002
6. Stephen M Stahl (2001) Essential psychopharmacology of depression and bipolas disorder. Cambridge University Press, pp 20
7. Hibbs W, van Koningsbruggen PJ, Arif AM, Shum WW, Miller JS (2003) Inorg Chem 42:5645. doi:10.1021/ic034226p
8. Horn E, Horiuchi A, Yamanaka M, Murakami M, Horiuchi CA (2005) Z Kristallogr New Cryst Struct 220:239
9. Horiuchi A, Horn E, Ito K, Nakahodo T, Watabe M, Takahashi TT, Houriuchi CA (2005) Z Kristallogr New Cryst Struct 220:27
10. Baran Y, Linert W (2001) J Chem Crystallogr 31:369. doi:10.1023/A:1015651422973
11. Shen L, Li MC, Jin ZM, Hu ML, Xuan RC (2004) Acta Crystallogr Sect E Struct Rep Online 60:m330. doi:10.1107/S1600536804003939
12. de Lourenco L, Lang ES, Fenner H, Castellano EE (2005) Z Anorg Allg Chem 631:745 and references therein. doi:10.1002/zaac.200400443
13. Kremer E, Facchin G, Estéves E, Alborés P, Baran EJ, Ellena J, Torre MH (2006) J Inorg Biochem 100:1167. doi:10.1016/j.jinorgbio.2006.01.042
14. Garcia-Raso A, Fiol JJ, Rigo S, Lopez A, Molins E, Espinosa E, Borras E, Alzuet G, Borras J, Castineiras (2000) Polyhedron 19:991. doi:10.1016/S0277-5387(00)00355-7
15. Torre MH, Calvo S, Pardo H, Mombru AW (2005) J Coord Chem 58:513. doi:10.1080/00958970500037516
16. Niemczyk HJ (2003) Patent US 6,518,254 B1 20,030,211
17. Nonius (1999) COLLECT. Nonius BV, Delft, The Netherlands
18. Duisenberg AJM, Kroon-Batenburg LMJ, Schreurs AMM (2003) J Appl Cryst 36:220. doi:10.1107/S0021889802022628
19. Sheldrick GM (2008) Acta Crystallogr A 64:112. doi:10.1107/S0108767307043930
20. Kremer E, Facchin G, Estéves E, Alborés P, Baran EJ, Ellena J, Torre MH (2006) J Inorg Biochem 100:1167. doi:10.1016/j.jinorgbio.2006.01.042
21. Garnovskii DA, Antsyshkina AS, Sadikov GG, Kurbatov VP, Yu. Eliseeva A, Vasilchenko IS, Garnovskii AD (2000) J Struct Chem 41:468. doi:10.1007/BF02742007

PAPER 10

Matrix Formulation of Complex Equilibria and Acid-Base Equilibria by use of the Extent of Reaction

Torben Birk

Chem. Educator, 2009, 14(3), 91-95.

Matrix Formulation of Complex Equilibria and Acid–Base Equilibria by Use of the Extent of Reaction

Torben Birk

University of Copenhagen, Universitetsparken 5 DK-2100, Copenhagen, Birk@kiku.dk

Received December 10, 2008 Accepted May 20, 2009.

Abstract: Equilibria considerations are an integrated part of all higher chemistry education. This article deals with a compact formalism to treat complicated equilibrium systems of, for example, acid–base and complex equilibria through a matrix formulation based on the extent of reaction.

Introduction

The aim of equilibrium calculations falls into two parts. One concerns extraction of information about the equilibrium constants from experimental data. The other regards determination of the composition of a given system at equilibrium based on the knowledge of the composition at initial state combined with expressions from the law of mass action. Here we shall discuss only the latter subject.

Traditionally, equilibrium calculations have been approached by consideration of the equations of mass and charge preservation for the system [1]. Even for simple systems, exact calculations of this kind become extremely tedious and calculation by hand is often impractical. Accordingly, more complex equilibrium systems are often considered by approximate methods or graphical methods such as logarithmic concentration diagrams [2].

In many chemistry classrooms, a number of students ask “how to do it right.” Depending on the students level within mathematics and chemical maturity the teacher can choose to introduce either the traditional description or a more advanced method described within this paper. This method builds on the students “normal” way of treating simple equilibria problems, which often is conceptually understood as finding “how many times” the reaction must occur before equilibrium is reached. This “normal way” of treating equilibria is nothing but use of the extent of reaction, ξ.

The concept of extent of reactions introduced by Th. De Donder has widespread applications in physical chemistry (e.g., thermodynamic and kinetic reacrtions) to describe a system’s chemical transformations towards equilibrium [3]. Use of the concept is not limited to advanced chemistry but also its be use as a unifying basis for stoichiometry in elementary chemistry has been described [4].

Direct use of the extent of reaction in the context of equilibria calculations implies, at first, no advantage. Even simple systems at equilibrium result in a complicated set of coupled equations. However, many of the traditional equilibrium problems from coordination chemistry and acid–base chemistry can be conveniently expressed in matrix formalism by use of the extent of reaction. Introduction of a matrix formulation increases the overview and results in a more systematic approach when equilibrium systems with many components are treated. It should be emphasized, that the matrix formulation using the extent of reaction does not lead to less algebraic manipulation when compared to the traditional approach, but the transparency will be increased. Additionally, this method provides interested students with a concrete example where their knowledge from linear algebra can be applied in chemistry.

Here it will be shown how to derive exact and general equations for a system at equilibrium (e.g., complex ion equilibria and acid–base equilibria) and how these equations, with suitable conditions, can be transformed so that exact calculations can be performed transparently by “hand”.

Matrix Formulation of Simple Equilibria

For a chemical system containing N different reaction entities $\{B_1, B_2, B_3, B_4, B_N\}$ entering M independent chemical reactions $\{\rho_1, \rho_2, \rho_3, \rho_4, \rho_M\}$ it can be shown (see appendix 1 **Error! Reference source not found.**) that the molar concentration of a reaction entity, $[B_j]$ (units mol·L^{-1}) is related to the extent of reaction $\hat{\xi}_{\rho_i}$ (units mol·L^{-1}) by

$$[B_j] = [B_j]_0 + \sum_{i=1}^{M} \upsilon_{B_j,\rho_i} \hat{\xi}_{\rho_i} \qquad (1)$$

where $[B_j]_0$ is the initial molar concentration of reaction entity B_j, and υ_{B_j,ρ_i} is the stoichiometric coefficient by which reaction entity B_j enters reaction ρ_i.

Equation 1 is valid and $[B_j \in \{B_1, B_2,..., B_N\}$ can be expressed as a matrix equation:

$$\begin{bmatrix} [B_1] \\ [B_2] \\ \vdots \\ [B_N] \end{bmatrix}_{N\times 1} = \begin{bmatrix} [B_1]_0 \\ [B_2]_0 \\ \vdots \\ [B_N]_0 \end{bmatrix}_{N\times 1} + \begin{bmatrix} \upsilon_{B_1,\rho_1} & \upsilon_{B_1,\rho_2} & \cdots & \upsilon_{B_1,\rho_M} \\ \upsilon_{B_2,\rho_1} & \upsilon_{B_2,\rho_2} & \cdots & \upsilon_{B_2,\rho_M} \\ \vdots & \vdots & \ddots & \vdots \\ \upsilon_{B_N,\rho_1} & \upsilon_{B_N,\rho_2} & \cdots & \upsilon_{B_N,\rho_M} \end{bmatrix}_{N\times M} \begin{bmatrix} \hat{\xi}_{\rho_1} \\ \hat{\xi}_{\rho_2} \\ \vdots \\ \hat{\xi}_{\rho_M} \end{bmatrix}_{M\times 1}$$

$$\Rightarrow \underline{B} = \underline{B}_0 + \underline{\underline{\upsilon}} \cdot \underline{\hat{\xi}} \qquad (2)$$

where $\underline{B}$ and $\underline{B}_0$ are two column vectors of the concentration of all reaction entities in the final and initial state, respectively and $\underline{\underline{\upsilon}}$ is the stoichiometric matrix.

© 2009 *The Chemical Educator*, S1430-4171(09)32206-6, Published on Web 06/05/2009, 10.1333/s00897092206a, 14090091tb.pdf

Example 1.

Consider the following equilibria for the diprotic acid, H_2A reaction with water:

$$(1)\quad H_2A + H_2O \rightleftharpoons HA^- + H_3O^+ \quad K_{1a} = \frac{[HA^-][H_3O^+]}{[H_2A]}$$

$$(2)\quad HA^- + H_2O \rightleftharpoons A^{2-} + H_3O^+ \quad K_{2a} = \frac{[A^{2-}][H_3O^+]}{[HA^-]}$$

$$(3)\quad 2H_2O \rightleftharpoons H_3O^+ + OH^- \quad K_W = [H_3O^+][OH^-] \tag{3}$$

In its most general form, the system in its initial state has non-vanishing concentrations of all reaction entities: $[H_2A]_0$, $[HA^-]_0$, $[A^{2-}]_0$, and $[OH^-]_0]$, which are collected in the concentration vector $\underline{B}_0$. The composition of the system at equilibrium, $\underline{B}$, is given by eq 2 in combination with the stoichiometric coefficient obtained directly from 3.

$$\underline{B} = \underline{B}_0 + \underline{\underline{\upsilon}} \cdot \hat{\underline{\xi}} \;\Rightarrow\; \begin{bmatrix} [H_2A] \\ [HA^-] \\ [A^{2-}] \\ [OH^-] \\ [H_3O^+] \end{bmatrix} = \begin{bmatrix} [H_2A]_0 \\ [HA^-]_0 \\ [A^{2-}]_0 \\ [OH^-]_0 \\ [H_3O^+]_0 \end{bmatrix} + \begin{bmatrix} -1 & 0 & 0 \\ 1 & -1 & 0 \\ 0 & 1 & 0 \\ 0 & 0 & 1 \\ 1 & 1 & 1 \end{bmatrix} \begin{bmatrix} \hat{\xi}_1 \\ \hat{\xi}_2 \\ \hat{\xi}_3 \end{bmatrix} \tag{4}$$

How to obtain the stoichiometric matrix is exemplified by with the reaction entity HA^-. The equilibria in 3 show that HA^- takes part in reaction 1 as a product with stoichiometric coefficient 1, in reaction 2 as reactant with stoichiometric coefficient 1, and this entity does not enter reaction 3. By using the sign convention of stoichiometric coefficients being negative for reactants and positive for products we obtain the second the row [1 –1 0] in 4. With similar arguments we also obtain the remaning rows of 4.

Equation 2 states that if we know $\hat{\underline{\xi}}$ for the system, we are able to calculate the concentration of all reaction species from their initial concentrations at any state towards equilibrium. Here we are interested in the state of equilibrium, and this state is implied in the rest of the paper.

The state of equilibrium is determined by the law of mass action, which in terms of the stoichiometric equilibrium constant, K_i, for reaction ρ_i (see appendix 2) is

$$K_i \equiv \prod_{j=1}^{N} [B_j]^{\upsilon_{B_j,\rho_i}} \tag{5}$$

Combination of equation 1 with 5 gives the general equation for calculation of the set of extents of reaction at the state of equilibrium.

$$K_i = \prod_{j=1}^{N} \left\{ [B_j]_0 + \sum_{k=1}^{M} \upsilon_{\upsilon_{B_j,\rho_k}} \hat{\xi}_{\rho_k} \right\}^{\upsilon_{B_j,\rho_i}} \tag{6}$$

The set of M simultaneous coupled equations 6 together with 2 represents a complete formulation for a general, composite system at equilibrium, and contains all required information to specify the equilibrium state of the system. The set of equations 6 must be solved for the set

$$\left\{ \hat{\xi}_{\rho_1}, \hat{\xi}_{\rho_2}, \ldots, \hat{\xi}_{\rho_M} \right\}$$

which afterwards are inserted into 2 to give the concentration of any reaction entity present in the system.

The set of simultaneous coupled equations 6 is in general very complicated, but most equilibrium systems known from coordination chemistry and acid–base chemistry form a system of reactions that can be simplified and treated in terms of linear algebra.

Equilibria problems in these two areas of chemistry have traditionally been expressed in different ways. Inside the area of coordination chemistry, complex equilibria is commonly consider as a central metal ion, M^p with a consecutive inclusion of ligands, X^q to form the different complex ions, MX_n^{p+nq} (e.g., reaction of Cd^{2+} with Cl^- to form $[CdCl]^+$, $[CdCl_2]$, $[CdCl_3]^-$, and $[CdCl_4]^{2-}$). The opposite way of describing the equilibria, is normally adopted in the area of acid–base chemistry, were protolysis of a polyvalent acid, $H_{n-j}A^{q-j}$ is a common problem (e.g., dissociation of H_3PO_4 or $[Al(OH_2)_6]^{3+}$).

For these kinds of systems it is common practice to regard the ligand concentration, $[X^q]$ or hydronium concentration, $[H_3O^+]$ as a parameter in terms of which all other concentrations are expressed. In general, the set of N different reaction entities forming the chemical system can be regarded as a union of two sets, one with the reaction entities we wish to use as parameters for the description of the system and one with the rest. On the basis of this, we define a further concentration vector $\underline{B}^\circ$ in addition to the proper concentration vector $\underline{B}$ from 2. $\underline{B}^\circ$ is the reduced concentration column vector of dimension $N^\circ \times 1$ and contains the concentration of all reactions entities which are not used as parameters. For the diprotic acid considered in Example 1, $[H_3O^+]$ would be the parameter concentration whereas $[H_2A]$, $[HA^-]$, $[A^{2-}]$, and $[OH^-]$ are the elements of $\underline{B}^\circ$. The vector $\underline{B}$ is connected with the reduced $\underline{B}^\circ$ through a transformation matrix of dimension $N^\circ \times N$, named $\underline{\underline{\tau}}$:

$$\underline{B}^\circ_{N^\circ \times 1} = \underline{\underline{\tau}}_{N^\circ \times N} \cdot \underline{B}_{N \times 1} \tag{7}$$

Example 1 Continued.

The concentration, $[H_3O^+]$is used as a parameter implying the following reduced concentration vector $\underline{B}^\circ$ and transformation matrix for its relation to $\underline{B}$:

$$\underline{B}^\circ = \underline{\underline{\tau}} \cdot \underline{B} \;\Rightarrow\; \begin{bmatrix} [H_2A] \\ [HA^-] \\ [A^{2-}] \\ [OH^-] \end{bmatrix} = \begin{bmatrix} 1 & 0 & 0 & 0 & 0 \\ 0 & 1 & 0 & 0 & 0 \\ 0 & 0 & 1 & 0 & 0 \\ 0 & 0 & 0 & 1 & 0 \end{bmatrix} \begin{bmatrix} [H_2A] \\ [HA^-] \\ [A^{2-}] \\ [OH^-] \\ [H_3O^+] \end{bmatrix} \tag{8}$$

© 2009 *The Chemical Educator*, S1430-4171(09)32206-6, Published on Web 06/05/2009, 10.1333/s00897092206a, 14090091tb.pdf

We now make that assumption that the set of mass action equations 5 for the system containing N different reaction entities entering M independent chemical reactions can be expressed as a matrix product of the following form:

$$\underline{\underline{E}}_{M\times N^{\circ}} \cdot \underline{B}^{\circ}_{N^{\circ}\times 1} = \underline{\kappa}_{M\times 1} \qquad (9)$$

where $\underline{\underline{E}}$ is a equilibrium matrix of dimension $M \times N^{o}$ containing information about the equilibrium constants and parameter concentrations, $\underline{B}^{o}$ is the reduced concentration column vector and $\underline{\kappa}$ a constant vector.

Insertion of 7 and 2 into 9 gives an equation from which the column vector of extent of reaction, $\hat{\underline{\xi}}$ can be obtained as:

$$\underline{\kappa}_{M\times 1} = \underline{\underline{E}}_{M\times N^{\circ}} \cdot \underline{B}^{\circ}_{N^{\circ}\times 1} = \underline{\underline{E}}_{M\times N^{\circ}} \left(\underline{\underline{\tau}}_{N^{\circ}\times N} \underline{B}_{N\times 1}\right) = \underline{\underline{E}}_{M\times N^{\circ}} \underline{\underline{\tau}}_{N^{\circ}\times N} \left(\underline{B}_{0N\times 1} + \underline{\underline{\upsilon}}_{N\times M} \hat{\underline{\xi}}_{M\times 1}\right)$$
$$\Downarrow$$
$$\hat{\underline{\xi}}_{M\times 1} = \left(\underline{\underline{E}}_{M\times N^{\circ}} \underline{\underline{\tau}}_{N^{\circ}\times N} \underline{\underline{\upsilon}}_{N\times M}\right)^{-1} \left(\underline{\kappa}_{M\times 1} - \underline{\underline{E}}_{M\times N^{\circ}} \underline{\underline{\tau}}_{N^{\circ}\times N} \underline{B}_{0N\times 1}\right) \qquad (10)$$

From $\hat{\underline{\xi}}$ the column vector of concentrations, $\underline{B}$ is given from insertion of 10 into 2:

$$\underline{B}_{N\times 1} = \underline{B}_{0N\times 1} + \underline{\underline{\upsilon}}_{N\times M} \left(\underline{\underline{E}}_{M\times N^{\circ}} \underline{\underline{\tau}}_{N^{\circ}\times N} \underline{\underline{\upsilon}}_{N\times M}\right)^{-1} \left(\underline{\kappa}_{M\times 1} - \underline{\underline{E}}_{M\times N^{\circ}} \underline{\underline{\tau}}_{N^{\circ}\times N} \underline{B}_{0N\times 1}\right) \qquad (11)$$

Equation 10 and 11 provide us with a compact and complete solution to the problem of calculating system composition at equilibrium.

This derivation relies on the assumption that the law of mass action could be expressed as the matrix product 9, which is valid for most complexation equlibria as well as acid–base equlibria. How to obtain this is shown by the following.

The mass action equations from 3 are transformed in the following manner:

$$\left.\begin{array}{l} K_{1a} = \dfrac{[\mathrm{HA^-}][\mathrm{H^+}]}{[\mathrm{H_2A}]} \\ K_{2a} = \dfrac{[\mathrm{A^{2-}}][\mathrm{H^+}]}{[\mathrm{HA^-}]} \\ K_W = [\mathrm{H^+}][\mathrm{OH^-}] \end{array}\right\} \Rightarrow \begin{array}{c} K_{1a}[\mathrm{H_2A}] - [\mathrm{HA^-}][\mathrm{H^+}] = 0 \\ K_{2a}[\mathrm{HA^-}] - [\mathrm{A^{2-}}][\mathrm{H^+}] = 0 \\ [\mathrm{H^+}][\mathrm{OH^-}] = K_W \end{array} \qquad (12)$$

where $[\mathrm{H^+}] = [\mathrm{H_3O^+}]$.

By introduction of the reduced concentration vector $\underline{B}^{o}$ from 8 equation 12 can be expressed as the following matrix product:

$$\underline{\underline{E}} \cdot \underline{B}^{\circ} = \underline{\kappa} \ \Rightarrow \ \begin{bmatrix} K_{1a} & -[\mathrm{H^+}] & 0 & 0 \\ 0 & K_{2a} & -[\mathrm{H^+}] & 0 \\ 0 & 0 & 0 & [\mathrm{H^+}] \end{bmatrix} \begin{bmatrix} [\mathrm{H_2A}] \\ [\mathrm{HA^-}] \\ [\mathrm{A^{2-}}] \\ [\mathrm{OH^-}] \end{bmatrix} = \begin{bmatrix} 0 \\ 0 \\ K_W \end{bmatrix} \qquad (13)$$

Insertion of the matrices from 4 and 13 into 10 gives the matrix equation for the extent of reaction vector, $\hat{\underline{\xi}}$:

$$\hat{\underline{\xi}} = \left(\underline{\underline{E}} \cdot \underline{\underline{\tau}} \cdot \underline{\underline{\upsilon}}\right)^{-1} \left(\underline{\kappa} - \underline{\underline{E}} \cdot \underline{\underline{\tau}} \cdot \underline{B}_0\right)$$
$$\Downarrow$$
$$\begin{bmatrix} \hat{\xi}_1 \\ \hat{\xi}_2 \\ \hat{\xi}_3 \end{bmatrix} = \begin{bmatrix} -K_{1a} - [\mathrm{H^+}] & [\mathrm{H^+}] & 0 \\ K_{2a} & -K_{2a} - [\mathrm{H^+}] & 0 \\ 0 & 0 & [\mathrm{H^+}] \end{bmatrix}^{-1} \begin{bmatrix} -K_{1a}[\mathrm{H_2A}]_0 + [\mathrm{H^+}][\mathrm{HA^-}]_0 \\ -K_{2a}[\mathrm{HA^-}]_0 + [\mathrm{H^+}][\mathrm{A^{2-}}]_0 \\ K_W - [\mathrm{H^+}][\mathrm{OH^-}]_0 \end{bmatrix} \qquad (14)$$

The inverse matrix in 14 is a block matrix, obey

$$\begin{bmatrix} \underline{\underline{A}} & 0 \\ 0 & \underline{\underline{B}} \end{bmatrix}^{-1} = \begin{bmatrix} \underline{\underline{A}}^{-1} & 0 \\ 0 & \underline{\underline{B}}^{-1} \end{bmatrix}$$

and is given as

$$\begin{bmatrix} \hat{\xi}_1 \\ \hat{\xi}_2 \\ \hat{\xi}_3 \end{bmatrix} = \frac{1}{D} \begin{bmatrix} -K_{2a} - [\mathrm{H^+}] & -[\mathrm{H^+}] & 0 \\ -K_{2a} & -K_{1a} - [\mathrm{H^+}] & 0 \\ 0 & 0 & \dfrac{D}{[\mathrm{H^+}]} \end{bmatrix} \begin{bmatrix} -K_{1a}[\mathrm{H_2A}]_0 + [\mathrm{H^+}][\mathrm{HA^-}]_0 \\ -K_{2a}[\mathrm{HA^-}]_0 + [\mathrm{H^+}][\mathrm{A^{2-}}]_0 \\ K_W - [\mathrm{H^+}][\mathrm{OH^-}]_0 \end{bmatrix}$$
$$= \frac{1}{D} \begin{bmatrix} -\left([\mathrm{HA^-}]_0 + [\mathrm{A^{2-}}]_0\right)[\mathrm{H^+}]^2 + K_{1a}[\mathrm{H_2A}]_0[\mathrm{H^+}] + K_{1a}K_{2a}[\mathrm{H_2A}]_0 \\ -[\mathrm{A^{2-}}]_0[\mathrm{H^+}]^2 - K_{1a}[\mathrm{A^{2-}}]_0[\mathrm{H^+}] + K_{1a}K_{2a}\left([\mathrm{H_2A}]_0 + [\mathrm{HA^-}]_0\right) \\ \dfrac{D\left(K_W - [\mathrm{H^+}][\mathrm{OH^-}]_0\right)}{[\mathrm{H^+}]} \end{bmatrix} \qquad (15)$$

where $D \equiv [\mathrm{H^+}]^2 + K_{1a}K_{2a}$.

To simplify the following equations we assume that $[\mathrm{H_3O^+}]_0 = [\mathrm{OH^-}] = 0_{\mathrm{M}}$.

Equation 15 is inserted into 4 to give the final expression for all the unknown concentrations of all reaction entities in the system at equilibrium.

$$\begin{bmatrix} [\mathrm{H_2A}] \\ [\mathrm{HA^-}] \\ [\mathrm{A^{2-}}] \\ [\mathrm{OH^-}] \\ [\mathrm{H^+}] \end{bmatrix} = \begin{bmatrix} [\mathrm{H_2A}]_0 \\ [\mathrm{HA^-}]_0 \\ [\mathrm{A^{2-}}]_0 \\ 0 \\ 0 \end{bmatrix} + \frac{1}{D} \begin{bmatrix} -1 & 0 & 0 \\ 1 & -1 & 0 \\ 0 & 1 & 0 \\ 0 & 0 & 1 \\ 1 & 1 & 1 \end{bmatrix} \begin{bmatrix} -\left([\mathrm{HA^-}]_0 + [\mathrm{A^{2-}}]_0\right)[\mathrm{H^+}]^2 + K_{1a}[\mathrm{H_2A}]_0[\mathrm{H^+}] + K_{1a}K_{2a}[\mathrm{H_2A}]_0 \\ -[\mathrm{A^{2-}}]_0[\mathrm{H^+}]^2 - K_{1a}[\mathrm{A^{2-}}]_0[\mathrm{H^+}] + K_{1a}K_{2a}\left([\mathrm{H_2A}]_0 + [\mathrm{HA^-}]_0\right) \\ \dfrac{DK_W}{[\mathrm{H^+}]} \end{bmatrix} \qquad (16)$$

Performing the matrix multiplication results in

$$\begin{bmatrix} [\mathrm{H_2A}] \\ [\mathrm{HA^-}] \\ [\mathrm{A^{2-}}] \\ [\mathrm{OH^-}] \\ [\mathrm{H_3O^+}] \end{bmatrix} = \frac{1}{D} \begin{bmatrix} \left([\mathrm{H_2A}]_0 + [\mathrm{HA^-}]_0 + [\mathrm{A^{2-}}]_0\right)[\mathrm{H^+}]^2 \\ K_{1a}\left([\mathrm{H_2A}]_0 + [\mathrm{HA^-}]_0 + [\mathrm{A^{2-}}]_0\right)[\mathrm{H^+}] \\ K_{1a}K_{2a}\left([\mathrm{H_2A}]_0 + [\mathrm{HA^-}]_0 + [\mathrm{A^{2-}}]_0\right) \\ \dfrac{DK_W}{[\mathrm{H^+}]} \\ \dfrac{1}{[\mathrm{H^+}]} \left\{ \begin{array}{l} -\left([\mathrm{HA^-}]_0 + 2[\mathrm{A^{2-}}]_0\right)[\mathrm{H^+}]^3 + \left(K_{1a}\left([\mathrm{H_2A}]_0 - [\mathrm{A^{2-}}]_0\right) + K_W\right)[\mathrm{H^+}]^2 \\ + \left(K_{1a}K_{2a}\left(2[\mathrm{H_2A}]_0 + [\mathrm{HA^-}]_0\right) + K_{1a}K_W\right)[\mathrm{H^+}] + K_{1a}K_{2a}K_W \end{array} \right\} \end{bmatrix} \qquad (17)$$

Equation 17 provides us with expression for $[H_2A]$, $[HA^-]$, $[A^{2-}]$, and $[OH^-]$ as a function of the parameter concentration $[H_3O^+]$, but also a expression for finding $[H_3O^+]$ as a function of the initial system composition as given in the following:

$$[H^+]^4 + \left(K_{1a} + [HA^-]_0 + 2[A^{2-}]_0\right)[H^+]^3 + \left(K_{1a}\left([A^{2-}]_0 - [H_2A]_0\right) + K_{1a}K_{2a} - K_W\right)[H^+]^2 + \left(K_{1a}K_{2a}\left(-2[H_2A]_0 - [HA^-]_0\right) - K_{1a}K_W\right)[H^+] - K_{1a}K_{2a}K_W = 0 \quad (18)$$

The method exemplified in the former sections shows how to deal with a simple acid–base equilibria problem in a general manner. Systems of greater complexity, for instance, mixtures of different polyprotic acids can be handled in the same way. The most practically complicated operations are the matrix inversions; however, they can easily be performed by use of symbolic manipulation software such as *Matematica, Maple*, or *MathCAD*.

It should be emphasized, that for some systems of acid–base and complex equilibria it will not be possible to express the set of mass action equations as a matrix product of a equilibrium matrix and a reduced concentration vector. For those situations it will usually be possible to separate the system into smaller sets that can be treated as described. An example of this situation is the system of equilibria describing the solubility of cadmium(II) sulfide in hydrochloric acid, where the solubility product of CdS, makes it impossible to treat both complexation equilibria and acid/base equilibria simultaneously.

Acknowledgment. Comments from Dr.s Jesper Bendix and Høgni Weihe in connection with this work are greatly appreciated.

Appendix 1. The Extent of Reaction ξ. Stoichiometry as the relationship between the amounts of substances that react together in a particular chemical reaction and the amounts of products that are formed [5] represents one of the most fundamental concepts in chemistry. All stoichiometric considerations are based on the the axiom of stoichiometry:

For any chemical transformation that take place in a closed system the sort and number of atoms is preserved.

This axiom will for the chemist be an intuitive part of his way of thinking. Because, the concept of "extent of reaction" is intimately related to the axiom of stoichiometry, it is often introduced with no direct justification. In the following, the concept of "extent of reaction" will be introduced in a deductive way starting from the axiom of stoichiometry.

We consider a system consisting of N distinct reaction entities, $\{B_1, B_2, B_3, B_4, B_N\}$, which enter into M independent reactions, $\{\rho_1, \rho_2, \rho_3, \rho_4, \rho_M\}$.

Any balanced chemical equation obeys the axiom of stoichiometry which may be stated as

$$\forall \rho_i : \sum_{j=1}^{N} \upsilon_{B_j,\rho_i} M_{B_j} = 0 \quad (19)$$

where M_{B_j} is the molar masse of B_j, and υ_{B_j,ρ_i} the stoichiometric coefficient with which the reaction entity, B_j participates in reaction ρ_i. Equation 19 is the general chemical equation for reaction ρ_i. Conventionally $\upsilon_{B_j,\rho_i} > 0$ indicate that reaction entity is being formed (products) and $\upsilon_{B_j,\rho_i} < 0$ that reaction entity is being consumed (reactants). According to the reaction equation 19 for the reaction, ρ_i, the molar amount, n_{B_j} of the N reaction entities will not be able to vary independently of each other without violating the axiom of stoichiometry. Therefore, a single extensive parameter for the reaction, ρ_i, must exist, which connect the way the molar amount of every reaction entities can vary at reaction. This parameter is given the symbol ξ and is named "extent of reaction".

The molar amount, n_{B_j,ρ_i} of an entity B_j with entering reaction ρ_i is a function of ξ_{ρ_i} and as consequence the differential change, dn_{B_j,ρ_i} is given as

$$dn_{B_j,\rho_i} = \frac{dn_{B_j,\rho_i}}{d\xi_{\rho_i}} d\xi_{\rho_i} \quad (20)$$

The differential change, dn_{B_j,ρ_i} is related to the differential change of mass, dm_{B_j,ρ_i} and given by

$$dm_{B_j,\rho_i} = dn_{B_j,\rho_i} M_{B_j} \quad (21)$$

Combining 21 with 20 and then performing a summation over all reaction entities gives

$$0 = \sum_{j=1}^{N} dm_{B_j,\rho_i} = \sum_{j=1}^{N} \frac{dn_{B_j,\rho_i}}{d\xi_{\rho_i}} d\xi_i M_{B_j} = d\xi_i \sum_{j=1}^{N} \frac{dn_{B_j,\rho_i}}{d\xi_{\rho_i}} M_{B_j} \Rightarrow \sum_{j=1}^{N} \frac{dn_{B_j,\rho_i}}{d\xi_{\rho_i}} M_{B_j} = 0 \quad (22)$$

We have here used the formulation of the axiom of stoichiometry

$$\forall \rho_i : \sum_{j=1}^{N} dm_{B_j,\rho_i} = 0 .$$

Equation 22 must be obeyed simultaneously with 19:

$$\sum_{j=1}^{N} \upsilon_{B_j,\rho_i} M_{B_j} = \sum_{j=1}^{N} \frac{dn_{B_j,\rho_i}}{d\xi_{\rho_i}} M_{B_j} \Rightarrow \upsilon_{B_j,\rho_i} = \frac{dn_{B_j,\rho_i}}{d\xi_{\rho_i}} \quad (23)$$

This equation gives an important relation between the stoichiometric coefficient and the extent of reaction. From eq 23 the variation of the molar amount of the reaction entity, B_j, with the reaction extent, ξ_{ρ_i} is given by integration from the initial state to the state (e.g., equilibrium) in consideration:

$$\upsilon_{B_j,\rho_i} = \frac{dn_{B_j,\rho_i}}{d\xi_{\rho_i}} \Rightarrow \int_{n^0_{B_j,\rho_i}}^{n_{B_j,\rho_i}} dn_{B_j,\rho_i} = \int_0^{\xi_{\rho_i}} \upsilon_{B_j,\rho_i} d\xi_{\rho_i} \Rightarrow n_{B_j,\rho_i} = n^0_{B_j,\rho_i} + \upsilon_{B_j,\rho_i}\xi_{\rho_i} \quad (24)$$

© 2009 *The Chemical Educator*, S1430-4171(09)32206-6, Published on Web 06/05/2009, 10.1333/s00897092206a, 14090091tb.pdf

Equation 24 gives the interpretation of the extent of reaction as a measure of progress of the chemical reaction [6].

For the system with M independent reactions the change in molar amount, $n_{\mathrm{B}_j}^{Total}$ of a reaction entity is given as a sum over all extent of reaction:

$$n_{\mathrm{B}_j}^{Total} = n_{\mathrm{B}_j,0}^{Total} + \sum_{i=1}^{M} \upsilon_{\mathrm{B}_j,\rho_i} \xi_{\rho_i} \quad (25)$$

Equation 25 for the change in molar amounts of entities is converted to an expression in molar concentration, by division with the system volume V_{sys}.

$$\frac{n_{\mathrm{B}_j}^{Total}}{V_{Sys.}} = \frac{n_{\mathrm{B}_j,0}^{Total} + \sum_{i=1}^{M} \upsilon_{\mathrm{B}_j,\rho_i} \xi_{\rho_i}}{V_{Sys.}} = \frac{n_{\mathrm{B}_j,0}^{Total}}{V_{Sys.}} + \sum_{i=1}^{M} \upsilon_{\mathrm{B}_j,\rho_i} \left(\frac{\xi_{\rho_i}}{V_{Sys.}} \right) \quad (26)$$

$$\Rightarrow \quad [\mathrm{B}_j] = [\mathrm{B}_j]_0 + \sum_{i=1}^{M} \upsilon_{\mathrm{B}_j,\rho_i} \hat{\xi}_i$$

where

$$\hat{\xi}_{\rho_i} \equiv \frac{\xi_{\rho_i}}{V_{Sys.}}$$

Appendix 2. General Treatment of Equilibria. For a system obeying eq 19, the thermodynamic equilibrium constant for reaction ρ_i named K_i^{o} is defined by

$$K_i^{\circ} \equiv \prod_{j=1}^{N} a_{\mathrm{B}_j}^{\ \upsilon_{\mathrm{B}_j,\rho_i}} \quad (27)$$

where a_{B_j} is the activity of the reaction entities, B_j.

The activity, a_{B_j}, is related to the molar concentration $[\mathrm{B}_j]$ by the activity coefficient, γ_{B_j} :

$$a_{\mathrm{B}_j} = \gamma_{\mathrm{B}_j} \frac{[\mathrm{B}_j]}{c^{\ominus}} \quad (28)$$

Insertion of 28 into 27 gives:

$$K_i^{\circ} \equiv \prod_{j=1}^{N} a_{\mathrm{B}_j}^{\ \upsilon_{\mathrm{B}_j,\rho_i}} = \prod_{j=1}^{N} \left(\gamma_{\mathrm{B}_j} \frac{[\mathrm{B}_j]}{c^{\ominus}} \right)^{\upsilon_{\mathrm{B}_j,\rho_i}} = \prod_{j=1}^{N} [\mathrm{B}_j]^{\upsilon_{\mathrm{B}_j,\rho_i}} \prod_{j=1}^{N} \left(\frac{\gamma_{\mathrm{B}_j}}{c^{\ominus}} \right)^{\upsilon_{\mathrm{B}_j,\rho_i}} \quad (29)$$

From 29 the stoichiometric equilibrium constant, K_i is defined as

$$K_i \equiv \prod_{j=1}^{N} [\mathrm{B}_j]^{\upsilon_{\mathrm{B}_j,\rho_i}} \quad (30)$$

It must be emphasized that only the thermodynamic equilibrium constant 27 is a true constant. However, traditionally, the stoichiometric equilibrium constant is the one used in equilibrium calculations both in theoretical derivations and in practice. All equations derived using the stoichiometric equilibrium constant can, if necessary, be converted to the true thermodynamic equilibrium constant by use of eq 29.

References and Notes

1. Auerbach, Fr.; Smolczyk, E. *Z. physik. Chem.* **1924**, *110*, 65–141.
2. de Levie, R. *Aqueous Acid-Base Equilibria and Titrations*; Oxford Chemistry Primer; Oxford University Press, 2006.
3. Prigogine, I.; Defay, R. translated by Everett, D. H. *Chemical Thermodynamics*; Longmans Green and Co., 1954.
4. Garst, J. F. *J. Chem. Educ.* **1974**, *51*, 194–195.
5. IUPAC Compendium of Chemical Terminology, Electronic version, http://goldbook.iupac.org/S06026.html. (accessed June 2009).
6. IUPAC Compendium of Chemical Terminology, Electronic version, http://goldbook.iupac.org/E02283.html (accessed June 2009).

DEPARTMENT OF CHEMISTRY
PHD THESIS 2012
ISBN 978-87-7611-511-1

TORBEN BIRK

FLUORIDE AS LIGAND. Chemistry of some new terminal and bridged systems

Academic advisor: Jesper Bendix
Submitted March 30, 2012
